# Proteome degradation in fossils: investigating the longevity of protein survival in ancient bone

**DOI:** 10.1002/rcm.6821

**Published:** 2014-02-12

**Authors:** Caroline Wadsworth, Mike Buckley

**Affiliations:** Faculty of Life Sciences, Manchester Institute of Biotechnology131 Princess Street, Manchester, M1 7DN, UK

## Abstract

**RATIONALE:**

We report the use of proteomics techniques to study how the fossil bone proteome changes in complexity over one million years.

**METHODS:**

We include the attempted use of a previously unreported methodology in proteome research, to remove the dominant bone collagens using bacterial collagenase as well as conventional shotgun proteomics methodology following digestion with the protease trypsin. In this study we expand upon a set of 19 bovine sub-fossil specimens ranging over one and a half million years that had previously been shown to possess collagen, using a total of 46 LTQ-Orbitrap liquid chromatography/tandem mass spectrometry (LC/MS/MS) analyses containing 462,186 precursor ion analyses.

**RESULTS:**

Although many types of proteins can typically be identified in recent bone, in degraded bone we observe a rapid loss of lower abundance proteins. Abundant serum proteins such as serum albumin and alpha-2-HS-glycoprotein appear to be more easily recovered in ancient bone, both being identified in specimens dating to the Early Pleistocene, the earliest period tested in this study. Proteins belonging to the leucine-rich repeat family such as lumican, biglycan and chondroadherin also survive well, possibly because of their interactions with bone collagen.

**CONCLUSIONS:**

Of these 'survivor proteins' A2HSG shows a remarkable amount of sequence variation, making it potentially one of the most useful proteins to study for species identification and phylogenetic inference in archaeological and palaeontological bone.

The survival of biomolecules in ancient tissues has been of great interest for their potential in recovering phylogenetic information for over 30 years. Since the early 1980s, such protein-derived information was retrieved using immunological approaches,^1^,^2^ methodology that continues in use even recently,^3^,^4^ despite notorious issues with false-positive results due to cross-reactivity with likely contaminants (e.g., fungi).^5^ The criticism that immunological methods are difficult to apply to fossil samples because the assays are pushed to their limits and are prone to yield false-positive results induced doubt into the authenticity of previous results.^6^ Early interest in protein survival focused on collagen, because of its dominance in bone but, during the 1980s, focus switched to the study of albumin,^7^ because it was one of the most abundant noncollagenous proteins (NCPs) that survived in bone, where NCPs were reportedly capable of surviving longer than collagen.^8^ For much of the 1990s and early 2000s, this focus shifted onto the protein osteocalcin (OC), believed capable of surviving longer than other proteins due to its mineral-binding properties.^9^ Osteocalcin was even thought to survive in Mesozoic dinosaurs until the aforementioned issues with immunological studies of fossil proteins were realised.^10^ At the turn of the century reports for identifications of glycoprotein survival in dinosaur fossils were made,^11^ and investigations appeared to then turn full-circle when collagen was once again the target protein, also reportedly extracted from dinosaur fossils.^12^,^13^ However, with the advent of new approaches to sequencing, collagen was found to be more species-specific than previously known,^14^,^15^ and so potentially of great value in inferring phylogenies.^16^ Over the past few decades the majority of studies have been targeted at particular proteins, due to the nature of the methods used, and so relative survival rates could not be readily compared. Gel electrophoresis has been used to study complex mixtures of proteins in well-preserved archaeological bone,^17^ but in typical degraded archaeological and palaeontological samples the degraded collagen is known to smear down throughout the gel rendering the identification of proteins within this complex mixture difficult.^18^,^19^

Here we report the use of proteomics techniques to study how the complex mixture of proteins present in the bone extracellular matrix reduces in complexity over hundreds of thousands of years. To do so we use conventional proteomics methodologies,^20^,^21^ as well as a previously unreported methodology in proteome research involving the removal of the dominant bone protein collagen using bacterial collagenase, which cleaves the collagen following Pro/Hyp in the sequence Pro/Hyp-Xaa-Gly (where Xaa is any amino acid) producing peptides of molecular weight <700 Da and is reported to display no activity for other proteins.^22^,^23^ We expand upon a set of bovine sub-fossil specimens from Britain ranging approximately one and a half million years that had previously been shown to possess collagen.^24^ As the thermal history of a sample is considered one of the most important aspects of protein degradation,^25^ we include two bovine specimens from a younger site (10,000 years old) in a much warmer climate (Cyprus) for comparison.

## EXPERIMENTAL

### Bone samples

A set of 19 ancient (archaeological/palaeontological) bovine bone samples ranging in age from approximately 4 thousand to 1.5 million years old were selected for analysis. Most samples were from British sites, with the exception of two samples from a 10,000 year old site, Ais Yiorkis, in Cyprus (AY2 and AY5), included to represent proteome preservation in warm climates. Samples from British sites include three from Kirkdale Cave, North Yorkshire (KC2, KC4, KC6; ∼130 Ka), and one from each of the following; Stonehenge, Wiltshire (UI3; ∼4 Ka), North Ferriby, East Yorkshire (NF1; ∼6 Ka), Carsington Pasture Cave, Derbyshire (AuCPC; ∼6 Ka), Windy Knoll cave, Peak District (WK1; ∼40-50 Ka), Titan Shaft, Titan Caves, Peak District (TSH; ∼45 Ka), and Happisburgh (Site 3), Norfolk, (HSB3; ∼900 Ka). Six samples were from West Runton, Norfolk (WR6, WR10 and WR15-18; ∼650 Ka–1.5 Ma), four of which come from the West Runton forest bed (WR15-18) and two from the Weybourne/Wroxham crag layer (WR6 and WR10; ∼1.5 Ma). One sample dredged from the North Sea (NS2) and one from Lynford Quarry (LQ1), both estimated at ∼20–60 Ka, were also included.^26^ Modern cow (*Bos taurus*) bone was obtained from a local butcher (York, UK).

### Materials

Hydrochloric acid (HCl) and acetonitrile were purchased from Fisher Scientific (UK), dithiothreitol (DTT), formic acid, iodoacetamide (IAM), trifluoroacetic acid (TFA) and guanidine hydrochloride (GuHCl) from Sigma-Aldritch (UK), ammonium bicarbonate (ABC) from Fluka Analytical (via Sigma-Aldritch, UK) and Tris was purchased from Formedium (UK).

### Protein extraction and enzymatic digestion

#### HCl demineralisation

Bone powder was obtained from each sample by diamond-tipped Dremel drilling. Samples were taken from the bone interior wherever possible to reduce the risk of introducing environmental contaminants from the bone surface. Approximately 50 mg bone powder from each sample was weighed into a 1.5 mL microtube and demineralised with 1 mL 0.6 M HCl for 18 h at 4 °C. After incubation samples were spun for 1 min at 14,000 *g* in a benchtop microfuge, the supernatant was collected and stored at −20 °C ('acid-soluble fraction') and the acid-insoluble pellet washed three times with 1 mL distilled water.

#### Extraction of NCPs

NCPs were extracted following the method described by Jiang *et al*.^20^ in which the acid-insoluble pellets obtained after HCl demineralisation were incubated for 72 h at 4 °C in a buffer containing 100 mM Tris and 6 M GuHCl at pH 7.4. After 72 h the samples were centrifuged for 1 min at 14,000 *g* in a benchtop microfuge, the supernatant collected ('acid-insoluble fraction') and the remaining pellets stored at −20 °C.

#### Trypsin digest

The acid-insoluble fractions were buffer exchanged into 50 mM ABC using 10 k molecular weight cut-off (MWCO) Vivaspin spin columns, reduced by incubating for 10 min at 60 °C in the presence of 5 mM DTT (final concentration) and alkylated with 15 mM IAM (final concentration) at room temperature in the dark for 45 min. Following reduction and alkylation, tryptic digests were carried out by incubating the sample overnight at 37 °C with 0.1 µg trypsin (sequencing grade; Promega UK).

#### Peptide concentration

Samples were acidified to a final concentration of 0.1% TFA and desalted, purified and concentrated with 100 μL C18 reversed-phase Zip-Tips (Millpore) as follows: each tip was prepared with one volume (100 μL) of a solution of 50% acetonitrile, 0.1% TFA and 49.9% distilled water (v/v) then washed with two volumes of a solution containing 0.1% TFA and 99.9% distilled water (v/v). Acidified samples were loaded onto the tip and aspirated through the membrane five times to ensure the maximum number of peptides were captured from the solution before washing the membrane with two volumes of a 0.1% TFA and 99.9% distilled water solution (v/v). Proteins were finally eluted from the membrane with one volume of a 50% acetonitrile, 0.1% TFA and 49.9% distilled water solution (v/v). Following purification the samples were completely lyophilised in a centrifugal evaporator and resuspended in 20 μL 0.1% TFA in preparation for LC/MS/MS analysis.

#### Bacterial collagenase digestion

A sub-sample of seven ancient samples (HSB3, KC2, KC6, TSH, NS2, AY5 and WK1) and one modern cow sample were treated using bacterial collagenase to determine whether enzymatically digesting the dominant well-preserved collagen in ancient samples would improve the number of detectable NCPs. Treatment with bacterial collagenase was used as the sole NCP extraction method for these samples, as an alternative to the GuHCl NCP extraction method. Samples were demineralised with 0.6 M HCl and the pellet washed with 3 × 1 mL of distilled water as described in the previous sections, then type 3 bacterial collagenase (Sigma) was added to each acid-insoluble pellet according to the manufacturer's recommendation and incubated at 37 °C for 5 h. After incubation, samples were spun at 14 000 *g* for 1 min in a benchtop microfuge and the supernatant was removed, completely lyophilised in a centrifugal evaporator and resuspended in 50 mM ABC in preparation for a trypsin digest. All samples were reduced and alkylated, digested with trypsin (Promega sequencing grade) acidified and desalted with C18 reversed-phase Zip-Tips (Millpore) as described above.

### Mass spectrometry

Digested samples were analysed by LC/MS/MS using an UltiMate® 3000 Rapid Separation LC (RSLC, Dionex Corporation, Sunnyvale, CA, USA) coupled to an Orbitrap Elite (Thermo Fisher Scientific, Waltham, MA, USA) mass spectrometer (120 k resolution, full scan, positive mode, normal mass range 350–1500). Peptides in the sample were separated on a Ethylene Bridged Hybrid (BEH) C18 analytical column (75 mm × 250 µm i.d., 1.7 μM; Waters) using a gradient from 92% A (0.1% formic acid in water) and 8% B (0.1% formic acid in acetonitrile) to 33% B in 44 min at a flow rate of 300 nL min^–1^. Peptides were then automatically selected for fragmentation by data-dependent analysis; six MS/MS scans (Velos ion trap, product ion scans, rapid scan rate, Centroid data; scan event: 500 count minimum signal threshold, top 6) were acquired per cycle, dynamic exclusion was employed, and 1 repeat scan (i.e. two MS/MS scans total) was acquired in a 30 s repeat duration with that precursor being excluded for the subsequent 30 s (activation: collision-induced dissociation (CID), 2+ default charge state, 2 *m/z* isolation width, 35 eV normalized collision energy, 0.25 Activation Q, 10.0 ms activation time).

### Data handling

Peptide masses obtained via LC/MS/MS were searched against the Swissprot database for matches to primary protein sequences using the Mascot search engine (version 2.2.0.6; Matrix Science, London, UK). Each search included the fixed carbamidomethyl modification of cysteine (+57.02 Da) and the variable modifications for deamidation (asparagine to glutamine, +0.98 Da), serine and threonine phosphorylation (+79.99 Da), and oxidation of lysine, proline and methionine residues (all +15.99 Da) to account for PTMs and diagenetic alterations; the oxidation of lysine and proline is equivalent to hydroxylation. Enzyme specificity was limited to trypsin with up to 2 missed cleavages allowed, mass tolerances were set at 5 ppm for the precursor ions and 0.5 Daltons for the fragment ions, all spectra were considered as having either 2+ or 3+ precursors and the peptide ion score cut off was set at 40. Resulting peptide matches from each sample were manually examined and proteins from the relevant genus (*Bos* or *Bison*) with at least two unique high-confidence peptide matches were collated to construct proteomes. Decoy (false-positive) rates were typically ∼4%, similar to those previously published for ancient proteome data (e.g.^21^,^22^). Error tolerant searches were also carried out for each sample using the fixed carbamidomethyl modification of cysteine as well as the variable modifications for serine and threonine phosphorylation (+79.99 Da) and proline oxidation to allow for single amino acid substitutions between *Bos* (present in the public databases) and *Bison* (not publicly available), as well as to check for unusual modifications potentially induced by decay.

An ancient bone proteome 'summary' was created by collating a list of all bovine proteins that were identified at least once in any of the ancient samples (according to the above criteria). The online STRING (Search Tool for the Retrieval of Interacting Genes) database was then used to create a network of functional relationships between all identified proteins.

## RESULTS

### Comparison of bacterial collagenase and GuHCl NCP extraction methods

**Figure 1:**
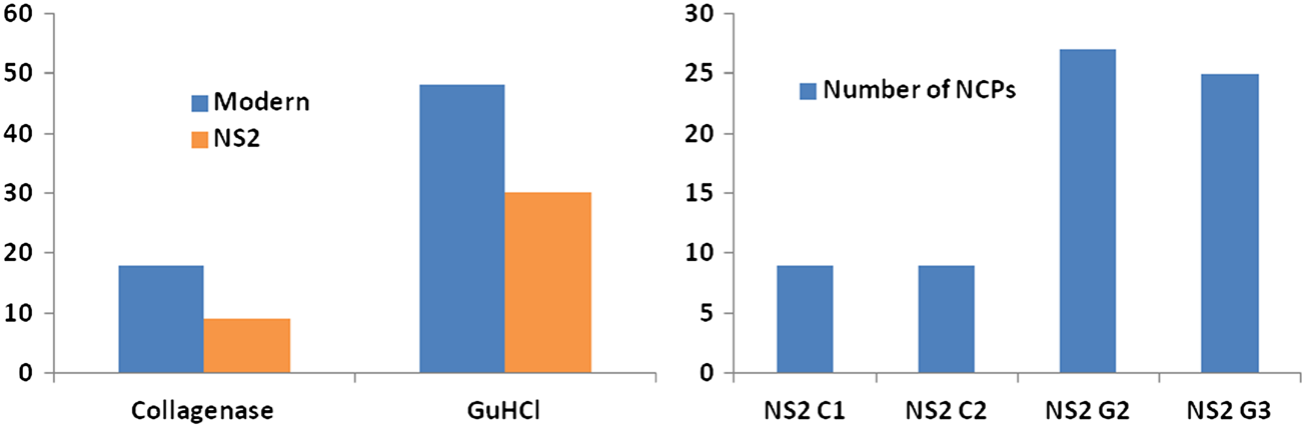
Comparison of the total number of NCPs extracted using both the bacterial collagenase and GuHCl extraction methods, illustrating the reduced proteome complexity obtained from the bacterial collagenase method compared to the GuHCl extraction method (Supplementary Table S1, see Supporting Information).^20^ The chart on the left compares the total number of NCPs recovered from a modern cow sample (*Bos taurus*; blue columns) and the NS2 (orange columns) using both extraction methods, and the chart on the right compares the number of NCPs identified from NS2 in four different extractions; C1 and C2 using the bacterial collagenase method (batches 1 and 2) and G2 and G3 (batches 2 and 3) using the GuHCl extraction method.

A potential problem with ancient proteomics is the fact that the exceptionally well-preserved collagen molecules present in an archaeological bone could be masking the presence of potentially more informative but less abundant NCPs. Reducing the amount of collagen present in a sample prior to proteomic analysis could allow NCPs to be more easily detectable, and one way to achieve this would be to enzymatically digest collagen molecules using bacterial collagenase. To test this, one modern cattle bone and seven archaeological bovine bones were treated with bacterial collagenase prior to proteomic analysis, and the proteomes obtained for each sample ('C' in Table 1) compared with those obtained via the GuHCl buffer NCP extraction method ('G' in Table 1) described in Jiang *et al*.^20^ In the modern sample it appears that treatment with bacterial collagenase does reduce the number of matched collagen peptides as the more abundant collagens (namely collagen alpha-2 (I) and collagen alpha-1 (I)) are removed as the top scoring protein matches on the Mascot database searches. However, this was unexpectedly accompanied by a significant loss in proteome complexity with only 18 NCPs identifiable in the modern sample treated with bacterial collagenase compared with 48 NCPs obtained from the modern sample via the GuHCl NCP extraction method (see Fig. 1).

**Table:** Sample information and number of NCPs identified per analysis

Sample name	Site name	Approximate age	Number of NCPs identified*
UI3^a^	Durrington Walls, Wiltshire	4 Ka	11 (G)
NF1^a^	North Ferriby, East Yorkshire	6 Ka	27 (G)
AuCPC^a^	Carsington Pasture Cave, Derbyshire	6 Ka	25 (G)
AY2^a^	Ais Yiorkis, Cyprus	10 Ka	0
AY5^a^	Ais Yiorkis, Cyprus	10 Ka	0 (C), 8 (G)
LQ1^b^	Lynford Quarry, Norfolk	20–60 Ka	17 (G)
NS2^b^	North Sea	20–60 Ka	9 (C), 30 (G)
WK1^b^	Windy Knoll Cave, Peak District	40–50 Ka	0 (C), 2 (G)
TSH^b^	Titan Shaft, Titan Cave, Peak District	45 Ka	1 (C), 0 (G)
KC2^a^	Kirkdale Cave, North Yorkshire	130 Ka	0
KC4	Kirkdale Cave, North Yorkshire	130 Ka	0
KC6	Kirkdale Cave, North Yorkshire	130 Ka	2 (C), 1 (G)
WR15	West Runton (forest bed), Norfolk	650 Ka	2 (G)
WR16	West Runton (forest bed), Norfolk	650 Ka	1 (G)
WR17	West Runton (forest bed), Norfolk	650 Ka	0
WR18	West Runton (forest bed), Norfolk	650 Ka	1 (G)
HSB3	Happisburgh, Norfolk	900 Ka	1 (C), 2 (G), 7 (100 mg)
WR6	West Runton (forest bed), Norfolk	650 Ka	0
WRC10	West Runton (Weybourne crag), Norfolk	1.5 Ma	0

Where known, Bos and Bison are superscripted: a and b, respectively.

* 'G' denotes Jiang *et al*.^20^ GuHCl method, 'C' denotes bacterial collagenase method.

Conversely, bacterial collagenase digestion of all seven archaeological samples failed to remove collagens as the top scoring proteins in Mascot searches and produced proteomes with much lower complexity than those obtained using the GuHCl NCP extraction method. Three of the seven samples (KC2, AY5 and WK1) had no identifiable NCPs after bacterial collagenase treatment; however, collagen alpha-2 (I) and collagen alpha-1 (I) were still detectable as the top scoring protein matches. NCPs were identified in the proteomes of four samples (HSB3, KC6, TSH and NS2) but in very low numbers (Table 1). Three of these four samples contained both collagen as well as NCPs (the exception being HSB3 in which no collagen peptides were matched in the sample digested with bacterial collagenase).

### Change in proteome complexity through time

Although it is known that proteins survive for longer than DNA and are estimated to be able to survive up to a million years, little is known about protein degradation over time. One aim of this study was to determine an approximate temporal limit for the survival of NCPs in archaeological bone from a temperate climate in comparison to collagen. A secondary aim was to identify which, if any, of these proteins may be more useful for constructing phylogenies and species identification of ancient organisms than collagen. In order to do this proteomes were obtained from 19 archaeological bone samples predominantly from the UK ranging in age from 4000 years old to 1.5 million years old.

A total of 50 unique proteins have been identified in these 19 samples collectively. Of these, 44 are NCPs and six are collagen chains. The two type 1 collagen chains, alpha-2 (I) and collagen alpha-1 (I), are present in every sample including six of the seven samples digested with bacterial collagenase; the exception being HSB3 which contained no collagen peptides (we know from previous work^24^ that type 1 collagen remains the dominant protein in this sample prior to the collagenase digest). The most complex ancient proteomes obtained using the GuHCl extraction method were those from NS2, NF1 and AuCPC, having 30, 27 and 25 identified NCPs in total. Of the 19 samples, 7 contained only collagen and keratin peptides (AY2, TSH, KC2, KC4, WR17, WR6 and WRC10) and 12 samples (AY5, UI3, HSB3, NF1, LQ1, NS2, AuCPC, WR15, WR16, WR18, KC6 and WK1) contained NCPs. In general, it appears that proteome complexity is inversely proportional to age, as most of the samples older than 20,000 years contain predominantly collagen peptides, with only five of these older samples (HSB3, WR15, WR16, WR18 and WK1) containing between one and two NCPs (see Supporting Information). Two NCPs were identified in HSB3 (albumin and fibronectin), WR15 (serum albumin and junction plakoglobulin) and WK1 (biglycan and A2HSG) and one NCP was identified in both WR16 (serum albumin) and WR18 (chondroadherin). A2HSG, biglycan, chondroadherin, lumican, osteomodulin, PEDF and serum albumin were also detected in HSB3 without bacterial collagenase treatment when the sample size was doubled to 100 mg bone powder. Of these NCPs, albumin, prothrombin, PEDF, chondroadherin and A2HSG are also found in many of the younger samples; however, unsurprisingly, the younger samples contained more identifiable peptides for each protein. LQ1 and NS2 appear to be exceptions as both are estimated to be between 20–60,000 years old but they have well-preserved and relatively complex proteomes (17 and 30 NCPs identified, respectively).

**Figure 2:**
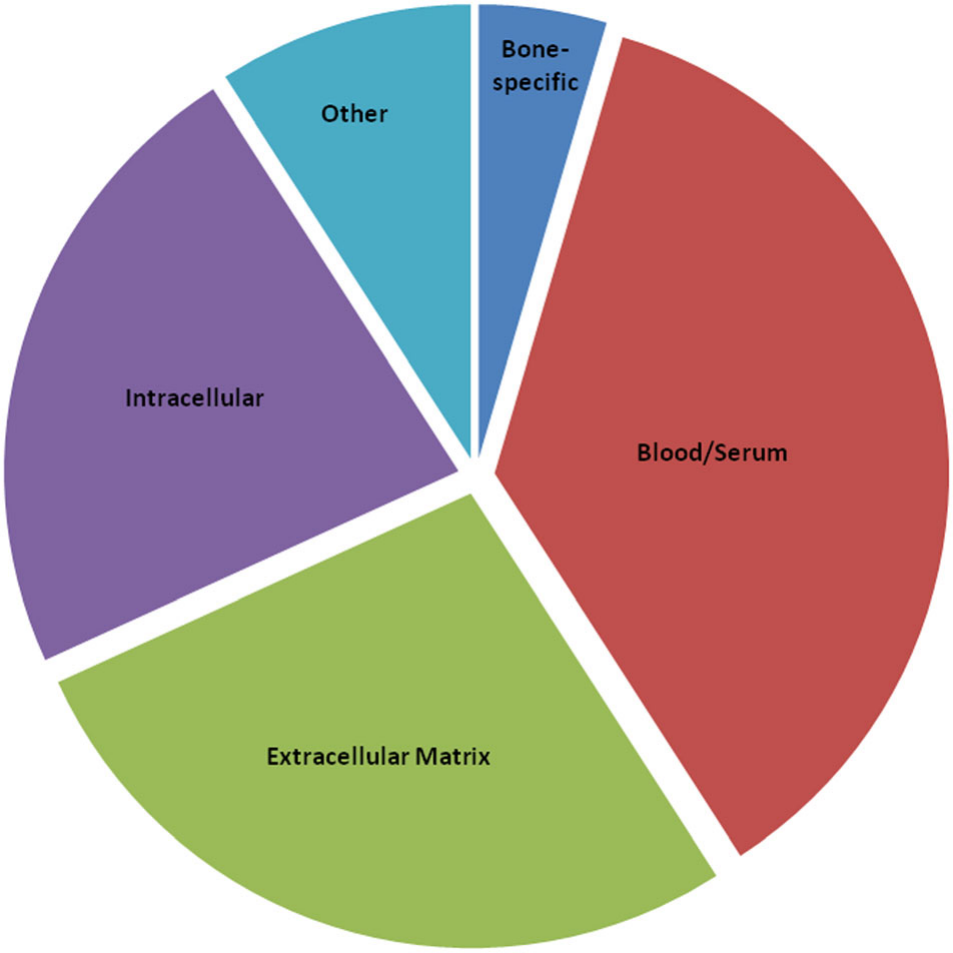
Proportion of matched proteins (n=44) from different sources preserved within the ancient bone samples in this study. Proteins specific to blood/serum make up 36.3% of the total, non-collagenous ECM proteins 27.2%, intracellular proteins 22.7% and only 4.5% were specific to bone.

Of the 44 total NCPs identified in the ancient samples (Fig. 2), the majority (16) can be classified as blood or serum proteins, 12 are non-collagenous extracellular matrix (ECM) proteins, 10 are intracellular and only two (SPARC (osteonectin) and osteomodulin) are only found in bone (see Table 2). The most common proteins identified include serum albumin and A2HSG, biglycan, chondroadherin, pigment epithelium derived factor (PEDF), lumican, and prothrombin.

**Table 2:** Summary list of proteins identified including the samples they are present in and their biological source derived from Supplementary Table S1; additional single peptide matches are also given in Supplementary Table S2 (see Supporting Information). The majority of proteins identified are found in blood/serum or the ECM with only two proteins being specific to bone

Protein	Samples identified in	Biological location
Collagen alpha-1 (I)	All	Bone
Collagen alpha-2 (I)	All	Bone
Biglycan	NS2, NF1, AuCPC, WK1, AY5, UI3, LQ1, HSB3 (100 mg)	ECM
Alpha-2-HS-glycoprotein	NS2, NF1, AuCPC, KC6, WK1, AY5, UI3, LQ1, HSB3 (100 mg)	Plasma
Pigment epithelium derived factor	NS2, NF1, AuCPC, AY5, UI3, LQ1, HSB3 (100 mg)	ECM
Complement C3	NS2, NF1, AuCPC, AY5, UI3, LQ1	Plasma
Chondroadherin	NS2, NF1, AuCPC, AY5, UI3, WR18, LQ1, HSB3 (100 mg)	Cartilage
Serum albumin	NS2, KC6, TSH, NF1, AuCPC, AY5, UI3, HSB3, WR15, WR16, LQ1, HSB3 (100 mg)	Plasma
Apolipoprotein A-I	NS2, NF1, AuCPC, UI3	Plasma
Prothrombin	NS2, NF1, AuCPC, UI3, LQ1	Plasma
SPARC	NS2, NF1, AuCPC, UI3, LQ1	Bone matrix
Lumican	NS2, NF1, AuCPC, AY5, UI3, LQ1, HSB3 (100 mg)	ECM
Nucleobindin-1	NS2, NF1, AuCPC, UI3	Skeletal muscle
Complement C9	NS2, NF1, UI3, AuCPC	Plasma
Olfactomedin-like protein 3	NS2, NF1, AuCPC	Plasma
Asporin	NS2, NF1, AY5, AuCPC	Cartilage
Tetranectin	NS2, NF1, AuCPC, UI3, LQ1	Plasma
Collagen alpha-2 (XI)	NS2, NF1, AuCPC, AY5, UI3, LQ1	Cartilage
72 kDa type IV collagenase	NS2, NF1, AuCPC	ECM
Mimecan	NS2	ECM
Serine protease HTRA1	NS2, NF1, AuCPC, UI3, LQ1	Cytoplasm
Antithrombin-III	NS2, NF1, AuCPC, LQ1	ECM
Decorin	NS2, AuCPC, UI3	ECM
Secreted phosphoprotein 24	NS2	Plasma
Collagen alpha-1 (III)	NS2, KC6, NF1, AuCPC, WK1	Tissue – skin, lungs and walls of blood vessels
Coagulation factor IX	NS2, NF1, AuCPC, UI3, LQ1	Plasma
Collagen alpha-1 (XI)	NS2, NF1, AuCPC, LQ1	Cartilage
Osteomodulin	NS2, NF1, AuCPC, HSB3 (100 mg)	Bone matrix
Vitamin D binding protein	NS2, NF1, AuCPC	Plasma
Vimentin	NS2	Cytoplasm
Fibrinogen beta chain	NS2	Plasma
Vitrin	NS2, NF1	ECM
Vitamin K dependent protein S	NS2, NF1, AuCPC, LQ1	Plasma
Ezrin	NS2	Cytoplasm
Radixin	NS2	Cytoplasm
Dermatopontin	NS2, NF1, LQ1	ECM
Collagen alpha-1 (II)	NS2, KC2, TSH, NF1, AY5, UI3, KC4, WR17, WR18, LQ1	Cartilage
Thrombospondin-1	NF1, AuCPC, NS2, LQ1	ER (cytoplasm)
Coagulation factor X	NF1, AuCPC	Plasma
Lysosomal alpha mannosidase	NF1, AuCPC	Lysosome (cytoplasm)
Alkaline phosphatase tissue nonspecific isozyme	AuCPC	Liver/bone/kidney
Serpin H1	NS2, AuCPC	ER lumen (cytoplasm)
Carboxypeptidase E	AuCPC	ECM
Complement C4	NS2, AuCPC	Plasma
Serine protease HTR4	AuCPC	ECM
Coagulation factor VII	NS2	Plasma
Endoplasmin	NS2	ER lumen (cytoplasm)
Junction plakoglobulin	WR15	Cytoplasm
DnaJ homolog subfamily C member 3	NS2	ER (cytoplasm)
Extracellular matrix protein 2	NS2	ECM

The majority of archaeological proteins (27 of 44) identified herein appear to survive at least into the Late Pleistocene (see Fig. 1); however, this appears to be due to the inclusion of two exceptionally well-preserved samples (LQ1 and NS2) dating to this period. One protein, coagulation factor X, was only detectable in samples dating to the Holocene which may indicate that it is more susceptible to degradation than other NCPs, or it is found in low concentrations in bone and is therefore difficult to detect in more ancient samples (although the other coagulation factor identified, coagulation factor IX, was present in samples dating from the Late Pleistocene). Three of the 44 proteins (collagens alpha-1 (II) and (III), chondroadherin and A2HSG), of which two (chondroadherin,and A2HSG ) are NCPs, were identified in samples from the Middle Pleistocene. Survival of these NCPs could be explained either by interactions with collagen molecules, presence in the extracellular matrix or presence in serum. Only two proteins were identifiable in samples from the Early Pleistocene, including albumin and both chains of type 1 collagen (collagen alpha-2 (I) and alpha-1 (I), both from the same protein molecule). Albumin is a serum protein whose survival may be mediated by high initial concentrations in bone tissue.

Fibronectin (FN1) and serum albumin were both identified in the HSB3 sample which dates from the early Pleistocene; however, fibronectin was not identified in any of the younger samples and both these proteins were only identified in one of the two HSB3 repeats. This highlights a problem with the study of ancient proteomes in that the matching of low-abundance proteins is not reproducible, especially when considering the less well-preserved NCPs which may have significantly fewer peptides present in each sample than NCPs such as serum albumin.

Alpha-2-HS-glycoprotein and albumin appear to be potentially the most useful of all the NCPs identified, being the only ones that were commonly found in many of the younger samples as well as both of the much older (∼650 Ka) West Runton forest bed sample (WR18) and ∼900 Ka year old (HSB3) samples (albumin only). Interestingly, A2HSG was only detectable in HSB3 by doubling the amount of bone powder used, perhaps suggesting that older samples are more susceptible to the potential problem of collagen masking much less abundant NCPs.

## DISCUSSION

In this study we expand upon a set of 19 bovine sub-fossil specimens ranging over one and a half million years that had previously been shown to possess collagen, using a total of 46 LC/MS (Orbitrap-Elite) analyses containing 462, 186 precursor ion analyses. Although collagen may be masking the presence of some of the low-abundance NCPs, it is difficult to remove via conventional means due to its diverse range in biochemical properties when hydrolysed, as is the case in degraded archaeological collagen.^27^ This results in collagen smearing throughout gels,^28^,^29^ and even its retention following other chromatographic separation techniques (e.g., anionic exchange, M. Buckley, unpublished data). We attempted to use bacterial collagenase to reduce the amount of collagen in each sample prior to conventional proteomics methodology; however, these attempts at improving NCP detection were unsuccessful. The very low complexity of the NS2 proteome after bacterial collagenase digestion is noteworthy as the GuHCl extraction method yields a proteome with almost four times as many NCPs for this sample. This reduced complexity could be due to the impurity of the purchased collagenase, which could potentially contain non-specific proteases such as pepsin that would cleave the already damaged ancient proteins. Alternatively, this could be due to the presence of the collagenase enzyme itself and its effects on the tryptic digest or its abundance affecting the subsequent LC/MS analyses.

Previous publications carrying out similar work have reported higher numbers of protein matches from NCP extractions on bone samples (2479 unique proteins from modern bone samples and 126 from an ancient mammoth sample).^20^,^21^ Due to the similarity in methods and even instrumentation used, we infer that the lower number of protein matches in our samples as compared with previous publications is most likely due to the lower starting sample size that we were confined to with some of the archaeological specimens. The types of proteins identified in this study agree in general with those expected from a bone extract as well as with those identified in other studies using ancient bone samples,^20^ the main difference being the majority of proteins identified herein are serum proteins rather than non-collagenous extracellular matrix proteins as reported elsewhere. This could be due to the initial high concentrations of serum proteins in bone, such as albumin, which is reportedly 50 times more concentrated in bone than serum due to binding with hydroxyapatite crystals.^28^ Despite this, the 44 NCPs we did observe cover the range of protein types of interest (e.g. abundant serum proteins such as albumin and A2HSG and extracellular matrix proteins associated with collagen such as biglycan), and allow for the identification of abundant (and thus more easily and reproducibly extracted) markers for phylogenetic information or species identification.

### Survivor proteins

The longest lived protein identified was type 1 collagen, with collagen alpha-1 (I) and alpha-2 (I) peptides being identified in every sample (the number of peptides identified being dependent on the preservation of the sample; i.e. the samples with the most complex proteomes yielded the most collagen peptides). Of the non-collagenous proteins, A2HSG, serum albumin and biglycan appear to be the longest lived proteins frequently identified in ancient bone (Table 2). Serum albumin and A2HSG are found in 11 and 8 samples, respectively, with sequence coverages ranging from 9–50% (albumin) and 12–54% (A2HSG) and biglycan is found in 7 samples with sequence coverages ranging from 11–57%.

### Types of NCPs most likely to survive

Many of the NCPs identified herein are commonly found in blood or serum (16 of the 44 identified proteins) or the extracellular matrix (12 of the 44 identified proteins) and only 2 were closely associated with bone matrix. In general this agrees with other studies investigating NCP survival in archaeological bone in which albumin and A2HSG have been detected using immunological methods.^29^–^34^

Non-collagenous proteins found in blood/serum or the ECM may be more detectable in archaeological bone than bone-specific proteins due to their abundance in the body during life. It has been suggested that fluids from decaying blood and soft tissues may permeate the surface of bones during putrefaction, causing unusually high concentrations of blood/serum and ECM proteins in bone tissue.^31^ The haematopoietic function of bone marrow may also play a part in the accumulation of blood and serum proteins in bone tissue, and it is thought that some NCPs can become incorporated into the bone matrix during bone formation. Given its suggested role in the initialisation of mineralisation this seems highly plausible for A2HSG which, although synthesised in the liver, is deposited in mineralising bone.^35^ The preservation of serum albumin, which is released from osteoblast cells,^36^ may also be due to a similar entrapment scenario within the forming bone but could be related to its much greater abundance; albumin is reportedly 50–100 times more concentrated in bone than serum due to its binding to hydroxyapatite crystals.^37^ Both A2HSG and serum albumin are exogenously derived, but known to bind to the hydroxyapatite bone mineral due to their acidic properties, further supporting the likelihood of this molecular entrapment.

**Figure 3:**
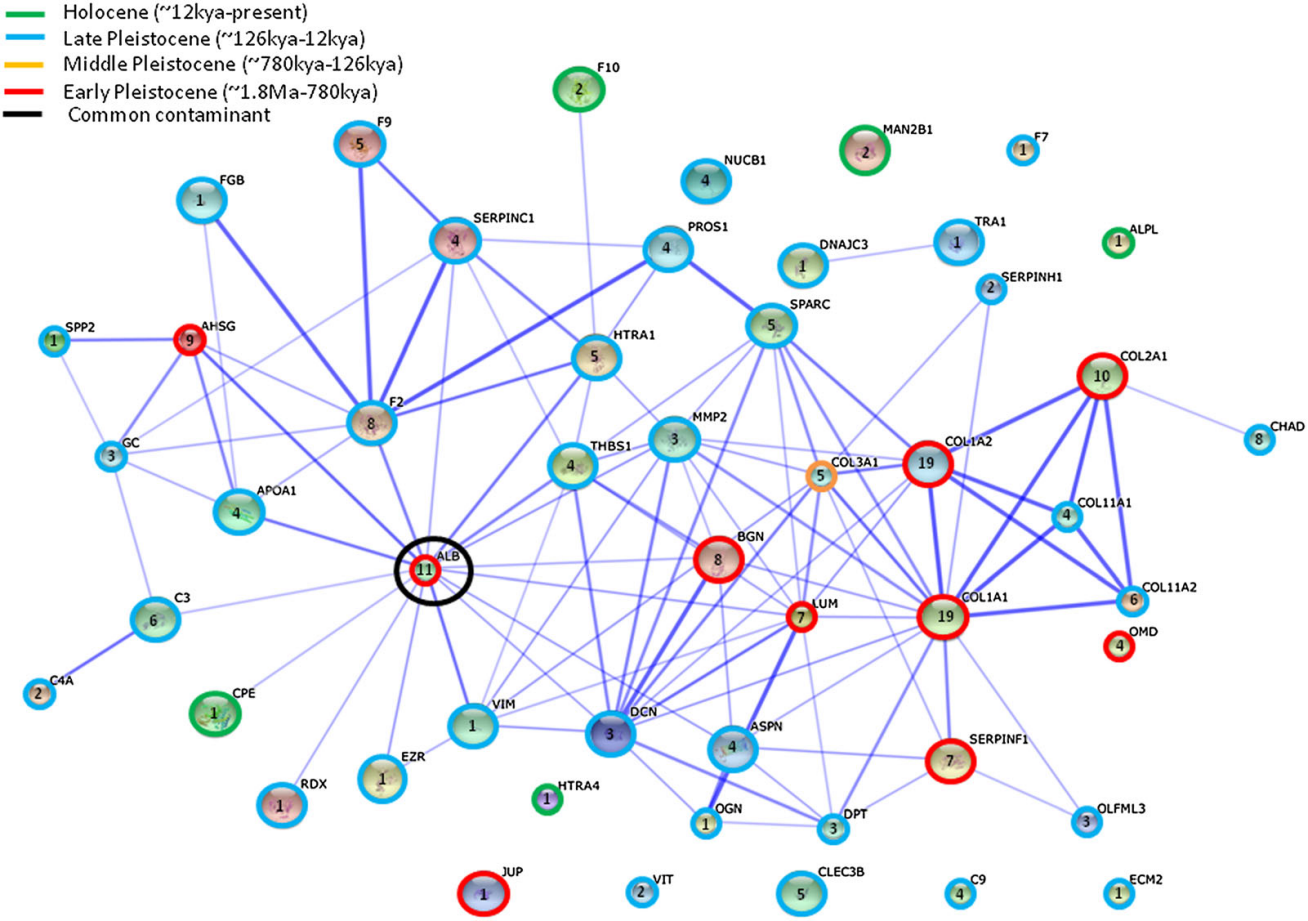
STRING network of bovine proteins. Blue lines between nodes represent functional associations between proteins with their thickness signifying the level of confidence; the colour of the ring surrounding each node indicates the minimum survival age; the number within the node indicates the number of specimens the protein was matched in, as a measure of confidence. Protein codes are listed in Supplementary Table S3 (see Supporting Information); STRING network without 100 mg HSB3 sample is presented in Supplementary Fig. S1 (see Supporting Information).

Proteins belonging to the small leucine-rich repeat (LRR) family, in particular biglycan, chondroadherin and lumican, also appear to survive well in archaeological bone. These LRR proteoglycans are known to interact with collagen to regulate fibril organisation,^38^,^39^ and additionally biglycan is known to have a role in bone growth and differentiation^40^; it may be this interaction with collagen that mediates their survival (Fig. 3).

### The effects of site history

Comparison of the proteomes identified in this study also indicates that the burial conditions of a sample have a significant effect on proteome complexity. Sample NS2 appears to have an unusually rich proteome containing more than double the number of unique identified proteins as the youngest sample analysed (UI3) and half the number of proteins identified in the modern bone standard (32 proteins in total). This specimen was recovered from the sea bed in the North Sea, and it is possible that the unusually good biomolecule preservation observed in this sample could be a result of this. Anoxic conditions underwater may have slowed the action of saprophytic microorganisms and the constant low temperatures may have delayed other forms of biomolecule decay such as chemical hydrolysis.

Conversely, samples AY2 and AY5 are relatively young (approximately 10,000 years old) but have very poor proteomes, containing no identifiable NCPs in AY2 and 8 NCPs in AY5. They are both from an archaeological assemblage at Ais Yiorkis, Cyprus, a warm climate which could potentially have accelerated biomolecule decay. These data concur with current knowledge of the effect of burial environment on biomolecule survival in fossil bones.^25^,^41^,^42^

### Sample contamination

Although proteomics techniques are much less sensitive to contamination than those used to study ancient DNA, there are still several sources of contamination which must be considered. The handling of fossils by researchers prior to proteomic analysis can introduce modern contaminant proteins such as keratins from skin and hair, and the use of latex gloves can introduce contaminants via leaching into the sample.^43^ In addition, bovine serum albumin (BSA) is commonly used in the laboratory and therefore a possible source of contamination.

To overcome these contamination issues any keratin peptides (including bovine) identified in Mascot searches were discounted as potential contaminants despite the use of nitrile gloves when handling and preparing samples. The problem of contaminant BSA is more difficult to resolve especially as this study focuses on bovine remains; the careful examination of any albumin peptides identified in an ancient sample for diagenetic alterations (e.g., deamidation) could potentially be used to support their authenticity, but in this study we found Mascot matches in most samples including the modern reference material, yet not in the oldest HSB3 sample. However, measuring the extent of deamidation within LC/MS data is more complex than from peptide mass fingerprinting data (e.g.^44^) due to the need to account for the separation of unmodified and modified forms during liquid chromatography and the likelihood of selection in mass spectrometric analysis. Using sequence differences to authenticate the endogeneity of ancient proteins would be ideal (e.g.^21^), but remains a limitation of this study, perhaps overcome in future proteome degradation studies of ancient remains from other species.

### Sequence variation in alpha-2-HS-glycoprotein

Albumin has long been considered a particularly useful protein for phylogenetic studies of extinct species because of its high amino acid variability. For example, brown rat (*Rattus norvegicus*) albumin varies from house mouse (*Mus musculus*) albumin at 62 positions (sequence similarity 89.8%; Supplementary Text S1, Supporting Information). By comparison, brown rat A2HSG differs from mouse A2HSG at 52 positions (sequence similarity 81.5%, although mouse has 7 insertions at 3 different positions; Supplementary Text S1). In collagen, brown rat alpha 1(I) differs from mouse in 16 positions; and the alpha 2(I) in 40 positions. However, it should also be noted that in this case the albumin is almost twice as long (608 amino acids) as the A2HSG (345 residues), making the latter relatively more variable (likewise each collagen chain is ∼3 times longer). A similar trend is observed with the ruminants, e.g., sheep (*Ovis aries*) has 30 amino acid substitutions from cow (*Bos taurus*; total 359 residues; sequence similarity 89.6%; cow has 5 amino acid insertions at one position), whereas this is 46 amino acid differences for albumin between the same species (of 607 residues; sequence similarity 92.4%). In addition, A2HSG may prove to be more useful for phylogenetic studies using ancient samples as it is a significantly smaller molecule than albumin, increasing the likelihood of obtaining more comparable sequence coverages from different samples resulting in improved molecular phylogenies. A2HSG has the added advantage of being uncommon in laboratories and thus is unlikely to be a modern contaminant.

We propose that A2HSG may be the most useful of the NCPs detected in this study for phylogenetic comparisons and species identification of fragmentary bone as it is a smaller protein than albumin, therefore increasing the chances that the same peptides can be reliably extracted from different samples for comparison (Table 3); it is also more variable (number of amino acid substitutions per site) than albumin, and is not a common laboratory contaminant.

**Table 3:** Comparison of the two A2HSG peptide sequences that were found in all archaeological and palaeontological bovine specimens to 22 other species (dot indicates conserved amino acid)

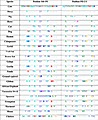

## CONCLUSIONS

The results of this study agree with much of the previously published work on ancient proteins in that collagen, in particular the collagen alpha-2 (I) and alpha-1 (I) chains, is the longest surviving protein in ancient bone. However, this study has also highlighted the fact that there are several other NCPs which can be commonly identified in archaeological bone samples, in particular serum albumin, A2HSG, prothrombin and biglycan. In addition we have shown that NCPs can be extracted from samples up to ∼900,000 years old, although as sample age increases the number of NCPs identified and the number of unique peptide matches for each protein decreases. Given the success in the Early Pleistocene/Lower Palaeolithic sample from Happisburgh, an archaeological site representative of the earliest human occupation of Britain,^45^ the potential for greater taxonomic resolution in NCP sequence information could yield a better source of information for past human-animal interactions than can be obtained using the current collagen-based methods.^46^

Although albumin has been targeted before as a potential source of phylogenetic information for ancient samples due to its known abundance and sequence variability,^30^ we propose that A2HSG is a more ideal target because it has a higher relative variability than serum albumin, and its smaller size may increase the likelihood that comparable peptides and sequence coverages can be obtained from different samples.
