# Supplementary material for: Proteome degradation in fossils: investigating the longevity of protein survival in ancient bone
Source: Rapid Commun Mass Spectrom. 2014 Feb 12;28(6):605–15. doi: 10.1002/rcm.6821 (PMC4282581; doi:10.1002/rcm.6821)
Supplement: Supplementary file 1 [file rcm0028-0605-SD1.docx]

**Table S1 – Summary Tables of Protein Matches**

**Collagenase Batch 1**

**KC2**

| **prot_desc** | **prot_score** |
| --- | --- |
| Collagen alpha-2(I) chain OS=Bos taurus GN=COL1A2 PE=1 SV=2 | 7090 |

| **pep_exp_mz** | **pep_exp_mr** | **pep_calc_mr** | **pep_score** | **pep_seq** | **pep_var_mod** |
| --- | --- | --- | --- | --- | --- |
| 434.7354 | 867.4562 | 867.4563 | 48.45 | GPSGPQGIR |  |
| 434.7354 | 867.4562 | 867.4563 | 48.98 | GPSGPQGIR |  |
| 435.2274 | 868.4402 | 868.4403 | 40.9 | GPSGPQGIR | Deamidated (NQ) |
| 446.7536 | 891.4926 | 891.4926 | 43.71 | PGPIGPAGAR |  |
| 446.7536 | 891.4926 | 891.4926 | 40.37 | PGPIGPAGAR |  |
| 454.751 | 907.4874 | 907.4876 | 42.44 | PGPIGPAGAR | Oxidation (P) |
| 529.7503 | 1057.486 | 1057.486 | 42.18 | PGEPGLMGPR | Oxidation (M); 2 Oxidation (P) |
| 529.7504 | 1057.486 | 1057.486 | 46.31 | PGEPGLMGPR | Oxidation (M); 2 Oxidation (P) |
| 542.7851 | 1083.556 | 1083.556 | 51.29 | GLVGEPGPAGSK | Oxidation (P) |
| 542.7854 | 1083.556 | 1083.556 | 54.41 | GLVGEPGPAGSK | Oxidation (P) |
| 542.7855 | 1083.556 | 1083.556 | 46.71 | GLVGEPGPAGSK | Oxidation (P) |
| 550.7829 | 1099.551 | 1099.551 | 45.21 | GLVGEPGPAGSK | Oxidation (K); Oxidation (P) |
| 591.8093 | 1181.604 | 1181.604 | 49.28 | EGPVGLPGIDGR | Oxidation (P) |
| 591.8093 | 1181.604 | 1181.604 | 61.43 | EGPVGLPGIDGR | Oxidation (P) |
| 591.8096 | 1181.605 | 1181.604 | 46.14 | EGPVGLPGIDGR | Oxidation (P) |
| 591.81 | 1181.605 | 1181.604 | 61.58 | EGPVGLPGIDGR | Oxidation (P) |
| 596.8433 | 1191.672 | 1191.672 | 62.88 | IGQPGAVGPAGIR |  |
| 596.8434 | 1191.672 | 1191.672 | 50.62 | IGQPGAVGPAGIR |  |
| 597.3353 | 1192.656 | 1192.656 | 64.23 | IGQPGAVGPAGIR | Deamidated (NQ) |
| 597.3353 | 1192.656 | 1192.656 | 64.57 | IGQPGAVGPAGIR | Deamidated (NQ) |
| 597.3353 | 1192.656 | 1192.656 | 72.69 | IGQPGAVGPAGIR | Deamidated (NQ) |
| 597.3353 | 1192.656 | 1192.656 | 59.27 | IGQPGAVGPAGIR | Deamidated (NQ) |
| 601.2957 | 1200.577 | 1200.578 | 56.88 | GEPGNIGFPGPK | 2 Oxidation (P) |
| 601.2957 | 1200.577 | 1200.578 | 57.29 | GEPGNIGFPGPK | 2 Oxidation (P) |
| 601.2966 | 1200.579 | 1200.578 | 41.13 | GEPGNIGFPGPK | 2 Oxidation (P) |
| 601.7878 | 1201.561 | 1201.562 | 44.25 | GEPGNIGFPGPK | Deamidated (NQ); Oxidation (K); Oxidation (P) |
| 601.7879 | 1201.561 | 1201.562 | 69.47 | GEPGNIGFPGPK | Deamidated (NQ); 2 Oxidation (P) |
| 403.5628 | 1207.667 | 1207.667 | 45.48 | IGQPGAVGPAGIR | Oxidation (P) |
| 604.8406 | 1207.667 | 1207.667 | 49.83 | IGQPGAVGPAGIR | Oxidation (P) |
| 604.8407 | 1207.667 | 1207.667 | 56.43 | IGQPGAVGPAGIR | Oxidation (P) |
| 604.841 | 1207.667 | 1207.667 | 54.6 | IGQPGAVGPAGIR | Oxidation (P) |
| 604.841 | 1207.667 | 1207.667 | 63.97 | IGQPGAVGPAGIR | Oxidation (P) |
| 605.3324 | 1208.65 | 1208.651 | 56.57 | IGQPGAVGPAGIR | Deamidated (NQ); Oxidation (P) |
| 605.3325 | 1208.65 | 1208.651 | 43.89 | IGQPGAVGPAGIR | Deamidated (NQ); Oxidation (P) |
| 605.3325 | 1208.65 | 1208.651 | 55.04 | IGQPGAVGPAGIR | Deamidated (NQ); Oxidation (P) |
| 403.8909 | 1208.651 | 1208.651 | 44.45 | IGQPGAVGPAGIR | Deamidated (NQ); Oxidation (P) |
| 605.3328 | 1208.651 | 1208.651 | 56.67 | IGQPGAVGPAGIR | Deamidated (NQ); Oxidation (P) |
| 605.3328 | 1208.651 | 1208.651 | 51.28 | IGQPGAVGPAGIR | Deamidated (NQ); Oxidation (P) |
| 605.3329 | 1208.651 | 1208.651 | 40.56 | IGQPGAVGPAGIR | Deamidated (NQ); Oxidation (P) |
| 611.809 | 1221.603 | 1221.603 | 40.08 | GFPGTPGLPGFK | 3 Oxidation (P) |
| 631.3179 | 1260.621 | 1260.621 | 73.14 | GEAGPAGPAGPAGPR |  |
| 631.3179 | 1260.621 | 1260.621 | 66.9 | GEAGPAGPAGPAGPR |  |
| 634.341 | 1266.667 | 1266.668 | 66.11 | GIPGPVGAAGATGAR | Oxidation (P) |
| 634.3411 | 1266.668 | 1266.668 | 41.33 | GIPGPVGAAGATGAR | Oxidation (P) |
| 634.3412 | 1266.668 | 1266.668 | 43.92 | GIPGPVGAAGATGAR | Oxidation (P) |
| 634.3415 | 1266.668 | 1266.668 | 73.89 | GIPGPVGAAGATGAR | Oxidation (P) |
| 634.3415 | 1266.668 | 1266.668 | 65.81 | GIPGPVGAAGATGAR | Oxidation (P) |
| 634.3416 | 1266.669 | 1266.668 | 75.5 | GIPGPVGAAGATGAR | Oxidation (P) |
| 634.3417 | 1266.669 | 1266.668 | 52.48 | GIPGPVGAAGATGAR | Oxidation (P) |
| 644.8114 | 1287.608 | 1287.61 | 67.71 | GFPGSPGNIGPAGK | Deamidated (NQ); 2 Oxidation (P) |
| 644.8115 | 1287.608 | 1287.61 | 40.63 | GFPGSPGNIGPAGK | Deamidated (NQ); 2 Oxidation (P) |
| 644.8116 | 1287.609 | 1287.61 | 79.24 | GFPGSPGNIGPAGK | Deamidated (NQ); 2 Oxidation (P) |
| 644.8116 | 1287.609 | 1287.61 | 50.74 | GFPGSPGNIGPAGK | Deamidated (NQ); 2 Oxidation (P) |
| 714.3672 | 1426.72 | 1426.721 | 66.21 | GIPGEFGLPGPAGAR | 2 Oxidation (P) |
| 714.3674 | 1426.72 | 1426.721 | 68.48 | GIPGEFGLPGPAGAR | 2 Oxidation (P) |
| 714.3676 | 1426.721 | 1426.721 | 58.48 | GIPGEFGLPGPAGAR | 2 Oxidation (P) |
| 714.3676 | 1426.721 | 1426.721 | 54.71 | GIPGEFGLPGPAGAR | 2 Oxidation (P) |
| 714.3677 | 1426.721 | 1426.721 | 50.26 | GIPGEFGLPGPAGAR | 2 Oxidation (P) |
| 714.3677 | 1426.721 | 1426.721 | 55.19 | GIPGEFGLPGPAGAR | 2 Oxidation (P) |
| 714.3677 | 1426.721 | 1426.721 | 40.12 | GIPGEFGLPGPAGAR | 2 Oxidation (P) |
| 714.3679 | 1426.721 | 1426.721 | 66.87 | GIPGEFGLPGPAGAR | 2 Oxidation (P) |
| 714.368 | 1426.721 | 1426.721 | 60.7 | GIPGEFGLPGPAGAR | 2 Oxidation (P) |
| 714.368 | 1426.721 | 1426.721 | 57.18 | GIPGEFGLPGPAGAR | 2 Oxidation (P) |
| 714.3681 | 1426.722 | 1426.721 | 42.86 | GIPGEFGLPGPAGAR | 2 Oxidation (P) |
| 714.3683 | 1426.722 | 1426.721 | 48.57 | GIPGEFGLPGPAGAR | 2 Oxidation (P) |
| 714.3685 | 1426.722 | 1426.721 | 45.5 | GIPGEFGLPGPAGAR | 2 Oxidation (P) |
| 722.366 | 1442.717 | 1442.715 | 55.4 | GIPGEFGLPGPAGAR | 3 Oxidation (P) |
| 722.366 | 1442.717 | 1442.715 | 47.44 | GIPGEFGLPGPAGAR | 3 Oxidation (P) |
| 729.3422 | 1456.67 | 1456.67 | 64.84 | GDGGPPGATGFPGAAGR | Oxidation (P) |
| 729.3426 | 1456.671 | 1456.67 | 75.35 | GDGGPPGATGFPGAAGR | Oxidation (P) |
| 737.3384 | 1472.662 | 1472.665 | 47.88 | GDGGPPGATGFPGAAGR | 2 Oxidation (P) |
| 737.3387 | 1472.663 | 1472.665 | 76.59 | GDGGPPGATGFPGAAGR | 2 Oxidation (P) |
| 737.3387 | 1472.663 | 1472.665 | 52.73 | GDGGPPGATGFPGAAGR | 2 Oxidation (P) |
| 737.3389 | 1472.663 | 1472.665 | 57.44 | GDGGPPGATGFPGAAGR | 2 Oxidation (P) |
| 746.8492 | 1491.684 | 1491.684 | 78.89 | SGETGASGPPGFVGEK | Oxidation (P) |
| 746.8496 | 1491.685 | 1491.684 | 76.61 | SGETGASGPPGFVGEK | Oxidation (P) |
| 511.5986 | 1531.774 | 1531.774 | 59.46 | GEPGPAGAVGPAGAVGPR | Oxidation (P) |
| 511.5986 | 1531.774 | 1531.774 | 59.3 | GEPGPAGAVGPAGAVGPR | Oxidation (P) |
| 766.8945 | 1531.774 | 1531.774 | 90.28 | GEPGPAGAVGPAGAVGPR | Oxidation (P) |
| 766.8945 | 1531.774 | 1531.774 | 59.66 | GEPGPAGAVGPAGAVGPR | Oxidation (P) |
| 766.8946 | 1531.775 | 1531.774 | 68.69 | GEPGPAGAVGPAGAVGPR | Oxidation (P) |
| 766.8947 | 1531.775 | 1531.774 | 89.64 | GEPGPAGAVGPAGAVGPR | Oxidation (P) |
| 766.8947 | 1531.775 | 1531.774 | 48.71 | GEPGPAGAVGPAGAVGPR | Oxidation (P) |
| 766.8947 | 1531.775 | 1531.774 | 53.83 | GEPGPAGAVGPAGAVGPR | Oxidation (P) |
| 766.8948 | 1531.775 | 1531.774 | 43.86 | GEPGPAGAVGPAGAVGPR | Oxidation (P) |
| 769.3717 | 1536.729 | 1536.728 | 53.69 | GAPGAIGAPGPAGANGDR | 2 Oxidation (P) |
| 769.864 | 1537.713 | 1537.712 | 52.04 | GAPGAIGAPGPAGANGDR | Deamidated (NQ); 2 Oxidation (P) |
| 769.864 | 1537.713 | 1537.712 | 56.89 | GAPGAIGAPGPAGANGDR | Deamidated (NQ); 2 Oxidation (P) |
| 781.9174 | 1561.82 | 1561.821 | 42.66 | GAAGLPGVAGAPGLPGPR | 3 Oxidation (P) |
| 781.9175 | 1561.82 | 1561.821 | 47.51 | GAAGLPGVAGAPGLPGPR | 3 Oxidation (P) |
| 781.9176 | 1561.821 | 1561.821 | 69.48 | GAAGLPGVAGAPGLPGPR | 3 Oxidation (P) |
| 781.9183 | 1561.822 | 1561.821 | 44.44 | GAAGLPGVAGAPGLPGPR | 3 Oxidation (P) |
| 527.5936 | 1579.759 | 1579.759 | 61.49 | GPPGESGAAGPTGPIGSR | Oxidation (P) |
| 527.5938 | 1579.76 | 1579.759 | 55.33 | GPPGESGAAGPTGPIGSR | Oxidation (P) |
| 790.8871 | 1579.76 | 1579.759 | 64.21 | GPPGESGAAGPTGPIGSR | Oxidation (P) |
| 790.8873 | 1579.76 | 1579.759 | 43.92 | GPPGESGAAGPTGPIGSR | Oxidation (P) |
| 790.8875 | 1579.76 | 1579.759 | 80.25 | GPPGESGAAGPTGPIGSR | Oxidation (P) |
| 790.8875 | 1579.76 | 1579.759 | 74.45 | GPPGESGAAGPTGPIGSR | Oxidation (P) |
| 790.8875 | 1579.76 | 1579.759 | 74.74 | GPPGESGAAGPTGPIGSR | Oxidation (P) |
| 800.4154 | 1598.816 | 1598.817 | 74.71 | GELGPVGNPGPAGPAGPR |  |
| 800.4156 | 1598.817 | 1598.817 | 67.5 | GELGPVGNPGPAGPAGPR |  |
| 800.9075 | 1599.8 | 1599.801 | 59.27 | GELGPVGNPGPAGPAGPR | Deamidated (NQ) |
| 800.908 | 1599.801 | 1599.801 | 58.63 | GELGPVGNPGPAGPAGPR | Deamidated (NQ) |
| 808.4128 | 1614.811 | 1614.811 | 62.92 | GELGPVGNPGPAGPAGPR | Oxidation (P) |
| 808.4133 | 1614.812 | 1614.811 | 54.93 | GELGPVGNPGPAGPAGPR | Oxidation (P) |
| 808.9046 | 1615.795 | 1615.795 | 65.42 | GELGPVGNPGPAGPAGPR | Deamidated (NQ); Oxidation (P) |
| 808.9052 | 1615.796 | 1615.795 | 66.45 | GELGPVGNPGPAGPAGPR | Deamidated (NQ); Oxidation (P) |
| 816.9025 | 1631.79 | 1631.79 | 52.72 | GELGPVGNPGPAGPAGPR | Deamidated (NQ); 2 Oxidation (P) |
| 824.9179 | 1647.821 | 1647.822 | 49.72 | GSTGEIGPAGPPGPPGLR | 2 Oxidation (P) |
| 824.918 | 1647.821 | 1647.822 | 48.48 | GSTGEIGPAGPPGPPGLR | 2 Oxidation (P) |
| 550.2811 | 1647.822 | 1647.822 | 45.87 | GSTGEIGPAGPPGPPGLR | 2 Oxidation (P) |
| 824.9181 | 1647.822 | 1647.822 | 46.42 | GSTGEIGPAGPPGPPGLR | 2 Oxidation (P) |
| 824.9183 | 1647.822 | 1647.822 | 50.12 | GSTGEIGPAGPPGPPGLR | 2 Oxidation (P) |
| 824.9183 | 1647.822 | 1647.822 | 48.1 | GSTGEIGPAGPPGPPGLR | 2 Oxidation (P) |
| 824.9183 | 1647.822 | 1647.822 | 57.26 | GSTGEIGPAGPPGPPGLR | 2 Oxidation (P) |
| 843.9427 | 1685.871 | 1685.87 | 57.69 | GAAGIPGGKGEKGETGLR | Oxidation (K); Oxidation (P) |
| 602.3149 | 1803.923 | 1803.923 | 49.58 | RGSTGEIGPAGPPGPPGLR | 2 Oxidation (P) |
| 912.4113 | 1822.808 | 1822.808 | 72.73 | GPPGNVGNPGVNGAPGEAGR | 2 Deamidated (NQ); 3 Oxidation (P) |
| 912.4121 | 1822.81 | 1822.808 | 63.23 | GPPGNVGNPGVNGAPGEAGR | 2 Deamidated (NQ); 3 Oxidation (P) |
| 912.9035 | 1823.792 | 1823.792 | 80.74 | GPPGNVGNPGVNGAPGEAGR | 3 Deamidated (NQ); 3 Oxidation (P) |
| 912.904 | 1823.793 | 1823.792 | 87.85 | GPPGNVGNPGVNGAPGEAGR | 3 Deamidated (NQ); 3 Oxidation (P) |
| 915.4553 | 1828.896 | 1828.896 | 44.97 | TGPPGPSGISGPPGPPGPAGK | Oxidation (K); 2 Oxidation (P) |
| 915.4555 | 1828.896 | 1828.896 | 43.78 | TGPPGPSGISGPPGPPGPAGK | Oxidation (K); 2 Oxidation (P) |
| 923.4532 | 1844.892 | 1844.891 | 40.43 | TGPPGPSGISGPPGPPGPAGK | 4 Oxidation (P) |
| 666.6685 | 1996.984 | 1996.983 | 49.04 | HGNRGEPGPAGAVGPAGAVGPR | Deamidated (NQ); Oxidation (P) |
| 666.6685 | 1996.984 | 1996.983 | 42.1 | HGNRGEPGPAGAVGPAGAVGPR | Deamidated (NQ); Oxidation (P) |
| 999.4994 | 1996.984 | 1996.983 | 51.62 | HGNRGEPGPAGAVGPAGAVGPR | Deamidated (NQ); Oxidation (P) |
| 1026 | 2049.985 | 2049.987 | 73.12 | GEVGPAGPNGFAGPAGAAGQPGAK | Oxidation (P) |
| 1026.001 | 2049.987 | 2049.987 | 74.1 | GEVGPAGPNGFAGPAGAAGQPGAK | Oxidation (P) |
| 1026.492 | 2050.969 | 2050.971 | 74.64 | GEVGPAGPNGFAGPAGAAGQPGAK | Deamidated (NQ); Oxidation (K) |
| 1026.492 | 2050.969 | 2050.971 | 90.31 | GEVGPAGPNGFAGPAGAAGQPGAK | Deamidated (NQ); Oxidation (P) |
| 684.664 | 2050.97 | 2050.971 | 45.49 | GEVGPAGPNGFAGPAGAAGQPGAK | Deamidated (NQ); Oxidation (K) |
| 1026.493 | 2050.971 | 2050.971 | 78.23 | GEVGPAGPNGFAGPAGAAGQPGAK | Deamidated (NQ); Oxidation (P) |
| 1026.493 | 2050.971 | 2050.971 | 55.59 | GEVGPAGPNGFAGPAGAAGQPGAK | Deamidated (NQ); Oxidation (P) |
| 1026.986 | 2051.957 | 2051.955 | 86.19 | GEVGPAGPNGFAGPAGAAGQPGAK | 2 Deamidated (NQ); Oxidation (P) |
| 1026.986 | 2051.957 | 2051.955 | 81.31 | GEVGPAGPNGFAGPAGAAGQPGAK | 2 Deamidated (NQ); Oxidation (P) |
| 705.7116 | 2114.113 | 2114.112 | 41.51 | GLPGVAGSVGEPGPLGIAGPPGAR | 2 Oxidation (P) |
| 1058.064 | 2114.113 | 2114.112 | 44.87 | GLPGVAGSVGEPGPLGIAGPPGAR | 2 Oxidation (P) |
| 705.7123 | 2114.115 | 2114.112 | 50.43 | GLPGVAGSVGEPGPLGIAGPPGAR | 2 Oxidation (P) |
| 1058.065 | 2114.115 | 2114.112 | 85.74 | GLPGVAGSVGEPGPLGIAGPPGAR | 2 Oxidation (P) |
| 1058.065 | 2114.115 | 2114.112 | 75.09 | GLPGVAGSVGEPGPLGIAGPPGAR | 2 Oxidation (P) |
| 1058.065 | 2114.115 | 2114.112 | 72.71 | GLPGVAGSVGEPGPLGIAGPPGAR | 2 Oxidation (P) |
| 1066.059 | 2130.103 | 2130.107 | 70.28 | GLPGVAGSVGEPGPLGIAGPPGAR | 3 Oxidation (P) |
| 711.0421 | 2130.105 | 2130.107 | 42.29 | GLPGVAGSVGEPGPLGIAGPPGAR | 3 Oxidation (P) |
| 711.0423 | 2130.105 | 2130.107 | 41.65 | GLPGVAGSVGEPGPLGIAGPPGAR | 3 Oxidation (P) |
| 1066.06 | 2130.105 | 2130.107 | 122.07 | GLPGVAGSVGEPGPLGIAGPPGAR | 3 Oxidation (P) |
| 1066.06 | 2130.105 | 2130.107 | 46.78 | GLPGVAGSVGEPGPLGIAGPPGAR | 3 Oxidation (P) |
| 1066.06 | 2130.105 | 2130.107 | 51.2 | GLPGVAGSVGEPGPLGIAGPPGAR | 3 Oxidation (P) |
| 1066.06 | 2130.105 | 2130.107 | 58.38 | GLPGVAGSVGEPGPLGIAGPPGAR | 3 Oxidation (P) |
| 1066.06 | 2130.105 | 2130.107 | 81.92 | GLPGVAGSVGEPGPLGIAGPPGAR | 3 Oxidation (P) |
| 1066.06 | 2130.105 | 2130.107 | 72.55 | GLPGVAGSVGEPGPLGIAGPPGAR | 3 Oxidation (P) |
| 1066.06 | 2130.105 | 2130.107 | 85.64 | GLPGVAGSVGEPGPLGIAGPPGAR | 3 Oxidation (P) |
| 711.0428 | 2130.107 | 2130.107 | 42.93 | GLPGVAGSVGEPGPLGIAGPPGAR | 3 Oxidation (P) |
| 711.043 | 2130.107 | 2130.107 | 68.56 | GLPGVAGSVGEPGPLGIAGPPGAR | 3 Oxidation (P) |
| 1066.061 | 2130.107 | 2130.107 | 95.37 | GLPGVAGSVGEPGPLGIAGPPGAR | 3 Oxidation (P) |
| 1066.061 | 2130.107 | 2130.107 | 107.11 | GLPGVAGSVGEPGPLGIAGPPGAR | 3 Oxidation (P) |
| 1066.061 | 2130.107 | 2130.107 | 101.41 | GLPGVAGSVGEPGPLGIAGPPGAR | 3 Oxidation (P) |
| 1066.061 | 2130.107 | 2130.107 | 103.85 | GLPGVAGSVGEPGPLGIAGPPGAR | 3 Oxidation (P) |
| 1066.061 | 2130.107 | 2130.107 | 64.79 | GLPGVAGSVGEPGPLGIAGPPGAR | 3 Oxidation (P) |
| 1066.061 | 2130.107 | 2130.107 | 71.34 | GLPGVAGSVGEPGPLGIAGPPGAR | 3 Oxidation (P) |
| 1066.061 | 2130.107 | 2130.107 | 50.46 | GLPGVAGSVGEPGPLGIAGPPGAR | 3 Oxidation (P) |
| 1066.061 | 2130.107 | 2130.107 | 44.35 | GLPGVAGSVGEPGPLGIAGPPGAR | 3 Oxidation (P) |
| 1066.061 | 2130.107 | 2130.107 | 102.32 | GLPGVAGSVGEPGPLGIAGPPGAR | 3 Oxidation (P) |
| 1066.061 | 2130.107 | 2130.107 | 47.72 | GLPGVAGSVGEPGPLGIAGPPGAR | 3 Oxidation (P) |
| 1066.061 | 2130.107 | 2130.107 | 49.78 | GLPGVAGSVGEPGPLGIAGPPGAR | 3 Oxidation (P) |
| 1066.061 | 2130.107 | 2130.107 | 79.04 | GLPGVAGSVGEPGPLGIAGPPGAR | 3 Oxidation (P) |
| 1066.061 | 2130.107 | 2130.107 | 84.29 | GLPGVAGSVGEPGPLGIAGPPGAR | 3 Oxidation (P) |
| 1066.061 | 2130.107 | 2130.107 | 87.93 | GLPGVAGSVGEPGPLGIAGPPGAR | 3 Oxidation (P) |
| 1066.061 | 2130.107 | 2130.107 | 70.06 | GLPGVAGSVGEPGPLGIAGPPGAR | 3 Oxidation (P) |
| 1066.061 | 2130.107 | 2130.107 | 52.33 | GLPGVAGSVGEPGPLGIAGPPGAR | 3 Oxidation (P) |
| 1066.061 | 2130.107 | 2130.107 | 55.51 | GLPGVAGSVGEPGPLGIAGPPGAR | 3 Oxidation (P) |
| 1066.061 | 2130.107 | 2130.107 | 58.53 | GLPGVAGSVGEPGPLGIAGPPGAR | 3 Oxidation (P) |
| 1066.061 | 2130.107 | 2130.107 | 69.21 | GLPGVAGSVGEPGPLGIAGPPGAR | 3 Oxidation (P) |
| 1066.061 | 2130.107 | 2130.107 | 47.21 | GLPGVAGSVGEPGPLGIAGPPGAR | 3 Oxidation (P) |
| 711.0432 | 2130.108 | 2130.107 | 51.59 | GLPGVAGSVGEPGPLGIAGPPGAR | 3 Oxidation (P) |
| 711.0434 | 2130.108 | 2130.107 | 64.27 | GLPGVAGSVGEPGPLGIAGPPGAR | 3 Oxidation (P) |
| 1066.062 | 2130.109 | 2130.107 | 58.54 | GLPGVAGSVGEPGPLGIAGPPGAR | 3 Oxidation (P) |
| 1066.062 | 2130.109 | 2130.107 | 92.24 | GLPGVAGSVGEPGPLGIAGPPGAR | 3 Oxidation (P) |
| 1066.062 | 2130.109 | 2130.107 | 110.16 | GLPGVAGSVGEPGPLGIAGPPGAR | 3 Oxidation (P) |
| 1066.062 | 2130.109 | 2130.107 | 73.05 | GLPGVAGSVGEPGPLGIAGPPGAR | 3 Oxidation (P) |
| 1066.062 | 2130.109 | 2130.107 | 74.42 | GLPGVAGSVGEPGPLGIAGPPGAR | 3 Oxidation (P) |
| 1066.062 | 2130.109 | 2130.107 | 75.57 | GLPGVAGSVGEPGPLGIAGPPGAR | 3 Oxidation (P) |
| 1066.062 | 2130.109 | 2130.107 | 65.8 | GLPGVAGSVGEPGPLGIAGPPGAR | 3 Oxidation (P) |
| 1066.062 | 2130.109 | 2130.107 | 61.63 | GLPGVAGSVGEPGPLGIAGPPGAR | 3 Oxidation (P) |
| 1066.062 | 2130.109 | 2130.107 | 82.51 | GLPGVAGSVGEPGPLGIAGPPGAR | 3 Oxidation (P) |
| 1066.062 | 2130.109 | 2130.107 | 58.59 | GLPGVAGSVGEPGPLGIAGPPGAR | 3 Oxidation (P) |
| 1066.062 | 2130.109 | 2130.107 | 63.74 | GLPGVAGSVGEPGPLGIAGPPGAR | 3 Oxidation (P) |
| 1066.062 | 2130.109 | 2130.107 | 69.81 | GLPGVAGSVGEPGPLGIAGPPGAR | 3 Oxidation (P) |
| 1066.062 | 2130.109 | 2130.107 | 73.63 | GLPGVAGSVGEPGPLGIAGPPGAR | 3 Oxidation (P) |
| 1066.062 | 2130.109 | 2130.107 | 92.62 | GLPGVAGSVGEPGPLGIAGPPGAR | 3 Oxidation (P) |
| 1066.062 | 2130.109 | 2130.107 | 90.41 | GLPGVAGSVGEPGPLGIAGPPGAR | 3 Oxidation (P) |
| 1066.062 | 2130.109 | 2130.107 | 87.89 | GLPGVAGSVGEPGPLGIAGPPGAR | 3 Oxidation (P) |
| 1066.062 | 2130.109 | 2130.107 | 46.28 | GLPGVAGSVGEPGPLGIAGPPGAR | 3 Oxidation (P) |
| 1066.062 | 2130.109 | 2130.107 | 78.19 | GLPGVAGSVGEPGPLGIAGPPGAR | 3 Oxidation (P) |
| 1066.063 | 2130.111 | 2130.107 | 86.12 | GLPGVAGSVGEPGPLGIAGPPGAR | 3 Oxidation (P) |
| 1066.063 | 2130.111 | 2130.107 | 96.93 | GLPGVAGSVGEPGPLGIAGPPGAR | 3 Oxidation (P) |
| 1066.063 | 2130.111 | 2130.107 | 51.95 | GLPGVAGSVGEPGPLGIAGPPGAR | 3 Oxidation (P) |
| 1066.063 | 2130.111 | 2130.107 | 68.62 | GLPGVAGSVGEPGPLGIAGPPGAR | 3 Oxidation (P) |
| 1066.066 | 2130.117 | 2130.107 | 57.45 | GLPGVAGSVGEPGPLGIAGPPGAR | 3 Oxidation (P) |
| 1066.066 | 2130.117 | 2130.107 | 67.05 | GLPGVAGSVGEPGPLGIAGPPGAR | 3 Oxidation (P) |
| 1074.058 | 2146.101 | 2146.102 | 70.21 | GLPGVAGSVGEPGPLGIAGPPGAR | 4 Oxidation (P) |
| 1074.058 | 2146.101 | 2146.102 | 60.12 | GLPGVAGSVGEPGPLGIAGPPGAR | 4 Oxidation (P) |
| 1074.058 | 2146.101 | 2146.102 | 56.75 | GLPGVAGSVGEPGPLGIAGPPGAR | 4 Oxidation (P) |
| 1074.058 | 2146.101 | 2146.102 | 58.82 | GLPGVAGSVGEPGPLGIAGPPGAR | 4 Oxidation (P) |
| 1074.059 | 2146.103 | 2146.102 | 43.56 | GLPGVAGSVGEPGPLGIAGPPGAR | 4 Oxidation (P) |
| 1074.059 | 2146.103 | 2146.102 | 58.43 | GLPGVAGSVGEPGPLGIAGPPGAR | 4 Oxidation (P) |
| 1074.059 | 2146.103 | 2146.102 | 49.1 | GLPGVAGSVGEPGPLGIAGPPGAR | 4 Oxidation (P) |
| 1074.059 | 2146.103 | 2146.102 | 52.09 | GLPGVAGSVGEPGPLGIAGPPGAR | 4 Oxidation (P) |
| 1074.06 | 2146.105 | 2146.102 | 44.73 | GLPGVAGSVGEPGPLGIAGPPGAR | 4 Oxidation (P) |
| 1082.057 | 2162.099 | 2162.097 | 44.66 | GLPGVAGSVGEPGPLGIAGPPGAR | 5 Oxidation (P) |
| 1206.064 | 2410.113 | 2410.115 | 83.34 | GEVGPAGPNGFAGPAGAAGQPGAKGER | 2 Deamidated (NQ); Oxidation (K); Oxidation (P) |
| 1206.065 | 2410.115 | 2410.115 | 72.92 | GEVGPAGPNGFAGPAGAAGQPGAKGER | 2 Deamidated (NQ); Oxidation (K); Oxidation (P) |
| 1215.616 | 2429.217 | 2429.219 | 57.69 | GEVGLPGLSGPVGPPGNPGANGLPGAK | 4 Oxidation (P) |
| 810.7469 | 2429.219 | 2429.219 | 43.02 | GEVGLPGLSGPVGPPGNPGANGLPGAK | 4 Oxidation (P) |
| 1215.619 | 2429.223 | 2429.219 | 68.71 | GEVGLPGLSGPVGPPGNPGANGLPGAK | 4 Oxidation (P) |
| 1216.108 | 2430.201 | 2430.203 | 60.19 | GEVGLPGLSGPVGPPGNPGANGLPGAK | Deamidated (NQ); 4 Oxidation (P) |
| 1216.108 | 2430.201 | 2430.203 | 48.89 | GEVGLPGLSGPVGPPGNPGANGLPGAK | Deamidated (NQ); 4 Oxidation (P) |
| 1216.601 | 2431.187 | 2431.187 | 67.28 | GEVGLPGLSGPVGPPGNPGANGLPGAK | 2 Deamidated (NQ); 4 Oxidation (P) |
| 1216.601 | 2431.187 | 2431.187 | 64 | GEVGLPGLSGPVGPPGNPGANGLPGAK | 2 Deamidated (NQ); 4 Oxidation (P) |
| 1223.615 | 2445.215 | 2445.214 | 65.9 | GEVGLPGLSGPVGPPGNPGANGLPGAK | Oxidation (K); 4 Oxidation (P) |
| 1223.615 | 2445.215 | 2445.214 | 64.37 | GEVGLPGLSGPVGPPGNPGANGLPGAK | Oxidation (K); 4 Oxidation (P) |
| 1223.619 | 2445.223 | 2445.214 | 66 | GEVGLPGLSGPVGPPGNPGANGLPGAK | Oxidation (K); 4 Oxidation (P) |
| 1224.105 | 2446.195 | 2446.198 | 46.37 | GEVGLPGLSGPVGPPGNPGANGLPGAK | Deamidated (NQ); Oxidation (K); 4 Oxidation (P) |
| 1224.106 | 2446.197 | 2446.198 | 50.05 | GEVGLPGLSGPVGPPGNPGANGLPGAK | Deamidated (NQ); Oxidation (K); 4 Oxidation (P) |
| 1224.598 | 2447.181 | 2447.182 | 58.98 | GEVGLPGLSGPVGPPGNPGANGLPGAK | 2 Deamidated (NQ); Oxidation (K); 4 Oxidation (P) |
| 858.4257 | 2572.255 | 2572.256 | 56.2 | GSDGSVGPVGPAGPIGSAGPPGFPGAPGPK | Oxidation (K); Oxidation (P) |
| 1287.136 | 2572.257 | 2572.256 | 68.64 | GSDGSVGPVGPAGPIGSAGPPGFPGAPGPK | Oxidation (K); Oxidation (P) |
| 1287.136 | 2572.257 | 2572.256 | 68.19 | GSDGSVGPVGPAGPIGSAGPPGFPGAPGPK | Oxidation (K); Oxidation (P) |
| 1287.137 | 2572.259 | 2572.256 | 80.81 | GSDGSVGPVGPAGPIGSAGPPGFPGAPGPK | Oxidation (K); Oxidation (P) |
| 861.0846 | 2580.232 | 2580.232 | 49.04 | GENGPVGPTGPVGAAGPSGPNGPPGPAGSR | Oxidation (P) |
| 1291.124 | 2580.233 | 2580.232 | 80.87 | GENGPVGPTGPVGAAGPSGPNGPPGPAGSR | Oxidation (P) |
| 1291.124 | 2580.233 | 2580.232 | 94.29 | GENGPVGPTGPVGAAGPSGPNGPPGPAGSR | Oxidation (P) |
| 1291.616 | 2581.217 | 2581.216 | 69.75 | GENGPVGPTGPVGAAGPSGPNGPPGPAGSR | Deamidated (NQ); Oxidation (P) |
| 1291.616 | 2581.217 | 2581.216 | 71.41 | GENGPVGPTGPVGAAGPSGPNGPPGPAGSR | Deamidated (NQ); Oxidation (P) |
| 861.4134 | 2581.218 | 2581.216 | 52.3 | GENGPVGPTGPVGAAGPSGPNGPPGPAGSR | Deamidated (NQ); Oxidation (P) |
| 861.7401 | 2582.199 | 2582.2 | 56.23 | GENGPVGPTGPVGAAGPSGPNGPPGPAGSR | 2 Deamidated (NQ); Oxidation (P) |
| 1292.108 | 2582.201 | 2582.2 | 78.93 | GENGPVGPTGPVGAAGPSGPNGPPGPAGSR | 2 Deamidated (NQ); Oxidation (P) |
| 1292.108 | 2582.201 | 2582.2 | 69.16 | GENGPVGPTGPVGAAGPSGPNGPPGPAGSR | 2 Deamidated (NQ); Oxidation (P) |
| 863.7579 | 2588.252 | 2588.251 | 42.53 | GSDGSVGPVGPAGPIGSAGPPGFPGAPGPK | 3 Oxidation (P) |
| 863.7579 | 2588.252 | 2588.251 | 40.32 | GSDGSVGPVGPAGPIGSAGPPGFPGAPGPK | 3 Oxidation (P) |
| 1295.134 | 2588.253 | 2588.251 | 87.49 | GSDGSVGPVGPAGPIGSAGPPGFPGAPGPK | Oxidation (K); 2 Oxidation (P) |
| 1295.134 | 2588.253 | 2588.251 | 89.8 | GSDGSVGPVGPAGPIGSAGPPGFPGAPGPK | Oxidation (K); 2 Oxidation (P) |
| 1295.136 | 2588.257 | 2588.251 | 41.93 | GSDGSVGPVGPAGPIGSAGPPGFPGAPGPK | Oxidation (K); 2 Oxidation (P) |
| 1295.139 | 2588.263 | 2588.251 | 50.2 | GSDGSVGPVGPAGPIGSAGPPGFPGAPGPK | Oxidation (K); 2 Oxidation (P) |
| 1303.13 | 2604.245 | 2604.246 | 46.28 | GSDGSVGPVGPAGPIGSAGPPGFPGAPGPK | Oxidation (K); 3 Oxidation (P) |
| 1303.132 | 2604.249 | 2604.246 | 47.1 | GSDGSVGPVGPAGPIGSAGPPGFPGAPGPK | Oxidation (K); 3 Oxidation (P) |
| 931.7733 | 2792.298 | 2792.3 | 44.92 | GEQGPAGPPGFQGLPGPAGTAGEAGKPGER | Deamidated (NQ); Oxidation (K); 2 Oxidation (P) |
| 955.4698 | 2863.388 | 2863.385 | 56.51 | GPKGENGPVGPTGPVGAAGPSGPNGPPGPAGSR | Deamidated (NQ); Oxidation (P) |
| 955.7974 | 2864.37 | 2864.369 | 56.69 | GPKGENGPVGPTGPVGAAGPSGPNGPPGPAGSR | 2 Deamidated (NQ); Oxidation (P) |
| 955.7985 | 2864.374 | 2864.369 | 57.62 | GPKGENGPVGPTGPVGAAGPSGPNGPPGPAGSR | 2 Deamidated (NQ); Oxidation (P) |
| 960.472 | 2878.394 | 2878.396 | 69.38 | GPKGENGPVGPTGPVGAAGPSGPNGPPGPAGSR | Oxidation (K); Oxidation (P) |
| 960.4726 | 2878.396 | 2878.396 | 46.15 | GPKGENGPVGPTGPVGAAGPSGPNGPPGPAGSR | Oxidation (K); Oxidation (P) |
| 960.8009 | 2879.381 | 2879.38 | 56.68 | GPKGENGPVGPTGPVGAAGPSGPNGPPGPAGSR | Deamidated (NQ); Oxidation (K); Oxidation (P) |
| 960.8015 | 2879.383 | 2879.38 | 56.97 | GPKGENGPVGPTGPVGAAGPSGPNGPPGPAGSR | Deamidated (NQ); Oxidation (K); Oxidation (P) |
| 961.1295 | 2880.367 | 2880.364 | 44.45 | GPKGENGPVGPTGPVGAAGPSGPNGPPGPAGSR | 2 Deamidated (NQ); Oxidation (K); Oxidation (P) |
| 961.1299 | 2880.368 | 2880.364 | 44.4 | GPKGENGPVGPTGPVGAAGPSGPNGPPGPAGSR | 2 Deamidated (NQ); Oxidation (K); Oxidation (P) |
| 1056.189 | 3165.545 | 3165.544 | 54.38 | GTKGPKGENGPVGPTGPVGAAGPSGPNGPPGPAGSR | Deamidated (NQ); Oxidation (K); Oxidation (P) |
| 1056.517 | 3166.529 | 3166.528 | 48.81 | GTKGPKGENGPVGPTGPVGAAGPSGPNGPPGPAGSR | 2 Deamidated (NQ); Oxidation (K); Oxidation (P) |
| 1056.517 | 3166.529 | 3166.528 | 54.61 | GTKGPKGENGPVGPTGPVGAAGPSGPNGPPGPAGSR | 2 Deamidated (NQ); Oxidation (K); Oxidation (P) |
| 1071.509 | 3211.505 | 3211.502 | 54.12 | GPSGPPGPDGNKGEPGVVGAPGTAGPSGPSGLPGER | Deamidated (NQ); Oxidation (K); 2 Oxidation (P) |

| Collagen alpha-1(I) chain OS=Bos taurus GN=COL1A1 PE=1 SV=3 | 5902 |
| --- | --- |

| 392.2217 | 782.4288 | 782.4286 | 42.82 | GAAGLPGPK | Oxidation (P) |
| --- | --- | --- | --- | --- | --- |
| 392.2218 | 782.429 | 782.4286 | 44.28 | GAAGLPGPK | Oxidation (P) |
| 434.2138 | 866.413 | 866.4134 | 42.13 | GFSGLDGAK | Oxidation (K) |
| 449.7587 | 897.5028 | 897.5032 | 47.87 | GVVGLPGQR | Oxidation (P) |
| 449.7588 | 897.503 | 897.5032 | 43.71 | GVVGLPGQR | Oxidation (P) |
| 449.7588 | 897.503 | 897.5032 | 54.56 | GVVGLPGQR | Oxidation (P) |
| 449.7589 | 897.5032 | 897.5032 | 43.78 | GVVGLPGQR | Oxidation (P) |
| 450.2505 | 898.4864 | 898.4872 | 41.17 | GVVGLPGQR | Deamidated (NQ); Oxidation (P) |
| 450.2505 | 898.4864 | 898.4872 | 45.86 | GVVGLPGQR | Deamidated (NQ); Oxidation (P) |
| 450.2508 | 898.487 | 898.4872 | 40.73 | GVVGLPGQR | Deamidated (NQ); Oxidation (P) |
| 544.772 | 1087.529 | 1087.53 | 66.07 | GFPGADGVAGPK | Oxidation (P) |
| 544.772 | 1087.529 | 1087.53 | 55.74 | GFPGADGVAGPK | Oxidation (P) |
| 544.7722 | 1087.53 | 1087.53 | 42 | GFPGADGVAGPK | Oxidation (P) |
| 553.2913 | 1104.568 | 1104.568 | 53.64 | GVQGPPGPAGPR | Oxidation (P) |
| 553.2913 | 1104.568 | 1104.568 | 53.65 | GVQGPPGPAGPR | Oxidation (P) |
| 553.7831 | 1105.552 | 1105.552 | 40.86 | GVQGPPGPAGPR | Deamidated (NQ); Oxidation (P) |
| 553.7833 | 1105.552 | 1105.552 | 45.57 | GVQGPPGPAGPR | Deamidated (NQ); Oxidation (P) |
| 553.7835 | 1105.552 | 1105.552 | 51.69 | GVQGPPGPAGPR | Deamidated (NQ); Oxidation (P) |
| 588.8223 | 1175.63 | 1175.63 | 47.98 | GVPGPPGAVGPAGK | Oxidation (P) |
| 589.2866 | 1176.559 | 1176.56 | 53.27 | GQAGVMGFPGPK | Oxidation (M); Oxidation (P) |
| 589.2866 | 1176.559 | 1176.56 | 61.19 | GQAGVMGFPGPK | Oxidation (M); Oxidation (P) |
| 589.2873 | 1176.56 | 1176.56 | 53.74 | GQAGVMGFPGPK | Oxidation (M); Oxidation (P) |
| 589.2874 | 1176.56 | 1176.56 | 51.25 | GQAGVMGFPGPK | Oxidation (M); Oxidation (P) |
| 589.2876 | 1176.561 | 1176.56 | 41.38 | GQAGVMGFPGPK | Oxidation (M); Oxidation (P) |
| 589.7792 | 1177.544 | 1177.544 | 68.93 | GQAGVMGFPGPK | Deamidated (NQ); Oxidation (M); Oxidation (P) |
| 589.7792 | 1177.544 | 1177.544 | 74.21 | GQAGVMGFPGPK | Deamidated (NQ); Oxidation (M); Oxidation (P) |
| 589.7793 | 1177.544 | 1177.544 | 77.12 | GQAGVMGFPGPK | Deamidated (NQ); Oxidation (M); Oxidation (P) |
| 589.7793 | 1177.544 | 1177.544 | 71.37 | GQAGVMGFPGPK | Deamidated (NQ); Oxidation (M); Oxidation (P) |
| 596.8193 | 1191.624 | 1191.625 | 47.93 | GVPGPPGAVGPAGK | 2 Oxidation (P) |
| 596.8195 | 1191.624 | 1191.625 | 42.04 | GVPGPPGAVGPAGK | 2 Oxidation (P) |
| 596.8198 | 1191.625 | 1191.625 | 46.36 | GVPGPPGAVGPAGK | 2 Oxidation (P) |
| 596.8198 | 1191.625 | 1191.625 | 46.23 | GVPGPPGAVGPAGK | 2 Oxidation (P) |
| 597.2847 | 1192.555 | 1192.555 | 43.02 | GQAGVMGFPGPK | Oxidation (K); Oxidation (M); Oxidation (P) |
| 597.2848 | 1192.555 | 1192.555 | 53.59 | GQAGVMGFPGPK | Oxidation (K); Oxidation (M); Oxidation (P) |
| 597.7761 | 1193.538 | 1193.539 | 47.92 | GQAGVMGFPGPK | Deamidated (NQ); Oxidation (K); Oxidation (M); Oxidation (P) |
| 597.7761 | 1193.538 | 1193.539 | 50.74 | GQAGVMGFPGPK | Deamidated (NQ); Oxidation (K); Oxidation (M); Oxidation (P) |
| 597.7764 | 1193.538 | 1193.539 | 58.5 | GQAGVMGFPGPK | Deamidated (NQ); Oxidation (K); Oxidation (M); Oxidation (P) |
| 597.7768 | 1193.539 | 1193.539 | 48.25 | GQAGVMGFPGPK | Deamidated (NQ); Oxidation (K); Oxidation (M); Oxidation (P) |
| 621.8019 | 1241.589 | 1241.589 | 42.9 | GLTGSPGSPGPDGK | Oxidation (P) |
| 621.8021 | 1241.59 | 1241.589 | 49.74 | GLTGSPGSPGPDGK | Oxidation (P) |
| 656.8304 | 1311.646 | 1311.646 | 44.23 | GFPGLPGPSGEPGK | Oxidation (P) |
| 656.8307 | 1311.647 | 1311.646 | 48.32 | GFPGLPGPSGEPGK | Oxidation (P) |
| 656.8309 | 1311.647 | 1311.646 | 40.66 | GFPGLPGPSGEPGK | Oxidation (P) |
| 664.8271 | 1327.64 | 1327.641 | 46.5 | GFPGLPGPSGEPGK | 2 Oxidation (P) |
| 664.8273 | 1327.64 | 1327.641 | 70.22 | GFPGLPGPSGEPGK | 2 Oxidation (P) |
| 664.8273 | 1327.64 | 1327.641 | 64.72 | GFPGLPGPSGEPGK | 2 Oxidation (P) |
| 664.8274 | 1327.64 | 1327.641 | 72.59 | GFPGLPGPSGEPGK | 2 Oxidation (P) |
| 664.8275 | 1327.64 | 1327.641 | 60.9 | GFPGLPGPSGEPGK | 2 Oxidation (P) |
| 664.8278 | 1327.641 | 1327.641 | 44.29 | GFPGLPGPSGEPGK | 2 Oxidation (P) |
| 664.8278 | 1327.641 | 1327.641 | 55.15 | GFPGLPGPSGEPGK | 2 Oxidation (P) |
| 664.8278 | 1327.641 | 1327.641 | 56 | GFPGLPGPSGEPGK | 2 Oxidation (P) |
| 664.8279 | 1327.641 | 1327.641 | 42.03 | GFPGLPGPSGEPGK | 2 Oxidation (P) |
| 664.8284 | 1327.642 | 1327.641 | 44.14 | GFPGLPGPSGEPGK | 2 Oxidation (P) |
| 667.3231 | 1332.632 | 1332.631 | 69.38 | GPSGPQGPSGPPGPK | Deamidated (NQ); Oxidation (P) |
| 672.8248 | 1343.635 | 1343.636 | 50.79 | GFPGLPGPSGEPGK | 3 Oxidation (P) |
| 672.8249 | 1343.635 | 1343.636 | 48.47 | GFPGLPGPSGEPGK | 3 Oxidation (P) |
| 672.8253 | 1343.636 | 1343.636 | 48.4 | GFPGLPGPSGEPGK | 3 Oxidation (P) |
| 672.8254 | 1343.636 | 1343.636 | 48 | GFPGLPGPSGEPGK | 3 Oxidation (P) |
| 672.8255 | 1343.636 | 1343.636 | 52.69 | GFPGLPGPSGEPGK | 3 Oxidation (P) |
| 718.3439 | 1434.673 | 1434.674 | 71.08 | GEPGPAGLPGPPGER | 3 Oxidation (P) |
| 718.3442 | 1434.674 | 1434.674 | 56.18 | GEPGPAGLPGPPGER | 3 Oxidation (P) |
| 718.3445 | 1434.674 | 1434.674 | 53.71 | GEPGPAGLPGPPGER | 3 Oxidation (P) |
| 718.3447 | 1434.675 | 1434.674 | 66.85 | GEPGPAGLPGPPGER | 3 Oxidation (P) |
| 718.3453 | 1434.676 | 1434.674 | 59.74 | GEPGPAGLPGPPGER | 3 Oxidation (P) |
| 718.3454 | 1434.676 | 1434.674 | 67.79 | GEPGPAGLPGPPGER | 3 Oxidation (P) |
| 730.3493 | 1458.684 | 1458.685 | 52.41 | GSAGPPGATGFPGAAGR | 2 Oxidation (P) |
| 730.3499 | 1458.685 | 1458.685 | 94.77 | GSAGPPGATGFPGAAGR | 2 Oxidation (P) |
| 730.3499 | 1458.685 | 1458.685 | 50.83 | GSAGPPGATGFPGAAGR | 2 Oxidation (P) |
| 730.3499 | 1458.685 | 1458.685 | 86.03 | GSAGPPGATGFPGAAGR | 2 Oxidation (P) |
| 730.35 | 1458.685 | 1458.685 | 105.7 | GSAGPPGATGFPGAAGR | 2 Oxidation (P) |
| 730.3501 | 1458.686 | 1458.685 | 42.59 | GSAGPPGATGFPGAAGR | 2 Oxidation (P) |
| 730.3501 | 1458.686 | 1458.685 | 90 | GSAGPPGATGFPGAAGR | 2 Oxidation (P) |
| 730.3501 | 1458.686 | 1458.685 | 81.3 | GSAGPPGATGFPGAAGR | 2 Oxidation (P) |
| 730.3502 | 1458.686 | 1458.685 | 77.91 | GSAGPPGATGFPGAAGR | 2 Oxidation (P) |
| 730.3502 | 1458.686 | 1458.685 | 88.32 | GSAGPPGATGFPGAAGR | 2 Oxidation (P) |
| 730.3502 | 1458.686 | 1458.685 | 88.1 | GSAGPPGATGFPGAAGR | 2 Oxidation (P) |
| 730.3503 | 1458.686 | 1458.685 | 48.51 | GSAGPPGATGFPGAAGR | 2 Oxidation (P) |
| 730.3503 | 1458.686 | 1458.685 | 57.13 | GSAGPPGATGFPGAAGR | 2 Oxidation (P) |
| 730.3505 | 1458.686 | 1458.685 | 45.83 | GSAGPPGATGFPGAAGR | 2 Oxidation (P) |
| 730.3508 | 1458.687 | 1458.685 | 46.37 | GSAGPPGATGFPGAAGR | 2 Oxidation (P) |
| 730.351 | 1458.687 | 1458.685 | 49.69 | GSAGPPGATGFPGAAGR | 2 Oxidation (P) |
| 730.3511 | 1458.688 | 1458.685 | 60.45 | GSAGPPGATGFPGAAGR | 2 Oxidation (P) |
| 730.3515 | 1458.688 | 1458.685 | 51.44 | GSAGPPGATGFPGAAGR | 2 Oxidation (P) |
| 745.86 | 1489.705 | 1489.705 | 49.05 | PGEVGPPGPPGPAGEK | 3 Oxidation (P) |
| 745.8601 | 1489.706 | 1489.705 | 42.14 | PGEVGPPGPPGPAGEK | Oxidation (K); 2 Oxidation (P) |
| 753.8578 | 1505.701 | 1505.7 | 44.15 | PGEVGPPGPPGPAGEK | Oxidation (K); 3 Oxidation (P) |
| 520.942 | 1559.804 | 1559.806 | 61.27 | GETGPAGPAGPIGPVGAR |  |
| 780.9107 | 1559.807 | 1559.806 | 42.27 | GETGPAGPAGPIGPVGAR |  |
| 521.2705 | 1560.79 | 1560.79 | 53.27 | DGLNGLPGPIGPPGPR | 3 Oxidation (P) |
| 521.2706 | 1560.79 | 1560.79 | 48.85 | DGLNGLPGPIGPPGPR | 3 Oxidation (P) |
| 781.4024 | 1560.79 | 1560.79 | 46.46 | DGLNGLPGPIGPPGPR | 3 Oxidation (P) |
| 781.4025 | 1560.79 | 1560.79 | 40.52 | DGLNGLPGPIGPPGPR | 3 Oxidation (P) |
| 781.8918 | 1561.769 | 1561.774 | 40.02 | DGLNGLPGPIGPPGPR | Deamidated (NQ); 3 Oxidation (P) |
| 781.8941 | 1561.774 | 1561.774 | 53.67 | DGLNGLPGPIGPPGPR | Deamidated (NQ); 3 Oxidation (P) |
| 781.8941 | 1561.774 | 1561.774 | 49.46 | DGLNGLPGPIGPPGPR | Deamidated (NQ); 3 Oxidation (P) |
| 781.8942 | 1561.774 | 1561.774 | 45.13 | DGLNGLPGPIGPPGPR | Deamidated (NQ); 3 Oxidation (P) |
| 781.8945 | 1561.774 | 1561.774 | 51.01 | DGLNGLPGPIGPPGPR | Deamidated (NQ); 3 Oxidation (P) |
| 781.8946 | 1561.775 | 1561.774 | 51.77 | DGLNGLPGPIGPPGPR | Deamidated (NQ); 3 Oxidation (P) |
| 781.8947 | 1561.775 | 1561.774 | 47.21 | DGLNGLPGPIGPPGPR | Deamidated (NQ); 3 Oxidation (P) |
| 781.8959 | 1561.777 | 1561.774 | 41.15 | DGLNGLPGPIGPPGPR | Deamidated (NQ); 3 Oxidation (P) |
| 781.8961 | 1561.778 | 1561.774 | 57.64 | DGLNGLPGPIGPPGPR | Deamidated (NQ); 3 Oxidation (P) |
| 788.9074 | 1575.8 | 1575.801 | 64.14 | GETGPAGPAGPIGPVGAR | Oxidation (P) |
| 788.9078 | 1575.801 | 1575.801 | 83.2 | GETGPAGPAGPIGPVGAR | Oxidation (P) |
| 789.8925 | 1577.77 | 1577.769 | 42.27 | DGLNGLPGPIGPPGPR | Deamidated (NQ); 4 Oxidation (P) |
| 793.3895 | 1584.764 | 1584.765 | 61.61 | GANGAPGIAGAPGFPGAR | 3 Oxidation (P) |
| 793.3896 | 1584.765 | 1584.765 | 60.05 | GANGAPGIAGAPGFPGAR | 3 Oxidation (P) |
| 793.8812 | 1585.748 | 1585.749 | 56.88 | GANGAPGIAGAPGFPGAR | Deamidated (NQ); 3 Oxidation (P) |
| 793.8812 | 1585.748 | 1585.749 | 65.21 | GANGAPGIAGAPGFPGAR | Deamidated (NQ); 3 Oxidation (P) |
| 809.3976 | 1616.781 | 1616.78 | 81.3 | GFSGLDGAKGDAGPAGPK | Oxidation (K) |
| 828.4105 | 1654.806 | 1654.806 | 57.74 | GFPGADGVAGPKGPAGER | Oxidation (P) |
| 828.411 | 1654.807 | 1654.806 | 54.16 | GFPGADGVAGPKGPAGER | Oxidation (P) |
| 845.8928 | 1689.771 | 1689.771 | 63.96 | DGEAGAQGPPGPAGPAGER |  |
| 845.8929 | 1689.771 | 1689.771 | 83.55 | DGEAGAQGPPGPAGPAGER |  |
| 846.3843 | 1690.754 | 1690.755 | 44.67 | DGEAGAQGPPGPAGPAGER | Deamidated (NQ) |
| 846.3846 | 1690.755 | 1690.755 | 77.96 | DGEAGAQGPPGPAGPAGER | Deamidated (NQ) |
| 846.3848 | 1690.755 | 1690.755 | 70.64 | DGEAGAQGPPGPAGPAGER | Deamidated (NQ) |
| 853.8905 | 1705.766 | 1705.766 | 43.15 | DGEAGAQGPPGPAGPAGER | Oxidation (P) |
| 853.8907 | 1705.767 | 1705.766 | 45.8 | DGEAGAQGPPGPAGPAGER | Oxidation (P) |
| 853.8907 | 1705.767 | 1705.766 | 80.11 | DGEAGAQGPPGPAGPAGER | Oxidation (P) |
| 853.8907 | 1705.767 | 1705.766 | 80.06 | DGEAGAQGPPGPAGPAGER | Oxidation (P) |
| 853.8909 | 1705.767 | 1705.766 | 89.06 | DGEAGAQGPPGPAGPAGER | Oxidation (P) |
| 853.8909 | 1705.767 | 1705.766 | 86.09 | DGEAGAQGPPGPAGPAGER | Oxidation (P) |
| 854.3825 | 1706.75 | 1706.75 | 104.81 | DGEAGAQGPPGPAGPAGER | Deamidated (NQ); Oxidation (P) |
| 854.3826 | 1706.751 | 1706.75 | 95.94 | DGEAGAQGPPGPAGPAGER | Deamidated (NQ); Oxidation (P) |
| 907.4391 | 1812.864 | 1812.864 | 42.52 | VGPPGPSGNAGPPGPPGPAGK | Deamidated (NQ); Oxidation (K); 2 Oxidation (P) |
| 907.44 | 1812.865 | 1812.864 | 48.61 | VGPPGPSGNAGPPGPPGPAGK | Deamidated (NQ); Oxidation (K); 2 Oxidation (P) |
| 908.9362 | 1815.858 | 1815.857 | 74.49 | GPPGPMGPPGLAGPPGESGR | Oxidation (M); Oxidation (P) |
| 908.9363 | 1815.858 | 1815.857 | 96.56 | GPPGPMGPPGLAGPPGESGR | Oxidation (M); Oxidation (P) |
| 908.9363 | 1815.858 | 1815.857 | 86.7 | GPPGPMGPPGLAGPPGESGR | Oxidation (M); Oxidation (P) |
| 908.9367 | 1815.859 | 1815.857 | 59.23 | GPPGPMGPPGLAGPPGESGR | Oxidation (M); Oxidation (P) |
| 915.4373 | 1828.86 | 1828.859 | 47.16 | VGPPGPSGNAGPPGPPGPAGK | Deamidated (NQ); 4 Oxidation (P) |
| 916.933 | 1831.851 | 1831.852 | 56.03 | GPPGPMGPPGLAGPPGESGR | Oxidation (M); 2 Oxidation (P) |
| 916.9333 | 1831.852 | 1831.852 | 86.06 | GPPGPMGPPGLAGPPGESGR | Oxidation (M); 2 Oxidation (P) |
| 916.9334 | 1831.852 | 1831.852 | 80.79 | GPPGPMGPPGLAGPPGESGR | Oxidation (M); 2 Oxidation (P) |
| 916.9335 | 1831.852 | 1831.852 | 75.67 | GPPGPMGPPGLAGPPGESGR | Oxidation (M); 2 Oxidation (P) |
| 611.6248 | 1831.853 | 1831.852 | 46.6 | GPPGPMGPPGLAGPPGESGR | Oxidation (M); 2 Oxidation (P) |
| 916.9337 | 1831.853 | 1831.852 | 41.39 | GPPGPMGPPGLAGPPGESGR | Oxidation (M); 2 Oxidation (P) |
| 916.9338 | 1831.853 | 1831.852 | 59.62 | GPPGPMGPPGLAGPPGESGR | 3 Oxidation (P) |
| 916.9339 | 1831.853 | 1831.852 | 71.63 | GPPGPMGPPGLAGPPGESGR | Oxidation (M); 2 Oxidation (P) |
| 924.9312 | 1847.848 | 1847.847 | 42.58 | GPPGPMGPPGLAGPPGESGR | Oxidation (M); 3 Oxidation (P) |
| 924.9313 | 1847.848 | 1847.847 | 46.5 | GPPGPMGPPGLAGPPGESGR | Oxidation (M); 3 Oxidation (P) |
| 924.9313 | 1847.848 | 1847.847 | 47.52 | GPPGPMGPPGLAGPPGESGR | Oxidation (M); 3 Oxidation (P) |
| 924.9314 | 1847.848 | 1847.847 | 53.63 | GPPGPMGPPGLAGPPGESGR | Oxidation (M); 3 Oxidation (P) |
| 924.9317 | 1847.849 | 1847.847 | 55.76 | GPPGPMGPPGLAGPPGESGR | Oxidation (M); 3 Oxidation (P) |
| 932.4399 | 1862.865 | 1862.865 | 66.49 | GEPGPTGIQGPPGPAGEEGK | 2 Oxidation (P) |
| 932.4401 | 1862.866 | 1862.865 | 64.01 | GEPGPTGIQGPPGPAGEEGK | 2 Oxidation (P) |
| 932.9284 | 1863.842 | 1863.842 | 43.08 | GPPGPMGPPGLAGPPGESGR | Oxidation (M); 4 Oxidation (P) |
| 932.9288 | 1863.843 | 1863.842 | 52.28 | GPPGPMGPPGLAGPPGESGR | 5 Oxidation (P) |
| 932.9292 | 1863.844 | 1863.842 | 49.28 | GPPGPMGPPGLAGPPGESGR | 5 Oxidation (P) |
| 932.9316 | 1863.849 | 1863.849 | 54.91 | GEPGPTGIQGPPGPAGEEGK | Deamidated (NQ); 2 Oxidation (P) |
| 932.9317 | 1863.849 | 1863.849 | 60.44 | GEPGPTGIQGPPGPAGEEGK | Deamidated (NQ); 2 Oxidation (P) |
| 659.3361 | 1974.987 | 1974.987 | 44.31 | SGDRGETGPAGPAGPIGPVGAR | |
| 988.5006 | 1974.987 | 1974.987 | 67.63 | SGDRGETGPAGPAGPIGPVGAR | |
| 659.3362 | 1974.987 | 1974.987 | 56.58 | SGDRGETGPAGPAGPIGPVGAR | |
| 659.3362 | 1974.987 | 1974.987 | 48.81 | SGDRGETGPAGPAGPIGPVGAR | |
| 988.501 | 1974.987 | 1974.987 | 59.83 | SGDRGETGPAGPAGPIGPVGAR | |
| 988.5011 | 1974.988 | 1974.987 | 51.95 | SGDRGETGPAGPAGPIGPVGAR | |
| 664.6679 | 1990.982 | 1990.982 | 50.57 | SGDRGETGPAGPAGPIGPVGAR | Oxidation (P) |
| 996.4987 | 1990.983 | 1990.982 | 52.49 | SGDRGETGPAGPAGPIGPVGAR | Oxidation (P) |
| 1010.491 | 2018.967 | 2018.966 | 48.26 | GEPGPTGIQGPPGPAGEEGKR | 2 Oxidation (P) |
| 1010.491 | 2018.967 | 2018.966 | 57.64 | GEPGPTGIQGPPGPAGEEGKR | 2 Oxidation (P) |
| 1010.492 | 2018.969 | 2018.966 | 44.21 | GEPGPTGIQGPPGPAGEEGKR | 2 Oxidation (P) |
| 674.3242 | 2019.951 | 2019.95 | 42.08 | GEPGPTGIQGPPGPAGEEGKR | Deamidated (NQ); 2 Oxidation (P) |
| 1037.01 | 2072.005 | 2072.004 | 64.18 | GAPGADGPAGAPGTPGPQGIAGQR | Oxidation (P) |
| 692.0034 | 2072.988 | 2072.988 | 43.61 | GAPGADGPAGAPGTPGPQGIAGQR | Deamidated (NQ); Oxidation (P) |
| 1037.502 | 2072.989 | 2072.988 | 73.5 | GAPGADGPAGAPGTPGPQGIAGQR | Deamidated (NQ); Oxidation (P) |
| 1037.502 | 2072.989 | 2072.988 | 60.51 | GAPGADGPAGAPGTPGPQGIAGQR | Deamidated (NQ); Oxidation (P) |
| 1037.502 | 2072.989 | 2072.988 | 65.66 | GAPGADGPAGAPGTPGPQGIAGQR | Deamidated (NQ); Oxidation (P) |
| 1037.502 | 2072.989 | 2072.988 | 58.22 | GAPGADGPAGAPGTPGPQGIAGQR | Deamidated (NQ); Oxidation (P) |
| 1037.993 | 2073.971 | 2073.972 | 95.04 | GAPGADGPAGAPGTPGPQGIAGQR | 2 Deamidated (NQ); Oxidation (P) |
| 1037.994 | 2073.973 | 2073.972 | 97.19 | GAPGADGPAGAPGTPGPQGIAGQR | 2 Deamidated (NQ); Oxidation (P) |
| 1037.994 | 2073.973 | 2073.972 | 41.39 | GAPGADGPAGAPGTPGPQGIAGQR | 2 Deamidated (NQ); Oxidation (P) |
| 692.3319 | 2073.974 | 2073.972 | 40.38 | GAPGADGPAGAPGTPGPQGIAGQR | 2 Deamidated (NQ); Oxidation (P) |
| 1045.007 | 2087.999 | 2087.999 | 53.99 | GAPGADGPAGAPGTPGPQGIAGQR | 2 Oxidation (P) |
| 1045.008 | 2088.001 | 2087.999 | 45.99 | GAPGADGPAGAPGTPGPQGIAGQR | 2 Oxidation (P) |
| 697.6625 | 2089.966 | 2089.967 | 53.47 | GAPGADGPAGAPGTPGPQGIAGQR | 2 Deamidated (NQ); 2 Oxidation (P) |
| 1045.991 | 2089.967 | 2089.967 | 57.38 | GAPGADGPAGAPGTPGPQGIAGQR | 2 Deamidated (NQ); 2 Oxidation (P) |
| 1045.991 | 2089.967 | 2089.967 | 77.12 | GAPGADGPAGAPGTPGPQGIAGQR | 2 Deamidated (NQ); 2 Oxidation (P) |
| 1053.496 | 2104.977 | 2104.977 | 50.96 | GAPGADGPAGAPGTPGPQGIAGQR | Deamidated (NQ); 3 Oxidation (P) |
| 1053.497 | 2104.979 | 2104.977 | 62.88 | GAPGADGPAGAPGTPGPQGIAGQR | Deamidated (NQ); 3 Oxidation (P) |
| 1053.989 | 2105.963 | 2105.961 | 71.19 | GAPGADGPAGAPGTPGPQGIAGQR | 2 Deamidated (NQ); 3 Oxidation (P) |
| 1053.989 | 2105.963 | 2105.961 | 73.31 | GAPGADGPAGAPGTPGPQGIAGQR | 2 Deamidated (NQ); 3 Oxidation (P) |
| 712.3275 | 2133.961 | 2133.96 | 45.05 | GEPGPPGPAGFAGPPGADGQPGAK | Deamidated (NQ); Oxidation (K); 2 Oxidation (P) |
| 717.6586 | 2149.954 | 2149.955 | 44.41 | GEPGPPGPAGFAGPPGADGQPGAK | Deamidated (NQ); 4 Oxidation (P) |
| 1077.036 | 2152.057 | 2152.055 | 75.44 | GETGPAGPPGAPGAPGAPGPVGPAGK | 2 Oxidation (P) |
| 1077.036 | 2152.057 | 2152.055 | 70.9 | GETGPAGPPGAPGAPGAPGPVGPAGK | 2 Oxidation (P) |
| 1085.033 | 2168.051 | 2168.05 | 58.74 | GETGPAGPPGAPGAPGAPGPVGPAGK | 3 Oxidation (P) |
| 1085.033 | 2168.051 | 2168.05 | 76.39 | GETGPAGPPGAPGAPGAPGPVGPAGK | 3 Oxidation (P) |
| 1085.033 | 2168.051 | 2168.05 | 67.75 | GETGPAGPPGAPGAPGAPGPVGPAGK | 3 Oxidation (P) |
| 723.6913 | 2168.052 | 2168.05 | 40.17 | GETGPAGPPGAPGAPGAPGPVGPAGK | 3 Oxidation (P) |
| 723.692 | 2168.054 | 2168.05 | 47.22 | GETGPAGPPGAPGAPGAPGPVGPAGK | 3 Oxidation (P) |
| 1093.03 | 2184.045 | 2184.045 | 58.37 | GETGPAGPPGAPGAPGAPGPVGPAGK | 4 Oxidation (P) |
| 1093.031 | 2184.047 | 2184.045 | 48.24 | GETGPAGPPGAPGAPGAPGPVGPAGK | 4 Oxidation (P) |
| 1107.988 | 2213.961 | 2213.961 | 66.26 | GDAGAPGAPGSQGAPGLQGMPGER | Oxidation (M); 4 Oxidation (P) |
| 1107.988 | 2213.961 | 2213.961 | 76.01 | GDAGAPGAPGSQGAPGLQGMPGER | Oxidation (M); 4 Oxidation (P) |
| 1108.48 | 2214.945 | 2214.945 | 47.09 | GDAGAPGAPGSQGAPGLQGMPGER | Deamidated (NQ); Oxidation (M); 4 Oxidation (P) |
| 1108.481 | 2214.947 | 2214.945 | 58.45 | GDAGAPGAPGSQGAPGLQGMPGER | Deamidated (NQ); Oxidation (M); 4 Oxidation (P) |
| 1108.533 | 2215.051 | 2215.051 | 40.3 | GETGPAGRPGEVGPPGPPGPAGEK | Oxidation (K); 2 Oxidation (P) |
| 1108.972 | 2215.929 | 2215.929 | 78.73 | GDAGAPGAPGSQGAPGLQGMPGER | 2 Deamidated (NQ); Oxidation (M); 4 Oxidation (P) |
| 1108.972 | 2215.929 | 2215.929 | 87.37 | GDAGAPGAPGSQGAPGLQGMPGER | 2 Deamidated (NQ); Oxidation (M); 4 Oxidation (P) |
| 1128.531 | 2255.047 | 2255.046 | 47.12 | GFPGLPGPSGEPGKQGPSGASGER | Deamidated (NQ); 2 Oxidation (P) |
| 1136.526 | 2271.037 | 2271.04 | 43.31 | GFPGLPGPSGEPGKQGPSGASGER | Deamidated (NQ); 3 Oxidation (P) |
| 1146.075 | 2290.135 | 2290.134 | 50.53 | GDAGPPGPAGPAGPPGPIGNVGAPGPK | Oxidation (K); Oxidation (P) |
| 764.7126 | 2291.116 | 2291.118 | 49.67 | GDAGPPGPAGPAGPPGPIGNVGAPGPK | Deamidated (NQ); Oxidation (K); Oxidation (P) |
| 1146.567 | 2291.119 | 2291.118 | 85.67 | GDAGPPGPAGPAGPPGPIGNVGAPGPK | Deamidated (NQ); Oxidation (K); Oxidation (P) |
| 1146.567 | 2291.119 | 2291.118 | 92.77 | GDAGPPGPAGPAGPPGPIGNVGAPGPK | Deamidated (NQ); Oxidation (K); Oxidation (P) |
| 772.6887 | 2315.044 | 2315.042 | 42.54 | GEPGPPGPAGAAGPAGNPGADGQPGAK | 4 Oxidation (P) |
| 1159.021 | 2316.027 | 2316.026 | 60.5 | GEPGPPGPAGAAGPAGNPGADGQPGAK | Deamidated (NQ); 4 Oxidation (P) |
| 1159.021 | 2316.027 | 2316.026 | 72.44 | GEPGPPGPAGAAGPAGNPGADGQPGAK | Deamidated (NQ); 4 Oxidation (P) |
| 1159.512 | 2317.009 | 2317.01 | 63.36 | GEPGPPGPAGAAGPAGNPGADGQPGAK | 2 Deamidated (NQ); 4 Oxidation (P) |
| 1159.512 | 2317.009 | 2317.01 | 56.15 | GEPGPPGPAGAAGPAGNPGADGQPGAK | 2 Deamidated (NQ); 4 Oxidation (P) |
| 773.3442 | 2317.011 | 2317.01 | 42.6 | GEPGPPGPAGAAGPAGNPGADGQPGAK | 2 Deamidated (NQ); 4 Oxidation (P) |
| 773.3444 | 2317.011 | 2317.01 | 46.51 | GEPGPPGPAGAAGPAGNPGADGQPGAK | 2 Deamidated (NQ); 4 Oxidation (P) |
| 1162.07 | 2322.125 | 2322.124 | 72.06 | GDAGPPGPAGPAGPPGPIGNVGAPGPK | 4 Oxidation (P) |
| 1162.562 | 2323.109 | 2323.108 | 61.45 | GDAGPPGPAGPAGPPGPIGNVGAPGPK | Deamidated (NQ); 4 Oxidation (P) |
| 819.0793 | 2454.216 | 2454.214 | 63.51 | GPPGSAGSPGKDGLNGLPGPIGPPGPR | Deamidated (NQ); 3 Oxidation (P) |
| 829.4149 | 2485.223 | 2485.22 | 42.79 | GPPGSAGSPGKDGLNGLPGPIGPPGPR | 5 Oxidation (P) |
| 1244.109 | 2486.203 | 2486.204 | 46.61 | GPPGSAGSPGKDGLNGLPGPIGPPGPR | Deamidated (NQ); 5 Oxidation (P) |
| 829.742 | 2486.204 | 2486.204 | 41.01 | GPPGSAGSPGKDGLNGLPGPIGPPGPR | Deamidated (NQ); 5 Oxidation (P) |
| 829.742 | 2486.204 | 2486.204 | 51.34 | GPPGSAGSPGKDGLNGLPGPIGPPGPR | Deamidated (NQ); 5 Oxidation (P) |
| 1244.11 | 2486.205 | 2486.204 | 55.08 | GPPGSAGSPGKDGLNGLPGPIGPPGPR | Deamidated (NQ); 5 Oxidation (P) |
| 1244.111 | 2486.207 | 2486.204 | 56.23 | GPPGSAGSPGKDGLNGLPGPIGPPGPR | Deamidated (NQ); 5 Oxidation (P) |
| 1244.111 | 2486.207 | 2486.204 | 52.47 | GPPGSAGSPGKDGLNGLPGPIGPPGPR | Deamidated (NQ); 5 Oxidation (P) |
| 1244.112 | 2486.209 | 2486.204 | 48.41 | GPPGSAGSPGKDGLNGLPGPIGPPGPR | Deamidated (NQ); 5 Oxidation (P) |
| 1244.112 | 2486.209 | 2486.204 | 43.02 | GPPGSAGSPGKDGLNGLPGPIGPPGPR | Deamidated (NQ); 5 Oxidation (P) |
| 833.0742 | 2496.201 | 2496.199 | 48.24 | GDRGETGPAGPPGAPGAPGAPGPVGPAGK | 3 Oxidation (P) |
| 833.0743 | 2496.201 | 2496.199 | 40.89 | GDRGETGPAGPPGAPGAPGAPGPVGPAGK | 3 Oxidation (P) |
| 838.4058 | 2512.196 | 2512.194 | 45.89 | GDRGETGPAGPPGAPGAPGAPGPVGPAGK | 4 Oxidation (P) |
| 838.4059 | 2512.196 | 2512.194 | 48.54 | GDRGETGPAGPPGAPGAPGAPGPVGPAGK | 4 Oxidation (P) |
| 844.7426 | 2531.206 | 2531.204 | 53.7 | GNDGATGAAGPPGPTGPAGPPGFPGAVGAK | 2 Oxidation (P) |
| 844.7428 | 2531.207 | 2531.204 | 53.82 | GNDGATGAAGPPGPTGPAGPPGFPGAVGAK | Oxidation (K); Oxidation (P) |
| 845.071 | 2532.191 | 2532.188 | 52.34 | GNDGATGAAGPPGPTGPAGPPGFPGAVGAK | Deamidated (NQ); 2 Oxidation (P) |
| 1267.104 | 2532.193 | 2532.188 | 41.5 | GNDGATGAAGPPGPTGPAGPPGFPGAVGAK | Deamidated (NQ); 2 Oxidation (P) |
| 850.0739 | 2547.2 | 2547.199 | 56.32 | GNDGATGAAGPPGPTGPAGPPGFPGAVGAK | 3 Oxidation (P) |
| 1274.608 | 2547.201 | 2547.199 | 71.39 | GNDGATGAAGPPGPTGPAGPPGFPGAVGAK | 3 Oxidation (P) |
| 1274.608 | 2547.201 | 2547.199 | 73.95 | GNDGATGAAGPPGPTGPAGPPGFPGAVGAK | 3 Oxidation (P) |
| 1275.1 | 2548.185 | 2548.183 | 83.82 | GNDGATGAAGPPGPTGPAGPPGFPGAVGAK | Deamidated (NQ); 3 Oxidation (P) |
| 1275.101 | 2548.187 | 2548.183 | 78.29 | GNDGATGAAGPPGPTGPAGPPGFPGAVGAK | Deamidated (NQ); 3 Oxidation (P) |
| 855.7337 | 2564.179 | 2564.178 | 40.82 | GNDGATGAAGPPGPTGPAGPPGFPGAVGAK | Deamidated (NQ); Oxidation (K); 3 Oxidation (P) |
| 1283.097 | 2564.179 | 2564.178 | 76.05 | GNDGATGAAGPPGPTGPAGPPGFPGAVGAK | Deamidated (NQ); Oxidation (K); 3 Oxidation (P) |
| 1283.097 | 2564.179 | 2564.178 | 55.49 | GNDGATGAAGPPGPTGPAGPPGFPGAVGAK | Deamidated (NQ); Oxidation (K); 3 Oxidation (P) |
| 1304.641 | 2607.267 | 2607.268 | 43.59 | GDAGPPGPAGPAGPPGPIGNVGAPGPKGAR | Deamidated (NQ); 4 Oxidation (P) |
| 897.0931 | 2688.258 | 2688.253 | 44.56 | GFSGLQGPPGPPGSPGEQGPSGASGPAGPR | 2 Oxidation (P) |
| 1345.625 | 2689.235 | 2689.237 | 57.38 | GFSGLQGPPGPPGSPGEQGPSGASGPAGPR | Deamidated (NQ); 2 Oxidation (P) |
| 1345.626 | 2689.237 | 2689.237 | 47.07 | GFSGLQGPPGPPGSPGEQGPSGASGPAGPR | Deamidated (NQ); 2 Oxidation (P) |
| 897.4211 | 2689.242 | 2689.237 | 53.05 | GFSGLQGPPGPPGSPGEQGPSGASGPAGPR | Deamidated (NQ); 2 Oxidation (P) |
| 1346.118 | 2690.221 | 2690.221 | 73.46 | GFSGLQGPPGPPGSPGEQGPSGASGPAGPR | 2 Deamidated (NQ); 2 Oxidation (P) |
| 897.7482 | 2690.223 | 2690.221 | 42.72 | GFSGLQGPPGPPGSPGEQGPSGASGPAGPR | 2 Deamidated (NQ); 2 Oxidation (P) |
| 1353.131 | 2704.247 | 2704.248 | 67.76 | GFSGLQGPPGPPGSPGEQGPSGASGPAGPR | 3 Oxidation (P) |
| 902.4232 | 2704.248 | 2704.248 | 49.02 | GFSGLQGPPGPPGSPGEQGPSGASGPAGPR | 3 Oxidation (P) |
| 902.4235 | 2704.249 | 2704.248 | 51.74 | GFSGLQGPPGPPGSPGEQGPSGASGPAGPR | 3 Oxidation (P) |
| 1353.133 | 2704.251 | 2704.248 | 53.88 | GFSGLQGPPGPPGSPGEQGPSGASGPAGPR | 3 Oxidation (P) |
| 902.7502 | 2705.229 | 2705.232 | 46.38 | GFSGLQGPPGPPGSPGEQGPSGASGPAGPR | Deamidated (NQ); 3 Oxidation (P) |
| 1353.623 | 2705.231 | 2705.232 | 45.33 | GFSGLQGPPGPPGSPGEQGPSGASGPAGPR | Deamidated (NQ); 3 Oxidation (P) |
| 902.7513 | 2705.232 | 2705.232 | 53.98 | GFSGLQGPPGPPGSPGEQGPSGASGPAGPR | Deamidated (NQ); 3 Oxidation (P) |
| 902.7515 | 2705.233 | 2705.232 | 47.21 | GFSGLQGPPGPPGSPGEQGPSGASGPAGPR | Deamidated (NQ); 3 Oxidation (P) |
| 1353.624 | 2705.233 | 2705.232 | 64.97 | GFSGLQGPPGPPGSPGEQGPSGASGPAGPR | Deamidated (NQ); 3 Oxidation (P) |
| 1353.624 | 2705.233 | 2705.232 | 97.02 | GFSGLQGPPGPPGSPGEQGPSGASGPAGPR | Deamidated (NQ); 3 Oxidation (P) |
| 1353.624 | 2705.233 | 2705.232 | 65.93 | GFSGLQGPPGPPGSPGEQGPSGASGPAGPR | Deamidated (NQ); 3 Oxidation (P) |
| 1353.624 | 2705.233 | 2705.232 | 65.39 | GFSGLQGPPGPPGSPGEQGPSGASGPAGPR | Deamidated (NQ); 3 Oxidation (P) |
| 1353.624 | 2705.233 | 2705.232 | 48.9 | GFSGLQGPPGPPGSPGEQGPSGASGPAGPR | Deamidated (NQ); 3 Oxidation (P) |
| 1353.625 | 2705.235 | 2705.232 | 43.37 | GFSGLQGPPGPPGSPGEQGPSGASGPAGPR | Deamidated (NQ); 3 Oxidation (P) |
| 903.079 | 2706.215 | 2706.216 | 46.06 | GFSGLQGPPGPPGSPGEQGPSGASGPAGPR | 2 Deamidated (NQ); 3 Oxidation (P) |
| 903.079 | 2706.215 | 2706.216 | 48.73 | GFSGLQGPPGPPGSPGEQGPSGASGPAGPR | 2 Deamidated (NQ); 3 Oxidation (P) |
| 1354.115 | 2706.215 | 2706.216 | 64.1 | GFSGLQGPPGPPGSPGEQGPSGASGPAGPR | 2 Deamidated (NQ); 3 Oxidation (P) |
| 1354.115 | 2706.215 | 2706.216 | 52.62 | GFSGLQGPPGPPGSPGEQGPSGASGPAGPR | 2 Deamidated (NQ); 3 Oxidation (P) |
| 903.0802 | 2706.219 | 2706.216 | 40.79 | GFSGLQGPPGPPGSPGEQGPSGASGPAGPR | 2 Deamidated (NQ); 3 Oxidation (P) |
| 907.4111 | 2719.212 | 2719.211 | 49.3 | GAPGDRGEPGPPGPAGFAGPPGADGQPGAK | Deamidated (NQ); 5 Oxidation (P) |
| 1427.21 | 2852.405 | 2852.405 | 57.33 | GLTGPIGPPGPAGAPGDKGEAGPSGPAGPTGAR | 2 Oxidation (P) |
| 1427.211 | 2852.407 | 2852.405 | 59.62 | GLTGPIGPPGPAGAPGDKGEAGPSGPAGPTGAR | 2 Oxidation (P) |
| 1427.211 | 2852.407 | 2852.405 | 50.11 | GLTGPIGPPGPAGAPGDKGEAGPSGPAGPTGAR | 2 Oxidation (P) |
| 1427.211 | 2852.407 | 2852.405 | 45.32 | GLTGPIGPPGPAGAPGDKGEAGPSGPAGPTGAR | Oxidation (K); Oxidation (P) |
| 1435.208 | 2868.401 | 2868.4 | 48.99 | GLTGPIGPPGPAGAPGDKGEAGPSGPAGPTGAR | Oxidation (K); 2 Oxidation (P) |
| 1435.208 | 2868.401 | 2868.4 | 55.91 | GLTGPIGPPGPAGAPGDKGEAGPSGPAGPTGAR | Oxidation (K); 2 Oxidation (P) |
| 1081.469 | 3241.385 | 3241.381 | 49.96 | GANGAPGNDGAKGDAGAPGAPGSQGAPGLQGMPGER | 2 Deamidated (NQ); Oxidation (M); 5 Oxidation (P) |

| Collagen alpha-1(II) chain OS=Bos taurus GN=COL2A1 PE=1 SV=4 | 342 |
| --- | --- |

| 656.8304 | 1311.646 | 1311.646 | 44.23 | GFPGLPGPSGEPGK | Oxidation (P) |
| --- | --- | --- | --- | --- | --- |
| 656.8307 | 1311.647 | 1311.646 | 48.32 | GFPGLPGPSGEPGK | Oxidation (P) |
| 656.8309 | 1311.647 | 1311.646 | 40.66 | GFPGLPGPSGEPGK | Oxidation (P) |
| 664.8271 | 1327.64 | 1327.641 | 46.5 | GFPGLPGPSGEPGK | 2 Oxidation (P) |
| 664.8273 | 1327.64 | 1327.641 | 70.22 | GFPGLPGPSGEPGK | 2 Oxidation (P) |
| 664.8273 | 1327.64 | 1327.641 | 64.72 | GFPGLPGPSGEPGK | 2 Oxidation (P) |
| 664.8274 | 1327.64 | 1327.641 | 72.59 | GFPGLPGPSGEPGK | 2 Oxidation (P) |
| 664.8275 | 1327.64 | 1327.641 | 60.9 | GFPGLPGPSGEPGK | 2 Oxidation (P) |
| 664.8278 | 1327.641 | 1327.641 | 44.29 | GFPGLPGPSGEPGK | 2 Oxidation (P) |
| 664.8278 | 1327.641 | 1327.641 | 55.15 | GFPGLPGPSGEPGK | 2 Oxidation (P) |
| 664.8278 | 1327.641 | 1327.641 | 56 | GFPGLPGPSGEPGK | 2 Oxidation (P) |
| 664.8279 | 1327.641 | 1327.641 | 42.03 | GFPGLPGPSGEPGK | 2 Oxidation (P) |
| 664.8284 | 1327.642 | 1327.641 | 44.14 | GFPGLPGPSGEPGK | 2 Oxidation (P) |
| 672.8248 | 1343.635 | 1343.636 | 50.79 | GFPGLPGPSGEPGK | 3 Oxidation (P) |
| 672.8249 | 1343.635 | 1343.636 | 48.47 | GFPGLPGPSGEPGK | 3 Oxidation (P) |
| 672.8253 | 1343.636 | 1343.636 | 48.4 | GFPGLPGPSGEPGK | 3 Oxidation (P) |
| 672.8254 | 1343.636 | 1343.636 | 48 | GFPGLPGPSGEPGK | 3 Oxidation (P) |
| 672.8255 | 1343.636 | 1343.636 | 52.69 | GFPGLPGPSGEPGK | 3 Oxidation (P) |
| 758.8602 | 1515.706 | 1515.707 | 62.8 | GAQGPPGATGFPGAAGR | 3 Oxidation (P) |
| 758.8604 | 1515.706 | 1515.707 | 44.94 | GAQGPPGATGFPGAAGR | 3 Oxidation (P) |
| 758.8608 | 1515.707 | 1515.707 | 40.7 | GAQGPPGATGFPGAAGR | 3 Oxidation (P) |

**KC6**

| Collagen alpha-2(I) chain OS=Bos taurus GN=COL1A2 PE=1 SV=2 | | | | 8522 | |
| --- | --- | --- | --- | --- | --- |
| **pep_exp_mz** | **pep_exp_mr** | **pep_calc_mr** | **pep_score** | | **pep_seq** | | **pep_var_mod** |
| 434.7352 | 867.4558 | 867.4563 | 44.39 | | GPSGPQGIR | |  |
| 434.7354 | 867.4562 | 867.4563 | 43.96 | | GPSGPQGIR | |  |
| 435.2272 | 868.4398 | 868.4403 | 46.49 | | GPSGPQGIR | | Deamidated (NQ) |
| 446.7534 | 891.4922 | 891.4926 | 50.27 | | PGPIGPAGAR | |  |
| 446.7536 | 891.4926 | 891.4926 | 42.85 | | PGPIGPAGAR | |  |
| 454.751 | 907.4874 | 907.4876 | 41.6 | | PGPIGPAGAR | | Oxidation (P) |
| 542.785 | 1083.555 | 1083.556 | 45.79 | | GLVGEPGPAGSK | | Oxidation (P) |
| 542.7851 | 1083.556 | 1083.556 | 40.69 | | GLVGEPGPAGSK | | Oxidation (P) |
| 542.7852 | 1083.556 | 1083.556 | 64.49 | | GLVGEPGPAGSK | | Oxidation (P) |
| 542.7853 | 1083.556 | 1083.556 | 62.12 | | GLVGEPGPAGSK | | Oxidation (P) |
| 591.8089 | 1181.603 | 1181.604 | 48.01 | | EGPVGLPGIDGR | | Oxidation (P) |
| 591.809 | 1181.603 | 1181.604 | 46.95 | | EGPVGLPGIDGR | | Oxidation (P) |
| 591.8091 | 1181.604 | 1181.604 | 49.28 | | EGPVGLPGIDGR | | Oxidation (P) |
| 591.8091 | 1181.604 | 1181.604 | 43.24 | | EGPVGLPGIDGR | | Oxidation (P) |
| 591.8093 | 1181.604 | 1181.604 | 51.05 | | EGPVGLPGIDGR | | Oxidation (P) |
| 591.8094 | 1181.604 | 1181.604 | 49.83 | | EGPVGLPGIDGR | | Oxidation (P) |
| 591.8097 | 1181.605 | 1181.604 | 49.74 | | EGPVGLPGIDGR | | Oxidation (P) |
| 591.8098 | 1181.605 | 1181.604 | 42.98 | | EGPVGLPGIDGR | | Oxidation (P) |
| 596.8436 | 1191.673 | 1191.672 | 44.32 | | IGQPGAVGPAGIR | |  |
| 597.3354 | 1192.656 | 1192.656 | 41.46 | | IGQPGAVGPAGIR | | Deamidated (NQ) |
| 597.3354 | 1192.656 | 1192.656 | 58.77 | | IGQPGAVGPAGIR | | Deamidated (NQ) |
| 597.3355 | 1192.656 | 1192.656 | 54.16 | | IGQPGAVGPAGIR | | Deamidated (NQ) |
| 597.3355 | 1192.656 | 1192.656 | 53.67 | | IGQPGAVGPAGIR | | Deamidated (NQ) |
| 597.3357 | 1192.657 | 1192.656 | 58 | | IGQPGAVGPAGIR | | Deamidated (NQ) |
| 601.2958 | 1200.577 | 1200.578 | 61.83 | | GEPGNIGFPGPK | | 2 Oxidation (P) |
| 601.2959 | 1200.577 | 1200.578 | 64.04 | | GEPGNIGFPGPK | | 2 Oxidation (P) |
| 601.2963 | 1200.578 | 1200.578 | 45.01 | | GEPGNIGFPGPK | | 2 Oxidation (P) |
| 601.2966 | 1200.579 | 1200.578 | 43.09 | | GEPGNIGFPGPK | | 2 Oxidation (P) |
| 601.7877 | 1201.561 | 1201.562 | 47.73 | | GEPGNIGFPGPK | | Deamidated (NQ); 2 Oxidation (P) |
| 601.7878 | 1201.561 | 1201.562 | 42.59 | | GEPGNIGFPGPK | | Deamidated (NQ); 2 Oxidation (P) |
| 604.8408 | 1207.667 | 1207.667 | 50.1 | | IGQPGAVGPAGIR | | Oxidation (P) |
| 604.8409 | 1207.667 | 1207.667 | 49.67 | | IGQPGAVGPAGIR | | Oxidation (P) |
| 604.8409 | 1207.667 | 1207.667 | 55.19 | | IGQPGAVGPAGIR | | Oxidation (P) |
| 604.841 | 1207.667 | 1207.667 | 54.12 | | IGQPGAVGPAGIR | | Oxidation (P) |
| 604.8412 | 1207.668 | 1207.667 | 42.3 | | IGQPGAVGPAGIR | | Oxidation (P) |
| 605.3326 | 1208.651 | 1208.651 | 52.81 | | IGQPGAVGPAGIR | | Deamidated (NQ); Oxidation (P) |
| 605.3327 | 1208.651 | 1208.651 | 52.26 | | IGQPGAVGPAGIR | | Deamidated (NQ); Oxidation (P) |
| 605.3327 | 1208.651 | 1208.651 | 44.21 | | IGQPGAVGPAGIR | | Deamidated (NQ); Oxidation (P) |
| 605.3329 | 1208.651 | 1208.651 | 71.02 | | IGQPGAVGPAGIR | | Deamidated (NQ); Oxidation (P) |
| 605.3329 | 1208.651 | 1208.651 | 49.73 | | IGQPGAVGPAGIR | | Deamidated (NQ); Oxidation (P) |
| 605.3329 | 1208.651 | 1208.651 | 42.82 | | IGQPGAVGPAGIR | | Deamidated (NQ); Oxidation (P) |
| 605.3329 | 1208.651 | 1208.651 | 45.84 | | IGQPGAVGPAGIR | | Deamidated (NQ); Oxidation (P) |
| 605.333 | 1208.651 | 1208.651 | 55.28 | | IGQPGAVGPAGIR | | Deamidated (NQ); Oxidation (P) |
| 605.333 | 1208.651 | 1208.651 | 42.6 | | IGQPGAVGPAGIR | | Deamidated (NQ); Oxidation (P) |
| 605.3331 | 1208.652 | 1208.651 | 40.42 | | IGQPGAVGPAGIR | | Deamidated (NQ); Oxidation (P) |
| 605.3331 | 1208.652 | 1208.651 | 56.81 | | IGQPGAVGPAGIR | | Deamidated (NQ); Oxidation (P) |
| 605.3333 | 1208.652 | 1208.651 | 45.09 | | IGQPGAVGPAGIR | | Deamidated (NQ); Oxidation (P) |
| 611.8087 | 1221.603 | 1221.603 | 40.76 | | GFPGTPGLPGFK | | 3 Oxidation (P) |
| 619.8062 | 1237.598 | 1237.598 | 44.38 | | GFPGTPGLPGFK | | Oxidation (K); 3 Oxidation (P) |
| 634.341 | 1266.667 | 1266.668 | 62.73 | | GIPGPVGAAGATGAR | | Oxidation (P) |
| 634.3411 | 1266.668 | 1266.668 | 76.18 | | GIPGPVGAAGATGAR | | Oxidation (P) |
| 634.3412 | 1266.668 | 1266.668 | 100.88 | | GIPGPVGAAGATGAR | | Oxidation (P) |
| 634.3413 | 1266.668 | 1266.668 | 49.08 | | GIPGPVGAAGATGAR | | Oxidation (P) |
| 423.23 | 1266.668 | 1266.668 | 41.12 | | GIPGPVGAAGATGAR | | Oxidation (P) |
| 634.3414 | 1266.668 | 1266.668 | 77.29 | | GIPGPVGAAGATGAR | | Oxidation (P) |
| 634.3414 | 1266.668 | 1266.668 | 54.97 | | GIPGPVGAAGATGAR | | Oxidation (P) |
| 644.3196 | 1286.625 | 1286.626 | 71.8 | | GFPGSPGNIGPAGK | | 2 Oxidation (P) |
| 644.3198 | 1286.625 | 1286.626 | 77.5 | | GFPGSPGNIGPAGK | | 2 Oxidation (P) |
| 644.8113 | 1287.608 | 1287.61 | 81.95 | | GFPGSPGNIGPAGK | | Deamidated (NQ); 2 Oxidation (P) |
| 644.8115 | 1287.608 | 1287.61 | 70.08 | | GFPGSPGNIGPAGK | | Deamidated (NQ); 2 Oxidation (P) |
| 644.8118 | 1287.609 | 1287.61 | 50.79 | | GFPGSPGNIGPAGK | | Deamidated (NQ); 2 Oxidation (P) |
| 463.235 | 1386.683 | 1386.685 | 41.99 | | GETGLRGDIGSPGR | | Oxidation (P) |
| 714.3654 | 1426.716 | 1426.721 | 44.02 | | GIPGEFGLPGPAGAR | | 2 Oxidation (P) |
| 714.3671 | 1426.72 | 1426.721 | 42.47 | | GIPGEFGLPGPAGAR | | 2 Oxidation (P) |
| 714.3672 | 1426.72 | 1426.721 | 58.09 | | GIPGEFGLPGPAGAR | | 2 Oxidation (P) |
| 714.3672 | 1426.72 | 1426.721 | 69.48 | | GIPGEFGLPGPAGAR | | 2 Oxidation (P) |
| 714.3672 | 1426.72 | 1426.721 | 65.04 | | GIPGEFGLPGPAGAR | | 2 Oxidation (P) |
| 714.3673 | 1426.72 | 1426.721 | 59.67 | | GIPGEFGLPGPAGAR | | 2 Oxidation (P) |
| 714.3674 | 1426.72 | 1426.721 | 49.45 | | GIPGEFGLPGPAGAR | | 2 Oxidation (P) |
| 714.3674 | 1426.72 | 1426.721 | 41.84 | | GIPGEFGLPGPAGAR | | 2 Oxidation (P) |
| 714.3676 | 1426.721 | 1426.721 | 43.52 | | GIPGEFGLPGPAGAR | | 2 Oxidation (P) |
| 714.3676 | 1426.721 | 1426.721 | 51.66 | | GIPGEFGLPGPAGAR | | 2 Oxidation (P) |
| 714.3676 | 1426.721 | 1426.721 | 51.5 | | GIPGEFGLPGPAGAR | | 2 Oxidation (P) |
| 714.3677 | 1426.721 | 1426.721 | 55.58 | | GIPGEFGLPGPAGAR | | 2 Oxidation (P) |
| 714.3677 | 1426.721 | 1426.721 | 65.77 | | GIPGEFGLPGPAGAR | | 2 Oxidation (P) |
| 714.3677 | 1426.721 | 1426.721 | 54.73 | | GIPGEFGLPGPAGAR | | 2 Oxidation (P) |
| 714.3677 | 1426.721 | 1426.721 | 46.14 | | GIPGEFGLPGPAGAR | | 2 Oxidation (P) |
| 714.3677 | 1426.721 | 1426.721 | 43.4 | | GIPGEFGLPGPAGAR | | 2 Oxidation (P) |
| 714.3679 | 1426.721 | 1426.721 | 40.54 | | GIPGEFGLPGPAGAR | | 2 Oxidation (P) |
| 714.3679 | 1426.721 | 1426.721 | 56.29 | | GIPGEFGLPGPAGAR | | 2 Oxidation (P) |
| 714.3679 | 1426.721 | 1426.721 | 40.33 | | GIPGEFGLPGPAGAR | | 2 Oxidation (P) |
| 714.3682 | 1426.722 | 1426.721 | 46.39 | | GIPGEFGLPGPAGAR | | 2 Oxidation (P) |
| 714.3682 | 1426.722 | 1426.721 | 43.32 | | GIPGEFGLPGPAGAR | | 2 Oxidation (P) |
| 714.3683 | 1426.722 | 1426.721 | 40.67 | | GIPGEFGLPGPAGAR | | 2 Oxidation (P) |
| 714.3683 | 1426.722 | 1426.721 | 50.21 | | GIPGEFGLPGPAGAR | | 2 Oxidation (P) |
| 714.3684 | 1426.722 | 1426.721 | 41.05 | | GIPGEFGLPGPAGAR | | 2 Oxidation (P) |
| 714.3684 | 1426.722 | 1426.721 | 47.85 | | GIPGEFGLPGPAGAR | | 2 Oxidation (P) |
| 722.3654 | 1442.716 | 1442.715 | 61.38 | | GIPGEFGLPGPAGAR | | 3 Oxidation (P) |
| 729.3422 | 1456.67 | 1456.67 | 88.94 | | GDGGPPGATGFPGAAGR | | Oxidation (P) |
| 729.3422 | 1456.67 | 1456.67 | 84.57 | | GDGGPPGATGFPGAAGR | | Oxidation (P) |
| 737.3384 | 1472.662 | 1472.665 | 70.92 | | GDGGPPGATGFPGAAGR | | 2 Oxidation (P) |
| 737.3386 | 1472.663 | 1472.665 | 45.83 | | GDGGPPGATGFPGAAGR | | 2 Oxidation (P) |
| 737.3389 | 1472.663 | 1472.665 | 74.8 | | GDGGPPGATGFPGAAGR | | 2 Oxidation (P) |
| 737.3389 | 1472.663 | 1472.665 | 44.76 | | GDGGPPGATGFPGAAGR | | 2 Oxidation (P) |
| 737.3391 | 1472.664 | 1472.665 | 52.24 | | GDGGPPGATGFPGAAGR | | 2 Oxidation (P) |
| 737.3393 | 1472.664 | 1472.665 | 65.62 | | GDGGPPGATGFPGAAGR | | 2 Oxidation (P) |
| 746.8491 | 1491.684 | 1491.684 | 72.75 | | SGETGASGPPGFVGEK | | Oxidation (P) |
| 746.8494 | 1491.684 | 1491.684 | 53.27 | | SGETGASGPPGFVGEK | | Oxidation (P) |
| 746.8495 | 1491.684 | 1491.684 | 61.04 | | SGETGASGPPGFVGEK | | Oxidation (P) |
| 746.8495 | 1491.684 | 1491.684 | 62.51 | | SGETGASGPPGFVGEK | | Oxidation (P) |
| 511.5985 | 1531.774 | 1531.774 | 50.92 | | GEPGPAGAVGPAGAVGPR | | Oxidation (P) |
| 511.5986 | 1531.774 | 1531.774 | 53.88 | | GEPGPAGAVGPAGAVGPR | | Oxidation (P) |
| 766.8943 | 1531.774 | 1531.774 | 45.03 | | GEPGPAGAVGPAGAVGPR | | Oxidation (P) |
| 766.8945 | 1531.774 | 1531.774 | 52.64 | | GEPGPAGAVGPAGAVGPR | | Oxidation (P) |
| 766.8945 | 1531.774 | 1531.774 | 45.17 | | GEPGPAGAVGPAGAVGPR | | Oxidation (P) |
| 766.8949 | 1531.775 | 1531.774 | 75.76 | | GEPGPAGAVGPAGAVGPR | | Oxidation (P) |
| 766.8949 | 1531.775 | 1531.774 | 86.92 | | GEPGPAGAVGPAGAVGPR | | Oxidation (P) |
| 769.3723 | 1536.73 | 1536.728 | 52.28 | | GAPGAIGAPGPAGANGDR | | 2 Oxidation (P) |
| 769.8635 | 1537.712 | 1537.712 | 44.93 | | GAPGAIGAPGPAGANGDR | | Deamidated (NQ); 2 Oxidation (P) |
| 781.9175 | 1561.82 | 1561.821 | 68.46 | | GAAGLPGVAGAPGLPGPR | | 3 Oxidation (P) |
| 781.9175 | 1561.82 | 1561.821 | 61.95 | | GAAGLPGVAGAPGLPGPR | | 3 Oxidation (P) |
| 521.6143 | 1561.821 | 1561.821 | 41.04 | | GAAGLPGVAGAPGLPGPR | | 3 Oxidation (P) |
| 521.6143 | 1561.821 | 1561.821 | 42.85 | | GAAGLPGVAGAPGLPGPR | | 3 Oxidation (P) |
| 781.9181 | 1561.822 | 1561.821 | 65.43 | | GAAGLPGVAGAPGLPGPR | | 3 Oxidation (P) |
| 781.9182 | 1561.822 | 1561.821 | 66.15 | | GAAGLPGVAGAPGLPGPR | | 3 Oxidation (P) |
| 790.8865 | 1579.758 | 1579.759 | 54.01 | | GPPGESGAAGPTGPIGSR | | Oxidation (P) |
| 790.8868 | 1579.759 | 1579.759 | 65.78 | | GPPGESGAAGPTGPIGSR | | Oxidation (P) |
| 790.887 | 1579.759 | 1579.759 | 43.66 | | GPPGESGAAGPTGPIGSR | | Oxidation (P) |
| 790.8871 | 1579.76 | 1579.759 | 43.45 | | GPPGESGAAGPTGPIGSR | | Oxidation (P) |
| 790.8871 | 1579.76 | 1579.759 | 79.65 | | GPPGESGAAGPTGPIGSR | | Oxidation (P) |
| 790.8871 | 1579.76 | 1579.759 | 51.91 | | GPPGESGAAGPTGPIGSR | | Oxidation (P) |
| 790.8872 | 1579.76 | 1579.759 | 74.34 | | GPPGESGAAGPTGPIGSR | | Oxidation (P) |
| 790.8873 | 1579.76 | 1579.759 | 58.28 | | GPPGESGAAGPTGPIGSR | | Oxidation (P) |
| 790.8874 | 1579.76 | 1579.759 | 76.44 | | GPPGESGAAGPTGPIGSR | | Oxidation (P) |
| 790.8881 | 1579.762 | 1579.759 | 49.76 | | GPPGESGAAGPTGPIGSR | | Oxidation (P) |
| 800.4153 | 1598.816 | 1598.817 | 71.16 | | GELGPVGNPGPAGPAGPR | |  |
| 800.4157 | 1598.817 | 1598.817 | 72.1 | | GELGPVGNPGPAGPAGPR | |  |
| 800.9077 | 1599.801 | 1599.801 | 66.81 | | GELGPVGNPGPAGPAGPR | | Deamidated (NQ) |
| 800.9083 | 1599.802 | 1599.801 | 53.22 | | GELGPVGNPGPAGPAGPR | | Deamidated (NQ) |
| 808.4131 | 1614.812 | 1614.811 | 73.56 | | GELGPVGNPGPAGPAGPR | | Oxidation (P) |
| 808.4132 | 1614.812 | 1614.811 | 69.92 | | GELGPVGNPGPAGPAGPR | | Oxidation (P) |
| 808.9047 | 1615.795 | 1615.795 | 44.12 | | GELGPVGNPGPAGPAGPR | | Deamidated (NQ); Oxidation (P) |
| 816.9022 | 1631.79 | 1631.79 | 62.73 | | GELGPVGNPGPAGPAGPR | | Deamidated (NQ); 2 Oxidation (P) |
| 816.9023 | 1631.79 | 1631.79 | 59.11 | | GELGPVGNPGPAGPAGPR | | Deamidated (NQ); 2 Oxidation (P) |
| 824.9177 | 1647.821 | 1647.822 | 49.86 | | GSTGEIGPAGPPGPPGLR | | 2 Oxidation (P) |
| 824.9179 | 1647.821 | 1647.822 | 58.29 | | GSTGEIGPAGPPGPPGLR | | 2 Oxidation (P) |
| 824.9182 | 1647.822 | 1647.822 | 69.12 | | GSTGEIGPAGPPGPPGLR | | 2 Oxidation (P) |
| 824.9186 | 1647.823 | 1647.822 | 71.88 | | GSTGEIGPAGPPGPPGLR | | 2 Oxidation (P) |
| 843.9425 | 1685.87 | 1685.87 | 48.59 | | GAAGIPGGKGEKGETGLR | | Oxidation (K); Oxidation (P) |
| 843.9429 | 1685.871 | 1685.87 | 57.34 | | GAAGIPGGKGEKGETGLR | | Oxidation (K); Oxidation (P) |
| 600.6042 | 1798.791 | 1798.79 | 52.98 | | GPNGDSGRPGEPGLMGPR | | Deamidated (NQ); Oxidation (M); 2 Oxidation (P) |
| 600.6042 | 1798.791 | 1798.79 | 42.56 | | GPNGDSGRPGEPGLMGPR | | Deamidated (NQ); Oxidation (M); 2 Oxidation (P) |
| 602.3145 | 1803.922 | 1803.923 | 52.32 | | RGSTGEIGPAGPPGPPGLR | | 2 Oxidation (P) |
| 911.9204 | 1821.826 | 1821.824 | 83.57 | | GPPGNVGNPGVNGAPGEAGR | | Deamidated (NQ); 3 Oxidation (P) |
| 911.9205 | 1821.826 | 1821.824 | 77.89 | | GPPGNVGNPGVNGAPGEAGR | | Deamidated (NQ); 3 Oxidation (P) |
| 912.4119 | 1822.809 | 1822.808 | 74.71 | | GPPGNVGNPGVNGAPGEAGR | | 2 Deamidated (NQ); 3 Oxidation (P) |
| 912.412 | 1822.809 | 1822.808 | 81.29 | | GPPGNVGNPGVNGAPGEAGR | | 2 Deamidated (NQ); 3 Oxidation (P) |
| 912.9038 | 1823.793 | 1823.792 | 80.8 | | GPPGNVGNPGVNGAPGEAGR | | 3 Deamidated (NQ); 3 Oxidation (P) |
| 912.904 | 1823.793 | 1823.792 | 85.03 | | GPPGNVGNPGVNGAPGEAGR | | 3 Deamidated (NQ); 3 Oxidation (P) |
| 915.4551 | 1828.896 | 1828.896 | 49.73 | | TGPPGPSGISGPPGPPGPAGK | | Oxidation (K); 2 Oxidation (P) |
| 915.4552 | 1828.896 | 1828.896 | 51.59 | | TGPPGPSGISGPPGPPGPAGK | | Oxidation (K); 2 Oxidation (P) |
| 915.4553 | 1828.896 | 1828.896 | 49.54 | | TGPPGPSGISGPPGPPGPAGK | | Oxidation (K); 2 Oxidation (P) |
| 915.4553 | 1828.896 | 1828.896 | 46.04 | | TGPPGPSGISGPPGPPGPAGK | | Oxidation (K); 2 Oxidation (P) |
| 666.6685 | 1996.984 | 1996.983 | 57.06 | | HGNRGEPGPAGAVGPAGAVGPR | | Deamidated (NQ); Oxidation (P) |
| 666.6687 | 1996.984 | 1996.983 | 66.1 | | HGNRGEPGPAGAVGPAGAVGPR | | Deamidated (NQ); Oxidation (P) |
| 999.4998 | 1996.985 | 1996.983 | 69.03 | | HGNRGEPGPAGAVGPAGAVGPR | | Deamidated (NQ); Oxidation (P) |
| 684.3363 | 2049.987 | 2049.987 | 54.88 | | GEVGPAGPNGFAGPAGAAGQPGAK | | Oxidation (P) |
| 1026.002 | 2049.989 | 2049.987 | 87.45 | | GEVGPAGPNGFAGPAGAAGQPGAK | | Oxidation (P) |
| 1026.493 | 2050.971 | 2050.971 | 52.27 | | GEVGPAGPNGFAGPAGAAGQPGAK | | Deamidated (NQ); Oxidation (K) |
| 1026.494 | 2050.973 | 2050.971 | 76.55 | | GEVGPAGPNGFAGPAGAAGQPGAK | | Deamidated (NQ); Oxidation (P) |
| 1026.494 | 2050.973 | 2050.971 | 57.34 | | GEVGPAGPNGFAGPAGAAGQPGAK | | Deamidated (NQ); Oxidation (P) |
| 1026.985 | 2051.955 | 2051.955 | 94.26 | | GEVGPAGPNGFAGPAGAAGQPGAK | | 2 Deamidated (NQ); Oxidation (P) |
| 1026.985 | 2051.955 | 2051.955 | 83.81 | | GEVGPAGPNGFAGPAGAAGQPGAK | | 2 Deamidated (NQ); Oxidation (P) |
| 1026.985 | 2051.955 | 2051.955 | 83.13 | | GEVGPAGPNGFAGPAGAAGQPGAK | | 2 Deamidated (NQ); Oxidation (P) |
| 1026.986 | 2051.957 | 2051.955 | 88.62 | | GEVGPAGPNGFAGPAGAAGQPGAK | | 2 Deamidated (NQ); Oxidation (P) |
| 686.0362 | 2055.087 | 2055.086 | 42.17 | | EGPVGLPGIDGRPGPIGPAGAR | | Oxidation (P) |
| 1028.551 | 2055.087 | 2055.086 | 42.64 | | EGPVGLPGIDGRPGPIGPAGAR | | Oxidation (P) |
| 1033.999 | 2065.983 | 2065.982 | 67.02 | | GEVGPAGPNGFAGPAGAAGQPGAK | | Oxidation (K); Oxidation (P) |
| 1034.49 | 2066.965 | 2066.966 | 73.4 | | GEVGPAGPNGFAGPAGAAGQPGAK | | Deamidated (NQ); Oxidation (K); Oxidation (P) |
| 1034.491 | 2066.967 | 2066.966 | 55.53 | | GEVGPAGPNGFAGPAGAAGQPGAK | | Deamidated (NQ); Oxidation (K); Oxidation (P) |
| 1034.982 | 2067.949 | 2067.95 | 78.78 | | GEVGPAGPNGFAGPAGAAGQPGAK | | 2 Deamidated (NQ); Oxidation (K); Oxidation (P) |
| 1034.983 | 2067.951 | 2067.95 | 49.22 | | GEVGPAGPNGFAGPAGAAGQPGAK | | 2 Deamidated (NQ); Oxidation (K); Oxidation (P) |
| 1058.066 | 2114.117 | 2114.112 | 63.84 | | GLPGVAGSVGEPGPLGIAGPPGAR | | 2 Oxidation (P) |
| 1066.057 | 2130.099 | 2130.107 | 70.64 | | GLPGVAGSVGEPGPLGIAGPPGAR | | 3 Oxidation (P) |
| 1066.057 | 2130.099 | 2130.107 | 65.07 | | GLPGVAGSVGEPGPLGIAGPPGAR | | 3 Oxidation (P) |
| 1066.057 | 2130.099 | 2130.107 | 80.73 | | GLPGVAGSVGEPGPLGIAGPPGAR | | 3 Oxidation (P) |
| 1066.06 | 2130.105 | 2130.107 | 69.68 | | GLPGVAGSVGEPGPLGIAGPPGAR | | 3 Oxidation (P) |
| 1066.06 | 2130.105 | 2130.107 | 52.5 | | GLPGVAGSVGEPGPLGIAGPPGAR | | 3 Oxidation (P) |
| 1066.061 | 2130.107 | 2130.107 | 87.94 | | GLPGVAGSVGEPGPLGIAGPPGAR | | 3 Oxidation (P) |
| 1066.061 | 2130.107 | 2130.107 | 51.18 | | GLPGVAGSVGEPGPLGIAGPPGAR | | 3 Oxidation (P) |
| 1066.061 | 2130.107 | 2130.107 | 109.23 | | GLPGVAGSVGEPGPLGIAGPPGAR | | 3 Oxidation (P) |
| 1066.061 | 2130.107 | 2130.107 | 99.48 | | GLPGVAGSVGEPGPLGIAGPPGAR | | 3 Oxidation (P) |
| 1066.061 | 2130.107 | 2130.107 | 46.48 | | GLPGVAGSVGEPGPLGIAGPPGAR | | 3 Oxidation (P) |
| 1066.061 | 2130.107 | 2130.107 | 43.85 | | GLPGVAGSVGEPGPLGIAGPPGAR | | 3 Oxidation (P) |
| 1066.061 | 2130.107 | 2130.107 | 53.03 | | GLPGVAGSVGEPGPLGIAGPPGAR | | 3 Oxidation (P) |
| 1066.061 | 2130.107 | 2130.107 | 71.92 | | GLPGVAGSVGEPGPLGIAGPPGAR | | 3 Oxidation (P) |
| 1066.061 | 2130.107 | 2130.107 | 78.59 | | GLPGVAGSVGEPGPLGIAGPPGAR | | 3 Oxidation (P) |
| 1066.061 | 2130.107 | 2130.107 | 77.21 | | GLPGVAGSVGEPGPLGIAGPPGAR | | 3 Oxidation (P) |
| 1066.061 | 2130.107 | 2130.107 | 57.12 | | GLPGVAGSVGEPGPLGIAGPPGAR | | 3 Oxidation (P) |
| 1066.061 | 2130.107 | 2130.107 | 56.28 | | GLPGVAGSVGEPGPLGIAGPPGAR | | 3 Oxidation (P) |
| 1066.061 | 2130.107 | 2130.107 | 78.44 | | GLPGVAGSVGEPGPLGIAGPPGAR | | 3 Oxidation (P) |
| 1066.061 | 2130.107 | 2130.107 | 86.79 | | GLPGVAGSVGEPGPLGIAGPPGAR | | 3 Oxidation (P) |
| 1066.061 | 2130.107 | 2130.107 | 94.08 | | GLPGVAGSVGEPGPLGIAGPPGAR | | 3 Oxidation (P) |
| 711.0433 | 2130.108 | 2130.107 | 47.97 | | GLPGVAGSVGEPGPLGIAGPPGAR | | 3 Oxidation (P) |
| 711.0434 | 2130.108 | 2130.107 | 59.06 | | GLPGVAGSVGEPGPLGIAGPPGAR | | 3 Oxidation (P) |
| 711.0434 | 2130.108 | 2130.107 | 44.65 | | GLPGVAGSVGEPGPLGIAGPPGAR | | 3 Oxidation (P) |
| 711.0434 | 2130.108 | 2130.107 | 58.45 | | GLPGVAGSVGEPGPLGIAGPPGAR | | 3 Oxidation (P) |
| 711.0436 | 2130.109 | 2130.107 | 56.81 | | GLPGVAGSVGEPGPLGIAGPPGAR | | 3 Oxidation (P) |
| 1066.062 | 2130.109 | 2130.107 | 52.27 | | GLPGVAGSVGEPGPLGIAGPPGAR | | 3 Oxidation (P) |
| 1066.062 | 2130.109 | 2130.107 | 55.75 | | GLPGVAGSVGEPGPLGIAGPPGAR | | 3 Oxidation (P) |
| 1066.062 | 2130.109 | 2130.107 | 103.43 | | GLPGVAGSVGEPGPLGIAGPPGAR | | 3 Oxidation (P) |
| 1066.062 | 2130.109 | 2130.107 | 106.55 | | GLPGVAGSVGEPGPLGIAGPPGAR | | 3 Oxidation (P) |
| 1066.062 | 2130.109 | 2130.107 | 93.23 | | GLPGVAGSVGEPGPLGIAGPPGAR | | 3 Oxidation (P) |
| 1066.062 | 2130.109 | 2130.107 | 99.48 | | GLPGVAGSVGEPGPLGIAGPPGAR | | 3 Oxidation (P) |
| 1066.062 | 2130.109 | 2130.107 | 47.78 | | GLPGVAGSVGEPGPLGIAGPPGAR | | 3 Oxidation (P) |
| 1066.062 | 2130.109 | 2130.107 | 103.75 | | GLPGVAGSVGEPGPLGIAGPPGAR | | 3 Oxidation (P) |
| 1066.062 | 2130.109 | 2130.107 | 55.64 | | GLPGVAGSVGEPGPLGIAGPPGAR | | 3 Oxidation (P) |
| 1066.062 | 2130.109 | 2130.107 | 74.25 | | GLPGVAGSVGEPGPLGIAGPPGAR | | 3 Oxidation (P) |
| 1066.062 | 2130.109 | 2130.107 | 63.73 | | GLPGVAGSVGEPGPLGIAGPPGAR | | 3 Oxidation (P) |
| 1066.062 | 2130.109 | 2130.107 | 65.41 | | GLPGVAGSVGEPGPLGIAGPPGAR | | 3 Oxidation (P) |
| 1066.062 | 2130.109 | 2130.107 | 82.7 | | GLPGVAGSVGEPGPLGIAGPPGAR | | 3 Oxidation (P) |
| 1066.062 | 2130.109 | 2130.107 | 61.71 | | GLPGVAGSVGEPGPLGIAGPPGAR | | 3 Oxidation (P) |
| 1066.062 | 2130.109 | 2130.107 | 67.42 | | GLPGVAGSVGEPGPLGIAGPPGAR | | 3 Oxidation (P) |
| 1066.063 | 2130.111 | 2130.107 | 80.94 | | GLPGVAGSVGEPGPLGIAGPPGAR | | 3 Oxidation (P) |
| 1066.063 | 2130.111 | 2130.107 | 73.71 | | GLPGVAGSVGEPGPLGIAGPPGAR | | 3 Oxidation (P) |
| 1066.063 | 2130.111 | 2130.107 | 59.86 | | GLPGVAGSVGEPGPLGIAGPPGAR | | 3 Oxidation (P) |
| 1066.063 | 2130.111 | 2130.107 | 66.87 | | GLPGVAGSVGEPGPLGIAGPPGAR | | 3 Oxidation (P) |
| 1066.064 | 2130.113 | 2130.107 | 47.16 | | GLPGVAGSVGEPGPLGIAGPPGAR | | 3 Oxidation (P) |
| 1066.064 | 2130.113 | 2130.107 | 58 | | GLPGVAGSVGEPGPLGIAGPPGAR | | 3 Oxidation (P) |
| 1074.058 | 2146.101 | 2146.102 | 54.78 | | GLPGVAGSVGEPGPLGIAGPPGAR | | 4 Oxidation (P) |
| 1074.059 | 2146.103 | 2146.102 | 63.72 | | GLPGVAGSVGEPGPLGIAGPPGAR | | 4 Oxidation (P) |
| 1074.059 | 2146.103 | 2146.102 | 65.71 | | GLPGVAGSVGEPGPLGIAGPPGAR | | 4 Oxidation (P) |
| 1074.059 | 2146.103 | 2146.102 | 51.13 | | GLPGVAGSVGEPGPLGIAGPPGAR | | 4 Oxidation (P) |
| 1074.06 | 2146.105 | 2146.102 | 59.14 | | GLPGVAGSVGEPGPLGIAGPPGAR | | 4 Oxidation (P) |
| 1082.057 | 2162.099 | 2162.097 | 45.65 | | GLPGVAGSVGEPGPLGIAGPPGAR | | 5 Oxidation (P) |
| 1082.058 | 2162.101 | 2162.097 | 48.54 | | GLPGVAGSVGEPGPLGIAGPPGAR | | 5 Oxidation (P) |
| 754.3822 | 2260.125 | 2260.124 | 43.77 | | GYPGNAGPVGAAGAPGPQGPVGPVGK | | 2 Oxidation (P) |
| 1131.07 | 2260.125 | 2260.124 | 74.89 | | GYPGNAGPVGAAGAPGPQGPVGPVGK | | 2 Oxidation (P) |
| 1131.07 | 2260.125 | 2260.124 | 57.46 | | GYPGNAGPVGAAGAPGPQGPVGPVGK | | 2 Oxidation (P) |
| 1131.563 | 2261.111 | 2261.108 | 58.82 | | GYPGNAGPVGAAGAPGPQGPVGPVGK | | Deamidated (NQ); 2 Oxidation (P) |
| 1132.053 | 2262.091 | 2262.092 | 50.85 | | GYPGNAGPVGAAGAPGPQGPVGPVGK | | 2 Deamidated (NQ); 2 Oxidation (P) |
| 1132.053 | 2262.091 | 2262.092 | 63.04 | | GYPGNAGPVGAAGAPGPQGPVGPVGK | | 2 Deamidated (NQ); 2 Oxidation (P) |
| 1205.08 | 2408.145 | 2408.147 | 110.95 | | GEVGPAGPNGFAGPAGAAGQPGAKGER | | Oxidation (K); Oxidation (P) |
| 1205.082 | 2408.149 | 2408.147 | 67.17 | | GEVGPAGPNGFAGPAGAAGQPGAKGER | | Oxidation (K); Oxidation (P) |
| 1205.573 | 2409.131 | 2409.131 | 91.49 | | GEVGPAGPNGFAGPAGAAGQPGAKGER | | Deamidated (NQ); Oxidation (K); Oxidation (P) |
| 1205.573 | 2409.131 | 2409.131 | 100.77 | | GEVGPAGPNGFAGPAGAAGQPGAKGER | | Deamidated (NQ); Oxidation (K); Oxidation (P) |
| 804.3784 | 2410.113 | 2410.115 | 44.63 | | GEVGPAGPNGFAGPAGAAGQPGAKGER | | 2 Deamidated (NQ); Oxidation (K); Oxidation (P) |
| 1206.065 | 2410.115 | 2410.115 | 89 | | GEVGPAGPNGFAGPAGAAGQPGAKGER | | 2 Deamidated (NQ); Oxidation (K); Oxidation (P) |
| 1206.066 | 2410.117 | 2410.115 | 93.84 | | GEVGPAGPNGFAGPAGAAGQPGAKGER | | 2 Deamidated (NQ); Oxidation (K); Oxidation (P) |
| 1215.617 | 2429.219 | 2429.219 | 61.24 | | GEVGLPGLSGPVGPPGNPGANGLPGAK | | 4 Oxidation (P) |
| 1215.617 | 2429.219 | 2429.219 | 58.95 | | GEVGLPGLSGPVGPPGNPGANGLPGAK | | 4 Oxidation (P) |
| 1215.617 | 2429.219 | 2429.219 | 66.5 | | GEVGLPGLSGPVGPPGNPGANGLPGAK | | 4 Oxidation (P) |
| 810.7474 | 2429.22 | 2429.219 | 52.66 | | GEVGLPGLSGPVGPPGNPGANGLPGAK | | 4 Oxidation (P) |
| 1215.618 | 2429.221 | 2429.219 | 62.14 | | GEVGLPGLSGPVGPPGNPGANGLPGAK | | 4 Oxidation (P) |
| 1216.108 | 2430.201 | 2430.203 | 66.71 | | GEVGLPGLSGPVGPPGNPGANGLPGAK | | Deamidated (NQ); 4 Oxidation (P) |
| 1216.109 | 2430.203 | 2430.203 | 70.12 | | GEVGLPGLSGPVGPPGNPGANGLPGAK | | Deamidated (NQ); 4 Oxidation (P) |
| 811.0751 | 2430.204 | 2430.203 | 67.47 | | GEVGLPGLSGPVGPPGNPGANGLPGAK | | Deamidated (NQ); 4 Oxidation (P) |
| 811.0754 | 2430.204 | 2430.203 | 46.23 | | GEVGLPGLSGPVGPPGNPGANGLPGAK | | Deamidated (NQ); 4 Oxidation (P) |
| 811.0756 | 2430.205 | 2430.203 | 42.06 | | GEVGLPGLSGPVGPPGNPGANGLPGAK | | Deamidated (NQ); 4 Oxidation (P) |
| 1216.601 | 2431.187 | 2431.187 | 63.55 | | GEVGLPGLSGPVGPPGNPGANGLPGAK | | 2 Deamidated (NQ); 4 Oxidation (P) |
| 1216.602 | 2431.189 | 2431.187 | 83.07 | | GEVGLPGLSGPVGPPGNPGANGLPGAK | | 2 Deamidated (NQ); 4 Oxidation (P) |
| 1216.602 | 2431.189 | 2431.187 | 74.8 | | GEVGLPGLSGPVGPPGNPGANGLPGAK | | 2 Deamidated (NQ); 4 Oxidation (P) |
| 1216.602 | 2431.189 | 2431.187 | 77.46 | | GEVGLPGLSGPVGPPGNPGANGLPGAK | | 2 Deamidated (NQ); 4 Oxidation (P) |
| 1223.615 | 2445.215 | 2445.214 | 65.5 | | GEVGLPGLSGPVGPPGNPGANGLPGAK | | Oxidation (K); 4 Oxidation (P) |
| 1224.107 | 2446.199 | 2446.198 | 65.98 | | GEVGLPGLSGPVGPPGNPGANGLPGAK | | Deamidated (NQ); Oxidation (K); 4 Oxidation (P) |
| 1224.107 | 2446.199 | 2446.198 | 70.18 | | GEVGLPGLSGPVGPPGNPGANGLPGAK | | Deamidated (NQ); Oxidation (K); 4 Oxidation (P) |
| 816.4073 | 2446.2 | 2446.198 | 45.35 | | GEVGLPGLSGPVGPPGNPGANGLPGAK | | Deamidated (NQ); Oxidation (K); 4 Oxidation (P) |
| 1224.6 | 2447.185 | 2447.182 | 56.15 | | GEVGLPGLSGPVGPPGNPGANGLPGAK | | 2 Deamidated (NQ); Oxidation (K); 4 Oxidation (P) |
| 1224.6 | 2447.185 | 2447.182 | 53.31 | | GEVGLPGLSGPVGPPGNPGANGLPGAK | | 2 Deamidated (NQ); Oxidation (K); 4 Oxidation (P) |
| 1224.602 | 2447.189 | 2447.182 | 68.59 | | GEVGLPGLSGPVGPPGNPGANGLPGAK | | 2 Deamidated (NQ); Oxidation (K); 4 Oxidation (P) |
| 1224.604 | 2447.193 | 2447.182 | 71.29 | | GEVGLPGLSGPVGPPGNPGANGLPGAK | | 2 Deamidated (NQ); Oxidation (K); 4 Oxidation (P) |
| 858.4257 | 2572.255 | 2572.256 | 58.77 | | GSDGSVGPVGPAGPIGSAGPPGFPGAPGPK | | 2 Oxidation (P) |
| 858.4261 | 2572.257 | 2572.256 | 48.27 | | GSDGSVGPVGPAGPIGSAGPPGFPGAPGPK | | Oxidation (K); Oxidation (P) |
| 858.4262 | 2572.257 | 2572.256 | 43.54 | | GSDGSVGPVGPAGPIGSAGPPGFPGAPGPK | | Oxidation (K); Oxidation (P) |
| 1287.136 | 2572.257 | 2572.256 | 95.57 | | GSDGSVGPVGPAGPIGSAGPPGFPGAPGPK | | Oxidation (K); Oxidation (P) |
| 1287.136 | 2572.257 | 2572.256 | 50.53 | | GSDGSVGPVGPAGPIGSAGPPGFPGAPGPK | | Oxidation (K); Oxidation (P) |
| 1287.136 | 2572.257 | 2572.256 | 56.39 | | GSDGSVGPVGPAGPIGSAGPPGFPGAPGPK | | Oxidation (K); Oxidation (P) |
| 1287.136 | 2572.257 | 2572.256 | 43.75 | | GSDGSVGPVGPAGPIGSAGPPGFPGAPGPK | | Oxidation (K); Oxidation (P) |
| 1287.137 | 2572.259 | 2572.256 | 97.45 | | GSDGSVGPVGPAGPIGSAGPPGFPGAPGPK | | Oxidation (K); Oxidation (P) |
| 1287.137 | 2572.259 | 2572.256 | 102.09 | | GSDGSVGPVGPAGPIGSAGPPGFPGAPGPK | | Oxidation (K); Oxidation (P) |
| 1287.137 | 2572.259 | 2572.256 | 112.9 | | GSDGSVGPVGPAGPIGSAGPPGFPGAPGPK | | Oxidation (K); Oxidation (P) |
| 861.0847 | 2580.232 | 2580.232 | 70.97 | | GENGPVGPTGPVGAAGPSGPNGPPGPAGSR | | Oxidation (P) |
| 861.0849 | 2580.233 | 2580.232 | 71.03 | | GENGPVGPTGPVGAAGPSGPNGPPGPAGSR | | Oxidation (P) |
| 1291.124 | 2580.233 | 2580.232 | 87.04 | | GENGPVGPTGPVGAAGPSGPNGPPGPAGSR | | Oxidation (P) |
| 1291.124 | 2580.233 | 2580.232 | 94.14 | | GENGPVGPTGPVGAAGPSGPNGPPGPAGSR | | Oxidation (P) |
| 1291.616 | 2581.217 | 2581.216 | 80.74 | | GENGPVGPTGPVGAAGPSGPNGPPGPAGSR | | Deamidated (NQ); Oxidation (P) |
| 1291.616 | 2581.217 | 2581.216 | 67.61 | | GENGPVGPTGPVGAAGPSGPNGPPGPAGSR | | Deamidated (NQ); Oxidation (P) |
| 861.7402 | 2582.199 | 2582.2 | 44.87 | | GENGPVGPTGPVGAAGPSGPNGPPGPAGSR | | 2 Deamidated (NQ); Oxidation (P) |
| 861.7406 | 2582.2 | 2582.2 | 63.64 | | GENGPVGPTGPVGAAGPSGPNGPPGPAGSR | | 2 Deamidated (NQ); Oxidation (P) |
| 861.7408 | 2582.201 | 2582.2 | 52.88 | | GENGPVGPTGPVGAAGPSGPNGPPGPAGSR | | 2 Deamidated (NQ); Oxidation (P) |
| 1292.109 | 2582.203 | 2582.2 | 83.34 | | GENGPVGPTGPVGAAGPSGPNGPPGPAGSR | | 2 Deamidated (NQ); Oxidation (P) |
| 1292.11 | 2582.205 | 2582.2 | 74.2 | | GENGPVGPTGPVGAAGPSGPNGPPGPAGSR | | 2 Deamidated (NQ); Oxidation (P) |
| 861.7435 | 2582.209 | 2582.2 | 69.35 | | GENGPVGPTGPVGAAGPSGPNGPPGPAGSR | | 2 Deamidated (NQ); Oxidation (P) |
| 1295.133 | 2588.251 | 2588.251 | 80.87 | | GSDGSVGPVGPAGPIGSAGPPGFPGAPGPK | | 3 Oxidation (P) |
| 1295.133 | 2588.251 | 2588.251 | 72.9 | | GSDGSVGPVGPAGPIGSAGPPGFPGAPGPK | | Oxidation (K); 2 Oxidation (P) |
| 1295.133 | 2588.251 | 2588.251 | 68.92 | | GSDGSVGPVGPAGPIGSAGPPGFPGAPGPK | | Oxidation (K); 2 Oxidation (P) |
| 1295.133 | 2588.251 | 2588.251 | 55.13 | | GSDGSVGPVGPAGPIGSAGPPGFPGAPGPK | | Oxidation (K); 2 Oxidation (P) |
| 863.7578 | 2588.252 | 2588.251 | 46.41 | | GSDGSVGPVGPAGPIGSAGPPGFPGAPGPK | | 3 Oxidation (P) |
| 1295.136 | 2588.257 | 2588.251 | 54.43 | | GSDGSVGPVGPAGPIGSAGPPGFPGAPGPK | | Oxidation (K); 2 Oxidation (P) |
| 1303.132 | 2604.249 | 2604.246 | 40.32 | | GSDGSVGPVGPAGPIGSAGPPGFPGAPGPK | | 4 Oxidation (P) |
| 1303.132 | 2604.249 | 2604.246 | 56.71 | | GSDGSVGPVGPAGPIGSAGPPGFPGAPGPK | | Oxidation (K); 3 Oxidation (P) |
| 931.7734 | 2792.298 | 2792.3 | 46.96 | | GEQGPAGPPGFQGLPGPAGTAGEAGKPGER | | Deamidated (NQ); Oxidation (K); 2 Oxidation (P) |
| 931.7744 | 2792.301 | 2792.3 | 41.19 | | GEQGPAGPPGFQGLPGPAGTAGEAGKPGER | | Deamidated (NQ); Oxidation (K); 2 Oxidation (P) |
| 932.1026 | 2793.286 | 2793.284 | 42.96 | | GEQGPAGPPGFQGLPGPAGTAGEAGKPGER | | 2 Deamidated (NQ); Oxidation (K); 2 Oxidation (P) |
| 955.7969 | 2864.369 | 2864.369 | 64.93 | | GPKGENGPVGPTGPVGAAGPSGPNGPPGPAGSR | | 2 Deamidated (NQ); Oxidation (P) |
| 955.7975 | 2864.371 | 2864.369 | 70.55 | | GPKGENGPVGPTGPVGAAGPSGPNGPPGPAGSR | | 2 Deamidated (NQ); Oxidation (P) |
| 960.473 | 2878.397 | 2878.396 | 59.41 | | GPKGENGPVGPTGPVGAAGPSGPNGPPGPAGSR | | Oxidation (K); Oxidation (P) |
| 960.8004 | 2879.379 | 2879.38 | 64.26 | | GPKGENGPVGPTGPVGAAGPSGPNGPPGPAGSR | | Deamidated (NQ); Oxidation (K); Oxidation (P) |
| 960.8011 | 2879.382 | 2879.38 | 65.68 | | GPKGENGPVGPTGPVGAAGPSGPNGPPGPAGSR | | Deamidated (NQ); Oxidation (K); Oxidation (P) |
| 961.1284 | 2880.363 | 2880.364 | 66.81 | | GPKGENGPVGPTGPVGAAGPSGPNGPPGPAGSR | | 2 Deamidated (NQ); Oxidation (K); Oxidation (P) |
| 961.129 | 2880.365 | 2880.364 | 72.44 | | GPKGENGPVGPTGPVGAAGPSGPNGPPGPAGSR | | 2 Deamidated (NQ); Oxidation (K); Oxidation (P) |
| 1125.891 | 3374.651 | 3374.649 | 52.55 | | GPSGEPGTAGPPGTPGPQGLLGAPGFLGLPGSRGER | | 5 Oxidation (P) |

| Collagen alpha-1(I) chain OS=Bos taurus GN=COL1A1 PE=1 SV=3 | 7580 |
| --- | --- |

| 392.2217 | 782.4288 | 782.4286 | 42.32 | GAAGLPGPK | Oxidation (P) |
| --- | --- | --- | --- | --- | --- |
| 426.2164 | 850.4182 | 850.4185 | 41.43 | GFSGLDGAK |  |
| 426.2164 | 850.4182 | 850.4185 | 44.15 | GFSGLDGAK |  |
| 426.2164 | 850.4182 | 850.4185 | 45.83 | GFSGLDGAK |  |
| 449.7587 | 897.5028 | 897.5032 | 41.02 | GVVGLPGQR | Oxidation (P) |
| 449.7587 | 897.5028 | 897.5032 | 45.15 | GVVGLPGQR | Oxidation (P) |
| 449.7588 | 897.503 | 897.5032 | 52.21 | GVVGLPGQR | Oxidation (P) |
| 450.2505 | 898.4864 | 898.4872 | 43.23 | GVVGLPGQR | Deamidated (NQ); Oxidation (P) |
| 450.2506 | 898.4866 | 898.4872 | 44.6 | GVVGLPGQR | Deamidated (NQ); Oxidation (P) |
| 464.7403 | 927.466 | 927.4662 | 54.48 | PGEAGLPGAK | 2 Oxidation (P) |
| 464.7403 | 927.466 | 927.4662 | 44.57 | PGEAGLPGAK | 2 Oxidation (P) |
| 544.772 | 1087.529 | 1087.53 | 54.17 | GFPGADGVAGPK | Oxidation (P) |
| 544.772 | 1087.529 | 1087.53 | 56.29 | GFPGADGVAGPK | Oxidation (P) |
| 544.7721 | 1087.53 | 1087.53 | 51.33 | GFPGADGVAGPK | Oxidation (P) |
| 544.7721 | 1087.53 | 1087.53 | 48.8 | GFPGADGVAGPK | Oxidation (P) |
| 544.7722 | 1087.53 | 1087.53 | 45.84 | GFPGADGVAGPK | Oxidation (P) |
| 553.2912 | 1104.568 | 1104.568 | 43.52 | GVQGPPGPAGPR | Oxidation (P) |
| 553.2914 | 1104.568 | 1104.568 | 45.25 | GVQGPPGPAGPR | Oxidation (P) |
| 553.2914 | 1104.568 | 1104.568 | 47.44 | GVQGPPGPAGPR | Oxidation (P) |
| 553.7831 | 1105.552 | 1105.552 | 45.08 | GVQGPPGPAGPR | Deamidated (NQ); Oxidation (P) |
| 553.7832 | 1105.552 | 1105.552 | 53.68 | GVQGPPGPAGPR | Deamidated (NQ); Oxidation (P) |
| 581.7816 | 1161.549 | 1161.549 | 44.95 | GQAGVMGFPGPK | Deamidated (NQ); Oxidation (K) |
| 581.7818 | 1161.549 | 1161.549 | 47.92 | GQAGVMGFPGPK | Deamidated (NQ); Oxidation (P) |
| 588.8221 | 1175.63 | 1175.63 | 43.9 | GVPGPPGAVGPAGK | Oxidation (P) |
| 588.8223 | 1175.63 | 1175.63 | 41.32 | GVPGPPGAVGPAGK | Oxidation (P) |
| 589.2869 | 1176.559 | 1176.56 | 59.08 | GQAGVMGFPGPK | Oxidation (M); Oxidation (P) |
| 589.2869 | 1176.559 | 1176.56 | 54.1 | GQAGVMGFPGPK | Oxidation (M); Oxidation (P) |
| 589.2869 | 1176.559 | 1176.56 | 57.19 | GQAGVMGFPGPK | Oxidation (M); Oxidation (P) |
| 589.2871 | 1176.56 | 1176.56 | 56.89 | GQAGVMGFPGPK | Oxidation (M); Oxidation (P) |
| 589.2872 | 1176.56 | 1176.56 | 42.62 | GQAGVMGFPGPK | Oxidation (M); Oxidation (P) |
| 589.7791 | 1177.544 | 1177.544 | 66.2 | GQAGVMGFPGPK | Deamidated (NQ); Oxidation (M); Oxidation (P) |
| 589.7791 | 1177.544 | 1177.544 | 51.45 | GQAGVMGFPGPK | Deamidated (NQ); Oxidation (M); Oxidation (P) |
| 589.7792 | 1177.544 | 1177.544 | 54.19 | GQAGVMGFPGPK | Deamidated (NQ); Oxidation (M); Oxidation (P) |
| 589.7794 | 1177.544 | 1177.544 | 62.01 | GQAGVMGFPGPK | Deamidated (NQ); Oxidation (M); Oxidation (P) |
| 596.8197 | 1191.625 | 1191.625 | 44.93 | GVPGPPGAVGPAGK | 2 Oxidation (P) |
| 597.2847 | 1192.555 | 1192.555 | 49.02 | GQAGVMGFPGPK | Oxidation (K); Oxidation (M); Oxidation (P) |
| 597.7762 | 1193.538 | 1193.539 | 42.63 | GQAGVMGFPGPK | Deamidated (NQ); Oxidation (K); Oxidation (M); Oxidation (P) |
| 597.7764 | 1193.538 | 1193.539 | 57.47 | GQAGVMGFPGPK | Deamidated (NQ); Oxidation (K); Oxidation (M); Oxidation (P) |
| 597.7764 | 1193.538 | 1193.539 | 53.23 | GQAGVMGFPGPK | Deamidated (NQ); Oxidation (K); Oxidation (M); Oxidation (P) |
| 597.7766 | 1193.539 | 1193.539 | 67.88 | GQAGVMGFPGPK | Deamidated (NQ); Oxidation (K); Oxidation (M); Oxidation (P) |
| 621.3336 | 1240.653 | 1240.652 | 44.13 | GVVGLPGQRGER | Deamidated (NQ); Oxidation (P) |
| 621.8018 | 1241.589 | 1241.589 | 50.72 | GLTGSPGSPGPDGK | Oxidation (P) |
| 621.8018 | 1241.589 | 1241.589 | 44.63 | GLTGSPGSPGPDGK | Oxidation (P) |
| 621.802 | 1241.589 | 1241.589 | 42.77 | GLTGSPGSPGPDGK | Oxidation (P) |
| 629.7993 | 1257.584 | 1257.584 | 45.77 | GLTGSPGSPGPDGK | 2 Oxidation (P) |
| 629.7993 | 1257.584 | 1257.584 | 45.51 | GLTGSPGSPGPDGK | 2 Oxidation (P) |
| 656.8302 | 1311.646 | 1311.646 | 54.18 | GFPGLPGPSGEPGK | Oxidation (P) |
| 656.8304 | 1311.646 | 1311.646 | 48.35 | GFPGLPGPSGEPGK | Oxidation (P) |
| 656.8306 | 1311.647 | 1311.646 | 47.47 | GFPGLPGPSGEPGK | Oxidation (P) |
| 656.8309 | 1311.647 | 1311.646 | 46.02 | GFPGLPGPSGEPGK | Oxidation (P) |
| 664.8273 | 1327.64 | 1327.641 | 60.87 | GFPGLPGPSGEPGK | 2 Oxidation (P) |
| 664.8273 | 1327.64 | 1327.641 | 43.71 | GFPGLPGPSGEPGK | 2 Oxidation (P) |
| 664.8275 | 1327.64 | 1327.641 | 71.05 | GFPGLPGPSGEPGK | 2 Oxidation (P) |
| 664.8276 | 1327.641 | 1327.641 | 55.63 | GFPGLPGPSGEPGK | 2 Oxidation (P) |
| 664.8276 | 1327.641 | 1327.641 | 40.13 | GFPGLPGPSGEPGK | 2 Oxidation (P) |
| 664.8276 | 1327.641 | 1327.641 | 43.94 | GFPGLPGPSGEPGK | 2 Oxidation (P) |
| 664.8277 | 1327.641 | 1327.641 | 49.46 | GFPGLPGPSGEPGK | 2 Oxidation (P) |
| 664.8278 | 1327.641 | 1327.641 | 51.32 | GFPGLPGPSGEPGK | 2 Oxidation (P) |
| 664.8278 | 1327.641 | 1327.641 | 41.98 | GFPGLPGPSGEPGK | 2 Oxidation (P) |
| 664.8278 | 1327.641 | 1327.641 | 51.92 | GFPGLPGPSGEPGK | 2 Oxidation (P) |
| 664.8278 | 1327.641 | 1327.641 | 41.59 | GFPGLPGPSGEPGK | 2 Oxidation (P) |
| 664.8278 | 1327.641 | 1327.641 | 55.57 | GFPGLPGPSGEPGK | 2 Oxidation (P) |
| 666.8302 | 1331.646 | 1331.647 | 51.95 | GPSGPQGPSGPPGPK | Oxidation (P) |
| 666.8313 | 1331.648 | 1331.647 | 42.93 | GPSGPQGPSGPPGPK | Oxidation (P) |
| 667.3234 | 1332.632 | 1332.631 | 72.36 | GPSGPQGPSGPPGPK | Deamidated (NQ); Oxidation (P) |
| 667.3237 | 1332.633 | 1332.631 | 79.09 | GPSGPQGPSGPPGPK | Deamidated (NQ); Oxidation (P) |
| 667.3237 | 1332.633 | 1332.631 | 57.53 | GPSGPQGPSGPPGPK | Deamidated (NQ); Oxidation (P) |
| 667.3238 | 1332.633 | 1332.631 | 64.42 | GPSGPQGPSGPPGPK | Deamidated (NQ); Oxidation (P) |
| 672.8249 | 1343.635 | 1343.636 | 43.14 | GFPGLPGPSGEPGK | 3 Oxidation (P) |
| 672.8251 | 1343.636 | 1343.636 | 62.67 | GFPGLPGPSGEPGK | 3 Oxidation (P) |
| 672.8251 | 1343.636 | 1343.636 | 50.04 | GFPGLPGPSGEPGK | 3 Oxidation (P) |
| 672.8251 | 1343.636 | 1343.636 | 41.2 | GFPGLPGPSGEPGK | 3 Oxidation (P) |
| 672.8252 | 1343.636 | 1343.636 | 69.88 | GFPGLPGPSGEPGK | 3 Oxidation (P) |
| 672.8252 | 1343.636 | 1343.636 | 47.84 | GFPGLPGPSGEPGK | 3 Oxidation (P) |
| 672.8253 | 1343.636 | 1343.636 | 40.59 | GFPGLPGPSGEPGK | 3 Oxidation (P) |
| 672.8253 | 1343.636 | 1343.636 | 40.57 | GFPGLPGPSGEPGK | 3 Oxidation (P) |
| 672.8254 | 1343.636 | 1343.636 | 54.39 | GFPGLPGPSGEPGK | 3 Oxidation (P) |
| 718.3447 | 1434.675 | 1434.674 | 74.15 | GEPGPAGLPGPPGER | 3 Oxidation (P) |
| 718.3447 | 1434.675 | 1434.674 | 65.27 | GEPGPAGLPGPPGER | 3 Oxidation (P) |
| 718.3447 | 1434.675 | 1434.674 | 42.13 | GEPGPAGLPGPPGER | 3 Oxidation (P) |
| 718.3448 | 1434.675 | 1434.674 | 53.24 | GEPGPAGLPGPPGER | 3 Oxidation (P) |
| 718.3448 | 1434.675 | 1434.674 | 61.6 | GEPGPAGLPGPPGER | 3 Oxidation (P) |
| 730.3475 | 1458.68 | 1458.685 | 79.75 | GSAGPPGATGFPGAAGR | 2 Oxidation (P) |
| 730.3493 | 1458.684 | 1458.685 | 69.26 | GSAGPPGATGFPGAAGR | 2 Oxidation (P) |
| 730.3494 | 1458.684 | 1458.685 | 62.34 | GSAGPPGATGFPGAAGR | 2 Oxidation (P) |
| 730.3495 | 1458.684 | 1458.685 | 41.35 | GSAGPPGATGFPGAAGR | 2 Oxidation (P) |
| 730.3496 | 1458.685 | 1458.685 | 89.38 | GSAGPPGATGFPGAAGR | 2 Oxidation (P) |
| 730.3496 | 1458.685 | 1458.685 | 76.52 | GSAGPPGATGFPGAAGR | 2 Oxidation (P) |
| 730.3497 | 1458.685 | 1458.685 | 86.66 | GSAGPPGATGFPGAAGR | 2 Oxidation (P) |
| 730.3497 | 1458.685 | 1458.685 | 53.4 | GSAGPPGATGFPGAAGR | 2 Oxidation (P) |
| 730.3498 | 1458.685 | 1458.685 | 49.44 | GSAGPPGATGFPGAAGR | 2 Oxidation (P) |
| 730.3499 | 1458.685 | 1458.685 | 78.88 | GSAGPPGATGFPGAAGR | 2 Oxidation (P) |
| 730.3499 | 1458.685 | 1458.685 | 93.65 | GSAGPPGATGFPGAAGR | 2 Oxidation (P) |
| 730.3499 | 1458.685 | 1458.685 | 50.41 | GSAGPPGATGFPGAAGR | 2 Oxidation (P) |
| 730.3499 | 1458.685 | 1458.685 | 42.8 | GSAGPPGATGFPGAAGR | 2 Oxidation (P) |
| 730.35 | 1458.685 | 1458.685 | 90.88 | GSAGPPGATGFPGAAGR | 2 Oxidation (P) |
| 730.35 | 1458.685 | 1458.685 | 104.52 | GSAGPPGATGFPGAAGR | 2 Oxidation (P) |
| 730.35 | 1458.685 | 1458.685 | 66.3 | GSAGPPGATGFPGAAGR | 2 Oxidation (P) |
| 730.35 | 1458.685 | 1458.685 | 74.82 | GSAGPPGATGFPGAAGR | 2 Oxidation (P) |
| 730.3501 | 1458.686 | 1458.685 | 68.23 | GSAGPPGATGFPGAAGR | 2 Oxidation (P) |
| 730.3501 | 1458.686 | 1458.685 | 77.14 | GSAGPPGATGFPGAAGR | 2 Oxidation (P) |
| 730.3502 | 1458.686 | 1458.685 | 56.13 | GSAGPPGATGFPGAAGR | 2 Oxidation (P) |
| 730.3503 | 1458.686 | 1458.685 | 73.26 | GSAGPPGATGFPGAAGR | 2 Oxidation (P) |
| 730.3503 | 1458.686 | 1458.685 | 76.38 | GSAGPPGATGFPGAAGR | 2 Oxidation (P) |
| 730.3503 | 1458.686 | 1458.685 | 67.01 | GSAGPPGATGFPGAAGR | 2 Oxidation (P) |
| 730.3503 | 1458.686 | 1458.685 | 54.61 | GSAGPPGATGFPGAAGR | 2 Oxidation (P) |
| 730.3503 | 1458.686 | 1458.685 | 52.86 | GSAGPPGATGFPGAAGR | 2 Oxidation (P) |
| 730.3504 | 1458.686 | 1458.685 | 49.27 | GSAGPPGATGFPGAAGR | 2 Oxidation (P) |
| 730.3506 | 1458.687 | 1458.685 | 70.88 | GSAGPPGATGFPGAAGR | 2 Oxidation (P) |
| 730.3506 | 1458.687 | 1458.685 | 43.58 | GSAGPPGATGFPGAAGR | 2 Oxidation (P) |
| 730.3506 | 1458.687 | 1458.685 | 53.97 | GSAGPPGATGFPGAAGR | 2 Oxidation (P) |
| 730.351 | 1458.687 | 1458.685 | 53.37 | GSAGPPGATGFPGAAGR | 2 Oxidation (P) |
| 730.3512 | 1458.688 | 1458.685 | 40.63 | GSAGPPGATGFPGAAGR | 2 Oxidation (P) |
| 730.3513 | 1458.688 | 1458.685 | 56.06 | GSAGPPGATGFPGAAGR | 2 Oxidation (P) |
| 745.86 | 1489.705 | 1489.705 | 49.06 | PGEVGPPGPPGPAGEK | 3 Oxidation (P) |
| 745.86 | 1489.705 | 1489.705 | 45.36 | PGEVGPPGPPGPAGEK | 3 Oxidation (P) |
| 773.8969 | 1545.779 | 1545.779 | 43.39 | DGLNGLPGPIGPPGPR | Deamidated (NQ); 2 Oxidation (P) |
| 520.9424 | 1559.805 | 1559.806 | 54.36 | GETGPAGPAGPIGPVGAR |  |
| 520.9424 | 1559.805 | 1559.806 | 63.23 | GETGPAGPAGPIGPVGAR |  |
| 780.9102 | 1559.806 | 1559.806 | 70.15 | GETGPAGPAGPIGPVGAR |  |
| 780.9104 | 1559.806 | 1559.806 | 53.47 | GETGPAGPAGPIGPVGAR |  |
| 781.4002 | 1560.786 | 1560.79 | 41.63 | DGLNGLPGPIGPPGPR | 3 Oxidation (P) |
| 521.2701 | 1560.789 | 1560.79 | 45.36 | DGLNGLPGPIGPPGPR | 3 Oxidation (P) |
| 521.2702 | 1560.789 | 1560.79 | 54.25 | DGLNGLPGPIGPPGPR | 3 Oxidation (P) |
| 781.8932 | 1561.772 | 1561.774 | 54.18 | DGLNGLPGPIGPPGPR | Deamidated (NQ); 3 Oxidation (P) |
| 781.8934 | 1561.772 | 1561.774 | 46.3 | DGLNGLPGPIGPPGPR | Deamidated (NQ); 3 Oxidation (P) |
| 781.8941 | 1561.774 | 1561.774 | 56.11 | DGLNGLPGPIGPPGPR | Deamidated (NQ); 3 Oxidation (P) |
| 781.8941 | 1561.774 | 1561.774 | 46.01 | DGLNGLPGPIGPPGPR | Deamidated (NQ); 3 Oxidation (P) |
| 781.8944 | 1561.774 | 1561.774 | 58.01 | DGLNGLPGPIGPPGPR | Deamidated (NQ); 3 Oxidation (P) |
| 781.8946 | 1561.775 | 1561.774 | 45.48 | DGLNGLPGPIGPPGPR | Deamidated (NQ); 3 Oxidation (P) |
| 781.8947 | 1561.775 | 1561.774 | 57.98 | DGLNGLPGPIGPPGPR | Deamidated (NQ); 3 Oxidation (P) |
| 781.8947 | 1561.775 | 1561.774 | 54.39 | DGLNGLPGPIGPPGPR | Deamidated (NQ); 3 Oxidation (P) |
| 781.8947 | 1561.775 | 1561.774 | 52.06 | DGLNGLPGPIGPPGPR | Deamidated (NQ); 3 Oxidation (P) |
| 781.895 | 1561.775 | 1561.774 | 50.09 | DGLNGLPGPIGPPGPR | Deamidated (NQ); 3 Oxidation (P) |
| 781.8951 | 1561.776 | 1561.774 | 41.56 | DGLNGLPGPIGPPGPR | Deamidated (NQ); 3 Oxidation (P) |
| 787.9126 | 1573.811 | 1573.81 | 46.34 | GLTGPIGPPGPAGAPGDK | Oxidation (P) |
| 789.8914 | 1577.768 | 1577.769 | 53.03 | DGLNGLPGPIGPPGPR | Deamidated (NQ); 4 Oxidation (P) |
| 793.3896 | 1584.765 | 1584.765 | 65.56 | GANGAPGIAGAPGFPGAR | 3 Oxidation (P) |
| 793.3896 | 1584.765 | 1584.765 | 55.35 | GANGAPGIAGAPGFPGAR | 3 Oxidation (P) |
| 793.39 | 1584.765 | 1584.765 | 48.7 | GANGAPGIAGAPGFPGAR | 3 Oxidation (P) |
| 793.8805 | 1585.746 | 1585.749 | 51.79 | GANGAPGIAGAPGFPGAR | Deamidated (NQ); 3 Oxidation (P) |
| 793.8805 | 1585.746 | 1585.749 | 46.81 | GANGAPGIAGAPGFPGAR | Deamidated (NQ); 3 Oxidation (P) |
| 793.8806 | 1585.747 | 1585.749 | 41.6 | GANGAPGIAGAPGFPGAR | Deamidated (NQ); 3 Oxidation (P) |
| 793.8812 | 1585.748 | 1585.749 | 56.23 | GANGAPGIAGAPGFPGAR | Deamidated (NQ); 3 Oxidation (P) |
| 793.8813 | 1585.748 | 1585.749 | 61.33 | GANGAPGIAGAPGFPGAR | Deamidated (NQ); 3 Oxidation (P) |
| 793.8815 | 1585.748 | 1585.749 | 48.44 | GANGAPGIAGAPGFPGAR | Deamidated (NQ); 3 Oxidation (P) |
| 793.8817 | 1585.749 | 1585.749 | 58.23 | GANGAPGIAGAPGFPGAR | Deamidated (NQ); 3 Oxidation (P) |
| 793.8821 | 1585.75 | 1585.749 | 43.33 | GANGAPGIAGAPGFPGAR | Deamidated (NQ); 3 Oxidation (P) |
| 539.2659 | 1614.776 | 1614.775 | 40.73 | GGPGSRGFPGADGVAGPK | 2 Oxidation (P) |
| 808.3953 | 1614.776 | 1614.775 | 42.58 | GGPGSRGFPGADGVAGPK | 2 Oxidation (P) |
| 539.9335 | 1616.779 | 1616.78 | 41.33 | GFSGLDGAKGDAGPAGPK | Oxidation (K) |
| 539.9337 | 1616.779 | 1616.78 | 48.08 | GFSGLDGAKGDAGPAGPK | Oxidation (K) |
| 809.3981 | 1616.782 | 1616.78 | 102.81 | GFSGLDGAKGDAGPAGPK | Oxidation (K) |
| 809.3989 | 1616.783 | 1616.78 | 66.12 | GFSGLDGAKGDAGPAGPK | Oxidation (K) |
| 845.8925 | 1689.77 | 1689.771 | 91.83 | DGEAGAQGPPGPAGPAGER |  |
| 845.893 | 1689.771 | 1689.771 | 77.94 | DGEAGAQGPPGPAGPAGER |  |
| 846.3843 | 1690.754 | 1690.755 | 45.03 | DGEAGAQGPPGPAGPAGER | Deamidated (NQ) |
| 853.8906 | 1705.767 | 1705.766 | 66.49 | DGEAGAQGPPGPAGPAGER | Oxidation (P) |
| 853.8907 | 1705.767 | 1705.766 | 77.39 | DGEAGAQGPPGPAGPAGER | Oxidation (P) |
| 853.8912 | 1705.768 | 1705.766 | 100.52 | DGEAGAQGPPGPAGPAGER | Oxidation (P) |
| 872.4305 | 1742.846 | 1742.845 | 59.25 | GARGSAGPPGATGFPGAAGR | 2 Oxidation (P) |
| 872.4305 | 1742.846 | 1742.845 | 61.65 | GARGSAGPPGATGFPGAAGR | 2 Oxidation (P) |
| 907.4395 | 1812.864 | 1812.864 | 47.85 | VGPPGPSGNAGPPGPPGPAGK | Deamidated (NQ); Oxidation (K); 2 Oxidation (P) |
| 907.4401 | 1812.866 | 1812.864 | 45.85 | VGPPGPSGNAGPPGPPGPAGK | Deamidated (NQ); Oxidation (K); 2 Oxidation (P) |
| 907.4403 | 1812.866 | 1812.864 | 41.37 | VGPPGPSGNAGPPGPPGPAGK | Deamidated (NQ); Oxidation (K); 2 Oxidation (P) |
| 908.9357 | 1815.857 | 1815.857 | 71.23 | GPPGPMGPPGLAGPPGESGR | 2 Oxidation (P) |
| 908.9359 | 1815.857 | 1815.857 | 99.84 | GPPGPMGPPGLAGPPGESGR | Oxidation (M); Oxidation (P) |
| 908.9361 | 1815.858 | 1815.857 | 50.14 | GPPGPMGPPGLAGPPGESGR | Oxidation (M); Oxidation (P) |
| 908.9362 | 1815.858 | 1815.857 | 51.53 | GPPGPMGPPGLAGPPGESGR | 2 Oxidation (P) |
| 908.9362 | 1815.858 | 1815.857 | 73.03 | GPPGPMGPPGLAGPPGESGR | Oxidation (M); Oxidation (P) |
| 908.9363 | 1815.858 | 1815.857 | 45.24 | GPPGPMGPPGLAGPPGESGR | Oxidation (M); Oxidation (P) |
| 908.9367 | 1815.859 | 1815.857 | 62.9 | GPPGPMGPPGLAGPPGESGR | Oxidation (M); Oxidation (P) |
| 908.937 | 1815.859 | 1815.857 | 59.18 | GPPGPMGPPGLAGPPGESGR | Oxidation (M); Oxidation (P) |
| 915.437 | 1828.859 | 1828.859 | 47.07 | VGPPGPSGNAGPPGPPGPAGK | Deamidated (NQ); 4 Oxidation (P) |
| 611.6244 | 1831.851 | 1831.852 | 41.47 | GPPGPMGPPGLAGPPGESGR | Oxidation (M); 2 Oxidation (P) |
| 916.9333 | 1831.852 | 1831.852 | 95.85 | GPPGPMGPPGLAGPPGESGR | Oxidation (M); 2 Oxidation (P) |
| 916.9333 | 1831.852 | 1831.852 | 40.55 | GPPGPMGPPGLAGPPGESGR | Oxidation (M); 2 Oxidation (P) |
| 916.9333 | 1831.852 | 1831.852 | 63.68 | GPPGPMGPPGLAGPPGESGR | Oxidation (M); 2 Oxidation (P) |
| 611.6247 | 1831.852 | 1831.852 | 47.41 | GPPGPMGPPGLAGPPGESGR | Oxidation (M); 2 Oxidation (P) |
| 916.9337 | 1831.853 | 1831.852 | 46.43 | GPPGPMGPPGLAGPPGESGR | Oxidation (M); 2 Oxidation (P) |
| 916.9338 | 1831.853 | 1831.852 | 96.31 | GPPGPMGPPGLAGPPGESGR | Oxidation (M); 2 Oxidation (P) |
| 916.9338 | 1831.853 | 1831.852 | 76.01 | GPPGPMGPPGLAGPPGESGR | Oxidation (M); 2 Oxidation (P) |
| 916.9339 | 1831.853 | 1831.852 | 63.6 | GPPGPMGPPGLAGPPGESGR | 3 Oxidation (P) |
| 916.9339 | 1831.853 | 1831.852 | 73.24 | GPPGPMGPPGLAGPPGESGR | Oxidation (M); 2 Oxidation (P) |
| 924.9309 | 1847.847 | 1847.847 | 44.49 | GPPGPMGPPGLAGPPGESGR | Oxidation (M); 3 Oxidation (P) |
| 924.931 | 1847.847 | 1847.847 | 43.14 | GPPGPMGPPGLAGPPGESGR | Oxidation (M); 3 Oxidation (P) |
| 924.9312 | 1847.848 | 1847.847 | 43.41 | GPPGPMGPPGLAGPPGESGR | Oxidation (M); 3 Oxidation (P) |
| 924.9314 | 1847.848 | 1847.847 | 50.23 | GPPGPMGPPGLAGPPGESGR | Oxidation (M); 3 Oxidation (P) |
| 932.4398 | 1862.865 | 1862.865 | 79.13 | GEPGPTGIQGPPGPAGEEGK | 2 Oxidation (P) |
| 932.4399 | 1862.865 | 1862.865 | 63.27 | GEPGPTGIQGPPGPAGEEGK | 2 Oxidation (P) |
| 932.9288 | 1863.843 | 1863.842 | 47.67 | GPPGPMGPPGLAGPPGESGR | 5 Oxidation (P) |
| 932.9295 | 1863.844 | 1863.842 | 47.23 | GPPGPMGPPGLAGPPGESGR | 5 Oxidation (P) |
| 932.9306 | 1863.847 | 1863.849 | 60.96 | GEPGPTGIQGPPGPAGEEGK | Deamidated (NQ); 2 Oxidation (P) |
| 932.9316 | 1863.849 | 1863.849 | 68.28 | GEPGPTGIQGPPGPAGEEGK | Deamidated (NQ); 2 Oxidation (P) |
| 659.3359 | 1974.986 | 1974.987 | 49.74 | SGDRGETGPAGPAGPIGPVGAR |  |
| 988.5005 | 1974.986 | 1974.987 | 60.05 | SGDRGETGPAGPAGPIGPVGAR |  |
| 988.5012 | 1974.988 | 1974.987 | 56.34 | SGDRGETGPAGPAGPIGPVGAR |  |
| 988.5013 | 1974.988 | 1974.987 | 48.24 | SGDRGETGPAGPAGPIGPVGAR |  |
| 1010.491 | 2018.967 | 2018.966 | 66.98 | GEPGPTGIQGPPGPAGEEGKR | 2 Oxidation (P) |
| 1010.491 | 2018.967 | 2018.966 | 64.09 | GEPGPTGIQGPPGPAGEEGKR | 2 Oxidation (P) |
| 1010.491 | 2018.967 | 2018.966 | 45.05 | GEPGPTGIQGPPGPAGEEGKR | 2 Oxidation (P) |
| 1010.492 | 2018.969 | 2018.966 | 60.41 | GEPGPTGIQGPPGPAGEEGKR | 2 Oxidation (P) |
| 1010.984 | 2019.953 | 2019.95 | 60.24 | GEPGPTGIQGPPGPAGEEGKR | Deamidated (NQ); 2 Oxidation (P) |
| 1010.985 | 2019.955 | 2019.95 | 70.74 | GEPGPTGIQGPPGPAGEEGKR | Deamidated (NQ); 2 Oxidation (P) |
| 691.6754 | 2072.004 | 2072.004 | 40.88 | GAPGADGPAGAPGTPGPQGIAGQR | Oxidation (P) |
| 691.6756 | 2072.005 | 2072.004 | 47.43 | GAPGADGPAGAPGTPGPQGIAGQR | Oxidation (P) |
| 1037.011 | 2072.007 | 2072.004 | 66.12 | GAPGADGPAGAPGTPGPQGIAGQR | Oxidation (P) |
| 1037.011 | 2072.007 | 2072.004 | 60.04 | GAPGADGPAGAPGTPGPQGIAGQR | Oxidation (P) |
| 1037.5 | 2072.985 | 2072.988 | 54.06 | GAPGADGPAGAPGTPGPQGIAGQR | Deamidated (NQ); Oxidation (P) |
| 1037.501 | 2072.987 | 2072.988 | 81.83 | GAPGADGPAGAPGTPGPQGIAGQR | Deamidated (NQ); Oxidation (P) |
| 1037.501 | 2072.987 | 2072.988 | 95.05 | GAPGADGPAGAPGTPGPQGIAGQR | Deamidated (NQ); Oxidation (P) |
| 1037.501 | 2072.987 | 2072.988 | 77.81 | GAPGADGPAGAPGTPGPQGIAGQR | Deamidated (NQ); Oxidation (P) |
| 692.0034 | 2072.988 | 2072.988 | 45.21 | GAPGADGPAGAPGTPGPQGIAGQR | Deamidated (NQ); Oxidation (P) |
| 1037.502 | 2072.989 | 2072.988 | 53.31 | GAPGADGPAGAPGTPGPQGIAGQR | Deamidated (NQ); Oxidation (P) |
| 1037.993 | 2073.971 | 2073.972 | 67.54 | GAPGADGPAGAPGTPGPQGIAGQR | 2 Deamidated (NQ); Oxidation (P) |
| 1037.993 | 2073.971 | 2073.972 | 78.67 | GAPGADGPAGAPGTPGPQGIAGQR | 2 Deamidated (NQ); Oxidation (P) |
| 692.3311 | 2073.972 | 2073.972 | 42.44 | GAPGADGPAGAPGTPGPQGIAGQR | 2 Deamidated (NQ); Oxidation (P) |
| 692.3312 | 2073.972 | 2073.972 | 44.21 | GAPGADGPAGAPGTPGPQGIAGQR | 2 Deamidated (NQ); Oxidation (P) |
| 1045.006 | 2087.997 | 2087.999 | 52.85 | GAPGADGPAGAPGTPGPQGIAGQR | 2 Oxidation (P) |
| 1045.007 | 2087.999 | 2087.999 | 82.18 | GAPGADGPAGAPGTPGPQGIAGQR | 2 Oxidation (P) |
| 1045.007 | 2087.999 | 2087.999 | 50.55 | GAPGADGPAGAPGTPGPQGIAGQR | 2 Oxidation (P) |
| 1045.008 | 2088.001 | 2087.999 | 44.88 | GAPGADGPAGAPGTPGPQGIAGQR | 2 Oxidation (P) |
| 1045.498 | 2088.981 | 2088.983 | 73.8 | GAPGADGPAGAPGTPGPQGIAGQR | Deamidated (NQ); 2 Oxidation (P) |
| 697.335 | 2088.983 | 2088.983 | 41.1 | GAPGADGPAGAPGTPGPQGIAGQR | Deamidated (NQ); 2 Oxidation (P) |
| 1045.499 | 2088.983 | 2088.983 | 63.28 | GAPGADGPAGAPGTPGPQGIAGQR | Deamidated (NQ); 2 Oxidation (P) |
| 1045.499 | 2088.983 | 2088.983 | 60.44 | GAPGADGPAGAPGTPGPQGIAGQR | Deamidated (NQ); 2 Oxidation (P) |
| 1045.499 | 2088.983 | 2088.983 | 56.49 | GAPGADGPAGAPGTPGPQGIAGQR | Deamidated (NQ); 2 Oxidation (P) |
| 1045.99 | 2089.965 | 2089.967 | 84.31 | GAPGADGPAGAPGTPGPQGIAGQR | 2 Deamidated (NQ); 2 Oxidation (P) |
| 1045.99 | 2089.965 | 2089.967 | 62.84 | GAPGADGPAGAPGTPGPQGIAGQR | 2 Deamidated (NQ); 2 Oxidation (P) |
| 697.663 | 2089.967 | 2089.967 | 51.98 | GAPGADGPAGAPGTPGPQGIAGQR | 2 Deamidated (NQ); 2 Oxidation (P) |
| 1045.991 | 2089.967 | 2089.967 | 75.22 | GAPGADGPAGAPGTPGPQGIAGQR | 2 Deamidated (NQ); 2 Oxidation (P) |
| 1045.991 | 2089.967 | 2089.967 | 69.76 | GAPGADGPAGAPGTPGPQGIAGQR | 2 Deamidated (NQ); 2 Oxidation (P) |
| 702.3387 | 2103.994 | 2103.993 | 42.5 | GAPGADGPAGAPGTPGPQGIAGQR | 3 Oxidation (P) |
| 1053.005 | 2103.995 | 2103.993 | 69.24 | GAPGADGPAGAPGTPGPQGIAGQR | 3 Oxidation (P) |
| 1053.005 | 2103.995 | 2103.993 | 74.92 | GAPGADGPAGAPGTPGPQGIAGQR | 3 Oxidation (P) |
| 1053.988 | 2105.961 | 2105.961 | 82.82 | GAPGADGPAGAPGTPGPQGIAGQR | 2 Deamidated (NQ); 3 Oxidation (P) |
| 1053.988 | 2105.961 | 2105.961 | 70.43 | GAPGADGPAGAPGTPGPQGIAGQR | 2 Deamidated (NQ); 3 Oxidation (P) |
| 1069.039 | 2136.063 | 2136.06 | 50.95 | GETGPAGPPGAPGAPGAPGPVGPAGK | Oxidation (P) |
| 717.6593 | 2149.956 | 2149.955 | 44.98 | GEPGPPGPAGFAGPPGADGQPGAK | Deamidated (NQ); 4 Oxidation (P) |
| 1077.034 | 2152.053 | 2152.055 | 58.96 | GETGPAGPPGAPGAPGAPGPVGPAGK | 2 Oxidation (P) |
| 1077.035 | 2152.055 | 2152.055 | 55.89 | GETGPAGPPGAPGAPGAPGPVGPAGK | 2 Oxidation (P) |
| 1085.033 | 2168.051 | 2168.05 | 55.75 | GETGPAGPPGAPGAPGAPGPVGPAGK | 3 Oxidation (P) |
| 1085.033 | 2168.051 | 2168.05 | 42.97 | GETGPAGPPGAPGAPGAPGPVGPAGK | 3 Oxidation (P) |
| 1093.03 | 2184.045 | 2184.045 | 48.47 | GETGPAGPPGAPGAPGAPGPVGPAGK | 4 Oxidation (P) |
| 729.0225 | 2184.046 | 2184.045 | 48.09 | GETGPAGPPGAPGAPGAPGPVGPAGK | 4 Oxidation (P) |
| 729.0226 | 2184.046 | 2184.045 | 47.65 | GETGPAGPPGAPGAPGAPGPVGPAGK | 4 Oxidation (P) |
| 729.0226 | 2184.046 | 2184.045 | 45.78 | GETGPAGPPGAPGAPGAPGPVGPAGK | 4 Oxidation (P) |
| 1093.031 | 2184.047 | 2184.045 | 41.85 | GETGPAGPPGAPGAPGAPGPVGPAGK | 4 Oxidation (P) |
| 1107.989 | 2213.963 | 2213.961 | 50.46 | GDAGAPGAPGSQGAPGLQGMPGER | Oxidation (M); 4 Oxidation (P) |
| 1108.48 | 2214.945 | 2214.945 | 57.58 | GDAGAPGAPGSQGAPGLQGMPGER | Deamidated (NQ); Oxidation (M); 4 Oxidation (P) |
| 1108.481 | 2214.947 | 2214.945 | 46.76 | GDAGAPGAPGSQGAPGLQGMPGER | Deamidated (NQ); Oxidation (M); 4 Oxidation (P) |
| 739.3578 | 2215.052 | 2215.051 | 44.23 | GETGPAGRPGEVGPPGPPGPAGEK | Oxidation (K); 2 Oxidation (P) |
| 1108.971 | 2215.927 | 2215.929 | 66.28 | GDAGAPGAPGSQGAPGLQGMPGER | 2 Deamidated (NQ); Oxidation (M); 4 Oxidation (P) |
| 1108.973 | 2215.931 | 2215.929 | 99.03 | GDAGAPGAPGSQGAPGLQGMPGER | 2 Deamidated (NQ); Oxidation (M); 4 Oxidation (P) |
| 1128.53 | 2255.045 | 2255.046 | 43.92 | GFPGLPGPSGEPGKQGPSGASGER | Deamidated (NQ); 2 Oxidation (P) |
| 1128.531 | 2255.047 | 2255.046 | 51.68 | GFPGLPGPSGEPGKQGPSGASGER | Deamidated (NQ); 2 Oxidation (P) |
| 1136.529 | 2271.043 | 2271.04 | 48.28 | GFPGLPGPSGEPGKQGPSGASGER | Deamidated (NQ); 3 Oxidation (P) |
| 1136.529 | 2271.043 | 2271.04 | 47.25 | GFPGLPGPSGEPGKQGPSGASGER | Deamidated (NQ); Oxidation (K); 2 Oxidation (P) |
| 759.0541 | 2274.141 | 2274.139 | 49.83 | GDAGPPGPAGPAGPPGPIGNVGAPGPK | Oxidation (P) |
| 1146.074 | 2290.133 | 2290.134 | 58.65 | GDAGPPGPAGPAGPPGPIGNVGAPGPK | Oxidation (K); Oxidation (P) |
| 1146.074 | 2290.133 | 2290.134 | 51.32 | GDAGPPGPAGPAGPPGPIGNVGAPGPK | Oxidation (K); Oxidation (P) |
| 1146.566 | 2291.117 | 2291.118 | 87.32 | GDAGPPGPAGPAGPPGPIGNVGAPGPK | Deamidated (NQ); Oxidation (K); Oxidation (P) |
| 1146.566 | 2291.117 | 2291.118 | 44.09 | GDAGPPGPAGPAGPPGPIGNVGAPGPK | Deamidated (NQ); Oxidation (K); Oxidation (P) |
| 764.7134 | 2291.118 | 2291.118 | 44.04 | GDAGPPGPAGPAGPPGPIGNVGAPGPK | Deamidated (NQ); Oxidation (K); Oxidation (P) |
| 764.7135 | 2291.119 | 2291.118 | 62.36 | GDAGPPGPAGPAGPPGPIGNVGAPGPK | Deamidated (NQ); Oxidation (K); Oxidation (P) |
| 764.7136 | 2291.119 | 2291.118 | 41.73 | GDAGPPGPAGPAGPPGPIGNVGAPGPK | Deamidated (NQ); Oxidation (K); Oxidation (P) |
| 764.7137 | 2291.119 | 2291.118 | 46.2 | GDAGPPGPAGPAGPPGPIGNVGAPGPK | Deamidated (NQ); Oxidation (K); Oxidation (P) |
| 1146.567 | 2291.119 | 2291.118 | 45.62 | GDAGPPGPAGPAGPPGPIGNVGAPGPK | Deamidated (NQ); 2 Oxidation (P) |
| 1151.515 | 2301.015 | 2301.015 | 50.4 | GEPGPPGPAGAAGPAGNPGADGQPGAK | 2 Deamidated (NQ); Oxidation (K); 2 Oxidation (P) |
| 1154.072 | 2306.129 | 2306.129 | 43.03 | GDAGPPGPAGPAGPPGPIGNVGAPGPK | Oxidation (K); 2 Oxidation (P) |
| 1154.073 | 2306.131 | 2306.129 | 50.85 | GDAGPPGPAGPAGPPGPIGNVGAPGPK | Oxidation (K); 2 Oxidation (P) |
| 1154.564 | 2307.113 | 2307.113 | 86.11 | GDAGPPGPAGPAGPPGPIGNVGAPGPK | Deamidated (NQ); Oxidation (K); 2 Oxidation (P) |
| 1154.564 | 2307.113 | 2307.113 | 63.17 | GDAGPPGPAGPAGPPGPIGNVGAPGPK | Deamidated (NQ); Oxidation (K); 2 Oxidation (P) |
| 770.0455 | 2307.115 | 2307.113 | 55.26 | GDAGPPGPAGPAGPPGPIGNVGAPGPK | Deamidated (NQ); Oxidation (K); 2 Oxidation (P) |
| 772.6882 | 2315.043 | 2315.042 | 42.43 | GEPGPPGPAGAAGPAGNPGADGQPGAK | 4 Oxidation (P) |
| 772.6888 | 2315.045 | 2315.042 | 40.79 | GEPGPPGPAGAAGPAGNPGADGQPGAK | Oxidation (K); 3 Oxidation (P) |
| 773.016 | 2316.026 | 2316.026 | 52.94 | GEPGPPGPAGAAGPAGNPGADGQPGAK | Deamidated (NQ); 4 Oxidation (P) |
| 1159.021 | 2316.027 | 2316.026 | 62.01 | GEPGPPGPAGAAGPAGNPGADGQPGAK | Deamidated (NQ); 4 Oxidation (P) |
| 1159.021 | 2316.027 | 2316.026 | 68.71 | GEPGPPGPAGAAGPAGNPGADGQPGAK | Deamidated (NQ); 4 Oxidation (P) |
| 1159.512 | 2317.009 | 2317.01 | 63.97 | GEPGPPGPAGAAGPAGNPGADGQPGAK | 2 Deamidated (NQ); 4 Oxidation (P) |
| 773.3439 | 2317.01 | 2317.01 | 55.6 | GEPGPPGPAGAAGPAGNPGADGQPGAK | 2 Deamidated (NQ); 4 Oxidation (P) |
| 773.3439 | 2317.01 | 2317.01 | 58.97 | GEPGPPGPAGAAGPAGNPGADGQPGAK | 2 Deamidated (NQ); 4 Oxidation (P) |
| 1159.513 | 2317.011 | 2317.01 | 62.91 | GEPGPPGPAGAAGPAGNPGADGQPGAK | 2 Deamidated (NQ); 4 Oxidation (P) |
| 1162.07 | 2322.125 | 2322.124 | 61.56 | GDAGPPGPAGPAGPPGPIGNVGAPGPK | 4 Oxidation (P) |
| 1162.562 | 2323.109 | 2323.108 | 69.07 | GDAGPPGPAGPAGPPGPIGNVGAPGPK | Deamidated (NQ); 4 Oxidation (P) |
| 1162.562 | 2323.109 | 2323.108 | 45.95 | GDAGPPGPAGPAGPPGPIGNVGAPGPK | Deamidated (NQ); 4 Oxidation (P) |
| 775.3773 | 2323.11 | 2323.108 | 51 | GDAGPPGPAGPAGPPGPIGNVGAPGPK | Deamidated (NQ); 4 Oxidation (P) |
| 819.0786 | 2454.214 | 2454.214 | 43.85 | GPPGSAGSPGKDGLNGLPGPIGPPGPR | Deamidated (NQ); 3 Oxidation (P) |
| 824.4101 | 2470.209 | 2470.209 | 40.9 | GPPGSAGSPGKDGLNGLPGPIGPPGPR | Deamidated (NQ); Oxidation (K); 3 Oxidation (P) |
| 1236.112 | 2470.209 | 2470.209 | 43.48 | GPPGSAGSPGKDGLNGLPGPIGPPGPR | Deamidated (NQ); 4 Oxidation (P) |
| 1236.113 | 2470.211 | 2470.209 | 47.7 | GPPGSAGSPGKDGLNGLPGPIGPPGPR | Deamidated (NQ); Oxidation (K); 3 Oxidation (P) |
| 1236.115 | 2470.215 | 2470.209 | 40.68 | GPPGSAGSPGKDGLNGLPGPIGPPGPR | Deamidated (NQ); 4 Oxidation (P) |
| 1241.11 | 2480.205 | 2480.205 | 56.06 | GDRGETGPAGPPGAPGAPGAPGPVGPAGK | 2 Oxidation (P) |
| 1241.11 | 2480.205 | 2480.205 | 61.17 | GDRGETGPAGPPGAPGAPGAPGPVGPAGK | 2 Oxidation (P) |
| 1244.11 | 2486.205 | 2486.204 | 41.13 | GPPGSAGSPGKDGLNGLPGPIGPPGPR | Deamidated (NQ); 5 Oxidation (P) |
| 829.7425 | 2486.206 | 2486.204 | 48.19 | GPPGSAGSPGKDGLNGLPGPIGPPGPR | Deamidated (NQ); 5 Oxidation (P) |
| 829.7426 | 2486.206 | 2486.204 | 53.24 | GPPGSAGSPGKDGLNGLPGPIGPPGPR | Deamidated (NQ); Oxidation (K); 4 Oxidation (P) |
| 1244.112 | 2486.209 | 2486.204 | 41.26 | GPPGSAGSPGKDGLNGLPGPIGPPGPR | Deamidated (NQ); 5 Oxidation (P) |
| 1244.113 | 2486.211 | 2486.204 | 47.74 | GPPGSAGSPGKDGLNGLPGPIGPPGPR | Deamidated (NQ); 5 Oxidation (P) |
| 829.7454 | 2486.214 | 2486.204 | 48.49 | GPPGSAGSPGKDGLNGLPGPIGPPGPR | Deamidated (NQ); 5 Oxidation (P) |
| 833.074 | 2496.2 | 2496.199 | 51.03 | GDRGETGPAGPPGAPGAPGAPGPVGPAGK | 3 Oxidation (P) |
| 844.7423 | 2531.205 | 2531.204 | 58.22 | GNDGATGAAGPPGPTGPAGPPGFPGAVGAK | Oxidation (K); Oxidation (P) |
| 845.0707 | 2532.19 | 2532.188 | 55.23 | GNDGATGAAGPPGPTGPAGPPGFPGAVGAK | Deamidated (NQ); Oxidation (K); Oxidation (P) |
| 845.0709 | 2532.191 | 2532.188 | 50.83 | GNDGATGAAGPPGPTGPAGPPGFPGAVGAK | Deamidated (NQ); 2 Oxidation (P) |
| 1267.104 | 2532.193 | 2532.188 | 55.02 | GNDGATGAAGPPGPTGPAGPPGFPGAVGAK | Deamidated (NQ); 2 Oxidation (P) |
| 1274.607 | 2547.199 | 2547.199 | 71.78 | GNDGATGAAGPPGPTGPAGPPGFPGAVGAK | 3 Oxidation (P) |
| 850.0743 | 2547.201 | 2547.199 | 58.53 | GNDGATGAAGPPGPTGPAGPPGFPGAVGAK | 3 Oxidation (P) |
| 1274.608 | 2547.201 | 2547.199 | 81.67 | GNDGATGAAGPPGPTGPAGPPGFPGAVGAK | 3 Oxidation (P) |
| 1274.609 | 2547.203 | 2547.199 | 50.23 | GNDGATGAAGPPGPTGPAGPPGFPGAVGAK | 3 Oxidation (P) |
| 850.4017 | 2548.183 | 2548.183 | 57.14 | GNDGATGAAGPPGPTGPAGPPGFPGAVGAK | Deamidated (NQ); 3 Oxidation (P) |
| 1275.099 | 2548.183 | 2548.183 | 48.48 | GNDGATGAAGPPGPTGPAGPPGFPGAVGAK | Deamidated (NQ); 3 Oxidation (P) |
| 1275.1 | 2548.185 | 2548.183 | 63.66 | GNDGATGAAGPPGPTGPAGPPGFPGAVGAK | Deamidated (NQ); 3 Oxidation (P) |
| 1275.1 | 2548.185 | 2548.183 | 63.54 | GNDGATGAAGPPGPTGPAGPPGFPGAVGAK | Deamidated (NQ); 3 Oxidation (P) |
| 1275.1 | 2548.185 | 2548.183 | 57.87 | GNDGATGAAGPPGPTGPAGPPGFPGAVGAK | Deamidated (NQ); 3 Oxidation (P) |
| 1275.101 | 2548.187 | 2548.183 | 40.42 | GNDGATGAAGPPGPTGPAGPPGFPGAVGAK | Deamidated (NQ); 3 Oxidation (P) |
| 1275.101 | 2548.187 | 2548.183 | 43.65 | GNDGATGAAGPPGPTGPAGPPGFPGAVGAK | Deamidated (NQ); 3 Oxidation (P) |
| 850.4045 | 2548.192 | 2548.183 | 50.42 | GNDGATGAAGPPGPTGPAGPPGFPGAVGAK | Deamidated (NQ); Oxidation (K); 2 Oxidation (P) |
| 1275.104 | 2548.193 | 2548.183 | 68.16 | GNDGATGAAGPPGPTGPAGPPGFPGAVGAK | Deamidated (NQ); 3 Oxidation (P) |
| 1282.606 | 2563.197 | 2563.194 | 58.17 | GNDGATGAAGPPGPTGPAGPPGFPGAVGAK | Oxidation (K); 3 Oxidation (P) |
| 855.7326 | 2564.176 | 2564.178 | 55.49 | GNDGATGAAGPPGPTGPAGPPGFPGAVGAK | Deamidated (NQ); Oxidation (K); 3 Oxidation (P) |
| 1283.097 | 2564.179 | 2564.178 | 65.38 | GNDGATGAAGPPGPTGPAGPPGFPGAVGAK | Deamidated (NQ); Oxidation (K); 3 Oxidation (P) |
| 1283.097 | 2564.179 | 2564.178 | 54.15 | GNDGATGAAGPPGPTGPAGPPGFPGAVGAK | Deamidated (NQ); Oxidation (K); 3 Oxidation (P) |
| 897.0919 | 2688.254 | 2688.253 | 43.36 | GFSGLQGPPGPPGSPGEQGPSGASGPAGPR | 2 Oxidation (P) |
| 897.0929 | 2688.257 | 2688.253 | 41.47 | GFSGLQGPPGPPGSPGEQGPSGASGPAGPR | 2 Oxidation (P) |
| 1345.627 | 2689.239 | 2689.237 | 62.72 | GFSGLQGPPGPPGSPGEQGPSGASGPAGPR | Deamidated (NQ); 2 Oxidation (P) |
| 897.4217 | 2689.243 | 2689.237 | 48.27 | GFSGLQGPPGPPGSPGEQGPSGASGPAGPR | Deamidated (NQ); 2 Oxidation (P) |
| 1345.629 | 2689.243 | 2689.237 | 61.51 | GFSGLQGPPGPPGSPGEQGPSGASGPAGPR | Deamidated (NQ); 2 Oxidation (P) |
| 1346.118 | 2690.221 | 2690.221 | 68.16 | GFSGLQGPPGPPGSPGEQGPSGASGPAGPR | 2 Deamidated (NQ); 2 Oxidation (P) |
| 902.423 | 2704.247 | 2704.248 | 48.91 | GFSGLQGPPGPPGSPGEQGPSGASGPAGPR | 3 Oxidation (P) |
| 902.4232 | 2704.248 | 2704.248 | 60.25 | GFSGLQGPPGPPGSPGEQGPSGASGPAGPR | 3 Oxidation (P) |
| 1353.132 | 2704.249 | 2704.248 | 64.52 | GFSGLQGPPGPPGSPGEQGPSGASGPAGPR | 3 Oxidation (P) |
| 1353.133 | 2704.251 | 2704.248 | 74.75 | GFSGLQGPPGPPGSPGEQGPSGASGPAGPR | 3 Oxidation (P) |
| 902.7509 | 2705.231 | 2705.232 | 51.49 | GFSGLQGPPGPPGSPGEQGPSGASGPAGPR | Deamidated (NQ); 3 Oxidation (P) |
| 902.7511 | 2705.232 | 2705.232 | 49.54 | GFSGLQGPPGPPGSPGEQGPSGASGPAGPR | Deamidated (NQ); 3 Oxidation (P) |
| 902.7516 | 2705.233 | 2705.232 | 51.93 | GFSGLQGPPGPPGSPGEQGPSGASGPAGPR | Deamidated (NQ); 3 Oxidation (P) |
| 1353.624 | 2705.233 | 2705.232 | 83.24 | GFSGLQGPPGPPGSPGEQGPSGASGPAGPR | Deamidated (NQ); 3 Oxidation (P) |
| 1353.624 | 2705.233 | 2705.232 | 84.86 | GFSGLQGPPGPPGSPGEQGPSGASGPAGPR | Deamidated (NQ); 3 Oxidation (P) |
| 1353.624 | 2705.233 | 2705.232 | 72.55 | GFSGLQGPPGPPGSPGEQGPSGASGPAGPR | Deamidated (NQ); 3 Oxidation (P) |
| 902.7519 | 2705.234 | 2705.232 | 56.24 | GFSGLQGPPGPPGSPGEQGPSGASGPAGPR | Deamidated (NQ); 3 Oxidation (P) |
| 1354.113 | 2706.211 | 2706.216 | 56.66 | GFSGLQGPPGPPGSPGEQGPSGASGPAGPR | 2 Deamidated (NQ); 3 Oxidation (P) |
| 903.0786 | 2706.214 | 2706.216 | 43.56 | GFSGLQGPPGPPGSPGEQGPSGASGPAGPR | 2 Deamidated (NQ); 3 Oxidation (P) |
| 1354.115 | 2706.215 | 2706.216 | 49.74 | GFSGLQGPPGPPGSPGEQGPSGASGPAGPR | 2 Deamidated (NQ); 3 Oxidation (P) |
| 903.0794 | 2706.216 | 2706.216 | 48.81 | GFSGLQGPPGPPGSPGEQGPSGASGPAGPR | 2 Deamidated (NQ); 3 Oxidation (P) |
| 907.4113 | 2719.212 | 2719.211 | 43.85 | GAPGDRGEPGPPGPAGFAGPPGADGQPGAK | Deamidated (NQ); 5 Oxidation (P) |
| 1427.211 | 2852.407 | 2852.405 | 64.08 | GLTGPIGPPGPAGAPGDKGEAGPSGPAGPTGAR | 2 Oxidation (P) |
| 1427.211 | 2852.407 | 2852.405 | 50.22 | GLTGPIGPPGPAGAPGDKGEAGPSGPAGPTGAR | 2 Oxidation (P) |
| 1427.211 | 2852.407 | 2852.405 | 63.22 | GLTGPIGPPGPAGAPGDKGEAGPSGPAGPTGAR | 2 Oxidation (P) |
| 1427.212 | 2852.409 | 2852.405 | 66.48 | GLTGPIGPPGPAGAPGDKGEAGPSGPAGPTGAR | 2 Oxidation (P) |
| 1435.209 | 2868.403 | 2868.4 | 62.79 | GLTGPIGPPGPAGAPGDKGEAGPSGPAGPTGAR | Oxidation (K); 2 Oxidation (P) |
| 1435.209 | 2868.403 | 2868.4 | 63.82 | GLTGPIGPPGPAGAPGDKGEAGPSGPAGPTGAR | Oxidation (K); 2 Oxidation (P) |
| 1041.166 | 3120.476 | 3120.475 | 42.28 | GEPGDAGAKGDAGPPGPAGPAGPPGPIGNVGAPGPK | 5 Oxidation (P) |
| 1081.468 | 3241.382 | 3241.381 | 45.61 | GANGAPGNDGAKGDAGAPGAPGSQGAPGLQGMPGER | 2 Deamidated (NQ); Oxidation (M); 5 Oxidation (P) |
| 1081.469 | 3241.385 | 3241.381 | 62.14 | GANGAPGNDGAKGDAGAPGAPGSQGAPGLQGMPGER | 2 Deamidated (NQ); Oxidation (M); 5 Oxidation (P) |

| Collagen alpha-1(III) chain OS=Bos taurus GN=COL3A1 PE=1 SV=1 | 434 |
| --- | --- |

| 637.2997 | 1272.585 | 1272.585 | 45.32 | GSPGGPGAAGFPGGR | 2 Oxidation (P) |
| --- | --- | --- | --- | --- | --- |
| 645.2972 | 1288.58 | 1288.58 | 68.51 | GSPGGPGAAGFPGGR | 3 Oxidation (P) |
| 645.2974 | 1288.58 | 1288.58 | 61.29 | GSPGGPGAAGFPGGR | 3 Oxidation (P) |
| 826.9255 | 1651.836 | 1651.835 | 51.46 | GEMGPAGIPGAPGLIGAR | Oxidation (M); Oxidation (P) |
| 565.2922 | 1692.855 | 1692.854 | 43.09 | GVAGEPGRNGLPGGPGLR | Deamidated (NQ); 2 Oxidation (P) |
| 1041.558 | 2081.101 | 2081.102 | 63.86 | GAPGPQGPPGAPGPLGIAGLTGAR | Oxidation (P) |
| 1041.558 | 2081.101 | 2081.102 | 64.44 | GAPGPQGPPGAPGPLGIAGLTGAR | Oxidation (P) |
| 694.7079 | 2081.102 | 2081.102 | 47.68 | GAPGPQGPPGAPGPLGIAGLTGAR | Oxidation (P) |
| 1042.051 | 2082.087 | 2082.086 | 59.74 | GAPGPQGPPGAPGPLGIAGLTGAR | Deamidated (NQ); Oxidation (P) |
| 1042.052 | 2082.089 | 2082.086 | 63.72 | GAPGPQGPPGAPGPLGIAGLTGAR | Deamidated (NQ); Oxidation (P) |
| 700.04 | 2097.098 | 2097.097 | 43.93 | GAPGPQGPPGAPGPLGIAGLTGAR | 2 Oxidation (P) |
| 700.0402 | 2097.099 | 2097.097 | 52.92 | GAPGPQGPPGAPGPLGIAGLTGAR | 2 Oxidation (P) |
| 1049.557 | 2097.099 | 2097.097 | 55.04 | GAPGPQGPPGAPGPLGIAGLTGAR | 2 Oxidation (P) |
| 1049.557 | 2097.099 | 2097.097 | 67.1 | GAPGPQGPPGAPGPLGIAGLTGAR | 2 Oxidation (P) |
| 700.3669 | 2098.079 | 2098.081 | 56.73 | GAPGPQGPPGAPGPLGIAGLTGAR | Deamidated (NQ); 2 Oxidation (P) |
| 700.3676 | 2098.081 | 2098.081 | 60.51 | GAPGPQGPPGAPGPLGIAGLTGAR | Deamidated (NQ); 2 Oxidation (P) |
| 700.3677 | 2098.081 | 2098.081 | 44.98 | GAPGPQGPPGAPGPLGIAGLTGAR | Deamidated (NQ); 2 Oxidation (P) |
| 705.3709 | 2113.091 | 2113.092 | 46.64 | GAPGPQGPPGAPGPLGIAGLTGAR | 3 Oxidation (P) |

**NS2**

| Collagen alpha-2(I) chain OS=Bos taurus GN=COL1A2 PE=1 SV=2 | | | 6605 | |
| --- | --- | --- | --- | --- |
| **pep_exp_mz** | **pep_exp_mr** | **pep_calc_mr** | | **pep_score** | | **pep_seq** |
| 420.7379 | 839.4612 | 839.4614 | | 42.27 | | GVVGPQGAR |
| 420.738 | 839.4614 | 839.4614 | | 42.64 | | GVVGPQGAR |
| 434.7354 | 867.4562 | 867.4563 | | 52.47 | | GPSGPQGIR |
| 434.7354 | 867.4562 | 867.4563 | | 51.2 | | GPSGPQGIR |
| 446.7534 | 891.4922 | 891.4926 | | 44.52 | | PGPIGPAGAR |
| 446.7536 | 891.4926 | 891.4926 | | 49.19 | | PGPIGPAGAR |
| 451.7292 | 901.4438 | 901.444 | | 43.7 | | AGVMGPAGSR |
| 455.7408 | 909.467 | 909.4668 | | 40.08 | | GHNGLDGLK |
| 456.2326 | 910.4506 | 910.4508 | | 43.63 | | GHNGLDGLK |
| 456.2328 | 910.451 | 910.4508 | | 45.83 | | GHNGLDGLK |
| 456.2328 | 910.451 | 910.4508 | | 43.73 | | GHNGLDGLK |
| 459.7268 | 917.439 | 917.4389 | | 46.73 | | AGVMGPAGSR |
| 459.7269 | 917.4392 | 917.4389 | | 47.21 | | AGVMGPAGSR |
| 463.7381 | 925.4616 | 925.4617 | | 42.18 | | GHNGLDGLK |
| 464.2298 | 926.445 | 926.4458 | | 51.59 | | GHNGLDGLK |
| 464.23 | 926.4454 | 926.4458 | | 46.23 | | GHNGLDGLK |
| 521.752 | 1041.489 | 1041.491 | | 53 | | PGEPGLMGPR |
| 521.7521 | 1041.49 | 1041.491 | | 57.37 | | PGEPGLMGPR |
| 542.7852 | 1083.556 | 1083.556 | | 57.16 | | GLVGEPGPAGSK |
| 542.7853 | 1083.556 | 1083.556 | | 54.87 | | GLVGEPGPAGSK |
| 591.8089 | 1181.603 | 1181.604 | | 45.83 | | EGPVGLPGIDGR |
| 591.8092 | 1181.604 | 1181.604 | | 45.02 | | EGPVGLPGIDGR |
| 592.2885 | 1182.562 | 1182.564 | | 56.4 | | GGGPGPMGLMGPR |
| 592.2889 | 1182.563 | 1182.564 | | 52.24 | | GGGPGPMGLMGPR |
| 596.843 | 1191.671 | 1191.672 | | 46.51 | | IGQPGAVGPAGIR |
| 398.2312 | 1191.672 | 1191.672 | | 44.78 | | IGQPGAVGPAGIR |
| 398.2313 | 1191.672 | 1191.672 | | 42.19 | | IGQPGAVGPAGIR |
| 596.8436 | 1191.673 | 1191.672 | | 66.62 | | IGQPGAVGPAGIR |
| 596.8436 | 1191.673 | 1191.672 | | 42.85 | | IGQPGAVGPAGIR |
| 596.8436 | 1191.673 | 1191.672 | | 49.97 | | IGQPGAVGPAGIR |
| 596.8436 | 1191.673 | 1191.672 | | 51.16 | | IGQPGAVGPAGIR |
| 596.8436 | 1191.673 | 1191.672 | | 42.4 | | IGQPGAVGPAGIR |
| 596.8438 | 1191.673 | 1191.672 | | 59.2 | | IGQPGAVGPAGIR |
| 597.3351 | 1192.656 | 1192.656 | | 55.51 | | IGQPGAVGPAGIR |
| 597.3353 | 1192.656 | 1192.656 | | 54.43 | | IGQPGAVGPAGIR |
| 601.2957 | 1200.577 | 1200.578 | | 61.89 | | GEPGNIGFPGPK |
| 601.2959 | 1200.577 | 1200.578 | | 64.45 | | GEPGNIGFPGPK |
| 601.296 | 1200.577 | 1200.578 | | 60.4 | | GEPGNIGFPGPK |
| 601.2961 | 1200.578 | 1200.578 | | 51.74 | | GEPGNIGFPGPK |
| 601.2962 | 1200.578 | 1200.578 | | 69.89 | | GEPGNIGFPGPK |
| 601.2963 | 1200.578 | 1200.578 | | 44.63 | | GEPGNIGFPGPK |
| 601.7881 | 1201.562 | 1201.562 | | 45.59 | | GEPGNIGFPGPK |
| 604.8407 | 1207.667 | 1207.667 | | 46.5 | | IGQPGAVGPAGIR |
| 403.5629 | 1207.667 | 1207.667 | | 43.43 | | IGQPGAVGPAGIR |
| 604.8408 | 1207.667 | 1207.667 | | 49.93 | | IGQPGAVGPAGIR |
| 604.8409 | 1207.667 | 1207.667 | | 72.88 | | IGQPGAVGPAGIR |
| 604.841 | 1207.667 | 1207.667 | | 61.11 | | IGQPGAVGPAGIR |
| 604.841 | 1207.667 | 1207.667 | | 62.89 | | IGQPGAVGPAGIR |
| 403.5631 | 1207.668 | 1207.667 | | 47.41 | | IGQPGAVGPAGIR |
| 604.8411 | 1207.668 | 1207.667 | | 51.62 | | IGQPGAVGPAGIR |
| 604.8412 | 1207.668 | 1207.667 | | 49.13 | | IGQPGAVGPAGIR |
| 605.3329 | 1208.651 | 1208.651 | | 54.79 | | IGQPGAVGPAGIR |
| 605.333 | 1208.651 | 1208.651 | | 54.32 | | IGQPGAVGPAGIR |
| 611.8082 | 1221.602 | 1221.603 | | 43.57 | | GFPGTPGLPGFK |
| 611.8085 | 1221.602 | 1221.603 | | 42.45 | | GFPGTPGLPGFK |
| 611.809 | 1221.603 | 1221.603 | | 41.13 | | GFPGTPGLPGFK |
| 611.809 | 1221.603 | 1221.603 | | 40.33 | | GFPGTPGLPGFK |
| 631.3179 | 1260.621 | 1260.621 | | 40.61 | | GEAGPAGPAGPAGPR |
| 634.341 | 1266.667 | 1266.668 | | 44.42 | | GIPGPVGAAGATGAR |
| 634.341 | 1266.667 | 1266.668 | | 62.67 | | GIPGPVGAAGATGAR |
| 634.3414 | 1266.668 | 1266.668 | | 60.73 | | GIPGPVGAAGATGAR |
| 634.3415 | 1266.668 | 1266.668 | | 61.97 | | GIPGPVGAAGATGAR |
| 634.3415 | 1266.668 | 1266.668 | | 45.17 | | GIPGPVGAAGATGAR |
| 634.3417 | 1266.669 | 1266.668 | | 58.54 | | GIPGPVGAAGATGAR |
| 634.3418 | 1266.669 | 1266.668 | | 62.26 | | GIPGPVGAAGATGAR |
| 644.3197 | 1286.625 | 1286.626 | | 75.38 | | GFPGSPGNIGPAGK |
| 644.3199 | 1286.625 | 1286.626 | | 85.53 | | GFPGSPGNIGPAGK |
| 644.8113 | 1287.608 | 1287.61 | | 53.09 | | GFPGSPGNIGPAGK |
| 644.8113 | 1287.608 | 1287.61 | | 47.32 | | GFPGSPGNIGPAGK |
| 463.2355 | 1386.685 | 1386.685 | | 56.08 | | GETGLRGDIGSPGR |
| 463.2357 | 1386.685 | 1386.685 | | 43.48 | | GETGLRGDIGSPGR |
| 714.3669 | 1426.719 | 1426.721 | | 43.3 | | GIPGEFGLPGPAGAR |
| 714.3671 | 1426.72 | 1426.721 | | 45.59 | | GIPGEFGLPGPAGAR |
| 714.3673 | 1426.72 | 1426.721 | | 51.69 | | GIPGEFGLPGPAGAR |
| 714.3676 | 1426.721 | 1426.721 | | 54.22 | | GIPGEFGLPGPAGAR |
| 714.3676 | 1426.721 | 1426.721 | | 40.67 | | GIPGEFGLPGPAGAR |
| 714.3677 | 1426.721 | 1426.721 | | 61.58 | | GIPGEFGLPGPAGAR |
| 714.3677 | 1426.721 | 1426.721 | | 67.19 | | GIPGEFGLPGPAGAR |
| 714.3677 | 1426.721 | 1426.721 | | 51.17 | | GIPGEFGLPGPAGAR |
| 714.3677 | 1426.721 | 1426.721 | | 41.28 | | GIPGEFGLPGPAGAR |
| 714.3679 | 1426.721 | 1426.721 | | 45.55 | | GIPGEFGLPGPAGAR |
| 714.368 | 1426.721 | 1426.721 | | 48.27 | | GIPGEFGLPGPAGAR |
| 714.3685 | 1426.722 | 1426.721 | | 41.48 | | GIPGEFGLPGPAGAR |
| 722.3647 | 1442.715 | 1442.715 | | 60.25 | | GIPGEFGLPGPAGAR |
| 722.3649 | 1442.715 | 1442.715 | | 64.94 | | GIPGEFGLPGPAGAR |
| 729.3423 | 1456.67 | 1456.67 | | 73.2 | | GDGGPPGATGFPGAAGR |
| 729.3425 | 1456.67 | 1456.67 | | 65.97 | | GDGGPPGATGFPGAAGR |
| 737.3387 | 1472.663 | 1472.665 | | 57.62 | | GDGGPPGATGFPGAAGR |
| 737.3388 | 1472.663 | 1472.665 | | 68.14 | | GDGGPPGATGFPGAAGR |
| 746.8492 | 1491.684 | 1491.684 | | 64.82 | | SGETGASGPPGFVGEK |
| 746.8495 | 1491.684 | 1491.684 | | 72.45 | | SGETGASGPPGFVGEK |
| 511.5986 | 1531.774 | 1531.774 | | 49.01 | | GEPGPAGAVGPAGAVGPR |
| 511.5987 | 1531.774 | 1531.774 | | 43.17 | | GEPGPAGAVGPAGAVGPR |
| 766.8948 | 1531.775 | 1531.774 | | 63.35 | | GEPGPAGAVGPAGAVGPR |
| 766.8948 | 1531.775 | 1531.774 | | 45.19 | | GEPGPAGAVGPAGAVGPR |
| 766.8949 | 1531.775 | 1531.774 | | 83.46 | | GEPGPAGAVGPAGAVGPR |
| 766.8949 | 1531.775 | 1531.774 | | 104.83 | | GEPGPAGAVGPAGAVGPR |
| 766.8952 | 1531.776 | 1531.774 | | 41.76 | | GEPGPAGAVGPAGAVGPR |
| 781.9177 | 1561.821 | 1561.821 | | 47.62 | | GAAGLPGVAGAPGLPGPR |
| 781.918 | 1561.821 | 1561.821 | | 66.98 | | GAAGLPGVAGAPGLPGPR |
| 781.918 | 1561.821 | 1561.821 | | 46.98 | | GAAGLPGVAGAPGLPGPR |
| 781.9183 | 1561.822 | 1561.821 | | 57.98 | | GAAGLPGVAGAPGLPGPR |
| 781.9183 | 1561.822 | 1561.821 | | 62.39 | | GAAGLPGVAGAPGLPGPR |
| 782.9097 | 1563.805 | 1563.805 | | 60.13 | | GFPGTPGLPGFKGIR |
| 782.9098 | 1563.805 | 1563.805 | | 71.86 | | GFPGTPGLPGFKGIR |
| 782.9101 | 1563.806 | 1563.805 | | 43.06 | | GFPGTPGLPGFKGIR |
| 782.9101 | 1563.806 | 1563.805 | | 47.2 | | GFPGTPGLPGFKGIR |
| 790.8869 | 1579.759 | 1579.759 | | 69.47 | | GPPGESGAAGPTGPIGSR |
| 790.8871 | 1579.76 | 1579.759 | | 43.74 | | GPPGESGAAGPTGPIGSR |
| 790.8873 | 1579.76 | 1579.759 | | 83.89 | | GPPGESGAAGPTGPIGSR |
| 790.8873 | 1579.76 | 1579.759 | | 52.23 | | GPPGESGAAGPTGPIGSR |
| 800.4155 | 1598.816 | 1598.817 | | 80.8 | | GELGPVGNPGPAGPAGPR |
| 800.4156 | 1598.817 | 1598.817 | | 61.12 | | GELGPVGNPGPAGPAGPR |
| 808.9047 | 1615.795 | 1615.795 | | 41.38 | | GELGPVGNPGPAGPAGPR |
| 816.9205 | 1631.826 | 1631.827 | | 44.88 | | GSTGEIGPAGPPGPPGLR |
| 816.9208 | 1631.827 | 1631.827 | | 40.65 | | GSTGEIGPAGPPGPPGLR |
| 824.9179 | 1647.821 | 1647.822 | | 77.53 | | GSTGEIGPAGPPGPPGLR |
| 824.918 | 1647.821 | 1647.822 | | 63.81 | | GSTGEIGPAGPPGPPGLR |
| 824.9183 | 1647.822 | 1647.822 | | 43.07 | | GSTGEIGPAGPPGPPGLR |
| 835.9453 | 1669.876 | 1669.875 | | 66.9 | | GAAGIPGGKGEKGETGLR |
| 835.9453 | 1669.876 | 1669.875 | | 74.26 | | GAAGIPGGKGEKGETGLR |
| 843.9427 | 1685.871 | 1685.87 | | 66.05 | | GAAGIPGGKGEKGETGLR |
| 843.943 | 1685.871 | 1685.87 | | 68.1 | | GAAGIPGGKGEKGETGLR |
| 851.9403 | 1701.866 | 1701.865 | | 94.58 | | GAAGIPGGKGEKGETGLR |
| 851.9404 | 1701.866 | 1701.865 | | 59.67 | | GAAGIPGGKGEKGETGLR |
| 594.9442 | 1781.811 | 1781.812 | | 41.77 | | GPNGDSGRPGEPGLMGPR |
| 891.9132 | 1781.812 | 1781.812 | | 40.31 | | GPNGDSGRPGEPGLMGPR |
| 891.9135 | 1781.812 | 1781.812 | | 49.85 | | GPNGDSGRPGEPGLMGPR |
| 595.2719 | 1782.794 | 1782.796 | | 40.35 | | GPNGDSGRPGEPGLMGPR |
| 902.9691 | 1803.924 | 1803.923 | | 47.36 | | RGSTGEIGPAGPPGPPGLR |
| 602.3152 | 1803.924 | 1803.923 | | 47.03 | | RGSTGEIGPAGPPGPPGLR |
| 902.9692 | 1803.924 | 1803.923 | | 48.9 | | RGSTGEIGPAGPPGPPGLR |
| 903.4303 | 1804.846 | 1804.845 | | 49.46 | | GPPGNVGNPGVNGAPGEAGR |
| 607.9541 | 1820.841 | 1820.84 | | 42.88 | | GPPGNVGNPGVNGAPGEAGR |
| 911.4278 | 1820.841 | 1820.84 | | 84.01 | | GPPGNVGNPGVNGAPGEAGR |
| 911.4279 | 1820.841 | 1820.84 | | 75.95 | | GPPGNVGNPGVNGAPGEAGR |
| 911.9186 | 1821.823 | 1821.824 | | 59.59 | | GPPGNVGNPGVNGAPGEAGR |
| 911.9196 | 1821.825 | 1821.824 | | 74.8 | | GPPGNVGNPGVNGAPGEAGR |
| 912.4117 | 1822.809 | 1822.808 | | 49.81 | | GPPGNVGNPGVNGAPGEAGR |
| 912.4117 | 1822.809 | 1822.808 | | 48.82 | | GPPGNVGNPGVNGAPGEAGR |
| 915.4547 | 1828.895 | 1828.896 | | 48.73 | | TGPPGPSGISGPPGPPGPAGK |
| 915.4551 | 1828.896 | 1828.896 | | 43.41 | | TGPPGPSGISGPPGPPGPAGK |
| 641.6487 | 1921.924 | 1921.924 | | 41.6 | | GERGPPGESGAAGPTGPIGSR |
| 641.6487 | 1921.924 | 1921.924 | | 41.01 | | GERGPPGESGAAGPTGPIGSR |
| 961.9701 | 1921.926 | 1921.924 | | 74.93 | | GERGPPGESGAAGPTGPIGSR |
| 999.007 | 1995.999 | 1995.999 | | 93.86 | | HGNRGEPGPAGAVGPAGAVGPR |
| 666.3406 | 1996 | 1995.999 | | 55.23 | | HGNRGEPGPAGAVGPAGAVGPR |
| 999.0076 | 1996.001 | 1995.999 | | 88.05 | | HGNRGEPGPAGAVGPAGAVGPR |
| 666.6683 | 1996.983 | 1996.983 | | 48.41 | | HGNRGEPGPAGAVGPAGAVGPR |
| 666.6685 | 1996.984 | 1996.983 | | 63.03 | | HGNRGEPGPAGAVGPAGAVGPR |
| 1018.005 | 2033.995 | 2033.992 | | 53.92 | | GEVGPAGPNGFAGPAGAAGQPGAK |
| 684.3364 | 2049.987 | 2049.987 | | 40.76 | | GEVGPAGPNGFAGPAGAAGQPGAK |
| 1026.001 | 2049.987 | 2049.987 | | 74.34 | | GEVGPAGPNGFAGPAGAAGQPGAK |
| 1026.002 | 2049.989 | 2049.987 | | 69.01 | | GEVGPAGPNGFAGPAGAAGQPGAK |
| 1026.491 | 2050.967 | 2050.971 | | 62.84 | | GEVGPAGPNGFAGPAGAAGQPGAK |
| 1026.491 | 2050.967 | 2050.971 | | 60.4 | | GEVGPAGPNGFAGPAGAAGQPGAK |
| 1026.492 | 2050.969 | 2050.971 | | 41.69 | | GEVGPAGPNGFAGPAGAAGQPGAK |
| 1026.493 | 2050.971 | 2050.971 | | 49.59 | | GEVGPAGPNGFAGPAGAAGQPGAK |
| 686.0361 | 2055.087 | 2055.086 | | 47.41 | | EGPVGLPGIDGRPGPIGPAGAR |
| 686.0361 | 2055.087 | 2055.086 | | 40.41 | | EGPVGLPGIDGRPGPIGPAGAR |
| 686.0362 | 2055.087 | 2055.086 | | 49.52 | | EGPVGLPGIDGRPGPIGPAGAR |
| 1028.551 | 2055.087 | 2055.086 | | 50.63 | | EGPVGLPGIDGRPGPIGPAGAR |
| 1028.551 | 2055.087 | 2055.086 | | 47.93 | | EGPVGLPGIDGRPGPIGPAGAR |
| 1033.999 | 2065.983 | 2065.982 | | 66.31 | | GEVGPAGPNGFAGPAGAAGQPGAK |
| 1033.999 | 2065.983 | 2065.982 | | 84.23 | | GEVGPAGPNGFAGPAGAAGQPGAK |
| 1036.548 | 2071.081 | 2071.081 | | 51.76 | | EGPVGLPGIDGRPGPIGPAGAR |
| 1058.064 | 2114.113 | 2114.112 | | 48.46 | | GLPGVAGSVGEPGPLGIAGPPGAR |
| 1066.06 | 2130.105 | 2130.107 | | 79.96 | | GLPGVAGSVGEPGPLGIAGPPGAR |
| 1066.06 | 2130.105 | 2130.107 | | 45.59 | | GLPGVAGSVGEPGPLGIAGPPGAR |
| 1066.061 | 2130.107 | 2130.107 | | 113.9 | | GLPGVAGSVGEPGPLGIAGPPGAR |
| 1066.061 | 2130.107 | 2130.107 | | 111.94 | | GLPGVAGSVGEPGPLGIAGPPGAR |
| 1066.061 | 2130.107 | 2130.107 | | 51.43 | | GLPGVAGSVGEPGPLGIAGPPGAR |
| 1066.061 | 2130.107 | 2130.107 | | 51.6 | | GLPGVAGSVGEPGPLGIAGPPGAR |
| 1066.061 | 2130.107 | 2130.107 | | 56.15 | | GLPGVAGSVGEPGPLGIAGPPGAR |
| 1066.061 | 2130.107 | 2130.107 | | 42.73 | | GLPGVAGSVGEPGPLGIAGPPGAR |
| 1066.061 | 2130.107 | 2130.107 | | 70.06 | | GLPGVAGSVGEPGPLGIAGPPGAR |
| 1066.061 | 2130.107 | 2130.107 | | 94.1 | | GLPGVAGSVGEPGPLGIAGPPGAR |
| 1066.061 | 2130.107 | 2130.107 | | 57.8 | | GLPGVAGSVGEPGPLGIAGPPGAR |
| 711.0431 | 2130.108 | 2130.107 | | 41.74 | | GLPGVAGSVGEPGPLGIAGPPGAR |
| 711.0432 | 2130.108 | 2130.107 | | 63.11 | | GLPGVAGSVGEPGPLGIAGPPGAR |
| 711.0433 | 2130.108 | 2130.107 | | 55.71 | | GLPGVAGSVGEPGPLGIAGPPGAR |
| 711.0436 | 2130.109 | 2130.107 | | 81.65 | | GLPGVAGSVGEPGPLGIAGPPGAR |
| 711.0436 | 2130.109 | 2130.107 | | 63.93 | | GLPGVAGSVGEPGPLGIAGPPGAR |
| 1066.062 | 2130.109 | 2130.107 | | 65.25 | | GLPGVAGSVGEPGPLGIAGPPGAR |
| 1066.062 | 2130.109 | 2130.107 | | 115.48 | | GLPGVAGSVGEPGPLGIAGPPGAR |
| 1066.062 | 2130.109 | 2130.107 | | 118.56 | | GLPGVAGSVGEPGPLGIAGPPGAR |
| 1066.062 | 2130.109 | 2130.107 | | 61.3 | | GLPGVAGSVGEPGPLGIAGPPGAR |
| 1066.062 | 2130.109 | 2130.107 | | 59.36 | | GLPGVAGSVGEPGPLGIAGPPGAR |
| 1066.062 | 2130.109 | 2130.107 | | 47.19 | | GLPGVAGSVGEPGPLGIAGPPGAR |
| 1066.062 | 2130.109 | 2130.107 | | 85.58 | | GLPGVAGSVGEPGPLGIAGPPGAR |
| 1066.063 | 2130.111 | 2130.107 | | 51.39 | | GLPGVAGSVGEPGPLGIAGPPGAR |
| 1074.059 | 2146.103 | 2146.102 | | 40.66 | | GLPGVAGSVGEPGPLGIAGPPGAR |
| 1074.059 | 2146.103 | 2146.102 | | 48.88 | | GLPGVAGSVGEPGPLGIAGPPGAR |
| 1074.06 | 2146.105 | 2146.102 | | 47.52 | | GLPGVAGSVGEPGPLGIAGPPGAR |
| 1074.06 | 2146.105 | 2146.102 | | 59.47 | | GLPGVAGSVGEPGPLGIAGPPGAR |
| 721.3456 | 2161.015 | 2161.015 | | 48.83 | | GAPGPDGNNGAQGPPGLQGVQGGK |
| 1081.515 | 2161.015 | 2161.015 | | 42.58 | | GAPGPDGNNGAQGPPGLQGVQGGK |
| 1081.516 | 2161.017 | 2161.015 | | 72.34 | | GAPGPDGNNGAQGPPGLQGVQGGK |
| 1131.069 | 2260.123 | 2260.124 | | 80.73 | | GYPGNAGPVGAAGAPGPQGPVGPVGK |
| 1131.069 | 2260.123 | 2260.124 | | 79.77 | | GYPGNAGPVGAAGAPGPQGPVGPVGK |
| 754.3821 | 2260.125 | 2260.124 | | 48.11 | | GYPGNAGPVGAAGAPGPQGPVGPVGK |
| 798.7189 | 2393.135 | 2393.136 | | 43.61 | | GEVGPAGPNGFAGPAGAAGQPGAKGER |
| 803.7233 | 2408.148 | 2408.147 | | 57.95 | | GEVGPAGPNGFAGPAGAAGQPGAKGER |
| 803.7234 | 2408.148 | 2408.147 | | 54.76 | | GEVGPAGPNGFAGPAGAAGQPGAKGER |
| 1205.082 | 2408.149 | 2408.147 | | 88.99 | | GEVGPAGPNGFAGPAGAAGQPGAKGER |
| 1205.082 | 2408.149 | 2408.147 | | 87.92 | | GEVGPAGPNGFAGPAGAAGQPGAKGER |
| 1205.573 | 2409.131 | 2409.131 | | 85.77 | | GEVGPAGPNGFAGPAGAAGQPGAKGER |
| 1205.573 | 2409.131 | 2409.131 | | 70.32 | | GEVGPAGPNGFAGPAGAAGQPGAKGER |
| 1215.617 | 2429.219 | 2429.219 | | 53.73 | | GEVGLPGLSGPVGPPGNPGANGLPGAK |
| 810.7474 | 2429.22 | 2429.219 | | 43.29 | | GEVGLPGLSGPVGPPGNPGANGLPGAK |
| 810.7477 | 2429.221 | 2429.219 | | 43.84 | | GEVGLPGLSGPVGPPGNPGANGLPGAK |
| 1215.618 | 2429.221 | 2429.219 | | 63.41 | | GEVGLPGLSGPVGPPGNPGANGLPGAK |
| 1216.112 | 2430.209 | 2430.203 | | 58.01 | | GEVGLPGLSGPVGPPGNPGANGLPGAK |
| 1223.615 | 2445.215 | 2445.214 | | 43.97 | | GEVGLPGLSGPVGPPGNPGANGLPGAK |
| 816.0793 | 2445.216 | 2445.214 | | 42.76 | | GEVGLPGLSGPVGPPGNPGANGLPGAK |
| 816.0794 | 2445.216 | 2445.214 | | 44.3 | | GEVGLPGLSGPVGPPGNPGANGLPGAK |
| 1223.616 | 2445.217 | 2445.214 | | 64.59 | | GEVGLPGLSGPVGPPGNPGANGLPGAK |
| 1223.616 | 2445.217 | 2445.214 | | 81.85 | | GEVGLPGLSGPVGPPGNPGANGLPGAK |
| 1223.616 | 2445.217 | 2445.214 | | 55.32 | | GEVGLPGLSGPVGPPGNPGANGLPGAK |
| 1224.108 | 2446.201 | 2446.198 | | 53.23 | | GEVGLPGLSGPVGPPGNPGANGLPGAK |
| 1224.108 | 2446.201 | 2446.198 | | 56 | | GEVGLPGLSGPVGPPGNPGANGLPGAK |
| 1224.109 | 2446.203 | 2446.198 | | 59 | | GEVGLPGLSGPVGPPGNPGANGLPGAK |
| 1287.136 | 2572.257 | 2572.256 | | 106.29 | | GSDGSVGPVGPAGPIGSAGPPGFPGAPGPK |
| 1287.137 | 2572.259 | 2572.256 | | 73.38 | | GSDGSVGPVGPAGPIGSAGPPGFPGAPGPK |
| 861.0848 | 2580.233 | 2580.232 | | 73.07 | | GENGPVGPTGPVGAAGPSGPNGPPGPAGSR |
| 1291.124 | 2580.233 | 2580.232 | | 94.43 | | GENGPVGPTGPVGAAGPSGPNGPPGPAGSR |
| 1291.124 | 2580.233 | 2580.232 | | 74.82 | | GENGPVGPTGPVGAAGPSGPNGPPGPAGSR |
| 861.0856 | 2580.235 | 2580.232 | | 65.42 | | GENGPVGPTGPVGAAGPSGPNGPPGPAGSR |
| 863.7577 | 2588.251 | 2588.251 | | 42.71 | | GSDGSVGPVGPAGPIGSAGPPGFPGAPGPK |
| 1295.133 | 2588.251 | 2588.251 | | 90.87 | | GSDGSVGPVGPAGPIGSAGPPGFPGAPGPK |
| 1295.133 | 2588.251 | 2588.251 | | 51.58 | | GSDGSVGPVGPAGPIGSAGPPGFPGAPGPK |
| 1295.134 | 2588.253 | 2588.251 | | 83.27 | | GSDGSVGPVGPAGPIGSAGPPGFPGAPGPK |
| 1295.134 | 2588.253 | 2588.251 | | 47.24 | | GSDGSVGPVGPAGPIGSAGPPGFPGAPGPK |
| 1295.135 | 2588.255 | 2588.251 | | 81.7 | | GSDGSVGPVGPAGPIGSAGPPGFPGAPGPK |
| 1295.135 | 2588.255 | 2588.251 | | 63.63 | | GSDGSVGPVGPAGPIGSAGPPGFPGAPGPK |
| 884.0922 | 2649.255 | 2649.253 | | 62.69 | | GSPGERGEVGPAGPNGFAGPAGAAGQPGAK |
| 884.0926 | 2649.256 | 2649.253 | | 49.62 | | GSPGERGEVGPAGPNGFAGPAGAAGQPGAK |
| 923.7907 | 2768.35 | 2768.349 | | 40.39 | | GHNGLQGLPGLAGHHGDQGAPGAVGPAGPR |
| 927.7827 | 2780.326 | 2780.323 | | 45.39 | | GAPGAIGAPGPAGANGDRGEAGPAGPAGPAGPR |
| 955.142 | 2862.404 | 2862.401 | | 41.04 | | GPKGENGPVGPTGPVGAAGPSGPNGPPGPAGSR |
| 955.4697 | 2863.387 | 2863.385 | | 49.82 | | GPKGENGPVGPTGPVGAAGPSGPNGPPGPAGSR |
| 996.7748 | 2987.303 | 2987.299 | | 46.37 | | GPPGNVGNPGVNGAPGEAGRDGNPGNDGPPGR |
| 998.1476 | 2991.421 | 2991.418 | | 65.42 | | GSPGERGEVGPAGPNGFAGPAGAAGQPGAKGER |
| 998.1484 | 2991.423 | 2991.418 | | 65.35 | | GSPGERGEVGPAGPNGFAGPAGAAGQPGAKGER |
| 1003.479 | 3007.415 | 3007.413 | | 46.75 | | GSPGERGEVGPAGPNGFAGPAGAAGQPGAKGER |
| 1003.807 | 3008.399 | 3008.397 | | 48.24 | | GSPGERGEVGPAGPNGFAGPAGAAGQPGAKGER |
| 1003.808 | 3008.402 | 3008.397 | | 40.33 | | GSPGERGEVGPAGPNGFAGPAGAAGQPGAKGER |
| 1003.808 | 3008.402 | 3008.397 | | 42.21 | | GSPGERGEVGPAGPNGFAGPAGAAGQPGAKGER |
| 1055.861 | 3164.561 | 3164.56 | | 53.17 | | GTKGPKGENGPVGPTGPVGAAGPSGPNGPPGPAGSR |
| 1055.862 | 3164.564 | 3164.56 | | 45.35 | | GTKGPKGENGPVGPTGPVGAAGPSGPNGPPGPAGSR |
| 1071.18 | 3210.518 | 3210.518 | | 50.96 | | GPSGPPGPDGNKGEPGVVGAPGTAGPSGPSGLPGER |
| 1122.906 | 3365.696 | 3365.698 | | 47.74 | | GLPGLKGHNGLQGLPGLAGHHGDQGAPGAVGPAGPR |
| 1122.912 | 3365.714 | 3365.698 | | 63.72 | | GLPGLKGHNGLQGLPGLAGHHGDQGAPGAVGPAGPR |
| 1127.911 | 3380.711 | 3380.709 | | 50.93 | | GLPGLKGHNGLQGLPGLAGHHGDQGAPGAVGPAGPR |
| 1128.238 | 3381.692 | 3381.693 | | 48.26 | | GLPGLKGHNGLQGLPGLAGHHGDQGAPGAVGPAGPR |
| 1128.238 | 3381.692 | 3381.693 | | 50.22 | | GLPGLKGHNGLQGLPGLAGHHGDQGAPGAVGPAGPR |
| 1128.238 | 3381.692 | 3381.693 | | 56.51 | | GLPGLKGHNGLQGLPGLAGHHGDQGAPGAVGPAGPR |
| 1128.24 | 3381.698 | 3381.693 | | 40.22 | | GLPGLKGHNGLQGLPGLAGHHGDQGAPGAVGPAGPR |
| 1144.545 | 3430.613 | 3430.61 | | 42.24 | | GHNGLDGLKGQPGAPGVKGEPGAPGENGTPGQTGAR |
| 1150.204 | 3447.59 | 3447.589 | | 40.02 | | GHNGLDGLKGQPGAPGVKGEPGAPGENGTPGQTGAR |
| 1150.209 | 3447.605 | 3447.589 | | 45.31 | | GHNGLDGLKGQPGAPGVKGEPGAPGENGTPGQTGAR |
| 1325.344 | 3973.01 | 3973.029 | | 40.01 | | GEVGLPGLSGPVGPPGNPGANGLPGAKGAAGLPGVAGAPGLPGPR |

| Collagen alpha-1(I) chain OS=Bos taurus GN=COL1A1 PE=1 SV=3 | 6303 |
| --- | --- |

| 392.2217 | 782.4288 | 782.4286 | 40.51 | GAAGLPGPK | Oxidation (P) |
| --- | --- | --- | --- | --- | --- |
| 426.2163 | 850.418 | 850.4185 | 44.11 | GFSGLDGAK | |
| 426.2164 | 850.4182 | 850.4185 | 41.58 | GFSGLDGAK | |
| 426.2165 | 850.4184 | 850.4185 | 46.09 | GFSGLDGAK | |
| 426.2165 | 850.4184 | 850.4185 | 40.62 | GFSGLDGAK | |
| 426.2166 | 850.4186 | 850.4185 | 40.13 | GFSGLDGAK | |
| 434.2139 | 866.4132 | 866.4134 | 47.46 | GFSGLDGAK | Oxidation (K) |
| 434.214 | 866.4134 | 866.4134 | 49.52 | GFSGLDGAK | Oxidation (K) |
| 441.7611 | 881.5076 | 881.5083 | 41.87 | GVVGLPGQR | |
| 449.7584 | 897.5022 | 897.5032 | 44.67 | GVVGLPGQR | Oxidation (P) |
| 449.7585 | 897.5024 | 897.5032 | 46.38 | GVVGLPGQR | Oxidation (P) |
| 449.7589 | 897.5032 | 897.5032 | 44.3 | GVVGLPGQR | Oxidation (P) |
| 544.7722 | 1087.53 | 1087.53 | 58.13 | GFPGADGVAGPK | Oxidation (P) |
| 544.7722 | 1087.53 | 1087.53 | 41.39 | GFPGADGVAGPK | Oxidation (P) |
| 544.7723 | 1087.53 | 1087.53 | 65.39 | GFPGADGVAGPK | Oxidation (P) |
| 544.7724 | 1087.53 | 1087.53 | 64.06 | GFPGADGVAGPK | Oxidation (P) |
| 544.7724 | 1087.53 | 1087.53 | 61.47 | GFPGADGVAGPK | Oxidation (P) |
| 553.2914 | 1104.568 | 1104.568 | 50.9 | GVQGPPGPAGPR | Oxidation (P) |
| 553.2914 | 1104.568 | 1104.568 | 50.06 | GVQGPPGPAGPR | Oxidation (P) |
| 565.7972 | 1129.58 | 1129.58 | 48.13 | GLPGTAGLPGMK | Oxidation (M); Oxidation (P) |
| 565.7972 | 1129.58 | 1129.58 | 52.38 | GLPGTAGLPGMK | Oxidation (M); Oxidation (P) |
| 565.7974 | 1129.58 | 1129.58 | 48.29 | GLPGTAGLPGMK | Oxidation (M); Oxidation (P) |
| 573.2914 | 1144.568 | 1144.57 | 54.48 | GQAGVMGFPGPK | |
| 573.2918 | 1144.569 | 1144.57 | 41.48 | GQAGVMGFPGPK | |
| 581.2896 | 1160.565 | 1160.565 | 50.86 | GQAGVMGFPGPK | Oxidation (K) |
| 581.2897 | 1160.565 | 1160.565 | 49.25 | GQAGVMGFPGPK | Oxidation (P) |
| 581.2897 | 1160.565 | 1160.565 | 54.03 | GQAGVMGFPGPK | Oxidation (P) |
| 581.2897 | 1160.565 | 1160.565 | 53.11 | GQAGVMGFPGPK | Oxidation (K) |
| 581.2897 | 1160.565 | 1160.565 | 43.14 | GQAGVMGFPGPK | Oxidation (P) |
| 581.7817 | 1161.549 | 1161.549 | 43.59 | GQAGVMGFPGPK | Deamidated (NQ); Oxidation (K) |
| 588.8223 | 1175.63 | 1175.63 | 46.29 | GVPGPPGAVGPAGK | Oxidation (P) |
| 589.2869 | 1176.559 | 1176.56 | 56.32 | GQAGVMGFPGPK | Oxidation (K); Oxidation (P) |
| 589.287 | 1176.559 | 1176.56 | 51.79 | GQAGVMGFPGPK | Oxidation (K); Oxidation (P) |
| 589.287 | 1176.559 | 1176.56 | 47.12 | GQAGVMGFPGPK | 2 Oxidation (P) |
| 589.2872 | 1176.56 | 1176.56 | 67.97 | GQAGVMGFPGPK | Oxidation (M); Oxidation (P) |
| 589.2873 | 1176.56 | 1176.56 | 50.47 | GQAGVMGFPGPK | Oxidation (M); Oxidation (P) |
| 589.2874 | 1176.56 | 1176.56 | 60.98 | GQAGVMGFPGPK | Oxidation (M); Oxidation (P) |
| 589.2874 | 1176.56 | 1176.56 | 61.76 | GQAGVMGFPGPK | Oxidation (M); Oxidation (P) |
| 589.2875 | 1176.56 | 1176.56 | 56.65 | GQAGVMGFPGPK | Oxidation (K); Oxidation (P) |
| 596.8197 | 1191.625 | 1191.625 | 53.34 | GVPGPPGAVGPAGK | 2 Oxidation (P) |
| 596.8197 | 1191.625 | 1191.625 | 43.58 | GVPGPPGAVGPAGK | 2 Oxidation (P) |
| 621.8018 | 1241.589 | 1241.589 | 40.62 | GLTGSPGSPGPDGK | Oxidation (P) |
| 621.8018 | 1241.589 | 1241.589 | 46.72 | GLTGSPGSPGPDGK | Oxidation (P) |
| 621.8021 | 1241.59 | 1241.589 | 48.58 | GLTGSPGSPGPDGK | Oxidation (P) |
| 629.7992 | 1257.584 | 1257.584 | 51 | GLTGSPGSPGPDGK | 2 Oxidation (P) |
| 629.7993 | 1257.584 | 1257.584 | 41.52 | GLTGSPGSPGPDGK | 2 Oxidation (P) |
| 629.7993 | 1257.584 | 1257.584 | 46.02 | GLTGSPGSPGPDGK | 2 Oxidation (P) |
| 629.7993 | 1257.584 | 1257.584 | 53.44 | GLTGSPGSPGPDGK | 2 Oxidation (P) |
| 629.7994 | 1257.584 | 1257.584 | 56.28 | GLTGSPGSPGPDGK | 2 Oxidation (P) |
| 629.7998 | 1257.585 | 1257.584 | 48.09 | GLTGSPGSPGPDGK | 2 Oxidation (P) |
| 641.3129 | 1280.611 | 1280.611 | 82.09 | GEAGPSGPAGPTGAR | |
| 641.313 | 1280.611 | 1280.611 | 58.92 | GEAGPSGPAGPTGAR | |
| 656.8298 | 1311.645 | 1311.646 | 40.3 | GFPGLPGPSGEPGK | Oxidation (P) |
| 656.8298 | 1311.645 | 1311.646 | 43.56 | GFPGLPGPSGEPGK | Oxidation (P) |
| 664.8278 | 1327.641 | 1327.641 | 54.1 | GFPGLPGPSGEPGK | 2 Oxidation (P) |
| 664.8278 | 1327.641 | 1327.641 | 64.61 | GFPGLPGPSGEPGK | 2 Oxidation (P) |
| 664.8279 | 1327.641 | 1327.641 | 55.81 | GFPGLPGPSGEPGK | 2 Oxidation (P) |
| 664.8279 | 1327.641 | 1327.641 | 65.47 | GFPGLPGPSGEPGK | 2 Oxidation (P) |
| 664.8279 | 1327.641 | 1327.641 | 52.13 | GFPGLPGPSGEPGK | 2 Oxidation (P) |
| 664.828 | 1327.641 | 1327.641 | 71.34 | GFPGLPGPSGEPGK | 2 Oxidation (P) |
| 664.8281 | 1327.642 | 1327.641 | 49.56 | GFPGLPGPSGEPGK | 2 Oxidation (P) |
| 664.8281 | 1327.642 | 1327.641 | 47.27 | GFPGLPGPSGEPGK | 2 Oxidation (P) |
| 664.8289 | 1327.643 | 1327.641 | 45.9 | GFPGLPGPSGEPGK | 2 Oxidation (P) |
| 666.831 | 1331.647 | 1331.647 | 61.17 | GPSGPQGPSGPPGPK | Oxidation (P) |
| 666.8312 | 1331.648 | 1331.647 | 85.78 | GPSGPQGPSGPPGPK | Oxidation (P) |
| 666.8312 | 1331.648 | 1331.647 | 85.05 | GPSGPQGPSGPPGPK | Oxidation (P) |
| 667.3233 | 1332.632 | 1332.631 | 86.08 | GPSGPQGPSGPPGPK | Deamidated (NQ); Oxidation (P) |
| 667.3234 | 1332.632 | 1332.631 | 85.5 | GPSGPQGPSGPPGPK | Deamidated (NQ); Oxidation (P) |
| 672.8249 | 1343.635 | 1343.636 | 40.07 | GFPGLPGPSGEPGK | 3 Oxidation (P) |
| 672.825 | 1343.635 | 1343.636 | 47.45 | GFPGLPGPSGEPGK | 3 Oxidation (P) |
| 672.8253 | 1343.636 | 1343.636 | 46.41 | GFPGLPGPSGEPGK | 3 Oxidation (P) |
| 672.8253 | 1343.636 | 1343.636 | 49.05 | GFPGLPGPSGEPGK | 3 Oxidation (P) |
| 672.8253 | 1343.636 | 1343.636 | 51.69 | GFPGLPGPSGEPGK | 3 Oxidation (P) |
| 672.8253 | 1343.636 | 1343.636 | 40.38 | GFPGLPGPSGEPGK | 3 Oxidation (P) |
| 672.8254 | 1343.636 | 1343.636 | 61.67 | GFPGLPGPSGEPGK | 3 Oxidation (P) |
| 718.344 | 1434.673 | 1434.674 | 54.77 | GEPGPAGLPGPPGER | 3 Oxidation (P) |
| 718.3441 | 1434.674 | 1434.674 | 59.46 | GEPGPAGLPGPPGER | 3 Oxidation (P) |
| 718.3446 | 1434.675 | 1434.674 | 54.6 | GEPGPAGLPGPPGER | 3 Oxidation (P) |
| 718.3446 | 1434.675 | 1434.674 | 49.91 | GEPGPAGLPGPPGER | 3 Oxidation (P) |
| 718.3447 | 1434.675 | 1434.674 | 76.89 | GEPGPAGLPGPPGER | 3 Oxidation (P) |
| 718.3447 | 1434.675 | 1434.674 | 49.21 | GEPGPAGLPGPPGER | 3 Oxidation (P) |
| 718.3449 | 1434.675 | 1434.674 | 58.56 | GEPGPAGLPGPPGER | 3 Oxidation (P) |
| 718.345 | 1434.675 | 1434.674 | 52.42 | GEPGPAGLPGPPGER | 3 Oxidation (P) |
| 728.9046 | 1455.795 | 1455.793 | 69.35 | ALLLQGSNEIEIR | Deamidated (NQ) |
| 728.905 | 1455.795 | 1455.793 | 72 | ALLLQGSNEIEIR | Deamidated (NQ) |
| 730.3486 | 1458.683 | 1458.685 | 51.63 | GSAGPPGATGFPGAAGR | 2 Oxidation (P) |
| 730.3489 | 1458.683 | 1458.685 | 77.32 | GSAGPPGATGFPGAAGR | 2 Oxidation (P) |
| 730.3489 | 1458.683 | 1458.685 | 58.79 | GSAGPPGATGFPGAAGR | 2 Oxidation (P) |
| 730.3493 | 1458.684 | 1458.685 | 90.56 | GSAGPPGATGFPGAAGR | 2 Oxidation (P) |
| 730.3493 | 1458.684 | 1458.685 | 40.08 | GSAGPPGATGFPGAAGR | 2 Oxidation (P) |
| 730.3493 | 1458.684 | 1458.685 | 42.71 | GSAGPPGATGFPGAAGR | 2 Oxidation (P) |
| 730.3493 | 1458.684 | 1458.685 | 59.62 | GSAGPPGATGFPGAAGR | 2 Oxidation (P) |
| 730.3496 | 1458.685 | 1458.685 | 68.8 | GSAGPPGATGFPGAAGR | 2 Oxidation (P) |
| 730.3499 | 1458.685 | 1458.685 | 42.49 | GSAGPPGATGFPGAAGR | 2 Oxidation (P) |
| 730.35 | 1458.685 | 1458.685 | 69.51 | GSAGPPGATGFPGAAGR | 2 Oxidation (P) |
| 730.35 | 1458.685 | 1458.685 | 79.79 | GSAGPPGATGFPGAAGR | 2 Oxidation (P) |
| 730.35 | 1458.685 | 1458.685 | 64.29 | GSAGPPGATGFPGAAGR | 2 Oxidation (P) |
| 730.3501 | 1458.686 | 1458.685 | 94.87 | GSAGPPGATGFPGAAGR | 2 Oxidation (P) |
| 730.3501 | 1458.686 | 1458.685 | 46.2 | GSAGPPGATGFPGAAGR | 2 Oxidation (P) |
| 730.3502 | 1458.686 | 1458.685 | 41.12 | GSAGPPGATGFPGAAGR | 2 Oxidation (P) |
| 730.3502 | 1458.686 | 1458.685 | 106.31 | GSAGPPGATGFPGAAGR | 2 Oxidation (P) |
| 730.3502 | 1458.686 | 1458.685 | 100.33 | GSAGPPGATGFPGAAGR | 2 Oxidation (P) |
| 730.3502 | 1458.686 | 1458.685 | 49.87 | GSAGPPGATGFPGAAGR | 2 Oxidation (P) |
| 730.3503 | 1458.686 | 1458.685 | 60 | GSAGPPGATGFPGAAGR | 2 Oxidation (P) |
| 730.3503 | 1458.686 | 1458.685 | 54.73 | GSAGPPGATGFPGAAGR | 2 Oxidation (P) |
| 730.3503 | 1458.686 | 1458.685 | 48.27 | GSAGPPGATGFPGAAGR | 2 Oxidation (P) |
| 730.3504 | 1458.686 | 1458.685 | 55.57 | GSAGPPGATGFPGAAGR | 2 Oxidation (P) |
| 730.3504 | 1458.686 | 1458.685 | 65.04 | GSAGPPGATGFPGAAGR | 2 Oxidation (P) |
| 730.3506 | 1458.687 | 1458.685 | 58.3 | GSAGPPGATGFPGAAGR | 2 Oxidation (P) |
| 730.3506 | 1458.687 | 1458.685 | 46.46 | GSAGPPGATGFPGAAGR | 2 Oxidation (P) |
| 730.3507 | 1458.687 | 1458.685 | 58.02 | GSAGPPGATGFPGAAGR | 2 Oxidation (P) |
| 730.3509 | 1458.687 | 1458.685 | 46.58 | GSAGPPGATGFPGAAGR | 2 Oxidation (P) |
| 730.351 | 1458.687 | 1458.685 | 52.4 | GSAGPPGATGFPGAAGR | 2 Oxidation (P) |
| 730.351 | 1458.687 | 1458.685 | 46.39 | GSAGPPGATGFPGAAGR | 2 Oxidation (P) |
| 730.3513 | 1458.688 | 1458.685 | 57.11 | GSAGPPGATGFPGAAGR | 2 Oxidation (P) |
| 738.3479 | 1474.681 | 1474.68 | 52.25 | GSAGPPGATGFPGAAGR | 3 Oxidation (P) |
| 520.9422 | 1559.805 | 1559.806 | 47.2 | GETGPAGPAGPIGPVGAR | |
| 780.9097 | 1559.805 | 1559.806 | 56.41 | GETGPAGPAGPIGPVGAR | |
| 780.9101 | 1559.806 | 1559.806 | 51.68 | GETGPAGPAGPIGPVGAR | |
| 780.9105 | 1559.806 | 1559.806 | 62.62 | GETGPAGPAGPIGPVGAR | |
| 780.9111 | 1559.808 | 1559.806 | 54.95 | GETGPAGPAGPIGPVGAR | |
| 781.402 | 1560.789 | 1560.79 | 42.9 | DGLNGLPGPIGPPGPR | 3 Oxidation (P) |
| 781.8946 | 1561.775 | 1561.774 | 43.82 | DGLNGLPGPIGPPGPR | Deamidated (NQ); 3 Oxidation (P) |
| 788.9079 | 1575.801 | 1575.801 | 54.75 | GETGPAGPAGPIGPVGAR | Oxidation (P) |
| 788.908 | 1575.801 | 1575.801 | 65.61 | GETGPAGPAGPIGPVGAR | Oxidation (P) |
| 529.2618 | 1584.764 | 1584.765 | 40.08 | GANGAPGIAGAPGFPGAR | 3 Oxidation (P) |
| 793.3893 | 1584.764 | 1584.765 | 43 | GANGAPGIAGAPGFPGAR | 3 Oxidation (P) |
| 529.262 | 1584.764 | 1584.765 | 40.11 | GANGAPGIAGAPGFPGAR | 3 Oxidation (P) |
| 793.3896 | 1584.765 | 1584.765 | 75.37 | GANGAPGIAGAPGFPGAR | 3 Oxidation (P) |
| 793.3896 | 1584.765 | 1584.765 | 54.87 | GANGAPGIAGAPGFPGAR | 3 Oxidation (P) |
| 793.3897 | 1584.765 | 1584.765 | 43.22 | GANGAPGIAGAPGFPGAR | 3 Oxidation (P) |
| 793.3898 | 1584.765 | 1584.765 | 62.82 | GANGAPGIAGAPGFPGAR | 3 Oxidation (P) |
| 793.3898 | 1584.765 | 1584.765 | 67.27 | GANGAPGIAGAPGFPGAR | 3 Oxidation (P) |
| 793.3899 | 1584.765 | 1584.765 | 63.69 | GANGAPGIAGAPGFPGAR | 3 Oxidation (P) |
| 793.39 | 1584.765 | 1584.765 | 41.75 | GANGAPGIAGAPGFPGAR | 3 Oxidation (P) |
| 793.3901 | 1584.766 | 1584.765 | 49.88 | GANGAPGIAGAPGFPGAR | 3 Oxidation (P) |
| 793.391 | 1584.767 | 1584.765 | 46.6 | GANGAPGIAGAPGFPGAR | 3 Oxidation (P) |
| 793.391 | 1584.767 | 1584.765 | 42.63 | GANGAPGIAGAPGFPGAR | 3 Oxidation (P) |
| 793.8813 | 1585.748 | 1585.749 | 55.87 | GANGAPGIAGAPGFPGAR | Deamidated (NQ); 3 Oxidation (P) |
| 793.8818 | 1585.749 | 1585.749 | 64.8 | GANGAPGIAGAPGFPGAR | Deamidated (NQ); 3 Oxidation (P) |
| 539.2654 | 1614.774 | 1614.775 | 45.79 | GGPGSRGFPGADGVAGPK | 2 Oxidation (P) |
| 539.9338 | 1616.78 | 1616.78 | 41.62 | GFSGLDGAKGDAGPAGPK | Oxidation (K) |
| 539.9339 | 1616.78 | 1616.78 | 45.49 | GFSGLDGAKGDAGPAGPK | Oxidation (K) |
| 809.3976 | 1616.781 | 1616.78 | 70.86 | GFSGLDGAKGDAGPAGPK | Oxidation (K) |
| 809.3984 | 1616.782 | 1616.78 | 63.4 | GFSGLDGAKGDAGPAGPK | Oxidation (K) |
| 809.3986 | 1616.783 | 1616.78 | 83.83 | GFSGLDGAKGDAGPAGPK | Oxidation (K) |
| 809.3992 | 1616.784 | 1616.78 | 54.92 | GFSGLDGAKGDAGPAGPK | Oxidation (K) |
| 818.9241 | 1635.834 | 1635.833 | 87.45 | GAAGLPGPKGDRGDAGPK | Oxidation (P) |
| 818.9242 | 1635.834 | 1635.833 | 49.73 | GAAGLPGPKGDRGDAGPK | Oxidation (P) |
| 828.4106 | 1654.807 | 1654.806 | 73.16 | GFPGADGVAGPKGPAGER | Oxidation (P) |
| 836.4003 | 1670.786 | 1670.786 | 57.76 | GSPGEAGRPGEAGLPGAK | Oxidation (K); 3 Oxidation (P) |
| 836.4007 | 1670.787 | 1670.786 | 40.14 | GSPGEAGRPGEAGLPGAK | Oxidation (K); 3 Oxidation (P) |
| 845.8928 | 1689.771 | 1689.771 | 84.68 | DGEAGAQGPPGPAGPAGER | |
| 845.8929 | 1689.771 | 1689.771 | 42.37 | DGEAGAQGPPGPAGPAGER | |
| 845.8933 | 1689.772 | 1689.771 | 93.69 | DGEAGAQGPPGPAGPAGER | |
| 853.8904 | 1705.766 | 1705.766 | 76.78 | DGEAGAQGPPGPAGPAGER | Oxidation (P) |
| 853.8906 | 1705.767 | 1705.766 | 52.35 | DGEAGAQGPPGPAGPAGER | Oxidation (P) |
| 853.8906 | 1705.767 | 1705.766 | 71.93 | DGEAGAQGPPGPAGPAGER | Oxidation (P) |
| 853.8909 | 1705.767 | 1705.766 | 86.07 | DGEAGAQGPPGPAGPAGER | Oxidation (P) |
| 853.8909 | 1705.767 | 1705.766 | 75.18 | DGEAGAQGPPGPAGPAGER | Oxidation (P) |
| 871.8741 | 1741.734 | 1741.733 | 92.59 | GEPGSPGENGAPGQMGPR | 3 Oxidation (P) |
| 871.8741 | 1741.734 | 1741.733 | 79.93 | GEPGSPGENGAPGQMGPR | 3 Oxidation (P) |
| 872.3661 | 1742.718 | 1742.717 | 93.72 | GEPGSPGENGAPGQMGPR | Deamidated (NQ); 3 Oxidation (P) |
| 872.3665 | 1742.718 | 1742.717 | 69.3 | GEPGSPGENGAPGQMGPR | Deamidated (NQ); 3 Oxidation (P) |
| 900.9388 | 1799.863 | 1799.863 | 69.01 | GPPGPMGPPGLAGPPGESGR | Oxidation (P) |
| 900.9388 | 1799.863 | 1799.863 | 69.09 | GPPGPMGPPGLAGPPGESGR | Oxidation (P) |
| 906.9479 | 1811.881 | 1811.88 | 44.24 | VGPPGPSGNAGPPGPPGPAGK | Oxidation (K); 2 Oxidation (P) |
| 908.9357 | 1815.857 | 1815.857 | 50.77 | GPPGPMGPPGLAGPPGESGR | Oxidation (M); Oxidation (P) |
| 908.9362 | 1815.858 | 1815.857 | 73.69 | GPPGPMGPPGLAGPPGESGR | 2 Oxidation (P) |
| 908.9363 | 1815.858 | 1815.857 | 73.91 | GPPGPMGPPGLAGPPGESGR | 2 Oxidation (P) |
| 908.9365 | 1815.858 | 1815.857 | 40.25 | GPPGPMGPPGLAGPPGESGR | Oxidation (M); Oxidation (P) |
| 914.9458 | 1827.877 | 1827.875 | 41.73 | VGPPGPSGNAGPPGPPGPAGK | 4 Oxidation (P) |
| 916.934 | 1831.853 | 1831.852 | 47.82 | GPPGPMGPPGLAGPPGESGR | Oxidation (M); 2 Oxidation (P) |
| 924.9314 | 1847.848 | 1847.847 | 45.49 | GPPGPMGPPGLAGPPGESGR | Oxidation (M); 3 Oxidation (P) |
| 924.9314 | 1847.848 | 1847.847 | 40.64 | GPPGPMGPPGLAGPPGESGR | Oxidation (M); 3 Oxidation (P) |
| 930.9486 | 1859.883 | 1859.884 | 54.08 | GQAGVMGFPGPKGAAGEPGK | Oxidation (K); 2 Oxidation (P) |
| 930.9493 | 1859.884 | 1859.884 | 67.88 | GQAGVMGFPGPKGAAGEPGK | 2 Oxidation (K); Oxidation (P) |
| 932.4385 | 1862.862 | 1862.865 | 72.81 | GEPGPTGIQGPPGPAGEEGK | 2 Oxidation (P) |
| 932.4389 | 1862.863 | 1862.865 | 65.69 | GEPGPTGIQGPPGPAGEEGK | 2 Oxidation (P) |
| 988.5011 | 1974.988 | 1974.987 | 44.57 | SGDRGETGPAGPAGPIGPVGAR | |
| 659.3365 | 1974.988 | 1974.987 | 50.91 | SGDRGETGPAGPAGPIGPVGAR | |
| 988.5012 | 1974.988 | 1974.987 | 74.62 | SGDRGETGPAGPAGPIGPVGAR | |
| 988.5012 | 1974.988 | 1974.987 | 42.93 | SGDRGETGPAGPAGPIGPVGAR | |
| 988.5013 | 1974.988 | 1974.987 | 73.29 | SGDRGETGPAGPAGPIGPVGAR | |
| 988.5014 | 1974.988 | 1974.987 | 50.45 | SGDRGETGPAGPAGPIGPVGAR | |
| 659.3367 | 1974.988 | 1974.987 | 44.82 | SGDRGETGPAGPAGPIGPVGAR | |
| 988.5019 | 1974.989 | 1974.987 | 64.87 | SGDRGETGPAGPAGPIGPVGAR | |
| 664.6682 | 1990.983 | 1990.982 | 44.82 | SGDRGETGPAGPAGPIGPVGAR | Oxidation (P) |
| 664.6682 | 1990.983 | 1990.982 | 40.36 | SGDRGETGPAGPAGPIGPVGAR | Oxidation (P) |
| 673.996 | 2018.966 | 2018.966 | 66.24 | GEPGPTGIQGPPGPAGEEGKR | 2 Oxidation (P) |
| 1010.491 | 2018.967 | 2018.966 | 52.74 | GEPGPTGIQGPPGPAGEEGKR | 2 Oxidation (P) |
| 1010.491 | 2018.967 | 2018.966 | 70.96 | GEPGPTGIQGPPGPAGEEGKR | 2 Oxidation (P) |
| 686.3314 | 2055.972 | 2055.972 | 43.84 | TGPPGPAGQDGRPGPPGPPGAR | 4 Oxidation (P) |
| 1037.009 | 2072.003 | 2072.004 | 60.18 | GAPGADGPAGAPGTPGPQGIAGQR | Oxidation (P) |
| 691.6752 | 2072.004 | 2072.004 | 41.08 | GAPGADGPAGAPGTPGPQGIAGQR | Oxidation (P) |
| 691.6754 | 2072.004 | 2072.004 | 42.95 | GAPGADGPAGAPGTPGPQGIAGQR | Oxidation (P) |
| 1037.01 | 2072.005 | 2072.004 | 59.54 | GAPGADGPAGAPGTPGPQGIAGQR | Oxidation (P) |
| 1037.01 | 2072.005 | 2072.004 | 55.93 | GAPGADGPAGAPGTPGPQGIAGQR | Oxidation (P) |
| 1037.5 | 2072.985 | 2072.988 | 71.12 | GAPGADGPAGAPGTPGPQGIAGQR | Deamidated (NQ); Oxidation (P) |
| 692.003 | 2072.987 | 2072.988 | 48.47 | GAPGADGPAGAPGTPGPQGIAGQR | Deamidated (NQ); Oxidation (P) |
| 1037.501 | 2072.987 | 2072.988 | 61.41 | GAPGADGPAGAPGTPGPQGIAGQR | Deamidated (NQ); Oxidation (P) |
| 692.0031 | 2072.988 | 2072.988 | 42.45 | GAPGADGPAGAPGTPGPQGIAGQR | Deamidated (NQ); Oxidation (P) |
| 1045.006 | 2087.997 | 2087.999 | 69.2 | GAPGADGPAGAPGTPGPQGIAGQR | 2 Oxidation (P) |
| 1045.006 | 2087.997 | 2087.999 | 69.83 | GAPGADGPAGAPGTPGPQGIAGQR | 2 Oxidation (P) |
| 697.0067 | 2087.998 | 2087.999 | 49.4 | GAPGADGPAGAPGTPGPQGIAGQR | 2 Oxidation (P) |
| 1045.007 | 2087.999 | 2087.999 | 52.77 | GAPGADGPAGAPGTPGPQGIAGQR | 2 Oxidation (P) |
| 1045.008 | 2088.001 | 2087.999 | 52.61 | GAPGADGPAGAPGTPGPQGIAGQR | 2 Oxidation (P) |
| 1045.008 | 2088.001 | 2087.999 | 52.22 | GAPGADGPAGAPGTPGPQGIAGQR | 2 Oxidation (P) |
| 1045.008 | 2088.001 | 2087.999 | 64.65 | GAPGADGPAGAPGTPGPQGIAGQR | 2 Oxidation (P) |
| 1045.5 | 2088.985 | 2088.983 | 56.53 | GAPGADGPAGAPGTPGPQGIAGQR | Deamidated (NQ); 2 Oxidation (P) |
| 1053.005 | 2103.995 | 2103.993 | 55.83 | GAPGADGPAGAPGTPGPQGIAGQR | 3 Oxidation (P) |
| 1053.005 | 2103.995 | 2103.993 | 67.11 | GAPGADGPAGAPGTPGPQGIAGQR | 3 Oxidation (P) |
| 1067.496 | 2132.977 | 2132.976 | 40 | GEPGPPGPAGFAGPPGADGQPGAK | 3 Oxidation (P) |
| 1085.032 | 2168.049 | 2168.05 | 65.66 | GETGPAGPPGAPGAPGAPGPVGPAGK | 3 Oxidation (P) |
| 1085.032 | 2168.049 | 2168.05 | 75.73 | GETGPAGPPGAPGAPGAPGPVGPAGK | 3 Oxidation (P) |
| 723.6906 | 2168.05 | 2168.05 | 41.34 | GETGPAGPPGAPGAPGAPGPVGPAGK | 3 Oxidation (P) |
| 1085.033 | 2168.051 | 2168.05 | 48.93 | GETGPAGPPGAPGAPGAPGPVGPAGK | 3 Oxidation (P) |
| 1085.033 | 2168.051 | 2168.05 | 56.09 | GETGPAGPPGAPGAPGAPGPVGPAGK | 3 Oxidation (P) |
| 1093.03 | 2184.045 | 2184.045 | 76.96 | GETGPAGPPGAPGAPGAPGPVGPAGK | 4 Oxidation (P) |
| 729.0226 | 2184.046 | 2184.045 | 40.03 | GETGPAGPPGAPGAPGAPGPVGPAGK | 4 Oxidation (P) |
| 733.6628 | 2197.967 | 2197.966 | 49.7 | GDAGAPGAPGSQGAPGLQGMPGER | 4 Oxidation (P) |
| 1099.991 | 2197.967 | 2197.966 | 55.4 | GDAGAPGAPGSQGAPGLQGMPGER | 4 Oxidation (P) |
| 1099.991 | 2197.967 | 2197.966 | 58.34 | GDAGAPGAPGSQGAPGLQGMPGER | 4 Oxidation (P) |
| 734.0261 | 2199.057 | 2199.056 | 41.93 | GETGPAGRPGEVGPPGPPGPAGEK | 2 Oxidation (P) |
| 1107.988 | 2213.961 | 2213.961 | 83.12 | GDAGAPGAPGSQGAPGLQGMPGER | Oxidation (M); 4 Oxidation (P) |
| 1107.989 | 2213.963 | 2213.961 | 65.66 | GDAGAPGAPGSQGAPGLQGMPGER | Oxidation (M); 4 Oxidation (P) |
| 1120.043 | 2238.071 | 2238.067 | 40.16 | GFPGLPGPSGEPGKQGPSGASGER | Oxidation (P) |
| 1120.043 | 2238.071 | 2238.067 | 60.76 | GFPGLPGPSGEPGKQGPSGASGER | Oxidation (P) |
| 1136.035 | 2270.055 | 2270.056 | 44.85 | GFPGLPGPSGEPGKQGPSGASGER | Oxidation (K); 2 Oxidation (P) |
| 1136.036 | 2270.057 | 2270.056 | 67.92 | GFPGLPGPSGEPGKQGPSGASGER | Oxidation (K); 2 Oxidation (P) |
| 1136.036 | 2270.057 | 2270.056 | 64.94 | GFPGLPGPSGEPGKQGPSGASGER | Oxidation (K); 2 Oxidation (P) |
| 1136.036 | 2270.057 | 2270.056 | 59.32 | GFPGLPGPSGEPGKQGPSGASGER | Oxidation (K); 2 Oxidation (P) |
| 1146.075 | 2290.135 | 2290.134 | 94.52 | GDAGPPGPAGPAGPPGPIGNVGAPGPK | 2 Oxidation (P) |
| 764.3862 | 2290.137 | 2290.134 | 41.96 | GDAGPPGPAGPAGPPGPIGNVGAPGPK | Oxidation (K); Oxidation (P) |
| 1146.076 | 2290.137 | 2290.134 | 67.74 | GDAGPPGPAGPAGPPGPIGNVGAPGPK | Oxidation (K); Oxidation (P) |
| 1158.528 | 2315.041 | 2315.042 | 40.41 | GEPGPPGPAGAAGPAGNPGADGQPGAK | 4 Oxidation (P) |
| 1158.528 | 2315.041 | 2315.042 | 42.51 | GEPGPPGPAGAAGPAGNPGADGQPGAK | 4 Oxidation (P) |
| 772.6884 | 2315.043 | 2315.042 | 48.41 | GEPGPPGPAGAAGPAGNPGADGQPGAK | 4 Oxidation (P) |
| 772.6884 | 2315.043 | 2315.042 | 40.1 | GEPGPPGPAGAAGPAGNPGADGQPGAK | 4 Oxidation (P) |
| 772.6899 | 2315.048 | 2315.042 | 40.04 | GEPGPPGPAGAAGPAGNPGADGQPGAK | 4 Oxidation (P) |
| 773.0161 | 2316.027 | 2316.026 | 64.84 | GEPGPPGPAGAAGPAGNPGADGQPGAK | Deamidated (NQ); 4 Oxidation (P) |
| 773.0161 | 2316.027 | 2316.026 | 52.48 | GEPGPPGPAGAAGPAGNPGADGQPGAK | Deamidated (NQ); 4 Oxidation (P) |
| 1162.069 | 2322.123 | 2322.124 | 65.68 | GDAGPPGPAGPAGPPGPIGNVGAPGPK | 4 Oxidation (P) |
| 1162.069 | 2322.123 | 2322.124 | 54.58 | GDAGPPGPAGPAGPPGPIGNVGAPGPK | 4 Oxidation (P) |
| 798.3793 | 2392.116 | 2392.114 | 42.34 | GDTGAKGEPGPTGIQGPPGPAGEEGK | 2 Oxidation (P) |
| 1235.621 | 2469.227 | 2469.225 | 42.53 | GPPGSAGSPGKDGLNGLPGPIGPPGPR | 4 Oxidation (P) |
| 1243.618 | 2485.221 | 2485.22 | 52.57 | GPPGSAGSPGKDGLNGLPGPIGPPGPR | 5 Oxidation (P) |
| 1243.618 | 2485.221 | 2485.22 | 71.7 | GPPGSAGSPGKDGLNGLPGPIGPPGPR | 5 Oxidation (P) |
| 1243.619 | 2485.223 | 2485.22 | 47.48 | GPPGSAGSPGKDGLNGLPGPIGPPGPR | 5 Oxidation (P) |
| 1244.112 | 2486.209 | 2486.204 | 40.64 | GPPGSAGSPGKDGLNGLPGPIGPPGPR | Deamidated (NQ); 5 Oxidation (P) |
| 831.7076 | 2492.101 | 2492.099 | 53.75 | GDAGPAGPKGEPGSPGENGAPGQMGPR | 3 Oxidation (P) |
| 832.035 | 2493.083 | 2493.083 | 44.37 | GDAGPAGPKGEPGSPGENGAPGQMGPR | Deamidated (NQ); 3 Oxidation (P) |
| 1249.107 | 2496.199 | 2496.199 | 89.91 | GDRGETGPAGPPGAPGAPGAPGPVGPAGK | 3 Oxidation (P) |
| 833.0739 | 2496.2 | 2496.199 | 44.71 | GDRGETGPAGPPGAPGAPGAPGPVGPAGK | 3 Oxidation (P) |
| 833.0741 | 2496.201 | 2496.199 | 50.64 | GDRGETGPAGPPGAPGAPGAPGPVGPAGK | 3 Oxidation (P) |
| 1257.105 | 2512.195 | 2512.194 | 84.39 | GDRGETGPAGPPGAPGAPGAPGPVGPAGK | 4 Oxidation (P) |
| 1257.105 | 2512.195 | 2512.194 | 74.12 | GDRGETGPAGPPGAPGAPGAPGPVGPAGK | 4 Oxidation (P) |
| 838.4064 | 2512.197 | 2512.194 | 40.8 | GDRGETGPAGPPGAPGAPGAPGPVGPAGK | 4 Oxidation (P) |
| 838.4066 | 2512.198 | 2512.194 | 42.76 | GDRGETGPAGPPGAPGAPGAPGPVGPAGK | 4 Oxidation (P) |
| 844.7424 | 2531.205 | 2531.204 | 44.27 | GNDGATGAAGPPGPTGPAGPPGFPGAVGAK | 2 Oxidation (P) |
| 1266.61 | 2531.205 | 2531.204 | 53 | GNDGATGAAGPPGPTGPAGPPGFPGAVGAK | 2 Oxidation (P) |
| 1266.61 | 2531.205 | 2531.204 | 53.24 | GNDGATGAAGPPGPTGPAGPPGFPGAVGAK | Oxidation (K); Oxidation (P) |
| 850.0739 | 2547.2 | 2547.199 | 43.3 | GNDGATGAAGPPGPTGPAGPPGFPGAVGAK | 3 Oxidation (P) |
| 850.0739 | 2547.2 | 2547.199 | 53.45 | GNDGATGAAGPPGPTGPAGPPGFPGAVGAK | 3 Oxidation (P) |
| 1274.608 | 2547.201 | 2547.199 | 75.24 | GNDGATGAAGPPGPTGPAGPPGFPGAVGAK | 3 Oxidation (P) |
| 1274.608 | 2547.201 | 2547.199 | 79.56 | GNDGATGAAGPPGPTGPAGPPGFPGAVGAK | 3 Oxidation (P) |
| 1274.608 | 2547.201 | 2547.199 | 85.63 | GNDGATGAAGPPGPTGPAGPPGFPGAVGAK | 3 Oxidation (P) |
| 850.4012 | 2548.182 | 2548.183 | 57.57 | GNDGATGAAGPPGPTGPAGPPGFPGAVGAK | Deamidated (NQ); 3 Oxidation (P) |
| 850.402 | 2548.184 | 2548.183 | 52.69 | GNDGATGAAGPPGPTGPAGPPGFPGAVGAK | Deamidated (NQ); 3 Oxidation (P) |
| 850.413 | 2548.217 | 2548.215 | 53.28 | GDTGAKGEPGPTGIQGPPGPAGEEGKR | Oxidation (K); Oxidation (P) |
| 855.4059 | 2563.196 | 2563.194 | 40.18 | GNDGATGAAGPPGPTGPAGPPGFPGAVGAK | Oxidation (K); 3 Oxidation (P) |
| 1282.606 | 2563.197 | 2563.194 | 60.11 | GNDGATGAAGPPGPTGPAGPPGFPGAVGAK | Oxidation (K); 3 Oxidation (P) |
| 1282.606 | 2563.197 | 2563.194 | 82.08 | GNDGATGAAGPPGPTGPAGPPGFPGAVGAK | Oxidation (K); 3 Oxidation (P) |
| 891.7598 | 2672.258 | 2672.258 | 49.05 | GFSGLQGPPGPPGSPGEQGPSGASGPAGPR | Oxidation (P) |
| 891.7599 | 2672.258 | 2672.258 | 46.52 | GFSGLQGPPGPPGSPGEQGPSGASGPAGPR | Oxidation (P) |
| 891.7602 | 2672.259 | 2672.258 | 40.19 | GFSGLQGPPGPPGSPGEQGPSGASGPAGPR | Oxidation (P) |
| 897.0914 | 2688.252 | 2688.253 | 45.83 | GFSGLQGPPGPPGSPGEQGPSGASGPAGPR | 2 Oxidation (P) |
| 897.0917 | 2688.253 | 2688.253 | 51.89 | GFSGLQGPPGPPGSPGEQGPSGASGPAGPR | 2 Oxidation (P) |
| 1345.134 | 2688.253 | 2688.253 | 68.65 | GFSGLQGPPGPPGSPGEQGPSGASGPAGPR | 2 Oxidation (P) |
| 1345.135 | 2688.255 | 2688.253 | 60.75 | GFSGLQGPPGPPGSPGEQGPSGASGPAGPR | 2 Oxidation (P) |
| 902.4225 | 2704.246 | 2704.248 | 53.85 | GFSGLQGPPGPPGSPGEQGPSGASGPAGPR | 3 Oxidation (P) |
| 902.4228 | 2704.247 | 2704.248 | 40.07 | GFSGLQGPPGPPGSPGEQGPSGASGPAGPR | 3 Oxidation (P) |
| 1353.132 | 2704.249 | 2704.248 | 60.24 | GFSGLQGPPGPPGSPGEQGPSGASGPAGPR | 3 Oxidation (P) |
| 1353.132 | 2704.249 | 2704.248 | 67.77 | GFSGLQGPPGPPGSPGEQGPSGASGPAGPR | 3 Oxidation (P) |
| 1353.132 | 2704.249 | 2704.248 | 55.76 | GFSGLQGPPGPPGSPGEQGPSGASGPAGPR | 3 Oxidation (P) |
| 902.4238 | 2704.25 | 2704.248 | 62.07 | GFSGLQGPPGPPGSPGEQGPSGASGPAGPR | 3 Oxidation (P) |
| 902.424 | 2704.25 | 2704.248 | 61.05 | GFSGLQGPPGPPGSPGEQGPSGASGPAGPR | 3 Oxidation (P) |
| 1353.133 | 2704.251 | 2704.248 | 57.7 | GFSGLQGPPGPPGSPGEQGPSGASGPAGPR | 3 Oxidation (P) |
| 902.7512 | 2705.232 | 2705.232 | 63.74 | GFSGLQGPPGPPGSPGEQGPSGASGPAGPR | Deamidated (NQ); 3 Oxidation (P) |
| 915.0998 | 2742.278 | 2742.278 | 57.14 | QGPSGASGERGPPGPMGPPGLAGPPGESGR | Oxidation (M); Oxidation (P) |
| 946.478 | 2836.412 | 2836.41 | 47.13 | GLTGPIGPPGPAGAPGDKGEAGPSGPAGPTGAR | Oxidation (P) |
| 1427.211 | 2852.407 | 2852.405 | 58.24 | GLTGPIGPPGPAGAPGDKGEAGPSGPAGPTGAR | 2 Oxidation (P) |
| 955.4706 | 2863.39 | 2863.385 | 45.01 | GVPGPPGAVGPAGKDGEAGAQGPPGPAGPAGER | 2 Oxidation (P) |
| 1025.17 | 3072.488 | 3072.49 | 49.41 | GEPGDAGAKGDAGPPGPAGPAGPPGPIGNVGAPGPK | Oxidation (K); Oxidation (P) |
| 1025.172 | 3072.494 | 3072.49 | 48.07 | GEPGDAGAKGDAGPPGPAGPAGPPGPIGNVGAPGPK | Oxidation (K); Oxidation (P) |
| 1030.503 | 3088.487 | 3088.485 | 48.68 | GEPGDAGAKGDAGPPGPAGPAGPPGPIGNVGAPGPK | Oxidation (K); 2 Oxidation (P) |
| 1030.503 | 3088.487 | 3088.485 | 41.76 | GEPGDAGAKGDAGPPGPAGPAGPPGPIGNVGAPGPK | Oxidation (K); 2 Oxidation (P) |
| 1037.17 | 3108.488 | 3108.486 | 57.27 | DGSPGAKGDRGETGPAGPPGAPGAPGAPGPVGPAGK | Oxidation (K); 2 Oxidation (P) |
| 1037.171 | 3108.491 | 3108.486 | 41.49 | DGSPGAKGDRGETGPAGPPGAPGAPGAPGPVGPAGK | Oxidation (K); 2 Oxidation (P) |
| 1047.833 | 3140.477 | 3140.476 | 54.51 | DGSPGAKGDRGETGPAGPPGAPGAPGAPGPVGPAGK | Oxidation (K); 4 Oxidation (P) |
| 1081.142 | 3240.404 | 3240.397 | 42.67 | GANGAPGNDGAKGDAGAPGAPGSQGAPGLQGMPGER | Deamidated (NQ); Oxidation (K); 5 Oxidation (P) |
| 1135.886 | 3404.636 | 3404.635 | 40.34 | GEPGDAGAKGDAGPPGPAGPAGPPGPIGNVGAPGPKGAR | 5 Oxidation (P) |

| Alpha-2-HS-glycoprotein OS=Bos taurus GN=AHSG PE=1 SV=2 | 414 |
| --- | --- |

| 408.714 | 815.4134 | 815.4137 | 67.06 | ALGGEDVR |
| --- | --- | --- | --- | --- |
| 408.7141 | 815.4136 | 815.4137 | 62.01 | ALGGEDVR |
| 635.3277 | 1268.641 | 1268.64 | 71.3 | QDGQFSVLFTK |
| 635.3278 | 1268.641 | 1268.64 | 63.74 | QDGQFSVLFTK |
| 635.3278 | 1268.641 | 1268.64 | 61.21 | QDGQFSVLFTK |
| 635.8195 | 1269.624 | 1269.624 | 41.53 | QDGQFSVLFTK |
| 635.8196 | 1269.625 | 1269.624 | 57.03 | QDGQFSVLFTK |
| 737.9224 | 1473.83 | 1473.83 | 56.61 | TPIVGQPSIPGGPVR |
| 737.9228 | 1473.831 | 1473.83 | 63.31 | TPIVGQPSIPGGPVR |
| 738.4147 | 1474.815 | 1474.814 | 58.78 | TPIVGQPSIPGGPVR |
| 565.3055 | 1692.895 | 1692.895 | 65.81 | HTLNQIDSVKVWPR |
| 707.3393 | 2118.996 | 2118.997 | 43.42 | HTFSGVASVESSSGEAFHVGK |
| 707.3399 | 2118.998 | 2118.997 | 54.81 | HTFSGVASVESSSGEAFHVGK |

| Prothrombin OS=Bos taurus GN=F2 PE=1 SV=2 | 344 |
| --- | --- |

| 393.2367 | 784.4588 | 784.4595 | 44.04 | LLHAGFK |
| --- | --- | --- | --- | --- |
| 393.2368 | 784.459 | 784.4595 | 44.01 | LLHAGFK |
| 446.2482 | 890.4818 | 890.4821 | 53.49 | LAVTTSGSR |
| 446.2482 | 890.4818 | 890.4821 | 55.37 | LAVTTSGSR |
| 456.779 | 911.5434 | 911.544 | 43 | VTVEVIPR |
| 456.7793 | 911.544 | 911.544 | 40.27 | VTVEVIPR |
| 574.7794 | 1147.544 | 1147.544 | 49.85 | SGIECQLWR |
| 574.7797 | 1147.545 | 1147.544 | 48.35 | SGIECQLWR |
| 595.2927 | 1188.571 | 1188.572 | 44.29 | YGFYTHVFR |
| 621.8038 | 1241.593 | 1241.593 | 54.51 | ELFESYIEGR |
| 621.8043 | 1241.594 | 1241.593 | 67.05 | ELFESYIEGR |
| 1137.582 | 2273.149 | 2273.151 | 78.64 | IVEGQDAEVGLSPWQVMLFR |
| 1137.583 | 2273.151 | 2273.151 | 76.02 | IVEGQDAEVGLSPWQVMLFR |
| 801.4225 | 2401.246 | 2401.246 | 49.03 | IVEGQDAEVGLSPWQVMLFRK |
| 801.4225 | 2401.246 | 2401.246 | 53.92 | IVEGQDAEVGLSPWQVMLFRK |

| Collagen alpha-1(II) chain OS=Bos taurus GN=COL2A1 PE=1 SV=4 | 310 |
| --- | --- |

| 656.8298 | 1311.645 | 1311.646 | 40.3 | GFPGLPGPSGEPGK | Oxidation (P) |
| --- | --- | --- | --- | --- | --- |
| 656.8298 | 1311.645 | 1311.646 | 43.56 | GFPGLPGPSGEPGK | Oxidation (P) |
| 664.8278 | 1327.641 | 1327.641 | 54.1 | GFPGLPGPSGEPGK | 2 Oxidation (P) |
| 664.8278 | 1327.641 | 1327.641 | 64.61 | GFPGLPGPSGEPGK | 2 Oxidation (P) |
| 664.8279 | 1327.641 | 1327.641 | 55.81 | GFPGLPGPSGEPGK | 2 Oxidation (P) |
| 664.8279 | 1327.641 | 1327.641 | 65.47 | GFPGLPGPSGEPGK | 2 Oxidation (P) |
| 664.8279 | 1327.641 | 1327.641 | 52.13 | GFPGLPGPSGEPGK | 2 Oxidation (P) |
| 664.828 | 1327.641 | 1327.641 | 71.34 | GFPGLPGPSGEPGK | 2 Oxidation (P) |
| 664.8281 | 1327.642 | 1327.641 | 49.56 | GFPGLPGPSGEPGK | 2 Oxidation (P) |
| 664.8281 | 1327.642 | 1327.641 | 47.27 | GFPGLPGPSGEPGK | 2 Oxidation (P) |
| 664.8289 | 1327.643 | 1327.641 | 45.9 | GFPGLPGPSGEPGK | 2 Oxidation (P) |
| 672.8249 | 1343.635 | 1343.636 | 40.07 | GFPGLPGPSGEPGK | 3 Oxidation (P) |
| 672.825 | 1343.635 | 1343.636 | 47.45 | GFPGLPGPSGEPGK | 3 Oxidation (P) |
| 672.8253 | 1343.636 | 1343.636 | 46.41 | GFPGLPGPSGEPGK | 3 Oxidation (P) |
| 672.8253 | 1343.636 | 1343.636 | 49.05 | GFPGLPGPSGEPGK | 3 Oxidation (P) |
| 672.8253 | 1343.636 | 1343.636 | 51.69 | GFPGLPGPSGEPGK | 3 Oxidation (P) |
| 672.8253 | 1343.636 | 1343.636 | 40.38 | GFPGLPGPSGEPGK | 3 Oxidation (P) |
| 672.8254 | 1343.636 | 1343.636 | 61.67 | GFPGLPGPSGEPGK | 3 Oxidation (P) |
| 758.8597 | 1515.705 | 1515.707 | 50.19 | GAQGPPGATGFPGAAGR | 3 Oxidation (P) |
| 758.86 | 1515.705 | 1515.707 | 43.95 | GAQGPPGATGFPGAAGR | 3 Oxidation (P) |

| Serum albumin OS=Bos taurus GN=ALB PE=1 SV=4 | 270 |
| --- | --- |

| | 507.8133 | 1013.612 | 1013.612 | 50.24 | QTALVELLK |  | | --- | --- | --- | --- | --- | --- | | 507.8133 | 1013.612 | 1013.612 | 46.37 | QTALVELLK |  | | 480.6087 | 1438.804 | 1438.805 | 57.79 | RHPEYAVSVLLR |  | | 480.6087 | 1438.804 | 1438.805 | 46.26 | RHPEYAVSVLLR |  | | 740.4019 | 1478.789 | 1478.788 | 98.06 | LGEYGFQNALIVR |  | | 740.403 | 1478.791 | 1478.788 | 69.42 | LGEYGFQNALIVR |  | | 784.3752 | 1566.736 | 1566.735 | 66.31 | DAFLGSFLYEYSR |  | | 784.3755 | 1566.736 | 1566.735 | 56.76 | DAFLGSFLYEYSR |  | | | |  |  |  |  |
| --- | --- | --- | --- | --- | --- | --- | --- | --- | --- | --- | --- | --- | --- | --- | --- | --- | --- | --- | --- | --- | --- | --- | --- | --- | --- | --- | --- | --- | --- | --- | --- | --- | --- | --- | --- | --- | --- | --- | --- | --- | --- | --- | --- | --- | --- | --- | --- | --- | --- | --- | --- | --- | --- | --- |
|  | | |  |  |  |  |
| Biglycan OS=Bos taurus GN=BGN PE=1 SV=3 | 269 |

| 656.8773 | 1311.74 | 1311.74 | 70.56 | IQAIELEDLLR | |
| --- | --- | --- | --- | --- | --- |
| 656.8773 | 1311.74 | 1311.74 | 56.38 | IQAIELEDLLR | |
| 748.3668 | 1494.719 | 1494.718 | 59.62 | VGVNDFCPVGFGVK | Deamidated (NQ) |
| 748.3673 | 1494.72 | 1494.718 | 55.63 | VGVNDFCPVGFGVK | Deamidated (NQ) |
| 748.3681 | 1494.722 | 1494.718 | 63.89 | VGVNDFCPVGFGVK | Deamidated (NQ) |
| 789.9129 | 1577.811 | 1577.812 | 86.38 | MIENGSLSFLPTLR | Deamidated (NQ) |
| 789.9132 | 1577.812 | 1577.812 | 61.64 | MIENGSLSFLPTLR | Deamidated (NQ) |
| 789.9133 | 1577.812 | 1577.812 | 54.27 | MIENGSLSFLPTLR | Deamidated (NQ) |

| Collagen alpha-2(XI) chain OS=Bos taurus GN=COL11A2 PE=3 SV=1 | 238 |
| --- | --- |

| 511.2876 | 1020.561 | 1020.56 | 55.5 | DFSLLTAVR |  |
| --- | --- | --- | --- | --- | --- |
| 615.8269 | 1229.639 | 1229.641 | 47.9 | LGVPGLPGYPGR | 3 Oxidation (P) |
| 615.8272 | 1229.64 | 1229.641 | 53.02 | LGVPGLPGYPGR | 3 Oxidation (P) |
| 615.8273 | 1229.64 | 1229.641 | 56.4 | LGVPGLPGYPGR | 3 Oxidation (P) |
| 671.0533 | 2010.138 | 2010.137 | 50.64 | ARPGLQAPLLTLYSAQGVR | |
| 671.0535 | 2010.139 | 2010.137 | 56.44 | ARPGLQAPLLTLYSAQGVR | |
| 1006.077 | 2010.139 | 2010.137 | 61.89 | ARPGLQAPLLTLYSAQGVR | |
| 1006.077 | 2010.139 | 2010.137 | 63.94 | ARPGLQAPLLTLYSAQGVR | |

| Pigment epithelium-derived factor OS=Bos taurus GN=SERPINF1 PE=1 SV=1 | 233 |
| --- | --- |

| 535.334 | 1068.653 | 1068.654 | 42.72 | TVQAVLTIPK | |
| --- | --- | --- | --- | --- | --- |
| 780.3972 | 1558.78 | 1558.778 | 69.28 | LAAAVSNFGYDLYR | |
| 780.3975 | 1558.78 | 1558.778 | 80.51 | LAAAVSNFGYDLYR | |
| 980.5274 | 2938.56 | 2938.561 | 43.56 | SGESPTANVLLSPLSVATALSALSLGAEQR | |
| 1036.541 | 3106.601 | 3106.586 | 70.41 | ALYYDLISNPDIHGTYKDLLASVTAPQK | Deamidated (NQ) |

| Collagen alpha-1(III) chain OS=Bos taurus GN=COL3A1 PE=1 SV=1 | 213 |
| --- | --- |

| 645.2974 | 1288.58 | 1288.58 | 56.79 | GSPGGPGAAGFPGGR | 3 Oxidation (P) |
| --- | --- | --- | --- | --- | --- |
| 645.2974 | 1288.58 | 1288.58 | 58.55 | GSPGGPGAAGFPGGR | 3 Oxidation (P) |
| 694.7093 | 2081.106 | 2081.102 | 47.81 | GAPGPQGPPGAPGPLGIAGLTGAR | Oxidation (P) |
| 700.0401 | 2097.099 | 2097.097 | 63.66 | GAPGPQGPPGAPGPLGIAGLTGAR | 2 Oxidation (P) |
| 700.0404 | 2097.099 | 2097.097 | 74.9 | GAPGPQGPPGAPGPLGIAGLTGAR | 2 Oxidation (P) |
| 1049.557 | 2097.099 | 2097.097 | 55.83 | GAPGPQGPPGAPGPLGIAGLTGAR | 2 Oxidation (P) |
| 1049.557 | 2097.099 | 2097.097 | 66.54 | GAPGPQGPPGAPGPLGIAGLTGAR | 2 Oxidation (P) |
| 1049.557 | 2097.099 | 2097.097 | 46.57 | GAPGPQGPPGAPGPLGIAGLTGAR | 2 Oxidation (P) |
| 1057.554 | 2113.093 | 2113.092 | 45.7 | GAPGPQGPPGAPGPLGIAGLTGAR | 3 Oxidation (P) |

| Collagen alpha-1(XI) chain (Fragment) OS=Bos taurus GN=COL11A1 PE=1 SV=1 | 201 |
| --- | --- |

| 615.8269 | 1229.639 | 1229.641 | 47.9 | LGVPGLPGYPGR | 3 Oxidation (P) |
| --- | --- | --- | --- | --- | --- |
| 615.8272 | 1229.64 | 1229.641 | 53.02 | LGVPGLPGYPGR | 3 Oxidation (P) |
| 615.8273 | 1229.64 | 1229.641 | 56.4 | LGVPGLPGYPGR | 3 Oxidation (P) |
| 1144.069 | 2286.123 | 2286.124 | 77.06 | TGPPGPGGVVGPQGPTGETGPIGER | Oxidation (P) |
| 1144.07 | 2286.125 | 2286.124 | 64.81 | TGPPGPGGVVGPQGPTGETGPIGER | Oxidation (P) |

| Nucleobindin-1 OS=Bos taurus GN=NUCB1 PE=2 SV=1 | 159 |
| --- | --- |

| 636.8628 | 1271.711 | 1271.709 | 78.17 | DLELLIQTATR |
| --- | --- | --- | --- | --- |
| 636.8629 | 1271.711 | 1271.709 | 86.35 | DLELLIQTATR |
| 644.9993 | 1931.976 | 1931.974 | 56.8 | YLQEVINVLETDGHFR |

| Coagulation factor IX (Fragment) OS=Bos taurus GN=F9 PE=1 SV=1 | 138 |
| --- | --- |

| 511.7982 | 1021.582 | 1021.581 | 40.14 | SASILQYLK |
| --- | --- | --- | --- | --- |
| 610.7905 | 1219.566 | 1219.566 | 65 | FGYGYVSGWGK |
| 610.7906 | 1219.567 | 1219.566 | 58.37 | FGYGYVSGWGK |
| 851.4991 | 1700.984 | 1700.983 | 45.91 | SASILQYLKVPLVDR |

| Decorin OS=Bos taurus GN=DCN PE=1 SV=2 | 72 |
| --- | --- |

| 550.3291 | 1098.644 | 1098.644 | 45.94 | ISPGAFAPLVK |
| --- | --- | --- | --- | --- |
| 921.7852 | 2762.334 | 2762.334 | 40.16 | ASYSGVSLFSNPVQYWEIQPSTFR |

| Coagulation factor X OS=Bos taurus GN=F10 PE=1 SV=1 | 65 |
| --- | --- |

| 447.2455 | 892.4764 | 892.4767 | 52.04 | TGIVSGFGR |
| --- | --- | --- | --- | --- |
| 671.3382 | 1340.662 | 1340.661 | 46.07 | ETYDFDIAVLR |

**WK1**

| Collagen alpha-2(I) chain OS=Bos taurus GN=COL1A2 PE=1 SV=2 | 173 |
| --- | --- |

| **pep_exp_mz** | **pep_exp_mr** | **pep_calc_mr** | **pep_score** | **pep_seq** | **pep_var_mod** |
| --- | --- | --- | --- | --- | --- |
| 456.2326 | 910.4506 | 910.4508 | 45.28 | GHNGLDGLK | Deamidated (NQ) |
| 464.2301 | 926.4456 | 926.4458 | 40.8 | GHNGLDGLK | Deamidated (NQ); Oxidation (K) |
| 464.2303 | 926.446 | 926.4458 | 41.46 | GHNGLDGLK | Deamidated (NQ); Oxidation (K) |
| 464.2303 | 926.446 | 926.4458 | 40.92 | GHNGLDGLK | Deamidated (NQ); Oxidation (K) |
| 714.368 | 1426.721 | 1426.721 | 42.63 | GIPGEFGLPGPAGAR | 2 Oxidation (P) |
| 790.8881 | 1579.762 | 1579.759 | 60.76 | GPPGESGAAGPTGPIGSR | Oxidation (P) |
| 1066.062 | 2130.109 | 2130.107 | 72.35 | GLPGVAGSVGEPGPLGIAGPPGAR | 3 Oxidation (P) |
| 1066.062 | 2130.109 | 2130.107 | 64.19 | GLPGVAGSVGEPGPLGIAGPPGAR | 3 Oxidation (P) |

| Collagen alpha-1(I) chain OS=Bos taurus GN=COL1A1 PE=1 SV=3 | 98 |
| --- | --- |

| 426.2165 | 850.4184 | 850.4185 | 43.78 | GFSGLDGAK | |
| --- | --- | --- | --- | --- | --- |
| 426.2167 | 850.4188 | 850.4185 | 40.02 | GFSGLDGAK | |
| 621.3334 | 1240.652 | 1240.652 | 41.95 | GVVGLPGQRGER | Deamidated (NQ); Oxidation (P) |
| 730.3494 | 1458.684 | 1458.685 | 50.67 | GSAGPPGATGFPGAAGR | 2 Oxidation (P) |
| 730.35 | 1458.685 | 1458.685 | 40.47 | GSAGPPGATGFPGAAGR | 2 Oxidation (P) |
| 730.3502 | 1458.686 | 1458.685 | 55.81 | GSAGPPGATGFPGAAGR | 2 Oxidation (P) |
| 730.3503 | 1458.686 | 1458.685 | 46.48 | GSAGPPGATGFPGAAGR | 2 Oxidation (P) |
| 780.9107 | 1559.807 | 1559.806 | 41.82 | GETGPAGPAGPIGPVGAR | |

**AY5**

| Collagen alpha-2(I) chain OS=Bos taurus GN=COL1A2 PE=1 SV=2 | 4153 |
| --- | --- |

| **pep_exp_mz** | **pep_exp_mr** | **pep_calc_mr** | **pep_score** | **pep_seq** | **pep_var_mod** |
| --- | --- | --- | --- | --- | --- |
| 454.751 | 907.4874 | 907.4876 | 41.26 | PGPIGPAGAR | Oxidation (P) |
| 454.751 | 907.4874 | 907.4876 | 42.73 | PGPIGPAGAR | Oxidation (P) |
| 529.7503 | 1057.486 | 1057.486 | 55.29 | PGEPGLMGPR | 2 Oxidation (P); Oxidation (M) |
| 529.7503 | 1057.486 | 1057.486 | 41.97 | PGEPGLMGPR | 2 Oxidation (P); Oxidation (M) |
| 542.7851 | 1083.556 | 1083.556 | 55.25 | GLVGEPGPAGSK | Oxidation (P) |
| 542.7852 | 1083.556 | 1083.556 | 63.65 | GLVGEPGPAGSK | Oxidation (P) |
| 591.8091 | 1181.604 | 1181.604 | 40.17 | EGPVGLPGIDGR | Oxidation (P) |
| 591.8093 | 1181.604 | 1181.604 | 46.14 | EGPVGLPGIDGR | Oxidation (P) |
| 591.8093 | 1181.604 | 1181.604 | 47.88 | EGPVGLPGIDGR | Oxidation (P) |
| 591.8094 | 1181.604 | 1181.604 | 44.6 | EGPVGLPGIDGR | Oxidation (P) |
| 591.8094 | 1181.604 | 1181.604 | 51.19 | EGPVGLPGIDGR | Oxidation (P) |
| 596.8434 | 1191.672 | 1191.672 | 64.28 | IGQPGAVGPAGIR | |
| 596.8434 | 1191.672 | 1191.672 | 51.89 | IGQPGAVGPAGIR | |
| 597.3354 | 1192.656 | 1192.656 | 56.98 | IGQPGAVGPAGIR | Deamidated (NQ) |
| 597.3355 | 1192.656 | 1192.656 | 47.92 | IGQPGAVGPAGIR | Deamidated (NQ) |
| 601.2957 | 1200.577 | 1200.578 | 57.37 | GEPGNIGFPGPK | 2 Oxidation (P) |
| 601.2958 | 1200.577 | 1200.578 | 46.04 | GEPGNIGFPGPK | 2 Oxidation (P) |
| 601.7881 | 1201.562 | 1201.562 | 41.3 | GEPGNIGFPGPK | Deamidated (NQ); Oxidation (K); Oxidation (P) |
| 601.7881 | 1201.562 | 1201.562 | 52.84 | GEPGNIGFPGPK | Deamidated (NQ); 2 Oxidation (P) |
| 601.7883 | 1201.562 | 1201.562 | 54.47 | GEPGNIGFPGPK | Deamidated (NQ); Oxidation (K); Oxidation (P) |
| 601.7883 | 1201.562 | 1201.562 | 44.11 | GEPGNIGFPGPK | Deamidated (NQ); Oxidation (K); Oxidation (P) |
| 604.8409 | 1207.667 | 1207.667 | 41.71 | IGQPGAVGPAGIR | Oxidation (P) |
| 604.8411 | 1207.668 | 1207.667 | 59.02 | IGQPGAVGPAGIR | Oxidation (P) |
| 604.8411 | 1207.668 | 1207.667 | 45.84 | IGQPGAVGPAGIR | Oxidation (P) |
| 605.3328 | 1208.651 | 1208.651 | 47.69 | IGQPGAVGPAGIR | Deamidated (NQ); Oxidation (P) |
| 605.3329 | 1208.651 | 1208.651 | 53.26 | IGQPGAVGPAGIR | Deamidated (NQ); Oxidation (P) |
| 605.3329 | 1208.651 | 1208.651 | 53.9 | IGQPGAVGPAGIR | Deamidated (NQ); Oxidation (P) |
| 605.3329 | 1208.651 | 1208.651 | 44.36 | IGQPGAVGPAGIR | Deamidated (NQ); Oxidation (P) |
| 605.333 | 1208.651 | 1208.651 | 45.94 | IGQPGAVGPAGIR | Deamidated (NQ); Oxidation (P) |
| 605.3331 | 1208.652 | 1208.651 | 41.34 | IGQPGAVGPAGIR | Deamidated (NQ); Oxidation (P) |
| 605.3333 | 1208.652 | 1208.651 | 49.93 | IGQPGAVGPAGIR | Deamidated (NQ); Oxidation (P) |
| 631.3178 | 1260.621 | 1260.621 | 78.13 | GEAGPAGPAGPAGPR | |
| 631.3178 | 1260.621 | 1260.621 | 75.46 | GEAGPAGPAGPAGPR | |
| 634.3411 | 1266.668 | 1266.668 | 43.72 | GIPGPVGAAGATGAR | Oxidation (P) |
| 634.3412 | 1266.668 | 1266.668 | 55.03 | GIPGPVGAAGATGAR | Oxidation (P) |
| 634.3412 | 1266.668 | 1266.668 | 40.77 | GIPGPVGAAGATGAR | Oxidation (P) |
| 634.3412 | 1266.668 | 1266.668 | 44.79 | GIPGPVGAAGATGAR | Oxidation (P) |
| 634.3413 | 1266.668 | 1266.668 | 74.78 | GIPGPVGAAGATGAR | Oxidation (P) |
| 634.3413 | 1266.668 | 1266.668 | 46.08 | GIPGPVGAAGATGAR | Oxidation (P) |
| 644.3201 | 1286.626 | 1286.626 | 54.11 | GFPGSPGNIGPAGK | 2 Oxidation (P) |
| 644.3202 | 1286.626 | 1286.626 | 71.81 | GFPGSPGNIGPAGK | 2 Oxidation (P) |
| 714.3674 | 1426.72 | 1426.721 | 68.78 | GIPGEFGLPGPAGAR | 2 Oxidation (P) |
| 714.3674 | 1426.72 | 1426.721 | 40.46 | GIPGEFGLPGPAGAR | 2 Oxidation (P) |
| 714.3676 | 1426.721 | 1426.721 | 62.42 | GIPGEFGLPGPAGAR | 2 Oxidation (P) |
| 714.3677 | 1426.721 | 1426.721 | 68.19 | GIPGEFGLPGPAGAR | 2 Oxidation (P) |
| 714.3677 | 1426.721 | 1426.721 | 46.32 | GIPGEFGLPGPAGAR | 2 Oxidation (P) |
| 714.3678 | 1426.721 | 1426.721 | 54.84 | GIPGEFGLPGPAGAR | 2 Oxidation (P) |
| 714.3679 | 1426.721 | 1426.721 | 50.78 | GIPGEFGLPGPAGAR | 2 Oxidation (P) |
| 714.3679 | 1426.721 | 1426.721 | 40.94 | GIPGEFGLPGPAGAR | 2 Oxidation (P) |
| 714.3682 | 1426.722 | 1426.721 | 40.64 | GIPGEFGLPGPAGAR | 2 Oxidation (P) |
| 722.3651 | 1442.716 | 1442.715 | 55.2 | GIPGEFGLPGPAGAR | 3 Oxidation (P) |
| 729.3423 | 1456.67 | 1456.67 | 68.82 | GDGGPPGATGFPGAAGR | Oxidation (P) |
| 729.3426 | 1456.671 | 1456.67 | 82.47 | GDGGPPGATGFPGAAGR | Oxidation (P) |
| 737.3394 | 1472.664 | 1472.665 | 76.73 | GDGGPPGATGFPGAAGR | 2 Oxidation (P) |
| 737.3394 | 1472.664 | 1472.665 | 74.25 | GDGGPPGATGFPGAAGR | 2 Oxidation (P) |
| 737.3397 | 1472.665 | 1472.665 | 45.76 | GDGGPPGATGFPGAAGR | 2 Oxidation (P) |
| 737.3401 | 1472.666 | 1472.665 | 63.68 | GDGGPPGATGFPGAAGR | 2 Oxidation (P) |
| 737.3407 | 1472.667 | 1472.665 | 50.08 | GDGGPPGATGFPGAAGR | 2 Oxidation (P) |
| 746.8495 | 1491.684 | 1491.684 | 74.71 | SGETGASGPPGFVGEK | Oxidation (P) |
| 746.8496 | 1491.685 | 1491.684 | 76.54 | SGETGASGPPGFVGEK | Oxidation (P) |
| 746.8496 | 1491.685 | 1491.684 | 40.86 | SGETGASGPPGFVGEK | Oxidation (P) |
| 754.8471 | 1507.68 | 1507.679 | 63.69 | SGETGASGPPGFVGEK | Oxidation (K); Oxidation (P) |
| 754.8474 | 1507.68 | 1507.679 | 62.55 | SGETGASGPPGFVGEK | Oxidation (K); Oxidation (P) |
| 511.5987 | 1531.774 | 1531.774 | 46.38 | GEPGPAGAVGPAGAVGPR | Oxidation (P) |
| 766.8949 | 1531.775 | 1531.774 | 58.19 | GEPGPAGAVGPAGAVGPR | Oxidation (P) |
| 766.8949 | 1531.775 | 1531.774 | 55.85 | GEPGPAGAVGPAGAVGPR | Oxidation (P) |
| 766.895 | 1531.775 | 1531.774 | 50.04 | GEPGPAGAVGPAGAVGPR | Oxidation (P) |
| 766.8952 | 1531.776 | 1531.774 | 78.92 | GEPGPAGAVGPAGAVGPR | Oxidation (P) |
| 766.8952 | 1531.776 | 1531.774 | 73.36 | GEPGPAGAVGPAGAVGPR | Oxidation (P) |
| 769.3715 | 1536.728 | 1536.728 | 51.95 | GAPGAIGAPGPAGANGDR | 2 Oxidation (P) |
| 769.3716 | 1536.729 | 1536.728 | 57.76 | GAPGAIGAPGPAGANGDR | 2 Oxidation (P) |
| 769.8635 | 1537.712 | 1537.712 | 48.81 | GAPGAIGAPGPAGANGDR | Deamidated (NQ); 2 Oxidation (P) |
| 781.918 | 1561.821 | 1561.821 | 61.92 | GAAGLPGVAGAPGLPGPR | 3 Oxidation (P) |
| 781.9181 | 1561.822 | 1561.821 | 62.8 | GAAGLPGVAGAPGLPGPR | 3 Oxidation (P) |
| 781.9185 | 1561.822 | 1561.821 | 45.45 | GAAGLPGVAGAPGLPGPR | 3 Oxidation (P) |
| 790.8869 | 1579.759 | 1579.759 | 74.51 | GPPGESGAAGPTGPIGSR | Oxidation (P) |
| 790.887 | 1579.759 | 1579.759 | 65.73 | GPPGESGAAGPTGPIGSR | Oxidation (P) |
| 790.8873 | 1579.76 | 1579.759 | 79.93 | GPPGESGAAGPTGPIGSR | Oxidation (P) |
| 790.8874 | 1579.76 | 1579.759 | 80.12 | GPPGESGAAGPTGPIGSR | Oxidation (P) |
| 790.8875 | 1579.76 | 1579.759 | 42.08 | GPPGESGAAGPTGPIGSR | Oxidation (P) |
| 790.8878 | 1579.761 | 1579.759 | 51.73 | GPPGESGAAGPTGPIGSR | Oxidation (P) |
| 800.9077 | 1599.801 | 1599.801 | 58.41 | GELGPVGNPGPAGPAGPR | Deamidated (NQ) |
| 800.9078 | 1599.801 | 1599.801 | 70.34 | GELGPVGNPGPAGPAGPR | Deamidated (NQ) |
| 808.9055 | 1615.796 | 1615.795 | 44.57 | GELGPVGNPGPAGPAGPR | Deamidated (NQ); Oxidation (P) |
| 808.9056 | 1615.797 | 1615.795 | 51.18 | GELGPVGNPGPAGPAGPR | Deamidated (NQ); Oxidation (P) |
| 824.9178 | 1647.821 | 1647.822 | 63.1 | GSTGEIGPAGPPGPPGLR | 2 Oxidation (P) |
| 824.918 | 1647.821 | 1647.822 | 58.11 | GSTGEIGPAGPPGPPGLR | 2 Oxidation (P) |
| 824.9185 | 1647.822 | 1647.822 | 53.52 | GSTGEIGPAGPPGPPGLR | 2 Oxidation (P) |
| 824.9186 | 1647.823 | 1647.822 | 45.22 | GSTGEIGPAGPPGPPGLR | 2 Oxidation (P) |
| 600.6045 | 1798.792 | 1798.79 | 43.72 | GPNGDSGRPGEPGLMGPR | Deamidated (NQ); 2 Oxidation (P); Oxidation (M) |
| 600.6045 | 1798.792 | 1798.79 | 47.03 | GPNGDSGRPGEPGLMGPR | Deamidated (NQ); 2 Oxidation (P); Oxidation (M) |
| 904.9059 | 1807.797 | 1807.797 | 58.19 | GPPGNVGNPGVNGAPGEAGR | 3 Deamidated (NQ); 2 Oxidation (P) |
| 911.92 | 1821.825 | 1821.824 | 53.96 | GPPGNVGNPGVNGAPGEAGR | Deamidated (NQ); 3 Oxidation (P) |
| 911.9205 | 1821.826 | 1821.824 | 66.04 | GPPGNVGNPGVNGAPGEAGR | Deamidated (NQ); 3 Oxidation (P) |
| 912.9037 | 1823.793 | 1823.792 | 90.37 | GPPGNVGNPGVNGAPGEAGR | 3 Deamidated (NQ); 3 Oxidation (P) |
| 912.9042 | 1823.794 | 1823.792 | 84.28 | GPPGNVGNPGVNGAPGEAGR | 3 Deamidated (NQ); 3 Oxidation (P) |
| 912.9056 | 1823.797 | 1823.792 | 72.3 | GPPGNVGNPGVNGAPGEAGR | 3 Deamidated (NQ); 3 Oxidation (P) |
| 915.4554 | 1828.896 | 1828.896 | 46.94 | TGPPGPSGISGPPGPPGPAGK | Oxidation (K); 2 Oxidation (P) |
| 915.4559 | 1828.897 | 1828.896 | 47.44 | TGPPGPSGISGPPGPPGPAGK | Oxidation (K); 2 Oxidation (P) |
| 666.6685 | 1996.984 | 1996.983 | 52.28 | HGNRGEPGPAGAVGPAGAVGPR | Deamidated (NQ); Oxidation (P) |
| 666.6686 | 1996.984 | 1996.983 | 46.84 | HGNRGEPGPAGAVGPAGAVGPR | Deamidated (NQ); Oxidation (P) |
| 684.6642 | 2050.971 | 2050.971 | 43.02 | GEVGPAGPNGFAGPAGAAGQPGAK | Deamidated (NQ); Oxidation (P) |
| 1026.494 | 2050.973 | 2050.971 | 69.51 | GEVGPAGPNGFAGPAGAAGQPGAK | Deamidated (NQ); Oxidation (K) |
| 1026.494 | 2050.973 | 2050.971 | 55.09 | GEVGPAGPNGFAGPAGAAGQPGAK | Deamidated (NQ); Oxidation (P) |
| 684.9923 | 2051.955 | 2051.955 | 46.36 | GEVGPAGPNGFAGPAGAAGQPGAK | 2 Deamidated (NQ); Oxidation (P) |
| 1026.986 | 2051.957 | 2051.955 | 82.52 | GEVGPAGPNGFAGPAGAAGQPGAK | 2 Deamidated (NQ); Oxidation (P) |
| 1026.986 | 2051.957 | 2051.955 | 95.78 | GEVGPAGPNGFAGPAGAAGQPGAK | 2 Deamidated (NQ); Oxidation (P) |
| 1034.49 | 2066.965 | 2066.966 | 52.23 | GEVGPAGPNGFAGPAGAAGQPGAK | Deamidated (NQ); Oxidation (K); Oxidation (P) |
| 1034.49 | 2066.965 | 2066.966 | 62.16 | GEVGPAGPNGFAGPAGAAGQPGAK | Deamidated (NQ); Oxidation (K); Oxidation (P) |
| 1034.983 | 2067.951 | 2067.95 | 52.65 | GEVGPAGPNGFAGPAGAAGQPGAK | 2 Deamidated (NQ); Oxidation (K); Oxidation (P) |
| 1034.983 | 2067.951 | 2067.95 | 57.81 | GEVGPAGPNGFAGPAGAAGQPGAK | 2 Deamidated (NQ); Oxidation (K); Oxidation (P) |
| 1066.06 | 2130.105 | 2130.107 | 69.9 | GLPGVAGSVGEPGPLGIAGPPGAR | 3 Oxidation (P) |
| 1066.061 | 2130.107 | 2130.107 | 85.24 | GLPGVAGSVGEPGPLGIAGPPGAR | 3 Oxidation (P) |
| 1066.061 | 2130.107 | 2130.107 | 96.34 | GLPGVAGSVGEPGPLGIAGPPGAR | 3 Oxidation (P) |
| 1066.061 | 2130.107 | 2130.107 | 74.08 | GLPGVAGSVGEPGPLGIAGPPGAR | 3 Oxidation (P) |
| 1066.061 | 2130.107 | 2130.107 | 55.59 | GLPGVAGSVGEPGPLGIAGPPGAR | 3 Oxidation (P) |
| 1066.061 | 2130.107 | 2130.107 | 55.01 | GLPGVAGSVGEPGPLGIAGPPGAR | 3 Oxidation (P) |
| 1066.061 | 2130.107 | 2130.107 | 42.91 | GLPGVAGSVGEPGPLGIAGPPGAR | 3 Oxidation (P) |
| 1066.061 | 2130.107 | 2130.107 | 78.92 | GLPGVAGSVGEPGPLGIAGPPGAR | 3 Oxidation (P) |
| 1066.061 | 2130.107 | 2130.107 | 80.73 | GLPGVAGSVGEPGPLGIAGPPGAR | 3 Oxidation (P) |
| 1066.061 | 2130.107 | 2130.107 | 42.4 | GLPGVAGSVGEPGPLGIAGPPGAR | 3 Oxidation (P) |
| 1066.061 | 2130.107 | 2130.107 | 83.01 | GLPGVAGSVGEPGPLGIAGPPGAR | 3 Oxidation (P) |
| 711.0433 | 2130.108 | 2130.107 | 56.8 | GLPGVAGSVGEPGPLGIAGPPGAR | 3 Oxidation (P) |
| 711.0433 | 2130.108 | 2130.107 | 44.7 | GLPGVAGSVGEPGPLGIAGPPGAR | 3 Oxidation (P) |
| 1066.062 | 2130.109 | 2130.107 | 85.6 | GLPGVAGSVGEPGPLGIAGPPGAR | 3 Oxidation (P) |
| 1066.062 | 2130.109 | 2130.107 | 53.55 | GLPGVAGSVGEPGPLGIAGPPGAR | 3 Oxidation (P) |
| 1066.062 | 2130.109 | 2130.107 | 65.15 | GLPGVAGSVGEPGPLGIAGPPGAR | 3 Oxidation (P) |
| 1066.062 | 2130.109 | 2130.107 | 68.01 | GLPGVAGSVGEPGPLGIAGPPGAR | 3 Oxidation (P) |
| 1066.062 | 2130.109 | 2130.107 | 61.54 | GLPGVAGSVGEPGPLGIAGPPGAR | 3 Oxidation (P) |
| 1066.062 | 2130.109 | 2130.107 | 66.48 | GLPGVAGSVGEPGPLGIAGPPGAR | 3 Oxidation (P) |
| 1066.062 | 2130.109 | 2130.107 | 67.6 | GLPGVAGSVGEPGPLGIAGPPGAR | 3 Oxidation (P) |
| 1066.062 | 2130.109 | 2130.107 | 77.74 | GLPGVAGSVGEPGPLGIAGPPGAR | 3 Oxidation (P) |
| 1066.062 | 2130.109 | 2130.107 | 87.49 | GLPGVAGSVGEPGPLGIAGPPGAR | 3 Oxidation (P) |
| 1066.062 | 2130.109 | 2130.107 | 48.69 | GLPGVAGSVGEPGPLGIAGPPGAR | 3 Oxidation (P) |
| 1066.063 | 2130.111 | 2130.107 | 71.17 | GLPGVAGSVGEPGPLGIAGPPGAR | 3 Oxidation (P) |
| 1066.063 | 2130.111 | 2130.107 | 86.32 | GLPGVAGSVGEPGPLGIAGPPGAR | 3 Oxidation (P) |
| 1066.064 | 2130.113 | 2130.107 | 43.86 | GLPGVAGSVGEPGPLGIAGPPGAR | 3 Oxidation (P) |
| 754.3824 | 2260.125 | 2260.124 | 74.4 | GYPGNAGPVGAAGAPGPQGPVGPVGK | 2 Oxidation (P) |
| 1131.563 | 2261.111 | 2261.108 | 42.49 | GYPGNAGPVGAAGAPGPQGPVGPVGK | Deamidated (NQ); 2 Oxidation (P) |
| 1215.617 | 2429.219 | 2429.219 | 65.16 | GEVGLPGLSGPVGPPGNPGANGLPGAK | 4 Oxidation (P) |
| 1215.618 | 2429.221 | 2429.219 | 77.22 | GEVGLPGLSGPVGPPGNPGANGLPGAK | 4 Oxidation (P) |
| 1216.109 | 2430.203 | 2430.203 | 52.23 | GEVGLPGLSGPVGPPGNPGANGLPGAK | Deamidated (NQ); 4 Oxidation (P) |
| 1216.11 | 2430.205 | 2430.203 | 79.89 | GEVGLPGLSGPVGPPGNPGANGLPGAK | Deamidated (NQ); 4 Oxidation (P) |
| 811.4035 | 2431.189 | 2431.187 | 58.69 | GEVGLPGLSGPVGPPGNPGANGLPGAK | 2 Deamidated (NQ); 4 Oxidation (P) |
| 1216.602 | 2431.189 | 2431.187 | 79.96 | GEVGLPGLSGPVGPPGNPGANGLPGAK | 2 Deamidated (NQ); 4 Oxidation (P) |
| 1216.602 | 2431.189 | 2431.187 | 61.74 | GEVGLPGLSGPVGPPGNPGANGLPGAK | 2 Deamidated (NQ); 4 Oxidation (P) |
| 1223.615 | 2445.215 | 2445.214 | 65.37 | GEVGLPGLSGPVGPPGNPGANGLPGAK | Oxidation (K); 4 Oxidation (P) |
| 1223.615 | 2445.215 | 2445.214 | 72.5 | GEVGLPGLSGPVGPPGNPGANGLPGAK | Oxidation (K); 4 Oxidation (P) |
| 816.4069 | 2446.199 | 2446.198 | 51.86 | GEVGLPGLSGPVGPPGNPGANGLPGAK | Deamidated (NQ); Oxidation (K); 4 Oxidation (P) |
| 1224.107 | 2446.199 | 2446.198 | 61.85 | GEVGLPGLSGPVGPPGNPGANGLPGAK | Deamidated (NQ); Oxidation (K); 4 Oxidation (P) |
| 1224.107 | 2446.199 | 2446.198 | 52.9 | GEVGLPGLSGPVGPPGNPGANGLPGAK | Deamidated (NQ); Oxidation (K); 4 Oxidation (P) |
| 1224.599 | 2447.183 | 2447.182 | 62.84 | GEVGLPGLSGPVGPPGNPGANGLPGAK | 2 Deamidated (NQ); Oxidation (K); 4 Oxidation (P) |
| 1224.599 | 2447.183 | 2447.182 | 71.23 | GEVGLPGLSGPVGPPGNPGANGLPGAK | 2 Deamidated (NQ); Oxidation (K); 4 Oxidation (P) |
| 1224.6 | 2447.185 | 2447.182 | 54.83 | GEVGLPGLSGPVGPPGNPGANGLPGAK | 2 Deamidated (NQ); Oxidation (K); 4 Oxidation (P) |
| 816.7365 | 2447.188 | 2447.182 | 42.66 | GEVGLPGLSGPVGPPGNPGANGLPGAK | 2 Deamidated (NQ); Oxidation (K); 4 Oxidation (P) |
| 816.7385 | 2447.194 | 2447.182 | 46.71 | GEVGLPGLSGPVGPPGNPGANGLPGAK | 2 Deamidated (NQ); Oxidation (K); 4 Oxidation (P) |
| 858.4262 | 2572.257 | 2572.256 | 40 | GSDGSVGPVGPAGPIGSAGPPGFPGAPGPK | 2 Oxidation (P) |
| 861.0847 | 2580.232 | 2580.232 | 46.42 | GENGPVGPTGPVGAAGPSGPNGPPGPAGSR | Oxidation (P) |
| 861.0853 | 2580.234 | 2580.232 | 56.51 | GENGPVGPTGPVGAAGPSGPNGPPGPAGSR | Oxidation (P) |
| 861.4128 | 2581.217 | 2581.216 | 54.85 | GENGPVGPTGPVGAAGPSGPNGPPGPAGSR | Deamidated (NQ); Oxidation (P) |
| 861.7404 | 2582.199 | 2582.2 | 57.97 | GENGPVGPTGPVGAAGPSGPNGPPGPAGSR | 2 Deamidated (NQ); Oxidation (P) |
| 861.7405 | 2582.2 | 2582.2 | 46.81 | GENGPVGPTGPVGAAGPSGPNGPPGPAGSR | 2 Deamidated (NQ); Oxidation (P) |
| 955.7984 | 2864.373 | 2864.369 | 45.39 | GPKGENGPVGPTGPVGAAGPSGPNGPPGPAGSR | 2 Deamidated (NQ); Oxidation (P) |
| 960.8008 | 2879.381 | 2879.38 | 55.05 | GPKGENGPVGPTGPVGAAGPSGPNGPPGPAGSR | Deamidated (NQ); Oxidation (K); Oxidation (P) |
| 961.1293 | 2880.366 | 2880.364 | 76.79 | GPKGENGPVGPTGPVGAAGPSGPNGPPGPAGSR | 2 Deamidated (NQ); Oxidation (K); Oxidation (P) |
| 961.1293 | 2880.366 | 2880.364 | 62.76 | GPKGENGPVGPTGPVGAAGPSGPNGPPGPAGSR | 2 Deamidated (NQ); Oxidation (K); Oxidation (P) |

| Collagen alpha-1(I) chain OS=Bos taurus GN=COL1A1 PE=1 SV=3 | 4148 |
| --- | --- |

| 544.7722 | 1087.53 | 1087.53 | 55.34 | GFPGADGVAGPK | Oxidation (P) |
| --- | --- | --- | --- | --- | --- |
| 544.7722 | 1087.53 | 1087.53 | 48.42 | GFPGADGVAGPK | Oxidation (P) |
| 553.7831 | 1105.552 | 1105.552 | 48.93 | GVQGPPGPAGPR | Deamidated (NQ); Oxidation (P) |
| 553.7831 | 1105.552 | 1105.552 | 47.84 | GVQGPPGPAGPR | Deamidated (NQ); Oxidation (P) |
| 588.8221 | 1175.63 | 1175.63 | 41.37 | GVPGPPGAVGPAGK | Oxidation (P) |
| 589.2872 | 1176.56 | 1176.56 | 52.28 | GQAGVMGFPGPK | Oxidation (P); Oxidation (M) |
| 589.2872 | 1176.56 | 1176.56 | 53.55 | GQAGVMGFPGPK | Oxidation (P); Oxidation (M) |
| 589.2872 | 1176.56 | 1176.56 | 52.01 | GQAGVMGFPGPK | Oxidation (P); Oxidation (M) |
| 589.2874 | 1176.56 | 1176.56 | 48.87 | GQAGVMGFPGPK | Oxidation (P); Oxidation (M) |
| 589.7791 | 1177.544 | 1177.544 | 68.59 | GQAGVMGFPGPK | Deamidated (NQ); Oxidation (P); Oxidation (M) |
| 589.7792 | 1177.544 | 1177.544 | 62.6 | GQAGVMGFPGPK | Deamidated (NQ); Oxidation (P); Oxidation (M) |
| 596.8197 | 1191.625 | 1191.625 | 44.74 | GVPGPPGAVGPAGK | 2 Oxidation (P) |
| 597.2854 | 1192.556 | 1192.555 | 53.1 | GQAGVMGFPGPK | Oxidation (K); Oxidation (P); Oxidation (M) |
| 597.7763 | 1193.538 | 1193.539 | 41.39 | GQAGVMGFPGPK | Deamidated (NQ); Oxidation (K); Oxidation (P); Oxidation (M) |
| 597.7766 | 1193.539 | 1193.539 | 43.92 | GQAGVMGFPGPK | Deamidated (NQ); Oxidation (K); Oxidation (P); Oxidation (M) |
| 597.7766 | 1193.539 | 1193.539 | 47.65 | GQAGVMGFPGPK | Deamidated (NQ); Oxidation (K); Oxidation (P); Oxidation (M) |
| 621.8017 | 1241.589 | 1241.589 | 56.16 | GLTGSPGSPGPDGK | Oxidation (P) |
| 621.8018 | 1241.589 | 1241.589 | 48.45 | GLTGSPGSPGPDGK | Oxidation (P) |
| 664.8278 | 1327.641 | 1327.641 | 42.08 | GFPGLPGPSGEPGK | 2 Oxidation (P) |
| 664.8279 | 1327.641 | 1327.641 | 46.64 | GFPGLPGPSGEPGK | 2 Oxidation (P) |
| 664.8279 | 1327.641 | 1327.641 | 49.63 | GFPGLPGPSGEPGK | 2 Oxidation (P) |
| 664.8279 | 1327.641 | 1327.641 | 58.63 | GFPGLPGPSGEPGK | 2 Oxidation (P) |
| 667.3226 | 1332.631 | 1332.631 | 60.89 | GPSGPQGPSGPPGPK | Deamidated (NQ); Oxidation (P) |
| 667.3229 | 1332.631 | 1332.631 | 56.16 | GPSGPQGPSGPPGPK | Deamidated (NQ); Oxidation (P) |
| 667.3232 | 1332.632 | 1332.631 | 77.21 | GPSGPQGPSGPPGPK | Deamidated (NQ); Oxidation (P) |
| 667.3232 | 1332.632 | 1332.631 | 74.58 | GPSGPQGPSGPPGPK | Deamidated (NQ); Oxidation (P) |
| 667.3234 | 1332.632 | 1332.631 | 54.26 | GPSGPQGPSGPPGPK | Deamidated (NQ); Oxidation (P) |
| 667.3234 | 1332.632 | 1332.631 | 64.24 | GPSGPQGPSGPPGPK | Deamidated (NQ); Oxidation (P) |
| 672.8253 | 1343.636 | 1343.636 | 52.4 | GFPGLPGPSGEPGK | 3 Oxidation (P) |
| 672.8254 | 1343.636 | 1343.636 | 41.78 | GFPGLPGPSGEPGK | 3 Oxidation (P) |
| 718.3442 | 1434.674 | 1434.674 | 45.63 | GEPGPAGLPGPPGER | 3 Oxidation (P) |
| 718.3442 | 1434.674 | 1434.674 | 48.48 | GEPGPAGLPGPPGER | 3 Oxidation (P) |
| 718.3447 | 1434.675 | 1434.674 | 67.1 | GEPGPAGLPGPPGER | 3 Oxidation (P) |
| 718.3447 | 1434.675 | 1434.674 | 71.34 | GEPGPAGLPGPPGER | 3 Oxidation (P) |
| 718.3449 | 1434.675 | 1434.674 | 49.25 | GEPGPAGLPGPPGER | 3 Oxidation (P) |
| 718.345 | 1434.675 | 1434.674 | 48.56 | GEPGPAGLPGPPGER | 3 Oxidation (P) |
| 730.3497 | 1458.685 | 1458.685 | 50.76 | GSAGPPGATGFPGAAGR | 2 Oxidation (P) |
| 730.3498 | 1458.685 | 1458.685 | 71.63 | GSAGPPGATGFPGAAGR | 2 Oxidation (P) |
| 730.3499 | 1458.685 | 1458.685 | 51.63 | GSAGPPGATGFPGAAGR | 2 Oxidation (P) |
| 730.3501 | 1458.686 | 1458.685 | 86.58 | GSAGPPGATGFPGAAGR | 2 Oxidation (P) |
| 730.3501 | 1458.686 | 1458.685 | 80.91 | GSAGPPGATGFPGAAGR | 2 Oxidation (P) |
| 730.3501 | 1458.686 | 1458.685 | 43.42 | GSAGPPGATGFPGAAGR | 2 Oxidation (P) |
| 730.3502 | 1458.686 | 1458.685 | 65.46 | GSAGPPGATGFPGAAGR | 2 Oxidation (P) |
| 730.3502 | 1458.686 | 1458.685 | 69.03 | GSAGPPGATGFPGAAGR | 2 Oxidation (P) |
| 730.3502 | 1458.686 | 1458.685 | 47.59 | GSAGPPGATGFPGAAGR | 2 Oxidation (P) |
| 730.3502 | 1458.686 | 1458.685 | 64.41 | GSAGPPGATGFPGAAGR | 2 Oxidation (P) |
| 730.3503 | 1458.686 | 1458.685 | 46.86 | GSAGPPGATGFPGAAGR | 2 Oxidation (P) |
| 730.3503 | 1458.686 | 1458.685 | 82.08 | GSAGPPGATGFPGAAGR | 2 Oxidation (P) |
| 730.3503 | 1458.686 | 1458.685 | 82.33 | GSAGPPGATGFPGAAGR | 2 Oxidation (P) |
| 730.3503 | 1458.686 | 1458.685 | 40.56 | GSAGPPGATGFPGAAGR | 2 Oxidation (P) |
| 730.3504 | 1458.686 | 1458.685 | 70.41 | GSAGPPGATGFPGAAGR | 2 Oxidation (P) |
| 730.3504 | 1458.686 | 1458.685 | 63.41 | GSAGPPGATGFPGAAGR | 2 Oxidation (P) |
| 730.3504 | 1458.686 | 1458.685 | 64.58 | GSAGPPGATGFPGAAGR | 2 Oxidation (P) |
| 730.3504 | 1458.686 | 1458.685 | 40.68 | GSAGPPGATGFPGAAGR | 2 Oxidation (P) |
| 730.3505 | 1458.686 | 1458.685 | 41.57 | GSAGPPGATGFPGAAGR | 2 Oxidation (P) |
| 730.351 | 1458.687 | 1458.685 | 42.54 | GSAGPPGATGFPGAAGR | 2 Oxidation (P) |
| 730.351 | 1458.687 | 1458.685 | 44.89 | GSAGPPGATGFPGAAGR | 2 Oxidation (P) |
| 730.3511 | 1458.688 | 1458.685 | 60.07 | GSAGPPGATGFPGAAGR | 2 Oxidation (P) |
| 730.3511 | 1458.688 | 1458.685 | 51.56 | GSAGPPGATGFPGAAGR | 2 Oxidation (P) |
| 730.3511 | 1458.688 | 1458.685 | 47.62 | GSAGPPGATGFPGAAGR | 2 Oxidation (P) |
| 730.3512 | 1458.688 | 1458.685 | 73.69 | GSAGPPGATGFPGAAGR | 2 Oxidation (P) |
| 730.3515 | 1458.688 | 1458.685 | 68.86 | GSAGPPGATGFPGAAGR | 2 Oxidation (P) |
| 730.3516 | 1458.689 | 1458.685 | 45.76 | GSAGPPGATGFPGAAGR | 2 Oxidation (P) |
| 520.9424 | 1559.805 | 1559.806 | 50.8 | GETGPAGPAGPIGPVGAR | |
| 520.9425 | 1559.806 | 1559.806 | 62.35 | GETGPAGPAGPIGPVGAR | |
| 780.9106 | 1559.807 | 1559.806 | 59.41 | GETGPAGPAGPIGPVGAR | |
| 780.9107 | 1559.807 | 1559.806 | 53.61 | GETGPAGPAGPIGPVGAR | |
| 780.9109 | 1559.807 | 1559.806 | 51.07 | GETGPAGPAGPIGPVGAR | |
| 780.9117 | 1559.809 | 1559.806 | 42.41 | GETGPAGPAGPIGPVGAR | |
| 781.3998 | 1560.785 | 1560.79 | 40.51 | DGLNGLPGPIGPPGPR | 3 Oxidation (P) |
| 781.4024 | 1560.79 | 1560.79 | 50.81 | DGLNGLPGPIGPPGPR | 3 Oxidation (P) |
| 781.8914 | 1561.768 | 1561.774 | 50.86 | DGLNGLPGPIGPPGPR | Deamidated (NQ); 3 Oxidation (P) |
| 781.8943 | 1561.774 | 1561.774 | 42.91 | DGLNGLPGPIGPPGPR | Deamidated (NQ); 3 Oxidation (P) |
| 781.8943 | 1561.774 | 1561.774 | 64.26 | DGLNGLPGPIGPPGPR | Deamidated (NQ); 3 Oxidation (P) |
| 781.8945 | 1561.774 | 1561.774 | 47.65 | DGLNGLPGPIGPPGPR | Deamidated (NQ); 3 Oxidation (P) |
| 781.8945 | 1561.774 | 1561.774 | 42.5 | DGLNGLPGPIGPPGPR | Deamidated (NQ); 3 Oxidation (P) |
| 781.8946 | 1561.775 | 1561.774 | 56.04 | DGLNGLPGPIGPPGPR | Deamidated (NQ); 3 Oxidation (P) |
| 781.8947 | 1561.775 | 1561.774 | 43.24 | DGLNGLPGPIGPPGPR | Deamidated (NQ); 3 Oxidation (P) |
| 788.908 | 1575.801 | 1575.801 | 50.22 | GETGPAGPAGPIGPVGAR | Oxidation (P) |
| 788.9081 | 1575.802 | 1575.801 | 63.37 | GETGPAGPAGPIGPVGAR | Oxidation (P) |
| 793.3903 | 1584.766 | 1584.765 | 40.83 | GANGAPGIAGAPGFPGAR | 3 Oxidation (P) |
| 793.3904 | 1584.766 | 1584.765 | 44.42 | GANGAPGIAGAPGFPGAR | 3 Oxidation (P) |
| 793.8796 | 1585.745 | 1585.749 | 47.7 | GANGAPGIAGAPGFPGAR | Deamidated (NQ); 3 Oxidation (P) |
| 793.8807 | 1585.747 | 1585.749 | 48.72 | GANGAPGIAGAPGFPGAR | Deamidated (NQ); 3 Oxidation (P) |
| 793.8808 | 1585.747 | 1585.749 | 49.77 | GANGAPGIAGAPGFPGAR | Deamidated (NQ); 3 Oxidation (P) |
| 793.8813 | 1585.748 | 1585.749 | 44.78 | GANGAPGIAGAPGFPGAR | Deamidated (NQ); 3 Oxidation (P) |
| 793.8817 | 1585.749 | 1585.749 | 40.65 | GANGAPGIAGAPGFPGAR | Deamidated (NQ); 3 Oxidation (P) |
| 793.8818 | 1585.749 | 1585.749 | 74.06 | GANGAPGIAGAPGFPGAR | Deamidated (NQ); 3 Oxidation (P) |
| 793.882 | 1585.749 | 1585.749 | 68.87 | GANGAPGIAGAPGFPGAR | Deamidated (NQ); 3 Oxidation (P) |
| 793.882 | 1585.749 | 1585.749 | 41.23 | GANGAPGIAGAPGFPGAR | Deamidated (NQ); 3 Oxidation (P) |
| 793.882 | 1585.749 | 1585.749 | 40.54 | GANGAPGIAGAPGFPGAR | Deamidated (NQ); 3 Oxidation (P) |
| 793.882 | 1585.749 | 1585.749 | 55.01 | GANGAPGIAGAPGFPGAR | Deamidated (NQ); 3 Oxidation (P) |
| 845.893 | 1689.771 | 1689.771 | 94.49 | DGEAGAQGPPGPAGPAGER | |
| 845.8934 | 1689.772 | 1689.771 | 80.1 | DGEAGAQGPPGPAGPAGER | |
| 846.3845 | 1690.754 | 1690.755 | 54 | DGEAGAQGPPGPAGPAGER | Deamidated (NQ) |
| 846.3848 | 1690.755 | 1690.755 | 71.13 | DGEAGAQGPPGPAGPAGER | Deamidated (NQ) |
| 853.8901 | 1705.766 | 1705.766 | 88.48 | DGEAGAQGPPGPAGPAGER | Oxidation (P) |
| 853.8907 | 1705.767 | 1705.766 | 58.37 | DGEAGAQGPPGPAGPAGER | Oxidation (P) |
| 853.891 | 1705.767 | 1705.766 | 82.75 | DGEAGAQGPPGPAGPAGER | Oxidation (P) |
| 854.3827 | 1706.751 | 1706.75 | 77.79 | DGEAGAQGPPGPAGPAGER | Deamidated (NQ); Oxidation (P) |
| 854.3828 | 1706.751 | 1706.75 | 81.07 | DGEAGAQGPPGPAGPAGER | Deamidated (NQ); Oxidation (P) |
| 907.4396 | 1812.865 | 1812.864 | 63.19 | VGPPGPSGNAGPPGPPGPAGK | Deamidated (NQ); Oxidation (K); 2 Oxidation (P) |
| 907.4398 | 1812.865 | 1812.864 | 49.67 | VGPPGPSGNAGPPGPPGPAGK | Deamidated (NQ); Oxidation (K); 2 Oxidation (P) |
| 907.4412 | 1812.868 | 1812.864 | 42 | VGPPGPSGNAGPPGPPGPAGK | Deamidated (NQ); Oxidation (K); 2 Oxidation (P) |
| 908.9365 | 1815.858 | 1815.857 | 83.1 | GPPGPMGPPGLAGPPGESGR | Oxidation (P); Oxidation (M) |
| 916.9337 | 1831.853 | 1831.852 | 82.49 | GPPGPMGPPGLAGPPGESGR | 2 Oxidation (P); Oxidation (M) |
| 916.9337 | 1831.853 | 1831.852 | 63.25 | GPPGPMGPPGLAGPPGESGR | 2 Oxidation (P); Oxidation (M) |
| 916.9337 | 1831.853 | 1831.852 | 57.13 | GPPGPMGPPGLAGPPGESGR | 2 Oxidation (P); Oxidation (M) |
| 916.934 | 1831.853 | 1831.852 | 79.02 | GPPGPMGPPGLAGPPGESGR | 2 Oxidation (P); Oxidation (M) |
| 611.6252 | 1831.854 | 1831.852 | 47.72 | GPPGPMGPPGLAGPPGESGR | 2 Oxidation (P); Oxidation (M) |
| 924.931 | 1847.847 | 1847.847 | 45.83 | GPPGPMGPPGLAGPPGESGR | 3 Oxidation (P); Oxidation (M) |
| 924.9313 | 1847.848 | 1847.847 | 41.61 | GPPGPMGPPGLAGPPGESGR | 4 Oxidation (P) |
| 924.9315 | 1847.848 | 1847.847 | 43.8 | GPPGPMGPPGLAGPPGESGR | 4 Oxidation (P) |
| 924.9323 | 1847.85 | 1847.847 | 40.55 | GPPGPMGPPGLAGPPGESGR | 3 Oxidation (P); Oxidation (M) |
| 932.4403 | 1862.866 | 1862.865 | 48.46 | GEPGPTGIQGPPGPAGEEGK | 2 Oxidation (P) |
| 932.4407 | 1862.867 | 1862.865 | 89.1 | GEPGPTGIQGPPGPAGEEGK | 2 Oxidation (P) |
| 932.9317 | 1863.849 | 1863.849 | 75.91 | GEPGPTGIQGPPGPAGEEGK | Deamidated (NQ); 2 Oxidation (P) |
| 932.9322 | 1863.85 | 1863.849 | 52.67 | GEPGPTGIQGPPGPAGEEGK | Deamidated (NQ); 2 Oxidation (P) |
| 659.3362 | 1974.987 | 1974.987 | 49.97 | SGDRGETGPAGPAGPIGPVGAR | |
| 988.5018 | 1974.989 | 1974.987 | 84.36 | SGDRGETGPAGPAGPIGPVGAR | |
| 1010.493 | 2018.971 | 2018.966 | 74.77 | GEPGPTGIQGPPGPAGEEGKR | 2 Oxidation (P) |
| 1010.494 | 2018.973 | 2018.966 | 57.9 | GEPGPTGIQGPPGPAGEEGKR | 2 Oxidation (P) |
| 686.6595 | 2056.957 | 2056.956 | 41.04 | TGPPGPAGQDGRPGPPGPPGAR | Deamidated (NQ); 4 Oxidation (P) |
| 1037.502 | 2072.989 | 2072.988 | 73.95 | GAPGADGPAGAPGTPGPQGIAGQR | Deamidated (NQ); Oxidation (P) |
| 1037.502 | 2072.989 | 2072.988 | 95.51 | GAPGADGPAGAPGTPGPQGIAGQR | Deamidated (NQ); Oxidation (P) |
| 1037.993 | 2073.971 | 2073.972 | 74.96 | GAPGADGPAGAPGTPGPQGIAGQR | 2 Deamidated (NQ); Oxidation (P) |
| 1037.994 | 2073.973 | 2073.972 | 70.63 | GAPGADGPAGAPGTPGPQGIAGQR | 2 Deamidated (NQ); Oxidation (P) |
| 1045.497 | 2088.979 | 2088.983 | 50.28 | GAPGADGPAGAPGTPGPQGIAGQR | Deamidated (NQ); 2 Oxidation (P) |
| 1045.498 | 2088.981 | 2088.983 | 47.47 | GAPGADGPAGAPGTPGPQGIAGQR | Deamidated (NQ); 2 Oxidation (P) |
| 1045.499 | 2088.983 | 2088.983 | 45.28 | GAPGADGPAGAPGTPGPQGIAGQR | Deamidated (NQ); 2 Oxidation (P) |
| 1045.991 | 2089.967 | 2089.967 | 54.22 | GAPGADGPAGAPGTPGPQGIAGQR | 2 Deamidated (NQ); 2 Oxidation (P) |
| 1045.991 | 2089.967 | 2089.967 | 86.39 | GAPGADGPAGAPGTPGPQGIAGQR | 2 Deamidated (NQ); 2 Oxidation (P) |
| 1045.991 | 2089.967 | 2089.967 | 69.32 | GAPGADGPAGAPGTPGPQGIAGQR | 2 Deamidated (NQ); 2 Oxidation (P) |
| 1045.991 | 2089.967 | 2089.967 | 72.68 | GAPGADGPAGAPGTPGPQGIAGQR | 2 Deamidated (NQ); 2 Oxidation (P) |
| 1053.497 | 2104.979 | 2104.977 | 56.94 | GAPGADGPAGAPGTPGPQGIAGQR | Deamidated (NQ); 3 Oxidation (P) |
| 1053.497 | 2104.979 | 2104.977 | 54.12 | GAPGADGPAGAPGTPGPQGIAGQR | Deamidated (NQ); 3 Oxidation (P) |
| 1053.989 | 2105.963 | 2105.961 | 69.51 | GAPGADGPAGAPGTPGPQGIAGQR | 2 Deamidated (NQ); 3 Oxidation (P) |
| 1053.989 | 2105.963 | 2105.961 | 57.67 | GAPGADGPAGAPGTPGPQGIAGQR | 2 Deamidated (NQ); 3 Oxidation (P) |
| 1067.988 | 2133.961 | 2133.96 | 42.94 | GEPGPPGPAGFAGPPGADGQPGAK | Deamidated (NQ); 3 Oxidation (P) |
| 1077.035 | 2152.055 | 2152.055 | 62.35 | GETGPAGPPGAPGAPGAPGPVGPAGK | 2 Oxidation (P) |
| 1077.036 | 2152.057 | 2152.055 | 64.06 | GETGPAGPPGAPGAPGAPGPVGPAGK | 2 Oxidation (P) |
| 1085.033 | 2168.051 | 2168.05 | 73.13 | GETGPAGPPGAPGAPGAPGPVGPAGK | 3 Oxidation (P) |
| 1085.033 | 2168.051 | 2168.05 | 55.2 | GETGPAGPPGAPGAPGAPGPVGPAGK | 3 Oxidation (P) |
| 1085.033 | 2168.051 | 2168.05 | 40.89 | GETGPAGPPGAPGAPGAPGPVGPAGK | 3 Oxidation (P) |
| 723.6911 | 2168.052 | 2168.05 | 41.46 | GETGPAGPPGAPGAPGAPGPVGPAGK | 3 Oxidation (P) |
| 729.0222 | 2184.045 | 2184.045 | 45.58 | GETGPAGPPGAPGAPGAPGPVGPAGK | 4 Oxidation (P) |
| 1093.03 | 2184.045 | 2184.045 | 87.66 | GETGPAGPPGAPGAPGAPGPVGPAGK | 4 Oxidation (P) |
| 1093.031 | 2184.047 | 2184.045 | 81.72 | GETGPAGPPGAPGAPGAPGPVGPAGK | 4 Oxidation (P) |
| 1108.48 | 2214.945 | 2214.945 | 56.54 | GDAGAPGAPGSQGAPGLQGMPGER | Deamidated (NQ); 4 Oxidation (P); Oxidation (M) |
| 1108.972 | 2215.929 | 2215.929 | 71.05 | GDAGAPGAPGSQGAPGLQGMPGER | 2 Deamidated (NQ); 4 Oxidation (P); Oxidation (M) |
| 1108.972 | 2215.929 | 2215.929 | 58.84 | GDAGAPGAPGSQGAPGLQGMPGER | 2 Deamidated (NQ); 4 Oxidation (P); Oxidation (M) |
| 764.3856 | 2290.135 | 2290.134 | 41.9 | GDAGPPGPAGPAGPPGPIGNVGAPGPK | Oxidation (K); Oxidation (P) |
| 1146.566 | 2291.117 | 2291.118 | 76.5 | GDAGPPGPAGPAGPPGPIGNVGAPGPK | Deamidated (NQ); Oxidation (K); Oxidation (P) |
| 764.7136 | 2291.119 | 2291.118 | 46.54 | GDAGPPGPAGPAGPPGPIGNVGAPGPK | Deamidated (NQ); Oxidation (K); Oxidation (P) |
| 764.7136 | 2291.119 | 2291.118 | 57.24 | GDAGPPGPAGPAGPPGPIGNVGAPGPK | Deamidated (NQ); Oxidation (K); Oxidation (P) |
| 773.0159 | 2316.026 | 2316.026 | 40.62 | GEPGPPGPAGAAGPAGNPGADGQPGAK | Deamidated (NQ); 4 Oxidation (P) |
| 773.3441 | 2317.011 | 2317.01 | 42.5 | GEPGPPGPAGAAGPAGNPGADGQPGAK | 2 Deamidated (NQ); 4 Oxidation (P) |
| 1159.513 | 2317.011 | 2317.01 | 79.33 | GEPGPPGPAGAAGPAGNPGADGQPGAK | 2 Deamidated (NQ); 4 Oxidation (P) |
| 1159.513 | 2317.011 | 2317.01 | 66.15 | GEPGPPGPAGAAGPAGNPGADGQPGAK | 2 Deamidated (NQ); 4 Oxidation (P) |
| 1162.563 | 2323.111 | 2323.108 | 63.21 | GDAGPPGPAGPAGPPGPIGNVGAPGPK | Deamidated (NQ); 4 Oxidation (P) |
| 1162.563 | 2323.111 | 2323.108 | 58.17 | GDAGPPGPAGPAGPPGPIGNVGAPGPK | Deamidated (NQ); 4 Oxidation (P) |
| 829.7421 | 2486.205 | 2486.204 | 44.15 | GPPGSAGSPGKDGLNGLPGPIGPPGPR | Deamidated (NQ); 5 Oxidation (P) |
| 829.7446 | 2486.212 | 2486.204 | 42.59 | GPPGSAGSPGKDGLNGLPGPIGPPGPR | Deamidated (NQ); 5 Oxidation (P) |
| 838.4061 | 2512.197 | 2512.194 | 44.41 | GDRGETGPAGPPGAPGAPGAPGPVGPAGK | 4 Oxidation (P) |
| 838.4061 | 2512.197 | 2512.194 | 47.54 | GDRGETGPAGPPGAPGAPGAPGPVGPAGK | 4 Oxidation (P) |
| 845.0703 | 2532.189 | 2532.188 | 57.76 | GNDGATGAAGPPGPTGPAGPPGFPGAVGAK | Deamidated (NQ); 2 Oxidation (P) |
| 850.0739 | 2547.2 | 2547.199 | 47.02 | GNDGATGAAGPPGPTGPAGPPGFPGAVGAK | 3 Oxidation (P) |
| 850.0771 | 2547.21 | 2547.199 | 47.68 | GNDGATGAAGPPGPTGPAGPPGFPGAVGAK | 3 Oxidation (P) |
| 850.4022 | 2548.185 | 2548.183 | 52.6 | GNDGATGAAGPPGPTGPAGPPGFPGAVGAK | Deamidated (NQ); 3 Oxidation (P) |
| 850.4024 | 2548.185 | 2548.183 | 48.39 | GNDGATGAAGPPGPTGPAGPPGFPGAVGAK | Deamidated (NQ); 3 Oxidation (P) |
| 855.7332 | 2564.178 | 2564.178 | 49.93 | GNDGATGAAGPPGPTGPAGPPGFPGAVGAK | Deamidated (NQ); Oxidation (K); 3 Oxidation (P) |
| 902.7518 | 2705.234 | 2705.232 | 58.04 | GFSGLQGPPGPPGSPGEQGPSGASGPAGPR | Deamidated (NQ); 3 Oxidation (P) |
| 902.7518 | 2705.234 | 2705.232 | 52.1 | GFSGLQGPPGPPGSPGEQGPSGASGPAGPR | Deamidated (NQ); 3 Oxidation (P) |
| 1082.125 | 3243.353 | 3243.349 | 41.08 | GANGAPGNDGAKGDAGAPGAPGSQGAPGLQGMPGER | 4 Deamidated (NQ); 5 Oxidation (P); Oxidation (M) |

**HSB3**

| Serum albumin OS=Bos taurus GN=ALB PE=1 SV=4 | 111 |
| --- | --- |

| **pep_exp_mz** | **pep_exp_mr** | **pep_calc_mr** | **pep_end** | **pep_score** | **pep_seq** | **pep_var_mod** |
| --- | --- | --- | --- | --- | --- | --- |
| 582.319 | 1162.623 | 1162.623 | 75 | 53.29 | LVNELTEFAK | |
| 653.3618 | 1304.709 | 1304.709 | 412 | 53 | HLVDEPQNLIK | |
| 653.3619 | 1304.709 | 1304.709 | 412 | 52.75 | HLVDEPQNLIK | |

**TSH**

| Collagen alpha-1(I) chain OS=Bos taurus GN=COL1A1 PE=1 SV=3 | 6326 |
| --- | --- |

| **pep_exp_mz** | **pep_exp_mr** | **pep_calc_mr** | **pep_score** | **pep_seq** | **pep_var_mod** |
| --- | --- | --- | --- | --- | --- |
| 392.2215 | 782.4284 | 782.4286 | 54.11 | GAAGLPGPK | Oxidation (K) |
| 426.2165 | 850.4184 | 850.4185 | 40.81 | GFSGLDGAK | |
| 426.2166 | 850.4186 | 850.4185 | 41.84 | GFSGLDGAK | |
| 449.7586 | 897.5026 | 897.5032 | 48.2 | GVVGLPGQR | Oxidation (P) |
| 449.7586 | 897.5026 | 897.5032 | 48.12 | GVVGLPGQR | Oxidation (P) |
| 450.2507 | 898.4868 | 898.4872 | 41.17 | GVVGLPGQR | Deamidated (NQ); Oxidation (P) |
| 450.2508 | 898.487 | 898.4872 | 45.26 | GVVGLPGQR | Deamidated (NQ); Oxidation (P) |
| 544.7722 | 1087.53 | 1087.53 | 52.34 | GFPGADGVAGPK | Oxidation (P) |
| 544.7723 | 1087.53 | 1087.53 | 54.41 | GFPGADGVAGPK | Oxidation (P) |
| 553.7833 | 1105.552 | 1105.552 | 42.22 | GVQGPPGPAGPR | Deamidated (NQ); Oxidation (P) |
| 589.2873 | 1176.56 | 1176.56 | 57.03 | GQAGVMGFPGPK | Oxidation (M); Oxidation (P) |
| 589.2874 | 1176.56 | 1176.56 | 45.93 | GQAGVMGFPGPK | Oxidation (M); Oxidation (P) |
| 589.2875 | 1176.56 | 1176.56 | 45.78 | GQAGVMGFPGPK | Oxidation (M); Oxidation (P) |
| 589.2875 | 1176.56 | 1176.56 | 51.74 | GQAGVMGFPGPK | Oxidation (M); Oxidation (P) |
| 589.7792 | 1177.544 | 1177.544 | 57.7 | GQAGVMGFPGPK | Deamidated (NQ); Oxidation (M); Oxidation (P) |
| 589.7793 | 1177.544 | 1177.544 | 68.46 | GQAGVMGFPGPK | Deamidated (NQ); Oxidation (M); Oxidation (P) |
| 589.7795 | 1177.544 | 1177.544 | 61.57 | GQAGVMGFPGPK | Deamidated (NQ); Oxidation (M); Oxidation (P) |
| 589.7795 | 1177.544 | 1177.544 | 61.63 | GQAGVMGFPGPK | Deamidated (NQ); Oxidation (M); Oxidation (P) |
| 596.8196 | 1191.625 | 1191.625 | 44.8 | GVPGPPGAVGPAGK | 2 Oxidation (P) |
| 596.8198 | 1191.625 | 1191.625 | 49.03 | GVPGPPGAVGPAGK | 2 Oxidation (P) |
| 597.2848 | 1192.555 | 1192.555 | 46.73 | GQAGVMGFPGPK | Oxidation (K); Oxidation (M); Oxidation (P) |
| 597.285 | 1192.555 | 1192.555 | 40.37 | GQAGVMGFPGPK | Oxidation (M); 2 Oxidation (P) |
| 597.7764 | 1193.538 | 1193.539 | 47.53 | GQAGVMGFPGPK | Deamidated (NQ); Oxidation (K); Oxidation (M); Oxidation (P) |
| 597.7766 | 1193.539 | 1193.539 | 47.73 | GQAGVMGFPGPK | Deamidated (NQ); Oxidation (K); Oxidation (M); Oxidation (P) |
| 597.7766 | 1193.539 | 1193.539 | 50.25 | GQAGVMGFPGPK | Deamidated (NQ); Oxidation (K); Oxidation (M); Oxidation (P) |
| 597.7767 | 1193.539 | 1193.539 | 49.66 | GQAGVMGFPGPK | Deamidated (NQ); Oxidation (K); Oxidation (M); Oxidation (P) |
| 656.8306 | 1311.647 | 1311.646 | 42.02 | GFPGLPGPSGEPGK | Oxidation (P) |
| 664.8279 | 1327.641 | 1327.641 | 52.92 | GFPGLPGPSGEPGK | 2 Oxidation (P) |
| 664.8279 | 1327.641 | 1327.641 | 42.25 | GFPGLPGPSGEPGK | 2 Oxidation (P) |
| 664.828 | 1327.641 | 1327.641 | 64.02 | GFPGLPGPSGEPGK | 2 Oxidation (P) |
| 664.828 | 1327.641 | 1327.641 | 56.43 | GFPGLPGPSGEPGK | 2 Oxidation (P) |
| 664.828 | 1327.641 | 1327.641 | 48.23 | GFPGLPGPSGEPGK | 2 Oxidation (P) |
| 664.8281 | 1327.642 | 1327.641 | 62.28 | GFPGLPGPSGEPGK | 2 Oxidation (P) |
| 664.8281 | 1327.642 | 1327.641 | 40.05 | GFPGLPGPSGEPGK | 2 Oxidation (P) |
| 664.8281 | 1327.642 | 1327.641 | 41.9 | GFPGLPGPSGEPGK | 2 Oxidation (P) |
| 664.8281 | 1327.642 | 1327.641 | 41.55 | GFPGLPGPSGEPGK | 2 Oxidation (P) |
| 664.8281 | 1327.642 | 1327.641 | 43.87 | GFPGLPGPSGEPGK | 2 Oxidation (P) |
| 672.8254 | 1343.636 | 1343.636 | 52.85 | GFPGLPGPSGEPGK | 3 Oxidation (P) |
| 672.8254 | 1343.636 | 1343.636 | 62.68 | GFPGLPGPSGEPGK | 3 Oxidation (P) |
| 672.8254 | 1343.636 | 1343.636 | 62.98 | GFPGLPGPSGEPGK | 3 Oxidation (P) |
| 672.8255 | 1343.636 | 1343.636 | 55.33 | GFPGLPGPSGEPGK | 3 Oxidation (P) |
| 718.3439 | 1434.673 | 1434.674 | 55.19 | GEPGPAGLPGPPGER | 3 Oxidation (P) |
| 718.3441 | 1434.674 | 1434.674 | 58.62 | GEPGPAGLPGPPGER | 3 Oxidation (P) |
| 718.3447 | 1434.675 | 1434.674 | 47.29 | GEPGPAGLPGPPGER | 3 Oxidation (P) |
| 718.3449 | 1434.675 | 1434.674 | 61.24 | GEPGPAGLPGPPGER | 3 Oxidation (P) |
| 718.3451 | 1434.676 | 1434.674 | 49.82 | GEPGPAGLPGPPGER | 3 Oxidation (P) |
| 718.3451 | 1434.676 | 1434.674 | 63.9 | GEPGPAGLPGPPGER | 3 Oxidation (P) |
| 730.3488 | 1458.683 | 1458.685 | 50.08 | GSAGPPGATGFPGAAGR | 2 Oxidation (P) |
| 730.3495 | 1458.684 | 1458.685 | 76.43 | GSAGPPGATGFPGAAGR | 2 Oxidation (P) |
| 730.3497 | 1458.685 | 1458.685 | 54.72 | GSAGPPGATGFPGAAGR | 2 Oxidation (P) |
| 730.3499 | 1458.685 | 1458.685 | 52.83 | GSAGPPGATGFPGAAGR | 2 Oxidation (P) |
| 730.3499 | 1458.685 | 1458.685 | 76.39 | GSAGPPGATGFPGAAGR | 2 Oxidation (P) |
| 730.35 | 1458.685 | 1458.685 | 80.29 | GSAGPPGATGFPGAAGR | 2 Oxidation (P) |
| 730.35 | 1458.685 | 1458.685 | 95.63 | GSAGPPGATGFPGAAGR | 2 Oxidation (P) |
| 730.35 | 1458.685 | 1458.685 | 41.63 | GSAGPPGATGFPGAAGR | 2 Oxidation (P) |
| 730.35 | 1458.685 | 1458.685 | 41.26 | GSAGPPGATGFPGAAGR | 2 Oxidation (P) |
| 730.3502 | 1458.686 | 1458.685 | 112.4 | GSAGPPGATGFPGAAGR | 2 Oxidation (P) |
| 730.3502 | 1458.686 | 1458.685 | 40.45 | GSAGPPGATGFPGAAGR | 2 Oxidation (P) |
| 730.3502 | 1458.686 | 1458.685 | 69.55 | GSAGPPGATGFPGAAGR | 2 Oxidation (P) |
| 730.3502 | 1458.686 | 1458.685 | 47.38 | GSAGPPGATGFPGAAGR | 2 Oxidation (P) |
| 730.3502 | 1458.686 | 1458.685 | 88.59 | GSAGPPGATGFPGAAGR | 2 Oxidation (P) |
| 730.3502 | 1458.686 | 1458.685 | 42.35 | GSAGPPGATGFPGAAGR | 2 Oxidation (P) |
| 730.3502 | 1458.686 | 1458.685 | 62.49 | GSAGPPGATGFPGAAGR | 2 Oxidation (P) |
| 730.3502 | 1458.686 | 1458.685 | 69.25 | GSAGPPGATGFPGAAGR | 2 Oxidation (P) |
| 730.3502 | 1458.686 | 1458.685 | 46.64 | GSAGPPGATGFPGAAGR | 2 Oxidation (P) |
| 730.3503 | 1458.686 | 1458.685 | 84.39 | GSAGPPGATGFPGAAGR | 2 Oxidation (P) |
| 730.3503 | 1458.686 | 1458.685 | 69.85 | GSAGPPGATGFPGAAGR | 2 Oxidation (P) |
| 730.3503 | 1458.686 | 1458.685 | 86.88 | GSAGPPGATGFPGAAGR | 2 Oxidation (P) |
| 730.3504 | 1458.686 | 1458.685 | 49.64 | GSAGPPGATGFPGAAGR | 2 Oxidation (P) |
| 730.3506 | 1458.687 | 1458.685 | 49.15 | GSAGPPGATGFPGAAGR | 2 Oxidation (P) |
| 730.3506 | 1458.687 | 1458.685 | 40.32 | GSAGPPGATGFPGAAGR | 2 Oxidation (P) |
| 730.3507 | 1458.687 | 1458.685 | 54.53 | GSAGPPGATGFPGAAGR | 2 Oxidation (P) |
| 730.3507 | 1458.687 | 1458.685 | 58.16 | GSAGPPGATGFPGAAGR | 2 Oxidation (P) |
| 730.3508 | 1458.687 | 1458.685 | 52.28 | GSAGPPGATGFPGAAGR | 2 Oxidation (P) |
| 730.3508 | 1458.687 | 1458.685 | 42.48 | GSAGPPGATGFPGAAGR | 2 Oxidation (P) |
| 745.86 | 1489.705 | 1489.705 | 45.73 | PGEVGPPGPPGPAGEK | 3 Oxidation (P) |
| 745.86 | 1489.705 | 1489.705 | 40.19 | PGEVGPPGPPGPAGEK | 3 Oxidation (P) |
| 520.9426 | 1559.806 | 1559.806 | 53.94 | GETGPAGPAGPIGPVGAR | |
| 520.9426 | 1559.806 | 1559.806 | 55.96 | GETGPAGPAGPIGPVGAR | |
| 780.9105 | 1559.806 | 1559.806 | 71.5 | GETGPAGPAGPIGPVGAR | |
| 780.9106 | 1559.807 | 1559.806 | 61.79 | GETGPAGPAGPIGPVGAR | |
| 780.9107 | 1559.807 | 1559.806 | 66.85 | GETGPAGPAGPIGPVGAR | |
| 780.9108 | 1559.807 | 1559.806 | 76.26 | GETGPAGPAGPIGPVGAR | |
| 781.4025 | 1560.79 | 1560.79 | 44.86 | DGLNGLPGPIGPPGPR | 3 Oxidation (P) |
| 781.4026 | 1560.791 | 1560.79 | 43 | DGLNGLPGPIGPPGPR | 3 Oxidation (P) |
| 781.8939 | 1561.773 | 1561.774 | 44.72 | DGLNGLPGPIGPPGPR | Deamidated (NQ); 3 Oxidation (P) |
| 781.894 | 1561.773 | 1561.774 | 49.96 | DGLNGLPGPIGPPGPR | Deamidated (NQ); 3 Oxidation (P) |
| 781.8942 | 1561.774 | 1561.774 | 41.9 | DGLNGLPGPIGPPGPR | Deamidated (NQ); 3 Oxidation (P) |
| 781.8943 | 1561.774 | 1561.774 | 59.49 | DGLNGLPGPIGPPGPR | Deamidated (NQ); 3 Oxidation (P) |
| 781.8944 | 1561.774 | 1561.774 | 56.34 | DGLNGLPGPIGPPGPR | Deamidated (NQ); 3 Oxidation (P) |
| 781.8944 | 1561.774 | 1561.774 | 52.32 | DGLNGLPGPIGPPGPR | Deamidated (NQ); 3 Oxidation (P) |
| 781.8945 | 1561.774 | 1561.774 | 46.94 | DGLNGLPGPIGPPGPR | Deamidated (NQ); 3 Oxidation (P) |
| 781.8945 | 1561.774 | 1561.774 | 62.35 | DGLNGLPGPIGPPGPR | Deamidated (NQ); 3 Oxidation (P) |
| 781.8946 | 1561.775 | 1561.774 | 41.57 | DGLNGLPGPIGPPGPR | Deamidated (NQ); 3 Oxidation (P) |
| 781.8946 | 1561.775 | 1561.774 | 54.11 | DGLNGLPGPIGPPGPR | Deamidated (NQ); 3 Oxidation (P) |
| 781.8946 | 1561.775 | 1561.774 | 42.42 | DGLNGLPGPIGPPGPR | Deamidated (NQ); 3 Oxidation (P) |
| 781.8947 | 1561.775 | 1561.774 | 62.33 | DGLNGLPGPIGPPGPR | Deamidated (NQ); 3 Oxidation (P) |
| 781.8947 | 1561.775 | 1561.774 | 66.61 | DGLNGLPGPIGPPGPR | Deamidated (NQ); 3 Oxidation (P) |
| 781.8947 | 1561.775 | 1561.774 | 44.97 | DGLNGLPGPIGPPGPR | Deamidated (NQ); 3 Oxidation (P) |
| 781.8947 | 1561.775 | 1561.774 | 41.31 | DGLNGLPGPIGPPGPR | Deamidated (NQ); 3 Oxidation (P) |
| 781.8953 | 1561.776 | 1561.774 | 40.55 | DGLNGLPGPIGPPGPR | Deamidated (NQ); 3 Oxidation (P) |
| 788.908 | 1575.801 | 1575.801 | 47.83 | GETGPAGPAGPIGPVGAR | Oxidation (P) |
| 788.9084 | 1575.802 | 1575.801 | 47.36 | GETGPAGPAGPIGPVGAR | Oxidation (P) |
| 793.8811 | 1585.748 | 1585.749 | 41.19 | GANGAPGIAGAPGFPGAR | Deamidated (NQ); 3 Oxidation (P) |
| 793.8813 | 1585.748 | 1585.749 | 40.98 | GANGAPGIAGAPGFPGAR | Deamidated (NQ); 3 Oxidation (P) |
| 793.8815 | 1585.748 | 1585.749 | 52.42 | GANGAPGIAGAPGFPGAR | Deamidated (NQ); 3 Oxidation (P) |
| 793.8817 | 1585.749 | 1585.749 | 42.79 | GANGAPGIAGAPGFPGAR | Deamidated (NQ); 3 Oxidation (P) |
| 793.8819 | 1585.749 | 1585.749 | 61.68 | GANGAPGIAGAPGFPGAR | Deamidated (NQ); 3 Oxidation (P) |
| 793.882 | 1585.749 | 1585.749 | 76.55 | GANGAPGIAGAPGFPGAR | Deamidated (NQ); 3 Oxidation (P) |
| 793.8821 | 1585.75 | 1585.749 | 68.62 | GANGAPGIAGAPGFPGAR | Deamidated (NQ); 3 Oxidation (P) |
| 793.8821 | 1585.75 | 1585.749 | 61.03 | GANGAPGIAGAPGFPGAR | Deamidated (NQ); 3 Oxidation (P) |
| 793.8822 | 1585.75 | 1585.749 | 48.88 | GANGAPGIAGAPGFPGAR | Deamidated (NQ); 3 Oxidation (P) |
| 793.8824 | 1585.75 | 1585.749 | 44.64 | GANGAPGIAGAPGFPGAR | Deamidated (NQ); 3 Oxidation (P) |
| 793.8824 | 1585.75 | 1585.749 | 44.3 | GANGAPGIAGAPGFPGAR | Deamidated (NQ); 3 Oxidation (P) |
| 808.3951 | 1614.776 | 1614.775 | 54.9 | GGPGSRGFPGADGVAGPK | 2 Oxidation (P) |
| 808.3954 | 1614.776 | 1614.775 | 69.69 | GGPGSRGFPGADGVAGPK | 2 Oxidation (P) |
| 808.3958 | 1614.777 | 1614.775 | 52.16 | GGPGSRGFPGADGVAGPK | 2 Oxidation (P) |
| 845.8927 | 1689.771 | 1689.771 | 68.08 | DGEAGAQGPPGPAGPAGER | |
| 845.8931 | 1689.772 | 1689.771 | 88.95 | DGEAGAQGPPGPAGPAGER | |
| 845.8931 | 1689.772 | 1689.771 | 71.96 | DGEAGAQGPPGPAGPAGER | |
| 845.8933 | 1689.772 | 1689.771 | 75.78 | DGEAGAQGPPGPAGPAGER | |
| 846.3846 | 1690.755 | 1690.755 | 85.84 | DGEAGAQGPPGPAGPAGER | Deamidated (NQ) |
| 846.3847 | 1690.755 | 1690.755 | 82.99 | DGEAGAQGPPGPAGPAGER | Deamidated (NQ) |
| 853.8906 | 1705.767 | 1705.766 | 56.97 | DGEAGAQGPPGPAGPAGER | Oxidation (P) |
| 853.8906 | 1705.767 | 1705.766 | 71.05 | DGEAGAQGPPGPAGPAGER | Oxidation (P) |
| 853.8907 | 1705.767 | 1705.766 | 46.33 | DGEAGAQGPPGPAGPAGER | Oxidation (P) |
| 853.8909 | 1705.767 | 1705.766 | 81.57 | DGEAGAQGPPGPAGPAGER | Oxidation (P) |
| 854.3821 | 1706.75 | 1706.75 | 57.63 | DGEAGAQGPPGPAGPAGER | Deamidated (NQ); Oxidation (P) |
| 854.3823 | 1706.75 | 1706.75 | 106.12 | DGEAGAQGPPGPAGPAGER | Deamidated (NQ); Oxidation (P) |
| 854.3823 | 1706.75 | 1706.75 | 45.01 | DGEAGAQGPPGPAGPAGER | Deamidated (NQ); Oxidation (P) |
| 854.3824 | 1706.75 | 1706.75 | 107.1 | DGEAGAQGPPGPAGPAGER | Deamidated (NQ); Oxidation (P) |
| 581.9554 | 1742.844 | 1742.845 | 40.64 | GARGSAGPPGATGFPGAAGR | 2 Oxidation (P) |
| 581.9556 | 1742.845 | 1742.845 | 54.14 | GARGSAGPPGATGFPGAAGR | 2 Oxidation (P) |
| 581.9559 | 1742.846 | 1742.845 | 85.25 | GARGSAGPPGATGFPGAAGR | 2 Oxidation (P) |
| 872.4309 | 1742.847 | 1742.845 | 61.35 | GARGSAGPPGATGFPGAAGR | 2 Oxidation (P) |
| 872.4314 | 1742.848 | 1742.845 | 43.45 | GARGSAGPPGATGFPGAAGR | 2 Oxidation (P) |
| 907.4394 | 1812.864 | 1812.864 | 41.25 | VGPPGPSGNAGPPGPPGPAGK | Deamidated (NQ); Oxidation (K); 2 Oxidation (P) |
| 907.4404 | 1812.866 | 1812.864 | 47.84 | VGPPGPSGNAGPPGPPGPAGK | Deamidated (NQ); Oxidation (K); 2 Oxidation (P) |
| 908.9363 | 1815.858 | 1815.857 | 81.2 | GPPGPMGPPGLAGPPGESGR | Oxidation (M); Oxidation (P) |
| 908.9365 | 1815.858 | 1815.857 | 93.23 | GPPGPMGPPGLAGPPGESGR | Oxidation (M); Oxidation (P) |
| 908.937 | 1815.859 | 1815.857 | 60.82 | GPPGPMGPPGLAGPPGESGR | Oxidation (M); Oxidation (P) |
| 908.9371 | 1815.86 | 1815.857 | 53.23 | GPPGPMGPPGLAGPPGESGR | Oxidation (M); Oxidation (P) |
| 915.4375 | 1828.86 | 1828.859 | 58.34 | VGPPGPSGNAGPPGPPGPAGK | Deamidated (NQ); Oxidation (K); 3 Oxidation (P) |
| 916.9333 | 1831.852 | 1831.852 | 59.82 | GPPGPMGPPGLAGPPGESGR | Oxidation (M); 2 Oxidation (P) |
| 611.6248 | 1831.853 | 1831.852 | 60.69 | GPPGPMGPPGLAGPPGESGR | Oxidation (M); 2 Oxidation (P) |
| 611.6248 | 1831.853 | 1831.852 | 50.58 | GPPGPMGPPGLAGPPGESGR | Oxidation (M); 2 Oxidation (P) |
| 916.9338 | 1831.853 | 1831.852 | 75.86 | GPPGPMGPPGLAGPPGESGR | Oxidation (M); 2 Oxidation (P) |
| 916.9338 | 1831.853 | 1831.852 | 69.72 | GPPGPMGPPGLAGPPGESGR | Oxidation (M); 2 Oxidation (P) |
| 916.9339 | 1831.853 | 1831.852 | 82.45 | GPPGPMGPPGLAGPPGESGR | Oxidation (M); 2 Oxidation (P) |
| 916.9341 | 1831.854 | 1831.852 | 61.12 | GPPGPMGPPGLAGPPGESGR | 3 Oxidation (P) |
| 916.9341 | 1831.854 | 1831.852 | 70.55 | GPPGPMGPPGLAGPPGESGR | Oxidation (M); 2 Oxidation (P) |
| 916.9341 | 1831.854 | 1831.852 | 70.27 | GPPGPMGPPGLAGPPGESGR | Oxidation (M); 2 Oxidation (P) |
| 916.9342 | 1831.854 | 1831.852 | 66.23 | GPPGPMGPPGLAGPPGESGR | 3 Oxidation (P) |
| 924.931 | 1847.847 | 1847.847 | 41.41 | GPPGPMGPPGLAGPPGESGR | Oxidation (M); 3 Oxidation (P) |
| 924.9312 | 1847.848 | 1847.847 | 41.74 | GPPGPMGPPGLAGPPGESGR | Oxidation (M); 3 Oxidation (P) |
| 924.9319 | 1847.849 | 1847.847 | 46 | GPPGPMGPPGLAGPPGESGR | Oxidation (M); 3 Oxidation (P) |
| 932.4398 | 1862.865 | 1862.865 | 63.3 | GEPGPTGIQGPPGPAGEEGK | 2 Oxidation (P) |
| 932.44 | 1862.865 | 1862.865 | 75.58 | GEPGPTGIQGPPGPAGEEGK | 2 Oxidation (P) |
| 932.44 | 1862.865 | 1862.865 | 73.19 | GEPGPTGIQGPPGPAGEEGK | 2 Oxidation (P) |
| 932.4401 | 1862.866 | 1862.865 | 68.27 | GEPGPTGIQGPPGPAGEEGK | 2 Oxidation (P) |
| 932.9289 | 1863.843 | 1863.842 | 46.33 | GPPGPMGPPGLAGPPGESGR | 5 Oxidation (P) |
| 932.9293 | 1863.844 | 1863.842 | 43.74 | GPPGPMGPPGLAGPPGESGR | 5 Oxidation (P) |
| 932.9313 | 1863.848 | 1863.849 | 71.74 | GEPGPTGIQGPPGPAGEEGK | Deamidated (NQ); 2 Oxidation (P) |
| 932.9318 | 1863.849 | 1863.849 | 59.31 | GEPGPTGIQGPPGPAGEEGK | Deamidated (NQ); 2 Oxidation (P) |
| 988.4977 | 1974.981 | 1974.987 | 42.64 | SGDRGETGPAGPAGPIGPVGAR | |
| 988.5013 | 1974.988 | 1974.987 | 42.04 | SGDRGETGPAGPAGPIGPVGAR | |
| 988.5018 | 1974.989 | 1974.987 | 65.66 | SGDRGETGPAGPAGPIGPVGAR | |
| 988.5021 | 1974.99 | 1974.987 | 69.84 | SGDRGETGPAGPAGPIGPVGAR | |
| 988.5023 | 1974.99 | 1974.987 | 62.37 | SGDRGETGPAGPAGPIGPVGAR | |
| 673.996 | 2018.966 | 2018.966 | 42.35 | GEPGPTGIQGPPGPAGEEGKR | 2 Oxidation (P) |
| 1010.491 | 2018.967 | 2018.966 | 41.88 | GEPGPTGIQGPPGPAGEEGKR | 2 Oxidation (P) |
| 1010.491 | 2018.967 | 2018.966 | 49.9 | GEPGPTGIQGPPGPAGEEGKR | 2 Oxidation (P) |
| 1010.492 | 2018.969 | 2018.966 | 55.4 | GEPGPTGIQGPPGPAGEEGKR | 2 Oxidation (P) |
| 1010.492 | 2018.969 | 2018.966 | 44.18 | GEPGPTGIQGPPGPAGEEGKR | 2 Oxidation (P) |
| 674.3241 | 2019.951 | 2019.95 | 47.15 | GEPGPTGIQGPPGPAGEEGKR | Deamidated (NQ); 2 Oxidation (P) |
| 1010.983 | 2019.951 | 2019.95 | 57.48 | GEPGPTGIQGPPGPAGEEGKR | Deamidated (NQ); 2 Oxidation (P) |
| 1010.983 | 2019.951 | 2019.95 | 75.59 | GEPGPTGIQGPPGPAGEEGKR | Deamidated (NQ); 2 Oxidation (P) |
| 691.6754 | 2072.004 | 2072.004 | 43.55 | GAPGADGPAGAPGTPGPQGIAGQR | Oxidation (P) |
| 1037.01 | 2072.005 | 2072.004 | 73.67 | GAPGADGPAGAPGTPGPQGIAGQR | Oxidation (P) |
| 1037.01 | 2072.005 | 2072.004 | 68.7 | GAPGADGPAGAPGTPGPQGIAGQR | Oxidation (P) |
| 692.0032 | 2072.988 | 2072.988 | 46.88 | GAPGADGPAGAPGTPGPQGIAGQR | Deamidated (NQ); Oxidation (P) |
| 1037.502 | 2072.989 | 2072.988 | 73.42 | GAPGADGPAGAPGTPGPQGIAGQR | Deamidated (NQ); Oxidation (P) |
| 1037.503 | 2072.991 | 2072.988 | 83.6 | GAPGADGPAGAPGTPGPQGIAGQR | Deamidated (NQ); Oxidation (P) |
| 1037.503 | 2072.991 | 2072.988 | 86.7 | GAPGADGPAGAPGTPGPQGIAGQR | Deamidated (NQ); Oxidation (P) |
| 1037.993 | 2073.971 | 2073.972 | 83.47 | GAPGADGPAGAPGTPGPQGIAGQR | 2 Deamidated (NQ); Oxidation (P) |
| 1045.008 | 2088.001 | 2087.999 | 46.7 | GAPGADGPAGAPGTPGPQGIAGQR | 2 Oxidation (P) |
| 1045.008 | 2088.001 | 2087.999 | 46.03 | GAPGADGPAGAPGTPGPQGIAGQR | 2 Oxidation (P) |
| 1045.008 | 2088.001 | 2087.999 | 58.22 | GAPGADGPAGAPGTPGPQGIAGQR | 2 Oxidation (P) |
| 1045.008 | 2088.001 | 2087.999 | 65.5 | GAPGADGPAGAPGTPGPQGIAGQR | 2 Oxidation (P) |
| 697.3347 | 2088.982 | 2088.983 | 40.88 | GAPGADGPAGAPGTPGPQGIAGQR | Deamidated (NQ); 2 Oxidation (P) |
| 1045.499 | 2088.983 | 2088.983 | 56.97 | GAPGADGPAGAPGTPGPQGIAGQR | Deamidated (NQ); 2 Oxidation (P) |
| 1045.5 | 2088.985 | 2088.983 | 40.39 | GAPGADGPAGAPGTPGPQGIAGQR | Deamidated (NQ); 2 Oxidation (P) |
| 697.6628 | 2089.967 | 2089.967 | 49.78 | GAPGADGPAGAPGTPGPQGIAGQR | 2 Deamidated (NQ); 2 Oxidation (P) |
| 1045.992 | 2089.969 | 2089.967 | 53.66 | GAPGADGPAGAPGTPGPQGIAGQR | 2 Deamidated (NQ); 2 Oxidation (P) |
| 1045.992 | 2089.969 | 2089.967 | 78.86 | GAPGADGPAGAPGTPGPQGIAGQR | 2 Deamidated (NQ); 2 Oxidation (P) |
| 1045.992 | 2089.969 | 2089.967 | 71.61 | GAPGADGPAGAPGTPGPQGIAGQR | 2 Deamidated (NQ); 2 Oxidation (P) |
| 1045.993 | 2089.971 | 2089.967 | 47.04 | GAPGADGPAGAPGTPGPQGIAGQR | 2 Deamidated (NQ); 2 Oxidation (P) |
| 1053.497 | 2104.979 | 2104.977 | 51.95 | GAPGADGPAGAPGTPGPQGIAGQR | Deamidated (NQ); 3 Oxidation (P) |
| 1053.988 | 2105.961 | 2105.961 | 69.53 | GAPGADGPAGAPGTPGPQGIAGQR | 2 Deamidated (NQ); 3 Oxidation (P) |
| 1053.989 | 2105.963 | 2105.961 | 74.77 | GAPGADGPAGAPGTPGPQGIAGQR | 2 Deamidated (NQ); 3 Oxidation (P) |
| 1067.988 | 2133.961 | 2133.96 | 50.34 | GEPGPPGPAGFAGPPGADGQPGAK | Deamidated (NQ); 3 Oxidation (P) |
| 1067.988 | 2133.961 | 2133.96 | 44.32 | GEPGPPGPAGFAGPPGADGQPGAK | Deamidated (NQ); 3 Oxidation (P) |
| 1077.035 | 2152.055 | 2152.055 | 60.04 | GETGPAGPPGAPGAPGAPGPVGPAGK | 2 Oxidation (P) |
| 1077.036 | 2152.057 | 2152.055 | 66.92 | GETGPAGPPGAPGAPGAPGPVGPAGK | 2 Oxidation (P) |
| 1085.033 | 2168.051 | 2168.05 | 66.56 | GETGPAGPPGAPGAPGAPGPVGPAGK | 3 Oxidation (P) |
| 1085.033 | 2168.051 | 2168.05 | 48.49 | GETGPAGPPGAPGAPGAPGPVGPAGK | 3 Oxidation (P) |
| 1085.033 | 2168.051 | 2168.05 | 46.08 | GETGPAGPPGAPGAPGAPGPVGPAGK | 3 Oxidation (P) |
| 1085.033 | 2168.051 | 2168.05 | 55.04 | GETGPAGPPGAPGAPGAPGPVGPAGK | 3 Oxidation (P) |
| 723.6913 | 2168.052 | 2168.05 | 42.9 | GETGPAGPPGAPGAPGAPGPVGPAGK | 3 Oxidation (P) |
| 1093.03 | 2184.045 | 2184.045 | 70.63 | GETGPAGPPGAPGAPGAPGPVGPAGK | 4 Oxidation (P) |
| 1093.031 | 2184.047 | 2184.045 | 52.9 | GETGPAGPPGAPGAPGAPGPVGPAGK | 4 Oxidation (P) |
| 1107.988 | 2213.961 | 2213.961 | 57.72 | GDAGAPGAPGSQGAPGLQGMPGER | Oxidation (M); 4 Oxidation (P) |
| 1107.989 | 2213.963 | 2213.961 | 70.53 | GDAGAPGAPGSQGAPGLQGMPGER | Oxidation (M); 4 Oxidation (P) |
| 1108.48 | 2214.945 | 2214.945 | 70.86 | GDAGAPGAPGSQGAPGLQGMPGER | Deamidated (NQ); Oxidation (M); 4 Oxidation (P) |
| 1108.481 | 2214.947 | 2214.945 | 73.48 | GDAGAPGAPGSQGAPGLQGMPGER | Deamidated (NQ); Oxidation (M); 4 Oxidation (P) |
| 1108.972 | 2215.929 | 2215.929 | 50.71 | GDAGAPGAPGSQGAPGLQGMPGER | 2 Deamidated (NQ); Oxidation (M); 4 Oxidation (P) |
| 1108.972 | 2215.929 | 2215.929 | 64.47 | GDAGAPGAPGSQGAPGLQGMPGER | 2 Deamidated (NQ); Oxidation (M); 4 Oxidation (P) |
| 764.3857 | 2290.135 | 2290.134 | 50.14 | GDAGPPGPAGPAGPPGPIGNVGAPGPK | Oxidation (K); Oxidation (P) |
| 764.3862 | 2290.137 | 2290.134 | 49.64 | GDAGPPGPAGPAGPPGPIGNVGAPGPK | Oxidation (K); Oxidation (P) |
| 1146.076 | 2290.137 | 2290.134 | 86.49 | GDAGPPGPAGPAGPPGPIGNVGAPGPK | Oxidation (K); Oxidation (P) |
| 1146.076 | 2290.137 | 2290.134 | 84.11 | GDAGPPGPAGPAGPPGPIGNVGAPGPK | Oxidation (K); Oxidation (P) |
| 764.7137 | 2291.119 | 2291.118 | 79.05 | GDAGPPGPAGPAGPPGPIGNVGAPGPK | Deamidated (NQ); Oxidation (K); Oxidation (P) |
| 764.714 | 2291.12 | 2291.118 | 71.51 | GDAGPPGPAGPAGPPGPIGNVGAPGPK | Deamidated (NQ); Oxidation (K); Oxidation (P) |
| 1146.568 | 2291.121 | 2291.118 | 91.19 | GDAGPPGPAGPAGPPGPIGNVGAPGPK | Deamidated (NQ); Oxidation (K); Oxidation (P) |
| 1146.568 | 2291.121 | 2291.118 | 42.58 | GDAGPPGPAGPAGPPGPIGNVGAPGPK | Deamidated (NQ); Oxidation (K); Oxidation (P) |
| 1154.564 | 2307.113 | 2307.113 | 68.22 | GDAGPPGPAGPAGPPGPIGNVGAPGPK | Deamidated (NQ); Oxidation (K); 2 Oxidation (P) |
| 1159.02 | 2316.025 | 2316.026 | 57.77 | GEPGPPGPAGAAGPAGNPGADGQPGAK | Deamidated (NQ); 4 Oxidation (P) |
| 773.016 | 2316.026 | 2316.026 | 54.7 | GEPGPPGPAGAAGPAGNPGADGQPGAK | Deamidated (NQ); 4 Oxidation (P) |
| 773.0161 | 2316.027 | 2316.026 | 42.26 | GEPGPPGPAGAAGPAGNPGADGQPGAK | Deamidated (NQ); 4 Oxidation (P) |
| 773.3439 | 2317.01 | 2317.01 | 53.69 | GEPGPPGPAGAAGPAGNPGADGQPGAK | 2 Deamidated (NQ); 4 Oxidation (P) |
| 773.3439 | 2317.01 | 2317.01 | 63.38 | GEPGPPGPAGAAGPAGNPGADGQPGAK | 2 Deamidated (NQ); 4 Oxidation (P) |
| 1159.517 | 2317.019 | 2317.01 | 55.25 | GEPGPPGPAGAAGPAGNPGADGQPGAK | 2 Deamidated (NQ); 4 Oxidation (P) |
| 1162.071 | 2322.127 | 2322.124 | 61.43 | GDAGPPGPAGPAGPPGPIGNVGAPGPK | 4 Oxidation (P) |
| 1162.071 | 2322.127 | 2322.124 | 56.14 | GDAGPPGPAGPAGPPGPIGNVGAPGPK | 4 Oxidation (P) |
| 1162.562 | 2323.109 | 2323.108 | 63.48 | GDAGPPGPAGPAGPPGPIGNVGAPGPK | Deamidated (NQ); 4 Oxidation (P) |
| 775.3771 | 2323.11 | 2323.108 | 40.41 | GDAGPPGPAGPAGPPGPIGNVGAPGPK | Deamidated (NQ); 4 Oxidation (P) |
| 1162.564 | 2323.113 | 2323.108 | 70.02 | GDAGPPGPAGPAGPPGPIGNVGAPGPK | Deamidated (NQ); 4 Oxidation (P) |
| 824.4108 | 2470.211 | 2470.209 | 50.72 | GPPGSAGSPGKDGLNGLPGPIGPPGPR | Deamidated (NQ); 4 Oxidation (P) |
| 824.4108 | 2470.211 | 2470.209 | 51.64 | GPPGSAGSPGKDGLNGLPGPIGPPGPR | Deamidated (NQ); 4 Oxidation (P) |
| 827.7426 | 2480.206 | 2480.205 | 40.58 | GDRGETGPAGPPGAPGAPGAPGPVGPAGK | 2 Oxidation (P) |
| 829.7421 | 2486.205 | 2486.204 | 47.32 | GPPGSAGSPGKDGLNGLPGPIGPPGPR | Deamidated (NQ); 5 Oxidation (P) |
| 829.7423 | 2486.205 | 2486.204 | 51.18 | GPPGSAGSPGKDGLNGLPGPIGPPGPR | Deamidated (NQ); 5 Oxidation (P) |
| 829.7423 | 2486.205 | 2486.204 | 63.5 | GPPGSAGSPGKDGLNGLPGPIGPPGPR | Deamidated (NQ); 5 Oxidation (P) |
| 1244.11 | 2486.205 | 2486.204 | 42.75 | GPPGSAGSPGKDGLNGLPGPIGPPGPR | Deamidated (NQ); 5 Oxidation (P) |
| 829.7426 | 2486.206 | 2486.204 | 47.46 | GPPGSAGSPGKDGLNGLPGPIGPPGPR | Deamidated (NQ); 5 Oxidation (P) |
| 829.7426 | 2486.206 | 2486.204 | 41.08 | GPPGSAGSPGKDGLNGLPGPIGPPGPR | Deamidated (NQ); 5 Oxidation (P) |
| 829.7426 | 2486.206 | 2486.204 | 40.8 | GPPGSAGSPGKDGLNGLPGPIGPPGPR | Deamidated (NQ); 5 Oxidation (P) |
| 833.0742 | 2496.201 | 2496.199 | 44.53 | GDRGETGPAGPPGAPGAPGAPGPVGPAGK | 3 Oxidation (P) |
| 838.4059 | 2512.196 | 2512.194 | 43.43 | GDRGETGPAGPPGAPGAPGAPGPVGPAGK | 4 Oxidation (P) |
| 838.4059 | 2512.196 | 2512.194 | 51.48 | GDRGETGPAGPPGAPGAPGAPGPVGPAGK | 4 Oxidation (P) |
| 845.0701 | 2532.189 | 2532.188 | 55.66 | GNDGATGAAGPPGPTGPAGPPGFPGAVGAK | Deamidated (NQ); 2 Oxidation (P) |
| 1267.106 | 2532.197 | 2532.188 | 41.91 | GNDGATGAAGPPGPTGPAGPPGFPGAVGAK | Deamidated (NQ); 2 Oxidation (P) |
| 1274.608 | 2547.201 | 2547.199 | 91.58 | GNDGATGAAGPPGPTGPAGPPGFPGAVGAK | 3 Oxidation (P) |
| 1274.609 | 2547.203 | 2547.199 | 63.55 | GNDGATGAAGPPGPTGPAGPPGFPGAVGAK | 3 Oxidation (P) |
| 1275.1 | 2548.185 | 2548.183 | 93.92 | GNDGATGAAGPPGPTGPAGPPGFPGAVGAK | Deamidated (NQ); 3 Oxidation (P) |
| 1275.1 | 2548.185 | 2548.183 | 97.84 | GNDGATGAAGPPGPTGPAGPPGFPGAVGAK | Deamidated (NQ); 3 Oxidation (P) |
| 850.4053 | 2548.194 | 2548.183 | 49.71 | GNDGATGAAGPPGPTGPAGPPGFPGAVGAK | Deamidated (NQ); 3 Oxidation (P) |
| 1283.097 | 2564.179 | 2564.178 | 75.89 | GNDGATGAAGPPGPTGPAGPPGFPGAVGAK | Deamidated (NQ); Oxidation (K); 3 Oxidation (P) |
| 1283.097 | 2564.179 | 2564.178 | 75.23 | GNDGATGAAGPPGPTGPAGPPGFPGAVGAK | Deamidated (NQ); Oxidation (K); 3 Oxidation (P) |
| 855.7441 | 2564.211 | 2564.21 | 40.41 | GDTGAKGEPGPTGIQGPPGPAGEEGKR | Oxidation (K); 2 Oxidation (P) |
| 897.4199 | 2689.238 | 2689.237 | 42.36 | GFSGLQGPPGPPGSPGEQGPSGASGPAGPR | Deamidated (NQ); 2 Oxidation (P) |
| 897.4203 | 2689.239 | 2689.237 | 44.24 | GFSGLQGPPGPPGSPGEQGPSGASGPAGPR | Deamidated (NQ); 2 Oxidation (P) |
| 902.4237 | 2704.249 | 2704.248 | 41.13 | GFSGLQGPPGPPGSPGEQGPSGASGPAGPR | 3 Oxidation (P) |
| 902.4238 | 2704.25 | 2704.248 | 48.23 | GFSGLQGPPGPPGSPGEQGPSGASGPAGPR | 3 Oxidation (P) |
| 902.424 | 2704.25 | 2704.248 | 44.48 | GFSGLQGPPGPPGSPGEQGPSGASGPAGPR | 3 Oxidation (P) |
| 902.7516 | 2705.233 | 2705.232 | 50.65 | GFSGLQGPPGPPGSPGEQGPSGASGPAGPR | Deamidated (NQ); 3 Oxidation (P) |
| 903.0788 | 2706.215 | 2706.216 | 41.82 | GFSGLQGPPGPPGSPGEQGPSGASGPAGPR | 2 Deamidated (NQ); 3 Oxidation (P) |
| 903.0804 | 2706.219 | 2706.216 | 42.07 | GFSGLQGPPGPPGSPGEQGPSGASGPAGPR | 2 Deamidated (NQ); 3 Oxidation (P) |
| 907.4142 | 2719.221 | 2719.211 | 41.37 | GAPGDRGEPGPPGPAGFAGPPGADGQPGAK | Deamidated (NQ); 5 Oxidation (P) |
| 951.8102 | 2852.409 | 2852.405 | 42.52 | GLTGPIGPPGPAGAPGDKGEAGPSGPAGPTGAR | 2 Oxidation (P) |
| 1030.503 | 3088.487 | 3088.485 | 50.17 | GEPGDAGAKGDAGPPGPAGPAGPPGPIGNVGAPGPK | Oxidation (K); 2 Oxidation (P) |
| 1041.495 | 3121.463 | 3121.459 | 43.36 | GEPGDAGAKGDAGPPGPAGPAGPPGPIGNVGAPGPK | Deamidated (NQ); 5 Oxidation (P) |
| 1081.797 | 3242.369 | 3242.365 | 43.67 | GANGAPGNDGAKGDAGAPGAPGSQGAPGLQGMPGER | 3 Deamidated (NQ); Oxidation (M); 5 Oxidation (P) |

| Collagen alpha-2(I) chain OS=Bos taurus GN=COL1A2 PE=1 SV=2 | 5454 |
| --- | --- |

| 446.7534 | 891.4922 | 891.4926 | 40.01 | PGPIGPAGAR | |
| --- | --- | --- | --- | --- | --- |
| 454.751 | 907.4874 | 907.4876 | 41.03 | PGPIGPAGAR | Oxidation (P) |
| 542.7852 | 1083.556 | 1083.556 | 60.89 | GLVGEPGPAGSK | Oxidation (P) |
| 591.8094 | 1181.604 | 1181.604 | 43.27 | EGPVGLPGIDGR | Oxidation (P) |
| 591.8094 | 1181.604 | 1181.604 | 41.52 | EGPVGLPGIDGR | Oxidation (P) |
| 591.8094 | 1181.604 | 1181.604 | 45.37 | EGPVGLPGIDGR | Oxidation (P) |
| 596.8436 | 1191.673 | 1191.672 | 73.28 | IGQPGAVGPAGIR | |
| 596.8436 | 1191.673 | 1191.672 | 68.52 | IGQPGAVGPAGIR | |
| 597.3352 | 1192.656 | 1192.656 | 53.5 | IGQPGAVGPAGIR | Deamidated (NQ) |
| 597.3354 | 1192.656 | 1192.656 | 55.94 | IGQPGAVGPAGIR | Deamidated (NQ) |
| 597.3357 | 1192.657 | 1192.656 | 56.55 | IGQPGAVGPAGIR | Deamidated (NQ) |
| 597.3357 | 1192.657 | 1192.656 | 42.81 | IGQPGAVGPAGIR | Deamidated (NQ) |
| 601.2961 | 1200.578 | 1200.578 | 48.73 | GEPGNIGFPGPK | 2 Oxidation (P) |
| 601.2963 | 1200.578 | 1200.578 | 55.35 | GEPGNIGFPGPK | 2 Oxidation (P) |
| 601.7881 | 1201.562 | 1201.562 | 44.41 | GEPGNIGFPGPK | Deamidated (NQ); 2 Oxidation (P) |
| 601.7882 | 1201.562 | 1201.562 | 46.23 | GEPGNIGFPGPK | Deamidated (NQ); 2 Oxidation (P) |
| 601.7883 | 1201.562 | 1201.562 | 52.03 | GEPGNIGFPGPK | Deamidated (NQ); 2 Oxidation (P) |
| 601.7883 | 1201.562 | 1201.562 | 51.09 | GEPGNIGFPGPK | Deamidated (NQ); 2 Oxidation (P) |
| 403.5629 | 1207.667 | 1207.667 | 42.79 | IGQPGAVGPAGIR | Oxidation (P) |
| 604.841 | 1207.667 | 1207.667 | 54.01 | IGQPGAVGPAGIR | Oxidation (P) |
| 604.841 | 1207.667 | 1207.667 | 55.03 | IGQPGAVGPAGIR | Oxidation (P) |
| 604.841 | 1207.667 | 1207.667 | 43.15 | IGQPGAVGPAGIR | Oxidation (P) |
| 604.8411 | 1207.668 | 1207.667 | 55.94 | IGQPGAVGPAGIR | Oxidation (P) |
| 604.8411 | 1207.668 | 1207.667 | 64.49 | IGQPGAVGPAGIR | Oxidation (P) |
| 605.3328 | 1208.651 | 1208.651 | 65.05 | IGQPGAVGPAGIR | Deamidated (NQ); Oxidation (P) |
| 605.3328 | 1208.651 | 1208.651 | 43.38 | IGQPGAVGPAGIR | Deamidated (NQ); Oxidation (P) |
| 605.3329 | 1208.651 | 1208.651 | 53.43 | IGQPGAVGPAGIR | Deamidated (NQ); Oxidation (P) |
| 605.333 | 1208.651 | 1208.651 | 60.67 | IGQPGAVGPAGIR | Deamidated (NQ); Oxidation (P) |
| 605.333 | 1208.651 | 1208.651 | 45.49 | IGQPGAVGPAGIR | Deamidated (NQ); Oxidation (P) |
| 605.3331 | 1208.652 | 1208.651 | 59.52 | IGQPGAVGPAGIR | Deamidated (NQ); Oxidation (P) |
| 605.3331 | 1208.652 | 1208.651 | 64.04 | IGQPGAVGPAGIR | Deamidated (NQ); Oxidation (P) |
| 605.3331 | 1208.652 | 1208.651 | 49.4 | IGQPGAVGPAGIR | Deamidated (NQ); Oxidation (P) |
| 605.3331 | 1208.652 | 1208.651 | 62.46 | IGQPGAVGPAGIR | Deamidated (NQ); Oxidation (P) |
| 605.3333 | 1208.652 | 1208.651 | 45.33 | IGQPGAVGPAGIR | Deamidated (NQ); Oxidation (P) |
| 611.809 | 1221.603 | 1221.603 | 41 | GFPGTPGLPGFK | 3 Oxidation (P) |
| 631.3179 | 1260.621 | 1260.621 | 53.72 | GEAGPAGPAGPAGPR | |
| 631.3179 | 1260.621 | 1260.621 | 57.72 | GEAGPAGPAGPAGPR | |
| 634.3413 | 1266.668 | 1266.668 | 43.55 | GIPGPVGAAGATGAR | Oxidation (P) |
| 634.3414 | 1266.668 | 1266.668 | 68.81 | GIPGPVGAAGATGAR | Oxidation (P) |
| 634.3414 | 1266.668 | 1266.668 | 64.31 | GIPGPVGAAGATGAR | Oxidation (P) |
| 634.3414 | 1266.668 | 1266.668 | 52.66 | GIPGPVGAAGATGAR | Oxidation (P) |
| 634.3415 | 1266.668 | 1266.668 | 57.38 | GIPGPVGAAGATGAR | Oxidation (P) |
| 644.811 | 1287.607 | 1287.61 | 54.12 | GFPGSPGNIGPAGK | Deamidated (NQ); 2 Oxidation (P) |
| 644.8119 | 1287.609 | 1287.61 | 41.73 | GFPGSPGNIGPAGK | Deamidated (NQ); 2 Oxidation (P) |
| 644.8121 | 1287.61 | 1287.61 | 77.11 | GFPGSPGNIGPAGK | Deamidated (NQ); 2 Oxidation (P) |
| 644.8122 | 1287.61 | 1287.61 | 64.14 | GFPGSPGNIGPAGK | Deamidated (NQ); 2 Oxidation (P) |
| 714.3676 | 1426.721 | 1426.721 | 76.79 | GIPGEFGLPGPAGAR | 2 Oxidation (P) |
| 714.3676 | 1426.721 | 1426.721 | 56.29 | GIPGEFGLPGPAGAR | 2 Oxidation (P) |
| 714.3676 | 1426.721 | 1426.721 | 49.99 | GIPGEFGLPGPAGAR | 2 Oxidation (P) |
| 714.3676 | 1426.721 | 1426.721 | 53.27 | GIPGEFGLPGPAGAR | 2 Oxidation (P) |
| 714.3676 | 1426.721 | 1426.721 | 41.96 | GIPGEFGLPGPAGAR | 2 Oxidation (P) |
| 714.3677 | 1426.721 | 1426.721 | 60.1 | GIPGEFGLPGPAGAR | 2 Oxidation (P) |
| 714.3677 | 1426.721 | 1426.721 | 40.13 | GIPGEFGLPGPAGAR | 2 Oxidation (P) |
| 714.3679 | 1426.721 | 1426.721 | 64.26 | GIPGEFGLPGPAGAR | 2 Oxidation (P) |
| 714.3679 | 1426.721 | 1426.721 | 43.68 | GIPGEFGLPGPAGAR | 2 Oxidation (P) |
| 714.368 | 1426.721 | 1426.721 | 53.31 | GIPGEFGLPGPAGAR | 2 Oxidation (P) |
| 714.368 | 1426.721 | 1426.721 | 60.71 | GIPGEFGLPGPAGAR | 2 Oxidation (P) |
| 714.3681 | 1426.722 | 1426.721 | 42.76 | GIPGEFGLPGPAGAR | 2 Oxidation (P) |
| 714.3687 | 1426.723 | 1426.721 | 44.74 | GIPGEFGLPGPAGAR | 2 Oxidation (P) |
| 714.3688 | 1426.723 | 1426.721 | 42.41 | GIPGEFGLPGPAGAR | 2 Oxidation (P) |
| 722.3654 | 1442.716 | 1442.715 | 47.24 | GIPGEFGLPGPAGAR | 3 Oxidation (P) |
| 722.3655 | 1442.716 | 1442.715 | 48.58 | GIPGEFGLPGPAGAR | 3 Oxidation (P) |
| 737.3395 | 1472.664 | 1472.665 | 71.44 | GDGGPPGATGFPGAAGR | 2 Oxidation (P) |
| 737.3395 | 1472.664 | 1472.665 | 90.01 | GDGGPPGATGFPGAAGR | 2 Oxidation (P) |
| 737.3395 | 1472.664 | 1472.665 | 46.51 | GDGGPPGATGFPGAAGR | 2 Oxidation (P) |
| 737.3396 | 1472.665 | 1472.665 | 42.53 | GDGGPPGATGFPGAAGR | 2 Oxidation (P) |
| 746.8496 | 1491.685 | 1491.684 | 71.5 | SGETGASGPPGFVGEK | Oxidation (P) |
| 746.8497 | 1491.685 | 1491.684 | 68.07 | SGETGASGPPGFVGEK | Oxidation (P) |
| 746.8497 | 1491.685 | 1491.684 | 72.22 | SGETGASGPPGFVGEK | Oxidation (P) |
| 746.8498 | 1491.685 | 1491.684 | 74.28 | SGETGASGPPGFVGEK | Oxidation (P) |
| 746.8498 | 1491.685 | 1491.684 | 41.43 | SGETGASGPPGFVGEK | Oxidation (P) |
| 511.5986 | 1531.774 | 1531.774 | 54.45 | GEPGPAGAVGPAGAVGPR | Oxidation (P) |
| 511.5986 | 1531.774 | 1531.774 | 54.2 | GEPGPAGAVGPAGAVGPR | Oxidation (P) |
| 766.8947 | 1531.775 | 1531.774 | 54 | GEPGPAGAVGPAGAVGPR | Oxidation (P) |
| 766.8948 | 1531.775 | 1531.774 | 92.61 | GEPGPAGAVGPAGAVGPR | Oxidation (P) |
| 766.8948 | 1531.775 | 1531.774 | 83.44 | GEPGPAGAVGPAGAVGPR | Oxidation (P) |
| 766.8948 | 1531.775 | 1531.774 | 87.22 | GEPGPAGAVGPAGAVGPR | Oxidation (P) |
| 766.8948 | 1531.775 | 1531.774 | 42.52 | GEPGPAGAVGPAGAVGPR | Oxidation (P) |
| 766.8949 | 1531.775 | 1531.774 | 57.88 | GEPGPAGAVGPAGAVGPR | Oxidation (P) |
| 769.3717 | 1536.729 | 1536.728 | 42.11 | GAPGAIGAPGPAGANGDR | 2 Oxidation (P) |
| 769.8636 | 1537.713 | 1537.712 | 46.13 | GAPGAIGAPGPAGANGDR | Deamidated (NQ); 2 Oxidation (P) |
| 769.8637 | 1537.713 | 1537.712 | 58.1 | GAPGAIGAPGPAGANGDR | Deamidated (NQ); 2 Oxidation (P) |
| 521.6143 | 1561.821 | 1561.821 | 48.66 | GAAGLPGVAGAPGLPGPR | 3 Oxidation (P) |
| 781.9182 | 1561.822 | 1561.821 | 66.89 | GAAGLPGVAGAPGLPGPR | 3 Oxidation (P) |
| 781.9184 | 1561.822 | 1561.821 | 64.03 | GAAGLPGVAGAPGLPGPR | 3 Oxidation (P) |
| 781.9185 | 1561.822 | 1561.821 | 45.22 | GAAGLPGVAGAPGLPGPR | 3 Oxidation (P) |
| 790.8871 | 1579.76 | 1579.759 | 92.4 | GPPGESGAAGPTGPIGSR | Oxidation (P) |
| 790.8871 | 1579.76 | 1579.759 | 53.09 | GPPGESGAAGPTGPIGSR | Oxidation (P) |
| 790.8872 | 1579.76 | 1579.759 | 88.99 | GPPGESGAAGPTGPIGSR | Oxidation (P) |
| 790.8875 | 1579.76 | 1579.759 | 71.28 | GPPGESGAAGPTGPIGSR | Oxidation (P) |
| 790.8876 | 1579.761 | 1579.759 | 75.9 | GPPGESGAAGPTGPIGSR | Oxidation (P) |
| 800.4157 | 1598.817 | 1598.817 | 60.65 | GELGPVGNPGPAGPAGPR | |
| 800.416 | 1598.817 | 1598.817 | 70.34 | GELGPVGNPGPAGPAGPR | |
| 800.908 | 1599.801 | 1599.801 | 61.26 | GELGPVGNPGPAGPAGPR | Deamidated (NQ) |
| 800.9081 | 1599.802 | 1599.801 | 78.08 | GELGPVGNPGPAGPAGPR | Deamidated (NQ) |
| 808.4133 | 1614.812 | 1614.811 | 54.81 | GELGPVGNPGPAGPAGPR | Oxidation (P) |
| 808.4133 | 1614.812 | 1614.811 | 44 | GELGPVGNPGPAGPAGPR | Oxidation (P) |
| 808.9052 | 1615.796 | 1615.795 | 58.84 | GELGPVGNPGPAGPAGPR | Deamidated (NQ); Oxidation (P) |
| 808.9052 | 1615.796 | 1615.795 | 56.44 | GELGPVGNPGPAGPAGPR | Deamidated (NQ); Oxidation (P) |
| 808.9052 | 1615.796 | 1615.795 | 42.31 | GELGPVGNPGPAGPAGPR | Deamidated (NQ); Oxidation (P) |
| 808.9056 | 1615.797 | 1615.795 | 43.4 | GELGPVGNPGPAGPAGPR | Deamidated (NQ); Oxidation (P) |
| 824.9184 | 1647.822 | 1647.822 | 59.67 | GSTGEIGPAGPPGPPGLR | 2 Oxidation (P) |
| 824.9185 | 1647.822 | 1647.822 | 51.32 | GSTGEIGPAGPPGPPGLR | 2 Oxidation (P) |
| 824.9185 | 1647.822 | 1647.822 | 54.75 | GSTGEIGPAGPPGPPGLR | 2 Oxidation (P) |
| 562.2897 | 1683.847 | 1683.848 | 42.62 | AGVMGPAGSRGATGPAGVR | Oxidation (M) |
| 562.2898 | 1683.848 | 1683.848 | 40.28 | AGVMGPAGSRGATGPAGVR | Oxidation (M) |
| 602.3151 | 1803.924 | 1803.923 | 44.84 | RGSTGEIGPAGPPGPPGLR | 2 Oxidation (P) |
| 912.9033 | 1823.792 | 1823.792 | 87.75 | GPPGNVGNPGVNGAPGEAGR | 3 Deamidated (NQ); 3 Oxidation (P) |
| 912.9038 | 1823.793 | 1823.792 | 82.85 | GPPGNVGNPGVNGAPGEAGR | 3 Deamidated (NQ); 3 Oxidation (P) |
| 912.9041 | 1823.794 | 1823.792 | 76.21 | GPPGNVGNPGVNGAPGEAGR | 3 Deamidated (NQ); 3 Oxidation (P) |
| 915.455 | 1828.895 | 1828.896 | 40.92 | TGPPGPSGISGPPGPPGPAGK | Oxidation (K); 2 Oxidation (P) |
| 915.4552 | 1828.896 | 1828.896 | 45.69 | TGPPGPSGISGPPGPPGPAGK | Oxidation (K); 2 Oxidation (P) |
| 915.4562 | 1828.898 | 1828.896 | 41.63 | TGPPGPSGISGPPGPPGPAGK | Oxidation (K); 2 Oxidation (P) |
| 923.4533 | 1844.892 | 1844.891 | 48.23 | TGPPGPSGISGPPGPPGPAGK | 4 Oxidation (P) |
| 666.6684 | 1996.983 | 1996.983 | 55.66 | HGNRGEPGPAGAVGPAGAVGPR | Deamidated (NQ); Oxidation (P) |
| 666.6685 | 1996.984 | 1996.983 | 49.55 | HGNRGEPGPAGAVGPAGAVGPR | Deamidated (NQ); Oxidation (P) |
| 684.6638 | 2050.97 | 2050.971 | 42.33 | GEVGPAGPNGFAGPAGAAGQPGAK | Deamidated (NQ); Oxidation (P) |
| 1026.494 | 2050.973 | 2050.971 | 80.66 | GEVGPAGPNGFAGPAGAAGQPGAK | Deamidated (NQ); Oxidation (P) |
| 1026.494 | 2050.973 | 2050.971 | 71.04 | GEVGPAGPNGFAGPAGAAGQPGAK | Deamidated (NQ); Oxidation (P) |
| 1026.494 | 2050.973 | 2050.971 | 84.4 | GEVGPAGPNGFAGPAGAAGQPGAK | Deamidated (NQ); Oxidation (P) |
| 684.9922 | 2051.955 | 2051.955 | 43.03 | GEVGPAGPNGFAGPAGAAGQPGAK | 2 Deamidated (NQ); Oxidation (P) |
| 1026.985 | 2051.955 | 2051.955 | 68.71 | GEVGPAGPNGFAGPAGAAGQPGAK | 2 Deamidated (NQ); Oxidation (P) |
| 1026.985 | 2051.955 | 2051.955 | 62.64 | GEVGPAGPNGFAGPAGAAGQPGAK | 2 Deamidated (NQ); Oxidation (P) |
| 1026.986 | 2051.957 | 2051.955 | 73.31 | GEVGPAGPNGFAGPAGAAGQPGAK | 2 Deamidated (NQ); Oxidation (P) |
| 1026.986 | 2051.957 | 2051.955 | 66.81 | GEVGPAGPNGFAGPAGAAGQPGAK | 2 Deamidated (NQ); Oxidation (P) |
| 1026.986 | 2051.957 | 2051.955 | 63.12 | GEVGPAGPNGFAGPAGAAGQPGAK | 2 Deamidated (NQ); Oxidation (K) |
| 1034.489 | 2066.963 | 2066.966 | 79.29 | GEVGPAGPNGFAGPAGAAGQPGAK | Deamidated (NQ); Oxidation (K); Oxidation (P) |
| 1034.49 | 2066.965 | 2066.966 | 55.95 | GEVGPAGPNGFAGPAGAAGQPGAK | Deamidated (NQ); Oxidation (K); Oxidation (P) |
| 1034.491 | 2066.967 | 2066.966 | 83.23 | GEVGPAGPNGFAGPAGAAGQPGAK | Deamidated (NQ); Oxidation (K); Oxidation (P) |
| 1034.491 | 2066.967 | 2066.966 | 56.72 | GEVGPAGPNGFAGPAGAAGQPGAK | Deamidated (NQ); Oxidation (K); Oxidation (P) |
| 1034.984 | 2067.953 | 2067.95 | 83.67 | GEVGPAGPNGFAGPAGAAGQPGAK | 2 Deamidated (NQ); Oxidation (K); Oxidation (P) |
| 1066.06 | 2130.105 | 2130.107 | 67.52 | GLPGVAGSVGEPGPLGIAGPPGAR | 3 Oxidation (P) |
| 1066.061 | 2130.107 | 2130.107 | 74.96 | GLPGVAGSVGEPGPLGIAGPPGAR | 3 Oxidation (P) |
| 1066.061 | 2130.107 | 2130.107 | 102.86 | GLPGVAGSVGEPGPLGIAGPPGAR | 3 Oxidation (P) |
| 1066.061 | 2130.107 | 2130.107 | 97.43 | GLPGVAGSVGEPGPLGIAGPPGAR | 3 Oxidation (P) |
| 1066.061 | 2130.107 | 2130.107 | 72.09 | GLPGVAGSVGEPGPLGIAGPPGAR | 3 Oxidation (P) |
| 1066.061 | 2130.107 | 2130.107 | 56.58 | GLPGVAGSVGEPGPLGIAGPPGAR | 3 Oxidation (P) |
| 1066.061 | 2130.107 | 2130.107 | 71.19 | GLPGVAGSVGEPGPLGIAGPPGAR | 3 Oxidation (P) |
| 1066.061 | 2130.107 | 2130.107 | 41.15 | GLPGVAGSVGEPGPLGIAGPPGAR | 3 Oxidation (P) |
| 1066.061 | 2130.107 | 2130.107 | 45.77 | GLPGVAGSVGEPGPLGIAGPPGAR | 3 Oxidation (P) |
| 1066.061 | 2130.107 | 2130.107 | 55.58 | GLPGVAGSVGEPGPLGIAGPPGAR | 3 Oxidation (P) |
| 1066.061 | 2130.107 | 2130.107 | 88.42 | GLPGVAGSVGEPGPLGIAGPPGAR | 3 Oxidation (P) |
| 1066.061 | 2130.107 | 2130.107 | 89.73 | GLPGVAGSVGEPGPLGIAGPPGAR | 3 Oxidation (P) |
| 1066.061 | 2130.107 | 2130.107 | 53.91 | GLPGVAGSVGEPGPLGIAGPPGAR | 3 Oxidation (P) |
| 711.0432 | 2130.108 | 2130.107 | 60.6 | GLPGVAGSVGEPGPLGIAGPPGAR | 3 Oxidation (P) |
| 711.0433 | 2130.108 | 2130.107 | 44.76 | GLPGVAGSVGEPGPLGIAGPPGAR | 3 Oxidation (P) |
| 711.0434 | 2130.108 | 2130.107 | 48.04 | GLPGVAGSVGEPGPLGIAGPPGAR | 3 Oxidation (P) |
| 711.0434 | 2130.108 | 2130.107 | 41.5 | GLPGVAGSVGEPGPLGIAGPPGAR | 3 Oxidation (P) |
| 711.0435 | 2130.109 | 2130.107 | 54.99 | GLPGVAGSVGEPGPLGIAGPPGAR | 3 Oxidation (P) |
| 711.0435 | 2130.109 | 2130.107 | 47.23 | GLPGVAGSVGEPGPLGIAGPPGAR | 3 Oxidation (P) |
| 1066.062 | 2130.109 | 2130.107 | 78.82 | GLPGVAGSVGEPGPLGIAGPPGAR | 3 Oxidation (P) |
| 1066.062 | 2130.109 | 2130.107 | 92.24 | GLPGVAGSVGEPGPLGIAGPPGAR | 3 Oxidation (P) |
| 1066.062 | 2130.109 | 2130.107 | 84.51 | GLPGVAGSVGEPGPLGIAGPPGAR | 3 Oxidation (P) |
| 1066.062 | 2130.109 | 2130.107 | 73.35 | GLPGVAGSVGEPGPLGIAGPPGAR | 3 Oxidation (P) |
| 1066.062 | 2130.109 | 2130.107 | 76.41 | GLPGVAGSVGEPGPLGIAGPPGAR | 3 Oxidation (P) |
| 1066.062 | 2130.109 | 2130.107 | 58.76 | GLPGVAGSVGEPGPLGIAGPPGAR | 3 Oxidation (P) |
| 711.0438 | 2130.11 | 2130.107 | 55.87 | GLPGVAGSVGEPGPLGIAGPPGAR | 3 Oxidation (P) |
| 1066.063 | 2130.111 | 2130.107 | 49.09 | GLPGVAGSVGEPGPLGIAGPPGAR | 3 Oxidation (P) |
| 1066.063 | 2130.111 | 2130.107 | 66.78 | GLPGVAGSVGEPGPLGIAGPPGAR | 3 Oxidation (P) |
| 1066.063 | 2130.111 | 2130.107 | 91.3 | GLPGVAGSVGEPGPLGIAGPPGAR | 3 Oxidation (P) |
| 1066.066 | 2130.117 | 2130.107 | 44.45 | GLPGVAGSVGEPGPLGIAGPPGAR | 3 Oxidation (P) |
| 1074.059 | 2146.103 | 2146.102 | 73.94 | GLPGVAGSVGEPGPLGIAGPPGAR | 4 Oxidation (P) |
| 1074.06 | 2146.105 | 2146.102 | 48.55 | GLPGVAGSVGEPGPLGIAGPPGAR | 4 Oxidation (P) |
| 804.0529 | 2409.137 | 2409.131 | 44.72 | GEVGPAGPNGFAGPAGAAGQPGAKGER | Deamidated (NQ); Oxidation (K); Oxidation (P) |
| 804.3802 | 2410.119 | 2410.115 | 60.89 | GEVGPAGPNGFAGPAGAAGQPGAKGER | 2 Deamidated (NQ); Oxidation (K); Oxidation (P) |
| 1216.11 | 2430.205 | 2430.203 | 66.48 | GEVGLPGLSGPVGPPGNPGANGLPGAK | Deamidated (NQ); 4 Oxidation (P) |
| 1216.11 | 2430.205 | 2430.203 | 80.26 | GEVGLPGLSGPVGPPGNPGANGLPGAK | Deamidated (NQ); 4 Oxidation (P) |
| 1216.601 | 2431.187 | 2431.187 | 64.84 | GEVGLPGLSGPVGPPGNPGANGLPGAK | 2 Deamidated (NQ); 4 Oxidation (P) |
| 1216.601 | 2431.187 | 2431.187 | 83.05 | GEVGLPGLSGPVGPPGNPGANGLPGAK | 2 Deamidated (NQ); 4 Oxidation (P) |
| 811.4032 | 2431.188 | 2431.187 | 50.29 | GEVGLPGLSGPVGPPGNPGANGLPGAK | 2 Deamidated (NQ); 4 Oxidation (P) |
| 811.4069 | 2431.199 | 2431.187 | 66.18 | GEVGLPGLSGPVGPPGNPGANGLPGAK | 2 Deamidated (NQ); 4 Oxidation (P) |
| 816.0792 | 2445.216 | 2445.214 | 61.26 | GEVGLPGLSGPVGPPGNPGANGLPGAK | Oxidation (K); 4 Oxidation (P) |
| 816.4073 | 2446.2 | 2446.198 | 54.93 | GEVGLPGLSGPVGPPGNPGANGLPGAK | Deamidated (NQ); Oxidation (K); 4 Oxidation (P) |
| 816.4074 | 2446.2 | 2446.198 | 44.14 | GEVGLPGLSGPVGPPGNPGANGLPGAK | Deamidated (NQ); Oxidation (K); 4 Oxidation (P) |
| 1224.108 | 2446.201 | 2446.198 | 55.24 | GEVGLPGLSGPVGPPGNPGANGLPGAK | Deamidated (NQ); Oxidation (K); 4 Oxidation (P) |
| 1224.6 | 2447.185 | 2447.182 | 78.6 | GEVGLPGLSGPVGPPGNPGANGLPGAK | 2 Deamidated (NQ); Oxidation (K); 4 Oxidation (P) |
| 1224.6 | 2447.185 | 2447.182 | 49.84 | GEVGLPGLSGPVGPPGNPGANGLPGAK | 2 Deamidated (NQ); Oxidation (K); 4 Oxidation (P) |
| 1287.135 | 2572.255 | 2572.256 | 95.5 | GSDGSVGPVGPAGPIGSAGPPGFPGAPGPK | Oxidation (K); Oxidation (P) |
| 858.4263 | 2572.257 | 2572.256 | 59.15 | GSDGSVGPVGPAGPIGSAGPPGFPGAPGPK | Oxidation (K); Oxidation (P) |
| 858.4265 | 2572.258 | 2572.256 | 60.25 | GSDGSVGPVGPAGPIGSAGPPGFPGAPGPK | Oxidation (K); Oxidation (P) |
| 861.0846 | 2580.232 | 2580.232 | 57.13 | GENGPVGPTGPVGAAGPSGPNGPPGPAGSR | Oxidation (P) |
| 861.0861 | 2580.237 | 2580.232 | 51.49 | GENGPVGPTGPVGAAGPSGPNGPPGPAGSR | Oxidation (P) |
| 861.7405 | 2582.2 | 2582.2 | 52.74 | GENGPVGPTGPVGAAGPSGPNGPPGPAGSR | 2 Deamidated (NQ); Oxidation (P) |
| 861.7406 | 2582.2 | 2582.2 | 63.06 | GENGPVGPTGPVGAAGPSGPNGPPGPAGSR | 2 Deamidated (NQ); Oxidation (P) |
| 861.7414 | 2582.202 | 2582.2 | 47.31 | GENGPVGPTGPVGAAGPSGPNGPPGPAGSR | 2 Deamidated (NQ); Oxidation (P) |
| 1295.133 | 2588.251 | 2588.251 | 47.32 | GSDGSVGPVGPAGPIGSAGPPGFPGAPGPK | 3 Oxidation (P) |
| 863.7579 | 2588.252 | 2588.251 | 45.47 | GSDGSVGPVGPAGPIGSAGPPGFPGAPGPK | 3 Oxidation (P) |
| 1295.134 | 2588.253 | 2588.251 | 85.19 | GSDGSVGPVGPAGPIGSAGPPGFPGAPGPK | 3 Oxidation (P) |
| 1295.135 | 2588.255 | 2588.251 | 73.66 | GSDGSVGPVGPAGPIGSAGPPGFPGAPGPK | Oxidation (K); 2 Oxidation (P) |
| 1303.132 | 2604.249 | 2604.246 | 50.9 | GSDGSVGPVGPAGPIGSAGPPGFPGAPGPK | Oxidation (K); 3 Oxidation (P) |
| 927.7822 | 2780.325 | 2780.323 | 44.87 | GAPGAIGAPGPAGANGDRGEAGPAGPAGPAGPR | Deamidated (NQ); 2 Oxidation (P) |
| 931.7748 | 2792.303 | 2792.3 | 58.1 | GEQGPAGPPGFQGLPGPAGTAGEAGKPGER | Deamidated (NQ); Oxidation (K); 2 Oxidation (P) |
| 955.797 | 2864.369 | 2864.369 | 49.89 | GPKGENGPVGPTGPVGAAGPSGPNGPPGPAGSR | 2 Deamidated (NQ); Oxidation (P) |
| 955.798 | 2864.372 | 2864.369 | 54.47 | GPKGENGPVGPTGPVGAAGPSGPNGPPGPAGSR | 2 Deamidated (NQ); Oxidation (P) |
| 961.1292 | 2880.366 | 2880.364 | 62.53 | GPKGENGPVGPTGPVGAAGPSGPNGPPGPAGSR | 2 Deamidated (NQ); Oxidation (K); Oxidation (P) |
| 961.1293 | 2880.366 | 2880.364 | 53.63 | GPKGENGPVGPTGPVGAAGPSGPNGPPGPAGSR | 2 Deamidated (NQ); Oxidation (K); Oxidation (P) |
| 1120.562 | 3358.664 | 3358.654 | 41.09 | GPSGEPGTAGPPGTPGPQGLLGAPGFLGLPGSRGER | 4 Oxidation (P) |
| 1125.891 | 3374.651 | 3374.649 | 45.12 | GPSGEPGTAGPPGTPGPQGLLGAPGFLGLPGSRGER | 5 Oxidation (P) |
| 1126.219 | 3375.635 | 3375.633 | 40.23 | GPSGEPGTAGPPGTPGPQGLLGAPGFLGLPGSRGER | Deamidated (NQ); 5 Oxidation (P) |
| 1126.22 | 3375.638 | 3375.633 | 43.86 | GPSGEPGTAGPPGTPGPQGLLGAPGFLGLPGSRGER | Deamidated (NQ); 5 Oxidation (P) |

| Collagen alpha-1(II) chain OS=Bos taurus GN=COL2A1 PE=1 SV=4 | 253 |
| --- | --- |

| 656.8306 | 1311.647 | 1311.646 | 42.02 | GFPGLPGPSGEPGK | Oxidation (P) |
| --- | --- | --- | --- | --- | --- |
| 664.8279 | 1327.641 | 1327.641 | 52.92 | GFPGLPGPSGEPGK | 2 Oxidation (P) |
| 664.8279 | 1327.641 | 1327.641 | 42.25 | GFPGLPGPSGEPGK | 2 Oxidation (P) |
| 664.828 | 1327.641 | 1327.641 | 64.02 | GFPGLPGPSGEPGK | 2 Oxidation (P) |
| 664.828 | 1327.641 | 1327.641 | 56.43 | GFPGLPGPSGEPGK | 2 Oxidation (P) |
| 664.828 | 1327.641 | 1327.641 | 48.23 | GFPGLPGPSGEPGK | 2 Oxidation (P) |
| 664.8281 | 1327.642 | 1327.641 | 62.28 | GFPGLPGPSGEPGK | 2 Oxidation (P) |
| 664.8281 | 1327.642 | 1327.641 | 40.05 | GFPGLPGPSGEPGK | 2 Oxidation (P) |
| 664.8281 | 1327.642 | 1327.641 | 41.9 | GFPGLPGPSGEPGK | 2 Oxidation (P) |
| 664.8281 | 1327.642 | 1327.641 | 41.55 | GFPGLPGPSGEPGK | 2 Oxidation (P) |
| 664.8281 | 1327.642 | 1327.641 | 43.87 | GFPGLPGPSGEPGK | 2 Oxidation (P) |
| 672.8254 | 1343.636 | 1343.636 | 52.85 | GFPGLPGPSGEPGK | 3 Oxidation (P) |
| 672.8254 | 1343.636 | 1343.636 | 62.68 | GFPGLPGPSGEPGK | 3 Oxidation (P) |
| 672.8254 | 1343.636 | 1343.636 | 62.98 | GFPGLPGPSGEPGK | 3 Oxidation (P) |
| 672.8255 | 1343.636 | 1343.636 | 55.33 | GFPGLPGPSGEPGK | 3 Oxidation (P) |
| 758.8606 | 1515.707 | 1515.707 | 43 | GAQGPPGATGFPGAAGR | 3 Oxidation (P) |
| 758.861 | 1515.707 | 1515.707 | 43.59 | GAQGPPGATGFPGAAGR | 3 Oxidation (P) |

**Collagenase Batch 2**

**Modern**

| Serum albumin OS=Bos taurus GN=ALB PE=1 SV=4 | 344 |
| --- | --- |

| **pep_exp_mz** | **pep_exp_mr** | **pep_calc_mr** | **pep_score** | **pep_seq** | **pep_var_mod** |
| --- | --- | --- | --- | --- | --- |
| 461.7478 | 921.481 | 921.4807 | 40.54 | AEFVEVTK | |
| 507.8135 | 1013.612 | 1013.612 | 44.76 | QTALVELLK | |
| 417.2119 | 1248.614 | 1248.614 | 50.07 | FKDLGEEHFK | |
| 700.3505 | 1398.686 | 1398.685 | 53.52 | TVMENFVAFVDK | |
| 480.6087 | 1438.804 | 1438.805 | 47.71 | RHPEYAVSVLLR | |
| 480.6087 | 1438.804 | 1438.805 | 49.67 | RHPEYAVSVLLR | |
| 722.3247 | 1442.635 | 1442.635 | 46.24 | YICDNQDTISSK | |
| 740.3992 | 1478.784 | 1478.788 | 48.67 | LGEYGFQNALIVR | |
| 740.8937 | 1479.773 | 1479.772 | 48.06 | LGEYGFQNALIVR | Deamidated (NQ) |

| Pigment epithelium-derived factor OS=Bos taurus GN=SERPINF1 PE=1 SV=1 | 335 |
| --- | --- |

| 535.3346 | 1068.655 | 1068.654 | 50.21 | TVQAVLTIPK | |
| --- | --- | --- | --- | --- | --- |
| 535.8265 | 1069.638 | 1069.638 | 48.95 | TVQAVLTIPK | Deamidated (NQ) |
| 592.7672 | 1183.52 | 1183.518 | 53.1 | YGLDSDLNCK | |
| 625.8339 | 1249.653 | 1249.655 | 74.76 | DTDTGALLFIGK | |
| 780.3972 | 1558.78 | 1558.778 | 40.7 | LAAAVSNFGYDLYR | |
| 822.8923 | 1643.77 | 1643.768 | 44.74 | KTSLEDFYLDEER | |
| 908.9491 | 1815.884 | 1815.883 | 48.86 | VDLQEINNWVQAQMK | Deamidated (NQ) |
| 916.4549 | 1830.895 | 1830.893 | 40.71 | VDLQEINNWVQAQMK | Oxidation (M) |
| 1037.601 | 2073.187 | 2073.187 | 68.39 | IAQLPLTGSTSIIFFLPQK | |
| 1117.112 | 2232.209 | 2232.21 | 60.14 | LTFPLDYHLNQPFIFVLR | |

| Alpha-2-HS-glycoprotein OS=Bos taurus GN=AHSG PE=1 SV=2 | 333 |
| --- | --- |

| 408.7146 | 815.4146 | 815.4137 | 56.32 | ALGGEDVR | |
| --- | --- | --- | --- | --- | --- |
| 424.2208 | 846.427 | 846.4269 | 43.25 | CNLLAEK |  |
| 577.8115 | 1153.608 | 1153.609 | 68.38 | HTLNQIDSVK | |
| 578.3042 | 1154.594 | 1154.593 | 48.52 | HTLNQIDSVK | Deamidated (NQ) |
| 635.3277 | 1268.641 | 1268.64 | 64.57 | QDGQFSVLFTK | |
| 808.4211 | 1614.828 | 1614.829 | 44.61 | EVVDPTKCNLLAEK | |
| 816.382 | 1630.749 | 1630.748 | 43.91 | CNLLAEKQYGFCK | Deamidated (NQ) |
| 989.9694 | 1977.924 | 1977.921 | 42.67 | QQTQHAVEGDCDIHVLK | Deamidated (NQ) |
| 1260.164 | 2518.313 | 2518.314 | 62.53 | AQFVPLPVSVSVEFAVAATDCIAK | |
| 1006.525 | 3016.553 | 3016.551 | 55.07 | VVHAVEVALATFNAESNGSYLQLVEISR | Deamidated (NQ) |

| Chondroadherin OS=Bos taurus GN=CHAD PE=1 SV=1 | 283 |
| --- | --- |

| 876.4467 | 1750.879 | 1750.878 | 48.74 | YLETLWLDNTNLEK | |
| --- | --- | --- | --- | --- | --- |
| 876.4472 | 1750.88 | 1750.878 | 58.26 | YLETLWLDNTNLEK | |
| 876.9397 | 1751.865 | 1751.862 | 69.11 | YLETLWLDNTNLEK | Deamidated (NQ) |
| 1006.086 | 2010.157 | 2010.151 | 72.17 | GLLSPLVNLFILQLNNNK | Deamidated (NQ) |
| 1141.173 | 2280.331 | 2280.321 | 93.05 | GLLSPLVNLFILQLNNNKIR | 2 Deamidated (NQ) |
| 1381.699 | 2761.383 | 2761.381 | 47.27 | WLYLSENSLSSLQPGALDDVENLAK | |
| 938.1481 | 2811.423 | 2811.423 | 46.41 | LHQLPSNFPFDSLETLTLTNNPWK | |

| Prothrombin OS=Bos taurus GN=F2 PE=1 SV=2 | 276 |
| --- | --- |

| 456.7794 | 911.5442 | 911.544 | 52.78 | VTVEVIPR | |
| --- | --- | --- | --- | --- | --- |
| 574.7794 | 1147.544 | 1147.544 | 46.54 | SGIECQLWR | |
| 574.7796 | 1147.545 | 1147.544 | 42.02 | SGIECQLWR | |
| 823.4156 | 1644.817 | 1644.814 | 41.88 | SPQELLCGASLISDR | |
| 922.398 | 1842.781 | 1842.782 | 47.08 | WYQMGIVSWGEGCDR | |
| 1061.998 | 2121.981 | 2121.979 | 63.43 | NPDGSITGPWCYTTSPTLR | |
| 1137.583 | 2273.151 | 2273.151 | 53.13 | IVEGQDAEVGLSPWQVMLFR | |
| 1145.581 | 2289.147 | 2289.146 | 57.82 | IVEGQDAEVGLSPWQVMLFR | Oxidation (M) |
| 1146.558 | 2291.101 | 2291.1 | 45.81 | ALSKDQDFNPAVPLAENFCR | |

| Biglycan OS=Bos taurus GN=BGN PE=1 SV=3 | 254 |
| --- | --- |

| 455.2738 | 908.533 | 908.5331 | 43.4 | VPAGLPDLK | |
| --- | --- | --- | --- | --- | --- |
| 455.2739 | 908.5332 | 908.5331 | 42.93 | VPAGLPDLK | |
| 504.2911 | 1006.568 | 1006.567 | 40.46 | LGLGHNQIR | |
| 504.7833 | 1007.552 | 1007.551 | 48.5 | LGLGHNQIR | Deamidated (NQ) |
| 438.2536 | 1311.739 | 1311.74 | 44.85 | IQAIELEDLLR | |
| 656.8776 | 1311.741 | 1311.74 | 48.83 | IQAIELEDLLR | |
| 747.8749 | 1493.735 | 1493.734 | 59.35 | VGVNDFCPVGFGVK | |
| 748.3671 | 1494.72 | 1494.718 | 40.23 | VGVNDFCPVGFGVK | Deamidated (NQ) |
| 791.46 | 1580.905 | 1580.904 | 55.5 | GLQHLYALVLVNNK | |
| 797.9115 | 1593.808 | 1593.807 | 44.17 | MIENGSLSFLPTLR | Deamidated (NQ); Oxidation (M) |
| 1014.562 | 2027.109 | 2027.105 | 40.08 | NHLVEIPPNLPSSLVELR | Deamidated (NQ) |
| 1014.562 | 2027.109 | 2027.105 | 49.29 | NHLVEIPPNLPSSLVELR | Deamidated (NQ) |
| 1143.577 | 2285.139 | 2285.139 | 46.44 | EISPDTTLLDLQNNDISELR | |

| Lumican OS=Bos taurus GN=LUM PE=1 SV=1 | 196 |
| --- | --- |

| 499.2766 | 996.5386 | 996.5392 | 46.56 | FSALQYLR | |
| --- | --- | --- | --- | --- | --- |
| 499.277 | 996.5394 | 996.5392 | 45.7 | FSALQYLR | |
| 499.2771 | 996.5396 | 996.5392 | 54.75 | FSALQYLR | |
| 499.7687 | 997.5228 | 997.5233 | 46.27 | FSALQYLR | Deamidated (NQ) |
| 844.9142 | 1687.814 | 1687.813 | 48.73 | SLEYLDLSFNQMTK | |
| 845.4063 | 1688.798 | 1688.797 | 45.35 | SLEYLDLSFNQMTK | Deamidated (NQ) |
| 852.9125 | 1703.81 | 1703.808 | 68.4 | SLEYLDLSFNQMTK | Oxidation (M) |
| 853.4021 | 1704.79 | 1704.792 | 63.82 | SLEYLDLSFNQMTK | Deamidated (NQ); Oxidation (K) |
| 653.0378 | 1956.092 | 1956.093 | 43.7 | LPSGLPVSLLTLYLDNNK | |

| Hemoglobin subunit beta OS=Bos taurus GN=HBB PE=1 SV=1 | 192 |
| --- | --- |

| 475.7581 | 949.5016 | 949.5022 | 41.66 | AAVTAFWGK | |
| --- | --- | --- | --- | --- | --- |
| 422.6143 | 1264.821 | 1264.823 | 44.51 | LLGNVLVVVLAR | |
| 633.4183 | 1264.822 | 1264.823 | 85.32 | LLGNVLVVVLAR | |
| 1045.483 | 2088.951 | 2088.946 | 66.94 | FFESFGDLSTADAVMNNPK | |
| 1053.479 | 2104.943 | 2104.941 | 59.5 | FFESFGDLSTADAVMNNPK | Oxidation (M) |

| Complement C3 OS=Bos taurus GN=C3 PE=1 SV=2 | 174 |
| --- | --- |

| 594.3431 | 1186.672 | 1186.671 | 55.25 | ILWESASLLR |
| --- | --- | --- | --- | --- |
| 606.3305 | 1210.646 | 1210.646 | 52.64 | KGYTQQLAFR |
| 634.8665 | 1267.718 | 1267.718 | 46.56 | LVAYYTLINAK |
| 686.416 | 1370.817 | 1370.817 | 42.47 | SSVAVPYVIVPLK |
| 941.0281 | 1880.042 | 1880.041 | 77.44 | LYNVEATSYALLALLAR |

| Tetranectin OS=Bos taurus GN=CLEC3B PE=2 SV=1 | 140 |
| --- | --- |

| 542.2867 | 1082.559 | 1082.558 | 49.07 | CFLAFVQAK |
| --- | --- | --- | --- | --- |
| 764.9335 | 1527.852 | 1527.851 | 75.7 | TQLDSLAQEVALLK |
| 1121.536 | 2241.057 | 2241.055 | 66.9 | GGTLGTPQTGSENDALYEYLR |

| Collagen alpha-1(I) chain OS=Bos taurus GN=COL1A1 PE=1 SV=3 | 139 |
| --- | --- |

| 426.2166 | 850.4186 | 850.4185 | 44.05 | GFSGLDGAK | |
| --- | --- | --- | --- | --- | --- |
| 426.2166 | 850.4186 | 850.4185 | 40.26 | GFSGLDGAK | |
| 426.2168 | 850.419 | 850.4185 | 40.01 | GFSGLDGAK | |
| 550.2785 | 1098.542 | 1098.542 | 60.01 | GADGAPGKDGVR | |
| 594.3227 | 1186.631 | 1186.631 | 52.59 | SLSQQIENIR | |
| 728.903 | 1455.791 | 1455.793 | 55.13 | ALLLQGSNEIEIR | Deamidated (NQ) |
| 848.9213 | 1695.828 | 1695.825 | 41.37 | NSVAYMDQQTGNLKK | |
| 1040.476 | 2078.937 | 2078.937 | 43.98 | SGEYWIDPNQGCNLDAIK | |

| Nucleobindin-1 OS=Bos taurus GN=NUCB1 PE=2 SV=1 | 133 |
| --- | --- |

| 552.2893 | 1102.564 | 1102.562 | 42.7 | LSQETEALGR |
| --- | --- | --- | --- | --- |
| 636.8622 | 1271.71 | 1271.709 | 81.99 | DLELLIQTATR |
| 736.8805 | 1471.746 | 1471.745 | 59.54 | ELQQAVLQMEQR |
| 966.9952 | 1931.976 | 1931.974 | 41.4 | YLQEVINVLETDGHFR |

| Secreted phosphoprotein 24 OS=Bos taurus GN=SPP2 PE=1 SV=2 | 131 |
| --- | --- |

| 538.276 | 1074.537 | 1074.536 | 42.06 | RYSNPWPR | |
| --- | --- | --- | --- | --- | --- |
| 706.8563 | 1411.698 | 1411.698 | 48.98 | VNSQSLSPYLFR | 2 Deamidated (NQ) |
| 723.8619 | 1445.709 | 1445.709 | 61.44 | MSAEQVQNVWVR | |
| 942.4407 | 1882.867 | 1882.862 | 58.82 | VNALDEDSLTMDLEFR | Oxidation (M) |

| Thrombospondin-1 OS=Bos taurus GN=THBS1 PE=2 SV=2 | 108 |
| --- | --- |

| 495.3103 | 988.606 | 988.6069 | 55.31 | GFLLLASLR | |
| --- | --- | --- | --- | --- | --- |
| 531.7827 | 1061.551 | 1061.551 | 42.72 | FQDLVDAVR | |
| 809.4025 | 1616.79 | 1616.791 | 43.66 | GGVNDNFQGVLQNVR | Deamidated (NQ) |
| 809.4033 | 1616.792 | 1616.791 | 63.34 | GGVNDNFQGVLQNVR | Deamidated (NQ) |

| Collagen alpha-2(I) chain OS=Bos taurus GN=COL1A2 PE=1 SV=2 | 106 |
| --- | --- |

| 393.1987 | 784.3828 | 784.3828 | 40.61 | GDQGPVGR | |
| --- | --- | --- | --- | --- | --- |
| 464.2302 | 926.4458 | 926.4458 | 44.72 | GHNGLDGLK | Deamidated (NQ); Oxidation (K) |
| 464.2302 | 926.4458 | 926.4458 | 40.97 | GHNGLDGLK | Deamidated (NQ); Oxidation (K) |
| 804.3455 | 1606.676 | 1606.676 | 52.25 | VYCDFSTGETCIR | |

| Collagen alpha-2(XI) chain OS=Bos taurus GN=COL11A2 PE=3 SV=1 | 95 |
| --- | --- |

| 416.7556 | 831.4966 | 831.4967 | 41.91 | GVIIFGAR |  |
| --- | --- | --- | --- | --- | --- |
| 511.2874 | 1020.56 | 1020.56 | 46.18 | DFSLLTAVR | |
| 671.0538 | 2010.14 | 2010.137 | 42.57 | ARPGLQAPLLTLYSAQGVR | |

| Apolipoprotein A-I OS=Bos taurus GN=APOA1 PE=1 SV=3 | 92 |
| --- | --- |

| 699.8486 | 1397.683 | 1397.683 | 61.22 | DYVAQFEASALGK | |
| --- | --- | --- | --- | --- | --- |
| 788.9153 | 1575.816 | 1575.814 | 49.02 | LLDNWDTLASTLSK | |
|  |  |  |  |  |  |

| Asporin OS=Bos taurus GN=ASPN PE=2 SV=1 | 87 |
| --- | --- |

| 810.9518 | 1619.889 | 1619.888 | 62.05 | KSLYSAISLSNNPVK | |
| --- | --- | --- | --- | --- | --- |
| 839.4651 | 1676.916 | 1676.914 | 42.03 | YLQIIFLHSNSITK | Deamidated (NQ) |

| Vitamin K-dependent protein S OS=Bos taurus GN=PROS1 PE=1 SV=1 | 73 |
| --- | --- |

| 509.7345 | 1017.454 | 1017.456 | 51.06 | FSAEFDFR |
| --- | --- | --- | --- | --- |
| 509.735 | 1017.455 | 1017.456 | 45.4 | FSAEFDFR |
| 536.7657 | 1071.517 | 1071.517 | 40.14 | YLGCLGSFR |

| Vitamin D-binding protein OS=Bos taurus GN=GC PE=2 SV=1 | 62 |
| --- | --- |

| 467.7645 | 933.5144 | 933.5139 | 44.15 | KLCMAALK |
| --- | --- | --- | --- | --- |
| 691.8697 | 1381.725 | 1381.724 | 49.19 | VLDQYIFELSR |

| Vimentin OS=Bos taurus GN=VIM PE=1 SV=3 | 61 |
| --- | --- |

| 736.874 | 1471.733 | 1471.731 | 40.87 | TLYTSSPGGVYATR |
| --- | --- | --- | --- | --- |
| 767.4296 | 1532.845 | 1532.845 | 45.81 | KVESLQEEIAFLK |

**KC2**

| Collagen alpha-2(I) chain OS=Bos taurus GN=COL1A2 PE=1 SV=2 | 1284 |
| --- | --- |

| **pep_exp_mz** | **pep_exp_mr** | **pep_calc_mr** | **pep_score** | **pep_seq** | **pep_var_mod** |
| --- | --- | --- | --- | --- | --- |
| 405.2226 | 808.4306 | 808.4304 | 40.16 | GHAGLAGAR | |
| 435.2275 | 868.4404 | 868.4403 | 47.82 | GPSGPQGIR | Deamidated (NQ) |
| 446.7537 | 891.4928 | 891.4926 | 42.58 | PGPIGPAGAR | |
| 448.2351 | 894.4556 | 894.4559 | 50.38 | GPAGPSGPAGK | |
| 454.7511 | 907.4876 | 907.4876 | 41.45 | PGPIGPAGAR | Oxidation (P) |
| 459.7266 | 917.4386 | 917.4389 | 48.73 | AGVMGPAGSR | Oxidation (M) |
| 467.7242 | 933.4338 | 933.4338 | 40.89 | AGVMGPAGSR | Oxidation (M); Oxidation (P) |
| 596.8438 | 1191.673 | 1191.672 | 57.1 | IGQPGAVGPAGIR | |
| 597.3354 | 1192.656 | 1192.656 | 56.87 | IGQPGAVGPAGIR | Deamidated (NQ) |
| 604.841 | 1207.667 | 1207.667 | 41.5 | IGQPGAVGPAGIR | Oxidation (P) |
| 604.8416 | 1207.669 | 1207.667 | 52.24 | IGQPGAVGPAGIR | Oxidation (P) |
| 605.3326 | 1208.651 | 1208.651 | 55.15 | IGQPGAVGPAGIR | Deamidated (NQ); Oxidation (P) |
| 605.3327 | 1208.651 | 1208.651 | 50.93 | IGQPGAVGPAGIR | Deamidated (NQ); Oxidation (P) |
| 605.333 | 1208.651 | 1208.651 | 42.66 | IGQPGAVGPAGIR | Deamidated (NQ); Oxidation (P) |
| 605.3335 | 1208.652 | 1208.651 | 53.59 | IGQPGAVGPAGIR | Deamidated (NQ); Oxidation (P) |
| 605.3335 | 1208.652 | 1208.651 | 52.11 | IGQPGAVGPAGIR | Deamidated (NQ); Oxidation (P) |
| 634.3416 | 1266.669 | 1266.668 | 50.62 | GIPGPVGAAGATGAR | Oxidation (P) |
| 634.342 | 1266.669 | 1266.668 | 74.11 | GIPGPVGAAGATGAR | Oxidation (P) |
| 634.3423 | 1266.67 | 1266.668 | 57.06 | GIPGPVGAAGATGAR | Oxidation (P) |
| 644.3205 | 1286.626 | 1286.626 | 68.95 | GFPGSPGNIGPAGK | 2 Oxidation (P) |
| 714.3679 | 1426.721 | 1426.721 | 44.38 | GIPGEFGLPGPAGAR | 2 Oxidation (P) |
| 729.3407 | 1456.667 | 1456.67 | 62.65 | GDGGPPGATGFPGAAGR | Oxidation (P) |
| 737.34 | 1472.665 | 1472.665 | 74.42 | GDGGPPGATGFPGAAGR | 2 Oxidation (P) |
| 737.3403 | 1472.666 | 1472.665 | 53.72 | GDGGPPGATGFPGAAGR | 2 Oxidation (P) |
| 746.8502 | 1491.686 | 1491.684 | 51.94 | SGETGASGPPGFVGEK | Oxidation (P) |
| 746.8503 | 1491.686 | 1491.684 | 41.81 | SGETGASGPPGFVGEK | Oxidation (P) |
| 766.8952 | 1531.776 | 1531.774 | 52.21 | GEPGPAGAVGPAGAVGPR | Oxidation (P) |
| 766.8956 | 1531.777 | 1531.774 | 53.34 | GEPGPAGAVGPAGAVGPR | Oxidation (P) |
| 790.8882 | 1579.762 | 1579.759 | 53.23 | GPPGESGAAGPTGPIGSR | Oxidation (P) |
| 539.606 | 1615.796 | 1615.795 | 56.74 | GELGPVGNPGPAGPAGPR | Deamidated (NQ); Oxidation (P) |
| 808.9063 | 1615.798 | 1615.795 | 40.94 | GELGPVGNPGPAGPAGPR | Deamidated (NQ); Oxidation (P) |
| 824.9186 | 1647.823 | 1647.822 | 47.33 | GSTGEIGPAGPPGPPGLR | 2 Oxidation (P) |
| 824.9186 | 1647.823 | 1647.822 | 51.37 | GSTGEIGPAGPPGPPGLR | 2 Oxidation (P) |
| 550.2817 | 1647.823 | 1647.822 | 50.78 | GSTGEIGPAGPPGPPGLR | 2 Oxidation (P) |
| 911.9205 | 1821.826 | 1821.824 | 57.61 | GPPGNVGNPGVNGAPGEAGR | Deamidated (NQ); 3 Oxidation (P) |
| 912.4126 | 1822.811 | 1822.808 | 56.08 | GPPGNVGNPGVNGAPGEAGR | 2 Deamidated (NQ); 3 Oxidation (P) |
| 912.9043 | 1823.794 | 1823.792 | 53.07 | GPPGNVGNPGVNGAPGEAGR | 3 Deamidated (NQ); 3 Oxidation (P) |
| 641.6489 | 1921.925 | 1921.924 | 43.33 | GERGPPGESGAAGPTGPIGSR | Oxidation (P) |
| 1058.065 | 2114.115 | 2114.112 | 42.99 | GLPGVAGSVGEPGPLGIAGPPGAR | 2 Oxidation (P) |
| 711.0436 | 2130.109 | 2130.107 | 40 | GLPGVAGSVGEPGPLGIAGPPGAR | 3 Oxidation (P) |
| 1066.062 | 2130.109 | 2130.107 | 69.89 | GLPGVAGSVGEPGPLGIAGPPGAR | 3 Oxidation (P) |
| 1066.062 | 2130.109 | 2130.107 | 72.14 | GLPGVAGSVGEPGPLGIAGPPGAR | 3 Oxidation (P) |
| 1066.062 | 2130.109 | 2130.107 | 40.68 | GLPGVAGSVGEPGPLGIAGPPGAR | 3 Oxidation (P) |
| 1066.062 | 2130.109 | 2130.107 | 62.26 | GLPGVAGSVGEPGPLGIAGPPGAR | 3 Oxidation (P) |
| 1066.062 | 2130.109 | 2130.107 | 54.86 | GLPGVAGSVGEPGPLGIAGPPGAR | 3 Oxidation (P) |
| 711.0439 | 2130.11 | 2130.107 | 41.33 | GLPGVAGSVGEPGPLGIAGPPGAR | 3 Oxidation (P) |
| 711.0444 | 2130.111 | 2130.107 | 48.03 | GLPGVAGSVGEPGPLGIAGPPGAR | 3 Oxidation (P) |
| 1074.061 | 2146.107 | 2146.102 | 43.54 | GLPGVAGSVGEPGPLGIAGPPGAR | 4 Oxidation (P) |
| 1216.11 | 2430.205 | 2430.203 | 42.23 | GEVGLPGLSGPVGPPGNPGANGLPGAK | Deamidated (NQ); 4 Oxidation (P) |
| 1224.108 | 2446.201 | 2446.198 | 47.25 | GEVGLPGLSGPVGPPGNPGANGLPGAK | Deamidated (NQ); Oxidation (K); 4 Oxidation (P) |
| 1287.137 | 2572.259 | 2572.256 | 63.02 | GSDGSVGPVGPAGPIGSAGPPGFPGAPGPK | Oxidation (K); Oxidation (P) |
| 955.471 | 2863.391 | 2863.385 | 64.55 | GPKGENGPVGPTGPVGAAGPSGPNGPPGPAGSR | Deamidated (NQ); Oxidation (P) |
| 955.7995 | 2864.377 | 2864.369 | 44.84 | GPKGENGPVGPTGPVGAAGPSGPNGPPGPAGSR | 2 Deamidated (NQ); Oxidation (P) |
| 960.8021 | 2879.385 | 2879.38 | 49.73 | GPKGENGPVGPTGPVGAAGPSGPNGPPGPAGSR | Deamidated (NQ); Oxidation (K); Oxidation (P) |
| 1056.519 | 3166.535 | 3166.528 | 65.26 | GTKGPKGENGPVGPTGPVGAAGPSGPNGPPGPAGSR | 2 Deamidated (NQ); Oxidation (K); Oxidation (P) |

| Collagen alpha-1(I) chain OS=Bos taurus GN=COL1A1 PE=1 SV=3 | 868 |
| --- | --- |

| 419.2142 | 836.4138 | 836.4141 | 41.28 | GPAGPQGPR | Deamidated (NQ) |
| --- | --- | --- | --- | --- | --- |
| 444.2144 | 886.4142 | 886.4145 | 43.13 | GSEGPQGVR | Deamidated (NQ) |
| 472.2275 | 942.4404 | 942.4407 | 71.65 | GPPGSAGSPGK | 2 Oxidation (P) |
| 473.2228 | 944.431 | 944.4312 | 43.69 | QGPSGASGER | |
| 365.86 | 1094.558 | 1094.558 | 45.73 | GRPGAPGPAGAR | 2 Oxidation (P) |
| 553.2913 | 1104.568 | 1104.568 | 42.24 | GVQGPPGPAGPR | Oxidation (P) |
| 553.7832 | 1105.552 | 1105.552 | 47.92 | GVQGPPGPAGPR | Deamidated (NQ); Oxidation (P) |
| 589.2878 | 1176.561 | 1176.56 | 51.63 | GQAGVMGFPGPK | Oxidation (M); Oxidation (P) |
| 589.7803 | 1177.546 | 1177.544 | 53.96 | GQAGVMGFPGPK | Deamidated (NQ); Oxidation (M); Oxidation (P) |
| 597.7764 | 1193.538 | 1193.539 | 44.58 | GQAGVMGFPGPK | Deamidated (NQ); Oxidation (K); Oxidation (M); Oxidation (P) |
| 629.7995 | 1257.584 | 1257.584 | 44.75 | GLTGSPGSPGPDGK | 2 Oxidation (P) |
| 633.2661 | 1264.518 | 1264.517 | 45.1 | GDKGETGEQGDR | Deamidated (NQ); Oxidation (K) |
| 641.3134 | 1280.612 | 1280.611 | 44.41 | GEAGPSGPAGPTGAR | |
| 664.8282 | 1327.642 | 1327.641 | 40 | GFPGLPGPSGEPGK | 2 Oxidation (P) |
| 664.8284 | 1327.642 | 1327.641 | 41.46 | GFPGLPGPSGEPGK | 2 Oxidation (P) |
| 666.8318 | 1331.649 | 1331.647 | 78.71 | GPSGPQGPSGPPGPK | Oxidation (P) |
| 667.3234 | 1332.632 | 1332.631 | 56.64 | GPSGPQGPSGPPGPK | Deamidated (NQ); Oxidation (P) |
| 667.3236 | 1332.633 | 1332.631 | 77.25 | GPSGPQGPSGPPGPK | Deamidated (NQ); Oxidation (P) |
| 672.8256 | 1343.637 | 1343.636 | 52.91 | GFPGLPGPSGEPGK | 3 Oxidation (P) |
| 675.3208 | 1348.627 | 1348.626 | 54.25 | GPSGPQGPSGPPGPK | Deamidated (NQ); 2 Oxidation (P) |
| 675.321 | 1348.627 | 1348.626 | 45.74 | GPSGPQGPSGPPGPK | Deamidated (NQ); 2 Oxidation (P) |
| 718.3452 | 1434.676 | 1434.674 | 45.09 | GEPGPAGLPGPPGER | 3 Oxidation (P) |
| 730.3505 | 1458.686 | 1458.685 | 70.07 | GSAGPPGATGFPGAAGR | 2 Oxidation (P) |
| 730.3508 | 1458.687 | 1458.685 | 69.92 | GSAGPPGATGFPGAAGR | 2 Oxidation (P) |
| 730.3509 | 1458.687 | 1458.685 | 64.34 | GSAGPPGATGFPGAAGR | 2 Oxidation (P) |
| 730.351 | 1458.687 | 1458.685 | 62.47 | GSAGPPGATGFPGAAGR | 2 Oxidation (P) |
| 521.5972 | 1561.77 | 1561.774 | 49.35 | DGLNGLPGPIGPPGPR | Deamidated (NQ); 3 Oxidation (P) |
| 793.3906 | 1584.767 | 1584.765 | 46.46 | GANGAPGIAGAPGFPGAR | 3 Oxidation (P) |
| 809.3986 | 1616.783 | 1616.78 | 54.08 | GFSGLDGAKGDAGPAGPK | Oxidation (K) |
| 845.8937 | 1689.773 | 1689.771 | 44.14 | DGEAGAQGPPGPAGPAGER | |
| 853.8919 | 1705.769 | 1705.766 | 70.74 | DGEAGAQGPPGPAGPAGER | Oxidation (P) |
| 854.3826 | 1706.751 | 1706.75 | 56.01 | DGEAGAQGPPGPAGPAGER | Deamidated (NQ); Oxidation (P) |
| 908.9391 | 1815.864 | 1815.857 | 52.1 | GPPGPMGPPGLAGPPGESGR | Oxidation (M); Oxidation (P) |
| 1037.503 | 2072.991 | 2072.988 | 75.47 | GAPGADGPAGAPGTPGPQGIAGQR | Deamidated (NQ); Oxidation (P) |
| 1045.991 | 2089.967 | 2089.967 | 44.26 | GAPGADGPAGAPGTPGPQGIAGQR | 2 Deamidated (NQ); 2 Oxidation (P) |
| 1045.992 | 2089.969 | 2089.967 | 60.3 | GAPGADGPAGAPGTPGPQGIAGQR | 2 Deamidated (NQ); 2 Oxidation (P) |
| 1053.99 | 2105.965 | 2105.961 | 51.88 | GAPGADGPAGAPGTPGPQGIAGQR | 2 Deamidated (NQ); 3 Oxidation (P) |
| 1085.034 | 2168.053 | 2168.05 | 52.13 | GETGPAGPPGAPGAPGAPGPVGPAGK | 3 Oxidation (P) |
| 1162.071 | 2322.127 | 2322.124 | 49.9 | GDAGPPGPAGPAGPPGPIGNVGAPGPK | 4 Oxidation (P) |
| 1244.111 | 2486.207 | 2486.204 | 40.81 | GPPGSAGSPGKDGLNGLPGPIGPPGPR | Deamidated (NQ); 5 Oxidation (P) |
| 1274.609 | 2547.203 | 2547.199 | 59.51 | GNDGATGAAGPPGPTGPAGPPGFPGAVGAK | 3 Oxidation (P) |
| 902.7519 | 2705.234 | 2705.232 | 46.32 | GFSGLQGPPGPPGSPGEQGPSGASGPAGPR | Deamidated (NQ); 3 Oxidation (P) |

**KC6**

| Collagen alpha-2(I) chain OS=Bos taurus GN=COL1A2 PE=1 SV=2 | 962 |
| --- | --- |

| **pep_exp_mz** | **pep_exp_mr** | **pep_calc_mr** | **pep_score** | **pep_seq** | **pep_var_mod** |
| --- | --- | --- | --- | --- | --- |
| 393.2168 | 784.419 | 784.4192 | 50.59 | GATGPAGVR | |
| 434.7354 | 867.4562 | 867.4563 | 40.02 | VGAPGPAGAR | Oxidation (P) |
| 434.7354 | 867.4562 | 867.4563 | 41.87 | GPSGPQGIR | |
| 435.2275 | 868.4404 | 868.4403 | 41.63 | GPSGPQGIR | Deamidated (NQ) |
| 446.7537 | 891.4928 | 891.4926 | 46.62 | PGPIGPAGAR | |
| 591.8096 | 1181.605 | 1181.604 | 40.46 | EGPVGLPGIDGR | Oxidation (P) |
| 398.2312 | 1191.672 | 1191.672 | 44.94 | IGQPGAVGPAGIR | |
| 597.3354 | 1192.656 | 1192.656 | 46.46 | IGQPGAVGPAGIR | Deamidated (NQ) |
| 597.3358 | 1192.657 | 1192.656 | 51.24 | IGQPGAVGPAGIR | Deamidated (NQ) |
| 601.7884 | 1201.562 | 1201.562 | 43.37 | GEPGNIGFPGPK | Deamidated (NQ); 2 Oxidation (P) |
| 604.8416 | 1207.669 | 1207.667 | 44.02 | IGQPGAVGPAGIR | Oxidation (P) |
| 604.8416 | 1207.669 | 1207.667 | 51.79 | IGQPGAVGPAGIR | Oxidation (P) |
| 403.8908 | 1208.651 | 1208.651 | 45.91 | IGQPGAVGPAGIR | Deamidated (NQ); Oxidation (P) |
| 605.3329 | 1208.651 | 1208.651 | 55.94 | IGQPGAVGPAGIR | Deamidated (NQ); Oxidation (P) |
| 605.3334 | 1208.652 | 1208.651 | 52.77 | IGQPGAVGPAGIR | Deamidated (NQ); Oxidation (P) |
| 404.2144 | 1209.621 | 1209.621 | 40.65 | AGEKGHAGLAGAR | Oxidation (K) |
| 634.3419 | 1266.669 | 1266.668 | 53.76 | GIPGPVGAAGATGAR | Oxidation (P) |
| 634.3422 | 1266.67 | 1266.668 | 64.67 | GIPGPVGAAGATGAR | Oxidation (P) |
| 644.3207 | 1286.627 | 1286.626 | 46.5 | GFPGSPGNIGPAGK | 2 Oxidation (P) |
| 644.8127 | 1287.611 | 1287.61 | 42.78 | GFPGSPGNIGPAGK | Deamidated (NQ); 2 Oxidation (P) |
| 714.3679 | 1426.721 | 1426.721 | 45.33 | GIPGEFGLPGPAGAR | 2 Oxidation (P) |
| 714.3683 | 1426.722 | 1426.721 | 43.06 | GIPGEFGLPGPAGAR | 2 Oxidation (P) |
| 729.343 | 1456.671 | 1456.67 | 47.32 | GDGGPPGATGFPGAAGR | Oxidation (P) |
| 737.3397 | 1472.665 | 1472.665 | 50.57 | GDGGPPGATGFPGAAGR | 2 Oxidation (P) |
| 737.3401 | 1472.666 | 1472.665 | 64.09 | GDGGPPGATGFPGAAGR | 2 Oxidation (P) |
| 737.3403 | 1472.666 | 1472.665 | 55.85 | GDGGPPGATGFPGAAGR | 2 Oxidation (P) |
| 746.8503 | 1491.686 | 1491.684 | 60.71 | SGETGASGPPGFVGEK | Oxidation (P) |
| 746.8506 | 1491.687 | 1491.684 | 51.39 | SGETGASGPPGFVGEK | Oxidation (P) |
| 766.8962 | 1531.778 | 1531.774 | 61.13 | GEPGPAGAVGPAGAVGPR | Oxidation (P) |
| 521.6143 | 1561.821 | 1561.821 | 42.89 | GAAGLPGVAGAPGLPGPR | 3 Oxidation (P) |
| 781.9186 | 1561.823 | 1561.821 | 47.63 | GAAGLPGVAGAPGLPGPR | 3 Oxidation (P) |
| 790.8878 | 1579.761 | 1579.759 | 69.01 | GPPGESGAAGPTGPIGSR | Oxidation (P) |
| 790.8882 | 1579.762 | 1579.759 | 43.75 | GPPGESGAAGPTGPIGSR | Oxidation (P) |
| 851.3695 | 1700.724 | 1700.724 | 45.8 | GEPGAPGENGTPGQTGAR | Deamidated (NQ); 3 Oxidation (P) |
| 911.9207 | 1821.827 | 1821.824 | 45.79 | GPPGNVGNPGVNGAPGEAGR | Deamidated (NQ); 3 Oxidation (P) |
| 912.4131 | 1822.812 | 1822.808 | 68.25 | GPPGNVGNPGVNGAPGEAGR | 2 Deamidated (NQ); 3 Oxidation (P) |
| 912.9032 | 1823.792 | 1823.792 | 47.65 | GPPGNVGNPGVNGAPGEAGR | 3 Deamidated (NQ); 3 Oxidation (P) |
| 1026.494 | 2050.973 | 2050.971 | 40.84 | GEVGPAGPNGFAGPAGAAGQPGAK | Deamidated (NQ); Oxidation (P) |
| 1026.494 | 2050.973 | 2050.971 | 41.95 | GEVGPAGPNGFAGPAGAAGQPGAK | Deamidated (NQ); Oxidation (P) |
| 1026.986 | 2051.957 | 2051.955 | 68.71 | GEVGPAGPNGFAGPAGAAGQPGAK | 2 Deamidated (NQ); Oxidation (P) |
| 1066.062 | 2130.109 | 2130.107 | 70.96 | GLPGVAGSVGEPGPLGIAGPPGAR | 3 Oxidation (P) |
| 1215.618 | 2429.221 | 2429.219 | 57.01 | GEVGLPGLSGPVGPPGNPGANGLPGAK | 4 Oxidation (P) |
| 961.1284 | 2880.363 | 2880.364 | 47.76 | GPKGENGPVGPTGPVGAAGPSGPNGPPGPAGSR | 2 Deamidated (NQ); Oxidation (K); Oxidation (P) |

| Collagen alpha-1(I) chain OS=Bos taurus GN=COL1A1 PE=1 SV=3 | 754 |
| --- | --- |

| 444.2142 | 886.4138 | 886.4145 | 54.59 | GSEGPQGVR | Deamidated (NQ) |
| --- | --- | --- | --- | --- | --- |
| 589.2876 | 1176.561 | 1176.56 | 43.44 | GQAGVMGFPGPK | Oxidation (M); Oxidation (P) |
| 589.7802 | 1177.546 | 1177.544 | 59.9 | GQAGVMGFPGPK | Deamidated (NQ); Oxidation (M); Oxidation (P) |
| 596.8199 | 1191.625 | 1191.625 | 43.17 | GVPGPPGAVGPAGK | 2 Oxidation (P) |
| 596.82 | 1191.625 | 1191.625 | 43.04 | GVPGPPGAVGPAGK | 2 Oxidation (P) |
| 621.8019 | 1241.589 | 1241.589 | 48.14 | GLTGSPGSPGPDGK | Oxidation (P) |
| 621.8024 | 1241.59 | 1241.589 | 42.46 | GLTGSPGSPGPDGK | Oxidation (P) |
| 629.7995 | 1257.584 | 1257.584 | 45.76 | GLTGSPGSPGPDGK | 2 Oxidation (P) |
| 629.8 | 1257.585 | 1257.584 | 42.24 | GLTGSPGSPGPDGK | 2 Oxidation (P) |
| 629.8008 | 1257.587 | 1257.584 | 51.8 | GLTGSPGSPGPDGK | 2 Oxidation (P) |
| 664.8279 | 1327.641 | 1327.641 | 42.72 | GFPGLPGPSGEPGK | 2 Oxidation (P) |
| 664.8283 | 1327.642 | 1327.641 | 47.22 | GFPGLPGPSGEPGK | 2 Oxidation (P) |
| 667.3234 | 1332.632 | 1332.631 | 68.11 | GPSGPQGPSGPPGPK | Deamidated (NQ); Oxidation (P) |
| 718.3447 | 1434.675 | 1434.674 | 47.11 | GEPGPAGLPGPPGER | 3 Oxidation (P) |
| 730.3505 | 1458.686 | 1458.685 | 85.1 | GSAGPPGATGFPGAAGR | 2 Oxidation (P) |
| 730.351 | 1458.687 | 1458.685 | 69.06 | GSAGPPGATGFPGAAGR | 2 Oxidation (P) |
| 780.9113 | 1559.808 | 1559.806 | 51.65 | GETGPAGPAGPIGPVGAR | |
| 781.8939 | 1561.773 | 1561.774 | 40.35 | DGLNGLPGPIGPPGPR | Deamidated (NQ); 3 Oxidation (P) |
| 793.3903 | 1584.766 | 1584.765 | 52.2 | GANGAPGIAGAPGFPGAR | 3 Oxidation (P) |
| 793.8816 | 1585.749 | 1585.749 | 46.94 | GANGAPGIAGAPGFPGAR | Deamidated (NQ); 3 Oxidation (P) |
| 793.8826 | 1585.751 | 1585.749 | 44.16 | GANGAPGIAGAPGFPGAR | Deamidated (NQ); 3 Oxidation (P) |
| 828.4036 | 1654.793 | 1654.791 | 56.14 | GSPGEAGRPGEAGLPGAK | 3 Oxidation (P) |
| 853.8909 | 1705.767 | 1705.766 | 63.04 | DGEAGAQGPPGPAGPAGER | Oxidation (P) |
| 854.3835 | 1706.752 | 1706.75 | 45.19 | DGEAGAQGPPGPAGPAGER | Deamidated (NQ); Oxidation (P) |
| 879.8719 | 1757.729 | 1757.728 | 46.47 | GEPGSPGENGAPGQMGPR | Oxidation (M); 3 Oxidation (P) |
| 880.3638 | 1758.713 | 1758.712 | 48.06 | GEPGSPGENGAPGQMGPR | Deamidated (NQ); Oxidation (M); 3 Oxidation (P) |
| 1037.011 | 2072.007 | 2072.004 | 63.09 | GAPGADGPAGAPGTPGPQGIAGQR | Oxidation (P) |
| 1045.009 | 2088.003 | 2087.999 | 58.63 | GAPGADGPAGAPGTPGPQGIAGQR | 2 Oxidation (P) |
| 1045.5 | 2088.985 | 2088.983 | 52.38 | GAPGADGPAGAPGTPGPQGIAGQR | Deamidated (NQ); 2 Oxidation (P) |
| 1045.991 | 2089.967 | 2089.967 | 46.93 | GAPGADGPAGAPGTPGPQGIAGQR | 2 Deamidated (NQ); 2 Oxidation (P) |
| 1045.992 | 2089.969 | 2089.967 | 53.8 | GAPGADGPAGAPGTPGPQGIAGQR | 2 Deamidated (NQ); 2 Oxidation (P) |
| 1053.005 | 2103.995 | 2103.993 | 41.05 | GAPGADGPAGAPGTPGPQGIAGQR | 3 Oxidation (P) |
| 1053.497 | 2104.979 | 2104.977 | 55.02 | GAPGADGPAGAPGTPGPQGIAGQR | Deamidated (NQ); 3 Oxidation (P) |
| 1053.99 | 2105.965 | 2105.961 | 41.37 | GAPGADGPAGAPGTPGPQGIAGQR | 2 Deamidated (NQ); 3 Oxidation (P) |

| Alpha-2-HS-glycoprotein OS=Bos taurus GN=AHSG PE=1 SV=2 | 180 |
| --- | --- |

| 1260.167 | 2518.319 | 2518.314 | 87.51 | AQFVPLPVSVSVEFAVAATDCIAK | |
| --- | --- | --- | --- | --- | --- |
| 1260.168 | 2518.321 | 2518.314 | 73.59 | AQFVPLPVSVSVEFAVAATDCIAK | |
| 1006.525 | 3016.553 | 3016.551 | 59.5 | VVHAVEVALATFNAESNGSYLQLVEISR | Deamidated (NQ) |

| Serum albumin OS=Bos taurus GN=ALB PE=1 SV=4 | 98 |
| --- | --- |

| 708.3489 | 1414.683 | 1414.68 | 41.66 | TVMENFVAFVDK | Oxidation (M) |
| --- | --- | --- | --- | --- | --- |
| 740.4033 | 1478.792 | 1478.788 | 66.22 | LGEYGFQNALIVR | |
| 740.8946 | 1479.775 | 1479.772 | 45.86 | LGEYGFQNALIVR | Deamidated (NQ) |

**NS2**

| Collagen alpha-2(I) chain OS=Bos taurus GN=COL1A2 PE=1 SV=2 | 306 |
| --- | --- |

| **pep_exp_mz** | **pep_exp_mr** | **pep_calc_mr** | **pep_score** | **pep_seq** | **pep_var_mod** |
| --- | --- | --- | --- | --- | --- |
| 596.8435 | 1191.672 | 1191.672 | 45.27 | IGQPGAVGPAGIR | |
| 597.2866 | 1192.559 | 1192.559 | 45.76 | AGEDGHPGKPGR | Oxidation (P) |
| 597.3358 | 1192.657 | 1192.656 | 49.6 | IGQPGAVGPAGIR | Deamidated (NQ) |
| 404.2143 | 1209.621 | 1209.621 | 40.45 | AGEKGHAGLAGAR | Oxidation (K) |
| 605.8182 | 1209.622 | 1209.621 | 52.84 | AGEKGHAGLAGAR | Oxidation (K) |
| 644.3202 | 1286.626 | 1286.626 | 65.84 | GFPGSPGNIGPAGK | 2 Oxidation (P) |
| 903.4312 | 1804.848 | 1804.845 | 48.56 | GPPGNVGNPGVNGAPGEAGR | 2 Oxidation (P) |
| 903.9213 | 1805.828 | 1805.829 | 69.12 | GPPGNVGNPGVNGAPGEAGR | Deamidated (NQ); 2 Oxidation (P) |
| 911.9212 | 1821.828 | 1821.824 | 71.48 | GPPGNVGNPGVNGAPGEAGR | Deamidated (NQ); 3 Oxidation (P) |

| Alpha-2-HS-glycoprotein OS=Bos taurus GN=AHSG PE=1 SV=2 | 252 |
| --- | --- |

| 408.7144 | 815.4142 | 815.4137 | 47.68 | ALGGEDVR | |
| --- | --- | --- | --- | --- | --- |
| 577.8118 | 1153.609 | 1153.609 | 55.28 | HTLNQIDSVK | |
| 578.3043 | 1154.594 | 1154.593 | 59.33 | HTLNQIDSVK | Deamidated (NQ) |
| 578.7963 | 1155.578 | 1155.577 | 45.39 | HTLNQIDSVK | 2 Deamidated (NQ) |
| 635.3276 | 1268.641 | 1268.64 | 55.68 | QDGQFSVLFTK | |
| 737.9233 | 1473.832 | 1473.83 | 40.11 | TPIVGQPSIPGGPVR | |
| 707.3398 | 2118.998 | 2118.997 | 44.63 | HTFSGVASVESSSGEAFHVGK | |
| 1260.167 | 2518.319 | 2518.314 | 59.4 | AQFVPLPVSVSVEFAVAATDCIAK | |
| 1006.527 | 3016.559 | 3016.551 | 52.15 | VVHAVEVALATFNAESNGSYLQLVEISR | Deamidated (NQ) |

| Serum albumin OS=Bos taurus GN=ALB PE=1 SV=4 | 190 |
| --- | --- |

| 582.8112 | 1163.608 | 1163.607 | 44.11 | LVNELTEFAK | Deamidated (NQ) |
| --- | --- | --- | --- | --- | --- |
| 740.4023 | 1478.79 | 1478.788 | 46.65 | LGEYGFQNALIVR | |
| 740.8941 | 1479.774 | 1479.772 | 47.15 | LGEYGFQNALIVR | Deamidated (NQ) |
| 862.9214 | 1723.828 | 1723.827 | 76.36 | MPCTEDYLSLILNR | |

| Collagen alpha-1(I) chain OS=Bos taurus GN=COL1A1 PE=1 SV=3 | 175 |
| --- | --- |

| 426.2173 | 850.42 | 850.4185 | 41.1 | GFSGLDGAK | |
| --- | --- | --- | --- | --- | --- |
| 472.2278 | 942.441 | 942.4407 | 42.41 | GPPGSAGSPGK | Oxidation (K); Oxidation (P) |
| 632.7739 | 1263.533 | 1263.533 | 43.56 | GDKGETGEQGDR | Oxidation (K) |
| 793.3909 | 1584.767 | 1584.765 | 62.78 | GANGAPGIAGAPGFPGAR | 3 Oxidation (P) |
| 793.3915 | 1584.768 | 1584.765 | 47.83 | GANGAPGIAGAPGFPGAR | 3 Oxidation (P) |
| 793.8836 | 1585.753 | 1585.749 | 54.69 | GANGAPGIAGAPGFPGAR | Deamidated (NQ); 3 Oxidation (P) |
| 872.3672 | 1742.72 | 1742.717 | 47.14 | GEPGSPGENGAPGQMGPR | Deamidated (NQ); 3 Oxidation (P) |

| Prothrombin OS=Bos taurus GN=F2 PE=1 SV=2 | 146 |
| --- | --- |

| 446.2485 | 890.4824 | 890.4821 | 40.34 | LAVTTSGSR |
| --- | --- | --- | --- | --- |
| 595.2933 | 1188.572 | 1188.572 | 55.04 | YGFYTHVFR |
| 621.8041 | 1241.594 | 1241.593 | 44.17 | ELFESYIEGR |
| 996.4719 | 1990.929 | 1990.927 | 45.05 | SGGSTTSQSPLLETCVPDR |
| 1137.585 | 2273.155 | 2273.151 | 72.44 | IVEGQDAEVGLSPWQVMLFR |

| Pigment epithelium-derived factor OS=Bos taurus GN=SERPINF1 PE=1 SV=1 | 118 |
| --- | --- |

| 780.3984 | 1558.782 | 1558.778 | 48.3 | LAAAVSNFGYDLYR | |
| --- | --- | --- | --- | --- | --- |
| 780.8893 | 1559.764 | 1559.762 | 40.43 | LAAAVSNFGYDLYR | Deamidated (NQ) |
| 992.0004 | 1981.986 | 1981.979 | 43.09 | ALYYDLISNPDIHGTYK | |
| 1037.601 | 2073.187 | 2073.187 | 54.7 | IAQLPLTGSTSIIFFLPQK | |

**AY5**

| Collagen alpha-2(I) chain OS=Bos taurus GN=COL1A2 PE=1 SV=2 | 276 |
| --- | --- |

| **pep_exp_mz** | **pep_exp_mr** | **pep_calc_mr** | **pep_score** | **pep_seq** | **pep_var_mod** |
| --- | --- | --- | --- | --- | --- |
| 393.2165 | 784.4184 | 784.4192 | 45.41 | GATGPAGVR | |
| 591.8092 | 1181.604 | 1181.604 | 53.15 | EGPVGLPGIDGR | Oxidation (P) |
| 605.3334 | 1208.652 | 1208.651 | 52.28 | IGQPGAVGPAGIR | Deamidated (NQ); Oxidation (P) |
| 746.8502 | 1491.686 | 1491.684 | 40.61 | SGETGASGPPGFVGEK | Oxidation (P) |
| 904.4165 | 1806.818 | 1806.813 | 43.76 | GPPGNVGNPGVNGAPGEAGR | 2 Deamidated (NQ); 2 Oxidation (P) |
| 904.9052 | 1807.796 | 1807.797 | 44.13 | GPPGNVGNPGVNGAPGEAGR | 3 Deamidated (NQ); 2 Oxidation (P) |
| 904.9067 | 1807.799 | 1807.797 | 41.44 | GPPGNVGNPGVNGAPGEAGR | 3 Deamidated (NQ); 2 Oxidation (P) |
| 912.4133 | 1822.812 | 1822.808 | 48.78 | GPPGNVGNPGVNGAPGEAGR | 2 Deamidated (NQ); 3 Oxidation (P) |
| 912.9034 | 1823.792 | 1823.792 | 50 | GPPGNVGNPGVNGAPGEAGR | 3 Deamidated (NQ); 3 Oxidation (P) |
| 912.9042 | 1823.794 | 1823.792 | 41.33 | GPPGNVGNPGVNGAPGEAGR | 3 Deamidated (NQ); 3 Oxidation (P) |
| 912.9063 | 1823.798 | 1823.792 | 41.87 | GPPGNVGNPGVNGAPGEAGR | 3 Deamidated (NQ); 3 Oxidation (P) |

| Collagen alpha-1(I) chain OS=Bos taurus GN=COL1A1 PE=1 SV=3 | 91 |
| --- | --- |

| 426.2169 | 850.4192 | 850.4185 | 43.66 | GFSGLDGAK | |
| --- | --- | --- | --- | --- | --- |
| 629.8 | 1257.585 | 1257.584 | 49.36 | GLTGSPGSPGPDGK | 2 Oxidation (P) |
| 793.3911 | 1584.768 | 1584.765 | 50.43 | GANGAPGIAGAPGFPGAR | 3 Oxidation (P) |
| 793.8822 | 1585.75 | 1585.749 | 48.39 | GANGAPGIAGAPGFPGAR | Deamidated (NQ); 3 Oxidation (P) |

**HSB3**

**No matches**

**TSH**

| Collagen alpha-2(I) chain OS=Bos taurus GN=COL1A2 PE=1 SV=2 | 380 |
| --- | --- |

| **pep_exp_mz** | **pep_exp_mr** | **pep_calc_mr** | **pep_score** | **pep_seq** | **pep_var_mod** |
| --- | --- | --- | --- | --- | --- |
| 596.8435 | 1191.672 | 1191.672 | 45.48 | IGQPGAVGPAGIR | |
| 601.7883 | 1201.562 | 1201.562 | 46.56 | GEPGNIGFPGPK | Deamidated (NQ); 2 Oxidation (P) |
| 604.8412 | 1207.668 | 1207.667 | 53.2 | IGQPGAVGPAGIR | Oxidation (P) |
| 605.3329 | 1208.651 | 1208.651 | 43.49 | IGQPGAVGPAGIR | Deamidated (NQ); Oxidation (P) |
| 605.333 | 1208.651 | 1208.651 | 42.82 | IGQPGAVGPAGIR | Deamidated (NQ); Oxidation (P) |
| 634.3414 | 1266.668 | 1266.668 | 40.26 | GIPGPVGAAGATGAR | Oxidation (P) |
| 644.8121 | 1287.61 | 1287.61 | 60.82 | GFPGSPGNIGPAGK | Deamidated (NQ); 2 Oxidation (P) |
| 737.3398 | 1472.665 | 1472.665 | 77.76 | GDGGPPGATGFPGAAGR | 2 Oxidation (P) |
| 746.8499 | 1491.685 | 1491.684 | 46.53 | SGETGASGPPGFVGEK | Oxidation (P) |
| 746.8501 | 1491.686 | 1491.684 | 51.71 | SGETGASGPPGFVGEK | Oxidation (P) |
| 1066.061 | 2130.107 | 2130.107 | 57.74 | GLPGVAGSVGEPGPLGIAGPPGAR | 3 Oxidation (P) |

| Collagen alpha-1(I) chain OS=Bos taurus GN=COL1A1 PE=1 SV=3 | 183 |
| --- | --- |

| 426.2166 | 850.4186 | 850.4185 | 41.71 | GFSGLDGAK | |
| --- | --- | --- | --- | --- | --- |
| 544.7724 | 1087.53 | 1087.53 | 45.04 | GFPGADGVAGPK | Oxidation (P) |
| 621.8018 | 1241.589 | 1241.589 | 40.4 | GLTGSPGSPGPDGK | Oxidation (P) |
| 621.8018 | 1241.589 | 1241.589 | 48.93 | GLTGSPGSPGPDGK | Oxidation (P) |
| 629.7996 | 1257.585 | 1257.584 | 40.1 | GLTGSPGSPGPDGK | 2 Oxidation (P) |
| 730.3505 | 1458.686 | 1458.685 | 69.56 | GSAGPPGATGFPGAAGR | 2 Oxidation (P) |
| 781.8932 | 1561.772 | 1561.774 | 42.69 | DGLNGLPGPIGPPGPR | Deamidated (NQ); 3 Oxidation (P) |
| 793.8821 | 1585.75 | 1585.749 | 44.41 | GANGAPGIAGAPGFPGAR | Deamidated (NQ); 3 Oxidation (P) |
| 793.8823 | 1585.75 | 1585.749 | 48.05 | GANGAPGIAGAPGFPGAR | Deamidated (NQ); 3 Oxidation (P) |
| 793.8823 | 1585.75 | 1585.749 | 43.48 | GANGAPGIAGAPGFPGAR | Deamidated (NQ); 3 Oxidation (P) |

| Serum albumin OS=Bos taurus GN=ALB PE=1 SV=4 | 83 |
| --- | --- |

| 740.894 | 1479.773 | 1479.772 | 58.37 | LGEYGFQNALIVR | Deamidated (NQ) |
| --- | --- | --- | --- | --- | --- |
| 784.376 | 1566.737 | 1566.735 | 48.43 | DAFLGSFLYEYSR | |

**GuHCl Batch 1**

**NF1**

| Collagen alpha-1(I) chain OS=Bos taurus GN=COL1A1 PE=1 SV=3 | 5786 |
| --- | --- |

| **pep_exp_mz** | **pep_exp_mr** | **pep_calc_mr** | **pep_score** | **pep_seq** | **pep_var_mod** |
| --- | --- | --- | --- | --- | --- |
| 392.2219 | 782.4292 | 782.4286 | 42.33 | GAAGLPGPK | Oxidation (P) |
| 418.7223 | 835.43 | 835.4301 | 43.31 | GPAGPQGPR | |
| 426.2165 | 850.4184 | 850.4185 | 41.14 | GFSGLDGAK | |
| 434.2139 | 866.4132 | 866.4134 | 44.68 | GFSGLDGAK | Oxidation (K) |
| 444.2145 | 886.4144 | 886.4145 | 51.89 | GSEGPQGVR | Deamidated (NQ) |
| 449.7584 | 897.5022 | 897.5032 | 50.12 | GVVGLPGQR | Oxidation (P) |
| 449.7585 | 897.5024 | 897.5032 | 46.13 | GVVGLPGQR | Oxidation (P) |
| 449.7587 | 897.5028 | 897.5032 | 46.66 | GVVGLPGQR | Oxidation (P) |
| 449.7587 | 897.5028 | 897.5032 | 40.35 | GVVGLPGQR | Oxidation (P) |
| 544.7717 | 1087.529 | 1087.53 | 52.05 | GFPGADGVAGPK | Oxidation (P) |
| 544.7719 | 1087.529 | 1087.53 | 49.04 | GFPGADGVAGPK | Oxidation (P) |
| 544.772 | 1087.529 | 1087.53 | 53.83 | GFPGADGVAGPK | Oxidation (P) |
| 544.772 | 1087.529 | 1087.53 | 67.5 | GFPGADGVAGPK | Oxidation (P) |
| 544.7722 | 1087.53 | 1087.53 | 56.77 | GFPGADGVAGPK | Oxidation (P) |
| 544.7722 | 1087.53 | 1087.53 | 42.8 | GFPGADGVAGPK | Oxidation (P) |
| 545.2936 | 1088.573 | 1088.573 | 40.11 | GVQGPPGPAGPR | |
| 553.2911 | 1104.568 | 1104.568 | 59.54 | GVQGPPGPAGPR | Oxidation (P) |
| 558.7492 | 1115.484 | 1115.484 | 42.29 | EGAPGAEGSPGR | 2 Oxidation (P) |
| 558.7495 | 1115.484 | 1115.484 | 43.24 | EGAPGAEGSPGR | 2 Oxidation (P) |
| 573.7839 | 1145.553 | 1145.554 | 43.13 | GQAGVMGFPGPK | Deamidated (NQ) |
| 573.7943 | 1145.574 | 1145.575 | 47.06 | GLPGTAGLPGMK | Oxidation (K); 2 Oxidation (P) |
| 581.2893 | 1160.564 | 1160.565 | 50.24 | GQAGVMGFPGPK | Oxidation (K) |
| 581.2895 | 1160.564 | 1160.565 | 64.23 | GQAGVMGFPGPK | Oxidation (P) |
| 581.2897 | 1160.565 | 1160.565 | 73.33 | GQAGVMGFPGPK | Oxidation (P) |
| 581.7814 | 1161.548 | 1161.549 | 46.23 | GQAGVMGFPGPK | Deamidated (NQ); Oxidation (K) |
| 588.8223 | 1175.63 | 1175.63 | 65.63 | GVPGPPGAVGPAGK | Oxidation (P) |
| 589.2864 | 1176.558 | 1176.56 | 44.56 | GQAGVMGFPGPK | 2 Oxidation (P) |
| 589.2867 | 1176.559 | 1176.56 | 65.12 | GQAGVMGFPGPK | Oxidation (K); Oxidation (P) |
| 589.287 | 1176.559 | 1176.56 | 57.35 | GQAGVMGFPGPK | Oxidation (M); Oxidation (P) |
| 589.2871 | 1176.56 | 1176.56 | 65.73 | GQAGVMGFPGPK | Oxidation (M); Oxidation (P) |
| 589.2874 | 1176.56 | 1176.56 | 49.75 | GQAGVMGFPGPK | Oxidation (K); Oxidation (P) |
| 589.7787 | 1177.543 | 1177.544 | 66.56 | GQAGVMGFPGPK | Deamidated (NQ); Oxidation (M); Oxidation (P) |
| 589.7792 | 1177.544 | 1177.544 | 53.43 | GQAGVMGFPGPK | Deamidated (NQ); Oxidation (M); Oxidation (P) |
| 596.8196 | 1191.625 | 1191.625 | 41.87 | GVPGPPGAVGPAGK | 2 Oxidation (P) |
| 597.2845 | 1192.554 | 1192.555 | 48.45 | GQAGVMGFPGPK | Oxidation (K); Oxidation (M); Oxidation (P) |
| 621.8018 | 1241.589 | 1241.589 | 65.28 | GLTGSPGSPGPDGK | Oxidation (P) |
| 629.7992 | 1257.584 | 1257.584 | 44.7 | GLTGSPGSPGPDGK | 2 Oxidation (P) |
| 629.7992 | 1257.584 | 1257.584 | 49.93 | GLTGSPGSPGPDGK | 2 Oxidation (P) |
| 629.7994 | 1257.584 | 1257.584 | 63.11 | GLTGSPGSPGPDGK | 2 Oxidation (P) |
| 629.7994 | 1257.584 | 1257.584 | 50.14 | GLTGSPGSPGPDGK | 2 Oxidation (P) |
| 629.7996 | 1257.585 | 1257.584 | 53.64 | GLTGSPGSPGPDGK | 2 Oxidation (P) |
| 641.313 | 1280.611 | 1280.611 | 43.23 | GEAGPSGPAGPTGAR | |
| 656.8303 | 1311.646 | 1311.646 | 42.62 | GFPGLPGPSGEPGK | Oxidation (P) |
| 656.8307 | 1311.647 | 1311.646 | 63.35 | GFPGLPGPSGEPGK | Oxidation (P) |
| 664.8275 | 1327.64 | 1327.641 | 50.53 | GFPGLPGPSGEPGK | 2 Oxidation (P) |
| 664.8275 | 1327.64 | 1327.641 | 46.53 | GFPGLPGPSGEPGK | 2 Oxidation (P) |
| 664.8276 | 1327.641 | 1327.641 | 61.01 | GFPGLPGPSGEPGK | 2 Oxidation (P) |
| 664.8276 | 1327.641 | 1327.641 | 72.11 | GFPGLPGPSGEPGK | 2 Oxidation (P) |
| 664.8276 | 1327.641 | 1327.641 | 69.32 | GFPGLPGPSGEPGK | 2 Oxidation (P) |
| 664.8279 | 1327.641 | 1327.641 | 43.25 | GFPGLPGPSGEPGK | 2 Oxidation (P) |
| 666.8306 | 1331.647 | 1331.647 | 56.87 | GPSGPQGPSGPPGPK | Oxidation (P) |
| 666.8306 | 1331.647 | 1331.647 | 70.47 | GPSGPQGPSGPPGPK | Oxidation (P) |
| 666.8309 | 1331.647 | 1331.647 | 86.87 | GPSGPQGPSGPPGPK | Oxidation (P) |
| 666.8309 | 1331.647 | 1331.647 | 79.77 | GPSGPQGPSGPPGPK | Oxidation (P) |
| 666.8309 | 1331.647 | 1331.647 | 55.95 | GPSGPQGPSGPPGPK | Oxidation (P) |
| 666.8311 | 1331.648 | 1331.647 | 64.12 | GPSGPQGPSGPPGPK | Oxidation (P) |
| 666.8311 | 1331.648 | 1331.647 | 62.38 | GPSGPQGPSGPPGPK | Oxidation (P) |
| 666.8312 | 1331.648 | 1331.647 | 59.64 | GPSGPQGPSGPPGPK | Oxidation (P) |
| 666.8312 | 1331.648 | 1331.647 | 43.15 | GPSGPQGPSGPPGPK | Oxidation (P) |
| 666.8313 | 1331.648 | 1331.647 | 65.01 | GPSGPQGPSGPPGPK | Oxidation (P) |
| 666.8314 | 1331.648 | 1331.647 | 63.43 | GPSGPQGPSGPPGPK | Oxidation (P) |
| 667.3231 | 1332.632 | 1332.631 | 48.3 | GPSGPQGPSGPPGPK | Deamidated (NQ); Oxidation (P) |
| 667.3235 | 1332.632 | 1332.631 | 87.85 | GPSGPQGPSGPPGPK | Deamidated (NQ); Oxidation (P) |
| 672.8248 | 1343.635 | 1343.636 | 43.09 | GFPGLPGPSGEPGK | 3 Oxidation (P) |
| 672.8249 | 1343.635 | 1343.636 | 52.54 | GFPGLPGPSGEPGK | 3 Oxidation (P) |
| 672.8251 | 1343.636 | 1343.636 | 64.48 | GFPGLPGPSGEPGK | 3 Oxidation (P) |
| 672.8253 | 1343.636 | 1343.636 | 57.32 | GFPGLPGPSGEPGK | 3 Oxidation (P) |
| 672.8254 | 1343.636 | 1343.636 | 40.91 | GFPGLPGPSGEPGK | 3 Oxidation (P) |
| 674.8279 | 1347.641 | 1347.642 | 52.82 | GPSGPQGPSGPPGPK | 2 Oxidation (P) |
| 674.8285 | 1347.642 | 1347.642 | 58.59 | GPSGPQGPSGPPGPK | 2 Oxidation (P) |
| 718.3437 | 1434.673 | 1434.674 | 74.48 | GEPGPAGLPGPPGER | 3 Oxidation (P) |
| 718.3445 | 1434.674 | 1434.674 | 53.05 | GEPGPAGLPGPPGER | 3 Oxidation (P) |
| 718.3447 | 1434.675 | 1434.674 | 57.77 | GEPGPAGLPGPPGER | 3 Oxidation (P) |
| 728.4122 | 1454.81 | 1454.809 | 55.61 | ALLLQGSNEIEIR | |
| 728.9041 | 1455.794 | 1455.793 | 74.86 | ALLLQGSNEIEIR | Deamidated (NQ) |
| 729.3965 | 1456.778 | 1456.777 | 41.24 | ALLLQGSNEIEIR | 2 Deamidated (NQ) |
| 730.3493 | 1458.684 | 1458.685 | 89.83 | GSAGPPGATGFPGAAGR | 2 Oxidation (P) |
| 487.2354 | 1458.684 | 1458.685 | 47.12 | GSAGPPGATGFPGAAGR | 2 Oxidation (P) |
| 730.3495 | 1458.684 | 1458.685 | 84.51 | GSAGPPGATGFPGAAGR | 2 Oxidation (P) |
| 730.3497 | 1458.685 | 1458.685 | 45.39 | GSAGPPGATGFPGAAGR | 2 Oxidation (P) |
| 730.3497 | 1458.685 | 1458.685 | 41.54 | GSAGPPGATGFPGAAGR | 2 Oxidation (P) |
| 730.3499 | 1458.685 | 1458.685 | 82.98 | GSAGPPGATGFPGAAGR | 2 Oxidation (P) |
| 730.3499 | 1458.685 | 1458.685 | 78.61 | GSAGPPGATGFPGAAGR | 2 Oxidation (P) |
| 730.3499 | 1458.685 | 1458.685 | 65.28 | GSAGPPGATGFPGAAGR | 2 Oxidation (P) |
| 730.35 | 1458.685 | 1458.685 | 76.27 | GSAGPPGATGFPGAAGR | 2 Oxidation (P) |
| 730.35 | 1458.685 | 1458.685 | 56.12 | GSAGPPGATGFPGAAGR | 2 Oxidation (P) |
| 730.35 | 1458.685 | 1458.685 | 77.35 | GSAGPPGATGFPGAAGR | 2 Oxidation (P) |
| 730.35 | 1458.685 | 1458.685 | 41.8 | GSAGPPGATGFPGAAGR | 2 Oxidation (P) |
| 730.35 | 1458.685 | 1458.685 | 61.31 | GSAGPPGATGFPGAAGR | 2 Oxidation (P) |
| 730.35 | 1458.685 | 1458.685 | 61.03 | GSAGPPGATGFPGAAGR | 2 Oxidation (P) |
| 730.35 | 1458.685 | 1458.685 | 53.73 | GSAGPPGATGFPGAAGR | 2 Oxidation (P) |
| 730.3501 | 1458.686 | 1458.685 | 41.49 | GSAGPPGATGFPGAAGR | 2 Oxidation (P) |
| 730.3502 | 1458.686 | 1458.685 | 85.31 | GSAGPPGATGFPGAAGR | 2 Oxidation (P) |
| 730.3503 | 1458.686 | 1458.685 | 78.86 | GSAGPPGATGFPGAAGR | 2 Oxidation (P) |
| 730.3503 | 1458.686 | 1458.685 | 40.05 | GSAGPPGATGFPGAAGR | 2 Oxidation (P) |
| 730.3504 | 1458.686 | 1458.685 | 57.26 | GSAGPPGATGFPGAAGR | 2 Oxidation (P) |
| 730.3505 | 1458.686 | 1458.685 | 75.33 | GSAGPPGATGFPGAAGR | 2 Oxidation (P) |
| 730.3506 | 1458.687 | 1458.685 | 89.05 | GSAGPPGATGFPGAAGR | 2 Oxidation (P) |
| 730.3506 | 1458.687 | 1458.685 | 65.61 | GSAGPPGATGFPGAAGR | 2 Oxidation (P) |
| 730.3511 | 1458.688 | 1458.685 | 59.5 | GSAGPPGATGFPGAAGR | 2 Oxidation (P) |
| 730.3512 | 1458.688 | 1458.685 | 43.28 | GSAGPPGATGFPGAAGR | 2 Oxidation (P) |
| 730.352 | 1458.689 | 1458.685 | 65.45 | GSAGPPGATGFPGAAGR | 2 Oxidation (P) |
| 748.8801 | 1495.746 | 1495.745 | 46.94 | STGISVPGPMGPSGPR | |
| 773.8965 | 1545.778 | 1545.779 | 57.38 | DGLNGLPGPIGPPGPR | Deamidated (NQ); 2 Oxidation (P) |
| 780.9077 | 1559.801 | 1559.806 | 50.51 | GETGPAGPAGPIGPVGAR | |
| 780.9092 | 1559.804 | 1559.806 | 79 | GETGPAGPAGPIGPVGAR | |
| 520.9419 | 1559.804 | 1559.806 | 46.93 | GETGPAGPAGPIGPVGAR | |
| 521.2704 | 1560.789 | 1560.79 | 51.16 | DGLNGLPGPIGPPGPR | 3 Oxidation (P) |
| 781.8922 | 1561.77 | 1561.774 | 43.43 | DGLNGLPGPIGPPGPR | Deamidated (NQ); 3 Oxidation (P) |
| 781.8936 | 1561.773 | 1561.774 | 64.8 | DGLNGLPGPIGPPGPR | Deamidated (NQ); 3 Oxidation (P) |
| 781.8937 | 1561.773 | 1561.774 | 57.63 | DGLNGLPGPIGPPGPR | Deamidated (NQ); 3 Oxidation (P) |
| 521.5983 | 1561.773 | 1561.774 | 45.12 | DGLNGLPGPIGPPGPR | Deamidated (NQ); 3 Oxidation (P) |
| 781.8941 | 1561.774 | 1561.774 | 42.47 | DGLNGLPGPIGPPGPR | Deamidated (NQ); 3 Oxidation (P) |
| 781.8944 | 1561.774 | 1561.774 | 53.63 | DGLNGLPGPIGPPGPR | Deamidated (NQ); 3 Oxidation (P) |
| 781.8965 | 1561.778 | 1561.774 | 41 | DGLNGLPGPIGPPGPR | Deamidated (NQ); 3 Oxidation (P) |
| 788.9082 | 1575.802 | 1575.801 | 59.04 | GETGPAGPAGPIGPVGAR | Oxidation (P) |
| 793.39 | 1584.765 | 1584.765 | 66.73 | GANGAPGIAGAPGFPGAR | 3 Oxidation (P) |
| 793.3901 | 1584.766 | 1584.765 | 42.44 | GANGAPGIAGAPGFPGAR | 3 Oxidation (P) |
| 793.8805 | 1585.746 | 1585.749 | 45.65 | GANGAPGIAGAPGFPGAR | Deamidated (NQ); 3 Oxidation (P) |
| 529.5895 | 1585.747 | 1585.749 | 46.1 | GANGAPGIAGAPGFPGAR | Deamidated (NQ); 3 Oxidation (P) |
| 793.8807 | 1585.747 | 1585.749 | 63.39 | GANGAPGIAGAPGFPGAR | Deamidated (NQ); 3 Oxidation (P) |
| 793.8813 | 1585.748 | 1585.749 | 40.33 | GANGAPGIAGAPGFPGAR | Deamidated (NQ); 3 Oxidation (P) |
| 793.8816 | 1585.749 | 1585.749 | 68.62 | GANGAPGIAGAPGFPGAR | Deamidated (NQ); 3 Oxidation (P) |
| 793.8823 | 1585.75 | 1585.749 | 43.76 | GANGAPGIAGAPGFPGAR | Deamidated (NQ); 3 Oxidation (P) |
| 808.3952 | 1614.776 | 1614.775 | 50.1 | GGPGSRGFPGADGVAGPK | 2 Oxidation (P) |
| 809.397 | 1616.779 | 1616.78 | 85.89 | GFSGLDGAKGDAGPAGPK | Oxidation (K) |
| 818.9238 | 1635.833 | 1635.833 | 54.61 | GAAGLPGPKGDRGDAGPK | Oxidation (P) |
| 828.4035 | 1654.792 | 1654.791 | 58.78 | GSPGEAGRPGEAGLPGAK | 3 Oxidation (P) |
| 828.4108 | 1654.807 | 1654.806 | 61.27 | GFPGADGVAGPKGPAGER | Oxidation (P) |
| 845.8929 | 1689.771 | 1689.771 | 50.01 | DGEAGAQGPPGPAGPAGER | |
| 569.5959 | 1705.766 | 1705.766 | 49.17 | DGEAGAQGPPGPAGPAGER | Oxidation (P) |
| 853.8905 | 1705.766 | 1705.766 | 98.97 | DGEAGAQGPPGPAGPAGER | Oxidation (P) |
| 853.8905 | 1705.766 | 1705.766 | 101.77 | DGEAGAQGPPGPAGPAGER | Oxidation (P) |
| 854.3823 | 1706.75 | 1706.75 | 107.79 | DGEAGAQGPPGPAGPAGER | Deamidated (NQ); Oxidation (P) |
| 871.8737 | 1741.733 | 1741.733 | 106.99 | GEPGSPGENGAPGQMGPR | 3 Oxidation (P) |
| 872.3663 | 1742.718 | 1742.717 | 78.06 | GEPGSPGENGAPGQMGPR | Deamidated (NQ); 3 Oxidation (P) |
| 883.9171 | 1765.82 | 1765.823 | 45.92 | PGEQGVPGDLGAPGPSGAR | 3 Oxidation (P) |
| 900.9387 | 1799.863 | 1799.863 | 104.28 | GPPGPMGPPGLAGPPGESGR | Oxidation (P) |
| 906.9482 | 1811.882 | 1811.88 | 49.72 | VGPPGPSGNAGPPGPPGPAGK | Oxidation (K); 2 Oxidation (P) |
| 907.4395 | 1812.864 | 1812.864 | 42.2 | VGPPGPSGNAGPPGPPGPAGK | Deamidated (NQ); Oxidation (K); 2 Oxidation (P) |
| 606.2929 | 1815.857 | 1815.857 | 55.85 | GPPGPMGPPGLAGPPGESGR | 2 Oxidation (P) |
| 908.9362 | 1815.858 | 1815.857 | 58.61 | GPPGPMGPPGLAGPPGESGR | 2 Oxidation (P) |
| 916.9341 | 1831.854 | 1831.852 | 69.84 | GPPGPMGPPGLAGPPGESGR | Oxidation (M); 2 Oxidation (P) |
| 916.9341 | 1831.854 | 1831.852 | 71.61 | GPPGPMGPPGLAGPPGESGR | Oxidation (M); 2 Oxidation (P) |
| 916.9345 | 1831.854 | 1831.852 | 41.61 | GPPGPMGPPGLAGPPGESGR | Oxidation (M); 2 Oxidation (P) |
| 916.9363 | 1831.858 | 1831.852 | 57.61 | GPPGPMGPPGLAGPPGESGR | Oxidation (M); 2 Oxidation (P) |
| 932.4399 | 1862.865 | 1862.865 | 79.49 | GEPGPTGIQGPPGPAGEEGK | 2 Oxidation (P) |
| 932.933 | 1863.851 | 1863.849 | 45.45 | GEPGPTGIQGPPGPAGEEGK | Deamidated (NQ); 2 Oxidation (P) |
| 659.3363 | 1974.987 | 1974.987 | 43.07 | SGDRGETGPAGPAGPIGPVGAR | |
| 659.3363 | 1974.987 | 1974.987 | 53.27 | SGDRGETGPAGPAGPIGPVGAR | |
| 659.3364 | 1974.987 | 1974.987 | 47.26 | SGDRGETGPAGPAGPIGPVGAR | |
| 988.5011 | 1974.988 | 1974.987 | 61.98 | SGDRGETGPAGPAGPIGPVGAR | |
| 988.5012 | 1974.988 | 1974.987 | 65.68 | SGDRGETGPAGPAGPIGPVGAR | |
| 988.5016 | 1974.989 | 1974.987 | 47 | SGDRGETGPAGPAGPIGPVGAR | |
| 988.502 | 1974.989 | 1974.987 | 41.08 | SGDRGETGPAGPAGPIGPVGAR | |
| 673.9962 | 2018.967 | 2018.966 | 52.4 | GEPGPTGIQGPPGPAGEEGKR | 2 Oxidation (P) |
| 1010.491 | 2018.967 | 2018.966 | 74.6 | GEPGPTGIQGPPGPAGEEGKR | 2 Oxidation (P) |
| 1010.492 | 2018.969 | 2018.966 | 43.58 | GEPGPTGIQGPPGPAGEEGKR | 2 Oxidation (P) |
| 1029.012 | 2056.009 | 2056.009 | 82.09 | GAPGADGPAGAPGTPGPQGIAGQR | |
| 1037.009 | 2072.003 | 2072.004 | 80.6 | GAPGADGPAGAPGTPGPQGIAGQR | Oxidation (P) |
| 1037.009 | 2072.003 | 2072.004 | 42.22 | GAPGADGPAGAPGTPGPQGIAGQR | Oxidation (P) |
| 691.6751 | 2072.004 | 2072.004 | 44.5 | GAPGADGPAGAPGTPGPQGIAGQR | Oxidation (P) |
| 1037.01 | 2072.005 | 2072.004 | 44.56 | GAPGADGPAGAPGTPGPQGIAGQR | Oxidation (P) |
| 1037.01 | 2072.005 | 2072.004 | 72.1 | GAPGADGPAGAPGTPGPQGIAGQR | Oxidation (P) |
| 1037.502 | 2072.989 | 2072.988 | 71.3 | GAPGADGPAGAPGTPGPQGIAGQR | Deamidated (NQ); Oxidation (P) |
| 1045.007 | 2087.999 | 2087.999 | 66.62 | GAPGADGPAGAPGTPGPQGIAGQR | 2 Oxidation (P) |
| 1045.007 | 2087.999 | 2087.999 | 80.41 | GAPGADGPAGAPGTPGPQGIAGQR | 2 Oxidation (P) |
| 1045.498 | 2088.981 | 2088.983 | 86.47 | GAPGADGPAGAPGTPGPQGIAGQR | Deamidated (NQ); 2 Oxidation (P) |
| 1045.499 | 2088.983 | 2088.983 | 55.61 | GAPGADGPAGAPGTPGPQGIAGQR | Deamidated (NQ); 2 Oxidation (P) |
| 1045.992 | 2089.969 | 2089.967 | 61.12 | GAPGADGPAGAPGTPGPQGIAGQR | 2 Deamidated (NQ); 2 Oxidation (P) |
| 1053.003 | 2103.991 | 2103.993 | 45.5 | GAPGADGPAGAPGTPGPQGIAGQR | 3 Oxidation (P) |
| 1053.005 | 2103.995 | 2103.993 | 86.29 | GAPGADGPAGAPGTPGPQGIAGQR | 3 Oxidation (P) |
| 1053.495 | 2104.975 | 2104.977 | 59.23 | GAPGADGPAGAPGTPGPQGIAGQR | Deamidated (NQ); 3 Oxidation (P) |
| 1053.495 | 2104.975 | 2104.977 | 45.53 | GAPGADGPAGAPGTPGPQGIAGQR | Deamidated (NQ); 3 Oxidation (P) |
| 1067.495 | 2132.975 | 2132.976 | 40.88 | GEPGPPGPAGFAGPPGADGQPGAK | 3 Oxidation (P) |
| 1067.988 | 2133.961 | 2133.96 | 60.65 | GEPGPPGPAGFAGPPGADGQPGAK | Deamidated (NQ); 3 Oxidation (P) |
| 1069.471 | 2136.927 | 2136.921 | 54.37 | FTYSVTYDGCTSHTGAWGK | |
| 1085.032 | 2168.049 | 2168.05 | 66.74 | GETGPAGPPGAPGAPGAPGPVGPAGK | 3 Oxidation (P) |
| 723.6906 | 2168.05 | 2168.05 | 48.42 | GETGPAGPPGAPGAPGAPGPVGPAGK | 3 Oxidation (P) |
| 1099.991 | 2197.967 | 2197.966 | 75.18 | GDAGAPGAPGSQGAPGLQGMPGER | 4 Oxidation (P) |
| 1100.483 | 2198.951 | 2198.95 | 77.68 | GDAGAPGAPGSQGAPGLQGMPGER | Deamidated (NQ); 4 Oxidation (P) |
| 1100.483 | 2198.951 | 2198.95 | 51.02 | GDAGAPGAPGSQGAPGLQGMPGER | Deamidated (NQ); 4 Oxidation (P) |
| 1100.537 | 2199.059 | 2199.056 | 43.25 | GETGPAGRPGEVGPPGPPGPAGEK | 2 Oxidation (P) |
| 739.3578 | 2215.052 | 2215.051 | 44.7 | GETGPAGRPGEVGPPGPPGPAGEK | Oxidation (K); 2 Oxidation (P) |
| 1108.97 | 2215.925 | 2215.929 | 51.66 | GDAGAPGAPGSQGAPGLQGMPGER | 2 Deamidated (NQ); Oxidation (M); 4 Oxidation (P) |
| 1138.077 | 2274.139 | 2274.139 | 87.98 | GDAGPPGPAGPAGPPGPIGNVGAPGPK | Oxidation (P) |
| 759.0541 | 2274.141 | 2274.139 | 45.21 | GDAGPPGPAGPAGPPGPIGNVGAPGPK | Oxidation (P) |
| 1138.079 | 2274.143 | 2274.139 | 80.15 | GDAGPPGPAGPAGPPGPIGNVGAPGPK | Oxidation (K) |
| 1146.074 | 2290.133 | 2290.134 | 43.39 | GDAGPPGPAGPAGPPGPIGNVGAPGPK | Oxidation (K); Oxidation (P) |
| 764.3856 | 2290.135 | 2290.134 | 55.31 | GDAGPPGPAGPAGPPGPIGNVGAPGPK | Oxidation (K); Oxidation (P) |
| 1146.075 | 2290.135 | 2290.134 | 77.93 | GDAGPPGPAGPAGPPGPIGNVGAPGPK | Oxidation (K); Oxidation (P) |
| 764.7135 | 2291.119 | 2291.118 | 44.97 | GDAGPPGPAGPAGPPGPIGNVGAPGPK | Deamidated (NQ); Oxidation (K); Oxidation (P) |
| 1146.569 | 2291.123 | 2291.118 | 87.59 | GDAGPPGPAGPAGPPGPIGNVGAPGPK | Deamidated (NQ); Oxidation (K); Oxidation (P) |
| 1154.072 | 2306.129 | 2306.129 | 41.94 | GDAGPPGPAGPAGPPGPIGNVGAPGPK | Oxidation (K); 2 Oxidation (P) |
| 1158.528 | 2315.041 | 2315.042 | 63.32 | GEPGPPGPAGAAGPAGNPGADGQPGAK | 4 Oxidation (P) |
| 772.6884 | 2315.043 | 2315.042 | 50.44 | GEPGPPGPAGAAGPAGNPGADGQPGAK | Oxidation (K); 3 Oxidation (P) |
| 772.6885 | 2315.044 | 2315.042 | 58.53 | GEPGPPGPAGAAGPAGNPGADGQPGAK | 4 Oxidation (P) |
| 1159.021 | 2316.027 | 2316.026 | 62.76 | GEPGPPGPAGAAGPAGNPGADGQPGAK | Deamidated (NQ); 4 Oxidation (P) |
| 778.0201 | 2331.039 | 2331.036 | 51.2 | GEPGPPGPAGAAGPAGNPGADGQPGAK | Oxidation (K); 4 Oxidation (P) |
| 827.7415 | 2480.203 | 2480.205 | 44.49 | GDRGETGPAGPPGAPGAPGAPGPVGPAGK | 2 Oxidation (P) |
| 829.7421 | 2486.205 | 2486.204 | 47.41 | GPPGSAGSPGKDGLNGLPGPIGPPGPR | Deamidated (NQ); 5 Oxidation (P) |
| 831.7076 | 2492.101 | 2492.099 | 47.04 | GDAGPAGPKGEPGSPGENGAPGQMGPR | 3 Oxidation (P) |
| 833.0742 | 2496.201 | 2496.199 | 46.92 | GDRGETGPAGPPGAPGAPGAPGPVGPAGK | 3 Oxidation (P) |
| 837.3673 | 2509.08 | 2509.078 | 51.71 | GDAGPAGPKGEPGSPGENGAPGQMGPR | Deamidated (NQ); Oxidation (M); 3 Oxidation (P) |
| 844.7424 | 2531.205 | 2531.204 | 45.37 | GNDGATGAAGPPGPTGPAGPPGFPGAVGAK | 2 Oxidation (P) |
| 1266.61 | 2531.205 | 2531.204 | 47.55 | GNDGATGAAGPPGPTGPAGPPGFPGAVGAK | 2 Oxidation (P) |
| 1267.102 | 2532.189 | 2532.188 | 43.89 | GNDGATGAAGPPGPTGPAGPPGFPGAVGAK | Deamidated (NQ); 2 Oxidation (P) |
| 850.0737 | 2547.199 | 2547.199 | 53.68 | GNDGATGAAGPPGPTGPAGPPGFPGAVGAK | 3 Oxidation (P) |
| 1274.607 | 2547.199 | 2547.199 | 75.25 | GNDGATGAAGPPGPTGPAGPPGFPGAVGAK | 3 Oxidation (P) |
| 850.0749 | 2547.203 | 2547.199 | 54.65 | GNDGATGAAGPPGPTGPAGPPGFPGAVGAK | 3 Oxidation (P) |
| 850.402 | 2548.184 | 2548.183 | 42.93 | GNDGATGAAGPPGPTGPAGPPGFPGAVGAK | Deamidated (NQ); 3 Oxidation (P) |
| 1275.1 | 2548.185 | 2548.183 | 81.04 | GNDGATGAAGPPGPTGPAGPPGFPGAVGAK | Deamidated (NQ); 3 Oxidation (P) |
| 850.413 | 2548.217 | 2548.215 | 50.99 | GDTGAKGEPGPTGIQGPPGPAGEEGKR | 2 Oxidation (P) |
| 1282.606 | 2563.197 | 2563.194 | 57.51 | GNDGATGAAGPPGPTGPAGPPGFPGAVGAK | Oxidation (K); 3 Oxidation (P) |
| 1283.096 | 2564.177 | 2564.178 | 56.45 | GNDGATGAAGPPGPTGPAGPPGFPGAVGAK | Deamidated (NQ); Oxidation (K); 3 Oxidation (P) |
| 1337.138 | 2672.261 | 2672.258 | 85.83 | GFSGLQGPPGPPGSPGEQGPSGASGPAGPR | Oxidation (P) |
| 892.088 | 2673.242 | 2673.242 | 44.23 | GFSGLQGPPGPPGSPGEQGPSGASGPAGPR | Deamidated (NQ); Oxidation (P) |
| 1337.632 | 2673.249 | 2673.242 | 111.08 | GFSGLQGPPGPPGSPGEQGPSGASGPAGPR | Deamidated (NQ); Oxidation (P) |
| 897.0914 | 2688.252 | 2688.253 | 60.62 | GFSGLQGPPGPPGSPGEQGPSGASGPAGPR | 2 Oxidation (P) |
| 1345.135 | 2688.255 | 2688.253 | 94.51 | GFSGLQGPPGPPGSPGEQGPSGASGPAGPR | 2 Oxidation (P) |
| 1345.136 | 2688.257 | 2688.253 | 93.47 | GFSGLQGPPGPPGSPGEQGPSGASGPAGPR | 2 Oxidation (P) |
| 897.4201 | 2689.239 | 2689.237 | 44.99 | GFSGLQGPPGPPGSPGEQGPSGASGPAGPR | Deamidated (NQ); 2 Oxidation (P) |
| 902.4229 | 2704.247 | 2704.248 | 56.13 | GFSGLQGPPGPPGSPGEQGPSGASGPAGPR | 3 Oxidation (P) |
| 1353.131 | 2704.247 | 2704.248 | 70.08 | GFSGLQGPPGPPGSPGEQGPSGASGPAGPR | 3 Oxidation (P) |
| 1353.131 | 2704.247 | 2704.248 | 47.54 | GFSGLQGPPGPPGSPGEQGPSGASGPAGPR | 3 Oxidation (P) |
| 1353.131 | 2704.247 | 2704.248 | 44.66 | GFSGLQGPPGPPGSPGEQGPSGASGPAGPR | 3 Oxidation (P) |
| 902.4233 | 2704.248 | 2704.248 | 51.06 | GFSGLQGPPGPPGSPGEQGPSGASGPAGPR | 3 Oxidation (P) |
| 1353.132 | 2704.249 | 2704.248 | 72.81 | GFSGLQGPPGPPGSPGEQGPSGASGPAGPR | 3 Oxidation (P) |
| 1353.132 | 2704.249 | 2704.248 | 74.78 | GFSGLQGPPGPPGSPGEQGPSGASGPAGPR | 3 Oxidation (P) |
| 902.7514 | 2705.232 | 2705.232 | 69.35 | GFSGLQGPPGPPGSPGEQGPSGASGPAGPR | Deamidated (NQ); 3 Oxidation (P) |
| 1353.624 | 2705.233 | 2705.232 | 83.37 | GFSGLQGPPGPPGSPGEQGPSGASGPAGPR | Deamidated (NQ); 3 Oxidation (P) |
| 1353.624 | 2705.233 | 2705.232 | 42.1 | GFSGLQGPPGPPGSPGEQGPSGASGPAGPR | Deamidated (NQ); 3 Oxidation (P) |
| 907.0839 | 2718.23 | 2718.227 | 42.89 | GAPGDRGEPGPPGPAGFAGPPGADGQPGAK | 5 Oxidation (P) |
| 951.8094 | 2852.406 | 2852.405 | 44.95 | GLTGPIGPPGPAGAPGDKGEAGPSGPAGPTGAR | 2 Oxidation (P) |
| 1427.211 | 2852.407 | 2852.405 | 54.4 | GLTGPIGPPGPAGAPGDKGEAGPSGPAGPTGAR | 2 Oxidation (P) |
| 1025.171 | 3072.491 | 3072.49 | 41.72 | GEPGDAGAKGDAGPPGPAGPAGPPGPIGNVGAPGPK | Oxidation (K); Oxidation (P) |
| 1030.503 | 3088.487 | 3088.485 | 44.48 | GEPGDAGAKGDAGPPGPAGPAGPPGPIGNVGAPGPK | Oxidation (K); 2 Oxidation (P) |
| 1035.835 | 3104.483 | 3104.48 | 47.34 | GEPGDAGAKGDAGPPGPAGPAGPPGPIGNVGAPGPK | Oxidation (K); 3 Oxidation (P) |
| 1036.163 | 3105.467 | 3105.464 | 41.34 | GEPGDAGAKGDAGPPGPAGPAGPPGPIGNVGAPGPK | Deamidated (NQ); Oxidation (K); 3 Oxidation (P) |
| 1037.17 | 3108.488 | 3108.486 | 46.26 | DGSPGAKGDRGETGPAGPPGAPGAPGAPGPVGPAGK | Oxidation (K); 2 Oxidation (P) |
| 1041.167 | 3120.479 | 3120.475 | 45.11 | GEPGDAGAKGDAGPPGPAGPAGPPGPIGNVGAPGPK | 5 Oxidation (P) |
| 1056.498 | 3166.472 | 3166.466 | 53.56 | GSEGPQGVRGEPGPPGPAGAAGPAGNPGADGQPGAK | Oxidation (K); 2 Oxidation (P) |
| 1061.828 | 3182.462 | 3182.461 | 40.68 | GSEGPQGVRGEPGPPGPAGAAGPAGNPGADGQPGAK | 4 Oxidation (P) |
| 1070.805 | 3209.393 | 3209.392 | 40.23 | GANGAPGNDGAKGDAGAPGAPGSQGAPGLQGMPGER | 2 Deamidated (NQ); 4 Oxidation (P) |
| 1075.808 | 3224.402 | 3224.403 | 41.01 | GANGAPGNDGAKGDAGAPGAPGSQGAPGLQGMPGER | Deamidated (NQ); 5 Oxidation (P) |
| 1086.803 | 3257.387 | 3257.376 | 40.1 | GANGAPGNDGAKGDAGAPGAPGSQGAPGLQGMPGER | 2 Deamidated (NQ); Oxidation (K); Oxidation (M); 5 Oxidation (P) |

| Collagen alpha-2(I) chain OS=Bos taurus GN=COL1A2 PE=1 SV=2 | 5421 |
| --- | --- |

| 434.7355 | 867.4564 | 867.4563 | 52.52 | GPSGPQGIR | |
| --- | --- | --- | --- | --- | --- |
| 446.7535 | 891.4924 | 891.4926 | 43.53 | PGPIGPAGAR | |
| 451.7293 | 901.444 | 901.444 | 47.81 | AGVMGPAGSR | |
| 459.7262 | 917.4378 | 917.4389 | 64.62 | AGVMGPAGSR | Oxidation (M) |
| 459.7268 | 917.439 | 917.4389 | 49.28 | AGVMGPAGSR | Oxidation (M) |
| 459.727 | 917.4394 | 917.4389 | 43.64 | AGVMGPAGSR | Oxidation (P) |
| 591.8089 | 1181.603 | 1181.604 | 48.48 | EGPVGLPGIDGR | Oxidation (P) |
| 591.8093 | 1181.604 | 1181.604 | 44.55 | EGPVGLPGIDGR | Oxidation (P) |
| 592.2886 | 1182.563 | 1182.564 | 50.26 | GGGPGPMGLMGPR | |
| 398.2312 | 1191.672 | 1191.672 | 52.43 | IGQPGAVGPAGIR | |
| 596.8433 | 1191.672 | 1191.672 | 66.77 | IGQPGAVGPAGIR | |
| 596.8433 | 1191.672 | 1191.672 | 49.92 | IGQPGAVGPAGIR | |
| 596.8435 | 1191.672 | 1191.672 | 67.49 | IGQPGAVGPAGIR | |
| 597.3352 | 1192.656 | 1192.656 | 60.99 | IGQPGAVGPAGIR | Deamidated (NQ) |
| 597.3355 | 1192.656 | 1192.656 | 57.34 | IGQPGAVGPAGIR | Deamidated (NQ) |
| 600.2864 | 1198.558 | 1198.559 | 48.9 | GGGPGPMGLMGPR | Oxidation (M) |
| 601.2957 | 1200.577 | 1200.578 | 63.86 | GEPGNIGFPGPK | 2 Oxidation (P) |
| 601.2962 | 1200.578 | 1200.578 | 45.19 | GEPGNIGFPGPK | 2 Oxidation (P) |
| 601.2964 | 1200.578 | 1200.578 | 54.31 | GEPGNIGFPGPK | 2 Oxidation (P) |
| 601.7883 | 1201.562 | 1201.562 | 47.77 | GEPGNIGFPGPK | Deamidated (NQ); 2 Oxidation (P) |
| 603.8112 | 1205.608 | 1205.608 | 42.31 | GFPGTPGLPGFK | Oxidation (K); Oxidation (P) |
| 604.8403 | 1207.666 | 1207.667 | 40.05 | IGQPGAVGPAGIR | Oxidation (P) |
| 604.8405 | 1207.666 | 1207.667 | 43.04 | IGQPGAVGPAGIR | Oxidation (P) |
| 604.8405 | 1207.666 | 1207.667 | 62.58 | IGQPGAVGPAGIR | Oxidation (P) |
| 604.8406 | 1207.667 | 1207.667 | 49.25 | IGQPGAVGPAGIR | Oxidation (P) |
| 604.8408 | 1207.667 | 1207.667 | 40.37 | IGQPGAVGPAGIR | Oxidation (P) |
| 604.8409 | 1207.667 | 1207.667 | 61.7 | IGQPGAVGPAGIR | Oxidation (P) |
| 604.8409 | 1207.667 | 1207.667 | 47.35 | IGQPGAVGPAGIR | Oxidation (P) |
| 604.8409 | 1207.667 | 1207.667 | 71.08 | IGQPGAVGPAGIR | Oxidation (P) |
| 604.8409 | 1207.667 | 1207.667 | 43.07 | IGQPGAVGPAGIR | Oxidation (P) |
| 604.8409 | 1207.667 | 1207.667 | 53.41 | IGQPGAVGPAGIR | Oxidation (P) |
| 604.841 | 1207.667 | 1207.667 | 42.13 | IGQPGAVGPAGIR | Oxidation (P) |
| 403.5631 | 1207.668 | 1207.667 | 45.79 | IGQPGAVGPAGIR | Oxidation (P) |
| 604.8411 | 1207.668 | 1207.667 | 43.3 | IGQPGAVGPAGIR | Oxidation (P) |
| 403.5633 | 1207.668 | 1207.667 | 55.05 | IGQPGAVGPAGIR | Oxidation (P) |
| 605.3329 | 1208.651 | 1208.651 | 55.91 | IGQPGAVGPAGIR | Deamidated (NQ); Oxidation (P) |
| 611.8084 | 1221.602 | 1221.603 | 40.93 | GFPGTPGLPGFK | 3 Oxidation (P) |
| 611.8086 | 1221.603 | 1221.603 | 40.05 | GFPGTPGLPGFK | Oxidation (K); 2 Oxidation (P) |
| 611.8087 | 1221.603 | 1221.603 | 49.76 | GFPGTPGLPGFK | 3 Oxidation (P) |
| 611.8091 | 1221.604 | 1221.603 | 41.08 | GFPGTPGLPGFK | 3 Oxidation (P) |
| 631.3174 | 1260.62 | 1260.621 | 44.66 | GEAGPAGPAGPAGPR | |
| 631.3177 | 1260.621 | 1260.621 | 72.76 | GEAGPAGPAGPAGPR | |
| 634.3407 | 1266.667 | 1266.668 | 86.23 | GIPGPVGAAGATGAR | Oxidation (P) |
| 634.3414 | 1266.668 | 1266.668 | 66.26 | GIPGPVGAAGATGAR | Oxidation (P) |
| 634.3417 | 1266.669 | 1266.668 | 80.38 | GIPGPVGAAGATGAR | Oxidation (P) |
| 644.3196 | 1286.625 | 1286.626 | 73.21 | GFPGSPGNIGPAGK | 2 Oxidation (P) |
| 644.8113 | 1287.608 | 1287.61 | 66.14 | GFPGSPGNIGPAGK | Deamidated (NQ); 2 Oxidation (P) |
| 463.2352 | 1386.684 | 1386.685 | 53.63 | GETGLRGDIGSPGR | Oxidation (P) |
| 714.3676 | 1426.721 | 1426.721 | 76.01 | GIPGEFGLPGPAGAR | 2 Oxidation (P) |
| 714.3676 | 1426.721 | 1426.721 | 45.8 | GIPGEFGLPGPAGAR | 2 Oxidation (P) |
| 714.3677 | 1426.721 | 1426.721 | 55.25 | GIPGEFGLPGPAGAR | 2 Oxidation (P) |
| 714.3679 | 1426.721 | 1426.721 | 42.62 | GIPGEFGLPGPAGAR | 2 Oxidation (P) |
| 714.3681 | 1426.722 | 1426.721 | 41.69 | GIPGEFGLPGPAGAR | 2 Oxidation (P) |
| 714.3684 | 1426.722 | 1426.721 | 41.27 | GIPGEFGLPGPAGAR | 2 Oxidation (P) |
| 729.3422 | 1456.67 | 1456.67 | 90.01 | GDGGPPGATGFPGAAGR | Oxidation (P) |
| 729.3422 | 1456.67 | 1456.67 | 77.48 | GDGGPPGATGFPGAAGR | Oxidation (P) |
| 729.3423 | 1456.67 | 1456.67 | 41.8 | GDGGPPGATGFPGAAGR | Oxidation (P) |
| 737.3384 | 1472.662 | 1472.665 | 84.63 | GDGGPPGATGFPGAAGR | 2 Oxidation (P) |
| 737.3384 | 1472.662 | 1472.665 | 51.33 | GDGGPPGATGFPGAAGR | 2 Oxidation (P) |
| 737.3386 | 1472.663 | 1472.665 | 52.37 | GDGGPPGATGFPGAAGR | 2 Oxidation (P) |
| 737.3387 | 1472.663 | 1472.665 | 91.54 | GDGGPPGATGFPGAAGR | 2 Oxidation (P) |
| 737.3392 | 1472.664 | 1472.665 | 55.75 | GDGGPPGATGFPGAAGR | 2 Oxidation (P) |
| 737.3394 | 1472.664 | 1472.665 | 53.54 | GDGGPPGATGFPGAAGR | 2 Oxidation (P) |
| 737.3395 | 1472.664 | 1472.665 | 60.49 | GDGGPPGATGFPGAAGR | 2 Oxidation (P) |
| 737.34 | 1472.665 | 1472.665 | 68.42 | GDGGPPGATGFPGAAGR | 2 Oxidation (P) |
| 737.34 | 1472.665 | 1472.665 | 46.3 | GDGGPPGATGFPGAAGR | 2 Oxidation (P) |
| 737.3403 | 1472.666 | 1472.665 | 64.62 | GDGGPPGATGFPGAAGR | 2 Oxidation (P) |
| 738.8519 | 1475.689 | 1475.689 | 58.2 | SGETGASGPPGFVGEK | |
| 746.8494 | 1491.684 | 1491.684 | 76.76 | SGETGASGPPGFVGEK | Oxidation (P) |
| 754.848 | 1507.681 | 1507.679 | 57.21 | SGETGASGPPGFVGEK | Oxidation (K); Oxidation (P) |
| 766.8937 | 1531.773 | 1531.774 | 69.77 | GEPGPAGAVGPAGAVGPR | Oxidation (P) |
| 511.5986 | 1531.774 | 1531.774 | 59.45 | GEPGPAGAVGPAGAVGPR | Oxidation (P) |
| 766.8946 | 1531.775 | 1531.774 | 64.41 | GEPGPAGAVGPAGAVGPR | Oxidation (P) |
| 766.8947 | 1531.775 | 1531.774 | 85.81 | GEPGPAGAVGPAGAVGPR | Oxidation (P) |
| 521.6142 | 1561.821 | 1561.821 | 56.79 | GAAGLPGVAGAPGLPGPR | 3 Oxidation (P) |
| 781.918 | 1561.821 | 1561.821 | 80.5 | GAAGLPGVAGAPGLPGPR | 3 Oxidation (P) |
| 781.918 | 1561.821 | 1561.821 | 60.51 | GAAGLPGVAGAPGLPGPR | 3 Oxidation (P) |
| 781.9183 | 1561.822 | 1561.821 | 40.84 | GAAGLPGVAGAPGLPGPR | 3 Oxidation (P) |
| 790.8864 | 1579.758 | 1579.759 | 72.46 | GPPGESGAAGPTGPIGSR | Oxidation (P) |
| 790.8864 | 1579.758 | 1579.759 | 46.98 | GPPGESGAAGPTGPIGSR | Oxidation (P) |
| 790.8867 | 1579.759 | 1579.759 | 58.92 | GPPGESGAAGPTGPIGSR | Oxidation (P) |
| 527.5936 | 1579.759 | 1579.759 | 58.01 | GPPGESGAAGPTGPIGSR | Oxidation (P) |
| 790.8868 | 1579.759 | 1579.759 | 74.22 | GPPGESGAAGPTGPIGSR | Oxidation (P) |
| 790.8875 | 1579.76 | 1579.759 | 41.69 | GPPGESGAAGPTGPIGSR | Oxidation (P) |
| 790.8876 | 1579.761 | 1579.759 | 67.14 | GPPGESGAAGPTGPIGSR | Oxidation (P) |
| 790.8877 | 1579.761 | 1579.759 | 66.66 | GPPGESGAAGPTGPIGSR | Oxidation (P) |
| 533.9457 | 1598.815 | 1598.817 | 54 | GELGPVGNPGPAGPAGPR | |
| 800.908 | 1599.801 | 1599.801 | 63.79 | GELGPVGNPGPAGPAGPR | Deamidated (NQ) |
| 804.344 | 1606.673 | 1606.676 | 48.76 | VYCDFSTGETCIR | |
| 539.2775 | 1614.811 | 1614.811 | 59.97 | GELGPVGNPGPAGPAGPR | Oxidation (P) |
| 539.2775 | 1614.811 | 1614.811 | 50.46 | GELGPVGNPGPAGPAGPR | Oxidation (P) |
| 808.413 | 1614.811 | 1614.811 | 50.42 | GELGPVGNPGPAGPAGPR | Oxidation (P) |
| 808.4133 | 1614.812 | 1614.811 | 86.07 | GELGPVGNPGPAGPAGPR | Oxidation (P) |
| 539.6054 | 1615.794 | 1615.795 | 73.32 | GELGPVGNPGPAGPAGPR | Deamidated (NQ); Oxidation (P) |
| 808.9046 | 1615.795 | 1615.795 | 51.33 | GELGPVGNPGPAGPAGPR | Deamidated (NQ); Oxidation (P) |
| 816.9207 | 1631.827 | 1631.827 | 54.21 | GSTGEIGPAGPPGPPGLR | Oxidation (P) |
| 550.2808 | 1647.821 | 1647.822 | 40.68 | GSTGEIGPAGPPGPPGLR | 2 Oxidation (P) |
| 824.9177 | 1647.821 | 1647.822 | 68.64 | GSTGEIGPAGPPGPPGLR | 2 Oxidation (P) |
| 824.9181 | 1647.822 | 1647.822 | 68.21 | GSTGEIGPAGPPGPPGLR | 2 Oxidation (P) |
| 600.6038 | 1798.79 | 1798.79 | 40.26 | GPNGDSGRPGEPGLMGPR | Deamidated (NQ); Oxidation (M); 2 Oxidation (P) |
| 602.3153 | 1803.924 | 1803.923 | 49.94 | RGSTGEIGPAGPPGPPGLR | 2 Oxidation (P) |
| 903.4304 | 1804.846 | 1804.845 | 53.44 | GPPGNVGNPGVNGAPGEAGR | 2 Oxidation (P) |
| 911.4277 | 1820.841 | 1820.84 | 66.89 | GPPGNVGNPGVNGAPGEAGR | 3 Oxidation (P) |
| 608.2819 | 1821.824 | 1821.824 | 50.03 | GPPGNVGNPGVNGAPGEAGR | Deamidated (NQ); 3 Oxidation (P) |
| 911.9203 | 1821.826 | 1821.824 | 78.98 | GPPGNVGNPGVNGAPGEAGR | Deamidated (NQ); 3 Oxidation (P) |
| 911.921 | 1821.827 | 1821.824 | 64.71 | GPPGNVGNPGVNGAPGEAGR | Deamidated (NQ); 3 Oxidation (P) |
| 912.4118 | 1822.809 | 1822.808 | 54.69 | GPPGNVGNPGVNGAPGEAGR | 2 Deamidated (NQ); 3 Oxidation (P) |
| 912.904 | 1823.793 | 1823.792 | 85.41 | GPPGNVGNPGVNGAPGEAGR | 3 Deamidated (NQ); 3 Oxidation (P) |
| 915.4553 | 1828.896 | 1828.896 | 63.1 | TGPPGPSGISGPPGPPGPAGK | Oxidation (K); 2 Oxidation (P) |
| 915.4557 | 1828.897 | 1828.896 | 43.19 | TGPPGPSGISGPPGPPGPAGK | Oxidation (K); 2 Oxidation (P) |
| 923.4523 | 1844.89 | 1844.891 | 45.24 | TGPPGPSGISGPPGPPGPAGK | Oxidation (K); 3 Oxidation (P) |
| 923.4526 | 1844.891 | 1844.891 | 44.13 | TGPPGPSGISGPPGPPGPAGK | 4 Oxidation (P) |
| 961.9692 | 1921.924 | 1921.924 | 56.9 | GERGPPGESGAAGPTGPIGSR | Oxidation (P) |
| 666.3405 | 1996 | 1995.999 | 63.71 | HGNRGEPGPAGAVGPAGAVGPR | Oxidation (P) |
| 999.0073 | 1996 | 1995.999 | 43.38 | HGNRGEPGPAGAVGPAGAVGPR | Oxidation (P) |
| 999.4988 | 1996.983 | 1996.983 | 80.07 | HGNRGEPGPAGAVGPAGAVGPR | Deamidated (NQ); Oxidation (P) |
| 666.6684 | 1996.983 | 1996.983 | 40.89 | HGNRGEPGPAGAVGPAGAVGPR | Deamidated (NQ); Oxidation (P) |
| 1004.473 | 2006.931 | 2006.929 | 67.25 | GEPGAVGQPGPPGPSGEEGKR | 3 Oxidation (P) |
| 1026.493 | 2050.971 | 2050.971 | 67.87 | GEVGPAGPNGFAGPAGAAGQPGAK | Deamidated (NQ); Oxidation (P) |
| 1026.984 | 2051.953 | 2051.955 | 74.36 | GEVGPAGPNGFAGPAGAAGQPGAK | 2 Deamidated (NQ); Oxidation (P) |
| 684.9923 | 2051.955 | 2051.955 | 46.29 | GEVGPAGPNGFAGPAGAAGQPGAK | 2 Deamidated (NQ); Oxidation (K) |
| 1026.986 | 2051.957 | 2051.955 | 79.62 | GEVGPAGPNGFAGPAGAAGQPGAK | 2 Deamidated (NQ); Oxidation (P) |
| 1028.55 | 2055.085 | 2055.086 | 49.35 | EGPVGLPGIDGRPGPIGPAGAR | Oxidation (P) |
| 1033.999 | 2065.983 | 2065.982 | 83.46 | GEVGPAGPNGFAGPAGAAGQPGAK | Oxidation (K); Oxidation (P) |
| 1034.49 | 2066.965 | 2066.966 | 50.61 | GEVGPAGPNGFAGPAGAAGQPGAK | Deamidated (NQ); Oxidation (K); Oxidation (P) |
| 689.996 | 2066.966 | 2066.966 | 48.63 | GEVGPAGPNGFAGPAGAAGQPGAK | Deamidated (NQ); Oxidation (K); Oxidation (P) |
| 1036.548 | 2071.081 | 2071.081 | 42.05 | EGPVGLPGIDGRPGPIGPAGAR | 2 Oxidation (P) |
| 705.7111 | 2114.112 | 2114.112 | 50.73 | GLPGVAGSVGEPGPLGIAGPPGAR | 2 Oxidation (P) |
| 1066.059 | 2130.103 | 2130.107 | 70.73 | GLPGVAGSVGEPGPLGIAGPPGAR | 3 Oxidation (P) |
| 1066.059 | 2130.103 | 2130.107 | 66.99 | GLPGVAGSVGEPGPLGIAGPPGAR | 3 Oxidation (P) |
| 1066.059 | 2130.103 | 2130.107 | 53.23 | GLPGVAGSVGEPGPLGIAGPPGAR | 3 Oxidation (P) |
| 1066.06 | 2130.105 | 2130.107 | 80.25 | GLPGVAGSVGEPGPLGIAGPPGAR | 3 Oxidation (P) |
| 1066.06 | 2130.105 | 2130.107 | 88.4 | GLPGVAGSVGEPGPLGIAGPPGAR | 3 Oxidation (P) |
| 711.0427 | 2130.106 | 2130.107 | 48.89 | GLPGVAGSVGEPGPLGIAGPPGAR | 3 Oxidation (P) |
| 711.0429 | 2130.107 | 2130.107 | 45.37 | GLPGVAGSVGEPGPLGIAGPPGAR | 3 Oxidation (P) |
| 1066.061 | 2130.107 | 2130.107 | 82.47 | GLPGVAGSVGEPGPLGIAGPPGAR | 3 Oxidation (P) |
| 1066.061 | 2130.107 | 2130.107 | 101.31 | GLPGVAGSVGEPGPLGIAGPPGAR | 3 Oxidation (P) |
| 1066.061 | 2130.107 | 2130.107 | 95.28 | GLPGVAGSVGEPGPLGIAGPPGAR | 3 Oxidation (P) |
| 1066.061 | 2130.107 | 2130.107 | 78.83 | GLPGVAGSVGEPGPLGIAGPPGAR | 3 Oxidation (P) |
| 1066.061 | 2130.107 | 2130.107 | 80.74 | GLPGVAGSVGEPGPLGIAGPPGAR | 3 Oxidation (P) |
| 1066.061 | 2130.107 | 2130.107 | 64.05 | GLPGVAGSVGEPGPLGIAGPPGAR | 3 Oxidation (P) |
| 711.0433 | 2130.108 | 2130.107 | 56.59 | GLPGVAGSVGEPGPLGIAGPPGAR | 3 Oxidation (P) |
| 1066.062 | 2130.109 | 2130.107 | 86.56 | GLPGVAGSVGEPGPLGIAGPPGAR | 3 Oxidation (P) |
| 1066.062 | 2130.109 | 2130.107 | 71.63 | GLPGVAGSVGEPGPLGIAGPPGAR | 3 Oxidation (P) |
| 1066.062 | 2130.109 | 2130.107 | 103.82 | GLPGVAGSVGEPGPLGIAGPPGAR | 3 Oxidation (P) |
| 1066.062 | 2130.109 | 2130.107 | 97.69 | GLPGVAGSVGEPGPLGIAGPPGAR | 3 Oxidation (P) |
| 1066.064 | 2130.113 | 2130.107 | 73.86 | GLPGVAGSVGEPGPLGIAGPPGAR | 3 Oxidation (P) |
| 1066.065 | 2130.115 | 2130.107 | 91.54 | GLPGVAGSVGEPGPLGIAGPPGAR | 3 Oxidation (P) |
| 1074.058 | 2146.101 | 2146.102 | 44.98 | GLPGVAGSVGEPGPLGIAGPPGAR | 4 Oxidation (P) |
| 1074.059 | 2146.103 | 2146.102 | 67.5 | GLPGVAGSVGEPGPLGIAGPPGAR | 4 Oxidation (P) |
| 1074.059 | 2146.103 | 2146.102 | 66.55 | GLPGVAGSVGEPGPLGIAGPPGAR | 4 Oxidation (P) |
| 1074.059 | 2146.103 | 2146.102 | 74.98 | GLPGVAGSVGEPGPLGIAGPPGAR | 4 Oxidation (P) |
| 1074.059 | 2146.103 | 2146.102 | 40.73 | GLPGVAGSVGEPGPLGIAGPPGAR | 4 Oxidation (P) |
| 1074.061 | 2146.107 | 2146.102 | 74.04 | GLPGVAGSVGEPGPLGIAGPPGAR | 4 Oxidation (P) |
| 1131.068 | 2260.121 | 2260.124 | 54.64 | GYPGNAGPVGAAGAPGPQGPVGPVGK | 2 Oxidation (P) |
| 754.3815 | 2260.123 | 2260.124 | 44.13 | GYPGNAGPVGAAGAPGPQGPVGPVGK | 2 Oxidation (P) |
| 1131.069 | 2260.123 | 2260.124 | 77.94 | GYPGNAGPVGAAGAPGPQGPVGPVGK | 2 Oxidation (P) |
| 1131.069 | 2260.123 | 2260.124 | 69.33 | GYPGNAGPVGAAGAPGPQGPVGPVGK | 2 Oxidation (P) |
| 1131.071 | 2260.127 | 2260.124 | 79.4 | GYPGNAGPVGAAGAPGPQGPVGPVGK | 2 Oxidation (P) |
| 804.0512 | 2409.132 | 2409.131 | 50.01 | GEVGPAGPNGFAGPAGAAGQPGAKGER | Deamidated (NQ); Oxidation (K); Oxidation (P) |
| 1206.064 | 2410.113 | 2410.115 | 68.53 | GEVGPAGPNGFAGPAGAAGQPGAKGER | 2 Deamidated (NQ); Oxidation (K); Oxidation (P) |
| 1215.618 | 2429.221 | 2429.219 | 71.17 | GEVGLPGLSGPVGPPGNPGANGLPGAK | 4 Oxidation (P) |
| 1216.109 | 2430.203 | 2430.203 | 81.79 | GEVGLPGLSGPVGPPGNPGANGLPGAK | Deamidated (NQ); 4 Oxidation (P) |
| 1223.613 | 2445.211 | 2445.214 | 61.7 | GEVGLPGLSGPVGPPGNPGANGLPGAK | Oxidation (K); 4 Oxidation (P) |
| 816.4064 | 2446.197 | 2446.198 | 55.93 | GEVGLPGLSGPVGPPGNPGANGLPGAK | Deamidated (NQ); Oxidation (K); 4 Oxidation (P) |
| 1224.107 | 2446.199 | 2446.198 | 78.17 | GEVGLPGLSGPVGPPGNPGANGLPGAK | Deamidated (NQ); Oxidation (K); 4 Oxidation (P) |
| 1287.136 | 2572.257 | 2572.256 | 96.6 | GSDGSVGPVGPAGPIGSAGPPGFPGAPGPK | Oxidation (K); Oxidation (P) |
| 861.0641 | 2580.171 | 2580.169 | 40.35 | GESGNKGEPGAVGQPGPPGPSGEEGKR | Deamidated (NQ); 3 Oxidation (P) |
| 861.0858 | 2580.236 | 2580.232 | 42.85 | GENGPVGPTGPVGAAGPSGPNGPPGPAGSR | Oxidation (P) |
| 861.4128 | 2581.217 | 2581.216 | 62.17 | GENGPVGPTGPVGAAGPSGPNGPPGPAGSR | Deamidated (NQ); Oxidation (P) |
| 1292.107 | 2582.199 | 2582.2 | 72.6 | GENGPVGPTGPVGAAGPSGPNGPPGPAGSR | 2 Deamidated (NQ); Oxidation (P) |
| 1292.108 | 2582.201 | 2582.2 | 96.95 | GENGPVGPTGPVGAAGPSGPNGPPGPAGSR | 2 Deamidated (NQ); Oxidation (P) |
| 863.7574 | 2588.25 | 2588.251 | 50.11 | GSDGSVGPVGPAGPIGSAGPPGFPGAPGPK | 3 Oxidation (P) |
| 884.7482 | 2651.223 | 2651.221 | 43.88 | GSPGERGEVGPAGPNGFAGPAGAAGQPGAK | 2 Deamidated (NQ); 2 Oxidation (P) |
| 931.4457 | 2791.315 | 2791.316 | 56.21 | GEQGPAGPPGFQGLPGPAGTAGEAGKPGER | Oxidation (K); 2 Oxidation (P) |
| 931.774 | 2792.3 | 2792.3 | 63.44 | GEQGPAGPPGFQGLPGPAGTAGEAGKPGER | Deamidated (NQ); Oxidation (K); 2 Oxidation (P) |
| 955.7978 | 2864.372 | 2864.369 | 54.78 | GPKGENGPVGPTGPVGAAGPSGPNGPPGPAGSR | 2 Deamidated (NQ); Oxidation (P) |
| 960.8008 | 2879.381 | 2879.38 | 79.87 | GPKGENGPVGPTGPVGAAGPSGPNGPPGPAGSR | Deamidated (NQ); Oxidation (K); Oxidation (P) |
| 961.1298 | 2880.368 | 2880.364 | 89.34 | GPKGENGPVGPTGPVGAAGPSGPNGPPGPAGSR | 2 Deamidated (NQ); Oxidation (K); Oxidation (P) |
| 996.7743 | 2987.301 | 2987.299 | 52.86 | GPPGNVGNPGVNGAPGEAGRDGNPGNDGPPGR | Deamidated (NQ); 5 Oxidation (P) |
| 1003.806 | 3008.396 | 3008.397 | 46.86 | GSPGERGEVGPAGPNGFAGPAGAAGQPGAKGER | Deamidated (NQ); Oxidation (K); 2 Oxidation (P) |
| 1056.188 | 3165.542 | 3165.544 | 42.54 | GTKGPKGENGPVGPTGPVGAAGPSGPNGPPGPAGSR | Deamidated (NQ); Oxidation (K); Oxidation (P) |
| 1126.22 | 3375.638 | 3375.633 | 43.04 | GPSGEPGTAGPPGTPGPQGLLGAPGFLGLPGSRGER | Deamidated (NQ); 5 Oxidation (P) |
| 1127.913 | 3380.717 | 3380.709 | 41.21 | GLPGLKGHNGLQGLPGLAGHHGDQGAPGAVGPAGPR | Oxidation (K); 3 Oxidation (P) |
| 1222.535 | 3664.583 | 3664.577 | 50.91 | GSQGSQGPAGPPGPPGPPGPPGPSGGGYEFGFDGDFYR | 4 Oxidation (P) |

| Chondroadherin OS=Bos taurus GN=CHAD PE=1 SV=1 | 866 |
| --- | --- |

| 410.7191 | 819.4236 | 819.4239 | 44.79 | EVAAGAFR | |
| --- | --- | --- | --- | --- | --- |
| 669.8249 | 1337.635 | 1337.636 | 44.31 | SIPDNAFQSFGR | |
| 678.3638 | 1354.713 | 1354.713 | 99.12 | FSDGAFLGVTTLK | |
| 678.3639 | 1354.713 | 1354.713 | 52.92 | FSDGAFLGVTTLK | |
| 678.3642 | 1354.714 | 1354.713 | 98.77 | FSDGAFLGVTTLK | |
| 678.3642 | 1354.714 | 1354.713 | 84.75 | FSDGAFLGVTTLK | |
| 678.3643 | 1354.714 | 1354.713 | 42.17 | FSDGAFLGVTTLK | |
| 678.3644 | 1354.714 | 1354.713 | 70.67 | FSDGAFLGVTTLK | |
| 678.3644 | 1354.714 | 1354.713 | 66.7 | FSDGAFLGVTTLK | |
| 683.3539 | 1364.693 | 1364.694 | 47.21 | NQLSSYPSAALSK | |
| 876.4451 | 1750.876 | 1750.878 | 101.11 | YLETLWLDNTNLEK | |
| 876.9379 | 1751.861 | 1751.862 | 61.26 | YLETLWLDNTNLEK | Deamidated (NQ) |
| 877.4297 | 1752.845 | 1752.846 | 48.96 | YLETLWLDNTNLEK | 2 Deamidated (NQ) |
| 877.4301 | 1752.846 | 1752.846 | 67.11 | YLETLWLDNTNLEK | 2 Deamidated (NQ) |
| 1005.591 | 2009.167 | 2009.167 | 80.63 | GLLSPLVNLFILQLNNNK | |
| 1013.59 | 2025.165 | 2025.162 | 53.31 | GLLSPLVNLFILQLNNNK | Oxidation (P) |
| 1031.019 | 2060.023 | 2060.021 | 78.08 | FYLDRNQLSSYPSAALSK | Deamidated (NQ) |
| 1031.021 | 2060.027 | 2060.021 | 42.01 | FYLDRNQLSSYPSAALSK | Deamidated (NQ) |
| 1100.033 | 2198.051 | 2198.053 | 90.69 | AGAFDDLTELTYLYLDHNK | |
| 1141.166 | 2280.317 | 2280.321 | 78.43 | GLLSPLVNLFILQLNNNKIR | 2 Deamidated (NQ) |
| 1149.656 | 2297.297 | 2297.299 | 46.87 | GLLSPLVNLFILQLNNNKIR | 3 Deamidated (NQ); Oxidation (P) |
| 1381.698 | 2761.381 | 2761.381 | 57.93 | WLYLSENSLSSLQPGALDDVENLAK | |
| 1406.718 | 2811.421 | 2811.423 | 60.72 | LHQLPSNFPFDSLETLTLTNNPWK | |

| Biglycan OS=Bos taurus GN=BGN PE=1 SV=3 | 861 |
| --- | --- |

| 455.2737 | 908.5328 | 908.5331 | 46.97 | VPAGLPDLK | |
| --- | --- | --- | --- | --- | --- |
| 455.2739 | 908.5332 | 908.5331 | 46.14 | VPAGLPDLK | |
| 455.274 | 908.5334 | 908.5331 | 48.34 | VPAGLPDLK | |
| 504.7827 | 1007.551 | 1007.551 | 61.8 | LGLGHNQIR | Deamidated (NQ) |
| 504.783 | 1007.551 | 1007.551 | 40.66 | LGLGHNQIR | Deamidated (NQ) |
| 505.2748 | 1008.535 | 1008.535 | 61.55 | LGLGHNQIR | 2 Deamidated (NQ) |
| 527.7816 | 1053.549 | 1053.55 | 45.69 | LAIQFGNYK | Deamidated (NQ) |
| 559.7969 | 1117.579 | 1117.58 | 42.08 | VVQCSDLGLK | |
| 399.8961 | 1196.667 | 1196.667 | 49.21 | IHEKAFSPLR | |
| 748.3644 | 1494.714 | 1494.718 | 48.42 | VGVNDFCPVGFGVK | Deamidated (NQ) |
| 748.366 | 1494.717 | 1494.718 | 55.31 | VGVNDFCPVGFGVK | Deamidated (NQ) |
| 748.3665 | 1494.718 | 1494.718 | 55.33 | VGVNDFCPVGFGVK | Deamidated (NQ) |
| 748.3668 | 1494.719 | 1494.718 | 54.77 | VGVNDFCPVGFGVK | Deamidated (NQ) |
| 789.9137 | 1577.813 | 1577.812 | 75.41 | MIENGSLSFLPTLR | Deamidated (NQ) |
| 789.9137 | 1577.813 | 1577.812 | 47.26 | MIENGSLSFLPTLR | Deamidated (NQ) |
| 789.9144 | 1577.814 | 1577.812 | 69.75 | MIENGSLSFLPTLR | Deamidated (NQ) |
| 791.4595 | 1580.904 | 1580.904 | 73 | GLQHLYALVLVNNK | |
| 791.9518 | 1581.889 | 1581.888 | 42.25 | GLQHLYALVLVNNK | Deamidated (NQ) |
| 792.4428 | 1582.871 | 1582.872 | 49.65 | GLQHLYALVLVNNK | 2 Deamidated (NQ) |
| 792.4433 | 1582.872 | 1582.872 | 62.26 | GLQHLYALVLVNNK | 2 Deamidated (NQ) |
| 792.4435 | 1582.872 | 1582.872 | 56.53 | GLQHLYALVLVNNK | 2 Deamidated (NQ) |
| 797.9105 | 1593.806 | 1593.807 | 72.24 | MIENGSLSFLPTLR | Deamidated (NQ); Oxidation (M) |
| 797.9113 | 1593.808 | 1593.807 | 83.86 | MIENGSLSFLPTLR | Deamidated (NQ); Oxidation (M) |
| 797.9114 | 1593.808 | 1593.807 | 63.69 | MIENGSLSFLPTLR | Deamidated (NQ); Oxidation (M) |
| 797.9117 | 1593.809 | 1593.807 | 48.71 | MIENGSLSFLPTLR | Deamidated (NQ); Oxidation (M) |
| 956.549 | 1911.083 | 1911.083 | 78.23 | GLQHLYALVLVNNKISK | 2 Deamidated (NQ) |
| 676.7087 | 2027.104 | 2027.105 | 43.01 | NHLVEIPPNLPSSLVELR | Deamidated (NQ) |
| 1424.193 | 2846.371 | 2846.37 | 69.45 | AYYNGISLFNNPVPYWEVQPATFR | Deamidated (NQ) |

| Serum albumin OS=Bos taurus GN=ALB PE=1 SV=4 | 513 |
| --- | --- |

| 501.7953 | 1001.576 | 1001.576 | 53.38 | LVVSTQTALA | |
| --- | --- | --- | --- | --- | --- |
| 507.8129 | 1013.611 | 1013.612 | 41 | QTALVELLK | |
| 508.3053 | 1014.596 | 1014.596 | 40.86 | QTALVELLK | Deamidated (NQ) |
| 700.3512 | 1398.688 | 1398.685 | 49.88 | TVMENFVAFVDK | |
| 480.6087 | 1438.804 | 1438.805 | 63.86 | RHPEYAVSVLLR | |
| 740.4013 | 1478.788 | 1478.788 | 80.23 | LGEYGFQNALIVR | |
| 740.8937 | 1479.773 | 1479.772 | 85.03 | LGEYGFQNALIVR | Deamidated (NQ) |
| 740.8939 | 1479.773 | 1479.772 | 98.17 | LGEYGFQNALIVR | Deamidated (NQ) |
| 741.3857 | 1480.757 | 1480.756 | 81.82 | LGEYGFQNALIVR | 2 Deamidated (NQ) |
| 784.3744 | 1566.734 | 1566.735 | 84.03 | DAFLGSFLYEYSR | |
| 784.3762 | 1566.738 | 1566.735 | 80.1 | DAFLGSFLYEYSR | |
| 1023.017 | 2044.019 | 2044.021 | 42.44 | RHPYFYAPELLYYANK | |
| 682.3476 | 2044.021 | 2044.021 | 60.27 | RHPYFYAPELLYYANK | |
| 1246.635 | 2491.255 | 2491.257 | 59.38 | GLVLIAFSQYLQQCPFDEHVK | |

| Alpha-2-HS-glycoprotein OS=Bos taurus GN=AHSG PE=1 SV=2 | 460 |
| --- | --- |

| 578.7956 | 1155.577 | 1155.577 | 47.45 | HTLNQIDSVK | 2 Deamidated (NQ) |
| --- | --- | --- | --- | --- | --- |
| 635.3275 | 1268.64 | 1268.64 | 54.7 | QDGQFSVLFTK | |
| 635.3277 | 1268.641 | 1268.64 | 70.84 | QDGQFSVLFTK | |
| 635.8189 | 1269.623 | 1269.624 | 40.3 | QDGQFSVLFTK | Deamidated (NQ) |
| 635.819 | 1269.623 | 1269.624 | 44.66 | QDGQFSVLFTK | Deamidated (NQ) |
| 635.8193 | 1269.624 | 1269.624 | 44.6 | QDGQFSVLFTK | Deamidated (NQ) |
| 635.8194 | 1269.624 | 1269.624 | 57.42 | QDGQFSVLFTK | Deamidated (NQ) |
| 737.9222 | 1473.83 | 1473.83 | 62.95 | TPIVGQPSIPGGPVR | |
| 737.923 | 1473.831 | 1473.83 | 43.46 | TPIVGQPSIPGGPVR | |
| 737.923 | 1473.831 | 1473.83 | 46.81 | TPIVGQPSIPGGPVR | |
| 738.4144 | 1474.814 | 1474.814 | 44.2 | TPIVGQPSIPGGPVR | Deamidated (NQ) |
| 1260.163 | 2518.311 | 2518.314 | 102.54 | AQFVPLPVSVSVEFAVAATDCIAK | |
| 840.4457 | 2518.315 | 2518.314 | 52.48 | AQFVPLPVSVSVEFAVAATDCIAK | |
| 1260.166 | 2518.317 | 2518.314 | 98.18 | AQFVPLPVSVSVEFAVAATDCIAK | |

| Pigment epithelium-derived factor OS=Bos taurus GN=SERPINF1 PE=1 SV=1 | 407 |
| --- | --- |

| 625.8345 | 1249.654 | 1249.655 | 48.66 | DTDTGALLFIGK | |
| --- | --- | --- | --- | --- | --- |
| 625.8351 | 1249.656 | 1249.655 | 82.34 | DTDTGALLFIGK | |
| 684.3459 | 1366.677 | 1366.677 | 40.71 | LQSLFDAPDFSK | |
| 758.8435 | 1515.672 | 1515.673 | 79.94 | TSLEDFYLDEER | |
| 520.6 | 1558.778 | 1558.778 | 52.44 | LAAAVSNFGYDLYR | |
| 780.3975 | 1558.78 | 1558.778 | 72.14 | LAAAVSNFGYDLYR | |
| 780.885 | 1559.755 | 1559.762 | 43 | LAAAVSNFGYDLYR | Deamidated (NQ) |
| 661.6669 | 1981.979 | 1981.979 | 46.64 | ALYYDLISNPDIHGTYK | |
| 1037.6 | 2073.185 | 2073.187 | 116.84 | IAQLPLTGSTSIIFFLPQK | |
| 745.0759 | 2232.206 | 2232.21 | 41.83 | LTFPLDYHLNQPFIFVLR | |
| 1117.112 | 2232.209 | 2232.21 | 54.35 | LTFPLDYHLNQPFIFVLR | |

| Complement C3 OS=Bos taurus GN=C3 PE=1 SV=2 | 396 |
| --- | --- |

| 634.8664 | 1267.718 | 1267.718 | 45.81 | LVAYYTLINAK | |
| --- | --- | --- | --- | --- | --- |
| 635.3582 | 1268.702 | 1268.702 | 51.71 | LVAYYTLINAK | Deamidated (NQ) |
| 686.4161 | 1370.818 | 1370.817 | 51.34 | SSVAVPYVIVPLK | |
| 723.4217 | 1444.829 | 1444.829 | 83.65 | VFALAANLIAIDSK | |
| 819.4489 | 1636.883 | 1636.883 | 102.29 | FVTVVATFGNVQVEK | |
| 597.6878 | 1790.042 | 1790.045 | 53.36 | RPYTVAIAAYALALLGK | |
| 898.967 | 1795.919 | 1795.918 | 46.56 | VELLYNPAFCSLATAK | |
| 627.6875 | 1880.041 | 1880.041 | 64.94 | LYNVEATSYALLALLAR | |
| 941.5195 | 1881.024 | 1881.025 | 97.99 | LYNVEATSYALLALLAR | Deamidated (NQ) |
| 684.7036 | 2051.089 | 2051.087 | 43.65 | VRVELLYNPAFCSLATAK | |

| Asporin OS=Bos taurus GN=ASPN PE=2 SV=1 | 325 |
| --- | --- |

| 648.8221 | 1295.63 | 1295.63 | 40.31 | YWEVQPATFR | |
| --- | --- | --- | --- | --- | --- |
| 767.434 | 1532.853 | 1532.856 | 67.48 | GLTSLYALILNNNK | |
| 839.4641 | 1676.914 | 1676.914 | 85.13 | YLQIIFLHSNSITK | Deamidated (NQ) |
| 839.4643 | 1676.914 | 1676.914 | 93.75 | YLQIIFLHSNSITK | Deamidated (NQ) |
| 839.9561 | 1677.898 | 1677.898 | 74.35 | YLQIIFLHSNSITK | 2 Deamidated (NQ) |
| 627.0186 | 1878.034 | 1878.035 | 52.72 | GLTSLYALILNNNKLTK | 3 Deamidated (NQ) |
| 660.3705 | 1978.09 | 1978.089 | 53.83 | LYLSHNQLSEIPLNLPK | |
| 712.4042 | 2134.191 | 2134.19 | 40.07 | RLYLSHNQLSEIPLNLPK | |
| 712.7316 | 2135.173 | 2135.174 | 42.09 | RLYLSHNQLSEIPLNLPK | Deamidated (NQ) |

| Collagen alpha-1(II) chain OS=Bos taurus GN=COL2A1 PE=1 SV=4 | 296 |
| --- | --- |

| 656.8303 | 1311.646 | 1311.646 | 42.62 | GFPGLPGPSGEPGK | Oxidation (P) |
| --- | --- | --- | --- | --- | --- |
| 656.8307 | 1311.647 | 1311.646 | 63.35 | GFPGLPGPSGEPGK | Oxidation (P) |
| 664.8275 | 1327.64 | 1327.641 | 50.53 | GFPGLPGPSGEPGK | 2 Oxidation (P) |
| 664.8275 | 1327.64 | 1327.641 | 46.53 | GFPGLPGPSGEPGK | 2 Oxidation (P) |
| 664.8276 | 1327.641 | 1327.641 | 61.01 | GFPGLPGPSGEPGK | 2 Oxidation (P) |
| 664.8276 | 1327.641 | 1327.641 | 72.11 | GFPGLPGPSGEPGK | 2 Oxidation (P) |
| 664.8276 | 1327.641 | 1327.641 | 69.32 | GFPGLPGPSGEPGK | 2 Oxidation (P) |
| 664.8279 | 1327.641 | 1327.641 | 43.25 | GFPGLPGPSGEPGK | 2 Oxidation (P) |
| 672.8248 | 1343.635 | 1343.636 | 43.09 | GFPGLPGPSGEPGK | 3 Oxidation (P) |
| 672.8249 | 1343.635 | 1343.636 | 52.54 | GFPGLPGPSGEPGK | 3 Oxidation (P) |
| 672.8251 | 1343.636 | 1343.636 | 64.48 | GFPGLPGPSGEPGK | 3 Oxidation (P) |
| 672.8253 | 1343.636 | 1343.636 | 57.32 | GFPGLPGPSGEPGK | 3 Oxidation (P) |
| 672.8254 | 1343.636 | 1343.636 | 40.91 | GFPGLPGPSGEPGK | 3 Oxidation (P) |
| 758.8599 | 1515.705 | 1515.707 | 42.97 | GAQGPPGATGFPGAAGR | 3 Oxidation (P) |
| 758.86 | 1515.705 | 1515.707 | 46.67 | GAQGPPGATGFPGAAGR | 3 Oxidation (P) |

| Collagen alpha-2(XI) chain OS=Bos taurus GN=COL11A2 PE=3 SV=1 | 287 |
| --- | --- |

| 615.8276 | 1229.641 | 1229.641 | 47.9 | LGVPGLPGYPGR | 3 Oxidation (P) |
| --- | --- | --- | --- | --- | --- |
| 615.8278 | 1229.641 | 1229.641 | 49.86 | LGVPGLPGYPGR | 3 Oxidation (P) |
| 617.3294 | 1232.644 | 1232.644 | 44.2 | GQSVTLIIDCK | |
| 671.0538 | 2010.14 | 2010.137 | 45.01 | ARPGLQAPLLTLYSAQGVR | |
| 1006.078 | 2010.141 | 2010.137 | 65 | ARPGLQAPLLTLYSAQGVR | |
| 1006.566 | 2011.117 | 2011.121 | 41.41 | ARPGLQAPLLTLYSAQGVR | Deamidated (NQ) |
| 671.3811 | 2011.122 | 2011.121 | 57.69 | ARPGLQAPLLTLYSAQGVR | Deamidated (NQ) |
| 671.3814 | 2011.122 | 2011.121 | 63.96 | ARPGLQAPLLTLYSAQGVR | Deamidated (NQ) |
| 671.7094 | 2012.106 | 2012.105 | 52.5 | ARPGLQAPLLTLYSAQGVR | 2 Deamidated (NQ) |

| Lumican OS=Bos taurus GN=LUM PE=1 SV=1 | 281 |
| --- | --- |

| 499.2765 | 996.5384 | 996.5392 | 49.07 | FSALQYLR | |
| --- | --- | --- | --- | --- | --- |
| 499.2766 | 996.5386 | 996.5392 | 49.46 | FSALQYLR | |
| 499.2767 | 996.5388 | 996.5392 | 49.38 | FSALQYLR | |
| 499.2768 | 996.539 | 996.5392 | 42.67 | FSALQYLR | |
| 499.2768 | 996.539 | 996.5392 | 51.82 | FSALQYLR | |
| 499.277 | 996.5394 | 996.5392 | 46.9 | FSALQYLR | |
| 499.769 | 997.5234 | 997.5233 | 52.09 | FSALQYLR | Deamidated (NQ) |
| 844.9147 | 1687.815 | 1687.813 | 80.63 | SLEYLDLSFNQMTK | |
| 845.406 | 1688.797 | 1688.797 | 40.05 | SLEYLDLSFNQMTK | Deamidated (NQ) |
| 845.407 | 1688.799 | 1688.797 | 70.48 | SLEYLDLSFNQMTK | Deamidated (NQ) |
| 852.9113 | 1703.808 | 1703.808 | 64.13 | SLEYLDLSFNQMTK | Oxidation (M) |
| 979.0526 | 1956.091 | 1956.093 | 72.61 | LPSGLPVSLLTLYLDNNK | |

| Serine protease HTRA1 OS=Bos taurus GN=HTRA1 PE=2 SV=1 | 214 |
| --- | --- |

| 440.8025 | 879.5904 | 879.5906 | 51.07 | LPVLLLGR |  |
| --- | --- | --- | --- | --- | --- |
| 584.7923 | 1167.57 | 1167.571 | 41.46 | TYTNLCQLR | |
| 653.3561 | 1304.698 | 1304.698 | 55.64 | VTAGISFAIPSDK | |
| 682.9063 | 1363.798 | 1363.798 | 63.94 | IAPAVVHIELFR | |
| 520.3192 | 1557.936 | 1557.936 | 40.1 | IDHQGKLPVLLLGR | |
| 1160.61 | 3478.808 | 3478.794 | 53.49 | SSELRPGEFVVAIGSPFSLQNTVTTGIVSTTQR | Deamidated (NQ) |

| Vitrin OS=Bos taurus GN=VIT PE=2 SV=2 | 204 |
| --- | --- |

| 516.2997 | 1030.585 | 1030.585 | 45.71 | LMILITDGR |
| --- | --- | --- | --- | --- |
| 624.3593 | 1246.704 | 1246.703 | 58.68 | TVLQFVANLSR |
| 769.41 | 1536.805 | 1536.804 | 91.1 | VDLSFLIDGSSSIGK |
| 1228.613 | 2455.211 | 2455.217 | 106.31 | VGYWSGGTSTGAAIHYALEQLFK |

| SPARC OS=Bos taurus GN=SPARC PE=1 SV=2 | 196 |
| --- | --- |

| 608.3126 | 1214.611 | 1214.612 | 46.65 | LHLDYIGPCK |
| --- | --- | --- | --- | --- |
| 710.3832 | 1418.752 | 1418.752 | 44.43 | LEAGDHPVELLAR |
| 821.8996 | 1641.785 | 1641.786 | 59.3 | YIALDEWAGCFGIK |
| 821.9011 | 1641.788 | 1641.786 | 88.44 | YIALDEWAGCFGIK |
| 821.9012 | 1641.788 | 1641.786 | 56.43 | YIALDEWAGCFGIK |
| 975.4882 | 1948.962 | 1948.96 | 46.26 | YIPPCLDSELTEFPLR |

| Prothrombin OS=Bos taurus GN=F2 PE=1 SV=2 | 195 |
| --- | --- |

| 446.2483 | 890.482 | 890.4821 | 43.69 | LAVTTSGSR |
| --- | --- | --- | --- | --- |
| 456.7791 | 911.5436 | 911.544 | 48.52 | VTVEVIPR |
| 621.8035 | 1241.592 | 1241.593 | 54.44 | ELFESYIEGR |
| 823.4167 | 1644.819 | 1644.814 | 64.42 | SPQELLCGASLISDR |
| 1137.581 | 2273.147 | 2273.151 | 93.87 | IVEGQDAEVGLSPWQVMLFR |

| 72 kDa type IV collagenase OS=Bos taurus GN=MMP2 PE=2 SV=1 | 191 |
| --- | --- |

| 709.875 | 1417.735 | 1417.735 | 54.62 | AFQVWSDVTPLR |
| --- | --- | --- | --- | --- |
| 709.8752 | 1417.736 | 1417.735 | 57.81 | AFQVWSDVTPLR |
| 1105.53 | 2209.045 | 2209.048 | 76.74 | AVFFAGNEYWVYSASTLER |
| 1105.533 | 2209.051 | 2209.048 | 101.47 | AVFFAGNEYWVYSASTLER |

| Vitamin K-dependent protein S OS=Bos taurus GN=PROS1 PE=1 SV=1 | 181 |
| --- | --- |

| 403.7295 | 805.4444 | 805.4446 | 56.45 | AGLFTAAR | |
| --- | --- | --- | --- | --- | --- |
| 509.7346 | 1017.455 | 1017.456 | 45.73 | FSAEFDFR | |
| 829.9595 | 1657.904 | 1657.907 | 62.76 | LQDILVSVESMVIGR | |
| 558.9747 | 1673.902 | 1673.902 | 41.96 | LQDILVSVESMVIGR | Oxidation (M) |
| 837.9592 | 1673.904 | 1673.902 | 68.9 | LQDILVSVESMVIGR | Oxidation (M) |
| 926.4498 | 1850.885 | 1850.884 | 67.65 | GSYYPGTGVAQFSINYK | |

| Apolipoprotein A-I OS=Bos taurus GN=APOA1 PE=1 SV=3 | 167 |
| --- | --- |

| 608.8426 | 1215.671 | 1215.671 | 60.99 | VSILAAIDEASK | |
| --- | --- | --- | --- | --- | --- |
| 699.8493 | 1397.684 | 1397.683 | 69.71 | DYVAQFEASALGK | |
| 789.407 | 1576.799 | 1576.798 | 101.55 | LLDNWDTLASTLSK | Deamidated (NQ) |

| Olfactomedin-like protein 3 OS=Bos taurus GN=OLFML3 PE=2 SV=1 | 164 |
| --- | --- |

| 632.8445 | 1263.674 | 1263.676 | 41.93 | LRDFTLAMAAR |
| --- | --- | --- | --- | --- |
| 881.4276 | 1760.841 | 1760.841 | 62.68 | QLYAWDDGYQIVYK |
| 1195.618 | 2389.221 | 2389.226 | 99.53 | VPFPWVGTGQLVYGGFLYYAR |
| 797.4152 | 2389.224 | 2389.226 | 41.65 | VPFPWVGTGQLVYGGFLYYAR |

| Nucleobindin-1 OS=Bos taurus GN=NUCB1 PE=2 SV=1 | 158 |
| --- | --- |

| 636.8611 | 1271.708 | 1271.709 | 67.93 | DLELLIQTATR | |
| --- | --- | --- | --- | --- | --- |
| 637.3537 | 1272.693 | 1272.693 | 81.86 | DLELLIQTATR | Deamidated (NQ) |
| 753.9113 | 1505.808 | 1505.809 | 69.01 | LVTLEEFLASTQR | |
| 644.9988 | 1931.975 | 1931.974 | 44.73 | YLQEVINVLETDGHFR | |
| 645.3256 | 1932.955 | 1932.958 | 46.86 | YLQEVINVLETDGHFR | Deamidated (NQ) |

| Collagen alpha-1(III) chain OS=Bos taurus GN=COL3A1 PE=1 SV=1 | 154 |
| --- | --- |

| 645.2974 | 1288.58 | 1288.58 | 68.15 | GSPGGPGAAGFPGGR | 3 Oxidation (P) |
| --- | --- | --- | --- | --- | --- |
| 500.9113 | 1499.712 | 1499.712 | 43.83 | DGASGHPGPIGPPGPR | 2 Oxidation (P) |
| 818.9269 | 1635.839 | 1635.84 | 66.64 | GEMGPAGIPGAPGLIGAR | Oxidation (P) |
| 1041.559 | 2081.103 | 2081.102 | 66.12 | GAPGPQGPPGAPGPLGIAGLTGAR | Oxidation (P) |

| Dermatopontin OS=Bos taurus GN=DPT PE=1 SV=3 | 154 |
| --- | --- |

| 570.2883 | 1138.562 | 1138.562 | 60.19 | GATTTFSAVER | |
| --- | --- | --- | --- | --- | --- |
| 753.332 | 2256.974 | 2256.972 | 42.37 | YFESVLDREWQFYCCR | |
| 753.6586 | 2257.954 | 2257.956 | 51.13 | YFESVLDREWQFYCCR | Deamidated (NQ) |
| 1188.531 | 2375.047 | 2375.042 | 81.29 | AGMEWYQTCSNNGLVAGFQSR | |

| Tetranectin OS=Bos taurus GN=CLEC3B PE=2 SV=1 | 148 |
| --- | --- |

| 726.8815 | 1451.748 | 1451.748 | 55.52 | DKLPYVCQFAIV | |
| --- | --- | --- | --- | --- | --- |
| 764.9346 | 1527.855 | 1527.851 | 76.3 | TQLDSLAQEVALLK | |
| 765.4249 | 1528.835 | 1528.835 | 81.89 | TQLDSLAQEVALLK | Deamidated (NQ) |

| Osteomodulin OS=Bos taurus GN=OMD PE=1 SV=1 | 118 |
| --- | --- |

| 606.8166 | 1211.619 | 1211.619 | 62.64 | IFLGYNEISR | Deamidated (NQ) |
| --- | --- | --- | --- | --- | --- |
| 606.8166 | 1211.619 | 1211.619 | 56.7 | IFLGYNEISR | Deamidated (NQ) |
| 1286.306 | 3855.896 | 3855.882 | 66.01 | APISSYIFLCFPHIHTIYYGEQQSTNGQTIQLK | 2 Deamidated (NQ) |

| Thrombospondin-1 OS=Bos taurus GN=THBS1 PE=2 SV=2 | 110 |
| --- | --- |

| 495.309 | 988.6034 | 988.6069 | 60.02 | GFLLLASLR | |
| --- | --- | --- | --- | --- | --- |
| 495.3106 | 988.6066 | 988.6069 | 65.33 | GFLLLASLR | |
| 652.3586 | 1302.703 | 1302.703 | 52.66 | AGTLDLSLTVQGK | Deamidated (NQ) |

| Antithrombin-III OS=Bos taurus GN=SERPINC1 PE=1 SV=2 | 101 |
| --- | --- |

| 409.8835 | 1226.629 | 1226.63 | 52.44 | FRIEDSFSVK | |
| --- | --- | --- | --- | --- | --- |
| 670.8359 | 1339.657 | 1339.656 | 44.55 | TSDQIHFFFAK | |
| 690.3718 | 1378.729 | 1378.728 | 79.47 | EVALNTIIFMGR | Oxidation (M) |

| Collagen alpha-1(XI) chain (Fragment) OS=Bos taurus GN=COL11A1 PE=1 SV=1 | 95 |
| --- | --- |

| 615.8276 | 1229.641 | 1229.641 | 47.9 | LGVPGLPGYPGR | 3 Oxidation (P) |
| --- | --- | --- | --- | --- | --- |
| 615.8278 | 1229.641 | 1229.641 | 49.86 | LGVPGLPGYPGR | 3 Oxidation (P) |
| 901.803 | 2702.387 | 2702.385 | 46.09 | GPPGRPGLPGADGLPGPPGTMLVLPFR | Oxidation (M); 4 Oxidation (P) |

| Complement component C9 OS=Bos taurus GN=C9 PE=2 SV=1 | 93 |
| --- | --- |

| 635.3934 | 1268.772 | 1268.774 | 45.27 | LVPIYDLIPVK | |
| --- | --- | --- | --- | --- | --- |
| 728.852 | 1455.689 | 1455.688 | 72.99 | AIEDYINEFSVR | Deamidated (NQ) |

| Coagulation factor IX (Fragment) OS=Bos taurus GN=F9 PE=1 SV=1 | 93 |
| --- | --- |

| 511.7975 | 1021.58 | 1021.581 | 44.77 | SASILQYLK | |
| --- | --- | --- | --- | --- | --- |
| 512.2895 | 1022.564 | 1022.565 | 48.33 | SASILQYLK | Deamidated (NQ) |
| 610.7903 | 1219.566 | 1219.566 | 58.48 | FGYGYVSGWGK | |

| Vitamin D-binding protein OS=Bos taurus GN=GC PE=2 SV=1 | 93 |
| --- | --- |

| 666.8584 | 1331.702 | 1331.702 | 45.58 | HFSLLTIMTNR | |
| --- | --- | --- | --- | --- | --- |
| 691.8697 | 1381.725 | 1381.724 | 60.7 | VLDQYIFELSR | |
| 692.3616 | 1382.709 | 1382.708 | 50.95 | VLDQYIFELSR | Deamidated (NQ) |

| Coagulation factor X OS=Bos taurus GN=F10 PE=1 SV=1 | 76 |
| --- | --- |

| 447.2455 | 892.4764 | 892.4767 | 42.53 | TGIVSGFGR |
| --- | --- | --- | --- | --- |
| 731.6867 | 2192.038 | 2192.036 | 46.65 | FKDTYFVTGIVSWGEGCAR |

| Lysosomal alpha-mannosidase OS=Bos taurus GN=MAN2B1 PE=1 SV=3 | 56 |
| --- | --- |

| 542.8112 | 1083.608 | 1083.608 | 46.43 | WGPETLLLR |
| --- | --- | --- | --- | --- |
| 653.3696 | 1304.725 | 1304.724 | 47.28 | FQVIVYNPLGR |

**AuCPC**

| Collagen alpha-1(I) chain OS=Bos taurus GN=COL1A1 PE=1 SV=3 | 5238 |
| --- | --- |

| **pep_exp_mz** | **pep_exp_mr** | **pep_calc_mr** | **pep_score** | **pep_seq** | **pep_var_mod** |
| --- | --- | --- | --- | --- | --- |
| 392.2218 | 782.429 | 782.4286 | 45.88 | GAAGLPGPK | Oxidation (P) |
| 392.222 | 782.4294 | 782.4286 | 42.37 | GAAGLPGPK | Oxidation (P) |
| 418.7223 | 835.43 | 835.4301 | 44.36 | GPAGPQGPR | |
| 449.7584 | 897.5022 | 897.5032 | 44.19 | GVVGLPGQR | Oxidation (P) |
| 449.7589 | 897.5032 | 897.5032 | 42.89 | GVVGLPGQR | Oxidation (P) |
| 544.7721 | 1087.53 | 1087.53 | 72.18 | GFPGADGVAGPK | Oxidation (P) |
| 544.7722 | 1087.53 | 1087.53 | 72.17 | GFPGADGVAGPK | Oxidation (P) |
| 544.7725 | 1087.53 | 1087.53 | 53.78 | GFPGADGVAGPK | Oxidation (P) |
| 545.2941 | 1088.574 | 1088.573 | 45.42 | GVQGPPGPAGPR | |
| 553.291 | 1104.567 | 1104.568 | 42.16 | GVQGPPGPAGPR | Oxidation (P) |
| 553.2914 | 1104.568 | 1104.568 | 45.54 | GVQGPPGPAGPR | Oxidation (P) |
| 553.7831 | 1105.552 | 1105.552 | 49.4 | GVQGPPGPAGPR | Deamidated (NQ); Oxidation (P) |
| 564.2937 | 1126.573 | 1126.573 | 44.96 | GAAGLPGPKGDR | 2 Oxidation (P) |
| 565.7967 | 1129.579 | 1129.58 | 40.1 | GLPGTAGLPGMK | Oxidation (M); Oxidation (P) |
| 581.2895 | 1160.564 | 1160.565 | 63.06 | GQAGVMGFPGPK | Oxidation (P) |
| 581.2899 | 1160.565 | 1160.565 | 68.4 | GQAGVMGFPGPK | Oxidation (P) |
| 581.2903 | 1160.566 | 1160.565 | 55.87 | GQAGVMGFPGPK | Oxidation (M) |
| 581.7814 | 1161.548 | 1161.549 | 56.01 | GQAGVMGFPGPK | Deamidated (NQ); Oxidation (P) |
| 588.8223 | 1175.63 | 1175.63 | 55.54 | GVPGPPGAVGPAGK | Oxidation (P) |
| 589.2871 | 1176.56 | 1176.56 | 69.79 | GQAGVMGFPGPK | Oxidation (K); Oxidation (P) |
| 589.2872 | 1176.56 | 1176.56 | 53.22 | GQAGVMGFPGPK | Oxidation (M); Oxidation (P) |
| 589.2875 | 1176.56 | 1176.56 | 58.45 | GQAGVMGFPGPK | Oxidation (M); Oxidation (P) |
| 589.2877 | 1176.561 | 1176.56 | 44.96 | GQAGVMGFPGPK | Oxidation (K); Oxidation (P) |
| 589.7786 | 1177.543 | 1177.544 | 63.08 | GQAGVMGFPGPK | Deamidated (NQ); Oxidation (M); Oxidation (P) |
| 589.7786 | 1177.543 | 1177.544 | 56.46 | GQAGVMGFPGPK | Deamidated (NQ); Oxidation (K); Oxidation (P) |
| 589.7795 | 1177.544 | 1177.544 | 68.29 | GQAGVMGFPGPK | Deamidated (NQ); Oxidation (M); Oxidation (P) |
| 596.8193 | 1191.624 | 1191.625 | 44.54 | GVPGPPGAVGPAGK | 2 Oxidation (P) |
| 596.8195 | 1191.624 | 1191.625 | 54.68 | GVPGPPGAVGPAGK | 2 Oxidation (P) |
| 597.2848 | 1192.555 | 1192.555 | 55.34 | GQAGVMGFPGPK | Oxidation (K); Oxidation (M); Oxidation (P) |
| 597.7767 | 1193.539 | 1193.539 | 51.72 | GQAGVMGFPGPK | Deamidated (NQ); Oxidation (K); Oxidation (M); Oxidation (P) |
| 597.7768 | 1193.539 | 1193.539 | 60.84 | GQAGVMGFPGPK | Deamidated (NQ); Oxidation (K); Oxidation (M); Oxidation (P) |
| 621.8015 | 1241.588 | 1241.589 | 60.01 | GLTGSPGSPGPDGK | Oxidation (P) |
| 621.8018 | 1241.589 | 1241.589 | 58.07 | GLTGSPGSPGPDGK | Oxidation (P) |
| 629.799 | 1257.583 | 1257.584 | 45.69 | GLTGSPGSPGPDGK | 2 Oxidation (P) |
| 629.799 | 1257.583 | 1257.584 | 50.29 | GLTGSPGSPGPDGK | 2 Oxidation (P) |
| 629.799 | 1257.583 | 1257.584 | 43.33 | GLTGSPGSPGPDGK | 2 Oxidation (P) |
| 629.7992 | 1257.584 | 1257.584 | 41.84 | GLTGSPGSPGPDGK | 2 Oxidation (P) |
| 629.7992 | 1257.584 | 1257.584 | 41.21 | GLTGSPGSPGPDGK | 2 Oxidation (P) |
| 629.7999 | 1257.585 | 1257.584 | 51.97 | GLTGSPGSPGPDGK | 2 Oxidation (P) |
| 641.3128 | 1280.611 | 1280.611 | 69.66 | GEAGPSGPAGPTGAR | |
| 656.83 | 1311.645 | 1311.646 | 46.6 | GFPGLPGPSGEPGK | Oxidation (P) |
| 656.8304 | 1311.646 | 1311.646 | 55.52 | GFPGLPGPSGEPGK | Oxidation (P) |
| 664.8278 | 1327.641 | 1327.641 | 59.82 | GFPGLPGPSGEPGK | 2 Oxidation (P) |
| 664.8281 | 1327.642 | 1327.641 | 51.53 | GFPGLPGPSGEPGK | 2 Oxidation (P) |
| 666.8306 | 1331.647 | 1331.647 | 67.04 | GPSGPQGPSGPPGPK | Oxidation (P) |
| 666.8307 | 1331.647 | 1331.647 | 64.97 | GPSGPQGPSGPPGPK | Oxidation (P) |
| 666.8309 | 1331.647 | 1331.647 | 83.58 | GPSGPQGPSGPPGPK | Oxidation (P) |
| 666.8309 | 1331.647 | 1331.647 | 61.92 | GPSGPQGPSGPPGPK | Oxidation (P) |
| 666.8309 | 1331.647 | 1331.647 | 47.23 | GPSGPQGPSGPPGPK | Oxidation (P) |
| 666.831 | 1331.647 | 1331.647 | 51.63 | GPSGPQGPSGPPGPK | Oxidation (P) |
| 666.831 | 1331.647 | 1331.647 | 66.98 | GPSGPQGPSGPPGPK | Oxidation (P) |
| 666.8312 | 1331.648 | 1331.647 | 67.23 | GPSGPQGPSGPPGPK | Oxidation (P) |
| 666.8315 | 1331.648 | 1331.647 | 84.02 | GPSGPQGPSGPPGPK | Oxidation (P) |
| 667.3228 | 1332.631 | 1332.631 | 78.33 | GPSGPQGPSGPPGPK | Deamidated (NQ); Oxidation (P) |
| 667.3231 | 1332.632 | 1332.631 | 47.97 | GPSGPQGPSGPPGPK | Deamidated (NQ); Oxidation (P) |
| 672.8253 | 1343.636 | 1343.636 | 53.27 | GFPGLPGPSGEPGK | 3 Oxidation (P) |
| 672.8254 | 1343.636 | 1343.636 | 43.88 | GFPGLPGPSGEPGK | 3 Oxidation (P) |
| 672.8259 | 1343.637 | 1343.636 | 53.17 | GFPGLPGPSGEPGK | 3 Oxidation (P) |
| 674.8286 | 1347.643 | 1347.642 | 72.21 | GPSGPQGPSGPPGPK | 2 Oxidation (P) |
| 718.3438 | 1434.673 | 1434.674 | 63.37 | GEPGPAGLPGPPGER | 3 Oxidation (P) |
| 718.3442 | 1434.674 | 1434.674 | 47.43 | GEPGPAGLPGPPGER | 3 Oxidation (P) |
| 718.345 | 1434.675 | 1434.674 | 42.84 | GEPGPAGLPGPPGER | 3 Oxidation (P) |
| 728.4114 | 1454.808 | 1454.809 | 56.49 | ALLLQGSNEIEIR | |
| 730.349 | 1458.683 | 1458.685 | 48.42 | GSAGPPGATGFPGAAGR | 2 Oxidation (P) |
| 730.3492 | 1458.684 | 1458.685 | 107.7 | GSAGPPGATGFPGAAGR | 2 Oxidation (P) |
| 730.3493 | 1458.684 | 1458.685 | 48.14 | GSAGPPGATGFPGAAGR | 2 Oxidation (P) |
| 730.3494 | 1458.684 | 1458.685 | 69.47 | GSAGPPGATGFPGAAGR | 2 Oxidation (P) |
| 730.3495 | 1458.684 | 1458.685 | 47.75 | GSAGPPGATGFPGAAGR | 2 Oxidation (P) |
| 730.3497 | 1458.685 | 1458.685 | 43.97 | GSAGPPGATGFPGAAGR | 2 Oxidation (P) |
| 730.3497 | 1458.685 | 1458.685 | 48.06 | GSAGPPGATGFPGAAGR | 2 Oxidation (P) |
| 730.35 | 1458.685 | 1458.685 | 91.23 | GSAGPPGATGFPGAAGR | 2 Oxidation (P) |
| 730.35 | 1458.685 | 1458.685 | 76.15 | GSAGPPGATGFPGAAGR | 2 Oxidation (P) |
| 730.35 | 1458.685 | 1458.685 | 44 | GSAGPPGATGFPGAAGR | 2 Oxidation (P) |
| 730.3501 | 1458.686 | 1458.685 | 40.05 | GSAGPPGATGFPGAAGR | 2 Oxidation (P) |
| 730.3502 | 1458.686 | 1458.685 | 84.44 | GSAGPPGATGFPGAAGR | 2 Oxidation (P) |
| 730.3502 | 1458.686 | 1458.685 | 56.55 | GSAGPPGATGFPGAAGR | 2 Oxidation (P) |
| 730.3503 | 1458.686 | 1458.685 | 74.81 | GSAGPPGATGFPGAAGR | 2 Oxidation (P) |
| 730.3503 | 1458.686 | 1458.685 | 46.79 | GSAGPPGATGFPGAAGR | 2 Oxidation (P) |
| 730.3504 | 1458.686 | 1458.685 | 115.42 | GSAGPPGATGFPGAAGR | 2 Oxidation (P) |
| 730.3505 | 1458.686 | 1458.685 | 109.02 | GSAGPPGATGFPGAAGR | 2 Oxidation (P) |
| 730.3506 | 1458.687 | 1458.685 | 86.22 | GSAGPPGATGFPGAAGR | 2 Oxidation (P) |
| 730.3506 | 1458.687 | 1458.685 | 48.94 | GSAGPPGATGFPGAAGR | 2 Oxidation (P) |
| 520.9424 | 1559.805 | 1559.806 | 63.96 | GETGPAGPAGPIGPVGAR | |
| 521.2701 | 1560.789 | 1560.79 | 51.49 | DGLNGLPGPIGPPGPR | 3 Oxidation (P) |
| 781.402 | 1560.789 | 1560.79 | 44.51 | DGLNGLPGPIGPPGPR | 3 Oxidation (P) |
| 781.8934 | 1561.772 | 1561.774 | 48.93 | DGLNGLPGPIGPPGPR | Deamidated (NQ); 3 Oxidation (P) |
| 781.8947 | 1561.775 | 1561.774 | 56.41 | DGLNGLPGPIGPPGPR | Deamidated (NQ); 3 Oxidation (P) |
| 793.389 | 1584.763 | 1584.765 | 64.11 | GANGAPGIAGAPGFPGAR | 3 Oxidation (P) |
| 793.8807 | 1585.747 | 1585.749 | 48.73 | GANGAPGIAGAPGFPGAR | Deamidated (NQ); 3 Oxidation (P) |
| 793.8821 | 1585.75 | 1585.749 | 64.22 | GANGAPGIAGAPGFPGAR | Deamidated (NQ); 3 Oxidation (P) |
| 801.3995 | 1600.784 | 1600.785 | 63.5 | GFSGLDGAKGDAGPAGPK | |
| 535.9491 | 1604.826 | 1604.827 | 45.53 | AGERGVPGPPGAVGPAGK | 2 Oxidation (P) |
| 808.3953 | 1614.776 | 1614.775 | 51.85 | GGPGSRGFPGADGVAGPK | 2 Oxidation (P) |
| 828.403 | 1654.791 | 1654.791 | 62.98 | GSPGEAGRPGEAGLPGAK | 3 Oxidation (P) |
| 845.8931 | 1689.772 | 1689.771 | 42.58 | DGEAGAQGPPGPAGPAGER | |
| 846.385 | 1690.755 | 1690.755 | 40.9 | DGEAGAQGPPGPAGPAGER | Deamidated (NQ) |
| 846.3854 | 1690.756 | 1690.755 | 100.76 | DGEAGAQGPPGPAGPAGER | Deamidated (NQ) |
| 853.8905 | 1705.766 | 1705.766 | 84.77 | DGEAGAQGPPGPAGPAGER | Oxidation (P) |
| 871.8743 | 1741.734 | 1741.733 | 76.19 | GEPGSPGENGAPGQMGPR | 3 Oxidation (P) |
| 872.3657 | 1742.717 | 1742.717 | 89.79 | GEPGSPGENGAPGQMGPR | Deamidated (NQ); 3 Oxidation (P) |
| 581.9556 | 1742.845 | 1742.845 | 70.15 | GARGSAGPPGATGFPGAAGR | 2 Oxidation (P) |
| 872.8581 | 1743.702 | 1743.701 | 92.01 | GEPGSPGENGAPGQMGPR | 2 Deamidated (NQ); 3 Oxidation (P) |
| 872.8583 | 1743.702 | 1743.701 | 67.98 | GEPGSPGENGAPGQMGPR | 2 Deamidated (NQ); 3 Oxidation (P) |
| 900.9395 | 1799.864 | 1799.863 | 47.65 | GPPGPMGPPGLAGPPGESGR | Oxidation (M) |
| 906.9473 | 1811.88 | 1811.88 | 40.53 | VGPPGPSGNAGPPGPPGPAGK | 3 Oxidation (P) |
| 906.9482 | 1811.882 | 1811.88 | 41.19 | VGPPGPSGNAGPPGPPGPAGK | Oxidation (K); 2 Oxidation (P) |
| 907.4398 | 1812.865 | 1812.864 | 48.39 | VGPPGPSGNAGPPGPPGPAGK | Deamidated (NQ); Oxidation (K); 2 Oxidation (P) |
| 908.9364 | 1815.858 | 1815.857 | 86.42 | GPPGPMGPPGLAGPPGESGR | 2 Oxidation (P) |
| 914.9457 | 1827.877 | 1827.875 | 45.01 | VGPPGPSGNAGPPGPPGPAGK | 4 Oxidation (P) |
| 916.9325 | 1831.85 | 1831.852 | 57.79 | GPPGPMGPPGLAGPPGESGR | Oxidation (M); 2 Oxidation (P) |
| 916.9337 | 1831.853 | 1831.852 | 72.64 | GPPGPMGPPGLAGPPGESGR | Oxidation (M); 2 Oxidation (P) |
| 916.934 | 1831.853 | 1831.852 | 94.67 | GPPGPMGPPGLAGPPGESGR | Oxidation (M); 2 Oxidation (P) |
| 932.44 | 1862.865 | 1862.865 | 69.68 | GEPGPTGIQGPPGPAGEEGK | 2 Oxidation (P) |
| 988.5003 | 1974.986 | 1974.987 | 61.54 | SGDRGETGPAGPAGPIGPVGAR | |
| 988.5008 | 1974.987 | 1974.987 | 54.4 | SGDRGETGPAGPAGPIGPVGAR | |
| 659.3365 | 1974.988 | 1974.987 | 44.81 | SGDRGETGPAGPAGPIGPVGAR | |
| 988.5017 | 1974.989 | 1974.987 | 71.4 | SGDRGETGPAGPAGPIGPVGAR | |
| 659.337 | 1974.989 | 1974.987 | 56.57 | SGDRGETGPAGPAGPIGPVGAR | |
| 673.9957 | 2018.965 | 2018.966 | 46.96 | GEPGPTGIQGPPGPAGEEGKR | 2 Oxidation (P) |
| 1010.491 | 2018.967 | 2018.966 | 88.45 | GEPGPTGIQGPPGPAGEEGKR | 2 Oxidation (P) |
| 686.3312 | 2055.972 | 2055.972 | 40.17 | TGPPGPAGQDGRPGPPGPPGAR | 4 Oxidation (P) |
| 1029.012 | 2056.009 | 2056.009 | 83.37 | GAPGADGPAGAPGTPGPQGIAGQR | |
| 686.66 | 2056.958 | 2056.956 | 41.14 | TGPPGPAGQDGRPGPPGPPGAR | Deamidated (NQ); 4 Oxidation (P) |
| 691.6755 | 2072.005 | 2072.004 | 40.26 | GAPGADGPAGAPGTPGPQGIAGQR | Oxidation (P) |
| 1037.01 | 2072.005 | 2072.004 | 92.09 | GAPGADGPAGAPGTPGPQGIAGQR | Oxidation (P) |
| 1037.01 | 2072.005 | 2072.004 | 84.03 | GAPGADGPAGAPGTPGPQGIAGQR | Oxidation (P) |
| 1037.501 | 2072.987 | 2072.988 | 74.94 | GAPGADGPAGAPGTPGPQGIAGQR | Deamidated (NQ); Oxidation (P) |
| 1037.502 | 2072.989 | 2072.988 | 75.87 | GAPGADGPAGAPGTPGPQGIAGQR | Deamidated (NQ); Oxidation (P) |
| 697.007 | 2087.999 | 2087.999 | 45.54 | GAPGADGPAGAPGTPGPQGIAGQR | 2 Oxidation (P) |
| 1045.499 | 2088.983 | 2088.983 | 43.23 | GAPGADGPAGAPGTPGPQGIAGQR | Deamidated (NQ); 2 Oxidation (P) |
| 1053.004 | 2103.993 | 2103.993 | 74.85 | GAPGADGPAGAPGTPGPQGIAGQR | 3 Oxidation (P) |
| 1067.496 | 2132.977 | 2132.976 | 40.73 | GEPGPPGPAGFAGPPGADGQPGAK | 3 Oxidation (P) |
| 1067.987 | 2133.959 | 2133.96 | 43.77 | GEPGPPGPAGFAGPPGADGQPGAK | Deamidated (NQ); 3 Oxidation (P) |
| 712.3281 | 2133.963 | 2133.96 | 46.67 | GEPGPPGPAGFAGPPGADGQPGAK | Deamidated (NQ); Oxidation (K); 2 Oxidation (P) |
| 1077.035 | 2152.055 | 2152.055 | 51.91 | GETGPAGPPGAPGAPGAPGPVGPAGK | 2 Oxidation (P) |
| 1085.032 | 2168.049 | 2168.05 | 60.25 | GETGPAGPPGAPGAPGAPGPVGPAGK | 3 Oxidation (P) |
| 1085.033 | 2168.051 | 2168.05 | 56.82 | GETGPAGPPGAPGAPGAPGPVGPAGK | 3 Oxidation (P) |
| 1100.482 | 2198.949 | 2198.95 | 69.59 | GDAGAPGAPGSQGAPGLQGMPGER | Deamidated (NQ); 4 Oxidation (P) |
| 1100.482 | 2198.949 | 2198.95 | 66.38 | GDAGAPGAPGSQGAPGLQGMPGER | Deamidated (NQ); 4 Oxidation (P) |
| 1108.479 | 2214.943 | 2214.945 | 45.21 | GDAGAPGAPGSQGAPGLQGMPGER | Deamidated (NQ); Oxidation (M); 4 Oxidation (P) |
| 1108.971 | 2215.927 | 2215.929 | 69.43 | GDAGAPGAPGSQGAPGLQGMPGER | 2 Deamidated (NQ); Oxidation (M); 4 Oxidation (P) |
| 1146.075 | 2290.135 | 2290.134 | 99.4 | GDAGPPGPAGPAGPPGPIGNVGAPGPK | Oxidation (K); Oxidation (P) |
| 764.7126 | 2291.116 | 2291.118 | 42.3 | GDAGPPGPAGPAGPPGPIGNVGAPGPK | Deamidated (NQ); Oxidation (K); Oxidation (P) |
| 1146.567 | 2291.119 | 2291.118 | 91.07 | GDAGPPGPAGPAGPPGPIGNVGAPGPK | Deamidated (NQ); Oxidation (K); Oxidation (P) |
| 767.3558 | 2299.046 | 2299.047 | 40.13 | GEPGPPGPAGAAGPAGNPGADGQPGAK | Oxidation (K); 2 Oxidation (P) |
| 767.6842 | 2300.031 | 2300.031 | 50.61 | GEPGPPGPAGAAGPAGNPGADGQPGAK | Deamidated (NQ); Oxidation (K); 2 Oxidation (P) |
| 772.6885 | 2315.044 | 2315.042 | 54.36 | GEPGPPGPAGAAGPAGNPGADGQPGAK | 4 Oxidation (P) |
| 1159.021 | 2316.027 | 2316.026 | 60.2 | GEPGPPGPAGAAGPAGNPGADGQPGAK | Deamidated (NQ); 4 Oxidation (P) |
| 773.0165 | 2316.028 | 2316.026 | 63 | GEPGPPGPAGAAGPAGNPGADGQPGAK | Deamidated (NQ); 4 Oxidation (P) |
| 773.3436 | 2317.009 | 2317.01 | 48 | GEPGPPGPAGAAGPAGNPGADGQPGAK | 2 Deamidated (NQ); Oxidation (K); 3 Oxidation (P) |
| 1159.512 | 2317.009 | 2317.01 | 71.28 | GEPGPPGPAGAAGPAGNPGADGQPGAK | 2 Deamidated (NQ); 4 Oxidation (P) |
| 1162.068 | 2322.121 | 2322.124 | 64.52 | GDAGPPGPAGPAGPPGPIGNVGAPGPK | 4 Oxidation (P) |
| 1162.068 | 2322.121 | 2322.124 | 65.99 | GDAGPPGPAGPAGPPGPIGNVGAPGPK | 4 Oxidation (P) |
| 778.0192 | 2331.036 | 2331.036 | 43.14 | GEPGPPGPAGAAGPAGNPGADGQPGAK | 5 Oxidation (P) |
| 778.3478 | 2332.022 | 2332.02 | 45.58 | GEPGPPGPAGAAGPAGNPGADGQPGAK | Deamidated (NQ); 5 Oxidation (P) |
| 829.7416 | 2486.203 | 2486.204 | 49.87 | GPPGSAGSPGKDGLNGLPGPIGPPGPR | Deamidated (NQ); 5 Oxidation (P) |
| 1244.11 | 2486.205 | 2486.204 | 42.91 | GPPGSAGSPGKDGLNGLPGPIGPPGPR | Deamidated (NQ); 5 Oxidation (P) |
| 1244.11 | 2486.205 | 2486.204 | 45.08 | GPPGSAGSPGKDGLNGLPGPIGPPGPR | Deamidated (NQ); 5 Oxidation (P) |
| 832.0353 | 2493.084 | 2493.083 | 54.29 | GDAGPAGPKGEPGSPGENGAPGQMGPR | Deamidated (NQ); 3 Oxidation (P) |
| 1274.608 | 2547.201 | 2547.199 | 65.79 | GNDGATGAAGPPGPTGPAGPPGFPGAVGAK | 3 Oxidation (P) |
| 1274.608 | 2547.201 | 2547.199 | 70.54 | GNDGATGAAGPPGPTGPAGPPGFPGAVGAK | 3 Oxidation (P) |
| 897.4188 | 2689.235 | 2689.237 | 62.78 | GFSGLQGPPGPPGSPGEQGPSGASGPAGPR | Deamidated (NQ); 2 Oxidation (P) |
| 897.4206 | 2689.24 | 2689.237 | 58.44 | GFSGLQGPPGPPGSPGEQGPSGASGPAGPR | Deamidated (NQ); 2 Oxidation (P) |
| 901.7513 | 2702.232 | 2702.232 | 40.84 | GAPGDRGEPGPPGPAGFAGPPGADGQPGAK | 4 Oxidation (P) |
| 902.4232 | 2704.248 | 2704.248 | 55.76 | GFSGLQGPPGPPGSPGEQGPSGASGPAGPR | 3 Oxidation (P) |
| 902.4236 | 2704.249 | 2704.248 | 60.06 | GFSGLQGPPGPPGSPGEQGPSGASGPAGPR | 3 Oxidation (P) |
| 1353.622 | 2705.229 | 2705.232 | 60 | GFSGLQGPPGPPGSPGEQGPSGASGPAGPR | Deamidated (NQ); 3 Oxidation (P) |
| 902.7511 | 2705.232 | 2705.232 | 89.37 | GFSGLQGPPGPPGSPGEQGPSGASGPAGPR | Deamidated (NQ); 3 Oxidation (P) |
| 902.7516 | 2705.233 | 2705.232 | 48.52 | GFSGLQGPPGPPGSPGEQGPSGASGPAGPR | Deamidated (NQ); 3 Oxidation (P) |
| 907.0834 | 2718.228 | 2718.227 | 42.57 | GAPGDRGEPGPPGPAGFAGPPGADGQPGAK | 5 Oxidation (P) |
| 946.478 | 2836.412 | 2836.41 | 42.3 | GLTGPIGPPGPAGAPGDKGEAGPSGPAGPTGAR | Oxidation (P) |
| 951.8096 | 2852.407 | 2852.405 | 52.54 | GLTGPIGPPGPAGAPGDKGEAGPSGPAGPTGAR | 2 Oxidation (P) |
| 951.8101 | 2852.409 | 2852.405 | 45.62 | GLTGPIGPPGPAGAPGDKGEAGPSGPAGPTGAR | 2 Oxidation (P) |
| 1081.139 | 3240.395 | 3240.397 | 54.44 | GANGAPGNDGAKGDAGAPGAPGSQGAPGLQGMPGER | Deamidated (NQ); Oxidation (K); 5 Oxidation (P) |

| Collagen alpha-2(I) chain OS=Bos taurus GN=COL1A2 PE=1 SV=2 | 5001 |
| --- | --- |

| 405.222 | 808.4294 | 808.4304 | 46.97 | GHAGLAGAR | |
| --- | --- | --- | --- | --- | --- |
| 405.2224 | 808.4302 | 808.4304 | 46.64 | GHAGLAGAR | |
| 434.7354 | 867.4562 | 867.4563 | 51.12 | GPSGPQGIR | |
| 435.2272 | 868.4398 | 868.4403 | 46.99 | GPSGPQGIR | Deamidated (NQ) |
| 446.7534 | 891.4922 | 891.4926 | 49.53 | PGPIGPAGAR | |
| 446.7536 | 891.4926 | 891.4926 | 41.9 | PGPIGPAGAR | |
| 451.7292 | 901.4438 | 901.444 | 44.74 | AGVMGPAGSR | |
| 451.7292 | 901.4438 | 901.444 | 41.35 | AGVMGPAGSR | |
| 454.751 | 907.4874 | 907.4876 | 44.04 | PGPIGPAGAR | Oxidation (P) |
| 459.7268 | 917.439 | 917.4389 | 49.37 | AGVMGPAGSR | Oxidation (M) |
| 524.2861 | 1046.558 | 1046.558 | 43.9 | LNIGPVCFK | |
| 529.7501 | 1057.486 | 1057.486 | 43.82 | PGEPGLMGPR | Oxidation (M); 2 Oxidation (P) |
| 542.7852 | 1083.556 | 1083.556 | 41.56 | GLVGEPGPAGSK | Oxidation (P) |
| 591.809 | 1181.603 | 1181.604 | 47.52 | EGPVGLPGIDGR | Oxidation (P) |
| 591.8093 | 1181.604 | 1181.604 | 55.38 | EGPVGLPGIDGR | Oxidation (P) |
| 592.2894 | 1182.564 | 1182.564 | 63.5 | GGGPGPMGLMGPR | |
| 398.2312 | 1191.672 | 1191.672 | 48.4 | IGQPGAVGPAGIR | |
| 596.8434 | 1191.672 | 1191.672 | 52.24 | IGQPGAVGPAGIR | |
| 596.8434 | 1191.672 | 1191.672 | 45.73 | IGQPGAVGPAGIR | |
| 596.8435 | 1191.672 | 1191.672 | 66.9 | IGQPGAVGPAGIR | |
| 596.844 | 1191.673 | 1191.672 | 55.85 | IGQPGAVGPAGIR | |
| 597.3356 | 1192.657 | 1192.656 | 51.63 | IGQPGAVGPAGIR | Deamidated (NQ) |
| 600.2872 | 1198.56 | 1198.559 | 60.99 | GGGPGPMGLMGPR | Oxidation (P) |
| 601.2955 | 1200.576 | 1200.578 | 58.04 | GEPGNIGFPGPK | 2 Oxidation (P) |
| 601.2959 | 1200.577 | 1200.578 | 65.47 | GEPGNIGFPGPK | 2 Oxidation (P) |
| 601.7885 | 1201.562 | 1201.562 | 52.45 | GEPGNIGFPGPK | Deamidated (NQ); 2 Oxidation (P) |
| 604.8403 | 1207.666 | 1207.667 | 56.23 | IGQPGAVGPAGIR | Oxidation (P) |
| 604.8408 | 1207.667 | 1207.667 | 59.93 | IGQPGAVGPAGIR | Oxidation (P) |
| 604.8408 | 1207.667 | 1207.667 | 43.38 | IGQPGAVGPAGIR | Oxidation (P) |
| 604.8408 | 1207.667 | 1207.667 | 51.77 | IGQPGAVGPAGIR | Oxidation (P) |
| 604.8409 | 1207.667 | 1207.667 | 62.92 | IGQPGAVGPAGIR | Oxidation (P) |
| 604.841 | 1207.667 | 1207.667 | 63.8 | IGQPGAVGPAGIR | Oxidation (P) |
| 604.8411 | 1207.668 | 1207.667 | 41.41 | IGQPGAVGPAGIR | Oxidation (P) |
| 604.8411 | 1207.668 | 1207.667 | 47.43 | IGQPGAVGPAGIR | Oxidation (P) |
| 605.3329 | 1208.651 | 1208.651 | 55.11 | IGQPGAVGPAGIR | Deamidated (NQ); Oxidation (P) |
| 605.3335 | 1208.652 | 1208.651 | 75.17 | IGQPGAVGPAGIR | Deamidated (NQ); Oxidation (P) |
| 608.2841 | 1214.554 | 1214.554 | 41.22 | GGGPGPMGLMGPR | 2 Oxidation (M) |
| 611.8086 | 1221.603 | 1221.603 | 43.34 | GFPGTPGLPGFK | 3 Oxidation (P) |
| 619.8062 | 1237.598 | 1237.598 | 40.14 | GFPGTPGLPGFK | Oxidation (K); 3 Oxidation (P) |
| 631.3181 | 1260.622 | 1260.621 | 52.44 | GEAGPAGPAGPAGPR | |
| 631.3181 | 1260.622 | 1260.621 | 41.08 | GEAGPAGPAGPAGPR | |
| 634.3408 | 1266.667 | 1266.668 | 89.15 | GIPGPVGAAGATGAR | Oxidation (P) |
| 634.3408 | 1266.667 | 1266.668 | 54.5 | GIPGPVGAAGATGAR | Oxidation (P) |
| 634.3409 | 1266.667 | 1266.668 | 78.56 | GIPGPVGAAGATGAR | Oxidation (P) |
| 634.3409 | 1266.667 | 1266.668 | 48.98 | GIPGPVGAAGATGAR | Oxidation (P) |
| 634.3417 | 1266.669 | 1266.668 | 62.51 | GIPGPVGAAGATGAR | Oxidation (P) |
| 644.3195 | 1286.624 | 1286.626 | 85.01 | GFPGSPGNIGPAGK | 2 Oxidation (P) |
| 644.3205 | 1286.626 | 1286.626 | 44.21 | GFPGSPGNIGPAGK | 2 Oxidation (P) |
| 644.8115 | 1287.608 | 1287.61 | 74 | GFPGSPGNIGPAGK | Deamidated (NQ); 2 Oxidation (P) |
| 714.3668 | 1426.719 | 1426.721 | 58.86 | GIPGEFGLPGPAGAR | 2 Oxidation (P) |
| 714.3677 | 1426.721 | 1426.721 | 75.49 | GIPGEFGLPGPAGAR | 2 Oxidation (P) |
| 714.3679 | 1426.721 | 1426.721 | 57.84 | GIPGEFGLPGPAGAR | 2 Oxidation (P) |
| 714.3681 | 1426.722 | 1426.721 | 47.29 | GIPGEFGLPGPAGAR | 2 Oxidation (P) |
| 714.3681 | 1426.722 | 1426.721 | 46.91 | GIPGEFGLPGPAGAR | 2 Oxidation (P) |
| 714.3682 | 1426.722 | 1426.721 | 49.52 | GIPGEFGLPGPAGAR | 2 Oxidation (P) |
| 722.3645 | 1442.714 | 1442.715 | 41.79 | GIPGEFGLPGPAGAR | 3 Oxidation (P) |
| 729.3419 | 1456.669 | 1456.67 | 59.18 | GDGGPPGATGFPGAAGR | Oxidation (P) |
| 729.342 | 1456.669 | 1456.67 | 103.88 | GDGGPPGATGFPGAAGR | Oxidation (P) |
| 729.3423 | 1456.67 | 1456.67 | 81.16 | GDGGPPGATGFPGAAGR | Oxidation (P) |
| 737.3388 | 1472.663 | 1472.665 | 41.98 | GDGGPPGATGFPGAAGR | 2 Oxidation (P) |
| 737.3391 | 1472.664 | 1472.665 | 83.82 | GDGGPPGATGFPGAAGR | 2 Oxidation (P) |
| 737.3391 | 1472.664 | 1472.665 | 54.12 | GDGGPPGATGFPGAAGR | 2 Oxidation (P) |
| 737.3391 | 1472.664 | 1472.665 | 58.8 | GDGGPPGATGFPGAAGR | 2 Oxidation (P) |
| 737.3397 | 1472.665 | 1472.665 | 56.06 | GDGGPPGATGFPGAAGR | 2 Oxidation (P) |
| 737.3398 | 1472.665 | 1472.665 | 59.12 | GDGGPPGATGFPGAAGR | 2 Oxidation (P) |
| 737.3398 | 1472.665 | 1472.665 | 59.64 | GDGGPPGATGFPGAAGR | 2 Oxidation (P) |
| 746.8495 | 1491.684 | 1491.684 | 79.44 | SGETGASGPPGFVGEK | Oxidation (P) |
| 746.8495 | 1491.684 | 1491.684 | 42.7 | SGETGASGPPGFVGEK | Oxidation (P) |
| 754.8468 | 1507.679 | 1507.679 | 45.83 | SGETGASGPPGFVGEK | Oxidation (K); Oxidation (P) |
| 511.5983 | 1531.773 | 1531.774 | 49.42 | GEPGPAGAVGPAGAVGPR | Oxidation (P) |
| 766.8947 | 1531.775 | 1531.774 | 99.61 | GEPGPAGAVGPAGAVGPR | Oxidation (P) |
| 766.8947 | 1531.775 | 1531.774 | 58.25 | GEPGPAGAVGPAGAVGPR | Oxidation (P) |
| 769.3715 | 1536.728 | 1536.728 | 42.2 | GAPGAIGAPGPAGANGDR | 2 Oxidation (P) |
| 769.8633 | 1537.712 | 1537.712 | 61.59 | GAPGAIGAPGPAGANGDR | Deamidated (NQ); 2 Oxidation (P) |
| 521.6141 | 1561.821 | 1561.821 | 53.47 | GAAGLPGVAGAPGLPGPR | 3 Oxidation (P) |
| 521.6144 | 1561.821 | 1561.821 | 42.95 | GAAGLPGVAGAPGLPGPR | 3 Oxidation (P) |
| 781.9184 | 1561.822 | 1561.821 | 69.64 | GAAGLPGVAGAPGLPGPR | 3 Oxidation (P) |
| 790.8864 | 1579.758 | 1579.759 | 81.14 | GPPGESGAAGPTGPIGSR | Oxidation (P) |
| 527.5935 | 1579.759 | 1579.759 | 41.22 | GPPGESGAAGPTGPIGSR | Oxidation (P) |
| 790.8867 | 1579.759 | 1579.759 | 68.91 | GPPGESGAAGPTGPIGSR | Oxidation (P) |
| 790.8868 | 1579.759 | 1579.759 | 83.63 | GPPGESGAAGPTGPIGSR | Oxidation (P) |
| 790.887 | 1579.759 | 1579.759 | 61.34 | GPPGESGAAGPTGPIGSR | Oxidation (P) |
| 790.887 | 1579.759 | 1579.759 | 70.55 | GPPGESGAAGPTGPIGSR | Oxidation (P) |
| 790.8871 | 1579.76 | 1579.759 | 85.76 | GPPGESGAAGPTGPIGSR | Oxidation (P) |
| 790.8874 | 1579.76 | 1579.759 | 70.92 | GPPGESGAAGPTGPIGSR | Oxidation (P) |
| 790.8875 | 1579.76 | 1579.759 | 73.12 | GPPGESGAAGPTGPIGSR | Oxidation (P) |
| 800.4156 | 1598.817 | 1598.817 | 76.86 | GELGPVGNPGPAGPAGPR | |
| 533.9466 | 1598.818 | 1598.817 | 56.16 | GELGPVGNPGPAGPAGPR | |
| 808.4124 | 1614.81 | 1614.811 | 69.62 | GELGPVGNPGPAGPAGPR | Oxidation (P) |
| 539.2775 | 1614.811 | 1614.811 | 68.33 | GELGPVGNPGPAGPAGPR | Oxidation (P) |
| 816.921 | 1631.827 | 1631.827 | 45.52 | GSTGEIGPAGPPGPPGLR | Oxidation (P) |
| 824.9175 | 1647.82 | 1647.822 | 56.85 | GSTGEIGPAGPPGPPGLR | 2 Oxidation (P) |
| 550.2809 | 1647.821 | 1647.822 | 49.45 | GSTGEIGPAGPPGPPGLR | 2 Oxidation (P) |
| 824.918 | 1647.821 | 1647.822 | 59.24 | GSTGEIGPAGPPGPPGLR | 2 Oxidation (P) |
| 824.9189 | 1647.823 | 1647.822 | 40.22 | GSTGEIGPAGPPGPPGLR | 2 Oxidation (P) |
| 602.3152 | 1803.924 | 1803.923 | 44.24 | RGSTGEIGPAGPPGPPGLR | 2 Oxidation (P) |
| 911.9194 | 1821.824 | 1821.824 | 91.47 | GPPGNVGNPGVNGAPGEAGR | Deamidated (NQ); 3 Oxidation (P) |
| 911.9202 | 1821.826 | 1821.824 | 67.61 | GPPGNVGNPGVNGAPGEAGR | Deamidated (NQ); 3 Oxidation (P) |
| 912.4111 | 1822.808 | 1822.808 | 64.09 | GPPGNVGNPGVNGAPGEAGR | 2 Deamidated (NQ); 3 Oxidation (P) |
| 912.9038 | 1823.793 | 1823.792 | 75.38 | GPPGNVGNPGVNGAPGEAGR | 3 Deamidated (NQ); 3 Oxidation (P) |
| 915.4544 | 1828.894 | 1828.896 | 49.68 | TGPPGPSGISGPPGPPGPAGK | Oxidation (K); 2 Oxidation (P) |
| 931.4509 | 1860.887 | 1860.885 | 40.56 | TGPPGPSGISGPPGPPGPAGK | 5 Oxidation (P) |
| 666.3406 | 1996 | 1995.999 | 46.76 | HGNRGEPGPAGAVGPAGAVGPR | Oxidation (P) |
| 666.3409 | 1996.001 | 1995.999 | 53.1 | HGNRGEPGPAGAVGPAGAVGPR | Oxidation (P) |
| 999.4989 | 1996.983 | 1996.983 | 42.26 | HGNRGEPGPAGAVGPAGAVGPR | Deamidated (NQ); Oxidation (P) |
| 1026.493 | 2050.971 | 2050.971 | 65.54 | GEVGPAGPNGFAGPAGAAGQPGAK | Deamidated (NQ); Oxidation (K) |
| 1026.984 | 2051.953 | 2051.955 | 95.99 | GEVGPAGPNGFAGPAGAAGQPGAK | 2 Deamidated (NQ); Oxidation (P) |
| 1028.551 | 2055.087 | 2055.086 | 54.86 | EGPVGLPGIDGRPGPIGPAGAR | Oxidation (P) |
| 1036.547 | 2071.079 | 2071.081 | 40.88 | EGPVGLPGIDGRPGPIGPAGAR | 2 Oxidation (P) |
| 1036.549 | 2071.083 | 2071.081 | 43.34 | EGPVGLPGIDGRPGPIGPAGAR | 2 Oxidation (P) |
| 711.0421 | 2130.105 | 2130.107 | 53.66 | GLPGVAGSVGEPGPLGIAGPPGAR | 3 Oxidation (P) |
| 711.0424 | 2130.105 | 2130.107 | 44.14 | GLPGVAGSVGEPGPLGIAGPPGAR | 3 Oxidation (P) |
| 1066.06 | 2130.105 | 2130.107 | 91.66 | GLPGVAGSVGEPGPLGIAGPPGAR | 3 Oxidation (P) |
| 1066.06 | 2130.105 | 2130.107 | 83.33 | GLPGVAGSVGEPGPLGIAGPPGAR | 3 Oxidation (P) |
| 1066.06 | 2130.105 | 2130.107 | 84.16 | GLPGVAGSVGEPGPLGIAGPPGAR | 3 Oxidation (P) |
| 1066.06 | 2130.105 | 2130.107 | 103.96 | GLPGVAGSVGEPGPLGIAGPPGAR | 3 Oxidation (P) |
| 1066.06 | 2130.105 | 2130.107 | 80.36 | GLPGVAGSVGEPGPLGIAGPPGAR | 3 Oxidation (P) |
| 711.0429 | 2130.107 | 2130.107 | 45.46 | GLPGVAGSVGEPGPLGIAGPPGAR | 3 Oxidation (P) |
| 1066.061 | 2130.107 | 2130.107 | 94.51 | GLPGVAGSVGEPGPLGIAGPPGAR | 3 Oxidation (P) |
| 1066.061 | 2130.107 | 2130.107 | 82 | GLPGVAGSVGEPGPLGIAGPPGAR | 3 Oxidation (P) |
| 1066.061 | 2130.107 | 2130.107 | 77.78 | GLPGVAGSVGEPGPLGIAGPPGAR | 3 Oxidation (P) |
| 711.0433 | 2130.108 | 2130.107 | 43.5 | GLPGVAGSVGEPGPLGIAGPPGAR | 3 Oxidation (P) |
| 1066.062 | 2130.109 | 2130.107 | 83.34 | GLPGVAGSVGEPGPLGIAGPPGAR | 3 Oxidation (P) |
| 1066.062 | 2130.109 | 2130.107 | 99.52 | GLPGVAGSVGEPGPLGIAGPPGAR | 3 Oxidation (P) |
| 1066.062 | 2130.109 | 2130.107 | 97.48 | GLPGVAGSVGEPGPLGIAGPPGAR | 3 Oxidation (P) |
| 1074.057 | 2146.099 | 2146.102 | 50.73 | GLPGVAGSVGEPGPLGIAGPPGAR | 4 Oxidation (P) |
| 1076.02 | 2150.025 | 2150.024 | 65.3 | GEPGVVGAPGTAGPSGPSGLPGER | 3 Oxidation (P) |
| 1131.07 | 2260.125 | 2260.124 | 71.79 | GYPGNAGPVGAAGAPGPQGPVGPVGK | 2 Oxidation (P) |
| 1131.07 | 2260.125 | 2260.124 | 55.62 | GYPGNAGPVGAAGAPGPQGPVGPVGK | 2 Oxidation (P) |
| 1132.053 | 2262.091 | 2262.092 | 70.13 | GYPGNAGPVGAAGAPGPQGPVGPVGK | 2 Deamidated (NQ); 2 Oxidation (P) |
| 1205.574 | 2409.133 | 2409.131 | 88.69 | GEVGPAGPNGFAGPAGAAGQPGAKGER | Deamidated (NQ); Oxidation (K); Oxidation (P) |
| 810.746 | 2429.216 | 2429.219 | 40.41 | GEVGLPGLSGPVGPPGNPGANGLPGAK | 4 Oxidation (P) |
| 1215.617 | 2429.219 | 2429.219 | 61.62 | GEVGLPGLSGPVGPPGNPGANGLPGAK | 4 Oxidation (P) |
| 1216.108 | 2430.201 | 2430.203 | 80.23 | GEVGLPGLSGPVGPPGNPGANGLPGAK | Deamidated (NQ); 4 Oxidation (P) |
| 1224.105 | 2446.195 | 2446.198 | 71.2 | GEVGLPGLSGPVGPPGNPGANGLPGAK | Deamidated (NQ); Oxidation (K); 4 Oxidation (P) |
| 825.0976 | 2472.271 | 2472.272 | 61.98 | GERGLPGVAGSVGEPGPLGIAGPPGAR | 3 Oxidation (P) |
| 1287.135 | 2572.255 | 2572.256 | 110.97 | GSDGSVGPVGPAGPIGSAGPPGFPGAPGPK | Oxidation (K); Oxidation (P) |
| 861.0847 | 2580.232 | 2580.232 | 74.01 | GENGPVGPTGPVGAAGPSGPNGPPGPAGSR | Oxidation (P) |
| 861.412 | 2581.214 | 2581.216 | 59.26 | GENGPVGPTGPVGAAGPSGPNGPPGPAGSR | Deamidated (NQ); Oxidation (P) |
| 861.7404 | 2582.199 | 2582.2 | 41.88 | GENGPVGPTGPVGAAGPSGPNGPPGPAGSR | 2 Deamidated (NQ); Oxidation (P) |
| 863.758 | 2588.252 | 2588.251 | 60.18 | GSDGSVGPVGPAGPIGSAGPPGFPGAPGPK | 3 Oxidation (P) |
| 927.7826 | 2780.326 | 2780.323 | 42.92 | GAPGAIGAPGPAGANGDRGEAGPAGPAGPAGPR | Deamidated (NQ); 2 Oxidation (P) |
| 931.4464 | 2791.317 | 2791.316 | 56.8 | GEQGPAGPPGFQGLPGPAGTAGEAGKPGER | Oxidation (K); 2 Oxidation (P) |
| 931.7734 | 2792.298 | 2792.3 | 45.04 | GEQGPAGPPGFQGLPGPAGTAGEAGKPGER | Deamidated (NQ); Oxidation (K); 2 Oxidation (P) |
| 955.4694 | 2863.386 | 2863.385 | 53.04 | GPKGENGPVGPTGPVGAAGPSGPNGPPGPAGSR | Deamidated (NQ); Oxidation (P) |
| 955.7978 | 2864.372 | 2864.369 | 43.41 | GPKGENGPVGPTGPVGAAGPSGPNGPPGPAGSR | 2 Deamidated (NQ); Oxidation (P) |
| 960.801 | 2879.381 | 2879.38 | 65.85 | GPKGENGPVGPTGPVGAAGPSGPNGPPGPAGSR | Deamidated (NQ); Oxidation (K); Oxidation (P) |
| 961.1293 | 2880.366 | 2880.364 | 87.47 | GPKGENGPVGPTGPVGAAGPSGPNGPPGPAGSR | 2 Deamidated (NQ); Oxidation (K); Oxidation (P) |
| 1071.181 | 3210.521 | 3210.518 | 45.05 | GPSGPPGPDGNKGEPGVVGAPGTAGPSGPSGLPGER | 3 Oxidation (P) |

| Pigment epithelium-derived factor OS=Bos taurus GN=SERPINF1 PE=1 SV=1 | 869 |
| --- | --- |

| 535.3345 | 1068.654 | 1068.654 | 63.68 | TVQAVLTIPK | |
| --- | --- | --- | --- | --- | --- |
| 625.8345 | 1249.654 | 1249.655 | 78.38 | DTDTGALLFIGK | |
| 625.8353 | 1249.656 | 1249.655 | 42.15 | DTDTGALLFIGK | |
| 625.8353 | 1249.656 | 1249.655 | 61.49 | DTDTGALLFIGK | |
| 625.8354 | 1249.656 | 1249.655 | 74.59 | DTDTGALLFIGK | |
| 427.5749 | 1279.703 | 1279.702 | 41.66 | LKLSYEGELTK | |
| 684.8382 | 1367.662 | 1367.661 | 40.02 | LQSLFDAPDFSK | Deamidated (NQ) |
| 758.8439 | 1515.673 | 1515.673 | 78.12 | TSLEDFYLDEER | |
| 780.3961 | 1558.778 | 1558.778 | 57.82 | LAAAVSNFGYDLYR | |
| 780.3967 | 1558.779 | 1558.778 | 66.34 | LAAAVSNFGYDLYR | |
| 520.6003 | 1558.779 | 1558.778 | 63.17 | LAAAVSNFGYDLYR | |
| 520.9277 | 1559.761 | 1559.762 | 55.19 | LAAAVSNFGYDLYR | Deamidated (NQ) |
| 780.8887 | 1559.763 | 1559.762 | 88.77 | LAAAVSNFGYDLYR | Deamidated (NQ) |
| 648.6771 | 1943.01 | 1943.011 | 46.14 | EMPSEISIFLLGVAYFK | |
| 972.5132 | 1943.012 | 1943.011 | 96.31 | EMPSEISIFLLGVAYFK | |
| 991.9976 | 1981.981 | 1981.979 | 72.38 | ALYYDLISNPDIHGTYK | |
| 692.0696 | 2073.187 | 2073.187 | 43.99 | IAQLPLTGSTSIIFFLPQK | |
| 1037.601 | 2073.187 | 2073.187 | 86.7 | IAQLPLTGSTSIIFFLPQK | |
| 700.0363 | 2097.087 | 2097.09 | 52.21 | VPVNKLAAAVSNFGYDLYR | Deamidated (NQ) |
| 1117.112 | 2232.209 | 2232.21 | 60.65 | LTFPLDYHLNQPFIFVLR | |
| 1036.537 | 3106.589 | 3106.586 | 51.8 | ALYYDLISNPDIHGTYKDLLASVTAPQK | Deamidated (NQ) |

| Alpha-2-HS-glycoprotein OS=Bos taurus GN=AHSG PE=1 SV=2 | 501 |
| --- | --- |

| 408.7141 | 815.4136 | 815.4137 | 56.47 | ALGGEDVR | |
| --- | --- | --- | --- | --- | --- |
| 578.7954 | 1155.576 | 1155.577 | 45.94 | HTLNQIDSVK | 2 Deamidated (NQ) |
| 635.3273 | 1268.64 | 1268.64 | 49.38 | QDGQFSVLFTK | |
| 635.8193 | 1269.624 | 1269.624 | 49.99 | QDGQFSVLFTK | Deamidated (NQ) |
| 635.8195 | 1269.624 | 1269.624 | 53.23 | QDGQFSVLFTK | Deamidated (NQ) |
| 737.9219 | 1473.829 | 1473.83 | 73.36 | TPIVGQPSIPGGPVR | |
| 737.9222 | 1473.83 | 1473.83 | 66.96 | TPIVGQPSIPGGPVR | |
| 808.9134 | 1615.812 | 1615.813 | 54.32 | EVVDPTKCNLLAEK | Deamidated (NQ) |
| 707.3403 | 2118.999 | 2118.997 | 41.03 | HTFSGVASVESSSGEAFHVGK | |
| 1260.165 | 2518.315 | 2518.314 | 95.33 | AQFVPLPVSVSVEFAVAATDCIAK | |
| 840.4459 | 2518.316 | 2518.314 | 54.84 | AQFVPLPVSVSVEFAVAATDCIAK | |

| Biglycan OS=Bos taurus GN=BGN PE=1 SV=3 | 496 |
| --- | --- |

| 455.2735 | 908.5324 | 908.5331 | 43.2 | VPAGLPDLK | |
| --- | --- | --- | --- | --- | --- |
| 455.2738 | 908.533 | 908.5331 | 43.51 | VPAGLPDLK | |
| 504.7831 | 1007.552 | 1007.551 | 54.78 | LGLGHNQIR | Deamidated (NQ) |
| 527.7817 | 1053.549 | 1053.55 | 47.31 | LAIQFGNYK | Deamidated (NQ) |
| 559.797 | 1117.579 | 1117.58 | 47.11 | VVQCSDLGLK | |
| 399.8958 | 1196.666 | 1196.667 | 42.66 | IHEKAFSPLR | |
| 656.8769 | 1311.739 | 1311.74 | 62.8 | IQAIELEDLLR | |
| 747.8746 | 1493.735 | 1493.734 | 65.83 | VGVNDFCPVGFGVK | |
| 748.3657 | 1494.717 | 1494.718 | 47.06 | VGVNDFCPVGFGVK | Deamidated (NQ) |
| 748.366 | 1494.717 | 1494.718 | 49.02 | VGVNDFCPVGFGVK | Deamidated (NQ) |
| 791.4597 | 1580.905 | 1580.904 | 61.2 | GLQHLYALVLVNNK | |
| 791.9516 | 1581.889 | 1581.888 | 67.93 | GLQHLYALVLVNNK | Deamidated (NQ) |
| 792.4432 | 1582.872 | 1582.872 | 60.86 | GLQHLYALVLVNNK | 2 Deamidated (NQ) |
| 797.9109 | 1593.807 | 1593.807 | 70.27 | MIENGSLSFLPTLR | Deamidated (NQ); Oxidation (M) |
| 797.9109 | 1593.807 | 1593.807 | 76.8 | MIENGSLSFLPTLR | Deamidated (NQ); Oxidation (M) |
| 638.3623 | 1912.065 | 1912.067 | 46.3 | GLQHLYALVLVNNKISK | 3 Deamidated (NQ) |

| Serum albumin OS=Bos taurus GN=ALB PE=1 SV=4 | 401 |
| --- | --- |

| 508.3049 | 1014.595 | 1014.596 | 42.48 | QTALVELLK | Deamidated (NQ) |
| --- | --- | --- | --- | --- | --- |
| 582.3187 | 1162.623 | 1162.623 | 42.84 | LVNELTEFAK | |
| 480.6086 | 1438.804 | 1438.805 | 41.53 | RHPEYAVSVLLR | |
| 480.6087 | 1438.804 | 1438.805 | 52.03 | RHPEYAVSVLLR | |
| 740.4022 | 1478.79 | 1478.788 | 66.97 | LGEYGFQNALIVR | |
| 740.8939 | 1479.773 | 1479.772 | 76.1 | LGEYGFQNALIVR | Deamidated (NQ) |
| 784.3747 | 1566.735 | 1566.735 | 61.18 | DAFLGSFLYEYSR | |
| 784.3749 | 1566.735 | 1566.735 | 76.93 | DAFLGSFLYEYSR | |
| 682.3477 | 2044.021 | 2044.021 | 65.14 | RHPYFYAPELLYYANK | |
| 1246.636 | 2491.257 | 2491.257 | 83.31 | GLVLIAFSQYLQQCPFDEHVK | |

| Chondroadherin OS=Bos taurus GN=CHAD PE=1 SV=1 | 367 |
| --- | --- |

| 378.74 | 755.4654 | 755.4653 | 45.01 | LLNLQR |  |
| --- | --- | --- | --- | --- | --- |
| 678.3636 | 1354.713 | 1354.713 | 42.82 | FSDGAFLGVTTLK | |
| 678.3638 | 1354.713 | 1354.713 | 79.93 | FSDGAFLGVTTLK | |
| 678.3638 | 1354.713 | 1354.713 | 84.4 | FSDGAFLGVTTLK | |
| 452.5783 | 1354.713 | 1354.713 | 40.96 | FSDGAFLGVTTLK | |
| 678.364 | 1354.713 | 1354.713 | 98.91 | FSDGAFLGVTTLK | |
| 678.3641 | 1354.714 | 1354.713 | 46.58 | FSDGAFLGVTTLK | |
| 678.3644 | 1354.714 | 1354.713 | 43.25 | FSDGAFLGVTTLK | |
| 1005.59 | 2009.165 | 2009.167 | 50.87 | GLLSPLVNLFILQLNNNK | |
| 1141.168 | 2280.321 | 2280.321 | 50.92 | GLLSPLVNLFILQLNNNKIR | 2 Deamidated (NQ) |

| Complement C3 OS=Bos taurus GN=C3 PE=1 SV=2 | 249 |
| --- | --- |

| 634.8655 | 1267.716 | 1267.718 | 56.35 | LVAYYTLINAK |
| --- | --- | --- | --- | --- |
| 819.4492 | 1636.884 | 1636.883 | 70.54 | FVTVVATFGNVQVEK |
| 597.689 | 1790.045 | 1790.045 | 63.34 | RPYTVAIAAYALALLGK |
| 627.6875 | 1880.041 | 1880.041 | 65.97 | LYNVEATSYALLALLAR |
| 941.0284 | 1880.042 | 1880.041 | 100.59 | LYNVEATSYALLALLAR |

| Decorin OS=Bos taurus GN=DCN PE=1 SV=2 | 193 |
| --- | --- |

| 550.3285 | 1098.642 | 1098.644 | 56.02 | ISPGAFAPLVK | |
| --- | --- | --- | --- | --- | --- |
| 647.3793 | 1292.744 | 1292.745 | 49.98 | NLHTLILINNK | Deamidated (NQ) |
| 647.3796 | 1292.745 | 1292.745 | 55.31 | NLHTLILINNK | Deamidated (NQ) |
| 541.9819 | 1622.924 | 1622.924 | 41.09 | NLHTLILINNKISK | 3 Deamidated (NQ) |
| 796.0681 | 2385.183 | 2385.181 | 65.51 | LGLSFNSISAVDNGSLANTPHLR | 3 Deamidated (NQ) |

| Olfactomedin-like protein 3 OS=Bos taurus GN=OLFML3 PE=2 SV=1 | 181 |
| --- | --- |

| 517.2763 | 1032.538 | 1032.539 | 74.86 | FGGPAGLWTK |
| --- | --- | --- | --- | --- |
| 1195.62 | 2389.225 | 2389.226 | 102.19 | VPFPWVGTGQLVYGGFLYYAR |

| Apolipoprotein A-I OS=Bos taurus GN=APOA1 PE=1 SV=3 | 179 |
| --- | --- |

| 608.8421 | 1215.67 | 1215.671 | 87.57 | VSILAAIDEASK |
| --- | --- | --- | --- | --- |
| 628.3325 | 1254.65 | 1254.65 | 70.71 | DFATVYVEAIK |
| 699.8487 | 1397.683 | 1397.683 | 79.56 | DYVAQFEASALGK |

| Collagen alpha-2(XI) chain OS=Bos taurus GN=COL11A2 PE=3 SV=1 | 178 |
| --- | --- |

| 511.2876 | 1020.561 | 1020.56 | 60 | DFSLLTAVR | |
| --- | --- | --- | --- | --- | --- |
| 428.2454 | 1281.714 | 1281.715 | 43.92 | VSRPAQLSAPTR | |
| 1006.076 | 2010.137 | 2010.137 | 40.16 | ARPGLQAPLLTLYSAQGVR | |
| 671.0535 | 2010.139 | 2010.137 | 45.89 | ARPGLQAPLLTLYSAQGVR | |
| 671.3806 | 2011.12 | 2011.121 | 67.03 | ARPGLQAPLLTLYSAQGVR | Deamidated (NQ) |

| Prothrombin OS=Bos taurus GN=F2 PE=1 SV=2 | 147 |
| --- | --- |

| 456.7795 | 911.5444 | 911.544 | 45.66 | VTVEVIPR |
| --- | --- | --- | --- | --- |
| 621.8033 | 1241.592 | 1241.593 | 57.91 | ELFESYIEGR |
| 1137.584 | 2273.153 | 2273.151 | 82.36 | IVEGQDAEVGLSPWQVMLFR |

| Serine protease HTRA1 OS=Bos taurus GN=HTRA1 PE=2 SV=1 | 144 |
| --- | --- |

| 440.8023 | 879.59 | 879.5906 | 45.9 | LPVLLLGR |  |
| --- | --- | --- | --- | --- | --- |
| 653.3557 | 1304.697 | 1304.698 | 71.93 | VTAGISFAIPSDK | |
| 682.9063 | 1363.798 | 1363.798 | 65.39 | IAPAVVHIELFR | |

| Lumican OS=Bos taurus GN=LUM PE=1 SV=1 | 142 |
| --- | --- |

| 499.2765 | 996.5384 | 996.5392 | 49.67 | FSALQYLR | |
| --- | --- | --- | --- | --- | --- |
| 499.2768 | 996.539 | 996.5392 | 47.16 | FSALQYLR | |
| 499.2768 | 996.539 | 996.5392 | 45.69 | FSALQYLR | |
| 499.7685 | 997.5224 | 997.5233 | 50.09 | FSALQYLR | Deamidated (NQ) |
| 385.2204 | 1152.639 | 1152.64 | 46.08 | RFSALQYLR | |
| 609.8761 | 1217.738 | 1217.738 | 42.73 | ILGPLSYSKIK | |

| Nucleobindin-1 OS=Bos taurus GN=NUCB1 PE=2 SV=1 | 140 |
| --- | --- |

| 636.8615 | 1271.708 | 1271.709 | 89.42 | DLELLIQTATR |
| --- | --- | --- | --- | --- |
| 644.9986 | 1931.974 | 1931.974 | 40.85 | YLQEVINVLETDGHFR |
| 644.9988 | 1931.975 | 1931.974 | 52.17 | YLQEVINVLETDGHFR |

| Alkaline phosphatase, tissue-nonspecific isozyme OS=Bos taurus GN=ALPL PE=1 SV=2 | 132 |
| --- | --- |

| 467.2713 | 932.528 | 932.5291 | 47.15 | SVGIVTTTR |
| --- | --- | --- | --- | --- |
| 547.8033 | 1093.592 | 1093.592 | 55.01 | GFFLLVEGGR |
| 969.9927 | 1937.971 | 1937.97 | 75.02 | NVIMFLGDGMGVSTVTAAR |

| Coagulation factor IX (Fragment) OS=Bos taurus GN=F9 PE=1 SV=1 | 115 |
| --- | --- |

| 511.7975 | 1021.58 | 1021.581 | 63.39 | SASILQYLK | |
| --- | --- | --- | --- | --- | --- |
| 512.2898 | 1022.565 | 1022.565 | 41.59 | SASILQYLK | Deamidated (NQ) |
| 610.7897 | 1219.565 | 1219.566 | 53.93 | FGYGYVSGWGK | |
| 783.4344 | 1564.854 | 1564.855 | 40.16 | WVVTAAHCIKPGVK | |

| Thrombospondin-1 OS=Bos taurus GN=THBS1 PE=2 SV=2 | 104 |
| --- | --- |

| 429.7557 | 857.4968 | 857.4971 | 41.35 | GTLLAVER |
| --- | --- | --- | --- | --- |
| 495.3106 | 988.6066 | 988.6069 | 52.8 | GFLLLASLR |
| 495.3109 | 988.6072 | 988.6069 | 58.18 | GFLLLASLR |
| 531.7828 | 1061.551 | 1061.551 | 51.93 | FQDLVDAVR |

| Tetranectin OS=Bos taurus GN=CLEC3B PE=2 SV=1 | 102 |
| --- | --- |

| 542.7784 | 1083.542 | 1083.542 | 42.09 | CFLAFVQAK | Deamidated (NQ) |
| --- | --- | --- | --- | --- | --- |
| 764.9329 | 1527.851 | 1527.851 | 90.96 | TQLDSLAQEVALLK | |

| Serpin H1 OS=Bos taurus GN=SERPINH1 PE=2 SV=1 | 94 |
| --- | --- |

| 729.3766 | 1456.739 | 1456.738 | 64.4 | SAGLAFSLYQAMAK |
| --- | --- | --- | --- | --- |
| 819.4453 | 1636.876 | 1636.877 | 56.78 | LFYADHPFIFLVR |

| SPARC OS=Bos taurus GN=SPARC PE=1 SV=2 | 88 |
| --- | --- |

| 710.3829 | 1418.751 | 1418.752 | 55.05 | LEAGDHPVELLAR |
| --- | --- | --- | --- | --- |
| 821.9005 | 1641.786 | 1641.786 | 64.49 | YIALDEWAGCFGIK |

| Collagen alpha-1(III) chain OS=Bos taurus GN=COL3A1 PE=1 SV=1 | 73 |
| --- | --- |

| 645.2972 | 1288.58 | 1288.58 | 50.36 | GSPGGPGAAGFPGGR | 3 Oxidation (P) |
| --- | --- | --- | --- | --- | --- |
| 500.9108 | 1499.711 | 1499.712 | 42.38 | DGASGHPGPIGPPGPR | 2 Oxidation (P) |

**KC6**

| Collagen alpha-1(I) chain OS=Bos taurus GN=COL1A1 PE=1 SV=3 | 3981 |
| --- | --- |

| **pep_exp_mz** | **pep_exp_mr** | **pep_calc_mr** | **pep_score** | **pep_seq** | **pep_var_mod** |
| --- | --- | --- | --- | --- | --- |
| 392.2217 | 782.4288 | 782.4286 | 42.78 | GAAGLPGPK | Oxidation (P) |
| 392.2218 | 782.429 | 782.4286 | 44.68 | GAAGLPGPK | Oxidation (P) |
| 426.2166 | 850.4186 | 850.4185 | 42.49 | GFSGLDGAK | |
| 426.2167 | 850.4188 | 850.4185 | 46.11 | GFSGLDGAK | |
| 449.7585 | 897.5024 | 897.5032 | 44.78 | GVVGLPGQR | Oxidation (P) |
| 449.7586 | 897.5026 | 897.5032 | 41.35 | GVVGLPGQR | Oxidation (P) |
| 450.2506 | 898.4866 | 898.4872 | 44.78 | GVVGLPGQR | Deamidated (NQ); Oxidation (P) |
| 464.7402 | 927.4658 | 927.4662 | 51.1 | PGEAGLPGAK | 2 Oxidation (P) |
| 544.7715 | 1087.528 | 1087.53 | 40.36 | GFPGADGVAGPK | Oxidation (P) |
| 544.772 | 1087.529 | 1087.53 | 62.25 | GFPGADGVAGPK | Oxidation (P) |
| 544.772 | 1087.529 | 1087.53 | 52.1 | GFPGADGVAGPK | Oxidation (P) |
| 544.772 | 1087.529 | 1087.53 | 42.77 | GFPGADGVAGPK | Oxidation (P) |
| 544.7721 | 1087.53 | 1087.53 | 41.09 | GFPGADGVAGPK | Oxidation (P) |
| 544.7723 | 1087.53 | 1087.53 | 47.21 | GFPGADGVAGPK | Oxidation (P) |
| 545.7856 | 1089.557 | 1089.557 | 44.56 | GVQGPPGPAGPR | Deamidated (NQ) |
| 553.2909 | 1104.567 | 1104.568 | 41.67 | GVQGPPGPAGPR | Oxidation (P) |
| 553.2913 | 1104.568 | 1104.568 | 40.92 | GVQGPPGPAGPR | Oxidation (P) |
| 553.7833 | 1105.552 | 1105.552 | 57.34 | GVQGPPGPAGPR | Deamidated (NQ); Oxidation (P) |
| 553.7833 | 1105.552 | 1105.552 | 41.08 | GVQGPPGPAGPR | Deamidated (NQ); Oxidation (P) |
| 573.7841 | 1145.554 | 1145.554 | 49.59 | GQAGVMGFPGPK | Deamidated (NQ) |
| 581.29 | 1160.565 | 1160.565 | 45.87 | GQAGVMGFPGPK | Oxidation (M) |
| 581.7817 | 1161.549 | 1161.549 | 65.2 | GQAGVMGFPGPK | Deamidated (NQ); Oxidation (M) |
| 581.782 | 1161.549 | 1161.549 | 61.35 | GQAGVMGFPGPK | Deamidated (NQ); Oxidation (K) |
| 588.8221 | 1175.63 | 1175.63 | 44.25 | GVPGPPGAVGPAGK | Oxidation (P) |
| 589.287 | 1176.559 | 1176.56 | 61.12 | GQAGVMGFPGPK | Oxidation (M); Oxidation (P) |
| 589.2873 | 1176.56 | 1176.56 | 62.94 | GQAGVMGFPGPK | Oxidation (M); Oxidation (P) |
| 589.7781 | 1177.542 | 1177.544 | 40.45 | GQAGVMGFPGPK | Deamidated (NQ); Oxidation (M); Oxidation (P) |
| 589.7789 | 1177.543 | 1177.544 | 50.46 | GQAGVMGFPGPK | Deamidated (NQ); Oxidation (M); Oxidation (P) |
| 589.7791 | 1177.544 | 1177.544 | 63.21 | GQAGVMGFPGPK | Deamidated (NQ); Oxidation (M); Oxidation (P) |
| 589.7791 | 1177.544 | 1177.544 | 47.26 | GQAGVMGFPGPK | Deamidated (NQ); Oxidation (M); Oxidation (P) |
| 589.7792 | 1177.544 | 1177.544 | 43.74 | GQAGVMGFPGPK | Deamidated (NQ); Oxidation (M); Oxidation (P) |
| 589.7793 | 1177.544 | 1177.544 | 68.08 | GQAGVMGFPGPK | Deamidated (NQ); Oxidation (M); Oxidation (P) |
| 589.7795 | 1177.544 | 1177.544 | 77.07 | GQAGVMGFPGPK | Deamidated (NQ); Oxidation (M); Oxidation (P) |
| 597.7758 | 1193.537 | 1193.539 | 58.17 | GQAGVMGFPGPK | Deamidated (NQ); Oxidation (K); Oxidation (M); Oxidation (P) |
| 597.7764 | 1193.538 | 1193.539 | 54.13 | GQAGVMGFPGPK | Deamidated (NQ); Oxidation (K); Oxidation (M); Oxidation (P) |
| 621.8018 | 1241.589 | 1241.589 | 57.18 | GLTGSPGSPGPDGK | Oxidation (P) |
| 629.7991 | 1257.584 | 1257.584 | 47.88 | GLTGSPGSPGPDGK | 2 Oxidation (P) |
| 629.7993 | 1257.584 | 1257.584 | 53.31 | GLTGSPGSPGPDGK | 2 Oxidation (P) |
| 629.7993 | 1257.584 | 1257.584 | 43.15 | GLTGSPGSPGPDGK | 2 Oxidation (P) |
| 629.7994 | 1257.584 | 1257.584 | 54.14 | GLTGSPGSPGPDGK | 2 Oxidation (P) |
| 641.3127 | 1280.611 | 1280.611 | 42.39 | GEAGPSGPAGPTGAR | |
| 656.8298 | 1311.645 | 1311.646 | 58.84 | GFPGLPGPSGEPGK | Oxidation (P) |
| 664.8269 | 1327.639 | 1327.641 | 66.43 | GFPGLPGPSGEPGK | 2 Oxidation (P) |
| 664.8278 | 1327.641 | 1327.641 | 54.89 | GFPGLPGPSGEPGK | 2 Oxidation (P) |
| 664.8278 | 1327.641 | 1327.641 | 44.33 | GFPGLPGPSGEPGK | 2 Oxidation (P) |
| 664.8281 | 1327.642 | 1327.641 | 56.39 | GFPGLPGPSGEPGK | 2 Oxidation (P) |
| 666.8306 | 1331.647 | 1331.647 | 79.31 | GPSGPQGPSGPPGPK | Oxidation (P) |
| 666.8306 | 1331.647 | 1331.647 | 57.32 | GPSGPQGPSGPPGPK | Oxidation (P) |
| 666.8312 | 1331.648 | 1331.647 | 81.59 | GPSGPQGPSGPPGPK | Oxidation (P) |
| 666.8312 | 1331.648 | 1331.647 | 58.76 | GPSGPQGPSGPPGPK | Oxidation (P) |
| 666.8312 | 1331.648 | 1331.647 | 47.4 | GPSGPQGPSGPPGPK | Oxidation (P) |
| 667.3223 | 1332.63 | 1332.631 | 48.51 | GPSGPQGPSGPPGPK | Deamidated (NQ); Oxidation (P) |
| 667.3232 | 1332.632 | 1332.631 | 53.2 | GPSGPQGPSGPPGPK | Deamidated (NQ); Oxidation (P) |
| 667.3233 | 1332.632 | 1332.631 | 52.27 | GPSGPQGPSGPPGPK | Deamidated (NQ); Oxidation (P) |
| 667.3234 | 1332.632 | 1332.631 | 86.72 | GPSGPQGPSGPPGPK | Deamidated (NQ); Oxidation (P) |
| 672.8248 | 1343.635 | 1343.636 | 48.65 | GFPGLPGPSGEPGK | 3 Oxidation (P) |
| 672.825 | 1343.635 | 1343.636 | 41.47 | GFPGLPGPSGEPGK | 3 Oxidation (P) |
| 675.3201 | 1348.626 | 1348.626 | 40.41 | GPSGPQGPSGPPGPK | Deamidated (NQ); 2 Oxidation (P) |
| 718.3448 | 1434.675 | 1434.674 | 65.87 | GEPGPAGLPGPPGER | 3 Oxidation (P) |
| 718.3448 | 1434.675 | 1434.674 | 46.58 | GEPGPAGLPGPPGER | 3 Oxidation (P) |
| 718.3448 | 1434.675 | 1434.674 | 44.72 | GEPGPAGLPGPPGER | 3 Oxidation (P) |
| 718.345 | 1434.675 | 1434.674 | 79.24 | GEPGPAGLPGPPGER | 3 Oxidation (P) |
| 718.3453 | 1434.676 | 1434.674 | 41.67 | GEPGPAGLPGPPGER | 3 Oxidation (P) |
| 730.3493 | 1458.684 | 1458.685 | 75.25 | GSAGPPGATGFPGAAGR | 2 Oxidation (P) |
| 730.3495 | 1458.684 | 1458.685 | 82.63 | GSAGPPGATGFPGAAGR | 2 Oxidation (P) |
| 487.2355 | 1458.685 | 1458.685 | 61.96 | GSAGPPGATGFPGAAGR | 2 Oxidation (P) |
| 730.3497 | 1458.685 | 1458.685 | 89.31 | GSAGPPGATGFPGAAGR | 2 Oxidation (P) |
| 730.3497 | 1458.685 | 1458.685 | 49 | GSAGPPGATGFPGAAGR | 2 Oxidation (P) |
| 730.3497 | 1458.685 | 1458.685 | 43.18 | GSAGPPGATGFPGAAGR | 2 Oxidation (P) |
| 730.3498 | 1458.685 | 1458.685 | 56.59 | GSAGPPGATGFPGAAGR | 2 Oxidation (P) |
| 730.35 | 1458.685 | 1458.685 | 53.35 | GSAGPPGATGFPGAAGR | 2 Oxidation (P) |
| 730.35 | 1458.685 | 1458.685 | 99.96 | GSAGPPGATGFPGAAGR | 2 Oxidation (P) |
| 730.3502 | 1458.686 | 1458.685 | 94.3 | GSAGPPGATGFPGAAGR | 2 Oxidation (P) |
| 730.3502 | 1458.686 | 1458.685 | 68 | GSAGPPGATGFPGAAGR | 2 Oxidation (P) |
| 730.3502 | 1458.686 | 1458.685 | 41.38 | GSAGPPGATGFPGAAGR | 2 Oxidation (P) |
| 730.3502 | 1458.686 | 1458.685 | 43.9 | GSAGPPGATGFPGAAGR | 2 Oxidation (P) |
| 730.3503 | 1458.686 | 1458.685 | 64.25 | GSAGPPGATGFPGAAGR | 2 Oxidation (P) |
| 730.3505 | 1458.686 | 1458.685 | 42.07 | GSAGPPGATGFPGAAGR | 2 Oxidation (P) |
| 730.3506 | 1458.687 | 1458.685 | 55.66 | GSAGPPGATGFPGAAGR | 2 Oxidation (P) |
| 730.351 | 1458.687 | 1458.685 | 70.53 | GSAGPPGATGFPGAAGR | 2 Oxidation (P) |
| 730.3514 | 1458.688 | 1458.685 | 55.68 | GSAGPPGATGFPGAAGR | 2 Oxidation (P) |
| 730.3524 | 1458.69 | 1458.685 | 41.76 | GSAGPPGATGFPGAAGR | 2 Oxidation (P) |
| 520.9426 | 1559.806 | 1559.806 | 59.74 | GETGPAGPAGPIGPVGAR | |
| 780.9107 | 1559.807 | 1559.806 | 68.93 | GETGPAGPAGPIGPVGAR | |
| 781.403 | 1560.791 | 1560.79 | 45.98 | DGLNGLPGPIGPPGPR | 3 Oxidation (P) |
| 781.8942 | 1561.774 | 1561.774 | 61.37 | DGLNGLPGPIGPPGPR | Deamidated (NQ); 3 Oxidation (P) |
| 781.8942 | 1561.774 | 1561.774 | 64.79 | DGLNGLPGPIGPPGPR | Deamidated (NQ); 3 Oxidation (P) |
| 781.8949 | 1561.775 | 1561.774 | 45.83 | DGLNGLPGPIGPPGPR | Deamidated (NQ); 3 Oxidation (P) |
| 793.3899 | 1584.765 | 1584.765 | 45.66 | GANGAPGIAGAPGFPGAR | 3 Oxidation (P) |
| 793.8811 | 1585.748 | 1585.749 | 44.41 | GANGAPGIAGAPGFPGAR | Deamidated (NQ); 3 Oxidation (P) |
| 793.8816 | 1585.749 | 1585.749 | 45.21 | GANGAPGIAGAPGFPGAR | Deamidated (NQ); 3 Oxidation (P) |
| 793.8819 | 1585.749 | 1585.749 | 57.03 | GANGAPGIAGAPGFPGAR | Deamidated (NQ); 3 Oxidation (P) |
| 793.882 | 1585.749 | 1585.749 | 69.71 | GANGAPGIAGAPGFPGAR | Deamidated (NQ); 3 Oxidation (P) |
| 534.6018 | 1600.784 | 1600.785 | 53.77 | GFSGLDGAKGDAGPAGPK | |
| 534.6021 | 1600.785 | 1600.785 | 43.51 | GFSGLDGAKGDAGPAGPK | |
| 808.3954 | 1614.776 | 1614.775 | 45.44 | GGPGSRGFPGADGVAGPK | 2 Oxidation (P) |
| 552.6046 | 1654.792 | 1654.791 | 53.98 | GSPGEAGRPGEAGLPGAK | Oxidation (K); 2 Oxidation (P) |
| 846.385 | 1690.755 | 1690.755 | 88.06 | DGEAGAQGPPGPAGPAGER | Deamidated (NQ) |
| 569.5958 | 1705.766 | 1705.766 | 45.17 | DGEAGAQGPPGPAGPAGER | Oxidation (P) |
| 853.8904 | 1705.766 | 1705.766 | 95.26 | DGEAGAQGPPGPAGPAGER | Oxidation (P) |
| 853.8913 | 1705.768 | 1705.766 | 55.44 | DGEAGAQGPPGPAGPAGER | Oxidation (P) |
| 854.3798 | 1706.745 | 1706.75 | 73.09 | DGEAGAQGPPGPAGPAGER | Deamidated (NQ); Oxidation (P) |
| 569.9237 | 1706.749 | 1706.75 | 42.16 | DGEAGAQGPPGPAGPAGER | Deamidated (NQ); Oxidation (P) |
| 854.3827 | 1706.751 | 1706.75 | 69.31 | DGEAGAQGPPGPAGPAGER | Deamidated (NQ); Oxidation (P) |
| 581.9555 | 1742.845 | 1742.845 | 47.83 | GARGSAGPPGATGFPGAAGR | 2 Oxidation (P) |
| 581.9557 | 1742.845 | 1742.845 | 50.13 | GARGSAGPPGATGFPGAAGR | 2 Oxidation (P) |
| 915.4375 | 1828.86 | 1828.859 | 49.6 | VGPPGPSGNAGPPGPPGPAGK | Deamidated (NQ); 4 Oxidation (P) |
| 915.4376 | 1828.861 | 1828.859 | 42.3 | VGPPGPSGNAGPPGPPGPAGK | Deamidated (NQ); 4 Oxidation (P) |
| 915.4388 | 1828.863 | 1828.859 | 45.22 | VGPPGPSGNAGPPGPPGPAGK | Deamidated (NQ); Oxidation (K); 3 Oxidation (P) |
| 916.9335 | 1831.852 | 1831.852 | 64.44 | GPPGPMGPPGLAGPPGESGR | Oxidation (M); 2 Oxidation (P) |
| 611.6248 | 1831.853 | 1831.852 | 42.95 | GPPGPMGPPGLAGPPGESGR | Oxidation (M); 2 Oxidation (P) |
| 916.9341 | 1831.854 | 1831.852 | 64.43 | GPPGPMGPPGLAGPPGESGR | Oxidation (M); 2 Oxidation (P) |
| 924.9301 | 1847.846 | 1847.847 | 48.94 | GPPGPMGPPGLAGPPGESGR | Oxidation (M); 3 Oxidation (P) |
| 932.9312 | 1863.848 | 1863.849 | 63.37 | GEPGPTGIQGPPGPAGEEGK | Deamidated (NQ); 2 Oxidation (P) |
| 659.3362 | 1974.987 | 1974.987 | 48.13 | SGDRGETGPAGPAGPIGPVGAR | |
| 659.3365 | 1974.988 | 1974.987 | 54.78 | SGDRGETGPAGPAGPIGPVGAR | |
| 988.5013 | 1974.988 | 1974.987 | 59.75 | SGDRGETGPAGPAGPIGPVGAR | |
| 988.5016 | 1974.989 | 1974.987 | 53.8 | SGDRGETGPAGPAGPIGPVGAR | |
| 996.4973 | 1990.98 | 1990.982 | 52.19 | SGDRGETGPAGPAGPIGPVGAR | Oxidation (P) |
| 1010.491 | 2018.967 | 2018.966 | 79.84 | GEPGPTGIQGPPGPAGEEGKR | 2 Oxidation (P) |
| 674.324 | 2019.95 | 2019.95 | 47.14 | GEPGPTGIQGPPGPAGEEGKR | Deamidated (NQ); 2 Oxidation (P) |
| 1010.983 | 2019.951 | 2019.95 | 76.43 | GEPGPTGIQGPPGPAGEEGKR | Deamidated (NQ); 2 Oxidation (P) |
| 686.3317 | 2055.973 | 2055.972 | 40.28 | TGPPGPAGQDGRPGPPGPPGAR | 4 Oxidation (P) |
| 1029.506 | 2056.997 | 2056.993 | 55.2 | GAPGADGPAGAPGTPGPQGIAGQR | Deamidated (NQ) |
| 1037.502 | 2072.989 | 2072.988 | 77.31 | GAPGADGPAGAPGTPGPQGIAGQR | Deamidated (NQ); Oxidation (P) |
| 1037.503 | 2072.991 | 2072.988 | 53.45 | GAPGADGPAGAPGTPGPQGIAGQR | Deamidated (NQ); Oxidation (P) |
| 1037.993 | 2073.971 | 2073.972 | 86.23 | GAPGADGPAGAPGTPGPQGIAGQR | 2 Deamidated (NQ); Oxidation (P) |
| 1045.498 | 2088.981 | 2088.983 | 55.42 | GAPGADGPAGAPGTPGPQGIAGQR | Deamidated (NQ); 2 Oxidation (P) |
| 1045.5 | 2088.985 | 2088.983 | 61.71 | GAPGADGPAGAPGTPGPQGIAGQR | Deamidated (NQ); 2 Oxidation (P) |
| 697.6629 | 2089.967 | 2089.967 | 50.1 | GAPGADGPAGAPGTPGPQGIAGQR | 2 Deamidated (NQ); 2 Oxidation (P) |
| 1045.991 | 2089.967 | 2089.967 | 74.35 | GAPGADGPAGAPGTPGPQGIAGQR | 2 Deamidated (NQ); 2 Oxidation (P) |
| 1053.497 | 2104.979 | 2104.977 | 43.15 | GAPGADGPAGAPGTPGPQGIAGQR | Deamidated (NQ); 3 Oxidation (P) |
| 1053.498 | 2104.981 | 2104.977 | 53.44 | GAPGADGPAGAPGTPGPQGIAGQR | Deamidated (NQ); 3 Oxidation (P) |
| 1053.988 | 2105.961 | 2105.961 | 70.93 | GAPGADGPAGAPGTPGPQGIAGQR | 2 Deamidated (NQ); 3 Oxidation (P) |
| 1053.988 | 2105.961 | 2105.961 | 71.96 | GAPGADGPAGAPGTPGPQGIAGQR | 2 Deamidated (NQ); 3 Oxidation (P) |
| 1067.987 | 2133.959 | 2133.96 | 41.79 | GEPGPPGPAGFAGPPGADGQPGAK | Deamidated (NQ); 3 Oxidation (P) |
| 1085.033 | 2168.051 | 2168.05 | 53.74 | GETGPAGPPGAPGAPGAPGPVGPAGK | 3 Oxidation (P) |
| 723.6911 | 2168.052 | 2168.05 | 44.78 | GETGPAGPPGAPGAPGAPGPVGPAGK | 3 Oxidation (P) |
| 1093.031 | 2184.047 | 2184.045 | 62.57 | GETGPAGPPGAPGAPGAPGPVGPAGK | 4 Oxidation (P) |
| 1108.973 | 2215.931 | 2215.929 | 88.29 | GDAGAPGAPGSQGAPGLQGMPGER | 2 Deamidated (NQ); Oxidation (M); 4 Oxidation (P) |
| 1136.526 | 2271.037 | 2271.04 | 48.98 | GFPGLPGPSGEPGKQGPSGASGER | Deamidated (NQ); Oxidation (K); 2 Oxidation (P) |
| 764.3852 | 2290.134 | 2290.134 | 48.46 | GDAGPPGPAGPAGPPGPIGNVGAPGPK | Oxidation (K); Oxidation (P) |
| 764.7141 | 2291.121 | 2291.118 | 55.49 | GDAGPPGPAGPAGPPGPIGNVGAPGPK | Deamidated (NQ); Oxidation (K); Oxidation (P) |
| 1146.568 | 2291.121 | 2291.118 | 101.4 | GDAGPPGPAGPAGPPGPIGNVGAPGPK | Deamidated (NQ); Oxidation (K); Oxidation (P) |
| 773.0161 | 2316.027 | 2316.026 | 50.66 | GEPGPPGPAGAAGPAGNPGADGQPGAK | Deamidated (NQ); 4 Oxidation (P) |
| 1162.072 | 2322.129 | 2322.124 | 55.35 | GDAGPPGPAGPAGPPGPIGNVGAPGPK | 4 Oxidation (P) |
| 807.6979 | 2420.072 | 2420.073 | 45.03 | GPSGPQGPSGPPGPKGNSGEPGAPGSK | 2 Deamidated (NQ); Oxidation (K); 3 Oxidation (P) |
| 819.0789 | 2454.215 | 2454.214 | 59.88 | GPPGSAGSPGKDGLNGLPGPIGPPGPR | Deamidated (NQ); 3 Oxidation (P) |
| 822.4103 | 2464.209 | 2464.21 | 45.83 | GDRGETGPAGPPGAPGAPGAPGPVGPAGK | Oxidation (P) |
| 829.7418 | 2486.204 | 2486.204 | 42.94 | GPPGSAGSPGKDGLNGLPGPIGPPGPR | Deamidated (NQ); 5 Oxidation (P) |
| 1244.11 | 2486.205 | 2486.204 | 48.45 | GPPGSAGSPGKDGLNGLPGPIGPPGPR | Deamidated (NQ); 5 Oxidation (P) |
| 829.7443 | 2486.211 | 2486.204 | 45.78 | GPPGSAGSPGKDGLNGLPGPIGPPGPR | Deamidated (NQ); 5 Oxidation (P) |
| 833.0746 | 2496.202 | 2496.199 | 44.55 | GDRGETGPAGPPGAPGAPGAPGPVGPAGK | 3 Oxidation (P) |
| 837.3668 | 2509.079 | 2509.078 | 54.04 | GDAGPAGPKGEPGSPGENGAPGQMGPR | Deamidated (NQ); Oxidation (M); 3 Oxidation (P) |
| 837.695 | 2510.063 | 2510.062 | 56.63 | GDAGPAGPKGEPGSPGENGAPGQMGPR | 2 Deamidated (NQ); Oxidation (M); 3 Oxidation (P) |
| 838.4057 | 2512.195 | 2512.194 | 43.84 | GDRGETGPAGPPGAPGAPGAPGPVGPAGK | 4 Oxidation (P) |
| 838.4066 | 2512.198 | 2512.194 | 51.99 | GDRGETGPAGPPGAPGAPGAPGPVGPAGK | 4 Oxidation (P) |
| 844.7429 | 2531.207 | 2531.204 | 50.57 | GNDGATGAAGPPGPTGPAGPPGFPGAVGAK | Oxidation (K); Oxidation (P) |
| 1267.102 | 2532.189 | 2532.188 | 40.84 | GNDGATGAAGPPGPTGPAGPPGFPGAVGAK | Deamidated (NQ); 2 Oxidation (P) |
| 845.0712 | 2532.192 | 2532.188 | 40.46 | GNDGATGAAGPPGPTGPAGPPGFPGAVGAK | Deamidated (NQ); Oxidation (K); Oxidation (P) |
| 1274.606 | 2547.197 | 2547.199 | 52.24 | GNDGATGAAGPPGPTGPAGPPGFPGAVGAK | 3 Oxidation (P) |
| 850.0733 | 2547.198 | 2547.199 | 44.61 | GNDGATGAAGPPGPTGPAGPPGFPGAVGAK | 3 Oxidation (P) |
| 1275.101 | 2548.187 | 2548.183 | 65.07 | GNDGATGAAGPPGPTGPAGPPGFPGAVGAK | Deamidated (NQ); 3 Oxidation (P) |
| 1275.102 | 2548.189 | 2548.183 | 83.3 | GNDGATGAAGPPGPTGPAGPPGFPGAVGAK | Deamidated (NQ); 3 Oxidation (P) |
| 1283.098 | 2564.181 | 2564.178 | 62.33 | GNDGATGAAGPPGPTGPAGPPGFPGAVGAK | Deamidated (NQ); Oxidation (K); 3 Oxidation (P) |
| 870.0969 | 2607.269 | 2607.268 | 58.42 | GDAGPPGPAGPAGPPGPIGNVGAPGPKGAR | Deamidated (NQ); 4 Oxidation (P) |
| 870.0982 | 2607.273 | 2607.268 | 49.47 | GDAGPPGPAGPAGPPGPIGNVGAPGPKGAR | Deamidated (NQ); 4 Oxidation (P) |
| 897.0923 | 2688.255 | 2688.253 | 48.82 | GFSGLQGPPGPPGSPGEQGPSGASGPAGPR | 2 Oxidation (P) |
| 1345.623 | 2689.231 | 2689.237 | 50.01 | GFSGLQGPPGPPGSPGEQGPSGASGPAGPR | Deamidated (NQ); 2 Oxidation (P) |
| 897.4188 | 2689.235 | 2689.237 | 61.85 | GFSGLQGPPGPPGSPGEQGPSGASGPAGPR | Deamidated (NQ); 2 Oxidation (P) |
| 1345.625 | 2689.235 | 2689.237 | 84.3 | GFSGLQGPPGPPGSPGEQGPSGASGPAGPR | Deamidated (NQ); 2 Oxidation (P) |
| 1345.626 | 2689.237 | 2689.237 | 48.74 | GFSGLQGPPGPPGSPGEQGPSGASGPAGPR | Deamidated (NQ); 2 Oxidation (P) |
| 902.4237 | 2704.249 | 2704.248 | 62.8 | GFSGLQGPPGPPGSPGEQGPSGASGPAGPR | 3 Oxidation (P) |
| 1353.621 | 2705.227 | 2705.232 | 78.37 | GFSGLQGPPGPPGSPGEQGPSGASGPAGPR | Deamidated (NQ); 3 Oxidation (P) |
| 1353.622 | 2705.229 | 2705.232 | 58.31 | GFSGLQGPPGPPGSPGEQGPSGASGPAGPR | Deamidated (NQ); 3 Oxidation (P) |
| 902.7507 | 2705.23 | 2705.232 | 74.61 | GFSGLQGPPGPPGSPGEQGPSGASGPAGPR | Deamidated (NQ); 3 Oxidation (P) |
| 902.7507 | 2705.23 | 2705.232 | 41.9 | GFSGLQGPPGPPGSPGEQGPSGASGPAGPR | Deamidated (NQ); 3 Oxidation (P) |
| 1353.625 | 2705.235 | 2705.232 | 69.62 | GFSGLQGPPGPPGSPGEQGPSGASGPAGPR | Deamidated (NQ); 3 Oxidation (P) |
| 1353.625 | 2705.235 | 2705.232 | 40.66 | GFSGLQGPPGPPGSPGEQGPSGASGPAGPR | Deamidated (NQ); 3 Oxidation (P) |
| 902.7532 | 2705.238 | 2705.232 | 52.96 | GFSGLQGPPGPPGSPGEQGPSGASGPAGPR | Deamidated (NQ); 3 Oxidation (P) |
| 902.7541 | 2705.241 | 2705.232 | 65.52 | GFSGLQGPPGPPGSPGEQGPSGASGPAGPR | Deamidated (NQ); 3 Oxidation (P) |
| 1354.113 | 2706.211 | 2706.216 | 42 | GFSGLQGPPGPPGSPGEQGPSGASGPAGPR | 2 Deamidated (NQ); 3 Oxidation (P) |
| 1427.212 | 2852.409 | 2852.405 | 62.65 | GLTGPIGPPGPAGAPGDKGEAGPSGPAGPTGAR | 2 Oxidation (P) |
| 1030.832 | 3089.474 | 3089.469 | 49.32 | GEPGDAGAKGDAGPPGPAGPAGPPGPIGNVGAPGPK | Deamidated (NQ); Oxidation (K); 2 Oxidation (P) |
| 1035.834 | 3104.48 | 3104.48 | 44.68 | GEPGDAGAKGDAGPPGPAGPAGPPGPIGNVGAPGPK | Oxidation (K); 3 Oxidation (P) |
| 1036.165 | 3105.473 | 3105.464 | 41.51 | GEPGDAGAKGDAGPPGPAGPAGPPGPIGNVGAPGPK | Deamidated (NQ); Oxidation (K); 3 Oxidation (P) |
| 1036.165 | 3105.473 | 3105.464 | 40.94 | GEPGDAGAKGDAGPPGPAGPAGPPGPIGNVGAPGPK | Deamidated (NQ); Oxidation (K); 3 Oxidation (P) |
| 1047.833 | 3140.477 | 3140.476 | 59.46 | DGSPGAKGDRGETGPAGPPGAPGAPGAPGPVGPAGK | Oxidation (K); 4 Oxidation (P) |
| 1062.813 | 3185.417 | 3185.413 | 44.31 | GSEGPQGVRGEPGPPGPAGAAGPAGNPGADGQPGAK | 3 Deamidated (NQ); 4 Oxidation (P) |
| 1068.144 | 3201.41 | 3201.408 | 47.54 | GSEGPQGVRGEPGPPGPAGAAGPAGNPGADGQPGAK | 3 Deamidated (NQ); Oxidation (K); 4 Oxidation (P) |
| 1081.472 | 3241.394 | 3241.381 | 40.53 | GANGAPGNDGAKGDAGAPGAPGSQGAPGLQGMPGER | 2 Deamidated (NQ); Oxidation (M); 5 Oxidation (P) |
| 1081.797 | 3242.369 | 3242.365 | 55.91 | GANGAPGNDGAKGDAGAPGAPGSQGAPGLQGMPGER | 3 Deamidated (NQ); Oxidation (M); 5 Oxidation (P) |
| 1081.797 | 3242.369 | 3242.365 | 42.11 | GANGAPGNDGAKGDAGAPGAPGSQGAPGLQGMPGER | 3 Deamidated (NQ); Oxidation (K); Oxidation (M); 4 Oxidation (P) |

| Collagen alpha-2(I) chain OS=Bos taurus GN=COL1A2 PE=1 SV=2 | 3859 |
| --- | --- |

| 426.7379 | 851.4612 | 851.4613 | 44.12 | VGAPGPAGAR | |
| --- | --- | --- | --- | --- | --- |
| 434.7354 | 867.4562 | 867.4563 | 49.96 | GPSGPQGIR | |
| 435.2274 | 868.4402 | 868.4403 | 51.26 | GPSGPQGIR | Deamidated (NQ) |
| 446.7534 | 891.4922 | 891.4926 | 46.62 | PGPIGPAGAR | |
| 459.7265 | 917.4384 | 917.4389 | 45.96 | AGVMGPAGSR | Oxidation (M) |
| 542.7852 | 1083.556 | 1083.556 | 43.44 | GLVGEPGPAGSK | Oxidation (P) |
| 550.7828 | 1099.551 | 1099.551 | 45.64 | GLVGEPGPAGSK | Oxidation (K); Oxidation (P) |
| 591.809 | 1181.603 | 1181.604 | 51.47 | EGPVGLPGIDGR | Oxidation (P) |
| 591.8095 | 1181.604 | 1181.604 | 44.28 | EGPVGLPGIDGR | Oxidation (P) |
| 596.8434 | 1191.672 | 1191.672 | 66.17 | IGQPGAVGPAGIR | |
| 398.2314 | 1191.672 | 1191.672 | 44.38 | IGQPGAVGPAGIR | |
| 596.8438 | 1191.673 | 1191.672 | 49.05 | IGQPGAVGPAGIR | |
| 398.5591 | 1192.656 | 1192.656 | 44.8 | IGQPGAVGPAGIR | Deamidated (NQ) |
| 597.3353 | 1192.656 | 1192.656 | 71.57 | IGQPGAVGPAGIR | Deamidated (NQ) |
| 601.2958 | 1200.577 | 1200.578 | 59.66 | GEPGNIGFPGPK | 2 Oxidation (P) |
| 601.7881 | 1201.562 | 1201.562 | 65.46 | GEPGNIGFPGPK | Deamidated (NQ); 2 Oxidation (P) |
| 601.7888 | 1201.563 | 1201.562 | 40.32 | GEPGNIGFPGPK | Deamidated (NQ); 2 Oxidation (P) |
| 403.563 | 1207.667 | 1207.667 | 41.07 | IGQPGAVGPAGIR | Oxidation (P) |
| 604.8409 | 1207.667 | 1207.667 | 62.57 | IGQPGAVGPAGIR | Oxidation (P) |
| 604.8409 | 1207.667 | 1207.667 | 42.7 | IGQPGAVGPAGIR | Oxidation (P) |
| 604.8411 | 1207.668 | 1207.667 | 60.4 | IGQPGAVGPAGIR | Oxidation (P) |
| 604.8411 | 1207.668 | 1207.667 | 41.32 | IGQPGAVGPAGIR | Oxidation (P) |
| 605.3326 | 1208.651 | 1208.651 | 46.16 | IGQPGAVGPAGIR | Deamidated (NQ); Oxidation (P) |
| 605.3326 | 1208.651 | 1208.651 | 42.85 | IGQPGAVGPAGIR | Deamidated (NQ); Oxidation (P) |
| 605.3327 | 1208.651 | 1208.651 | 49.51 | IGQPGAVGPAGIR | Deamidated (NQ); Oxidation (P) |
| 605.3329 | 1208.651 | 1208.651 | 57.98 | IGQPGAVGPAGIR | Deamidated (NQ); Oxidation (P) |
| 605.333 | 1208.651 | 1208.651 | 46.35 | IGQPGAVGPAGIR | Deamidated (NQ); Oxidation (P) |
| 605.3334 | 1208.652 | 1208.651 | 40.78 | IGQPGAVGPAGIR | Deamidated (NQ); Oxidation (P) |
| 605.3334 | 1208.652 | 1208.651 | 41.26 | IGQPGAVGPAGIR | Deamidated (NQ); Oxidation (P) |
| 611.8082 | 1221.602 | 1221.603 | 47.98 | GFPGTPGLPGFK | Oxidation (K); 2 Oxidation (P) |
| 631.3181 | 1260.622 | 1260.621 | 70.39 | GEAGPAGPAGPAGPR | |
| 634.3406 | 1266.667 | 1266.668 | 47.41 | GIPGPVGAAGATGAR | Oxidation (P) |
| 634.3411 | 1266.668 | 1266.668 | 62.51 | GIPGPVGAAGATGAR | Oxidation (P) |
| 634.3413 | 1266.668 | 1266.668 | 84.11 | GIPGPVGAAGATGAR | Oxidation (P) |
| 634.3413 | 1266.668 | 1266.668 | 59.13 | GIPGPVGAAGATGAR | Oxidation (P) |
| 423.23 | 1266.668 | 1266.668 | 42.76 | GIPGPVGAAGATGAR | Oxidation (P) |
| 644.8117 | 1287.609 | 1287.61 | 72.88 | GFPGSPGNIGPAGK | Deamidated (NQ); 2 Oxidation (P) |
| 644.812 | 1287.609 | 1287.61 | 49.18 | GFPGSPGNIGPAGK | Deamidated (NQ); 2 Oxidation (P) |
| 463.2357 | 1386.685 | 1386.685 | 46.7 | GETGLRGDIGSPGR | Oxidation (P) |
| 694.35 | 1386.685 | 1386.685 | 42.79 | GETGLRGDIGSPGR | Oxidation (P) |
| 714.3668 | 1426.719 | 1426.721 | 58.69 | GIPGEFGLPGPAGAR | 2 Oxidation (P) |
| 714.3672 | 1426.72 | 1426.721 | 48.02 | GIPGEFGLPGPAGAR | 2 Oxidation (P) |
| 714.3672 | 1426.72 | 1426.721 | 48.59 | GIPGEFGLPGPAGAR | 2 Oxidation (P) |
| 714.3672 | 1426.72 | 1426.721 | 41.58 | GIPGEFGLPGPAGAR | 2 Oxidation (P) |
| 714.3676 | 1426.721 | 1426.721 | 49.06 | GIPGEFGLPGPAGAR | 2 Oxidation (P) |
| 714.3679 | 1426.721 | 1426.721 | 64.71 | GIPGEFGLPGPAGAR | 2 Oxidation (P) |
| 714.3679 | 1426.721 | 1426.721 | 44.77 | GIPGEFGLPGPAGAR | 2 Oxidation (P) |
| 714.3681 | 1426.722 | 1426.721 | 70.7 | GIPGEFGLPGPAGAR | 2 Oxidation (P) |
| 714.3681 | 1426.722 | 1426.721 | 44.01 | GIPGEFGLPGPAGAR | 2 Oxidation (P) |
| 714.3689 | 1426.723 | 1426.721 | 47.71 | GIPGEFGLPGPAGAR | 2 Oxidation (P) |
| 729.3412 | 1456.668 | 1456.67 | 46.26 | GDGGPPGATGFPGAAGR | Oxidation (P) |
| 729.3415 | 1456.668 | 1456.67 | 100.25 | GDGGPPGATGFPGAAGR | Oxidation (P) |
| 737.3389 | 1472.663 | 1472.665 | 96.64 | GDGGPPGATGFPGAAGR | 2 Oxidation (P) |
| 737.3391 | 1472.664 | 1472.665 | 59.49 | GDGGPPGATGFPGAAGR | 2 Oxidation (P) |
| 737.34 | 1472.665 | 1472.665 | 56.2 | GDGGPPGATGFPGAAGR | 2 Oxidation (P) |
| 746.8492 | 1491.684 | 1491.684 | 54.02 | SGETGASGPPGFVGEK | Oxidation (P) |
| 746.8495 | 1491.684 | 1491.684 | 60.13 | SGETGASGPPGFVGEK | Oxidation (P) |
| 766.8947 | 1531.775 | 1531.774 | 86.21 | GEPGPAGAVGPAGAVGPR | Oxidation (P) |
| 769.8623 | 1537.71 | 1537.712 | 49.57 | GAPGAIGAPGPAGANGDR | Deamidated (NQ); 2 Oxidation (P) |
| 769.8637 | 1537.713 | 1537.712 | 82.83 | GAPGAIGAPGPAGANGDR | Deamidated (NQ); 2 Oxidation (P) |
| 781.9172 | 1561.82 | 1561.821 | 62.8 | GAAGLPGVAGAPGLPGPR | 3 Oxidation (P) |
| 781.9186 | 1561.823 | 1561.821 | 64.73 | GAAGLPGVAGAPGLPGPR | 3 Oxidation (P) |
| 790.8867 | 1579.759 | 1579.759 | 54.57 | GPPGESGAAGPTGPIGSR | Oxidation (P) |
[truncated: 1,018,104 more chars]
