# Supplementary material for: Proteome degradation in fossils: investigating the longevity of protein survival in ancient bone
Source: Rapid Commun Mass Spectrom. 2014 Feb 12;28(6):605–15. doi: 10.1002/rcm.6821 (PMC4282581; doi:10.1002/rcm.6821)
Supplement: Supplementary file 2 [file rcm0028-0605-SD2.docx]

**Table S2 – Single Protein Matches**

**Collagenase Batch 1**

**KC2**

| Collagen alpha-2(XI) chain OS=Bos taurus GN=COL11A2 PE=3 SV=1 | 56 |
| --- | --- |

| **pep_exp_mz** | **pep_exp_mr** | **pep_calc_mr** | **pep_score** | **pep_seq** | **pep_var_mod** |
| --- | --- | --- | --- | --- | --- |
| 1080.024 | 2158.033 | 2158.037 | 44.91 | AGLPGSDGAPGPPGTSLMLPFR | Oxidation (M); 3 Oxidation (P) |

| Biglycan OS=Bos taurus GN=BGN PE=1 SV=3 | 52 |
| --- | --- |

| 657.3699 | 1312.725 | 1312.724 | 52.39 | IQAIELEDLLR | Deamidated (NQ) |
| --- | --- | --- | --- | --- | --- |

**NS2**

| Complement C3 OS=Bos taurus GN=C3 PE=1 SV=2 | 98 |
| --- | --- |

| **pep_exp_mz** | **pep_exp_mr** | **pep_calc_mr** | **pep_score** | **pep_seq** | **pep_var_mod** |
| --- | --- | --- | --- | --- | --- |
| 634.8663 | 1267.718 | 1267.718 | 53.7 | LVAYYTLINAK | |
| 634.8667 | 1267.719 | 1267.718 | 50.58 | LVAYYTLINAK | |

| Osteomodulin OS=Bos taurus GN=OMD PE=1 SV=1 | 91 |
| --- | --- |

| 606.8159 | 1211.617 | 1211.619 | 44.6 | IFLGYNEISR | Deamidated (NQ) |
| --- | --- | --- | --- | --- | --- |
| 606.8168 | 1211.619 | 1211.619 | 53.81 | IFLGYNEISR | Deamidated (NQ) |
| 606.8168 | 1211.619 | 1211.619 | 54.11 | IFLGYNEISR | Deamidated (NQ) |

| Serine protease HTRA1 OS=Bos taurus GN=HTRA1 PE=2 SV=1 | 77 |
| --- | --- |

| 682.9067 | 1363.799 | 1363.798 | 47.28 | IAPAVVHIELFR |
| --- | --- | --- | --- | --- |
| 682.9067 | 1363.799 | 1363.798 | 51.3 | IAPAVVHIELFR |

| Coagulation factor VII OS=Bos taurus GN=F7 PE=1 SV=2 | 73 |
| --- | --- |

| 725.3782 | 1448.742 | 1448.741 | 72.6 | FSAVSGWGQLLER |
| --- | --- | --- | --- | --- |

| Secreted phosphoprotein 24 OS=Bos taurus GN=SPP2 PE=1 SV=2 | 73 |
| --- | --- |

| 705.8735 | 1409.732 | 1409.73 | 47.13 | VNSQSLSPYLFR |
| --- | --- | --- | --- | --- |
| 705.8737 | 1409.733 | 1409.73 | 54.91 | VNSQSLSPYLFR |

| Chondroadherin OS=Bos taurus GN=CHAD PE=1 SV=1 | 55 |
| --- | --- |

| 876.4464 | 1750.878 | 1750.878 | 46.7 | YLETLWLDNTNLEK |
| --- | --- | --- | --- | --- |

| Thrombospondin-1 OS=Bos taurus GN=THBS1 PE=2 SV=2 | 53 |
| --- | --- |

| 697.8702 | 1393.726 | 1393.724 | 42.96 | FVFGTTPEDILR |
| --- | --- | --- | --- | --- |

| Tetranectin OS=Bos taurus GN=CLEC3B PE=2 SV=1 | 50 |
| --- | --- |

| 542.2864 | 1082.558 | 1082.558 | 45.74 | CFLAFVQAK |
| --- | --- | --- | --- | --- |

| 72 kDa type IV collagenase OS=Bos taurus GN=MMP2 PE=2 SV=1 | 45 |
| --- | --- |

| 709.8754 | 1417.736 | 1417.735 | 45.19 | AFQVWSDVTPLR |
| --- | --- | --- | --- | --- |

**WK1**

| Biglycan OS=Bos taurus GN=BGN PE=1 SV=3 | 63 |
| --- | --- |

| **prot_mass** | **pep_exp_mz** | **pep_exp_mr** | **pep_calc_mr** | **pep_score** | **pep_seq** | **pep_var_mod** |
| --- | --- | --- | --- | --- | --- | --- |
| 41963 | 657.3691 | 1312.724 | 1312.724 | 47.94 | IQAIELEDLLR | Deamidated (NQ) |
| 41963 | 657.3692 | 1312.724 | 1312.724 | 52.99 | IQAIELEDLLR | Deamidated (NQ) |

**AY5**

| Histone H2B type 1-K OS=Bos taurus GN=HIST1H2BK PE=1 SV=3 | 74 |
| --- | --- |

| **pep_exp_mz** | **pep_exp_mr** | **pep_calc_mr** | **pep_score** | **pep_seq** | **pep_var_mod** |
| --- | --- | --- | --- | --- | --- |
| 888.4084 | 1774.802 | 1774.802 | 40.06 | AMGIMNSFVNDIFER | 2 Oxidation (M) |
| 888.4092 | 1774.804 | 1774.802 | 59.41 | AMGIMNSFVNDIFER | 2 Oxidation (M) |

| Histone H2B type 1 OS=Bos taurus PE=1 SV=2 | 74 |
| --- | --- |

| 888.4084 | 1774.802 | 1774.802 | 40.06 | AMGIMNSFVNDIFER | 2 Oxidation (M) |
| --- | --- | --- | --- | --- | --- |
| 888.4092 | 1774.804 | 1774.802 | 59.41 | AMGIMNSFVNDIFER | 2 Oxidation (M) |

**HSB3**

| Histone H2B type 1-K OS=Bos taurus GN=HIST1H2BK PE=1 SV=3 | | | 118 | |  |  |  |
| --- | --- | --- | --- | --- | --- | --- | --- |
| **pep_exp_mz** | **pep_exp_mr** | **pep_calc_mr** | | **pep_score** | | **pep_seq** | **pep_var_mod** |
| 888.4086 | 1774.803 | 1774.802 | | 66.58 | | AMGIMNSFVNDIFER | 2 Oxidation (M) |
| 888.4099 | 1774.805 | 1774.802 | | 77.86 | | AMGIMNSFVNDIFER | 2 Oxidation (M) |

| Histone H2B type 1 OS=Bos taurus PE=1 SV=2 | 118 |
| --- | --- |

| 888.4086 | 1774.803 | 1774.802 | 66.58 | AMGIMNSFVNDIFER | 2 Oxidation (M) |
| --- | --- | --- | --- | --- | --- |
| 888.4099 | 1774.805 | 1774.802 | 77.86 | AMGIMNSFVNDIFER | 2 Oxidation (M) |

| Collagen alpha-2(I) chain OS=Bos taurus GN=COL1A2 PE=1 SV=2 | 89 |
| --- | --- |

| 790.8873 | 1579.76 | 1579.759 | 59.31 | GPPGESGAAGPTGPIGSR |
| --- | --- | --- | --- | --- |
| 790.8877 | 1579.761 | 1579.759 | 59.95 | GPPGESGAAGPTGPIGSR |

| Histone H3.3C OS=Bos taurus GN=H3F3C PE=2 SV=1 | 81 |
| --- | --- |

| 416.2503 | 830.486 | 830.4861 | 56.85 | STELLIR |  |
| --- | --- | --- | --- | --- | --- |
| 416.2503 | 830.486 | 830.4861 | 64.18 | STELLIR |  |

| Histone H3.1 OS=Bos taurus PE=1 SV=2 | 81 |
| --- | --- |

| 416.2503 | 830.486 | 830.4861 | 56.85 | STELLIR |  |
| --- | --- | --- | --- | --- | --- |
| 416.2503 | 830.486 | 830.4861 | 64.18 | STELLIR |  |

| Histone H3.2 OS=Bos taurus PE=1 SV=2 | 81 |
| --- | --- |

| 416.2503 | 830.486 | 830.4861 | 56.85 | STELLIR |  |
| --- | --- | --- | --- | --- | --- |
| 416.2503 | 830.486 | 830.4861 | 64.18 | STELLIR |  |

| Histone H3.3 OS=Bos taurus GN=H3F3A PE=1 SV=3 | 81 |
| --- | --- |

| 416.2503 | 830.486 | 830.4861 | 56.85 | STELLIR |  |
| --- | --- | --- | --- | --- | --- |
| 416.2503 | 830.486 | 830.4861 | 64.18 | STELLIR |  |

| Collagen alpha-1(I) chain OS=Bos taurus GN=COL1A1 PE=1 SV=3 | 57 |
| --- | --- |

| 730.3502 | 1458.686 | 1458.685 | 46.82 | GSAGPPGATGFPGAAGR | 2 Oxidation (P) |
| --- | --- | --- | --- | --- | --- |
| 730.3506 | 1458.687 | 1458.685 | 49.04 | GSAGPPGATGFPGAAGR | 2 Oxidation (P) |

| Histone H2A type 1 OS=Bos taurus PE=1 SV=2 | 40 |
| --- | --- |

| 472.7692 | 943.5238 | 943.524 | 40.07 | AGLQFPVGR |
| --- | --- | --- | --- | --- |

| Histone H2A type 2-C OS=Bos taurus GN=HIST2H2AC PE=2 SV=1 | 40 |
| --- | --- |

| 472.7692 | 943.5238 | 943.524 | 40.07 | AGLQFPVGR |
| --- | --- | --- | --- | --- |

| Histone H2A.J OS=Bos taurus GN=H2AFJ PE=2 SV=1 | 40 |
| --- | --- |

| 472.7692 | 943.5238 | 943.524 | 40.07 | AGLQFPVGR |
| --- | --- | --- | --- | --- |

| Histone H2A.V OS=Bos taurus GN=H2AFV PE=2 SV=3 | 40 |
| --- | --- |

| 472.7692 | 943.5238 | 943.524 | 40.07 | AGLQFPVGR |
| --- | --- | --- | --- | --- |

| Histone H2A.Z OS=Bos taurus GN=H2AFZ PE=1 SV=2 | 40 |
| --- | --- |

| 472.7692 | 943.5238 | 943.524 | 40.07 | AGLQFPVGR |
| --- | --- | --- | --- | --- |

Collagenase Batch 2

| Hemoglobin fetal subunit beta OS=Bos taurus PE=1 SV=1 | 118 |
| --- | --- |

| 422.6143 | 1264.821 | 1264.823 | 44.51 | LLGNVLVVVLAR |
| --- | --- | --- | --- | --- |
| 633.4183 | 1264.822 | 1264.823 | 85.32 | LLGNVLVVVLAR |

| Matrix Gla protein OS=Bos taurus GN=MGP PE=1 SV=1 | 112 |
| --- | --- |

| 778.8464 | 1555.678 | 1555.677 | 53.5 | YAMVYGYNAAYDR | |
| --- | --- | --- | --- | --- | --- |
| 786.8432 | 1571.672 | 1571.671 | 59.14 | YAMVYGYNAAYDR | Oxidation (M) |

| Decorin OS=Bos taurus GN=DCN PE=1 SV=2 | 89 |
| --- | --- |

| 1151.628 | 2301.241 | 2301.24 | 70.35 | KSVFNGLNQMIVVELGTNPLK | Deamidated (NQ) |
| --- | --- | --- | --- | --- | --- |

| Osteomodulin OS=Bos taurus GN=OMD PE=1 SV=1 | 75 |
| --- | --- |

| 606.8169 | 1211.619 | 1211.619 | 49.38 | IFLGYNEISR | Deamidated (NQ) |
| --- | --- | --- | --- | --- | --- |
| 606.817 | 1211.619 | 1211.619 | 45.56 | IFLGYNEISR | Deamidated (NQ) |

| Apolipoprotein A-II OS=Bos taurus GN=APOA2 PE=1 SV=2 | 72 |
| --- | --- |

| 869.4572 | 1736.9 | 1736.899 | 67.4 | AGTDLLNFLSSFIDPK |
| --- | --- | --- | --- | --- |

| 72 kDa type IV collagenase OS=Bos taurus GN=MMP2 PE=2 SV=1 | 58 |
| --- | --- |

| 709.8751 | 1417.736 | 1417.735 | 53.18 | AFQVWSDVTPLR |
| --- | --- | --- | --- | --- |

| Collagen alpha-1(XI) chain (Fragment) OS=Bos taurus GN=COL11A1 PE=1 SV=1 | 55 |
| --- | --- |

| 984.0186 | 1966.023 | 1966.023 | 54.68 | GPTVSAQEAQAQAILQQAR |
| --- | --- | --- | --- | --- |

| Ubiquitin-60S ribosomal protein L40 OS=Bos taurus GN=UBA52 PE=1 SV=2 | 50 |
| --- | --- |

| 534.314 | 1066.613 | 1066.614 | 50.42 | ESTLHLVLR |
| --- | --- | --- | --- | --- |

| Ubiquitin-40S ribosomal protein S27a OS=Bos taurus GN=RPS27A PE=1 SV=2 | 50 |
| --- | --- |

| 534.314 | 1066.613 | 1066.614 | 50.42 | ESTLHLVLR |
| --- | --- | --- | --- | --- |

| Polyubiquitin-B OS=Bos taurus GN=UBB PE=1 SV=1 | 50 |
| --- | --- |

| 534.314 | 1066.613 | 1066.614 | 50.42 | ESTLHLVLR |
| --- | --- | --- | --- | --- |

| Polyubiquitin-C OS=Bos taurus GN=UBC PE=1 SV=1 | 50 |
| --- | --- |

| 534.314 | 1066.613 | 1066.614 | 50.42 | ESTLHLVLR |
| --- | --- | --- | --- | --- |

| Vitamin K-dependent protein C (Fragment) OS=Bos taurus GN=PROC PE=1 SV=1 | 47 |
| --- | --- |

| 823.4048 | 1644.795 | 1644.796 | 47.47 | ISENMLCAGILGDPR |
| --- | --- | --- | --- | --- |

| Unconventional myosin-Id OS=Bos taurus GN=MYO1D PE=2 SV=1 | 45 |
| --- | --- |

| 540.7867 | 1079.559 | 1079.559 | 45.28 | LFCWIVSR |
| --- | --- | --- | --- | --- |

| Complement component C9 OS=Bos taurus GN=C9 PE=2 SV=1 | 45 |
| --- | --- |

| 728.3602 | 1454.706 | 1454.704 | 41.62 | AIEDYINEFSVR |
| --- | --- | --- | --- | --- |

| 45 kDa calcium-binding protein OS=Bos taurus GN=SDF4 PE=2 SV=1 | 44 |
| --- | --- |

| 477.2507 | 952.4868 | 952.4874 | 44.36 | GMLQFMVK |
| --- | --- | --- | --- | --- |

| Complement factor B OS=Bos taurus GN=CFB PE=1 SV=2 | 43 |
| --- | --- |

| 663.8312 | 1325.648 | 1325.647 | 43.03 | ALLEVYNMMSR |
| --- | --- | --- | --- | --- |

| Hyaluronan and proteoglycan link protein 1 OS=Bos taurus GN=HAPLN1 PE=2 SV=1 | 42 |
| --- | --- |

| 510.2865 | 1018.558 | 1018.56 | 41.81 | VGQIFAAWK |
| --- | --- | --- | --- | --- |

| Macrophage migration inhibitory factor OS=Bos taurus GN=MIF PE=1 SV=6 | 41 |
| --- | --- |

| 537.8024 | 1073.59 | 1073.59 | 41.09 | LLCGLLTER |
| --- | --- | --- | --- | --- |

| Lactadherin OS=Bos taurus GN=MFGE8 PE=1 SV=2 | 41 |
| --- | --- |

| 854.9497 | 1707.885 | 1707.883 | 40.91 | INLFDTPLETQYVR |
| --- | --- | --- | --- | --- |

| Actin, cytoplasmic 1 OS=Bos taurus GN=ACTB PE=1 SV=1 | 40 |
| --- | --- |

| 895.9513 | 1789.888 | 1789.885 | 43.43 | SYELPDGQVITIGNER |
| --- | --- | --- | --- | --- |

| Actin, cytoplasmic 2 OS=Bos taurus GN=ACTG1 PE=1 SV=1 | 40 |
| --- | --- |

| 895.9513 | 1789.888 | 1789.885 | 43.43 | SYELPDGQVITIGNER |
| --- | --- | --- | --- | --- |

| Actin, aortic smooth muscle OS=Bos taurus GN=ACTA2 PE=1 SV=1 | 40 |
| --- | --- |

| 895.9513 | 1789.888 | 1789.885 | 43.43 | SYELPDGQVITIGNER |
| --- | --- | --- | --- | --- |

| Actin, alpha cardiac muscle 1 OS=Bos taurus GN=ACTC1 PE=2 SV=1 | 40 |
| --- | --- |

| 895.9513 | 1789.888 | 1789.885 | 43.43 | SYELPDGQVITIGNER |
| --- | --- | --- | --- | --- |

| Actin, gamma-enteric smooth muscle OS=Bos taurus GN=ACTG2 PE=2 SV=1 | 40 |
| --- | --- |

| 895.9513 | 1789.888 | 1789.885 | 43.43 | SYELPDGQVITIGNER |
| --- | --- | --- | --- | --- |

| Actin, alpha skeletal muscle OS=Bos taurus GN=ACTA1 PE=1 SV=1 | 40 |
| --- | --- |

| 895.9513 | 1789.888 | 1789.885 | 43.43 | SYELPDGQVITIGNER |
| --- | --- | --- | --- | --- |

| Transforming growth factor beta-1 OS=Bos taurus GN=TGFB1 PE=1 SV=2 | 40 |
| --- | --- |

| 594.8237 | 1187.633 | 1187.633 | 42.15 | VEQLSNMIVR |
| --- | --- | --- | --- | --- |

| Coagulation factor IX (Fragment) OS=Bos taurus GN=F9 PE=1 SV=1 | 40 |
| --- | --- |

| 610.7906 | 1219.567 | 1219.566 | 44.99 | FGYGYVSGWGK |
| --- | --- | --- | --- | --- |

**KC2**

| Serum albumin OS=Bos taurus GN=ALB PE=1 SV=4 | 70 |
| --- | --- |

| **pep_exp_mz** | **pep_exp_mr** | **pep_calc_mr** | **pep_score** | **pep_seq** | **pep_var_mod** |
| --- | --- | --- | --- | --- | --- |
| 740.4021 | 1478.79 | 1478.788 | 46.89 | LGEYGFQNALIVR | |

**KC6**

| Collagen alpha-1(III) chain OS=Bos taurus GN=COL3A1 PE=1 SV=1 | 84 |
| --- | --- |

| **pep_exp_mz** | **pep_exp_mr** | **pep_calc_mr** | **pep_score** | **pep_seq** | **pep_var_mod** |
| --- | --- | --- | --- | --- | --- |
| 637.2999 | 1272.585 | 1272.585 | 64.46 | GSPGGPGAAGFPGGR | 2 Oxidation (P) |
| 645.2975 | 1288.58 | 1288.58 | 46.88 | GSPGGPGAAGFPGGR | 3 Oxidation (P) |

| Complement C3 OS=Bos taurus GN=C3 PE=1 SV=2 | 76 |
| --- | --- |

| 941.0293 | 1880.044 | 1880.041 | 75.78 | LYNVEATSYALLALLAR |
| --- | --- | --- | --- | --- |

| Hemoglobin subunit beta OS=Bos taurus GN=HBB PE=1 SV=1 | 55 |
| --- | --- |

| 633.4194 | 1264.824 | 1264.823 | 55.26 | LLGNVLVVVLAR |
| --- | --- | --- | --- | --- |

| Pigment epithelium-derived factor OS=Bos taurus GN=SERPINF1 PE=1 SV=1 | 49 |
| --- | --- |

| 1037.603 | 2073.191 | 2073.187 | 40.22 | IAQLPLTGSTSIIFFLPQK |
| --- | --- | --- | --- | --- |

| Lumican OS=Bos taurus GN=LUM PE=1 SV=1 | 40 |
| --- | --- |

| 499.2767 | 996.5388 | 996.5392 | 40.82 | FSALQYLR |
| --- | --- | --- | --- | --- |

| SPARC OS=Bos taurus GN=SPARC PE=1 SV=2 | 27 |
| --- | --- |

| 821.9011 | 1641.788 | 1641.786 | 41.06 | YIALDEWAGCFGIK |
| --- | --- | --- | --- | --- |

**NS2**

| Matrix Gla protein OS=Bos taurus GN=MGP PE=1 SV=1 | 71 |
| --- | --- |

| **pep_exp_mz** | **pep_exp_mr** | **pep_calc_mr** | **pep_score** | **pep_seq** | **pep_var_mod** |
| --- | --- | --- | --- | --- | --- |
| 779.338 | 1556.661 | 1556.661 | 57.3 | YAMVYGYNAAYDR | Deamidated (NQ) |

| Collagen alpha-2(XI) chain OS=Bos taurus GN=COL11A2 PE=3 SV=1 | 70 |
| --- | --- |

| 511.2875 | 1020.56 | 1020.56 | 62.49 | DFSLLTAVR |
| --- | --- | --- | --- | --- |

| Lumican OS=Bos taurus GN=LUM PE=1 SV=1 | 62 |
| --- | --- |

| 499.2768 | 996.539 | 996.5392 | 49.09 | FSALQYLR | |
| --- | --- | --- | --- | --- | --- |
| 499.2772 | 996.5398 | 996.5392 | 43.82 | FSALQYLR | |
| 499.769 | 997.5234 | 997.5233 | 44.46 | FSALQYLR | Deamidated (NQ) |

| Tetranectin OS=Bos taurus GN=CLEC3B PE=2 SV=1 | 60 |
| --- | --- |

| 765.424 | 1528.833 | 1528.835 | 44.13 | TQLDSLAQEVALLK | Deamidated (NQ) |
| --- | --- | --- | --- | --- | --- |

| Olfactomedin-like protein 3 OS=Bos taurus GN=OLFML3 PE=2 SV=1 | 58 |
| --- | --- |

| 517.277 | 1032.539 | 1032.539 | 57.75 | FGGPAGLWTK |
| --- | --- | --- | --- | --- |

| Collagen alpha-1(XI) chain (Fragment) OS=Bos taurus GN=COL11A1 PE=1 SV=1 | 58 |
| --- | --- |

| 984.0199 | 1966.025 | 1966.023 | 57.56 | GPTVSAQEAQAQAILQQAR |
| --- | --- | --- | --- | --- |

| Vitamin K-dependent protein S OS=Bos taurus GN=PROS1 PE=1 SV=1 | 55 |
| --- | --- |

| 536.7661 | 1071.518 | 1071.517 | 48.43 | YLGCLGSFR |
| --- | --- | --- | --- | --- |

| Coagulation factor VII OS=Bos taurus GN=F7 PE=1 SV=2 | 53 |
| --- | --- |

| 725.3785 | 1448.742 | 1448.741 | 53.01 | FSAVSGWGQLLER |
| --- | --- | --- | --- | --- |

| Transforming growth factor beta-1 OS=Bos taurus GN=TGFB1 PE=1 SV=2 | 53 |
| --- | --- |

| 708.8997 | 1415.785 | 1415.785 | 52.76 | PFLLLMATPLER | Oxidation (M) |
| --- | --- | --- | --- | --- | --- |

| SPARC OS=Bos taurus GN=SPARC PE=1 SV=2 | 49 |
| --- | --- |

| 553.814 | 1105.613 | 1105.613 | 41.44 | NVLVTLYER |
| --- | --- | --- | --- | --- |

| Secreted phosphoprotein 24 OS=Bos taurus GN=SPP2 PE=1 SV=2 | 49 |
| --- | --- |

| 706.3652 | 1410.716 | 1410.714 | 50.8 | VNSQSLSPYLFR | Deamidated (NQ) |
| --- | --- | --- | --- | --- | --- |

| Complement component C9 OS=Bos taurus GN=C9 PE=2 SV=1 | 48 |
| --- | --- |

| 728.3606 | 1454.707 | 1454.704 | 44.66 | AIEDYINEFSVR |
| --- | --- | --- | --- | --- |

| Coagulation factor IX (Fragment) OS=Bos taurus GN=F9 PE=1 SV=1 | 45 |
| --- | --- |

| 511.798 | 1021.581 | 1021.581 | 45.33 | SASILQYLK |
| --- | --- | --- | --- | --- |

| Dermatopontin OS=Bos taurus GN=DPT PE=1 SV=3 | 44 |
| --- | --- |

| 514.7562 | 1027.498 | 1027.498 | 43.6 | YFESVLDR |
| --- | --- | --- | --- | --- |

| Thrombospondin-1 OS=Bos taurus GN=THBS1 PE=2 SV=2 | 41 |
| --- | --- |

| 531.7827 | 1061.551 | 1061.551 | 47.65 | FQDLVDAVR |
| --- | --- | --- | --- | --- |

**AY5**

| Alpha-2-HS-glycoprotein OS=Bos taurus GN=AHSG PE=1 SV=2 | 74 |
| --- | --- |

| **pep_exp_mz** | **pep_exp_mr** | **pep_calc_mr** | **pep_score** | **pep_seq** | **pep_var_mod** | **pep_var_mod_pos** |
| --- | --- | --- | --- | --- | --- | --- |
| 1260.167 | 2518.319 | 2518.314 | 67.04 | AQFVPLPVSVSVEFAVAATDCIAK | | |

| Pigment epithelium-derived factor OS=Bos taurus GN=SERPINF1 PE=1 SV=1 | 69 |
| --- | --- |

| 1037.602 | 2073.189 | 2073.187 | 53.19 | IAQLPLTGSTSIIFFLPQK |
| --- | --- | --- | --- | --- |

| Hemoglobin fetal subunit beta OS=Bos taurus PE=1 SV=1 | 52 |
| --- | --- |

| 633.4193 | 1264.824 | 1264.823 | 51.91 | LLGNVLVVVLAR |
| --- | --- | --- | --- | --- |

| Hemoglobin subunit beta OS=Bos taurus GN=HBB PE=1 SV=1 | 52 |
| --- | --- |

| 633.4193 | 1264.824 | 1264.823 | 51.91 | LLGNVLVVVLAR |
| --- | --- | --- | --- | --- |

| Lumican OS=Bos taurus GN=LUM PE=1 SV=1 | 45 |
| --- | --- |

| 499.2768 | 996.539 | 996.5392 | 44.81 | FSALQYLR |
| --- | --- | --- | --- | --- |

| SPARC OS=Bos taurus GN=SPARC PE=1 SV=2 | 45 |
| --- | --- |

| 821.9017 | 1641.789 | 1641.786 | 44.58 | YIALDEWAGCFGIK |
| --- | --- | --- | --- | --- |

**HSB3**

| Serum albumin OS=Bos taurus GN=ALB PE=1 SV=4 | 53 |
| --- | --- |

| **pep_exp_mz** | **pep_exp_mr** | **pep_calc_mr** | **pep_score** | **pep_seq** | **pep_var_mod** |
| --- | --- | --- | --- | --- | --- |
| 740.4014 | 1478.788 | 1478.788 | 45.79 | LGEYGFQNALIVR | |

| Serotransferrin OS=Bos taurus GN=TF PE=2 SV=1 | 52 |
| --- | --- |

| 923.9305 | 1845.846 | 1845.846 | 52.31 | GEADAMSLDGGYLYIAGK | Oxidation (M) |
| --- | --- | --- | --- | --- | --- |

| Collagen alpha-2(I) chain OS=Bos taurus GN=COL1A2 PE=1 SV=2 | 50 |
| --- | --- |

| 605.3331 | 1208.652 | 1208.651 | 50.1 | IGQPGAVGPAGIR | Deamidated (NQ); Oxidation (P) |
| --- | --- | --- | --- | --- | --- |

| SPARC OS=Bos taurus GN=SPARC PE=1 SV=2 | 42 |
| --- | --- |

| 821.9012 | 1641.788 | 1641.786 | 41.78 | YIALDEWAGCFGIK |
| --- | --- | --- | --- | --- |

**TSH**

| Cytochrome c OS=Bos taurus GN=CYCS PE=1 SV=2 | 43 |
| --- | --- |

| **prot_mass** | **pep_exp_mz** | **pep_exp_mr** | **pep_calc_mr** | **pep_score** | **pep_seq** | **pep_var_mod** |
| --- | --- | --- | --- | --- | --- | --- |
| 11810 | 584.8148 | 1167.615 | 1167.615 | 43.29 | TGPNLHGLFGR | |

**GuHCl Batch 1**

**NF1**

| Matrix Gla protein OS=Bos taurus GN=MGP PE=1 SV=1 | 188 |
| --- | --- |

| **pep_exp_mz** | **pep_exp_mr** | **pep_calc_mr** | **pep_score** | **pep_seq** | **pep_var_mod** |
| --- | --- | --- | --- | --- | --- |
| 779.3373 | 1556.66 | 1556.661 | 52.2 | YAMVYGYNAAYDR | Deamidated (NQ) |
| 779.3377 | 1556.661 | 1556.661 | 63.97 | YAMVYGYNAAYDR | Deamidated (NQ) |
| 787.3344 | 1572.654 | 1572.656 | 63.71 | YAMVYGYNAAYDR | Deamidated (NQ); Oxidation (M) |
| 787.3347 | 1572.655 | 1572.656 | 77.36 | YAMVYGYNAAYDR | Deamidated (NQ); Oxidation (M) |

| Secreted phosphoprotein 24 OS=Bos taurus GN=SPP2 PE=1 SV=2 | 138 |
| --- | --- |

| 705.8727 | 1409.731 | 1409.73 | 54.24 | VNSQSLSPYLFR | |
| --- | --- | --- | --- | --- | --- |
| 706.3643 | 1410.714 | 1410.714 | 70.15 | VNSQSLSPYLFR | Deamidated (NQ) |
| 706.8571 | 1411.7 | 1411.698 | 56.82 | VNSQSLSPYLFR | 2 Deamidated (NQ) |

| Apolipoprotein A-II OS=Bos taurus GN=APOA2 PE=1 SV=2 | 132 |
| --- | --- |

| 869.4564 | 1736.898 | 1736.899 | 81.27 | AGTDLLNFLSSFIDPK |
| --- | --- | --- | --- | --- |
| 869.457 | 1736.899 | 1736.899 | 87.66 | AGTDLLNFLSSFIDPK |

| Tubulin beta-4A chain OS=Bos taurus GN=TUBB4A PE=2 SV=1 | 77 |
| --- | --- |

| 520.3004 | 1038.586 | 1038.586 | 76.54 | YLTVAAVFR |
| --- | --- | --- | --- | --- |

| Tubulin beta-4B chain OS=Bos taurus GN=TUBB4B PE=2 SV=1 | 77 |
| --- | --- |

| 520.3004 | 1038.586 | 1038.586 | 76.54 | YLTVAAVFR |
| --- | --- | --- | --- | --- |

| Tubulin beta-5 chain OS=Bos taurus GN=TUBB5 PE=2 SV=1 | 77 |
| --- | --- |

| 520.3004 | 1038.586 | 1038.586 | 76.54 | YLTVAAVFR |
| --- | --- | --- | --- | --- |

| Tubulin beta-6 chain OS=Bos taurus GN=TUBB6 PE=2 SV=1 | 77 |
| --- | --- |

| 520.3004 | 1038.586 | 1038.586 | 76.54 | YLTVAAVFR |
| --- | --- | --- | --- | --- |

| Complement C1q subcomponent subunit B OS=Bos taurus GN=C1QB PE=1 SV=1 | 75 |
| --- | --- |

| 822.913 | 1643.811 | 1643.81 | 63.49 | VPGLYFFTYHASSR |
| --- | --- | --- | --- | --- |

| Coagulation factor VII OS=Bos taurus GN=F7 PE=1 SV=2 | 72 |
| --- | --- |

| 725.3781 | 1448.742 | 1448.741 | 69.7 | FSAVSGWGQLLER |
| --- | --- | --- | --- | --- |

| Bone sialoprotein 2 OS=Bos taurus GN=IBSP PE=1 SV=1 | 64 |
| --- | --- |

| 548.2844 | 1094.554 | 1094.555 | 47.46 | HGYFYPALK |
| --- | --- | --- | --- | --- |

| Fibromodulin OS=Bos taurus GN=FMOD PE=1 SV=2 | 61 |
| --- | --- |

| 779.4366 | 1556.859 | 1556.856 | 46.03 | SLILLDLSYNHLR | Deamidated (NQ) |
| --- | --- | --- | --- | --- | --- |

| Hyaluronan and proteoglycan link protein 1 OS=Bos taurus GN=HAPLN1 PE=2 SV=1 | 55 |
| --- | --- |

| 510.287 | 1018.559 | 1018.56 | 45.56 | VGQIFAAWK |
| --- | --- | --- | --- | --- |
| 510.2871 | 1018.56 | 1018.56 | 45.21 | VGQIFAAWK |

| Platelet glycoprotein 4 OS=Bos taurus GN=CD36 PE=1 SV=5 | 54 |
| --- | --- |

| 686.3403 | 1370.666 | 1370.665 | 54.28 | VLQFFSSDICR |
| --- | --- | --- | --- | --- |

| Alpha-1-antiproteinase OS=Bos taurus GN=SERPINA1 PE=1 SV=1 | 52 |
| --- | --- |

| 970.5278 | 1939.041 | 1939.046 | 52.22 | VLDPNTVFALVNYISFK |
| --- | --- | --- | --- | --- |

| Lysyl oxidase homolog 4 OS=Bos taurus GN=LOXL4 PE=2 SV=1 | 49 |
| --- | --- |

| 736.0704 | 2205.189 | 2205.191 | 49.27 | EALFGAQLGQALGPIHLSEVR |
| --- | --- | --- | --- | --- |

| Kininogen-1 OS=Bos taurus GN=KNG1 PE=1 SV=1 | 48 |
| --- | --- |

| 484.7817 | 967.5488 | 967.5491 | 48.3 | YSIVFIAR |  |
| --- | --- | --- | --- | --- | --- |

| Vitamin K-dependent protein C (Fragment) OS=Bos taurus GN=PROC PE=1 SV=1 | 46 |
| --- | --- |

| 477.7861 | 953.5576 | 953.5586 | 40.63 | TFVLSFIK |  |
| --- | --- | --- | --- | --- | --- |

| Lysozyme C, milk isozyme OS=Bos taurus PE=2 SV=1 | 46 |
| --- | --- |

| 673.3562 | 1344.698 | 1344.697 | 45.83 | GVSLANWVCLAR |
| --- | --- | --- | --- | --- |

| Serotransferrin OS=Bos taurus GN=TF PE=2 SV=1 | 44 |
| --- | --- |

| 460.2841 | 918.5536 | 918.5538 | 44.12 | GYLAVAVVK |
| --- | --- | --- | --- | --- |

| Mammalian ependymin-related protein 1 OS=Bos taurus GN=EPDR1 PE=2 SV=1 | 43 |
| --- | --- |

| 601.347 | 1200.679 | 1200.679 | 43.49 | LFEYILLYK |  |
| --- | --- | --- | --- | --- | --- |

| Fibrinogen beta chain OS=Bos taurus GN=FGB PE=1 SV=2 | 43 |
| --- | --- |

| 744.9003 | 1487.786 | 1487.787 | 42.83 | TSSSTFQYITLLK |
| --- | --- | --- | --- | --- |

| Fibronectin OS=Bos taurus GN=FN1 PE=1 SV=4 | 42 |
| --- | --- |

| 643.0189 | 1926.035 | 1926.036 | 42.43 | FLATTPNSLLVSWQPPR |
| --- | --- | --- | --- | --- |

| Transforming growth factor beta-1 OS=Bos taurus GN=TGFB1 PE=1 SV=2 | 42 |
| --- | --- |

| 926.4212 | 2776.242 | 2776.241 | 41.68 | GYHANFCLGPCPYIWSLDTQYSK |
| --- | --- | --- | --- | --- |

| Matrix Gla protein OS=Bos taurus GN=MGP PE=1 SV=1 | 173 |
| --- | --- |

| **pep_exp_mz** | **pep_exp_mr** | **pep_calc_mr** | **pep_score** | **pep_seq** | **pep_var_mod** |
| --- | --- | --- | --- | --- | --- |
| 778.8455 | 1555.676 | 1555.677 | 63.92 | YAMVYGYNAAYDR | |
| 779.338 | 1556.661 | 1556.661 | 55.06 | YAMVYGYNAAYDR | Deamidated (NQ) |
| 787.3354 | 1572.656 | 1572.656 | 65.05 | YAMVYGYNAAYDR | Deamidated (NQ); Oxidation (M) |
| 787.3359 | 1572.657 | 1572.656 | 52.57 | YAMVYGYNAAYDR | Deamidated (NQ); Oxidation (M) |

| Secreted phosphoprotein 24 OS=Bos taurus GN=SPP2 PE=1 SV=2 | 165 |
| --- | --- |

| 705.8717 | 1409.729 | 1409.73 | 54.9 | VNSQSLSPYLFR | |
| --- | --- | --- | --- | --- | --- |
| 706.3646 | 1410.715 | 1410.714 | 65.77 | VNSQSLSPYLFR | Deamidated (NQ) |
| 706.8567 | 1411.699 | 1411.698 | 45.54 | VNSQSLSPYLFR | 2 Deamidated (NQ) |

| Apolipoprotein A-II OS=Bos taurus GN=APOA2 PE=1 SV=2 | 120 |
| --- | --- |

| 869.4564 | 1736.898 | 1736.899 | 64.33 | AGTDLLNFLSSFIDPK | |
| --- | --- | --- | --- | --- | --- |
| 869.949 | 1737.883 | 1737.883 | 77.16 | AGTDLLNFLSSFIDPK | Deamidated (NQ) |

| Asporin OS=Bos taurus GN=ASPN PE=2 SV=1 | 106 |
| --- | --- |

| 838.973 | 1675.931 | 1675.93 | 57.47 | YLQIIFLHSNSITK | |
| --- | --- | --- | --- | --- | --- |
| 839.4636 | 1676.913 | 1676.914 | 81.15 | YLQIIFLHSNSITK | Deamidated (NQ) |

| Antithrombin-III OS=Bos taurus GN=SERPINC1 PE=1 SV=2 | 71 |
| --- | --- |

| 682.3737 | 1362.733 | 1362.733 | 71.32 | EVALNTIIFMGR |
| --- | --- | --- | --- | --- |

| Osteomodulin OS=Bos taurus GN=OMD PE=1 SV=1 | 82 |
| --- | --- |

| 606.8166 | 1211.619 | 1211.619 | 47.77 | IFLGYNEISR | Deamidated (NQ) |
| --- | --- | --- | --- | --- | --- |
| 606.8168 | 1211.619 | 1211.619 | 54.39 | IFLGYNEISR | Deamidated (NQ) |

| Vitamin D-binding protein OS=Bos taurus GN=GC PE=2 SV=1 | 74 |
| --- | --- |

| 691.8694 | 1381.724 | 1381.724 | 73.65 | VLDQYIFELSR |
| --- | --- | --- | --- | --- |

| Collagen alpha-1(XI) chain (Fragment) OS=Bos taurus GN=COL11A1 PE=1 SV=1 | 71 |
| --- | --- |

| 763.0495 | 2286.127 | 2286.124 | 43.25 | TGPPGPGGVVGPQGPTGETGPIGER | Oxidation (P) |
| --- | --- | --- | --- | --- | --- |

| Bone sialoprotein 2 OS=Bos taurus GN=IBSP PE=1 SV=1 | 70 |
| --- | --- |

| 548.2849 | 1094.555 | 1094.555 | 44.44 | HGYFYPALK |
| --- | --- | --- | --- | --- |

| Clusterin OS=Bos taurus GN=CLU PE=1 SV=1 | 69 |
| --- | --- |

| 763.8955 | 1525.776 | 1525.778 | 69.08 | LYDQLLQSYQQK |
| --- | --- | --- | --- | --- |

| 72 kDa type IV collagenase OS=Bos taurus GN=MMP2 PE=2 SV=1 | 65 |
| --- | --- |

| 710.3662 | 1418.718 | 1418.719 | 61.77 | AFQVWSDVTPLR | Deamidated (NQ) |
| --- | --- | --- | --- | --- | --- |

| DnaJ homolog subfamily C member 3 OS=Bos taurus GN=DNAJC3 PE=1 SV=1 | 59 |
| --- | --- |

| 469.2621 | 936.5096 | 936.5103 | 59.23 | ATVFLAMGK |
| --- | --- | --- | --- | --- |

| Versican core protein OS=Bos taurus GN=VCAN PE=1 SV=2 | 58 |
| --- | --- |

| 657.862 | 1313.709 | 1313.709 | 58.05 | LATVGELQAAWR |
| --- | --- | --- | --- | --- |

| Vitamin K-dependent protein C (Fragment) OS=Bos taurus GN=PROC PE=1 SV=1 | 57 |
| --- | --- |

| 654.343 | 1306.671 | 1306.671 | 47.45 | YLDWIYGHIK |
| --- | --- | --- | --- | --- |

| Gelsolin OS=Bos taurus GN=GSN PE=2 SV=1 | 56 |
| --- | --- |

| 436.7631 | 871.5116 | 871.5127 | 55.85 | TGALELLR |  |
| --- | --- | --- | --- | --- | --- |

| Dermatopontin OS=Bos taurus GN=DPT PE=1 SV=3 | 55 |
| --- | --- |

| 514.756 | 1027.497 | 1027.498 | 43.55 | YFESVLDR |
| --- | --- | --- | --- | --- |

| Moesin OS=Bos taurus GN=MSN PE=2 SV=3 | 53 |
| --- | --- |

| 591.8007 | 1181.587 | 1181.587 | 46.67 | APDFVFYAPR |
| --- | --- | --- | --- | --- |

| Ezrin OS=Bos taurus GN=EZR PE=1 SV=2 | 53 |
| --- | --- |

| 591.8007 | 1181.587 | 1181.587 | 46.67 | APDFVFYAPR |
| --- | --- | --- | --- | --- |

| Radixin OS=Bos taurus GN=RDX PE=2 SV=1 | 53 |
| --- | --- |

| 591.8007 | 1181.587 | 1181.587 | 46.67 | APDFVFYAPR |
| --- | --- | --- | --- | --- |

| Mimecan OS=Bos taurus GN=OGN PE=1 SV=2 | 49 |
| --- | --- |

| 520.3111 | 1038.608 | 1038.607 | 49.25 | LEGNPVILGK |
| --- | --- | --- | --- | --- |

| Vitamin K-dependent protein S OS=Bos taurus GN=PROS1 PE=1 SV=1 | 46 |
| --- | --- |

| 509.7346 | 1017.455 | 1017.456 | 44.73 | FSAEFDFR |
| --- | --- | --- | --- | --- |

| Complement factor D OS=Bos taurus GN=CFD PE=2 SV=1 | 44 |
| --- | --- |

| 774.4565 | 1546.898 | 1546.898 | 44.4 | AVLGPAVQLLPWQR |
| --- | --- | --- | --- | --- |

| Vitrin OS=Bos taurus GN=VIT PE=2 SV=2 | 43 |
| --- | --- |

| 516.2991 | 1030.584 | 1030.585 | 44.3 | LMILITDGR |
| --- | --- | --- | --- | --- |

| Coagulation factor X OS=Bos taurus GN=F10 PE=1 SV=1 | 42 |
| --- | --- |

| 447.2454 | 892.4762 | 892.4767 | 42.49 | TGIVSGFGR |
| --- | --- | --- | --- | --- |

| Eukaryotic initiation factor 4A-II OS=Bos taurus GN=EIF4A2 PE=2 SV=1 | 42 |
| --- | --- |

| 557.845 | 1113.675 | 1113.676 | 42.36 | VLITTDLLAR |
| --- | --- | --- | --- | --- |

| Eukaryotic initiation factor 4A-I OS=Bos taurus GN=EIF4A1 PE=2 SV=1 | 42 |
| --- | --- |

| 557.845 | 1113.675 | 1113.676 | 42.36 | VLITTDLLAR |
| --- | --- | --- | --- | --- |

| Peptidyl-prolyl cis-trans isomerase B OS=Bos taurus GN=PPIB PE=1 SV=4 | 42 |
| --- | --- |

| 416.7682 | 831.5218 | 831.5218 | 41.71 | VVIGLFGK |
| --- | --- | --- | --- | --- |

| Integrin beta-1 OS=Bos taurus GN=ITGB1 PE=1 SV=3 | 41 |
| --- | --- |

| 492.2638 | 982.513 | 982.5124 | 41.34 | IGFGSFVEK |
| --- | --- | --- | --- | --- |

| Insulin-like growth factor-binding protein 3 OS=Bos taurus GN=IGFBP3 PE=1 SV=3 | 41 |
| --- | --- |

| 489.265 | 976.5154 | 976.5164 | 41.3 | FLNMLSPR |
| --- | --- | --- | --- | --- |

| Protein AMBP OS=Bos taurus GN=AMBP PE=1 SV=2 | 40 |
| --- | --- |

| 511.7615 | 1021.508 | 1021.508 | 40.37 | ESLLEEFR |  |
| --- | --- | --- | --- | --- | --- |

**KC6**

| Serum albumin OS=Bos taurus GN=ALB PE=1 SV=4 | 111 |
| --- | --- |

| **pep_exp_mz** | **pep_exp_mr** | **pep_calc_mr** | **pep_score** | **pep_seq** | **pep_var_mod** |
| --- | --- | --- | --- | --- | --- |
| 740.402 | 1478.789 | 1478.788 | 65.97 | LGEYGFQNALIVR | |
| 740.8945 | 1479.774 | 1479.772 | 72.26 | LGEYGFQNALIVR | Deamidated (NQ) |

| Lumican OS=Bos taurus GN=LUM PE=1 SV=1 | 58 |
| --- | --- |

| 499.2766 | 996.5386 | 996.5392 | 54.22 | FSALQYLR | |
| --- | --- | --- | --- | --- | --- |
| 499.768 | 997.5214 | 997.5233 | 41.32 | FSALQYLR | Deamidated (NQ) |

| Decorin OS=Bos taurus GN=DCN PE=1 SV=2 | 40 |
| --- | --- |

| 550.3284 | 1098.642 | 1098.644 | 40.22 | ISPGAFAPLVK |
| --- | --- | --- | --- | --- |

**NS2**

| Matrix Gla protein OS=Bos taurus GN=MGP PE=1 SV=1 | 200 |
| --- | --- |

| **pep_exp_mz** | **pep_exp_mr** | **pep_calc_mr** | **pep_score** | **pep_seq** | **pep_var_mod** |
| --- | --- | --- | --- | --- | --- |
| 778.8452 | 1555.676 | 1555.677 | 62.26 | YAMVYGYNAAYDR | |
| 778.8455 | 1555.676 | 1555.677 | 52.92 | YAMVYGYNAAYDR | |
| 779.3377 | 1556.661 | 1556.661 | 56.78 | YAMVYGYNAAYDR | Deamidated (NQ) |
| 786.8424 | 1571.67 | 1571.671 | 65.86 | YAMVYGYNAAYDR | Oxidation (M) |
| 787.3363 | 1572.658 | 1572.656 | 54.27 | YAMVYGYNAAYDR | Deamidated (NQ); Oxidation (M) |

| Extracellular matrix protein 2 OS=Bos taurus GN=ECM2 PE=2 SV=1 | 79 |
| --- | --- |

| 530.983 | 1589.927 | 1589.925 | 58.9 | SLVHLVLIGNQIER |
| --- | --- | --- | --- | --- |

| Serpin H1 OS=Bos taurus GN=SERPINH1 PE=2 SV=1 | 79 |
| --- | --- |

| 837.4138 | 1672.813 | 1672.81 | 78.88 | LYGPSSVSFAEDFVR |
| --- | --- | --- | --- | --- |

| Apolipoprotein A-II OS=Bos taurus GN=APOA2 PE=1 SV=2 | 71 |
| --- | --- |

| 869.949 | 1737.883 | 1737.883 | 70.92 | AGTDLLNFLSSFIDPK | Deamidated (NQ) |
| --- | --- | --- | --- | --- | --- |

| Clusterin OS=Bos taurus GN=CLU PE=1 SV=1 | 69 |
| --- | --- |

| 763.8967 | 1525.779 | 1525.778 | 68.93 | LYDQLLQSYQQK |
| --- | --- | --- | --- | --- |

| Bone sialoprotein 2 OS=Bos taurus GN=IBSP PE=1 SV=1 | 60 |
| --- | --- |

| 548.2844 | 1094.554 | 1094.555 | 45.14 | HGYFYPALK |
| --- | --- | --- | --- | --- |

| Tubulin beta-2B chain OS=Bos taurus GN=TUBB2B PE=1 SV=2 | 57 |
| --- | --- |

| 615.3024 | 1228.59 | 1228.591 | 56.71 | ISEQFTAMFR |
| --- | --- | --- | --- | --- |

| Tubulin beta-3 chain OS=Bos taurus GN=TUBB3 PE=2 SV=1 | 57 |
| --- | --- |

| 615.3024 | 1228.59 | 1228.591 | 56.71 | ISEQFTAMFR |
| --- | --- | --- | --- | --- |

| Tubulin beta-4A chain OS=Bos taurus GN=TUBB4A PE=2 SV=1 | 57 |
| --- | --- |

| 615.3024 | 1228.59 | 1228.591 | 56.71 | ISEQFTAMFR |
| --- | --- | --- | --- | --- |

| Tubulin beta-4B chain OS=Bos taurus GN=TUBB4B PE=2 SV=1 | 57 |
| --- | --- |

| 615.3024 | 1228.59 | 1228.591 | 56.71 | ISEQFTAMFR |
| --- | --- | --- | --- | --- |

| Tubulin beta-5 chain OS=Bos taurus GN=TUBB5 PE=2 SV=1 | 57 |
| --- | --- |

| 615.3024 | 1228.59 | 1228.591 | 56.71 | ISEQFTAMFR |
| --- | --- | --- | --- | --- |

| Gelsolin OS=Bos taurus GN=GSN PE=2 SV=1 | 55 |
| --- | --- |

| 436.7634 | 871.5122 | 871.5127 | 55.33 | TGALELLR |  |
| --- | --- | --- | --- | --- | --- |

| Protein AMBP OS=Bos taurus GN=AMBP PE=1 SV=2 | 53 |
| --- | --- |

| 511.7605 | 1021.506 | 1021.508 | 53.02 | ESLLEEFR |  |
| --- | --- | --- | --- | --- | --- |

| Actin, alpha cardiac muscle 1 OS=Bos taurus GN=ACTC1 PE=2 SV=1 | 43 |
| --- | --- |

| 505.9207 | 1514.74 | 1514.742 | 43.24 | IWHHTFYNELR |
| --- | --- | --- | --- | --- |

| Actin, alpha skeletal muscle OS=Bos taurus GN=ACTA1 PE=1 SV=1 | 43 |
| --- | --- |

| 505.9207 | 1514.74 | 1514.742 | 43.24 | IWHHTFYNELR |
| --- | --- | --- | --- | --- |

| Prostaglandin-H2 D-isomerase OS=Bos taurus GN=PTGDS PE=1 SV=1 | 51 |
| --- | --- |

| 768.9171 | 1535.82 | 1535.824 | 51.43 | SLGFTEEGIVFLPK |
| --- | --- | --- | --- | --- |

| Coagulation factor X OS=Bos taurus GN=F10 PE=1 SV=1 | 51 |
| --- | --- |

| 447.2459 | 892.4772 | 892.4767 | 50.6 | TGIVSGFGR |
| --- | --- | --- | --- | --- |

| Fibromodulin OS=Bos taurus GN=FMOD PE=1 SV=2 | 50 |
| --- | --- |

| 779.4348 | 1556.855 | 1556.856 | 50.31 | SLILLDLSYNHLR | Deamidated (NQ) |
| --- | --- | --- | --- | --- | --- |

| Hemoglobin fetal subunit beta OS=Bos taurus PE=1 SV=1 | 48 |
| --- | --- |

| 637.8677 | 1273.721 | 1273.718 | 48.44 | LLVVYPWTQR |
| --- | --- | --- | --- | --- |

| Integrin beta-6 OS=Bos taurus GN=ITGB6 PE=1 SV=1 | 48 |
| --- | --- |

| 492.2631 | 982.5116 | 982.5124 | 48.34 | LGFGSFVEK |
| --- | --- | --- | --- | --- |

| Serotransferrin OS=Bos taurus GN=TF PE=2 SV=1 | 47 |
| --- | --- |

| 460.2841 | 918.5536 | 918.5538 | 46.66 | GYLAVAVVK |
| --- | --- | --- | --- | --- |

| Protein disulfide-isomerase OS=Bos taurus GN=P4HB PE=1 SV=1 | 46 |
| --- | --- |

| 541.342 | 1080.669 | 1080.67 | 42.46 | THILLFLPK |
| --- | --- | --- | --- | --- |

| Carboxypeptidase E OS=Bos taurus GN=CPE PE=1 SV=2 | 41 |
| --- | --- |

| 838.941 | 1675.867 | 1675.868 | 41.44 | AASQLGELKDWFVGR |
| --- | --- | --- | --- | --- |

**WK1**

| Chondroadherin OS=Bos taurus GN=CHAD PE=1 SV=1 | 72 |
| --- | --- |

| **pep_exp_mz** | **pep_exp_mr** | **pep_calc_mr** | **pep_score** | **pep_seq** | **pep_var_mod** |
| --- | --- | --- | --- | --- | --- |
| 678.3645 | 1354.714 | 1354.713 | 72.13 | FSDGAFLGVTTLK | |

| Pigment epithelium-derived factor OS=Bos taurus GN=SERPINF1 PE=1 SV=1 | 71 |
| --- | --- |

| 625.8358 | 1249.657 | 1249.655 | 70.99 | DTDTGALLFIGK |
| --- | --- | --- | --- | --- |

| Lumican OS=Bos taurus GN=LUM PE=1 SV=1 | 60 |
| --- | --- |

| 499.2761 | 996.5376 | 996.5392 | 51.17 | FSALQYLR | |
| --- | --- | --- | --- | --- | --- |
| 499.7686 | 997.5226 | 997.5233 | 46.94 | FSALQYLR | Deamidated (NQ) |

| Osteomodulin OS=Bos taurus GN=OMD PE=1 SV=1 | 55 |
| --- | --- |

| 606.8165 | 1211.618 | 1211.619 | 42.35 | IFLGYNEISR | Deamidated (NQ) |
| --- | --- | --- | --- | --- | --- |
| 606.8168 | 1211.619 | 1211.619 | 44.8 | IFLGYNEISR | Deamidated (NQ) |

| Prothrombin OS=Bos taurus GN=F2 PE=1 SV=2 | 48 |
| --- | --- |

| 456.7791 | 911.5436 | 911.544 | 48.3 | VTVEVIPR |
| --- | --- | --- | --- | --- |

| Alpha-S1-casein OS=Bos taurus GN=CSN1S1 PE=1 SV=2 | 45 |
| --- | --- |

| 692.8692 | 1383.724 | 1383.723 | 45.38 | FFVAPFPEVFGK |
| --- | --- | --- | --- | --- |

| Collagen alpha-2(XI) chain OS=Bos taurus GN=COL11A2 PE=3 SV=1 | 44 |
| --- | --- |

| 416.7557 | 831.4968 | 831.4967 | 42.41 | GVIIFGAR |  |
| --- | --- | --- | --- | --- | --- |

**AY5**

| SPARC OS=Bos taurus GN=SPARC PE=1 SV=2 | 80 |
| --- | --- |

| **pep_exp_mz** | **pep_exp_mr** | **pep_calc_mr** | **pep_score** | **pep_seq** | **pep_var_mod** |
| --- | --- | --- | --- | --- | --- |
| 821.9014 | 1641.788 | 1641.786 | 83.38 | YIALDEWAGCFGIK | |

| Olfactomedin-like protein 3 OS=Bos taurus GN=OLFML3 PE=2 SV=1 | 69 |
| --- | --- |

| 517.2766 | 1032.539 | 1032.539 | 53.67 | FGGPAGLWTK |
| --- | --- | --- | --- | --- |

| Carboxypeptidase E OS=Bos taurus GN=CPE PE=1 SV=2 | 65 |
| --- | --- |

| 844.9434 | 1687.872 | 1687.872 | 65.33 | EALVSVWLQCAAVSR |
| --- | --- | --- | --- | --- |

| Osteomodulin OS=Bos taurus GN=OMD PE=1 SV=1 | 57 |
| --- | --- |

| 606.3246 | 1210.635 | 1210.635 | 56.71 | IFLGYNEISR |
| --- | --- | --- | --- | --- |

| Antithrombin-III OS=Bos taurus GN=SERPINC1 PE=1 SV=2 | 55 |
| --- | --- |

| 682.3746 | 1362.735 | 1362.733 | 54.62 | EVALNTIIFMGR |
| --- | --- | --- | --- | --- |

| Coagulation factor X OS=Bos taurus GN=F10 PE=1 SV=1 | 54 |
| --- | --- |

| 731.6867 | 2192.038 | 2192.036 | 41.66 | FKDTYFVTGIVSWGEGCAR |
| --- | --- | --- | --- | --- |

| Coagulation factor IX (Fragment) OS=Bos taurus GN=F9 PE=1 SV=1 | 52 |
| --- | --- |

| 610.7905 | 1219.566 | 1219.566 | 48.4 | FGYGYVSGWGK |
| --- | --- | --- | --- | --- |

| Thrombospondin-1 OS=Bos taurus GN=THBS1 PE=2 SV=2 | 52 |
| --- | --- |

| 495.3104 | 988.6062 | 988.6069 | 56.92 | GFLLLASLR |
| --- | --- | --- | --- | --- |

| Nucleobindin-1 OS=Bos taurus GN=NUCB1 PE=2 SV=1 | 51 |
| --- | --- |

| 637.3538 | 1272.693 | 1272.693 | 51.17 | DLELLIQTATR | Deamidated (NQ) |
| --- | --- | --- | --- | --- | --- |

| Matrix Gla protein OS=Bos taurus GN=MGP PE=1 SV=1 | 45 |
| --- | --- |

| 786.8414 | 1571.668 | 1571.671 | 44.7 | YAMVYGYNAAYDR | Oxidation (M) |
| --- | --- | --- | --- | --- | --- |

| Collagen alpha-1(XI) chain (Fragment) OS=Bos taurus GN=COL11A1 PE=1 SV=1 | 44 |
| --- | --- |

| 615.8275 | 1229.64 | 1229.641 | 43.7 | LGVPGLPGYPGR | 3 Oxidation (P) |
| --- | --- | --- | --- | --- | --- |

| Prothrombin OS=Bos taurus GN=F2 PE=1 SV=2 | 44 |
| --- | --- |

| 823.4149 | 1644.815 | 1644.814 | 43.55 | SPQELLCGASLISDR |
| --- | --- | --- | --- | --- |

| Alkaline phosphatase, tissue-nonspecific isozyme OS=Bos taurus GN=ALPL PE=1 SV=2 | 43 |
| --- | --- |

| 547.8035 | 1093.592 | 1093.592 | 43.36 | GFFLLVEGGR |
| --- | --- | --- | --- | --- |

| Tetranectin OS=Bos taurus GN=CLEC3B PE=2 SV=1 | 41 |
| --- | --- |

| 542.2863 | 1082.558 | 1082.558 | 41.02 | CFLAFVQAK |
| --- | --- | --- | --- | --- |

**UI3**

| Tetranectin OS=Bos taurus GN=CLEC3B PE=2 SV=1 | 162 |
| --- | --- |

| **pep_exp_mz** | **pep_exp_mr** | **pep_calc_mr** | **pep_score** | **pep_seq** | **pep_var_mod** |
| --- | --- | --- | --- | --- | --- |
| 764.9326 | 1527.851 | 1527.851 | 90.95 | TQLDSLAQEVALLK | |
| 765.4247 | 1528.835 | 1528.835 | 100.74 | TQLDSLAQEVALLK | Deamidated (NQ) |

| SPARC OS=Bos taurus GN=SPARC PE=1 SV=2 | 108 |
| --- | --- |

| 821.8995 | 1641.784 | 1641.786 | 83.69 | YIALDEWAGCFGIK |
| --- | --- | --- | --- | --- |
| 821.9008 | 1641.787 | 1641.786 | 45.51 | YIALDEWAGCFGIK |

| Thrombospondin-1 OS=Bos taurus GN=THBS1 PE=2 SV=2 | 91 |
| --- | --- |

| 495.3102 | 988.6058 | 988.6069 | 58.5 | GFLLLASLR |
| --- | --- | --- | --- | --- |
| 495.3106 | 988.6066 | 988.6069 | 65.16 | GFLLLASLR |

| Antithrombin-III OS=Bos taurus GN=SERPINC1 PE=1 SV=2 | 81 |
| --- | --- |

| 690.8624 | 1379.71 | 1379.712 | 80.71 | EVALNTIIFMGR | Deamidated (NQ); Oxidation (M) |
| --- | --- | --- | --- | --- | --- |

| Osteomodulin OS=Bos taurus GN=OMD PE=1 SV=1 | 79 |
| --- | --- |

| 606.8162 | 1211.618 | 1211.619 | 53.69 | IFLGYNEISR | Deamidated (NQ) |
| --- | --- | --- | --- | --- | --- |
| 606.8165 | 1211.618 | 1211.619 | 48.2 | IFLGYNEISR | Deamidated (NQ) |
| 606.8168 | 1211.619 | 1211.619 | 52.84 | IFLGYNEISR | Deamidated (NQ) |

| Secreted phosphoprotein 24 OS=Bos taurus GN=SPP2 PE=1 SV=2 | 73 |
| --- | --- |

| 706.3645 | 1410.714 | 1410.714 | 52.21 | VNSQSLSPYLFR | Deamidated (NQ) |
| --- | --- | --- | --- | --- | --- |
| 706.8569 | 1411.699 | 1411.698 | 47.95 | VNSQSLSPYLFR | 2 Deamidated (NQ) |

| Olfactomedin-like protein 3 OS=Bos taurus GN=OLFML3 PE=2 SV=1 | 65 |
| --- | --- |

| 517.277 | 1032.539 | 1032.539 | 65.11 | FGGPAGLWTK |
| --- | --- | --- | --- | --- |

| Coagulation factor X OS=Bos taurus GN=F10 PE=1 SV=1 | 65 |
| --- | --- |

| 671.3367 | 1340.659 | 1340.661 | 65.08 | ETYDFDIAVLR |
| --- | --- | --- | --- | --- |

| Vitamin D-binding protein OS=Bos taurus GN=GC PE=2 SV=1 | 65 |
| --- | --- |

| 691.8696 | 1381.725 | 1381.724 | 64.74 | VLDQYIFELSR |
| --- | --- | --- | --- | --- |

| Prothrombin OS=Bos taurus GN=F2 PE=1 SV=2 | 61 |
| --- | --- |

| 1138.075 | 2274.135 | 2274.136 | 54.57 | IVEGQDAEVGLSPWQVMLFR | Deamidated (NQ) |
| --- | --- | --- | --- | --- | --- |

| Apolipoprotein A-II OS=Bos taurus GN=APOA2 PE=1 SV=2 | 56 |
| --- | --- |

| 869.4583 | 1736.902 | 1736.899 | 55.56 | AGTDLLNFLSSFIDPK |
| --- | --- | --- | --- | --- |

| Histone H2A.V OS=Bos taurus GN=H2AFV PE=2 SV=3 | 51 |
| --- | --- |

| 472.7693 | 943.524 | 943.524 | 50.7 | AGLQFPVGR |
| --- | --- | --- | --- | --- |

| Histone H2A.Z OS=Bos taurus GN=H2AFZ PE=1 SV=2 | 51 |
| --- | --- |

| 472.7693 | 943.524 | 943.524 | 50.7 | AGLQFPVGR |
| --- | --- | --- | --- | --- |

| Vitamin K-dependent protein C (Fragment) OS=Bos taurus GN=PROC PE=1 SV=1 | 50 |
| --- | --- |

| 654.3434 | 1306.672 | 1306.671 | 49.48 | YLDWIYGHIK |
| --- | --- | --- | --- | --- |

| Dermatopontin OS=Bos taurus GN=DPT PE=1 SV=3 | 48 |
| --- | --- |

| 514.7559 | 1027.497 | 1027.498 | 47.96 | YFESVLDR |
| --- | --- | --- | --- | --- |

| Complement C3 OS=Bos taurus GN=C3 PE=1 SV=2 | 43 |
| --- | --- |

| 635.3585 | 1268.702 | 1268.702 | 42.5 | LVAYYTLINAK | Deamidated (NQ) |
| --- | --- | --- | --- | --- | --- |

| Peptidyl-prolyl cis-trans isomerase B OS=Bos taurus GN=PPIB PE=1 SV=4 | 41 |
| --- | --- |

| 416.7681 | 831.5216 | 831.5218 | 41.48 | VVIGLFGK |
| --- | --- | --- | --- | --- |

**GuHCl Batch 2**

**Modern**

| Peripherin OS=Bos taurus GN=PRPH PE=2 SV=1 | 72 |
| --- | --- |

| **pep_exp_mz** | **pep_exp_mr** | **pep_calc_mr** | **pep_score** | **pep_seq** | **pep_var_mod** |
| --- | --- | --- | --- | --- | --- |
| 648.336 | 1294.657 | 1294.659 | 70.71 | MALDIEIATYR | |

| Histone H2B type 1-N OS=Bos taurus GN=HIST1H2BN PE=1 SV=3 | 48 |
| --- | --- |

| 633.324 | 1264.633 | 1264.634 | 46.66 | KESYSVYVYK |
| --- | --- | --- | --- | --- |

| Serpin H1 OS=Bos taurus GN=SERPINH1 PE=2 SV=1 | 99 |
| --- | --- |

| 855.8199 | 2564.438 | 2564.443 | 71.28 | DQAVENILLSPVVVASSLGLVSLGGK |
| --- | --- | --- | --- | --- |

| Lysyl oxidase homolog 4 OS=Bos taurus GN=LOXL4 PE=2 SV=1 | 92 |
| --- | --- |

| 1103.604 | 2205.193 | 2205.191 | 86.61 | EALFGAQLGQALGPIHLSEVR |
| --- | --- | --- | --- | --- |

| Histone H1.2 OS=Bos taurus GN=HIST1H1C PE=1 SV=2 | 85 |
| --- | --- |

| 423.2581 | 844.5016 | 844.5018 | 72.28 | SGVSLAALK |
| --- | --- | --- | --- | --- |
| 487.3053 | 972.596 | 972.5968 | 49.48 | SGVSLAALKK |

| Histone H1.3 OS=Bos taurus GN=HIST1H1D PE=1 SV=1 | 85 |
| --- | --- |

| 423.2581 | 844.5016 | 844.5018 | 72.28 | SGVSLAALK |
| --- | --- | --- | --- | --- |
| 487.3053 | 972.596 | 972.5968 | 49.48 | SGVSLAALKK |

| Histone H1.1 OS=Bos taurus GN=HIST1H1A PE=1 SV=1 | 84 |
| --- | --- |

| 423.2581 | 844.5016 | 844.5018 | 72.28 | SGVSLAALK |
| --- | --- | --- | --- | --- |
| 487.3053 | 972.596 | 972.5968 | 49.48 | SGVSLAALKK |

| Histone H1.0 OS=Bos taurus GN=H1F0 PE=2 SV=3 | 84 |
| --- | --- |

| 719.8666 | 1437.719 | 1437.717 | 83.58 | YSDMIVAAIQAEK |
| --- | --- | --- | --- | --- |

| Complement C1q subcomponent subunit B OS=Bos taurus GN=C1QB PE=1 SV=1 | 83 |
| --- | --- |

| 822.9113 | 1643.808 | 1643.81 | 75.52 | VPGLYFFTYHASSR |
| --- | --- | --- | --- | --- |

| Complement C4 (Fragments) OS=Bos taurus GN=C4 PE=1 SV=2 | 82 |
| --- | --- |

| 716.3647 | 1430.715 | 1430.715 | 86.37 | AELADQAASWLTR |
| --- | --- | --- | --- | --- |

| Endoplasmin OS=Bos taurus GN=HSP90B1 PE=2 SV=1 | 81 |
| --- | --- |

| 1004.023 | 2006.031 | 2006.03 | 68.84 | KYSQFINFPIYVWSSK |
| --- | --- | --- | --- | --- |

| Carbonic anhydrase 3 OS=Bos taurus GN=CA3 PE=2 SV=3 | 77 |
| --- | --- |

| 809.9388 | 1617.863 | 1617.861 | 72.02 | EKGEFQLLLDALDK |
| --- | --- | --- | --- | --- |

| ATP synthase subunit beta, mitochondrial OS=Bos taurus GN=ATP5B PE=1 SV=2 | 75 |
| --- | --- |

| 720.3987 | 1438.783 | 1438.782 | 69.6 | VALTGLTVAEYFR |
| --- | --- | --- | --- | --- |

| 72 kDa type IV collagenase OS=Bos taurus GN=MMP2 PE=2 SV=1 | 74 |
| --- | --- |

| 703.0162 | 2106.027 | 2106.027 | 40.48 | IIGYTPDLDPQTVDDAFAR |
| --- | --- | --- | --- | --- |
| 1054.022 | 2106.029 | 2106.027 | 41.78 | IIGYTPDLDPQTVDDAFAR |

| Clusterin OS=Bos taurus GN=CLU PE=1 SV=1 | 72 |
| --- | --- |

| 763.8957 | 1525.777 | 1525.778 | 72.32 | LYDQLLQSYQQK |
| --- | --- | --- | --- | --- |

| Serotransferrin OS=Bos taurus GN=TF PE=2 SV=1 | 72 |
| --- | --- |

| 822.8577 | 1643.701 | 1643.704 | 70.03 | FDEFFSAGCAPGSPR |
| --- | --- | --- | --- | --- |

| Moesin OS=Bos taurus GN=MSN PE=2 SV=3 | 71 |
| --- | --- |

| 945.9702 | 1889.926 | 1889.923 | 58.46 | IAQDLEMYGVNYFSIK |
| --- | --- | --- | --- | --- |

| Tubulin beta-3 chain OS=Bos taurus GN=TUBB3 PE=2 SV=1 | 57 |
| --- | --- |

| 615.3027 | 1228.591 | 1228.591 | 56.61 | ISEQFTAMFR |
| --- | --- | --- | --- | --- |

| Tubulin beta-6 chain OS=Bos taurus GN=TUBB6 PE=2 SV=1 | 47 |
| --- | --- |

| 520.3002 | 1038.586 | 1038.586 | 47.48 | YLTVAAVFR |
| --- | --- | --- | --- | --- |

| Beta-2-microglobulin OS=Bos taurus GN=B2M PE=1 SV=2 | 65 |
| --- | --- |

| 1021.978 | 2041.941 | 2041.942 | 54.09 | DWSFYLLSHAEFTPNSK | Deamidated (NQ) |
| --- | --- | --- | --- | --- | --- |

| Reticulocalbin-3 OS=Bos taurus GN=RCN3 PE=2 SV=1 | 64 |
| --- | --- |

| 751.3925 | 1500.77 | 1500.767 | 64.17 | DIVIAETLEDLDR |
| --- | --- | --- | --- | --- |

| Glutathione S-transferase P OS=Bos taurus GN=GSTP1 PE=1 SV=2 | 64 |
| --- | --- |

| 768.9151 | 1535.816 | 1535.814 | 63.52 | PPYTIVYFPVQGR |
| --- | --- | --- | --- | --- |

| Alpha-actinin-4 OS=Bos taurus GN=ACTN4 PE=2 SV=1 | 62 |
| --- | --- |

| 608.3405 | 1214.666 | 1214.666 | 62.04 | LASDLLEWIR |
| --- | --- | --- | --- | --- |

| Alpha-actinin-1 OS=Bos taurus GN=ACTN1 PE=2 SV=1 | 62 |
| --- | --- |

| 608.3405 | 1214.666 | 1214.666 | 62.04 | LASDLLEWIR |
| --- | --- | --- | --- | --- |

| Elongation factor 1-alpha 2 OS=Bos taurus GN=EEF1A2 PE=2 SV=1 | 46 |
| --- | --- |

| 657.8748 | 1313.735 | 1313.734 | 46.06 | EHALLAYTLGVK |
| --- | --- | --- | --- | --- |

| Fibrinogen beta chain OS=Bos taurus GN=FGB PE=1 SV=2 | 61 |
| --- | --- |

| 806.3505 | 1610.686 | 1610.686 | 40.01 | YYWGGAYTWDMAK | |
| --- | --- | --- | --- | --- | --- |
| 814.3486 | 1626.683 | 1626.681 | 52.76 | YYWGGAYTWDMAK | Oxidation (M) |

| Beta-enolase OS=Bos taurus GN=ENO3 PE=2 SV=1 | 60 |
| --- | --- |

| 902.9752 | 1803.936 | 1803.937 | 59.8 | AAVPSGASTGIYEALELR |
| --- | --- | --- | --- | --- |

| 14-3-3 protein beta/alpha OS=Bos taurus GN=YWHAB PE=1 SV=2 | 49 |
| --- | --- |

| 595.3344 | 1188.654 | 1188.654 | 45.84 | DSTLIMQLLR | |
| --- | --- | --- | --- | --- | --- |
| 603.332 | 1204.649 | 1204.649 | 40.57 | DSTLIMQLLR | Oxidation (M) |

| 14-3-3 protein eta OS=Bos taurus GN=YWHAH PE=1 SV=2 | 49 |
| --- | --- |

| 595.3344 | 1188.654 | 1188.654 | 45.84 | DSTLIMQLLR | |
| --- | --- | --- | --- | --- | --- |
| 603.332 | 1204.649 | 1204.649 | 40.57 | DSTLIMQLLR | Oxidation (M) |

| 14-3-3 protein gamma OS=Bos taurus GN=YWHAG PE=1 SV=2 | 49 |
| --- | --- |

| 595.3344 | 1188.654 | 1188.654 | 45.84 | DSTLIMQLLR | |
| --- | --- | --- | --- | --- | --- |
| 603.332 | 1204.649 | 1204.649 | 40.57 | DSTLIMQLLR | Oxidation (M) |

| 14-3-3 protein sigma OS=Bos taurus GN=SFN PE=2 SV=1 | 49 |
| --- | --- |

| 595.3344 | 1188.654 | 1188.654 | 45.84 | DSTLIMQLLR | |
| --- | --- | --- | --- | --- | --- |
| 603.332 | 1204.649 | 1204.649 | 40.57 | DSTLIMQLLR | Oxidation (M) |

| 14-3-3 protein theta OS=Bos taurus GN=YWHAQ PE=2 SV=1 | 49 |
| --- | --- |

| 595.3344 | 1188.654 | 1188.654 | 45.84 | DSTLIMQLLR | |
| --- | --- | --- | --- | --- | --- |
| 603.332 | 1204.649 | 1204.649 | 40.57 | DSTLIMQLLR | Oxidation (M) |

| 14-3-3 protein zeta/delta OS=Bos taurus GN=YWHAZ PE=1 SV=1 | 49 |
| --- | --- |

| 595.3344 | 1188.654 | 1188.654 | 45.84 | DSTLIMQLLR | |
| --- | --- | --- | --- | --- | --- |
| 603.332 | 1204.649 | 1204.649 | 40.57 | DSTLIMQLLR | Oxidation (M) |

| Apolipoprotein E OS=Bos taurus GN=APOE PE=2 SV=1 | 59 |
| --- | --- |

| 609.3169 | 1216.619 | 1216.62 | 59.44 | FGPLVEQGQSR |
| --- | --- | --- | --- | --- |

| Histone H3.1 OS=Bos taurus PE=1 SV=2 | 58 |
| --- | --- |

| 416.2501 | 830.4856 | 830.4861 | 60.41 | STELLIR |  |
| --- | --- | --- | --- | --- | --- |

| Histone H3.2 OS=Bos taurus PE=1 SV=2 | 58 |
| --- | --- |

| 416.2501 | 830.4856 | 830.4861 | 60.41 | STELLIR |  |
| --- | --- | --- | --- | --- | --- |

| Histone H3.3 OS=Bos taurus GN=H3F3A PE=1 SV=3 | 58 |
| --- | --- |

| 416.2501 | 830.4856 | 830.4861 | 60.41 | STELLIR |  |
| --- | --- | --- | --- | --- | --- |

| Histone H3.3C OS=Bos taurus GN=H3F3C PE=2 SV=1 | 58 |
| --- | --- |

| 416.2501 | 830.4856 | 830.4861 | 60.41 | STELLIR |  |
| --- | --- | --- | --- | --- | --- |

| Tropomyosin beta chain OS=Bos taurus GN=TPM2 PE=2 SV=1 | 57 |
| --- | --- |

| 433.5942 | 1297.761 | 1297.761 | 56.82 | KLVILEGELER |
| --- | --- | --- | --- | --- |

| Heat shock protein HSP 90-alpha OS=Bos taurus GN=HSP90AA1 PE=2 SV=3 | 56 |
| --- | --- |

| 675.3704 | 1348.726 | 1348.727 | 49.44 | TLTIVDTGIGMTK |
| --- | --- | --- | --- | --- |

| Nucleotide exchange factor SIL1 OS=Bos taurus GN=SIL1 PE=2 SV=1 | 56 |
| --- | --- |

| 770.4715 | 1538.928 | 1538.928 | 55.91 | LLVILATEQPLTTK |
| --- | --- | --- | --- | --- |

| Annexin A2 OS=Bos taurus GN=ANXA2 PE=1 SV=2 | 56 |
| --- | --- |

| 514.9538 | 1541.84 | 1541.841 | 55.75 | GVDEVTIVNILTNR |
| --- | --- | --- | --- | --- |

| Complement component C7 OS=Bos taurus GN=C7 PE=2 SV=1 | 55 |
| --- | --- |

| 735.4021 | 1468.79 | 1468.793 | 55.18 | LSGNILSYTFQVK |
| --- | --- | --- | --- | --- |

| Aggrecan core protein OS=Bos taurus GN=ACAN PE=1 SV=3 | 55 |
| --- | --- |

| 461.9137 | 1382.719 | 1382.72 | 54.96 | ARPNCGGNLLGVR |
| --- | --- | --- | --- | --- |

| Heat shock protein beta-1 OS=Bos taurus GN=HSPB1 PE=2 SV=1 | 54 |
| --- | --- |

| 1157.554 | 2313.093 | 2313.097 | 54.46 | LPEEWSQWLSHSGWPGYVR |
| --- | --- | --- | --- | --- |

| Bone sialoprotein 2 OS=Bos taurus GN=IBSP PE=1 SV=1 | 54 |
| --- | --- |

| 548.2844 | 1094.554 | 1094.555 | 52.7 | HGYFYPALK |
| --- | --- | --- | --- | --- |

| Purine nucleoside phosphorylase OS=Bos taurus GN=PNP PE=1 SV=3 | 52 |
| --- | --- |

| 563.3179 | 1124.621 | 1124.623 | 51.75 | VFGFSLITNK |
| --- | --- | --- | --- | --- |

| Cadherin-2 (Fragment) OS=Bos taurus GN=CDH2 PE=2 SV=1 | 51 |
| --- | --- |

| 662.8771 | 1323.74 | 1323.74 | 51.03 | DVLEGQPLLNVK |
| --- | --- | --- | --- | --- |

| Collagen alpha-1(III) chain OS=Bos taurus GN=COL3A1 PE=1 SV=1 | 51 |
| --- | --- |

| 1049.556 | 2097.097 | 2097.097 | 52.51 | GAPGPQGPPGAPGPLGIAGLTGAR | 2 Oxidation (P) |
| --- | --- | --- | --- | --- | --- |

| Macrophage migration inhibitory factor OS=Bos taurus GN=MIF PE=1 SV=6 | 50 |
| --- | --- |

| 537.8021 | 1073.59 | 1073.59 | 49.56 | LLCGLLTER |
| --- | --- | --- | --- | --- |

| Heat shock protein HSP 90-beta OS=Bos taurus GN=HSP90AB1 PE=2 SV=3 | 49 |
| --- | --- |

| 675.3704 | 1348.726 | 1348.727 | 49.44 | TLTLVDTGIGMTK |
| --- | --- | --- | --- | --- |

| Serpin A3-7 OS=Bos taurus GN=SERPINA3-7 PE=3 SV=1 | 49 |
| --- | --- |

| 493.7979 | 985.5812 | 985.5808 | 48.84 | DILSQLGIK |
| --- | --- | --- | --- | --- |

| Coagulation factor VII OS=Bos taurus GN=F7 PE=1 SV=2 | 48 |
| --- | --- |

| 463.7965 | 925.5784 | 925.5783 | 41.2 | LMVVLVPR |
| --- | --- | --- | --- | --- |

| Platelet-activating factor acetylhydrolase IB subunit beta OS=Bos taurus GN=PAFAH1B2 PE=1 SV=1 | 48 |
| --- | --- |

| 497.3448 | 992.675 | 992.6746 | 47.82 | IIVLGLLPR |
| --- | --- | --- | --- | --- |

| Fibrinogen alpha chain OS=Bos taurus GN=FGA PE=1 SV=5 | 46 |
| --- | --- |

| 560.7612 | 1119.508 | 1119.51 | 45.89 | GNLDDFFHR |
| --- | --- | --- | --- | --- |

| Fibroleukin OS=Bos taurus GN=FGL2 PE=2 SV=1 | 46 |
| --- | --- |

| 738.4086 | 1474.803 | 1474.803 | 42.3 | IEEVFKEVQNLK |
| --- | --- | --- | --- | --- |

| Alpha-crystallin B chain OS=Bos taurus GN=CRYAB PE=1 SV=2 | 44 |
| --- | --- |

| 461.2554 | 920.4962 | 920.4967 | 45.86 | FSVNLDVK |
| --- | --- | --- | --- | --- |

| 78 kDa glucose-regulated protein OS=Bos taurus GN=HSPA5 PE=2 SV=1 | 42 |
| --- | --- |

| 768.9035 | 1535.792 | 1535.791 | 42.5 | TFAPEEISAMVLTK |
| --- | --- | --- | --- | --- |

| GTP-binding nuclear protein Ran OS=Bos taurus GN=RAN PE=2 SV=3 | 42 |
| --- | --- |

| 595.6423 | 1783.905 | 1783.905 | 41.76 | SNYNFEKPFLWLAR |
| --- | --- | --- | --- | --- |

| Heat shock 70 kDa protein 1-like OS=Bos taurus GN=HSPA1L PE=3 SV=1 | 42 |
| --- | --- |

| 599.351 | 1196.687 | 1196.688 | 41.59 | DAGVIAGLNVLR |
| --- | --- | --- | --- | --- |

| Heat shock 70 kDa protein 1A OS=Bos taurus GN=HSPA1A PE=2 SV=2 | 42 |
| --- | --- |

| 599.351 | 1196.687 | 1196.688 | 41.59 | DAGVIAGLNVLR |
| --- | --- | --- | --- | --- |

| Heat shock 70 kDa protein 1B OS=Bos taurus GN=HSPA1B PE=2 SV=1 | 42 |
| --- | --- |

| 599.351 | 1196.687 | 1196.688 | 41.59 | DAGVIAGLNVLR |
| --- | --- | --- | --- | --- |

**AuCPC**

| Collagen alpha-1(II) chain OS=Bos taurus GN=COL2A1 PE=1 SV=4 | 203 |
| --- | --- |

| **pep_exp_mz** | **pep_exp_mr** | **pep_calc_mr** | **pep_score** | **pep_seq** | **pep_var_mod** |
| --- | --- | --- | --- | --- | --- |
| 656.8306 | 1311.647 | 1311.646 | 47.54 | GFPGLPGPSGEPGK | Oxidation (P) |
| 656.8309 | 1311.647 | 1311.646 | 56.77 | GFPGLPGPSGEPGK | Oxidation (P) |
| 664.8282 | 1327.642 | 1327.641 | 54.24 | GFPGLPGPSGEPGK | 2 Oxidation (P) |
| 664.8282 | 1327.642 | 1327.641 | 41.26 | GFPGLPGPSGEPGK | 2 Oxidation (P) |
| 664.8284 | 1327.642 | 1327.641 | 48.56 | GFPGLPGPSGEPGK | 2 Oxidation (P) |
| 664.8284 | 1327.642 | 1327.641 | 40.78 | GFPGLPGPSGEPGK | 2 Oxidation (P) |
| 664.8285 | 1327.642 | 1327.641 | 50.73 | GFPGLPGPSGEPGK | 2 Oxidation (P) |
| 664.8287 | 1327.643 | 1327.641 | 51.08 | GFPGLPGPSGEPGK | 2 Oxidation (P) |
| 672.8251 | 1343.636 | 1343.636 | 51.04 | GFPGLPGPSGEPGK | 3 Oxidation (P) |
| 672.8256 | 1343.637 | 1343.636 | 53.1 | GFPGLPGPSGEPGK | 3 Oxidation (P) |
| 672.826 | 1343.637 | 1343.636 | 62.33 | GFPGLPGPSGEPGK | 3 Oxidation (P) |
| 672.8261 | 1343.638 | 1343.636 | 46.75 | GFPGLPGPSGEPGK | Oxidation (K); 2 Oxidation (P) |

| Matrix Gla protein OS=Bos taurus GN=MGP PE=1 SV=1 | 141 |
| --- | --- |

| 779.3369 | 1556.659 | 1556.661 | 54.34 | YAMVYGYNAAYDR | Deamidated (NQ) |
| --- | --- | --- | --- | --- | --- |
| 786.8433 | 1571.672 | 1571.671 | 59.9 | YAMVYGYNAAYDR | Oxidation (M) |
| 787.3352 | 1572.656 | 1572.656 | 54.77 | YAMVYGYNAAYDR | Deamidated (NQ); Oxidation (M) |
| 787.336 | 1572.657 | 1572.656 | 46.58 | YAMVYGYNAAYDR | Deamidated (NQ); Oxidation (M) |

| Secreted phosphoprotein 24 OS=Bos taurus GN=SPP2 PE=1 SV=2 | 134 |
| --- | --- |

| 705.8727 | 1409.731 | 1409.73 | 51.96 | VNSQSLSPYLFR | |
| --- | --- | --- | --- | --- | --- |
| 705.873 | 1409.731 | 1409.73 | 56.34 | VNSQSLSPYLFR | |
| 706.3648 | 1410.715 | 1410.714 | 67.87 | VNSQSLSPYLFR | Deamidated (NQ) |

| Nucleobindin-1 OS=Bos taurus GN=NUCB1 PE=2 SV=1 | 95 |
| --- | --- |

| 966.994 | 1931.973 | 1931.974 | 81.43 | YLQEVINVLETDGHFR |
| --- | --- | --- | --- | --- |

| Complement component C7 OS=Bos taurus GN=C7 PE=2 SV=1 | 82 |
| --- | --- |

| 735.4045 | 1468.794 | 1468.793 | 82.48 | LSGNILSYTFQVK |
| --- | --- | --- | --- | --- |

| Extracellular matrix protein 2 OS=Bos taurus GN=ECM2 PE=2 SV=1 | 76 |
| --- | --- |

| 650.3271 | 1298.64 | 1298.641 | 52.73 | VSFYGAYHSLR |
| --- | --- | --- | --- | --- |

| Gelsolin OS=Bos taurus GN=GSN PE=2 SV=1 | 70 |
| --- | --- |

| 861.9238 | 1721.833 | 1721.83 | 69.88 | EVQGFESATFLGYFK |
| --- | --- | --- | --- | --- |

| Apolipoprotein A-II OS=Bos taurus GN=APOA2 PE=1 SV=2 | 64 |
| --- | --- |

| 869.9477 | 1737.881 | 1737.883 | 63.9 | AGTDLLNFLSSFIDPK | Deamidated (NQ) |
| --- | --- | --- | --- | --- | --- |

| Vitamin K-dependent protein C (Fragment) OS=Bos taurus GN=PROC PE=1 SV=1 | 60 |
| --- | --- |

| 477.787 | 953.5594 | 953.5586 | 45.98 | TFVLSFIK |  |
| --- | --- | --- | --- | --- | --- |

| Olfactomedin-like protein 3 OS=Bos taurus GN=OLFML3 PE=2 SV=1 | 57 |
| --- | --- |

| 881.4254 | 1760.836 | 1760.841 | 57.27 | QLYAWDDGYQIVYK |
| --- | --- | --- | --- | --- |

| Lactadherin OS=Bos taurus GN=MFGE8 PE=1 SV=2 | 57 |
| --- | --- |

| 854.9514 | 1707.888 | 1707.883 | 56.64 | INLFDTPLETQYVR |
| --- | --- | --- | --- | --- |

| Hyaluronan and proteoglycan link protein 1 OS=Bos taurus GN=HAPLN1 PE=2 SV=1 | 52 |
| --- | --- |

| 510.2875 | 1018.56 | 1018.56 | 52.33 | VGQIFAAWK |
| --- | --- | --- | --- | --- |

| Fibromodulin OS=Bos taurus GN=FMOD PE=1 SV=2 | 48 |
| --- | --- |

| 519.9597 | 1556.857 | 1556.856 | 50.99 | SLILLDLSYNHLR | Deamidated (NQ) |
| --- | --- | --- | --- | --- | --- |

| Coagulation factor VII OS=Bos taurus GN=F7 PE=1 SV=2 | 50 |
| --- | --- |

| 725.3796 | 1448.745 | 1448.741 | 43.66 | FSAVSGWGQLLER |
| --- | --- | --- | --- | --- |

| Kininogen-1 OS=Bos taurus GN=KNG1 PE=1 SV=1 | 50 |
| --- | --- |

| 484.7818 | 967.549 | 967.5491 | 49.69 | YSIVFIAR |  |
| --- | --- | --- | --- | --- | --- |

| Serotransferrin OS=Bos taurus GN=TF PE=2 SV=1 | 49 |
| --- | --- |

| 460.2845 | 918.5544 | 918.5538 | 49.04 | GYLAVAVVK |
| --- | --- | --- | --- | --- |

| Sorting nexin-4 OS=Bos taurus GN=SNX4 PE=2 SV=1 | 47 |
| --- | --- |

| 537.8193 | 1073.624 | 1073.623 | 47.23 | IGLENFLLR |
| --- | --- | --- | --- | --- |

| Unconventional myosin-Id OS=Bos taurus GN=MYO1D PE=2 SV=1 | 45 |
| --- | --- |

| 540.7872 | 1079.56 | 1079.559 | 44.57 | LFCWIVSR |
| --- | --- | --- | --- | --- |

| Collagen alpha-1(III) chain OS=Bos taurus GN=COL3A1 PE=1 SV=1 | 41 |
| --- | --- |

| 700.0402 | 2097.099 | 2097.097 | 40.63 | GAPGPQGPPGAPGPLGIAGLTGAR | 2 Oxidation (P) |
| --- | --- | --- | --- | --- | --- |

| Vitrin OS=Bos taurus GN=VIT PE=2 SV=2 | 40 |
| --- | --- |

| 516.2994 | 1030.584 | 1030.585 | 40.38 | LMILITDGR |
| --- | --- | --- | --- | --- |

**NF1**

| Matrix Gla protein OS=Bos taurus GN=MGP PE=1 SV=1 | 122 |
| --- | --- |

| **pep_exp_mz** | **pep_exp_mr** | **pep_calc_mr** | **pep_score** | **pep_seq** | **pep_var_mod** |
| --- | --- | --- | --- | --- | --- |
| 779.3361 | 1556.658 | 1556.661 | 52.18 | YAMVYGYNAAYDR | Deamidated (NQ) |
| 779.3376 | 1556.661 | 1556.661 | 50.56 | YAMVYGYNAAYDR | Deamidated (NQ) |
| 787.3348 | 1572.655 | 1572.656 | 47.5 | YAMVYGYNAAYDR | Deamidated (NQ); Oxidation (M) |
| 787.3356 | 1572.657 | 1572.656 | 44.04 | YAMVYGYNAAYDR | Deamidated (NQ); Oxidation (M) |

| Olfactomedin-like protein 3 OS=Bos taurus GN=OLFML3 PE=2 SV=1 | 94 |
| --- | --- |

| 881.4258 | 1760.837 | 1760.841 | 66.16 | QLYAWDDGYQIVYK |
| --- | --- | --- | --- | --- |
| 881.4279 | 1760.841 | 1760.841 | 58.35 | QLYAWDDGYQIVYK |

| Antithrombin-III OS=Bos taurus GN=SERPINC1 PE=1 SV=2 | 87 |
| --- | --- |

| 682.8657 | 1363.717 | 1363.717 | 61.05 | EVALNTIIFMGR | Deamidated (NQ) |
| --- | --- | --- | --- | --- | --- |
| 690.3704 | 1378.726 | 1378.728 | 60.6 | EVALNTIIFMGR | Oxidation (M) |

| Fibromodulin OS=Bos taurus GN=FMOD PE=1 SV=2 | 85 |
| --- | --- |

| 519.9586 | 1556.854 | 1556.856 | 45.71 | SLILLDLSYNHLR | Deamidated (NQ) |
| --- | --- | --- | --- | --- | --- |
| 779.4346 | 1556.855 | 1556.856 | 54.99 | SLILLDLSYNHLR | Deamidated (NQ) |
| 779.435 | 1556.855 | 1556.856 | 64.04 | SLILLDLSYNHLR | Deamidated (NQ) |

| Vitrin OS=Bos taurus GN=VIT PE=2 SV=2 | 85 |
| --- | --- |

| 1022.001 | 2041.987 | 2041.99 | | 62.26 | | TNGFYSLTVQNWFSLHK | Deamidated (NQ) |
| --- | --- | --- | --- | --- | --- | --- | --- |
| Complement component C9 OS=Bos taurus GN=C9 PE=2 SV=1 | | | 81 | |  |  |  |

| 728.8506 | 1455.687 | 1455.688 | 72.45 | AIEDYINEFSVR | Deamidated (NQ) |
| --- | --- | --- | --- | --- | --- |

| Secreted phosphoprotein 24 OS=Bos taurus GN=SPP2 PE=1 SV=2 | 79 |
| --- | --- |

| 705.8721 | 1409.73 | 1409.73 | 41.71 | VNSQSLSPYLFR | |
| --- | --- | --- | --- | --- | --- |
| 706.8571 | 1411.7 | 1411.698 | 56.62 | VNSQSLSPYLFR | 2 Deamidated (NQ) |

| Nucleobindin-1 OS=Bos taurus GN=NUCB1 PE=2 SV=1 | 73 |
| --- | --- |

| 644.9974 | 1931.97 | 1931.974 | 46.64 | YLQEVINVLETDGHFR |
| --- | --- | --- | --- | --- |
| 966.9926 | 1931.971 | 1931.974 | 55.25 | YLQEVINVLETDGHFR |

| Tubulin beta-4A chain OS=Bos taurus GN=TUBB4A PE=2 SV=1 | 72 |
| --- | --- |

| 615.3029 | 1228.591 | 1228.591 | 56.05 | ISEQFTAMFR |
| --- | --- | --- | --- | --- |

| Tubulin beta-2B chain OS=Bos taurus GN=TUBB2B PE=1 SV=2 | 56 |
| --- | --- |

| 615.3029 | 1228.591 | 1228.591 | 56.05 | ISEQFTAMFR |
| --- | --- | --- | --- | --- |

| Extracellular matrix protein 2 OS=Bos taurus GN=ECM2 PE=2 SV=1 | 60 |
| --- | --- |

| 796.4615 | 1590.908 | 1590.909 | 51.93 | SLVHLVLIGNQIER | Deamidated (NQ) |
| --- | --- | --- | --- | --- | --- |

| Versican core protein OS=Bos taurus GN=VCAN PE=1 SV=2 | 56 |
| --- | --- |

| 657.8617 | 1313.709 | 1313.709 | 55.57 | LATVGELQAAWR |
| --- | --- | --- | --- | --- |

| Complement component C7 OS=Bos taurus GN=C7 PE=2 SV=1 | 56 |
| --- | --- |

| 735.4032 | 1468.792 | 1468.793 | 55.55 | LSGNILSYTFQVK |
| --- | --- | --- | --- | --- |

| Mimecan OS=Bos taurus GN=OGN PE=1 SV=2 | 55 |
| --- | --- |

| 609.686 | 1826.036 | 1826.04 | 40.53 | LSLLEELTLAENQLLK |
| --- | --- | --- | --- | --- |

| Tubulin alpha-3 chain OS=Bos taurus GN=TUBA3 PE=2 SV=1 | 51 |
| --- | --- |

| 729.4372 | 1456.86 | 1456.861 | 50.51 | LIGQIVSSITASLR |
| --- | --- | --- | --- | --- |

| Kininogen-1 OS=Bos taurus GN=KNG1 PE=1 SV=1 | 55 |
| --- | --- |

| 484.7811 | 967.5476 | 967.5491 | 55.03 | YSIVFIAR |  |
| --- | --- | --- | --- | --- | --- |

| Collagen alpha-1(III) chain OS=Bos taurus GN=COL3A1 PE=1 SV=1 | 54 |
| --- | --- |

| 1041.558 | 2081.101 | 2081.102 | 45.71 | GAPGPQGPPGAPGPLGIAGLTGAR | Oxidation (P) |
| --- | --- | --- | --- | --- | --- |

| Sorting nexin-4 OS=Bos taurus GN=SNX4 PE=2 SV=1 | 53 |
| --- | --- |

| 537.8181 | 1073.622 | 1073.623 | 53.35 | IGLENFLLR |
| --- | --- | --- | --- | --- |

| Dermatopontin OS=Bos taurus GN=DPT PE=1 SV=3 | 52 |
| --- | --- |

| 514.7552 | 1027.496 | 1027.498 | 54.7 | YFESVLDR |
| --- | --- | --- | --- | --- |

| Alkaline phosphatase, tissue-nonspecific isozyme OS=Bos taurus GN=ALPL PE=1 SV=2 | 52 |
| --- | --- |

| 547.8028 | 1093.591 | 1093.592 | 51.63 | GFFLLVEGGR |
| --- | --- | --- | --- | --- |

| Decorin OS=Bos taurus GN=DCN PE=1 SV=2 | 51 |
| --- | --- |

| 1382.175 | 2762.335 | 2762.334 | 40.4 | ASYSGVSLFSNPVQYWEIQPSTFR |
| --- | --- | --- | --- | --- |

| Integrin beta-6 OS=Bos taurus GN=ITGB6 PE=1 SV=1 | 48 |
| --- | --- |

| 492.2627 | 982.5108 | 982.5124 | 47.56 | LGFGSFVEK |
| --- | --- | --- | --- | --- |

| Apolipoprotein A-II OS=Bos taurus GN=APOA2 PE=1 SV=2 | 47 |
| --- | --- |

| 869.4565 | 1736.898 | 1736.899 | 51.99 | AGTDLLNFLSSFIDPK |
| --- | --- | --- | --- | --- |

| DnaJ homolog subfamily C member 3 OS=Bos taurus GN=DNAJC3 PE=1 SV=1 | 46 |
| --- | --- |

| 469.2619 | 936.5092 | 936.5103 | 46.42 | ATVFLAMGK |
| --- | --- | --- | --- | --- |

| Lysosomal alpha-mannosidase OS=Bos taurus GN=MAN2B1 PE=1 SV=3 | 46 |
| --- | --- |

| 542.8107 | 1083.607 | 1083.608 | 40.53 | WGPETLLLR |
| --- | --- | --- | --- | --- |

| Ezrin OS=Bos taurus GN=EZR PE=1 SV=2 | 45 |
| --- | --- |

| 591.8003 | 1181.586 | 1181.587 | 43.9 | APDFVFYAPR |
| --- | --- | --- | --- | --- |

| Moesin OS=Bos taurus GN=MSN PE=2 SV=3 | 45 |
| --- | --- |

| 591.8003 | 1181.586 | 1181.587 | 43.9 | APDFVFYAPR |
| --- | --- | --- | --- | --- |

| Radixin OS=Bos taurus GN=RDX PE=2 SV=1 | 45 |
| --- | --- |

| 591.8003 | 1181.586 | 1181.587 | 43.9 | APDFVFYAPR |
| --- | --- | --- | --- | --- |

| Alpha-actinin-4 OS=Bos taurus GN=ACTN4 PE=2 SV=1 | 44 |
| --- | --- |

| 608.3414 | 1214.668 | 1214.666 | 43.95 | LASDLLEWIR |
| --- | --- | --- | --- | --- |

| Alpha-actinin-1 OS=Bos taurus GN=ACTN1 PE=2 SV=1 | 44 |
| --- | --- |

| 608.3414 | 1214.668 | 1214.666 | 43.95 | LASDLLEWIR |
| --- | --- | --- | --- | --- |

| Fibrinogen beta chain OS=Bos taurus GN=FGB PE=1 SV=2 | 42 |
| --- | --- |

| 744.8998 | 1487.785 | 1487.787 | 42.03 | TSSSTFQYITLLK |
| --- | --- | --- | --- | --- |

| Gelsolin OS=Bos taurus GN=GSN PE=2 SV=1 | 41 |
| --- | --- |

| 861.922 | 1721.829 | 1721.83 | 41.22 | EVQGFESATFLGYFK |
| --- | --- | --- | --- | --- |

| Hyaluronan and proteoglycan link protein 1 OS=Bos taurus GN=HAPLN1 PE=2 SV=1 | | | | 40 | |  |
| --- | --- | --- | --- | --- | --- | --- |
| 510.2865 | 1018.558 | 1018.56 | 40.36 | | VGQIFAAWK | |

**NS2**

| Matrix Gla protein OS=Bos taurus GN=MGP PE=1 SV=1 | 169 |
| --- | --- |

| **pep_exp_mz** | **pep_exp_mr** | **pep_calc_mr** | **pep_score** | **pep_seq** | **pep_var_mod** |
| --- | --- | --- | --- | --- | --- |
| 778.8463 | 1555.678 | 1555.677 | 47.41 | YAMVYGYNAAYDR | |
| 778.8474 | 1555.68 | 1555.677 | 47.5 | YAMVYGYNAAYDR | |
| 786.8437 | 1571.673 | 1571.671 | 59.23 | YAMVYGYNAAYDR | Oxidation (M) |
| 787.335 | 1572.655 | 1572.656 | 64.3 | YAMVYGYNAAYDR | Deamidated (NQ); Oxidation (M) |
| 787.3356 | 1572.657 | 1572.656 | 65.11 | YAMVYGYNAAYDR | Deamidated (NQ); Oxidation (M) |

| Tubulin alpha-3 chain OS=Bos taurus GN=TUBA3 PE=2 SV=1 | 83 |
| --- | --- |

| 729.4387 | 1456.863 | 1456.861 | 82.53 | LIGQIVSSITASLR |
| --- | --- | --- | --- | --- |

| Tubulin alpha-1B chain OS=Bos taurus PE=1 SV=2 | 59 |
| --- | --- |

| 543.3134 | 1084.612 | 1084.613 | 45.43 | EIIDLVLDR |
| --- | --- | --- | --- | --- |

| Tubulin alpha-1C chain OS=Bos taurus GN=TUBA1C PE=1 SV=1 | 59 |
| --- | --- |

| 614.6746 | 1841.002 | 1841.005 | 45.3 | GHYTIGKEIIDLVLDR |
| --- | --- | --- | --- | --- |

| Osteomodulin OS=Bos taurus GN=OMD PE=1 SV=1 | 90 |
| --- | --- |

| 606.3246 | 1210.635 | 1210.635 | 55.75 | IFLGYNEISR | |
| --- | --- | --- | --- | --- | --- |
| 606.3253 | 1210.636 | 1210.635 | 54.67 | IFLGYNEISR | |
| 606.817 | 1211.619 | 1211.619 | 52.28 | IFLGYNEISR | Deamidated (NQ) |

| Tetranectin OS=Bos taurus GN=CLEC3B PE=2 SV=1 | 86 |
| --- | --- |

| 510.2916 | 1527.853 | 1527.851 | 42.11 | TQLDSLAQEVALLK |
| --- | --- | --- | --- | --- |
| 764.934 | 1527.853 | 1527.851 | 74.84 | TQLDSLAQEVALLK |

| Extracellular matrix protein 2 OS=Bos taurus GN=ECM2 PE=2 SV=1 | 86 |
| --- | --- |

| 795.9712 | 1589.928 | 1589.925 | 48.72 | SLVHLVLIGNQIER |
| --- | --- | --- | --- | --- |

| Fibromodulin OS=Bos taurus GN=FMOD PE=1 SV=2 | 84 |
| --- | --- |

| 519.9594 | 1556.856 | 1556.856 | 41.07 | SLILLDLSYNHLR | Deamidated (NQ) |
| --- | --- | --- | --- | --- | --- |
| 779.4363 | 1556.858 | 1556.856 | 82.81 | SLILLDLSYNHLR | Deamidated (NQ) |

| Versican core protein OS=Bos taurus GN=VCAN PE=1 SV=2 | 76 |
| --- | --- |

| 657.8621 | 1313.71 | 1313.709 | 75.56 | LATVGELQAAWR |
| --- | --- | --- | --- | --- |

| Apolipoprotein A-II OS=Bos taurus GN=APOA2 PE=1 SV=2 | 74 |
| --- | --- |

| 579.9733 | 1736.898 | 1736.899 | 46.08 | AGTDLLNFLSSFIDPK |
| --- | --- | --- | --- | --- |
| 869.459 | 1736.903 | 1736.899 | 56.96 | AGTDLLNFLSSFIDPK |

| Mimecan OS=Bos taurus GN=OGN PE=1 SV=2 | 66 |
| --- | --- |

| 520.3114 | 1038.608 | 1038.607 | 47.93 | LEGNPVILGK |
| --- | --- | --- | --- | --- |

| DnaJ homolog subfamily C member 3 OS=Bos taurus GN=DNAJC3 PE=1 SV=1 | 64 |
| --- | --- |

| 469.2625 | 936.5104 | 936.5103 | 64.23 | ATVFLAMGK |
| --- | --- | --- | --- | --- |

| Bone sialoprotein 2 OS=Bos taurus GN=IBSP PE=1 SV=1 | 64 |
| --- | --- |

| 548.2852 | 1094.556 | 1094.555 | 44.71 | HGYFYPALK |
| --- | --- | --- | --- | --- |

| Gelsolin OS=Bos taurus GN=GSN PE=2 SV=1 | 62 |
| --- | --- |

| 861.9247 | 1721.835 | 1721.83 | 62.33 | EVQGFESATFLGYFK |
| --- | --- | --- | --- | --- |

| Clusterin OS=Bos taurus GN=CLU PE=1 SV=1 | 62 |
| --- | --- |

| 763.8968 | 1525.779 | 1525.778 | 61.68 | LYDQLLQSYQQK |
| --- | --- | --- | --- | --- |

| Procollagen-lysine,2-oxoglutarate 5-dioxygenase 1 OS=Bos taurus GN=PLOD1 PE=2 SV=2 | 61 |
| --- | --- |

| 814.4294 | 1626.844 | 1626.841 | 61.46 | FLGSGGFIGYAPNLSK |
| --- | --- | --- | --- | --- |

| Insulin-like growth factor-binding protein 6 OS=Bos taurus GN=IGFBP6 PE=2 SV=2 | 60 |
| --- | --- |

| 906.9849 | 1811.955 | 1811.953 | 60 | HLDSVLQQLQTEVFR |
| --- | --- | --- | --- | --- |

| Tubulin beta-4B chain OS=Bos taurus GN=TUBB4B PE=2 SV=1 | 60 |
| --- | --- |

| 520.3004 | 1038.586 | 1038.586 | 59.9 | YLTVAAVFR |
| --- | --- | --- | --- | --- |

| Tubulin beta-5 chain OS=Bos taurus GN=TUBB5 PE=2 SV=1 | 60 |
| --- | --- |

| 520.3004 | 1038.586 | 1038.586 | 59.9 | YLTVAAVFR |
| --- | --- | --- | --- | --- |

| Actin, cytoplasmic 1 OS=Bos taurus GN=ACTB PE=1 SV=1 | 60 |
| --- | --- |

| 566.7675 | 1131.52 | 1131.52 | 59.54 | GYSFTTTAER |
| --- | --- | --- | --- | --- |

| Actin, cytoplasmic 2 OS=Bos taurus GN=ACTG1 PE=1 SV=1 | 60 |
| --- | --- |

| 566.7675 | 1131.52 | 1131.52 | 59.54 | GYSFTTTAER |
| --- | --- | --- | --- | --- |

| Decorin OS=Bos taurus GN=DCN PE=1 SV=2 | 59 |
| --- | --- |

| 1382.175 | 2762.335 | 2762.334 | 45.93 | ASYSGVSLFSNPVQYWEIQPSTFR |
| --- | --- | --- | --- | --- |

| Vimentin OS=Bos taurus GN=VIM PE=1 SV=3 | 59 |
| --- | --- |

| 585.3603 | 1168.706 | 1168.707 | 58.81 | ILLAELEQLK |
| --- | --- | --- | --- | --- |

| Elongation factor 1-gamma OS=Bos taurus GN=EEF1G PE=2 SV=1 | 58 |
| --- | --- |

| 364.8978 | 1091.672 | 1091.67 | 45.52 | ILGLLDAHLK |
| --- | --- | --- | --- | --- |

| Complement factor D OS=Bos taurus GN=CFD PE=2 SV=1 | 55 |
| --- | --- |

| 774.457 | 1546.899 | 1546.898 | 55.28 | AVLGPAVQLLPWQR |
| --- | --- | --- | --- | --- |

| Glycosyltransferase 8 domain-containing protein 2 OS=Bos taurus GN=GLT8D2 PE=2 SV=1 | 53 |
| --- | --- |

| 579.3502 | 1156.686 | 1156.686 | 53.22 | IVEFNPVVLK |
| --- | --- | --- | --- | --- |

| Protein disulfide-isomerase OS=Bos taurus GN=P4HB PE=1 SV=1 | 53 |
| --- | --- |

| 547.8339 | 1093.653 | 1093.654 | 46.86 | ILEFFGLKK |
| --- | --- | --- | --- | --- |

| Collagen alpha-1(XI) chain (Fragment) OS=Bos taurus GN=COL11A1 PE=1 SV=1 | 52 |
| --- | --- |

| 615.8279 | 1229.641 | 1229.641 | 42.23 | LGVPGLPGYPGR | 3 Oxidation (P) |
| --- | --- | --- | --- | --- | --- |

| Fibrinogen alpha chain OS=Bos taurus GN=FGA PE=1 SV=5 | 50 |
| --- | --- |

| 531.2864 | 1060.558 | 1060.559 | 50.06 | ALLEMQQTK |
| --- | --- | --- | --- | --- |

| Ribonuclease 4 OS=Bos taurus GN=RNASE4 PE=1 SV=4 | 48 |
| --- | --- |

| 852.9295 | 1703.844 | 1703.842 | 48.45 | FNTFIHEDLWNIR |
| --- | --- | --- | --- | --- |

| Complement C1q subcomponent subunit B OS=Bos taurus GN=C1QB PE=1 SV=1 | 47 |
| --- | --- |

| 822.9152 | 1643.816 | 1643.81 | 41.37 | VPGLYFFTYHASSR |
| --- | --- | --- | --- | --- |

| Moesin OS=Bos taurus GN=MSN PE=2 SV=3 | 46 |
| --- | --- |

| 591.8008 | 1181.587 | 1181.587 | 49.62 | APDFVFYAPR |
| --- | --- | --- | --- | --- |

| Ezrin OS=Bos taurus GN=EZR PE=1 SV=2 | 46 |
| --- | --- |

| 591.8008 | 1181.587 | 1181.587 | 49.62 | APDFVFYAPR |
| --- | --- | --- | --- | --- |

| Radixin OS=Bos taurus GN=RDX PE=2 SV=1 | 46 |
| --- | --- |

| 591.8008 | 1181.587 | 1181.587 | 49.62 | APDFVFYAPR |
| --- | --- | --- | --- | --- |

| 60S acidic ribosomal protein P2 OS=Bos taurus GN=RPLP2 PE=3 SV=1 | 46 |
| --- | --- |

| 628.8453 | 1255.676 | 1255.677 | 45.69 | NIEDVIAQGIGK |
| --- | --- | --- | --- | --- |

| Kininogen-1 OS=Bos taurus GN=KNG1 PE=1 SV=1 | 44 |
| --- | --- |

| 484.7821 | 967.5496 | 967.5491 | 44.49 | YSIVFIAR |  |
| --- | --- | --- | --- | --- | --- |

| Fibulin-5 OS=Bos taurus GN=FBLN5 PE=2 SV=1 | 44 |
| --- | --- |

| 681.8585 | 1361.702 | 1361.702 | 44.06 | YPGAYYIFQIK |
| --- | --- | --- | --- | --- |

| Alpha-S1-casein OS=Bos taurus GN=CSN1S1 PE=1 SV=2 | 43 |
| --- | --- |

| 634.3549 | 1266.695 | 1266.697 | 43.44 | YLGYLEQLLR |
| --- | --- | --- | --- | --- |

| Peptidyl-prolyl cis-trans isomerase B OS=Bos taurus GN=PPIB PE=1 SV=4 | 43 |
| --- | --- |

| 682.8587 | 1363.703 | 1363.698 | 43.01 | TVDNFVALATGEK |
| --- | --- | --- | --- | --- |

| Coagulation factor X OS=Bos taurus GN=F10 PE=1 SV=1 | 42 |
| --- | --- |

| 447.2455 | 892.4764 | 892.4767 | 42.44 | TGIVSGFGR |
| --- | --- | --- | --- | --- |

| Vitamin K-dependent protein C (Fragment) OS=Bos taurus GN=PROC PE=1 SV=1 | 42 |
| --- | --- |

| 480.7399 | 959.4652 | 959.4647 | 42.31 | FCLHEVR |  |
| --- | --- | --- | --- | --- | --- |

| Bone morphogenetic protein 3 OS=Bos taurus GN=BMP3 PE=1 SV=2 | 41 |
| --- | --- |

| 551.309 | 1650.905 | 1650.905 | 41.02 | SLKPSNHATIQSIVR | Deamidated (NQ) |
| --- | --- | --- | --- | --- | --- |

| Cadherin-2 (Fragment) OS=Bos taurus GN=CDH2 PE=2 SV=1 | 40 |
| --- | --- |

| 662.8774 | 1323.74 | 1323.74 | 40.05 | DVLEGQPLLNVK |
| --- | --- | --- | --- | --- |

**WK1**

| Collagen alpha-1(III) chain OS=Bos taurus GN=COL3A1 PE=1 SV=1 | 56 |
| --- | --- |

| **pep_exp_mz** | **pep_exp_mr** | **pep_calc_mr** | **pep_score** | **pep_seq** | **pep_var_mod** |
| --- | --- | --- | --- | --- | --- |
| 645.2968 | 1288.579 | 1288.58 | 55.89 | GSPGGPGAAGFPGGR | 3 Oxidation (P) |

**AY2**

| Collagen alpha-1(II) chain OS=Bos taurus GN=COL2A1 PE=1 SV=4 | 137 |
| --- | --- |

| **pep_exp_mz** | **pep_exp_mr** | **pep_calc_mr** | **pep_score** | **pep_seq** | **pep_var_mod** |
| --- | --- | --- | --- | --- | --- |
| 664.8278 | 1327.641 | 1327.641 | 49.75 | GFPGLPGPSGEPGK | 2 Oxidation (P) |
| 664.8279 | 1327.641 | 1327.641 | 58.48 | GFPGLPGPSGEPGK | 2 Oxidation (P) |
| 664.8283 | 1327.642 | 1327.641 | 53.37 | GFPGLPGPSGEPGK | 2 Oxidation (P) |
| 672.8252 | 1343.636 | 1343.636 | 43.42 | GFPGLPGPSGEPGK | 3 Oxidation (P) |
| 672.8253 | 1343.636 | 1343.636 | 52.9 | GFPGLPGPSGEPGK | 3 Oxidation (P) |
| 672.8255 | 1343.636 | 1343.636 | 59.85 | GFPGLPGPSGEPGK | 3 Oxidation (P) |

| Collagen alpha-1(XI) chain (Fragment) OS=Bos taurus GN=COL11A1 PE=1 SV=1 | 54 |
| --- | --- |

| 1144.563 | 2287.111 | 2287.108 | 53.62 | TGPPGPGGVVGPQGPTGETGPIGER | Deamidated (NQ); Oxidation (P) |
| --- | --- | --- | --- | --- | --- |

| Biglycan OS=Bos taurus GN=BGN PE=1 SV=3 | 48 |
| --- | --- |

| 657.369 | 1312.723 | 1312.724 | 47.85 | IQAIELEDLLR | Deamidated (NQ) |
| --- | --- | --- | --- | --- | --- |

| Alpha-S1-casein OS=Bos taurus GN=CSN1S1 PE=1 SV=2 | 42 |
| --- | --- |

| 692.869 | 1383.723 | 1383.723 | 41.79 | FFVAPFPEVFGK |
| --- | --- | --- | --- | --- |

**AY5**

| Alpha-S1-casein OS=Bos taurus GN=CSN1S1 PE=1 SV=2 | 45 |
| --- | --- |

| **pep_exp_mz** | **pep_exp_mr** | **pep_calc_mr** | **pep_score** | **pep_seq** | **pep_var_mod** |
| --- | --- | --- | --- | --- | --- |
| 692.8679 | 1383.721 | 1383.723 | 44.74 | FFVAPFPEVFGK | |

**HSB3**

| Actin, cytoplasmic 1 OS=Bos taurus GN=ACTB PE=1 SV=1 | 80 |
| --- | --- |

| **pep_exp_mz** | **pep_exp_mr** | **pep_calc_mr** | **pep_score** | **pep_seq** | **pep_var_mod** |
| --- | --- | --- | --- | --- | --- |
| 1275.589 | 2549.163 | 2549.167 | 79.66 | LCYVALDFEQEMATAASSSSLEK | |

| Vimentin OS=Bos taurus GN=VIM PE=1 SV=3 | 51 |
| --- | --- |

| 709.3596 | 2125.057 | 2125.058 | 51.06 | LLQDSVDFSLADAINTEFK |
| --- | --- | --- | --- | --- |

**TSH**

| Antithrombin-III OS=Bos taurus GN=SERPINC1 PE=1 SV=2 | 50 |
| --- | --- |

| **pep_exp_mz** | **pep_exp_mr** | **pep_calc_mr** | **pep_score** | **pep_seq** | **pep_var_mod** |
| --- | --- | --- | --- | --- | --- |
| 690.864 | 1379.713 | 1379.712 | 49.55 | EVALNTIIFMGR | Deamidated (NQ); Oxidation (M) |

| Biglycan OS=Bos taurus GN=BGN PE=1 SV=3 | 47 |
| --- | --- |

| 657.3701 | 1312.726 | 1312.724 | 47.37 | IQAIELEDLLR | Deamidated (NQ) |
| --- | --- | --- | --- | --- | --- |

| Collagen alpha-2(XI) chain OS=Bos taurus GN=COL11A2 PE=3 SV=1 | 40 |
| --- | --- |

| 369.693 | 737.3714 | 737.3708 | 40.08 | GPPGPPGK | 2 Oxidation (P) |
| --- | --- | --- | --- | --- | --- |

| Collagen alpha-1(XI) chain (Fragment) OS=Bos taurus GN=COL11A1 PE=1 SV=1 | 40 |
| --- | --- |

| 369.693 | 737.3714 | 737.3708 | 40.08 | GPPGPPGK | 2 Oxidation (P) |
| --- | --- | --- | --- | --- | --- |

**UI3**

| Decorin OS=Bos taurus GN=DCN PE=1 SV=2 | 100 |
| --- | --- |

| **pep_exp_mz** | **pep_exp_mr** | **pep_calc_mr** | **pep_score** | **pep_seq** | **pep_var_mod** |
| --- | --- | --- | --- | --- | --- |
| 796.0671 | 2385.18 | 2385.181 | 54.29 | LGLSFNSISAVDNGSLANTPHLR | 3 Deamidated (NQ) |
| 796.0673 | 2385.18 | 2385.181 | 56.43 | LGLSFNSISAVDNGSLANTPHLR | 3 Deamidated (NQ) |

| Thrombospondin-1 OS=Bos taurus GN=THBS1 PE=2 SV=2 | 98 |
| --- | --- |

| 429.7553 | 857.496 | 857.4971 | 55.63 | GTLLAVER |
| --- | --- | --- | --- | --- |
| 495.3102 | 988.6058 | 988.6069 | 54.32 | GFLLLASLR |
| 495.3108 | 988.607 | 988.6069 | 54.56 | GFLLLASLR |

| Nucleobindin-1 OS=Bos taurus GN=NUCB1 PE=2 SV=1 | 91 |
| --- | --- |

| 636.8616 | 1271.709 | 1271.709 | 88.45 | DLELLIQTATR |
| --- | --- | --- | --- | --- |

| Matrix Gla protein OS=Bos taurus GN=MGP PE=1 SV=1 | 77 |
| --- | --- |

| 786.8425 | 1571.67 | 1571.671 | 49.82 | YAMVYGYNAAYDR | Oxidation (M) |
| --- | --- | --- | --- | --- | --- |
| 787.3347 | 1572.655 | 1572.656 | 58.85 | YAMVYGYNAAYDR | Deamidated (NQ); Oxidation (M) |

| Antithrombin-III OS=Bos taurus GN=SERPINC1 PE=1 SV=2 | 72 |
| --- | --- |

| 690.8631 | 1379.712 | 1379.712 | 67.09 | EVALNTIIFMGR | Deamidated (NQ); Oxidation (M) |
| --- | --- | --- | --- | --- | --- |

| Bone sialoprotein 2 OS=Bos taurus GN=IBSP PE=1 SV=1 | 71 |
| --- | --- |

| 548.2842 | 1094.554 | 1094.555 | 40.28 | HGYFYPALK |
| --- | --- | --- | --- | --- |
| 417.8923 | 1250.655 | 1250.656 | 42.02 | HGYFYPALKR |

| Apolipoprotein A-I OS=Bos taurus GN=APOA1 PE=1 SV=3 | 71 |
| --- | --- |

| 608.8427 | 1215.671 | 1215.671 | 70.67 | VSILAAIDEASK |
| --- | --- | --- | --- | --- |

| Olfactomedin-like protein 3 OS=Bos taurus GN=OLFML3 PE=2 SV=1 | 68 |
| --- | --- |

| 517.2753 | 1032.536 | 1032.539 | 62.77 | FGGPAGLWTK |
| --- | --- | --- | --- | --- |

| Vitamin D-binding protein OS=Bos taurus GN=GC PE=2 SV=1 | 67 |
| --- | --- |

| 692.3589 | 1382.703 | 1382.708 | 54.76 | VLDQYIFELSR | Deamidated (NQ) |
| --- | --- | --- | --- | --- | --- |

| Osteomodulin OS=Bos taurus GN=OMD PE=1 SV=1 | 66 |
| --- | --- |

| 606.324 | 1210.633 | 1210.635 | 52.27 | IFLGYNEISR | |
| --- | --- | --- | --- | --- | --- |
| 606.8163 | 1211.618 | 1211.619 | 41.91 | IFLGYNEISR | Deamidated (NQ) |
| 606.8164 | 1211.618 | 1211.619 | 43.12 | IFLGYNEISR | Deamidated (NQ) |

| Alkaline phosphatase, tissue-nonspecific isozyme OS=Bos taurus GN=ALPL PE=1 SV=2 | 59 |
| --- | --- |

| 547.803 | 1093.591 | 1093.592 | 58.75 | GFFLLVEGGR |
| --- | --- | --- | --- | --- |

| Bone morphogenetic protein 3 OS=Bos taurus GN=BMP3 PE=1 SV=2 | 58 |
| --- | --- |

| 931.9696 | 1861.925 | 1861.925 | 57.56 | VDFADIGWSEWIISPK |
| --- | --- | --- | --- | --- |

| Vitrin OS=Bos taurus GN=VIT PE=2 SV=2 | 57 |
| --- | --- |

| 516.2984 | 1030.582 | 1030.585 | 56.92 | LMILITDGR |
| --- | --- | --- | --- | --- |

| Secreted phosphoprotein 24 OS=Bos taurus GN=SPP2 PE=1 SV=2 | 57 |
| --- | --- |

| 706.8562 | 1411.698 | 1411.698 | 56.53 | VNSQSLSPYLFR | 2 Deamidated (NQ) |
| --- | --- | --- | --- | --- | --- |

| Collagen alpha-1(XI) chain (Fragment) OS=Bos taurus GN=COL11A1 PE=1 SV=1 | 52 |
| --- | --- |

| 1144.068 | 2286.121 | 2286.124 | 43.48 | TGPPGPGGVVGPQGPTGETGPIGER | Oxidation (P) |
| --- | --- | --- | --- | --- | --- |

| Carboxypeptidase E OS=Bos taurus GN=CPE PE=1 SV=2 | 51 |
| --- | --- |

| 844.9459 | 1687.877 | 1687.872 | 51.06 | EALVSVWLQCAAVSR |
| --- | --- | --- | --- | --- |

| Coagulation factor VII OS=Bos taurus GN=F7 PE=1 SV=2 | 50 |
| --- | --- |

| 725.8705 | 1449.726 | 1449.725 | 50.35 | FSAVSGWGQLLER | Deamidated (NQ) |
| --- | --- | --- | --- | --- | --- |

| Lysosomal alpha-mannosidase OS=Bos taurus GN=MAN2B1 PE=1 SV=3 | 50 |
| --- | --- |

| 653.3691 | 1304.724 | 1304.724 | 50.15 | FQVIVYNPLGR |
| --- | --- | --- | --- | --- |

| Collagen alpha-2(XI) chain OS=Bos taurus GN=COL11A2 PE=3 SV=1 | 48 |
| --- | --- |

| 511.2868 | 1020.559 | 1020.56 | 50.04 | DFSLLTAVR |
| --- | --- | --- | --- | --- |

| Coagulation factor IX (Fragment) OS=Bos taurus GN=F9 PE=1 SV=1 | 47 |
| --- | --- |

| 610.7902 | 1219.566 | 1219.566 | 47.23 | FGYGYVSGWGK |
| --- | --- | --- | --- | --- |

| Vitamin K-dependent protein C (Fragment) OS=Bos taurus GN=PROC PE=1 SV=1 | 44 |
| --- | --- |

| 477.7864 | 953.5582 | 953.5586 | 43.76 | TFVLSFIK |  |
| --- | --- | --- | --- | --- | --- |

| Apolipoprotein A-II OS=Bos taurus GN=APOA2 PE=1 SV=2 | 43 |
| --- | --- |

| 869.457 | 1736.899 | 1736.899 | 43.02 | AGTDLLNFLSSFIDPK |
| --- | --- | --- | --- | --- |

| Tubulin beta-4A chain OS=Bos taurus GN=TUBB4A PE=2 SV=1 | 42 |
| --- | --- |

| 520.2996 | 1038.585 | 1038.586 | 41.76 | YLTVAAVFR |
| --- | --- | --- | --- | --- |

| Tubulin beta-4B chain OS=Bos taurus GN=TUBB4B PE=2 SV=1 | 42 |
| --- | --- |

| 520.2996 | 1038.585 | 1038.586 | 41.76 | YLTVAAVFR |
| --- | --- | --- | --- | --- |

| Tubulin beta-5 chain OS=Bos taurus GN=TUBB5 PE=2 SV=1 | 42 |
| --- | --- |

| 520.2996 | 1038.585 | 1038.586 | 41.76 | YLTVAAVFR |
| --- | --- | --- | --- | --- |

| Tubulin beta-6 chain OS=Bos taurus GN=TUBB6 PE=2 SV=1 | 42 |
| --- | --- |

| 520.2996 | 1038.585 | 1038.586 | 41.76 | YLTVAAVFR |
| --- | --- | --- | --- | --- |

| Inactive hydroxysteroid dehydrogenase-like protein 1 OS=Bos taurus GN=HSDL1 PE=2 SV=1 | 41 |
| --- | --- |

| 493.3051 | 984.5956 | 984.5968 | 41.39 | GLNIVLISR | Deamidated (NQ) |
| --- | --- | --- | --- | --- | --- |

| Collagen alpha-1(III) chain OS=Bos taurus GN=COL3A1 PE=1 SV=1 | 40 |
| --- | --- |

| 645.296 | 1288.577 | 1288.58 | 41.97 | GSPGGPGAAGFPGGR | 3 Oxidation (P) |
| --- | --- | --- | --- | --- | --- |

**GuHCl Batch 3**

| Collagen alpha-1(XI) chain (Fragment) OS=Bos taurus GN=COL11A1 PE=1 SV=1 | 128 |
| --- | --- |

| **pep_exp_mz** | **pep_exp_mr** | **pep_calc_mr** | **pep_score** | **pep_seq** | **pep_var_mod** |
| --- | --- | --- | --- | --- | --- |
| 1144.071 | 2286.127 | 2286.124 | 67.14 | TGPPGPGGVVGPQGPTGETGPIGER | Oxidation (P) |
| 1144.564 | 2287.113 | 2287.108 | 63.64 | TGPPGPGGVVGPQGPTGETGPIGER | Deamidated (NQ); Oxidation (P) |

| Osteomodulin OS=Bos taurus GN=OMD PE=1 SV=1 | 119 |
| --- | --- |

| 606.3244 | 1210.634 | 1210.635 | 56.52 | IFLGYNEISR | |
| --- | --- | --- | --- | --- | --- |
| 606.8159 | 1211.617 | 1211.619 | 44.28 | IFLGYNEISR | Deamidated (NQ) |
| 606.816 | 1211.617 | 1211.619 | 44.08 | IFLGYNEISR | Deamidated (NQ) |
| 606.8163 | 1211.618 | 1211.619 | 54.34 | IFLGYNEISR | Deamidated (NQ) |

| Monocyte differentiation antigen CD14 OS=Bos taurus GN=CD14 PE=2 SV=2 | 96 |
| --- | --- |

| 810.0035 | 1617.992 | 1617.993 | 67.1 | LGAAQVPAQLLVAVLR |
| --- | --- | --- | --- | --- |

| Fibromodulin OS=Bos taurus GN=FMOD PE=1 SV=2 | 88 |
| --- | --- |

| 779.4343 | 1556.854 | 1556.856 | 67.05 | SLILLDLSYNHLR | Deamidated (NQ) |
| --- | --- | --- | --- | --- | --- |
| 779.4353 | 1556.856 | 1556.856 | 58.43 | SLILLDLSYNHLR | Deamidated (NQ) |

| Vitamin D-binding protein OS=Bos taurus GN=GC PE=2 SV=1 | 86 |
| --- | --- |

| 691.8694 | 1381.724 | 1381.724 | 78.51 | VLDQYIFELSR |
| --- | --- | --- | --- | --- |

| Hemoglobin fetal subunit beta OS=Bos taurus PE=1 SV=1 | 80 |
| --- | --- |

| 633.4182 | 1264.822 | 1264.823 | 80.24 | LLGNVLVVVLAR |
| --- | --- | --- | --- | --- |

| Hemoglobin subunit beta OS=Bos taurus GN=HBB PE=1 SV=1 | 80 |
| --- | --- |

| 633.4182 | 1264.822 | 1264.823 | 80.24 | LLGNVLVVVLAR |
| --- | --- | --- | --- | --- |

| Actin, alpha cardiac muscle 1 OS=Bos taurus GN=ACTC1 PE=2 SV=1 | 58 |
| --- | --- |

| 505.921 | 1514.741 | 1514.742 | 52.51 | IWHHTFYNELR | |
| --- | --- | --- | --- | --- | --- |
| 506.2479 | 1515.722 | 1515.726 | 44.49 | IWHHTFYNELR | Deamidated (NQ) |

| Actin, alpha skeletal muscle OS=Bos taurus GN=ACTA1 PE=1 SV=1 | 58 |
| --- | --- |

| 505.921 | 1514.741 | 1514.742 | 52.51 | IWHHTFYNELR | |
| --- | --- | --- | --- | --- | --- |
| 506.2479 | 1515.722 | 1515.726 | 44.49 | IWHHTFYNELR | Deamidated (NQ) |

| Vitamin K-dependent protein S OS=Bos taurus GN=PROS1 PE=1 SV=1 | 72 |
| --- | --- |

| 829.9609 | 1657.907 | 1657.907 | 51.33 | LQDILVSVESMVIGR |
| --- | --- | --- | --- | --- |

| Complement C4 (Fragments) OS=Bos taurus GN=C4 PE=1 SV=2 | 72 |
| --- | --- |

| 684.3633 | 1366.712 | 1366.713 | 61.78 | DSSTWLTAFVLK |
| --- | --- | --- | --- | --- |

| Secreted phosphoprotein 24 OS=Bos taurus GN=SPP2 PE=1 SV=2 | 66 |
| --- | --- |

| 705.8712 | 1409.728 | 1409.73 | 40.7 | VNSQSLSPYLFR | |
| --- | --- | --- | --- | --- | --- |
| 706.8568 | 1411.699 | 1411.698 | 53.26 | VNSQSLSPYLFR | 2 Deamidated (NQ) |

| Moesin OS=Bos taurus GN=MSN PE=2 SV=3 | 65 |
| --- | --- |

| 591.7998 | 1181.585 | 1181.587 | 64.77 | APDFVFYAPR |
| --- | --- | --- | --- | --- |

| Radixin OS=Bos taurus GN=RDX PE=2 SV=1 | 65 |
| --- | --- |

| 591.7998 | 1181.585 | 1181.587 | 64.77 | APDFVFYAPR |
| --- | --- | --- | --- | --- |

| Ezrin OS=Bos taurus GN=EZR PE=1 SV=2 | 65 |
| --- | --- |

| 591.7998 | 1181.585 | 1181.587 | 64.77 | APDFVFYAPR |
| --- | --- | --- | --- | --- |

| Apolipoprotein A-II OS=Bos taurus GN=APOA2 PE=1 SV=2 | 64 |
| --- | --- |

| 869.4578 | 1736.901 | 1736.899 | 63.88 | AGTDLLNFLSSFIDPK |
| --- | --- | --- | --- | --- |

| Complement component C7 OS=Bos taurus GN=C7 PE=2 SV=1 | 61 |
| --- | --- |

| 735.8948 | 1469.775 | 1469.777 | 58.66 | LSGNILSYTFQVK | Deamidated (NQ) |
| --- | --- | --- | --- | --- | --- |

| Matrix Gla protein OS=Bos taurus GN=MGP PE=1 SV=1 | 57 |
| --- | --- |

| 787.3361 | 1572.658 | 1572.656 | 57.38 | YAMVYGYNAAYDR | Deamidated (NQ); Oxidation (M) |
| --- | --- | --- | --- | --- | --- |

| Coagulation factor IX (Fragment) OS=Bos taurus GN=F9 PE=1 SV=1 | 57 |
| --- | --- |

| 511.7972 | 1021.58 | 1021.581 | 44.71 | SASILQYLK | |
| --- | --- | --- | --- | --- | --- |
| 512.2892 | 1022.564 | 1022.565 | 49.53 | SASILQYLK | Deamidated (NQ) |

| Complement factor B OS=Bos taurus GN=CFB PE=1 SV=2 | 54 |
| --- | --- |

| 621.3233 | 1240.632 | 1240.634 | 53.67 | YGLVTYATEPK |
| --- | --- | --- | --- | --- |

| Gelsolin OS=Bos taurus GN=GSN PE=2 SV=1 | 53 |
| --- | --- |

| 436.7635 | 871.5124 | 871.5127 | 53.25 | TGALELLR |  |
| --- | --- | --- | --- | --- | --- |

| Complement C1q subcomponent subunit B OS=Bos taurus GN=C1QB PE=1 SV=1 | 53 |
| --- | --- |

| 822.9128 | 1643.811 | 1643.81 | 45.63 | VPGLYFFTYHASSR |
| --- | --- | --- | --- | --- |

| Lysosomal alpha-mannosidase OS=Bos taurus GN=MAN2B1 PE=1 SV=3 | 50 |
| --- | --- |

| 653.3688 | 1304.723 | 1304.724 | 50.43 | FQVIVYNPLGR |
| --- | --- | --- | --- | --- |

| Vitamin K-dependent protein C (Fragment) OS=Bos taurus GN=PROC PE=1 SV=1 | 50 |
| --- | --- |

| 654.3425 | 1306.67 | 1306.671 | 46.69 | YLDWIYGHIK |
| --- | --- | --- | --- | --- |

| Unconventional myosin-Id OS=Bos taurus GN=MYO1D PE=2 SV=1 | 48 |
| --- | --- |

| 540.7851 | 1079.556 | 1079.559 | 47.72 | LFCWIVSR |
| --- | --- | --- | --- | --- |

| Mammalian ependymin-related protein 1 OS=Bos taurus GN=EPDR1 PE=2 SV=1 | 45 |
| --- | --- |

| 601.3469 | 1200.679 | 1200.679 | 44.81 | LFEYILLYK |  |
| --- | --- | --- | --- | --- | --- |

| Fibronectin OS=Bos taurus GN=FN1 PE=1 SV=4 | 45 |
| --- | --- |

| 964.0244 | 1926.034 | 1926.036 | 46.4 | FLATTPNSLLVSWQPPR |
| --- | --- | --- | --- | --- |

| Adiponectin OS=Bos taurus GN=ADIPOQ PE=1 SV=1 | 43 |
| --- | --- |

| 483.2556 | 964.4966 | 964.4978 | 43.31 | SAFSVGLER |
| --- | --- | --- | --- | --- |

| Serotransferrin OS=Bos taurus GN=TF PE=2 SV=1 | 43 |
| --- | --- |

| 460.284 | 918.5534 | 918.5538 | 42.66 | GYLAVAVVK |
| --- | --- | --- | --- | --- |

| Complement component C6 OS=Bos taurus GN=C6 PE=2 SV=1 | 40 |
| --- | --- |

| 985.5336 | 1969.053 | 1969.052 | 40.49 | ENPSVIDFALAPITDLVR |
| --- | --- | --- | --- | --- |

| Collagen alpha-1(III) chain OS=Bos taurus GN=COL3A1 PE=1 SV=1 | 36 |
| --- | --- |

| 705.3713 | 2113.092 | 2113.092 | 42 | GAPGPQGPPGAPGPLGIAGLTGAR | 3 Oxidation (P) |
| --- | --- | --- | --- | --- | --- |

**KC4**

| ATP synthase subunit beta, mitochondrial OS=Bos taurus GN=ATP5B PE=1 SV=2 | 86 |
| --- | --- |

| **pep_exp_mz** | **pep_exp_mr** | **pep_calc_mr** | **pep_score** | **pep_seq** | **pep_var_mod** |
| --- | --- | --- | --- | --- | --- |
| 720.3996 | 1438.785 | 1438.782 | 86.21 | VALTGLTVAEYFR | |

| Glyceraldehyde-3-phosphate dehydrogenase OS=Bos taurus GN=GAPDH PE=1 SV=4 | 72 |
| --- | --- |

| 778.9091 | 1555.804 | 1555.803 | 71.68 | VPTPNVSVVDLTCR |
| --- | --- | --- | --- | --- |

| Collagen alpha-1(XI) chain (Fragment) OS=Bos taurus GN=COL11A1 PE=1 SV=1 | 50 |
| --- | --- |

| 369.6932 | 737.3718 | 737.3708 | 51.83 | GPPGPPGK | 2 Oxidation (P) |
| --- | --- | --- | --- | --- | --- |

| Collagen alpha-2(XI) chain OS=Bos taurus GN=COL11A2 PE=3 SV=1 | 48 |
| --- | --- |

| 369.6932 | 737.3718 | 737.3708 | 51.83 | GPPGPPGK | 2 Oxidation (P) |
| --- | --- | --- | --- | --- | --- |

**WR6**

| Collagen alpha-1(II) chain OS=Bos taurus GN=COL2A1 PE=1 SV=4 | 111 |
| --- | --- |

| **pep_exp_mz** | **pep_exp_mr** | **pep_calc_mr** | **pep_score** | **pep_seq** | **pep_var_mod** |
| --- | --- | --- | --- | --- | --- |
| 664.8278 | 1327.641 | 1327.641 | 47.51 | GFPGLPGPSGEPGK | 2 Oxidation (P) |
| 664.8279 | 1327.641 | 1327.641 | 42.87 | GFPGLPGPSGEPGK | 2 Oxidation (P) |
| 664.8281 | 1327.642 | 1327.641 | 45.27 | GFPGLPGPSGEPGK | 2 Oxidation (P) |

**WRC10**

| Collagen alpha-1(II) chain OS=Bos taurus GN=COL2A1 PE=1 SV=4 | 84 |
| --- | --- |

| **pep_exp_mz** | **pep_exp_mr** | **pep_calc_mr** | **pep_score** | **pep_seq** | **pep_var_mod** |
| --- | --- | --- | --- | --- | --- |
| 664.8278 | 1327.641 | 1327.641 | 55.74 | GFPGLPGPSGEPGK | 2 Oxidation (P) |
| 664.8279 | 1327.641 | 1327.641 | 47.98 | GFPGLPGPSGEPGK | 2 Oxidation (P) |

| Serum albumin OS=Bos taurus GN=ALB PE=1 SV=4 | 41 |
| --- | --- |

| 740.4019 | 1478.789 | 1478.788 | 41.39 | LGEYGFQNALIVR |
| --- | --- | --- | --- | --- |

**WR15**

| Alpha-2-HS-glycoprotein OS=Bos taurus GN=AHSG PE=1 SV=2 | 106 |
| --- | --- |

| **pep_exp_mz** | **pep_exp_mr** | **pep_calc_mr** | **pep_score** | **pep_seq** | **pep_var_mod** |
| --- | --- | --- | --- | --- | --- |
| 1260.167 | 2518.319 | 2518.314 | 92.32 | AQFVPLPVSVSVEFAVAATDCIAK | |

| Collagen alpha-1(II) chain OS=Bos taurus GN=COL2A1 PE=1 SV=4 | 61 |
| --- | --- |

| 664.8278 | 1327.641 | 1327.641 | 47.56 | GFPGLPGPSGEPGK | 2 Oxidation (P) |
| --- | --- | --- | --- | --- | --- |
| 672.8251 | 1343.636 | 1343.636 | 43.24 | GFPGLPGPSGEPGK | 3 Oxidation (P) |

| Plakophilin-1 OS=Bos taurus GN=PKP1 PE=2 SV=1 | 45 |
| --- | --- |

| 762.897 | 1523.779 | 1523.78 | 44.55 | SPNQNVQQAAAGALR |
| --- | --- | --- | --- | --- |

**WR16**

| Collagen alpha-1(II) chain OS=Bos taurus GN=COL2A1 PE=1 SV=4 | 127 |
| --- | --- |

| **pep_exp_mz** | **pep_exp_mr** | **pep_calc_mr** | **pep_score** | **pep_seq** | **pep_var_mod** |
| --- | --- | --- | --- | --- | --- |
| 664.8276 | 1327.641 | 1327.641 | 59.8 | GFPGLPGPSGEPGK | 2 Oxidation (P) |
| 664.8279 | 1327.641 | 1327.641 | 82.86 | GFPGLPGPSGEPGK | 2 Oxidation (P) |

| Cytochrome c OS=Bos taurus GN=CYCS PE=1 SV=2 | 43 |
| --- | --- |

| 584.8148 | 1167.615 | 1167.615 | 43.12 | TGPNLHGLFGR |
| --- | --- | --- | --- | --- |

| Elongation factor 1-alpha 1 OS=Bos taurus GN=EEF1A1 PE=2 SV=1 | 43 |
| --- | --- |

| 513.3093 | 1024.604 | 1024.603 | 42.94 | IGGIGTVPVGR |
| --- | --- | --- | --- | --- |
| 513.3093 | 1024.604 | 1024.603 | 42.94 | IGGIGTVPVGR |

| Serotransferrin OS=Bos taurus GN=TF PE=2 SV=1 | 40 |
| --- | --- |

| 733.8261 | 1465.638 | 1465.636 | 40.23 | TYDSYLGDDYVR |
| --- | --- | --- | --- | --- |

**WR17**

| Serotransferrin OS=Bos taurus GN=TF PE=2 SV=1 | 57 |
| --- | --- |

| **pep_exp_mz** | **pep_exp_mr** | **pep_calc_mr** | **pep_score** | **pep_seq** | **pep_var_mod** |
| --- | --- | --- | --- | --- | --- |
| 586.6244 | 1756.851 | 1756.853 | 48.71 | DKPDNFQLFQSPHGK | |

**WR18**

| Biglycan OS=Bos taurus GN=BGN PE=1 SV=3 | 80 |
| --- | --- |

| **pep_exp_mz** | **pep_exp_mr** | **pep_calc_mr** | **pep_score** | **pep_seq** | **pep_var_mod** |
| --- | --- | --- | --- | --- | --- |
| 748.3667 | 1494.719 | 1494.718 | 49.88 | VGVNDFCPVGFGVK | Deamidated (NQ) |
| 748.3673 | 1494.72 | 1494.718 | 59.22 | VGVNDFCPVGFGVK | Deamidated (NQ) |

| Pigment epithelium-derived factor OS=Bos taurus GN=SERPINF1 PE=1 SV=1 | 70 |
| --- | --- |

| 625.8366 | 1249.659 | 1249.655 | 69.95 | DTDTGALLFIGK |
| --- | --- | --- | --- | --- |

| Lumican OS=Bos taurus GN=LUM PE=1 SV=1 | 57 |
| --- | --- |

| 499.2772 | 996.5398 | 996.5392 | 44.16 | FSALQYLR | |
| --- | --- | --- | --- | --- | --- |
| 499.7689 | 997.5232 | 997.5233 | 50.8 | FSALQYLR | Deamidated (NQ) |

| Tetranectin OS=Bos taurus GN=CLEC3B PE=2 SV=1 | 49 |
| --- | --- |

| 542.7783 | 1083.542 | 1083.542 | 40.79 | CFLAFVQAK | Deamidated (NQ) |
| --- | --- | --- | --- | --- | --- |

**LQ1**

| Secreted phosphoprotein 24 OS=Bos taurus GN=SPP2 PE=1 SV=2 | 132 |
| --- | --- |

| **pep_exp_mz** | **pep_exp_mr** | **pep_calc_mr** | **pep_score** | **pep_seq** | **pep_var_mod** |
| --- | --- | --- | --- | --- | --- |
| 705.873 | 1409.731 | 1409.73 | 61.49 | VNSQSLSPYLFR | |
| 706.3637 | 1410.713 | 1410.714 | 52.64 | VNSQSLSPYLFR | Deamidated (NQ) |
| 471.2454 | 1410.714 | 1410.714 | 45.49 | VNSQSLSPYLFR | Deamidated (NQ) |
| 706.856 | 1411.697 | 1411.698 | 54.11 | VNSQSLSPYLFR | 2 Deamidated (NQ) |

| Osteomodulin OS=Bos taurus GN=OMD PE=1 SV=1 | 90 |
| --- | --- |

| 606.3248 | 1210.635 | 1210.635 | 62.03 | IFLGYNEISR | |
| --- | --- | --- | --- | --- | --- |
| 606.8157 | 1211.617 | 1211.619 | 51.02 | IFLGYNEISR | Deamidated (NQ) |
| 606.8163 | 1211.618 | 1211.619 | 40.7 | IFLGYNEISR | Deamidated (NQ) |

| Nucleobindin-1 OS=Bos taurus GN=NUCB1 PE=2 SV=1 | 78 |
| --- | --- |

| 753.9113 | 1505.808 | 1505.809 | 71.12 | LVTLEEFLASTQR |
| --- | --- | --- | --- | --- |

| Vitamin D-binding protein OS=Bos taurus GN=GC PE=2 SV=1 | 74 |
| --- | --- |

| 691.8692 | 1381.724 | 1381.724 | 73.87 | VLDQYIFELSR |
| --- | --- | --- | --- | --- |

| Matrix Gla protein OS=Bos taurus GN=MGP PE=1 SV=1 | 61 |
| --- | --- |

| 787.3345 | 1572.654 | 1572.656 | 61.47 | YAMVYGYNAAYDR | Deamidated (NQ); Oxidation (M) |
| --- | --- | --- | --- | --- | --- |

| Gelsolin OS=Bos taurus GN=GSN PE=2 SV=1 | 60 |
| --- | --- |

| 436.7634 | 871.5122 | 871.5127 | 59.71 | TGALELLR |  |
| --- | --- | --- | --- | --- | --- |

| Carboxypeptidase E OS=Bos taurus GN=CPE PE=1 SV=2 | 59 |
| --- | --- |

| 844.9426 | 1687.871 | 1687.872 | 58.8 | EALVSVWLQCAAVSR |
| --- | --- | --- | --- | --- |

| Bone morphogenetic protein 3 OS=Bos taurus GN=BMP3 PE=1 SV=2 | 59 |
| --- | --- |

| 931.9697 | 1861.925 | 1861.925 | 58.53 | VDFADIGWSEWIISPK |
| --- | --- | --- | --- | --- |

| Bone sialoprotein 2 OS=Bos taurus GN=IBSP PE=1 SV=1 | 59 |
| --- | --- |

| 427.2204 | 1278.639 | 1278.64 | 44.96 | YRPQYYVYK |
| --- | --- | --- | --- | --- |

| Versican core protein OS=Bos taurus GN=VCAN PE=1 SV=2 | 58 |
| --- | --- |

| 657.8631 | 1313.712 | 1313.709 | 57.68 | LATVGELQAAWR |
| --- | --- | --- | --- | --- |

| Adiponectin OS=Bos taurus GN=ADIPOQ PE=1 SV=1 | 55 |
| --- | --- |

| 483.2556 | 964.4966 | 964.4978 | 54.54 | SAFSVGLER |
| --- | --- | --- | --- | --- |

| Asporin OS=Bos taurus GN=ASPN PE=2 SV=1 | 52 |
| --- | --- |

| 648.8222 | 1295.63 | 1295.63 | 43.56 | YWEVQPATFR |
| --- | --- | --- | --- | --- |

| Apolipoprotein A-I OS=Bos taurus GN=APOA1 PE=1 SV=3 | 52 |
| --- | --- |

| 788.9131 | 1575.812 | 1575.814 | 44.77 | LLDNWDTLASTLSK |
| --- | --- | --- | --- | --- |

| Ezrin OS=Bos taurus GN=EZR PE=1 SV=2 | 49 |
| --- | --- |

| 591.8018 | 1181.589 | 1181.587 | 48.78 | APDFVFYAPR |
| --- | --- | --- | --- | --- |

| Moesin OS=Bos taurus GN=MSN PE=2 SV=3 | 49 |
| --- | --- |

| 591.8018 | 1181.589 | 1181.587 | 48.78 | APDFVFYAPR |
| --- | --- | --- | --- | --- |

| Radixin OS=Bos taurus GN=RDX PE=2 SV=1 | 49 |
| --- | --- |

| 591.8018 | 1181.589 | 1181.587 | 48.78 | APDFVFYAPR |
| --- | --- | --- | --- | --- |

| CD44 antigen OS=Bos taurus GN=CD44 PE=2 SV=1 | 43 |
| --- | --- |

| 462.9221 | 1385.745 | 1385.746 | 43.48 | YGFIEGHVVIPR |
| --- | --- | --- | --- | --- |

| Coagulation factor VII OS=Bos taurus GN=F7 PE=1 SV=2 | 42 |
| --- | --- |

| 919.4594 | 2755.356 | 2755.357 | 41.99 | LNGALLCGGTLVGPAWVVSAAHCFER | Deamidated (NQ) |
| --- | --- | --- | --- | --- | --- |

| Lysozyme C, milk isozyme OS=Bos taurus PE=2 SV=1 | 41 |
| --- | --- |

| 673.3554 | 1344.696 | 1344.697 | 40.71 | GVSLANWVCLAR |
| --- | --- | --- | --- | --- |

| Unconventional myosin-Id OS=Bos taurus GN=MYO1D PE=2 SV=1 | 41 |
| --- | --- |

| 540.785 | 1079.555 | 1079.559 | 40.52 | LFCWIVSR |
| --- | --- | --- | --- | --- |

| Fibronectin OS=Bos taurus GN=FN1 PE=1 SV=4 | 40 |
| --- | --- |

| 948.5316 | 1895.049 | 1895.052 | 40.12 | VTWAPPSSIELTNLLVR |
| --- | --- | --- | --- | --- |

**NS2**

| Matrix Gla protein OS=Bos taurus GN=MGP PE=1 SV=1 | 126 |
| --- | --- |

| **pep_exp_mz** | **pep_exp_mr** | **pep_calc_mr** | **pep_score** | **pep_seq** | **pep_var_mod** |
| --- | --- | --- | --- | --- | --- |
| 779.3347 | 1556.655 | 1556.661 | 42.35 | YAMVYGYNAAYDR | Deamidated (NQ) |
| 779.335 | 1556.655 | 1556.661 | 56.39 | YAMVYGYNAAYDR | Deamidated (NQ) |
| 787.3339 | 1572.653 | 1572.656 | 51.61 | YAMVYGYNAAYDR | Deamidated (NQ); Oxidation (M) |
| 787.3347 | 1572.655 | 1572.656 | 59.24 | YAMVYGYNAAYDR | Deamidated (NQ); Oxidation (M) |

| Transthyretin OS=Bos taurus GN=TTR PE=1 SV=1 | 91 |
| --- | --- |

| 1235.644 | 2469.273 | 2469.279 | 90.76 | HYTIAALLSPYSYSTTALVSSPK |
| --- | --- | --- | --- | --- |

| Bone sialoprotein 2 OS=Bos taurus GN=IBSP PE=1 SV=1 | 76 |
| --- | --- |

| 548.2844 | 1094.554 | 1094.555 | 40.24 | HGYFYPALK |
| --- | --- | --- | --- | --- |
| 548.2845 | 1094.554 | 1094.555 | 43.83 | HGYFYPALK |
| 417.8923 | 1250.655 | 1250.656 | 40.95 | HGYFYPALKR |

| Procollagen-lysine,2-oxoglutarate 5-dioxygenase 1 OS=Bos taurus GN=PLOD1 PE=2 SV=2 | 71 |
| --- | --- |

| 814.4265 | 1626.838 | 1626.841 | 70.93 | FLGSGGFIGYAPNLSK |
| --- | --- | --- | --- | --- |

| Versican core protein OS=Bos taurus GN=VCAN PE=1 SV=2 | 70 |
| --- | --- |

| 657.8613 | 1313.708 | 1313.709 | 69.98 | LATVGELQAAWR |
| --- | --- | --- | --- | --- |

| Tubulin beta-5 chain OS=Bos taurus GN=TUBB5 PE=2 SV=1 | 69 |
| --- | --- |

| 615.3027 | 1228.591 | 1228.591 | 68.83 | ISEQFTAMFR |
| --- | --- | --- | --- | --- |

| Tubulin beta-2B chain OS=Bos taurus GN=TUBB2B PE=1 SV=2 | 69 |
| --- | --- |

| 615.3027 | 1228.591 | 1228.591 | 68.83 | ISEQFTAMFR |
| --- | --- | --- | --- | --- |

| Tubulin beta-3 chain OS=Bos taurus GN=TUBB3 PE=2 SV=1 | 69 |
| --- | --- |

| 615.3027 | 1228.591 | 1228.591 | 68.83 | ISEQFTAMFR |
| --- | --- | --- | --- | --- |

| Tubulin beta-4A chain OS=Bos taurus GN=TUBB4A PE=2 SV=1 | 69 |
| --- | --- |

| 615.3027 | 1228.591 | 1228.591 | 68.83 | ISEQFTAMFR |
| --- | --- | --- | --- | --- |

| Tubulin beta-4B chain OS=Bos taurus GN=TUBB4B PE=2 SV=1 | 69 |
| --- | --- |

| 615.3027 | 1228.591 | 1228.591 | 68.83 | ISEQFTAMFR |
| --- | --- | --- | --- | --- |

| 72 kDa type IV collagenase OS=Bos taurus GN=MMP2 PE=2 SV=1 | 69 |
| --- | --- |

| 709.8754 | 1417.736 | 1417.735 | 61.92 | AFQVWSDVTPLR |
| --- | --- | --- | --- | --- |

| Complement C1q subcomponent subunit B OS=Bos taurus GN=C1QB PE=1 SV=1 | 67 |
| --- | --- |

| 822.9133 | 1643.812 | 1643.81 | 61.12 | VPGLYFFTYHASSR |
| --- | --- | --- | --- | --- |

| Osteomodulin OS=Bos taurus GN=OMD PE=1 SV=1 | 66 |
| --- | --- |

| 606.3242 | 1210.634 | 1210.635 | 47.08 | IFLGYNEISR | |
| --- | --- | --- | --- | --- | --- |
| 606.8165 | 1211.618 | 1211.619 | 44.39 | IFLGYNEISR | Deamidated (NQ) |
| 606.8171 | 1211.62 | 1211.619 | 45.28 | IFLGYNEISR | Deamidated (NQ) |

| Carboxypeptidase E OS=Bos taurus GN=CPE PE=1 SV=2 | 65 |
| --- | --- |

| 844.9442 | 1687.874 | 1687.872 | 70.38 | EALVSVWLQCAAVSR |
| --- | --- | --- | --- | --- |

| Fibrinogen alpha chain OS=Bos taurus GN=FGA PE=1 SV=5 | 64 |
| --- | --- |

| 797.4701 | 1592.926 | 1592.925 | 58.52 | QLEQVIAINLLPSR |
| --- | --- | --- | --- | --- |

| Actin, cytoplasmic 1 OS=Bos taurus GN=ACTB PE=1 SV=1 | 63 |
| --- | --- |

| 599.8558 | 1197.697 | 1197.698 | 54.51 | AVFPSIVGRPR |
| --- | --- | --- | --- | --- |

| Actin, alpha cardiac muscle 1 OS=Bos taurus GN=ACTC1 PE=2 SV=1 | 63 |
| --- | --- |

| 599.8558 | 1197.697 | 1197.698 | 54.51 | AVFPSIVGRPR |
| --- | --- | --- | --- | --- |

| Actin, cytoplasmic 2 OS=Bos taurus GN=ACTG1 PE=1 SV=1 | 63 |
| --- | --- |

| 599.8558 | 1197.697 | 1197.698 | 54.51 | AVFPSIVGRPR |
| --- | --- | --- | --- | --- |

| Actin, alpha skeletal muscle OS=Bos taurus GN=ACTA1 PE=1 SV=1 | 63 |
| --- | --- |

| 599.8558 | 1197.697 | 1197.698 | 54.51 | AVFPSIVGRPR |
| --- | --- | --- | --- | --- |

| Actin, aortic smooth muscle OS=Bos taurus GN=ACTA2 PE=1 SV=1 | 55 |
| --- | --- |

| 599.8558 | 1197.697 | 1197.698 | 54.51 | AVFPSIVGRPR |
| --- | --- | --- | --- | --- |

| Actin, gamma-enteric smooth muscle OS=Bos taurus GN=ACTG2 PE=2 SV=1 | 55 |
| --- | --- |

| 599.8558 | 1197.697 | 1197.698 | 54.51 | AVFPSIVGRPR |
| --- | --- | --- | --- | --- |

| Complement C4 (Fragments) OS=Bos taurus GN=C4 PE=1 SV=2 | 62 |
| --- | --- |

| 684.3633 | 1366.712 | 1366.713 | 61.72 | DSSTWLTAFVLK |
| --- | --- | --- | --- | --- |

| Lysyl oxidase homolog 4 OS=Bos taurus GN=LOXL4 PE=2 SV=1 | 61 |
| --- | --- |

| 736.0707 | 2205.19 | 2205.191 | 61.16 | EALFGAQLGQALGPIHLSEVR |
| --- | --- | --- | --- | --- |

| Tubulin alpha-8 chain OS=Bos taurus GN=TUBA8 PE=2 SV=1 | 61 |
| --- | --- |

| 496.6312 | 1486.872 | 1486.872 | 54.88 | LISQIVSSITASLR |
| --- | --- | --- | --- | --- |

| Tubulin alpha-1B chain OS=Bos taurus PE=1 SV=2 | 55 |
| --- | --- |

| 496.6312 | 1486.872 | 1486.872 | 54.88 | LISQIVSSITASLR |
| --- | --- | --- | --- | --- |

| Tubulin alpha-4A chain OS=Bos taurus GN=TUBA4A PE=1 SV=2 | 55 |
| --- | --- |

| 496.6312 | 1486.872 | 1486.872 | 54.88 | LISQIVSSITASLR |
| --- | --- | --- | --- | --- |

| Apolipoprotein A-II OS=Bos taurus GN=APOA2 PE=1 SV=2 | 60 |
| --- | --- |

| 933.505 | 1864.995 | 1864.994 | 52.4 | KAGTDLLNFLSSFIDPK |
| --- | --- | --- | --- | --- |

| Fibromodulin OS=Bos taurus GN=FMOD PE=1 SV=2 | 58 |
| --- | --- |

| 519.9595 | 1556.857 | 1556.856 | 42.3 | SLILLDLSYNHLR | Deamidated (NQ) |
| --- | --- | --- | --- | --- | --- |

| Nucleotide exchange factor SIL1 OS=Bos taurus GN=SIL1 PE=2 SV=1 | 57 |
| --- | --- |

| 582.3337 | 1162.653 | 1162.653 | 57.39 | ALFALCSLLR |
| --- | --- | --- | --- | --- |

| Moesin OS=Bos taurus GN=MSN PE=2 SV=3 | 57 |
| --- | --- |

| 591.8006 | 1181.587 | 1181.587 | 56.45 | APDFVFYAPR |
| --- | --- | --- | --- | --- |

| Ezrin OS=Bos taurus GN=EZR PE=1 SV=2 | 57 |
| --- | --- |

| 591.8006 | 1181.587 | 1181.587 | 56.45 | APDFVFYAPR |
| --- | --- | --- | --- | --- |

| Gelsolin OS=Bos taurus GN=GSN PE=2 SV=1 | 53 |
| --- | --- |

| 861.9259 | 1721.837 | 1721.83 | 52.56 | EVQGFESATFLGYFK |
| --- | --- | --- | --- | --- |

| Lysosomal alpha-mannosidase OS=Bos taurus GN=MAN2B1 PE=1 SV=3 | 51 |
| --- | --- |

| 653.3693 | 1304.724 | 1304.724 | 51.26 | FQVIVYNPLGR |
| --- | --- | --- | --- | --- |

| Elongation factor 1-gamma OS=Bos taurus GN=EEF1G PE=2 SV=1 | 50 |
| --- | --- |

| 805.4014 | 1608.788 | 1608.787 | 45.48 | WFLTCINQPQFR |
| --- | --- | --- | --- | --- |

| 60S ribosomal protein L6 OS=Bos taurus GN=RPL6 PE=2 SV=3 | 48 |
| --- | --- |

| 763.4689 | 1524.923 | 1524.924 | 47.5 | ASITPGTILIILTGR |
| --- | --- | --- | --- | --- |

| Histone H2A.V OS=Bos taurus GN=H2AFV PE=2 SV=3 | 47 |
| --- | --- |

| 472.7689 | 943.5232 | 943.524 | 47.47 | AGLQFPVGR |
| --- | --- | --- | --- | --- |
| 472.7689 | 943.5232 | 943.524 | 47.47 | AGLQFPVGR |

| Adiponectin OS=Bos taurus GN=ADIPOQ PE=1 SV=1 | 47 |
| --- | --- |

| 483.2559 | 964.4972 | 964.4978 | 47.06 | SAFSVGLER |
| --- | --- | --- | --- | --- |

| Monocyte differentiation antigen CD14 OS=Bos taurus GN=CD14 PE=2 SV=2 | 47 |
| --- | --- |

| 810.005 | 1617.995 | 1617.993 | 47.12 | LGAAQVPAQLLVAVLR |
| --- | --- | --- | --- | --- |

| Complement C1q subcomponent subunit A OS=Bos taurus GN=C1QA PE=2 SV=1 | 46 |
| --- | --- |

| 1079.529 | 2157.043 | 2157.042 | 45.69 | IYHGSEADSIFSGFLIFPSA |
| --- | --- | --- | --- | --- |

| Vimentin OS=Bos taurus GN=VIM PE=1 SV=3 | 44 |
| --- | --- |

| 585.3604 | 1168.706 | 1168.707 | 44.06 | ILLAELEQLK |
| --- | --- | --- | --- | --- |

| Hemoglobin fetal subunit beta OS=Bos taurus PE=1 SV=1 | 43 |
| --- | --- |

| 633.4189 | 1264.823 | 1264.823 | 42.83 | LLGNVLVVVLAR |
| --- | --- | --- | --- | --- |

| Hemoglobin subunit beta OS=Bos taurus GN=HBB PE=1 SV=1 | 43 |
| --- | --- |

| 633.4189 | 1264.823 | 1264.823 | 42.83 | LLGNVLVVVLAR |
| --- | --- | --- | --- | --- |

| Histone H2A type 1 OS=Bos taurus PE=1 SV=2 | 42 |
| --- | --- |

| 472.7689 | 943.5232 | 943.524 | 47.47 | AGLQFPVGR |
| --- | --- | --- | --- | --- |

| Histone H2A type 2-C OS=Bos taurus GN=HIST2H2AC PE=2 SV=1 | 42 |
| --- | --- |

| 472.7689 | 943.5232 | 943.524 | 47.47 | AGLQFPVGR |
| --- | --- | --- | --- | --- |

| Histone H2A.J OS=Bos taurus GN=H2AFJ PE=2 SV=1 | 42 |
| --- | --- |

| 472.7689 | 943.5232 | 943.524 | 47.47 | AGLQFPVGR |
| --- | --- | --- | --- | --- |

**HSB3 100 mg sample**

| Hemoglobin fetal subunit beta OS=Bos taurus PE=1 SV=1 | 78 |
| --- | --- |

| **pep_exp_mz** | **pep_exp_mr** | **pep_calc_mr** | **pep_score** | **pep_seq** | **pep_var_mod** |
| --- | --- | --- | --- | --- | --- |
| 633.4196 | 1264.825 | 1264.823 | 77.76 | LLGNVLVVVLAR | |

| Secreted phosphoprotein 24 OS=Bos taurus GN=SPP2 PE=1 SV=2 | 83 |
| --- | --- |

| 934.4421 | 1866.87 | 1866.867 | 82.79 | VNALDEDSLTMDLEFR |
| --- | --- | --- | --- | --- |

| Tetranectin OS=Bos taurus GN=CLEC3B PE=2 SV=1 | 73 |
| --- | --- |

| 764.933 | 1527.851 | 1527.851 | 73.34 | TQLDSLAQEVALLK |
| --- | --- | --- | --- | --- |

| SPARC OS=Bos taurus GN=SPARC PE=1 SV=2 | 48 |
| --- | --- |

| 724.3197 | 1446.625 | 1446.624 | 43.01 | TFDSSCHFFATK |
| --- | --- | --- | --- | --- |

| Matrix Gla protein OS=Bos taurus GN=MGP PE=1 SV=1 | 44 |
| --- | --- |

| 778.8459 | 1555.677 | 1555.677 | 43.68 | YAMVYGYNAAYDR |
| --- | --- | --- | --- | --- |
