# Supplementary material for: Proteome degradation in fossils: investigating the longevity of protein survival in ancient bone
Source: Rapid Commun Mass Spectrom. 2014 Feb 12;28(6):605–15. doi: 10.1002/rcm.6821 (PMC4282581; doi:10.1002/rcm.6821)
Supplement: Supplementary file 3 [file rcm0028-0605-SD3.docx]

**Protein Code List**

| **Protein name** | **Gene name** |
| --- | --- |
| Collagen alpha-2 (I) | COL1A2 |
| Collagen alpha-1 (I) | COL1A1 |
| Collagen alpha-1 (II) | COL2A1 |
| Collagen alpha-1 (III) | COL3A1 |
| Collagen alpha-1 (XI) | COL11A1 |
| Alpha-2-HS-glycoprotein | AHSG |
| Antithrombin-III | SERPIN C1 |
| Apolipoprotein A-I | APOA1 |
| Apolipoprotein A-II | APOA2 |
| Asporin | ASPN |
| Biglycan | BGN |
| Bone morphogenic protein 3 | BMP3 |
| Chondroadherin | CHAD |
| Coagulation factor IX | F9 |
| Complement C3 | C3 |
| Complement C4 | C4A |
| Complement C9 | C9 |
| Decorin | DCN |
| Lumican | LUM |
| Nucleobindin-1 | NUCB1 |
| Olfactomedin-like protein 3 | OLFML3 |
| Osteomodulin | OMD |
| Pigment epithelium-derived factor (PEDF) | SERPINF1 |
| Prothrombin | F2 |
| Serum albumin | ALB |
| SPARC | SPARC |
| Tetranectin | CLEC3B |
| Thrombospondin-1 | THBS1 |
| Vitamin D binding protein | GC |
| Vitamin K dependent protein C | PROC |
| Vitamin K dependent protein S | PROS1 |
| Vitrin | VIT |
