# Supplementary material for: Proteome degradation in fossils: investigating the longevity of protein survival in ancient bone
Source: Rapid Commun Mass Spectrom. 2014 Feb 12;28(6):605–15. doi: 10.1002/rcm.6821 (PMC4282581; doi:10.1002/rcm.6821)
Supplement: Supplementary file 4 [file rcm0028-0605-SD4.docx]

**Supplementary Text S1**

Alpha 2 HS glycoprotein and albumin sequences were obtained from UniProt or Ensembl and aligned using ClustalW.

**Alpha 2 HS glycoprotein**

>Gallus (E1BZE1 - E1BZE1_CHICK

MKALVAFILLVQLPIHRAAPAAPPPPLGCDDPEIEAAAEFAVIYINGHSHHGYRFALNRI

EQVRVLFQGPNNEILFLELDLLETTCHILNPTPLVNCSVRTFAEHAVEGDCDVKLQKVNG

QFSVLASKCHSHADSAEDIREVCPNCPLLANLNDTDVLAAVSSALNDYNSKNPDVYLMLL

EIGRAVKQYHPVRMVSVEFAVAATNCTSQQAKDNLAACQLLPEDQSNFGFCTAKMVTEPS

QDLIAECQLYGHQPGVTYPHPGQDTSAGLVPSAGQGFTNHNLKISHNNPVASESSSSEFP

SLLSAKSVAKRAAAEVAQHDKVPHPVGFVPPPPLCPGKIRHFKI

>Bos (P12763 – FETUA_BOVIN)

MKSFVLLFCLAQLWGCHSIPLDPVAGYKEPACDDPDTEQAALAAVDYINKHLPRGYKHTL

NQIDSVKVWPRRPTGEVYDIEIDTLETTCHVLDPTPLANCSVRQQTQHAVEGDCDIHVLK

QDGQFSVLFTKCDSSPDSAEDVRKLCPDCPLLAPLNDSRVVHAVEVALATFNAESNGSYL

QLVEISRAQFVPLPVSVSVEFAVAATDCIAKEVVDPTKCNLLAEKQYGFCKGSVIQKALG

GEDVRVTCTLFQTQPVIPQPQPDGAEAEAPSAVPDAAGPTPSAAGPPVASVVVGPSVVAV

PLPLHRAHYDLRHTFSGVASVESSSGEAFHVGKTPIVGQPSIPGGPVRLCPGRIRYFKI

>Ovis (P29701 – FETUA_SHEEP)

MKSFLLLFCLAQLCSCRSIPLDPIAGYKEPACDDPDTEQAALAAVDYINKHLPRGYKHTL

NQIDSVKVWPRRPTGEVYDIEIDTLETTCHVLDPTPLVNCSVRQQTEHAVEGDCDIHVLK

QDGQFSVLFTKCDSSPDSAEDVRKLCPDCPLLAPLNNSQVVHAAEVALATFNAQNNGSYF

QLVEISRAQFVPLPGSVSVEFAVAATDCIAKEVVDPTKCNLLAEKQYGFCKGSVIQKALG

GEDVTVTCTLFQTQPVIPQPQPEGAEAGAPSAVPDAAVPDAAVPAPSAAGLPVGSVVAGP

SVVAVPLPLHRAHYDLRHTFSGVASVESASGEAFHVGKTPIVGQPSVPGGPVHLCPGRIR

YFKI

>Sus (P29700 – FETUA_PIG)

LILFFCLAQLWGCRAVPHGPILGYREPACDDVETEQAALAAVDYINKHLPRGYKHTLNQV

DSVKVWPRRPAGEVFDIEIDTLETTCHVLDPTPLANCSVRQLTEHAVEGDCDFHVLKQDG

QFSVLFAKCDSSPDSAEDVHKVCPNCPLLAPLNDSRVVHAAESALAAFNAQSNGSYLQLV

EISRAQLVPLSASVSVEFAVAVTDCVAKEAYSPTKCNLLVEKQYGFCKGTVTAKVNEEDV

AVTCTVFQTQPVVLQPQPAGADAGATPVVDAAATASPLADVPAASLVVGPMVVAVPPGIP

PVHRSHYDLRHSFSGVASVESASGEAFHVGKTPKGAQPSIPAADGSVPVVRPCPGRIRHF

KI

>Equus (F7C450 - F7C450_HORSE)

MKSFVLLFCLAQLCHCLSLPNGLSPAYRQLNCDDPETEQAALLAVDYINSHIHQGYKHVL

NQIDKVQVWAQPTGESFKLEIDTLETTCHALDPTPLANCSVRQLTQHAVEGDCDVRLLKQ

NGQFSVSFVKCKSSPDSAEDVRKVCLDCPLLAPLNDTRVVHAVEAALAAFNAQNNGSYFQ

LVEISRAQLVPLPVSVHVEFAVAATDCVAKEVIDPAKCNLLAEKQYGFCKATLTEKVGGE

DVAVTCTVFQTQPVVLLPQPDGPDVGVPGPVADAVTPAPSPADLPVASLVVGPVVLPPPV

HRAHYDLRHAFAGVGSGESASGEAFHVEKPPKVAHPNTAAAAGPVVRPCPGRIRYFKI

>Felis (M3WG97 - M3WG97_FELCA)

MKSLALLLCLAQLWGCHAAPHIPGLVYRELDCDDPETEQAALVAVDYINNHVLQGYKHTL

NQIDKVKVWPRRPMGEVFELEIDTLETTCYIRDPTPVANCTVRQLMEHAVEGDCDFQVLK

QDGQFTVLFAKCDSSPGLDSAEDVRKVCPQCPLLAPLNDTKVVHAVEVALTAFNTQSNGS

YFQLVEVSRAQLVPLPPSTYVEFAVAATDCVAAEVTDPAKCNLLAEKQYGFCKATLTEKV

GGEDVAVTCTMFQTQPVLPQPQPDGTEASPVADSAVTAPPPADPPAAALVVGPVVVAAPQ

APLPGHRAHYDLRHAFMGVASVESASGEAVHVGKVPNVAQPSVPVAAGPVVRPCPGRIRH

FKI

>Canis (E2QUV3 - E2QUV3_CANFA)

MKSLALFLCLAQLWGCHSVPVGPALVYRELDCDDPETEQAALVAVDYINNHVLRGYKHTL

NQIDKVKVWPRRPMGEVFELEIDTLETTCHVLDPTPLANCSVRPQMQHAVEGDCDFRLLK

QDGQFTVLFAKCDSSPDSAEDVRKVCPQCPLLAPLNDTRVVHAVDAALTAFNAQSNGSYF

RLVEVSRAQLTPLPPSTYVEFAVAATDCVAAEVTDPAKCNLLAEKVHLQYEILKMSVIRK

CYITITLLLVIMGHLLPCPSESHNINCPGYMESATNERIFQPCLPLPRASGFSCPGGRTR

VVAFPEAPLPEHRAHYDLRHAFMGVASVESASGEAFHVGKVPKVVQPDVAVAAGPVVHPC

PGRIRHFKI

>Homo (P02765 – FETUA_HUMAN)

MKSLVLLLCLAQLWGCHSAPHGPGLIYRQPNCDDPETEEAALVAIDYINQNLPWGYKHTL

NQIDEVKVWPQQPSGELFEIEIDTLETTCHVLDPTPVARCSVRQLKEHAVEGDCDFQLLK

LDGKFSVVYAKCDSSPDSAEDVRKVCQDCPLLAPLNDTRVVHAAKAALAAFNAQNNGSNF

QLEEISRAQLVPLPPSTYVEFTVSGTDCVAKEATEAAKCNLLAEKQYGFCKATLSEKLGG

AEVAVTCTVFQTQPVTSQPQPEGANEAVPTPVVDPDAPPSPPLGAPGLPPAGSPPDSHVL

LAAPPGHQLHRAHYDLRHTFMGVVSLGSPSGEVSHPRKTRTVVQPSVGAAAGPVVPPCPG

RIRHFKV

>Pan (Q9N2D0 – FETUA_PANTR)

MKSLVLLLCLAQLWGCHSAPRGLGLIYRQPNCDDPETEEAALVAIDYINQNHPWGYKHTL

NQIDEVKVWPRQPSGELFEIEIDTLETTCHVLDPTPVARCSVRQLKEHAVEGDCDFQLLK

LDGKFSVVYAKCDSSPDSAEDVRKVCQDCPLLAPLNDTRVVHAAKAALAAFNAQNNGSNF

QLEEISRAQLVPLPPSTYVEFTVSGTDCVAKEATEAAKCNLLAEKQYGFCKATLSEKLGG

AEVAVTCTVFQTQPVTSQPQPEGANETVPTPVVDPDAPPSPPLGAPGLPPAGSPPDSHVL

LAAPPGHQLHWAHYDLRHTFMGVVSLGSPSGEASHPRKTRTVVQPSVGAAAGPVVPPCPG

RIRHFKV

>Meriones (P97515 – FETUA_MERUN)

MKTLVLLLCFTLLWGCQSAPQGTGLGFREVACDDPEVEQVALTAVDYLNQHLLQGFKHIL

NQIDKVKVWSRRPFGEVYELELDTLETTCHALDPTPLANCSVRQLAQHAVEGDCDFHILK

QDGQFSVMHTKCHSNPDSAEDVRKVCPHCALLTPFNSSNVVYAVNAALGAFNEKNNKTYF

KLVELARAQTVPFPPSTHVEFVIAATDCAAPKVADPAKCNLLAEKQYSFCKASLFQNLGG

EEVTVTCTAFPTQANGVTPASPAPAVEKGIPVALPDAPPASLVVGPMVVPAEHLPHKTHH

DLRHAFSPVASVESASGEAFQSPTQAGNAGAAGPAVPLCPGRVRHFKI

>Pteropus (L5JPU2 - L5JPU2_PTEAL)

MRSLVLVLCLAQLWGCHSAPHGPGLGYRELNCDDPETEQAALLAVDYINSHLLRGYKHTL

NQIDKVKVWPRRPMGEVFEIEIDTLETTCHALDPTPVANCSVRQLQEHAVEGDCDFRVLK

QDGQFSVLFAKCDSSPDSAEDVLKVCPDCPLLAPLNDTRVVHAVEAALAAFNARNNGSYY

QLVEVSRAQLVPFPVSTYVEFVVAATDCVSKEVTDPAKCNLLAEKQYGFCKARLTGKFGG

EDVAVTCVVFQTQPVLPQPQPEGTNSPTPAETPAKPLSPPANLPAATVVVGPLVAAAPPP

SLPVHRAHYDLRHAFGGVASVESASGEAFLVEKAPTVVQPGVAVAAGPVVRPCPGRIRHF

KI

>Rattus (P24090 – FETUA_RAT)

MKSLVLLLCFAQLWSCQSAPQGAGLGFRELACDDPETEHVALIAVDYLNKHLLQGFRQIL

NQIDKVKVWSRRPFGEVYELEIDTLETTCHALDPTPLANCSVRQQAEHAVEGDCDFHILK

QDGQFRVLHAQCHSTPDSAEDVRKFCPRCPILIRFNDTNVVHTVKTALAAFNAQNNGTYF

KLVEISRAQNVPFPVSTLVEFVIAATDCTGQEVTDPAKCNLLAEKQYGFCKATLIHRLGG

EEVSVACKLFQTQPQPANANPAGPAPTVGQAAPVAPPAGPPESVVVGPVAVPLGLPDHRT

HHDLRHAFSPVASVESASGEVLHSPKVGQPGDAGAAGPVAPLCPGRVRYFKI

>Otolemur (H0XG01 - H0XG01_OTOGA)

MKSLILLVCLAQLWGCHSAPHGPQLTYSQPNCDDPETEQAALAAVDYLNSHLQQGYKHVL

NQIDKVKVWPRRPFGVIFELGVDTLESTCHVFDPTALANCTVRQVAEHAVEGDCDFRVLK

QDGQFSVVFAKCDSSPGADSAEDVRKVCPQCPLLAQLNDTRVVHAVEAALAAFNVQNNGS

YFQLVETSRAQLVPLPPSTRVEFAIAATDCVAKEVADPAKCNLLAEKQYGFCKATVTEKL

GGEEVAVTCTVFQPQPAAPQPQPADPTMSVLVAVDPPGPAFPPAGPPAAAMVVGPMVVAA

RPGPPLHRTHYDLRHAFSGVASAESASGEAFHPGKAPGVTQPGVAGAVGPMVLPPCPGRV

RHFKV

>Gorilla (E1U7Q5 - E1U7Q5_9PRIM)

MKSLVLLLCLAQLWGCHSAPHGPGLIYRQPNCDDPETEEAALVAIDYINQNLPWGYKHTL

NQIDEVKVWPRQPSGELFEIEIDTLETTCHVLDPTPVAKCSVRQLKEHAVEGDCDFQLLK

LDGKFSVVYAKCDSSPDSAEDVRKVCQDCPLLAPLNDTRVVHAAKAALAAFNAQNNGSNF

QLEEISRAQLVPLPPSTYVEFTVSGTDCVAKEATEAAKCNLLAEKQYGFCKATLSEKLGG

AEVAVTCTVFQTQPVTSQPQPEGANEAVPTPVVDPDAPPSPPLGAPGLPPAGLPPDSHVL

LAAPPGHQLHWAHYDLRHTFMGVVSLGSPSGEASHPRKTRTVVQPSVGAAAGPVVPPCPG

RIRHFKV

>Ailuropoda (G1L2K2 - G1L2K2_AILME)

MRSLALLLCLAQLCSCRSIPLGTALTYRQLNCDDPETEQAALAAVDYINSHMLQGYKHTL

NQIDKVKVWPRRPMGEVFELEIDTLETTCHVLDPTPLANCSVRQLVEHAVEGDCDFQVLK

QDGQFSVLFAKCDSSPDSAEDVRRLCPGCPLLAPLNDTKVVHAVDVALTAFNAQSNGSYF

RLLEVSRAQLVPLPPSTYVEFAVAATDCAAKDVTDPAKCNLLAEKQYGFCKATLTEKVGG

EDVAVTCTVFQTQPLLPQPQPEGPDAVAVDPGADPAVPVPSQADVPVASLVVGPVVPEAP

LPGHRTHYDLRRAFTGVVSEESASGEAVHVEKGPTVVQPGIAVVPDPALRPCPGRIRYFK

V

>Spermophilus (I3MDL1 - I3MDL1_SPETR)

MKSFILLLCLTQLWGCHSAPHGPLGYREPNCDDPETEQAALAAVDHINKFLLQGYKQILN

QIDKVKVSSRRPFGEMYELEIDTLETTCHVLDPTPLANCSVRQVAQHAVEGDCKVHVLKL

NGKFSVVFAKCDSTPDSAEDVRKVCPICPLLAQLNDTRVVHAVEAALAAFNGQKNGSYYQ

LLEISRAQIVPLPVSTYVEFAVAATDCVAKEVTDPAKCNLLAEKQYGFCKATVNEKVGGE

EVAVTCTLFQTQPVVIQPQPDANVANPDAVVEQATPASLPAGQPAASQVVGPMVVSPPRG

PPSHRVHYDLRYAFSGVASVESASGEAFHPGKTQPGVAGAADPMVRPCPGRIRHFKI

>Nomascus (G1R4B1 - G1R4B1_NOMLE)

MKSLVLLLCLAQLWGCHSAPHGPGLIYTQPNCDDPETEEAALAAIDYINQNLPWGYKHTL

NQIDEVKVWPRRPFGELFEIEIDTLETTCHVLDPTPVARCSVRQLKEHAVEGDCDFRLLK

LDGKFSVEYAKCDSSPDSAEDVRKVCRDCPLLAPLNDTRVVHAAEAALAAFNAQNNGSNF

QLEDISRAQLVPLPPSTYVEFTMSGTDCVAKEATEAAECNLLAEKQYGFCKATLSEKLGG

EEVAVTCTVFQTQPVTSQPQPEGADEAVPTPVVDPDARPSPPLGAPGPPPAGLPPHSHVL

AAAPQALPSHRAHYDLRHVFMGVVRLGSPLGEALHPQKTHPVVQPSVGAAAGPVVPPCPG

RIRHFKV

>Loxodonta (G3T019 - G3T019_LOXAF)

MKSLALLLCAQLWDCHLASPSPLLGYREPNCDDPETEQVAKAAVDYINAHVLHGYKHVLN

QIDEVKVWSPDPTREVFELELDTLETICHVLDPTPVANCTPRQLTEHAVEGDCDFQVLKQ

GGQFVVLFAKCDSSPDSAEDVRKVCPHCPLLAPFNDTKVVHAVEAALAAFNAQSNGSYYK

IVEVSRAQLVLLPPSAYVEFAVAATDCVAKEVTDPAKCNLLAEKQYGFCKATLTEKVGGE

DVAVTCMVFQTQPVVPQPQPDGTNAAAPTPAVDPATAEPSPAGPPVAAVVVGPLVLAAPQ

GPPLHRAHYDLRHAFTGVVSVESASGEAFHPVKPPVVVGPGPRVHSCPGRIRYFKV

>Sarcophilus (G3VEN1 - G3VEN1_SARHA)

MRSLVAFICLAQLLSWAAARSPGVPVAPVRDPDCDDLDVEQAALAAVQYVNNNHKHGYKY

TLNQVDKARVYQRRPTGEVYVLEIDLVETCCHVLDPTPLENCTVRQLTEHAVEGDCDVSV

LKVDNQFMVLNAKCESSPDSAEDVRKICPNCPLLAPLNDTKVVHAVEAALTAFNAQKHNN

YFKLQEISRAQFSPLQSSVYTEFVTVPTDCKPIDVIDPAACNPLPNQHGFCKGTLTEKVG

GGEDVVATCTVFVAVPAVPAPQPDGIESSLGPAVPAEPAVPAGPPVAAGPAVALGPLLVD

APAVLPAKPILTHLPHHDLRHSIVGGSSFESASGEYGLNAGPVAPGPGPGPAAPAIPLCP

GRIRHFKI

>Monodelphis (F6Q5X0 - F6Q5X0_MONDO)

MRSLVAFLCLAQLLSHAAARSHPLLPLRDPDCDDPDVELAAMEITQHVNSQLQSGYKYRL

NQIDKVRVYPRRPAGEVYVMEVDMLETTCHVMDKTPLENCTVRQLAEHAVEGDCEASVLK

MDGQYKVLNVKCESNPDSAEDVRKVCPQCSLLAPLNDTRVVHAVEASLAAFISQNQSTFY

KLLEVSRAQISPIQPSVYVEFVIAPTDCGPKGADDPSSCHPLTDQYGFCKGTVTKKDGGE

DVAVSCTISVRTLVQISSVPFGGHEHSSFLLYEMKPAPTPQGDIVRIQYCVPAPVGPVVR

PVRPFISHLPHHDLRHSIVGVASLESASGEHGPVVGLAAPAVPGAAAPGVPLCPGRIRHF

KV

>Pongo (H2PC98 - H2PC98_PONAB)

MKSLVLLLCLAQLWGCHSAPHGPGLIYRQPNCDDPETEEAALVAIDYINQNHPWGYKHTL

NQIDEVKVWPRQPSGELFEIEIDTLETTCHVLDPTPVARCSVRQLKEHAVEGDCDFKLLK

LDGKFSVVYAKCDSSPDSAEDVRKVCQDCPLLAPLNDTRVVHAAEAALAAFNAQNNRSHF

QLEEISRAQLVPLPPSTYVEFTMSGTDCVAKEDTEAAKCNLLAEKQYGFCKATLSEKLGG

EEVAVTCTVFQTQPVTSQPQPEGANEAVPTPVVDPDAPPSPPLGAPGPPPAGSPPYSHVL

AAASPAHQLYRAHYDLRHTFMGVVSWRSLSGEVSHPRKTRTVVQPSVGAAAGPVVPPCPG

RIRHFKV

>Callithrix (F7GYN3 - F7GYN3_CALJA)

MKSLVLLLCLAQLWGCHSAPQGQGLHYRQLNCDDPEAEEAALAVDYINQNLPSIQTHLET

KTETVKKSPQRPFGEVFVMEIDTLETTCHARDPTPVANCSVRQLTEHAVEGDCDFRLLKL

NGQFSVFYAKCHSSPDSAEDVRKVCQDCPLLAPLNDTRVVHAADAALAAFNAQNNGSFFQ

LEEVSRAQLVPLPPSTYVEFTVSAPDCVAKEATEAAKCNLLAKKQYGFCKATLTEKLNGE

EVAVTCTVFQPQPEAPQPQPEVADVVGPKPAADPDVHVPLPSDTAANPQASPPLRRVAPQ

VLPWHRAHYDLRHIFLGGVSMESTSGEAWHLRKTRAVGAAAAPAVPLCPGRIRHFKV

>Mus (P29699 – FETUA_MOUSE)

MKSLVLLLCFAQLWGCQSAPQGTGLGFRELACDDPEAEQVALLAVDYLNNHLLQGFKQVL

NQIDKVKVWSRRPFGVVYEMEVDTLETTCHALDPTPLANCSVRQLTEHAVEGDCDFHILK

QDGQFRVMHTQCHSTPDSAEDVRKLCPRCPLLTPFNDTNVVHTVNTALAAFNTQNNGTYF

KLVEISRAQNVPLPVSTLVEFVIAATDCTAKEVTDPAKCNLLAEKQHGFCKANLMHNLGG

EEVSVACKLFQTQPQPANANAVGPVPTANAALPADPPASVVVGPVVVPRGLSDHRTYHDL

RHAFSPVASVESASGETLHSPKVGQPGAAGPVSPMCPGRIRHFKI

**Albumin**

>Gallus (P19121 - ALBU_CHICK)

MKWVTLISFIFLFSSATSRNLQRFARDAEHKSEIAHRYNDLKEETFKAVAMITFAQYLQR

CSYEGLSKLVKDVVDLAQKCVANEDAPECSKPLPSIILDEICQVEKLRDSYGAMADCCSK

ADPERNECFLSFKVSQPDFVQPYQRPASDVICQEYQDNRVSFLGHFIYSVARRHPFLYAP

AILSFAVDFEHALQSCCKESDVGACLDTKEIVMREKAKGVSVKQQYFCGILKQFGDRVFQ

ARQLIYLSQKYPKAPFSEVSKFVHDSIGVHKECCEGDMVECMDDMARMMSNLCSQQDVFS

GKIKDCCEKPIVERSQCIMEAEFDEKPADLPSLVEKYIEDKEVCKSFEAGHDAFMAEFVY

EYSRRHPEFSIQLIMRIAKGYESLLEKCCKTDNPAECYANAQEQLNQHIKETQDVVKTNC

DLLHDHGEADFLKSILIRYTKKMPQVPTDLLLETGKKMTTIGTKCCQLGEDRRMACSEGY

LSIVIHDTCRKQETTPINDNVSQCCSQLYANRRPCFTAMGVDTKYVPPPFNPDMFSFDEK

LCSAPAEEREVGQMKLLINLIKRKPQMTEEQIKTIADGFTAMVDKCCKQSDINTCFGEEG

ANLIVQSRATLGIGA

>Bos (P02769 - ALBU_BOVIN)

MKWVTFISLLLLFSSAYSRGVFRRDTHKSEIAHRFKDLGEEHFKGLVLIAFSQYLQQCPF

DEHVKLVNELTEFAKTCVADESHAGCEKSLHTLFGDELCKVASLRETYGDMADCCEKQEP

ERNECFLSHKDDSPDLPKLKPDPNTLCDEFKADEKKFWGKYLYEIARRHPYFYAPELLYY

ANKYNGVFQECCQAEDKGACLLPKIETMREKVLASSARQRLRCASIQKFGERALKAWSVA

RLSQKFPKAEFVEVTKLVTDLTKVHKECCHGDLLECADDRADLAKYICDNQDTISSKLKE

CCDKPLLEKSHCIAEVEKDAIPENLPPLTADFAEDKDVCKNYQEAKDAFLGSFLYEYSRR

HPEYAVSVLLRLAKEYEATLEECCAKDDPHACYSTVFDKLKHLVDEPQNLIKQNCDQFEK

LGEYGFQNALIVRYTRKVPQVSTPTLVEVSRSLGKVGTRCCTKPESERMPCTEDYLSLIL

NRLCVLHEKTPVSEKVTKCCTESLVNRRPCFSALTPDETYVPKAFDEKLFTFHADICTLP

DTEKQIKKQTALVELLKHKPKATEEQLKTVMENFVAFVDKCCAADDKEACFAVEGPKLVV

STQTALA

>Ovis (P14639 - ALBU_SHEEP)

MKWVTFISLLLLFSSAYSRGVFRRDTHKSEIAHRFNDLGEENFQGLVLIAFSQYLQQCPF

DEHVKLVKELTEFAKTCVADESHAGCDKSLHTLFGDELCKVATLRETYGDMADCCEKQEP

ERNECFLNHKDDSPDLPKLKPEPDTLCAEFKADEKKFWGKYLYEVARRHPYFYAPELLYY

ANKYNGVFQECCQAEDKGACLLPKIDAMREKVLASSARQRLRCASIQKFGERALKAWSVA

RLSQKFPKADFTDVTKIVTDLTKVHKECCHGDLLECADDRADLAKYICDHQDALSSKLKE

CCDKPVLEKSHCIAEVDKDAVPENLPPLTADFAEDKEVCKNYQEAKDVFLGSFLYEYSRR

HPEYAVSVLLRLAKEYEATLEDCCAKEDPHACYATVFDKLKHLVDEPQNLIKKNCELFEK

HGEYGFQNALIVRYTRKAPQVSTPTLVEISRSLGKVGTKCCAKPESERMPCTEDYLSLIL

NRLCVLHEKTPVSEKVTKCCTESLVNRRPCFSDLTLDETYVPKPFDEKFFTFHADICTLP

DTEKQIKKQTALVELLKHKPKATDEQLKTVMENFVAFVDKCCAADDKEGCFVLEGPKLVA

STQAALA

>Sus (P08835 - ALBU_PIG)

MKWVTFISLLFLFSSAYSRGVFRRDTYKSEIAHRFKDLGEQYFKGLVLIAFSQHLQQCPY

EEHVKLVREVTEFAKTCVADESAENCDKSIHTLFGDKLCAIPSLREHYGDLADCCEKEEP

ERNECFLQHKNDNPDIPKLKPDPVALCADFQEDEQKFWGKYLYEIARRHPYFYAPELLYY

AIIYKDVFSECCQAADKAACLLPKIEHLREKVLTSAAKQRLKCASIQKFGERAFKAWSLA

RLSQRFPKADFTEISKIVTDLAKVHKECCHGDLLECADDRADLAKYICENQDTISTKLKE

CCDKPLLEKSHCIAEAKRDELPADLNPLEHDFVEDKEVCKNYKEAKHVFLGTFLYEYSRR

HPDYSVSLLLRIAKIYEATLEDCCAKEDPPACYATVFDKFQPLVDEPKNLIKQNCELFEK

LGEYGFQNALIVRYTKKVPQVSTPTLVEVARKLGLVGSRCCKRPEEERLSCAEDYLSLVL

NRLCVLHEKTPVSEKVTKCCTESLVNRRPCFSALTPDETYKPKEFVEGTFTFHADLCTLP

EDEKQIKKQTALVELLKHKPHATEEQLRTVLGNFAAFVQKCCAAPDHEACFAVEGPKFVI

EIRGILA

>Equus (Q5XLE4 - ALBU_EQUAS)

MKWVTFVSLLFLFSSAYSRGVLRRDTHKSEIAHRFNDLGEKHFKGLVLVAFSQYLQQCPF

EDHVKLVNEVTEFAKKCAADESAENCDKSLHTLFGDKLCTVATLRATYGELADCCEKQEP

ERNECFLTHKDDHPNLPKLKPEPDAQCAAFQEDPDKFLGKYLYEVARRHPYFYGPELLFH

AEEYKADFTECCPADDKLACLIPKLDALKERILLSSAKERLKCSSFQNFGERAVKAWSVA

RLSQKFPKADFAEVSKIVTDLTKVHKECCHGDLLECADDRADLAKYICEHQDSISGKLKA

CCDKPLLQKSHCIAEVKEDDLPSDLPALAADFAEDKEICKHYKDAKDVFLGTFLYEYSRR

HPDYSVSLLLRIAKTYEATLEKCCAEADPPACYRTVFDQFTPLVEEPKSLVKKNCDLFEE

VGEYDFQNALIVRYTKKAPQVSTPTLVEIGRTLGKVGSRCCKLPESERLPCSENHLALAL

NRLCVLHEKTPVSEKITKCCTDSLAERRPCFSALELDEGYVPKEFKAETFTFHADICTLP

EDEKQIKKQSALAELVKHKPKATKEQLKTVLGNFSAFVAKCCGREDKEACFAEEGPKLVA

SSQLALA

>Felis (P49064 - ALBU_FELCA)

MKWVTFISLLLLFSSAYSRGVTRREAHQSEIAHRFNDLGEEHFRGLVLVAFSQYLQQCPF

EDHVKLVNEVTEFAKGCVADQSAANCEKSLHELLGDKLCTVASLRDKYGEMADCCEKKEP

ERNECFLQHKDDNPGFGQLVTPEADAMCTAFHENEQRFLGKYLYEIARRHPYFYAPELLY

YAEEYKGVFTECCEAADKAACLTPKVDALREKVLASSAKERLKCASLQKFGERAFKAWSV

ARLSQKFPKAEFAEISKLVTDLAKIHKECCHGDLLECADDRADLAKYICENQDSISTKLK

ECCGKPVLEKSHCISEVERDELPADLPPLAVDFVEDKEVCKNYQEAKDVFLGTFLYEYSR

RHPEYSVSLLLRLAKEYEATLEKCCATDDPPACYAHVFDEFKPLVEEPHNLVKTNCELFE

KLGEYGFQNALLVRYTKKVPQVSTPTLVEVSRSLGKVGSKCCTHPEAERLSCAEDYLSVV

LNRLCVLHEKTPVSERVTKCCTESLVNRRPCFSALQVDETYVPKEFSAETFTFHADLCTL

PEAEKQIKKQSALVELLKHKPKATEEQLKTVMGDFGSFVDKCCAAEDKEACFAEEGPKLV

AAAQAALA

>Canis (P49822 - ALBU_CANFA)

MKWVTFISLFFLFSSAYSRGLVRREAYKSEIAHRYNDLGEEHFRGLVLVAFSQYLQQCPF

EDHVKLAKEVTEFAKACAAEESGANCDKSLHTLFGDKLCTVASLRDKYGDMADCCEKQEP

DRNECFLAHKDDNPGFPPLVAPEPDALCAAFQDNEQLFLGKYLYEIARRHPYFYAPELLY

YAQQYKGVFAECCQAADKAACLGPKIEALREKVLLSSAKERFKCASLQKFGDRAFKAWSV

ARLSQRFPKADFAEISKVVTDLTKVHKECCHGDLLECADDRADLAKYMCENQDSISTKLK

ECCDKPVLEKSQCLAEVERDELPGDLPSLAADFVEDKEVCKNYQEAKDVFLGTFLYEYAR

RHPEYSVSLLLRLAKEYEATLEKCCATDDPPTCYAKVLDEFKPLVDEPQNLVKTNCELFE

KLGEYGFQNALLVRYTKKAPQVSTPTLVEVSRKLGKVGTKCCKKPESERMSCAEDFLSVV

LNRLCVLHEKTPVSERVTKCCSESLVNRRPCFSGLEVDETYVPKEFNAETFTFHADLCTL

PEAEKQVKKQTALVELLKHKPKATDEQLKTVMGDFGAFVEKCCAAENKEGCFSEEGPKLV

AAAQAALV

>Homo (P02768 - ALBU_HUMAN)

MKWVTFISLLFLFSSAYSRGVFRRDAHKSEVAHRFKDLGEENFKALVLIAFAQYLQQCPF

EDHVKLVNEVTEFAKTCVADESAENCDKSLHTLFGDKLCTVATLRETYGEMADCCAKQEP

ERNECFLQHKDDNPNLPRLVRPEVDVMCTAFHDNEETFLKKYLYEIARRHPYFYAPELLF

FAKRYKAAFTECCQAADKAACLLPKLDELRDEGKASSAKQRLKCASLQKFGERAFKAWAV

ARLSQRFPKAEFAEVSKLVTDLTKVHTECCHGDLLECADDRADLAKYICENQDSISSKLK

ECCEKPLLEKSHCIAEVENDEMPADLPSLAADFVESKDVCKNYAEAKDVFLGMFLYEYAR

RHPDYSVVLLLRLAKTYETTLEKCCAAADPHECYAKVFDEFKPLVEEPQNLIKQNCELFE

QLGEYKFQNALLVRYTKKVPQVSTPTLVEVSRNLGKVGSKCCKHPEAKRMPCAEDYLSVV

LNQLCVLHEKTPVSDRVTKCCTESLVNRRPCFSALEVDETYVPKEFNAETFTFHADICTL

SEKERQIKKQTALVELVKHKPKATKEQLKAVMDDFAAFVEKCCKADDKETCFAEEGKKLV

AASQAALGL

# >Pan (ENSPTRT00000064657)

MKWVTFISLLFLFSSAYSRGVFRRDAHKSEVAHRFKDLGEENFKALVLVAFAQYLQQCPF

EDHVKLVNEVTEFAKTCVADESAENCDKSLHTLFGDKLCTVATLREKYGEMADCCAKQEP

ERNECFLQHKDDNPNLPRLVRPEVDVMCTAFHDNEGTFLKKYLYEVARRHPYFYAPELLF

FAERYKAAFTECCQAADKAACLLPKLDELRDEGKASSAKQRLKCASLQKFGERAFKAWAV

ARLSQRFPKAEFAEVSKLVTDLTKVHTECCHGDLLECADDRADLAKYICENQDSISSKLK

ECCEKPLLEKSHCLAEVENDEMPADLPSLAADFVESKEVCKNYAEAKDVFLGMFLYEYAR

RHPDYSVVLLLRLAKTYETTLEKCCAAADPHECYAKVFDEFKPLVEEPQNLIKQNCELFE

QLGEYKFQNALLVRYTKKVPQVSTPTLVEVSRNLGKVGSKCCKHPEAKRMPCAEDYLSVV

LNQLCVLHEKTPVSDRVTKCCTESLVNRRPCFSALEVDETYVPKEFNAETFTFHADICTL

SEKERQIKKQTALVELVKHKPKATKEQLKAVMDDFAAFVEKCCKADDKETCFAEEGKKLV

AASQAALGL

>Meriones (O35090 - ALBU_MERUN)

MKWVTFLLLLFVSGSAFSRGVFRRDAAHKSEIAHRYKDLGEKYFKGLVLYTFSQYLQKCS

YEEHVKLVREVTDFASNCAKDESAENCDKSLHTLFGDKLCSLPNFGEKYAEMADCCAKQE

PERNECFLQHKDDNPQLPPFKRAEPDAMCTAFQENAEAFMGHYLHEVARRHPYFYGPELL

YLADKYTAVLTECCAADDKGACLTPKLDALKEKALVSAVRQRLKCSSMKKFGERAFKAWA

VARMSQTFPNADFAEITKLATDLTKVTQECCHGDLLECADDRAELAKYMCENQASISSKL

QACCDKEMLQKSQCLAEVEHDDMPADLPALTADFVEDKDVCKNYAEAKDVFLGTFLYEYS

RRHPEYSVSLLLRLAKKYEATLEKCCAEADPHACYGHVFDEFKPLVEEPQNLVKSNCELY

EKLGEYGFQNAVLVRYTKKAPQVSTPTLVEAARSLGRVGTHCCALPEKKRLPCVEDYLSA

ILNRVCLLHEKTPVSEQVTKCCSGSLVERRPCFSALPVDETYVPKEFKAETFTFHANICT

LPEKEKQMEKQTALAELVKHKPQATEEQLKKVMGDFAEFLEKCCKQEDKEACFSTEGPKL

VAESQKALA

>Pteropus (L5K939 - L5K939_PTEAL)

MKWVTFISLLFLFSSAYSRGVFRRDTHKSEIAHRYNDLGEEHFRELVLITFSQYLQQCPF

DEHSKLTNEVTEFAKTCVADESAANCDKSLHTLFGDKLCTVASLRETYGELADCCEKQDP

ERNECFLKHKDDDPNLPPVVKPEPEALCTAFQENNNKFLENYLYEVARRHPYFYGPELLY

YVKQYKAILTECCQAADKATCLAPKAKVLKEKLLASSAKQRHKCASIQKFGERAFKAWSI

ARLSQRFPKADFMDLSKLVTDLSKIHKECCHGDLLECADDREDLAKYVCDNQDSFSSKLK

ECCDKPLLEKSHCISELENDDLPNDLPSITTDFVEDKDVCKNYKEAKDVFLGTFLYEYSR

RHPEYAVSLLLRIAKGYEATLERCCATDDAHACYSKVFDELQPLVDEPQKLMKRNCELFE

NLGAYGFQNALIIRYTKKMPQVSTPTLLVISKELANMGNKCCTLPESKRMACAEDYLSLV

LNRLCVLHEKTPVSDKITKCCTDSLVNRRPCFSSLEADETYVPKEFNAETFTFHADVCTL

PDHEKHLKTQTALVELLKHKPKAADEQLKTVMGNFSAFIEKCCTADDKEACFAEEVL

>Rattus (P02770 - ALBU_RAT)

MKWVTFLLLLFISGSAFSRGVFRREAHKSEIAHRFKDLGEQHFKGLVLIAFSQYLQKCPY

EEHIKLVQEVTDFAKTCVADENAENCDKSIHTLFGDKLCAIPKLRDNYGELADCCAKQEP

ERNECFLQHKDDNPNLPPFQRPEAEAMCTSFQENPTSFLGHYLHEVARRHPYFYAPELLY

YAEKYNEVLTQCCTESDKAACLTPKLDAVKEKALVAAVRQRMKCSSMQRFGERAFKAWAV

ARMSQRFPNAEFAEITKLATDVTKINKECCHGDLLECADDRAELAKYMCENQATISSKLQ

ACCDKPVLQKSQCLAEIEHDNIPADLPSIAADFVEDKEVCKNYAEAKDVFLGTFLYEYSR

RHPDYSVSLLLRLAKKYEATLEKCCAEGDPPACYGTVLAEFQPLVEEPKNLVKTNCELYE

KLGEYGFQNAVLVRYTQKAPQVSTPTLVEAARNLGRVGTKCCTLPEAQRLPCVEDYLSAI

LNRLCVLHEKTPVSEKVTKCCSGSLVERRPCFSALTVDETYVPKEFKAETFTFHSDICTL

PDKEKQIKKQTALAELVKHKPKATEDQLKTVMGDFAQFVDKCCKAADKDNCFATEGPNLV

ARSKEALA

>Otolemur (H0X1I5 - H0X1I5_OTOGA)

MKWVTFISLLFLFSSAYSRGVFRRDTHKSELAHRYNDLGEEHFKALVLVTFSQFLQKCPF

EDHVKLVNEVTEFAKTCAADESAENCDKSLHTLLGDKLCTVASLREKYGEMADCCAKQEP

ERNQCFLDHKDDKPDLPPLVRPEVDVMCTSFQDNEKTFLGHYLYEVARRHPYFYGPELLF

FAEKYKAAFTECCQAADKAACLLPKLDTLKEEGMAASAKQRLKCSSLEKFGDRAFKAWAV

ARLSQRFPKAEFAEVSKLVTDLTKVHTECCHGDLLECADDRADLAKYMCENQDSLSSKLK

ECCEKPLLEKSHCLAHVENDDLPTDLAPLAADFVEDKDVCKNYAEAKDVFLGTFLYEYSR

RHPEYSVFLLLRLAKHYEATLEKCCATDDPHTCYAKVLDELQHDVEEPQNLVKQNCELFE

KLGEYGFQNALLVRYTKKVPQVSTPTLVEVSRSLGRVGSKCCKMDEAKRMGCAEDYLSVV

LNRLCVLHEKTPVSDRVTKCCTESLVNRRPCFSALEVDETYVPKEFHAETFTFHADMCTL

PDKEKQLKKQTALVELVKHKPKATDEQLKAVMGKFTDFVEKCCKADDKEACFAEEGPKLV

AESQAALA

>Gorilla (G3RE98 - G3RE98_GORGO)

MKWVTFISLLFLFSSAYSRGVFRRDAHKSEVAHRFKDLGEETFKALVLVAFAQYLQQCPF

EDHVKLVNEVTEFAKTCVADESAENCDKSLHTLFGDKLCTVATLRETYGEMADCCAKQEP

ERNECFLQHKDDNPNLPRLVRPEVDVMCTAFHDNEETFLKKYLYEIARRHPYFYAPELLF

FAARYKAAFTECCQAADKAACLLPKLDELRDEGKASSAKQRLKCASLQKFGERAFKAWAV

ARLSQRFPKAEFAEVSKLVTDLTKVHTECCHGDLLECADDRADLAKYICENQDSISSKLK

ECCEKPLLEKSHCLAEVENDEMPADLPSLAADFVESKDVCKNYAEAKDVFLGMFLYEYAR

RHPDYSVVLLLRLAKTYETTLEKCCAAADPHECYAKVFDEFKPLVEEPQNLIKQNCELFE

QLGEYKFQNALLVRYTKKVPQVSTPTLVEVSRNLGKVGSKCCKHPEAKRMPCAEDYLSVV

LNQLCVLHEKTPVSDRVTKCCTESLVNRRPCFSALEVDETYVPKEFNAETFTFHADICTL

SEKERQIKKQTLSALAELVKHKPKATKEQLKTVMDDFAAFVEKCCKADDKETCFAEEGKK

LVAASQAALGL

>Ailuropoda (G1LEJ5 - G1LEJ5_AILME)

MKWVTFISLLFLFSSAYSRGVTRREAQQSEIAHRYNDLGEEHFRGLVLVAFSQYLQQCPF

EDHAKLAKEVTEFARGCAADQSGADCGKSLHTLFGDKLCTVASLREKYGELADCCEKQEP

ERNECFLKHKDDNPGFPPLVTPEPDALCAAFQENEQRFLGKKCRYLYEVARRHPYFYGPE

LLYYAQQYKGVFAECCQAADKAACLTPKIDDLREKVLVSSAKERFKCASLQKFGDRAFKA

WSIARLSQKFPKADFAEVSKVVTDLTKVHKECCHGDLLECADDRADLAKYMCENQDSISS

KLKECCDKPVLEKSQCLSEVEKDELPGDLPLLAADFVEDKEVCKNYQEAKDVFLGTFLYE

YSRRHPEYSVSLLLRLAKEYEATLEKCCATDDPPTCYGKVLDEFKPLVEEPQNLVKANCE

LFEKLGEYGFQNALLVRYTKKVPQVSTPTLVEVSRKLGKVGTKCCKKPESERMSCAEDYL

SVVLNRLCVLHEKTPVSERVTKCCTESLVNRRPCFSALEVDETYVPKEFNAETFTFHADL

CTLPEAEKQVKKQSCSALVELLKHKPKATEEQLKTVMGDFGAFVDKCCAAENKEGCFAEE

GPKLVATAQAALV

>Spermophilus (I3MJG0 - I3MJG0_SPETR)

MKWVTFIFLLFLFSSAYSRGVFRREAGKSEIIKRFREFGEQEFKGLVLVTFSQILQKTSY

DDLAKFTTEVTDLAKACVADEFGVDCNKPLDTIFGEEACKIATLRDTYGDMADCCANQDL

ERFQCLIKYKEDTPTIPPLHPIDPDALCISFDESSQNVLGHFVYEVARRNPYLCGQKVMY

FAEKYKGFLTECCKAADKGECLTQKTENLKKTIMLSSAKDRFKCSVLEKYEERGLKAWLI

SRLSRKFPQAEFTEITKIATDLTKIIKESCSGDLLESTHDRVTLANYICENQDKISKKVG

ECCVKPLLERYHCIIDLEEDDKLADLPALTADYAEDKDVCKNYAEAKDIFLGMFLYEYSR

RHPEYGSLLLLRIAKAYEAKLEKCCAEADPPACYGKVFEEFEPLATEPQNVVKQNCDLYE

QLGEYKFQNALLIRYTQKAPQVSTPTLVEASRNLGRVGTKCCKLPESHRMPCVEDYLTAI

LNTVCVMHEKNPVSERITKCCSESFVNKRACFSALSVDDTYVPKEFHADTFTFHADICTL

PETEQQIKKQTALAELVKHKPTATIDQLKTVMGDFVAFLDKCCKADDKDTCFSQEGPKFV

AASQAALA

>Nomascus (G1R8T8 - G1R8T8_NOMLE)

MKWVTFISLLLLFSSAYSRGVFRRDAHKSEIAHRFKDLGEENFKALVLVAFAQYLQQCPF

EDHVKLVNEVTEFAKTCVADESAENCDKSLHTLFGDKLCTVATLRETYGEMADCCAKQEP

ERNECFLQHKDDNPNLPPLVRPEVDVMCTAFHDNEETFLKKYLYEIARRHPYFYAPELLF

FAERYKAAFTECCQAADKAACLLPKLDGLRDEGKASSAKQRLKCASLQKFGERAFKAWAV

ARLSQRFPKAEFAEVSKLVTDLTKVHTECCHGDLLECADDRADLAKYICENQDSISSKLK

ECCEKPLLEKSHCLAEVENDEMPADLPSLAADFVESKDVCKNYAEAKDVFLGMFLYEYAR

RHPDYSVVLLLRLAKAYETTLEKCCAAADPHECYAKVFDEFKPLVEEPQNLIKHNCELFE

QLGEYKFQNELLVRYTKKVPQVSTPTLVEVSRNLGKVGSKCCKHPEAQRMPCTEDYLSVV

LNRLCVLHEKTPVSDRVTKCCTESLVNRRPCFSALEVDETYVPKEFNAETFTFHADICTL

SEKDRQVKKQTALVELVKHKPKATKEQLKTVMEDFAAFVEKCCKADDKETCFAEEGKKLV

AASQAALGL

>Loxodonta (Q6B3Z0 - Q6B3Z0_ELEMA)

MKWVIFISLSFLFSSAYSRGVFRREAYKSEIAHRYKDLGEDLFKGLLLISFAQYLQKSPY

DEHVQSVTAVTDLAKTCAADESAEHCGDSLHTIFGDKLCARVTAHQDTYGEFAECCGKQE

PERNECFLKHKDDNPALPPLVRPPADALCASFEENERKFFGVYLYEVARRHPYFYAPELI

YYSEKYKDILTECCHADDKAACLKPKIDALKDTVLASAARQRLKCANIHKFGERAFKAWA

VTHLSQRFPKADFAEVSKLATDLGKVYQECCHGDLLECADDRADLAKYICDNQETLSSKL

KECCDKPVLAKSHCIAELDKDDPPADLPSIVPDYVEDKDVCKNYQEAKDIFLGTFLYEYS

RRHPEYSAFLLLRIAKEYEATLERCCAAADPHACYAKVLDAFTPLVEEPQGLVKQNCEHF

EQLGEYGFTNELVVRYTRKVPQVSTPTLVEVSRKLGRVGSKCCKLAEAKRMPCAEDYLSL

VLNRLCVLHEKTPVSERITKCCTESLVNRRPCFTALQTDETYVPKEFNAETFTFHADLCT

LPEDQKQIKKQSVLVELVKHKPKATDEQLKTVTGQFTGMVEKCCAAEDKEACFAEEGPKL

VTASQAALL

>Sarcophilus (G3WQH5 - G3WQH5_SARHA)

MKWVTFISLIFLFSSVHSREIFRRDAPTSEIAKRYRDLGEENVKGLVLITFAQYLQKCPF

EDHVKLVSEVVEFAKGCAADESAENCGKSLHELLGDKLCKVPSLRENYGEMADCCAKEEP

ERHRCFLSHKDDQPNLPKIEAPEPETLCKNFQENENRVLGFYLYEVARRHPYFYAPALLA

YAHQYKEAVKGCCQEADKGACFDNKLSVLRDKVISSGAKQRFRCSSLDEFGERAVKAGLV

ARLSQKFPKADFAEIHKIVEDLAKIHTECCHGDLLECADDRVALSEYLCNNKETISTKLG

ECCDKPLIEKSQCIADLGNDAIPDGLPDLLAEYVNTKDACQNYKEAKDLFLATYLYDNGR

RHPELAVTTLLRIAKDYETTLEECCATADPPTCYAKAGEHRKNFIDESQNLIKENCDLFD

KVGEYGFENALLVRYTRKAPQVSTPTLLALGHKLAKVGSKCCKVSDNEKLGCAEGYLAIV

IDNLCRLHEKTPVSDRITKCCKDSLVNRRPCLSALGVDDTYVAKPFSADTFTFHADLCTL

PEEEKQDKKQGILVELVKHKPKITDDQLKGVITDFTAFVDKCCKADNQEACFAEDGPKLV

ATTQAALA

>Monodelphis (F7BJP5 - F7BJP5_MONDO)

MKWVTFISLIFLFSSVYSRDLFRRDAPKSEIAKRYRDLGEENVKALVLITFAQYLQKCPF

EDHVKLVDEVVQFAKGCAADETAENCGKSLHQLLGDKLCKIASLRESYGEMADCCAKEEP

ERNQCFLSHKDDHPDLPKIVAPEPDKLCQEFQENENKVMGYYLHQVARRHPYFYAPALLA

YAIQYRDAVRECCAAADKATCLNDKLTDLREKVLSAGAKQRFRCASLDKFGERAVKAGLV

ARLSQKFPKADFAEIHKIVEDLANVHKECCHGDLLECADDRAALSDYVCQNKDSISSKLT

KCCDKSLVEKSQCIADLENDDLPADLPDFDETYVTSKEACQNYKEAKDLFLANYLYDSAR

RGANLAVTTLLRLAKGYETTLEECCATDDPPSCYAKVTLQRKAIIDDSNNLVKQNCDIFE

KAGEYGFANELVVRYTKKMPQVSTPTVVELSQKLAKVGSKCCKLSDDEKMGCAEGYLAIV

VDKLCRQHEKTPVSDKITKCCTESLVNRRPCFTALGVDETYEPKAFSADTFTFHADLCTL

PEEEKQTKKQTVLAELVKHKPKITQDQLKAVISDFTAFVDKCCKADSQEGCFAEDGPKLV

ASAQAALA

>Pongo (Q5NVH5 - ALBU_PONAB)

MKWVTFISLLFLFSSAYSRGVFRRDAHKSEVAHRFKDLGEEKFKALVLIAFAQYLQQCPF

EDHVKLVNEVTEFAKTCVADESAENCDKSLHTLFGDKLCTVATLRETYGEMADCCAKQEP

ERNECFLQHKDDNPNLPRLVRPEVDVMCTAFHDNEETFLKKYLYEIARRHPYFYAPELLF

FAVRYKAAFTECCQAADKAACLLPKLDELRDEGKASSAKQRLKCASLQKFGERAFKAWAV

ARLSQRFPKAEFAEVSKLVTDLTKVHTECCHGDLLECADDRADLAKYICENQDSISSKLK

ECCEKPLLEKSHCLAEVENDEMPADLPSLAADFVESKDVCKNYAEAKDVFLGMFLYEYAR

RHPDYSVVLLLRLAKTYETTLEKCCAAADPHECYAKVFDEFKPLVEEPQNLIKQNCELFE

QLGEYKFQNELLVRYTKKVPQVSTPTLVEVSRNLGKVGSKCCKHPEPKRMPCAEDYLSVV

LNQLCVLHEKTPVSERVTKCCTESLVNRRPCFSALEVDETYVPKEFNADTFTFHADICTL

SEKERQIKKQTALVELVKHKPKATKEQLKTVMEDFAAFVEKCCKADDKETCFAEEGKKLV

AASQAALGL

>Callithrix (F7CSG0 - F7CSG0_CALJA)

MKWVTFISLLFLFSSAYSRGVFRRDAHKSEVAHRFKDLGEEHFKDLVLVPFSQYLQQCPF

EEHLKLVNEVTEFAKTCVADETAENCDKSLHTLFGDKLCTVATLRETYGDMADCCAKQEP

ERNECFLQHKDDKPDLPPLVRPEVDVMCTAFQEHEDTFLKKYLYEVARRHPYFYAPELLF

FAQKYKAAFTECCQAADKGACLLPKLDELRDQGKASSAKQRLKCASLQKFGERAFKAWSV

ARVSQRFPKADFTEVTKVVTDLTKVHTECCHGDLLECADDRAELAKYMCENQDSLSSKLK

ECCEKPLLEKSHCLAHVENDEMPADLHALTDDYVESKDVCKNYAEAKDVFMGMFLYEYSR

RHPDYSAMLLLRLAKAYEATLEKCCATADPHGCYAKVFDEFQPLVEDPQNLVKHNCELFE

QFGEYKFQNELLVRYTKKVPQVSTPTLVEVSRNLGKVGTKCCKQPEAKRMPCVEDYLSVV

LNQLCVLHEKTPVSDRVTKCCTESLVNRRPCFSALEVDETYLPKAFNAETFTFHADMCTL

SEKEQQVKKQTALAELVKHKPKATNEQLKTVMEDFAAFVEKCCKADDKEACFAEEGPKLV

AASETALA

>Mus (P07724 - ALBU_MOUSE)

MKWVTFLLLLFVSGSAFSRGVFRREAHKSEIAHRYNDLGEQHFKGLVLIAFSQYLQKCSY

DEHAKLVQEVTDFAKTCVADESAANCDKSLHTLFGDKLCAIPNLRENYGELADCCTKQEP

ERNECFLQHKDDNPSLPPFERPEAEAMCTSFKENPTTFMGHYLHEVARRHPYFYAPELLY

YAEQYNEILTQCCAEADKESCLTPKLDGVKEKALVSSVRQRMKCSSMQKFGERAFKAWAV

ARLSQTFPNADFAEITKLATDLTKVNKECCHGDLLECADDRAELAKYMCENQATISSKLQ

TCCDKPLLKKAHCLSEVEHDTMPADLPAIAADFVEDQEVCKNYAEAKDVFLGTFLYEYSR

RHPDYSVSLLLRLAKKYEATLEKCCAEANPPACYGTVLAEFQPLVEEPKNLVKTNCDLYE

KLGEYGFQNAILVRYTQKAPQVSTPTLVEAARNLGRVGTKCCTLPEDQRLPCVEDYLSAI

LNRVCLLHEKTPVSEHVTKCCSGSLVERRPCFSALTVDETYVPKEFKAETFTFHSDICTL

PEKEKQIKKQTALAELVKHKPKATAEQLKTVMDDFAQFLDTCCKAADKDTCFSTEGPNLV

TRCKDALA

Alpha 2 HS Glycoprotein Sequences showing variation throughout 23 different species (dots indicate conserved residue; highlight indicates two peptides matched in every sample)

10 20 30 40 50 60 70 80 90 100

....|....|....|....|....|....|....|....|....|....|....|....|....|....|....|....|....|....|....|....|

**Bos**  **MKSFVLLFCLAQL--WGCHSIPLDPVAGYKEPACDDPDTEQAALAAVDYINKHLPRGYKHTLNQIDSVKVWPRRPTGEVYDIEIDTLETTCHVLDPTPLA**

**Ovis**  **....L........--CS.R......I.........................................................................V**

**Sus**  **---LI.F......--...RAV.HG.IL..R......VE..........................V..........A...F....................**

**Equus**  **.............--CH.L.L.NGLSPA.RQLN....E......L......S.IHQ....V.....K.Q..A-Q....SFKL..........A.......**

**Felis**  **...LA..L.....--....AA.HI.GLV.R.LD....E......V......N.VLQ..........K........M...FEL.........YIR....V.**

**Canis**  **...LA.FL.....--.....V.VG.ALV.R.LD....E......V......N.VL...........K........M...FEL..................**

**Homo**  **...L...L.....--.....A.HG.GLI.RQ.N....E..E...V.I....QN..W..........E.....QQ.S..LFE.................V.**

**Pan**  **...L...L.....--.....A.RGLGLI.RQ.N....E..E...V.I....QNH.W..........E......Q.S..LFE.................V.**

**Meriones**  **..TL...L.FTL.--...Q.A.QGTGL.FR.V.....EV..V..T....L.Q..LQ.F..I.....K....S...F....EL.L........A.......**

**Pteropus**  **.R.L..VL.....--.....A.HG.GL..R.LN....E......L......S..L...........K........M...FE...........A.....V.**

**Rattus**  **...L...L.F...--.S.Q.A.QGAGL.FR.L.....E..HV..I....L....LQ.FRQI.....K....S...F....EL..........A.......**

**Otolemur**  **...LI..V.....--.....A.HG.QLT.SQ.N....E...........L.S..QQ....V.....K........F.VIFELGV....S....F...A..**

**Gorilla**  **...L...L.....--.....A.HG.GLI.RQ.N....E..E...V.I....QN..W..........E......Q.S..LFE.................V.**

**Ailuropoda**  **.R.LA..L.....--CS.R....GTALT.RQLN....E.............S.MLQ..........K........M...FEL..................**

**Spermophilus** **....I..L..T..--.....A.HG.-L..R..N....E..........H...F.LQ...QI.....K...SS...F..M.EL..................**

**Nomascus**  **...L...L.....--.....A.HG.GLI.TQ.N....E..E.....I....QN..W..........E........F..LFE.................V.**

**Loxodonta**  **...LA..L.-...--.D..LASPS.LL..R..N....E...V.K.......A.VLH....V.....E....SPD..R..FEL.L.....I........V.**

**Sarcophilus**  **.R.L.AFI.....LS.AAARS.GV...PVRD.D...L.V........Q.V.NNHKH...Y....V.KAR.YQ........VL...LV..C.........E**

**Monodelphis**  **.R.L.AFL.....--LSHAAARSH.LLPLRD.D.....V.L..MEITQHV.SQ.QS...YR.....K.R.Y....A....VM.V.M.......M.K...E**

**Pongo**  **...L...L.....--.....A.HG.GLI.RQ.N....E..E...V.I....QNH.W..........E......Q.S..LFE.................V.**

**Callithrix**  **...L...L.....--.....A.QGQGLH.RQLN....EA.E...-......QN..SIQT.LETKTET..KS.Q..F...FVM..........AR....V.**

**Mus**  **...L...L.F...--...Q.A.QGTGL.FR.L.....EA..V..L....L.N..LQ.F.QV.....K....S...F.V..EM.V........A.......**

**Gallus**  **..AL.AFIL.V..---PI.RA--A.A.PPPPLG....EI.A..EF..I...G.SHH..RFA..R.EQ.R.LFQG.NN.ILFL.L.L......I.N....V**

110 120 130 140 150 160 170 180 190 200

....|....|....|....|....|....|....|....|....|....|....|....|....|....|....|....|....|....|....|....|

**Bos**  **NCSVRQQTQHAVEGDCDIHVLKQDGQFSVLFTKCDSSP--DSAEDVRKLCPDCPLLAPLNDSRVVHAVEVALATFNAESNGSYLQLVEISRAQFVPLPVS**

**Ovis**  **........E.............................--....................N.Q....A.........QN....F..............G.**

**Sus**  **......L.E........F.............A......--......H.V..N...............A.S...A...Q...............L...SA.**

**Equus**  **......L..........VRL...N.....S.V..K...--........V.L..........T.......A...A...QN....F.........L......**

**Felis**  **..T...LME........FQ........T...A......GL........V..Q.........TK.........TA..TQ.....F....V....L....P.**

**Canis**  **.....P.M.........FRL.......T...A......--........V..Q.........T......DA..TA...Q.....FR...V....LT...P.**

**Homo**  **R.....LKE........FQL..L..K...VYA......--........V.Q..........T.....AKA...A...QN...NF..E......L....P.**

**Pan**  **R.....LKE........FQL..L..K...VYA......--........V.Q..........T.....AKA...A...QN...NF..E......L....P.**

**Meriones**  **......LA.........F.I.........MH...H.N.--........V..H.A..T.F.S.N..Y..NA..GA..EKN.KT.FK...LA...T..F.P.**

**Pteropus**  **......LQE........FR............A......--......L.V............T.......A...A...RN....Y....V....L..F...**

**Rattus**  **.......AE........F.I.......R..HAQ.H.T.--........F..R..I.IRF..TN...T.KT...A...QN..T.FK........N..F...**

**Otolemur**  **..T...VAE........FR..........V.A......GA........V..Q.....Q...T.......A...A..VQN....F....T....L....P.**

**Gorilla**  **K.....LKE........FQL..L..K...VYA......--........V.Q..........T.....AKA...A...QN...NF..E......L....P.**

**Ailuropoda**  **......LVE........FQ............A......--.......R...G.........TK.....D...TA...Q.....FR.L.V....L....P.**

**Spermophilus** **......VA........KV....LN.K...V.A....T.--........V..I.....Q...T.......A...A..GQK....Y..L......I......**

**Nomascus**  **R.....LKE........FRL..L..K...EYA......--........V.R..........T.....A.A...A...QN...NF..ED.....L....P.**

**Loxodonta**  **..TP..L.E........FQ....G...V...A......--........V..H......F..TK......A...A...Q.....YKI..V....L.L..P.**

**Sarcophilus**  **..T...L.E........VS...V.N..M..NA..E...--........I..N.........TK......A..TA...QKHNN.FK.Q.......S..QS.**

**Monodelphis**  **..T...LAE.......EAS...M...YK..NV..E.N.--........V..Q.S.......T.......AS..A.ISQNQSTFYK.L.V....IS.IQP.**

**Pongo**  **R.....LKE........FKL..L..K...VYA......--........V.Q..........T.....A.A...A...QN.R.HF..E......L....P.**

**Callithrix**  **......L.E........FRL..LN.....FYA..H...--........V.Q..........T.....ADA...A...QN...FF..E.V....L....P.**

**Mus**  **......L.E........F.I.......R.MH.Q.H.T.--...........R....T.F..TN...T.NT...A..TQN..T.FK........N......**

**Gallus**  **.....TFAE........VKLQ.VN......AS..H.HA--.....I.EV..N.....N...TD.LA..SS..NDY.SKNPDV..M.L..G..VKQYH..R**

210 220 230 240 250 260 270 280 290 300

....|....|....|....|....|....|....|....|....|....|....|....|....|....|....|....|....|....|....|....|

**Bos**  **-VSVEFAVAATDCIAKEVVDP-TKCNLLAEK---QYGFCKGSVIQKALGGEDVRVTCTLFQTQPVIPQPQPDG---AEAEAPSAVP--DAAGPTPSAAGP**

**Ovis**  **-....................-.........---...................T.................E.AEAGAPS.VPDAAVP...V.A.....L**

**Sus**  **-........V...V...AYS.-......V..---.......T.TA.-VNE...A....V......VL....------AGADAG.T.VV...ATASPL.DV**

**Equus**  **-.H..........V....I..-A........---......ATLTE.-V.....A....V......VLL...-----DGPDVGVPG.VA..VT.A..P.DL**

**Felis**  **-TY..........V.A..T..-A........---......ATLTE.-V.....A....M......L.....--------DGTE.S.VA.S.VTA.PP.D.**

**Canis**  **-TY..........V.A..T..-A........VHL..EIL.M...R.----CYITI.LL.VIMGHLL.C.SESH----NINC.GYMESATNERIFQPCLPL**

**Homo**  **-TY...T.SG...V...ATEA-A........---......ATLSE.-...AE.A....V......TS....E----GAN..V-PT.VV.PDA.PSPPL.A**

**Pan**  **-TY...T.SG...V...ATEA-A........---......ATLSE.-...AE.A....V......TS....-----EG.NETVPT.VV.PDA.PSPPL.A**

**Meriones**  **-TH...VI.....A.PK.A..-A........---..S...A.LF.N-....E.T....A.P..ANGVT.AS------------PA.AVEKGI.VALP-DA**

**Pteropus**  **-TY...V......VS...T..-A........---......ARLTG.-F.....A...VV......L.....-------EGTN.PT.AETP.K.LSPP.NL**

**Rattus**  **-TL...VI.....TGQ..T..-A........---......ATL.HR-....E.S.A.K......QPANAN.----------AGPA.TVGQ.A.VAPP...**

**Otolemur**  **-TR....I.....V....A..-A........---......AT.TE.-....E.A....V..P..AA.....------ADPTM.VLVAV.PP..AFPP...**

**Gorilla**  **-TY...T.SG...V...ATEA-A........---......ATLSE.-...AE.A....V......TS....-----EG.NEAVPT.VV.PDA.PSPPL.A**

**Ailuropoda**  **-TY..........A..D.T..-A........---......ATLTE.-V.....A....V.....LL.....-----EGPD.VAVD.GA.P.V.V..Q.DV**

**Spermophilus** **-TY..........V....T..-A........---......AT.NE.-V...E.A...........VI....------D.NVANPDAVVEQ.T.ASLP..Q**

**Nomascus**  **-TY...TMSG...V...ATEA-AE.......---......ATLSE.-....E.A....V......TS....-----EG.DEAVPT.VV.PDARPSPPL.A**

**Loxodonta**  **-AY..........V....T..-A........---......ATLTE.-V.....A...MV......V.....-----DGTN.AAPT.AV.P.TAE..P...**

**Sarcophilus**  **-.YT..VTVP...KPID.I..-AA..P.PN----.H.....TLTE.VG.....VA...V.VAV.AV.A....----GIESSLGPAVPAEP.V.AGPPV-A**

**Monodelphis**  **-.Y...VI.P...GP.GAD..-SS.HP.TD----.......T.TK.D-.....A.S..ISVRTL.QISSV.------FGGHEHSSFLLYEMK.A.TPQ.D**

**Pongo**  **-TY...TMSG...V...DTEA-A........---......ATLSE.-....E.A....V......TS....-----EG.NEAVPT.VV.PDA.PSPPL.A**

**Callithrix**  **-TY...T.S.P..V...ATEA-A......K.---......ATLTE.-.N..E.A....V..P..EA.....-----EV.DVVGPK.AA.PDVHV.LPSDT**

**Mus**  **-TL...VI.....T....T..-A........---.H....ANLMHN-....E.S.A.K......---..AN-------.N.VGP..TAN..L.ADPP.--**

**Gallus**  **M..........N.TSQQAK.NLAA.Q..P.D-QSNF...TAKMVTE--PSQ.LIAE.Q.YGH..GVTY.H.GQ----------D-----TSAGLVPS..Q**

310 320 330 340 350 360 370 380

....|....|....|....|....|....|....|....|....|....|....|....|....|....|....|....|....|

**Bos**  **PVASVVVGP----SVVAVP---LPLHRAHYDLRHTFSGVASVESSSGEAFHVGKTPIVGQPSIPG--GP---VRLCPGRIRYFKI**

**Ovis**  **..G...A..----......---......................A.................V..--..---.H...........**

**Sus**  **.A..L....----M.....PGIP.V..S......S.........A...........KGA.....AAD.SVPV..P......H...**

**Equus**  **....L....------.VL.P---.V.........A.A..G.G..A.......E.P.K.AH.NTAAAA..--V..P..........**

**Felis**  **.A.AL....----V...A.QAP..G.........A.M.......A....V....V.N.A...V.VAA..--V..P......H...**

**Canis**  **.R..GFSC.GGRTR...F.EAP..E.........A.M.......A.........V.K.V..DVAVAA..--V.HP......H...**

**Homo**  **.GLPPAGS.PDSHVLL.A.PG-HQ............M..V.LG.P...VS.PR..RT.V...VGAAA..--V.PP......H..V**

**Pan**  **.GLPPAGS.PDSHVLL.A.PG-HQ..W.........M..V.LG.P....S.PR..RT.V...VGAAA..--V.PP......H..V**

**Meriones**  **.P..L....------MV..AEH..-.KT.H....A..P......A.....Q-----SPT.AGNA.AA..--A.P.....V.H...**

**Pteropus**  **.A.T.....----L.A.A.PPS..V.........A.G.......A.....L.E.A.T.V..GVAVAA..--V..P......H...**

**Rattus**  **.-E......------....LG-..D..T.H....A..P......A...VL.---S.K....GDA.AA..--VAP.....V.....**

**Otolemur**  **.A.AM....-----M.VAARPGP....T......A......A..A......P..A.G.T..GVA.AV..M-VLPP....V.H..V**

**Gorilla**  **.GLPPAGL.PDSHVLL.A.PG-HQ..W.........M..V.LG.P....S.PR..RT.V...VGAAA..--V.PP......H..V**

**Ailuropoda**  **....L....-------V..EAP..G..T.....RA.T..V.E..A....V..E.G.T.V..G.AVVPD.--AL.P.........V**

**Spermophilus** **.A..Q....-----M.VS.PRGP.S..V.....YA.........A......P...----..GVA.AAD.--M..P......H...**

**Nomascus**  **.GPPPAGL.PHSHVLA.A.QA-..S.........V.M..VRLG.PL...L.PQ..HP.V...VGAAA..--V.PP......H..V**

**Loxodonta**  **...A.....-----L.LAAPQGP...........A.T..V....A......PV.P.V.VG.------..--R.HS.........V**

**Sarcophilus**  **AGPA.AL..LLVDAPAVL.AKPILT.LP.H....SIV.GS.F..A...-YGLNAG.VAPG.GPGPAAP---AIP.......H...**

**Monodelphis**  **I.RIQYCV.APVG-P.VR.VRPFIS.LP.H....SIV....L..A...H---.PVVGLAA.AV..AAA.--G.P.......H..V**

**Pongo**  **.GPPPAGS.PYSHVLA.ASPA-HQ.Y..........M..V.WR.L...VS.PR..RT.V...VGAAA..--V.PP......H..V**

**Callithrix**  **AANPQASP.-----LRR.APQV..W.........I.L.GV.M..T....W.LR..R-----AVGAAAA.--A.P.......H..V**

**Mus**  **---......-------V.VPRG.SD..TYH....A..P......A...TL.---S.K....G---AA..--VSPM......H...**

**Gallus**  **GFTNHN--------LKISHNNPVASESSSSEFPSLL.AKSVAKRAAA.VAQHD.V.---H.-VGFVPP.----P....K..H...**
